# Diretrizes Brasileiras de Hipertensão Arterial – 2020

**DOI:** 10.36660/abc.20201238

**Published:** 2021-03-03

**Authors:** Weimar Kunz Sebba Barroso, Cibele Isaac Saad Rodrigues, Luiz Aparecido Bortolotto, Marco Antônio Mota-Gomes, Andréa Araujo Brandão, Audes Diógenes de Magalhães Feitosa, Carlos Alberto Machado, Carlos Eduardo Poli-de-Figueiredo, Celso Amodeo, Décio Mion, Eduardo Costa Duarte Barbosa, Fernando Nobre, Isabel Cristina Britto Guimarães, José Fernando Vilela-Martin, Juan Carlos Yugar-Toledo, Maria Eliane Campos Magalhães, Mário Fritsch Toros Neves, Paulo César Brandão Veiga Jardim, Roberto Dischinger Miranda, Rui Manuel dos Santos Póvoa, Sandra C Fuchs, Alexandre Alessi, Alexandre Jorge Gomes de Lucena, Alvaro Avezum, Ana Luiza Lima Sousa, Andrea Pio-Abreu, Andrei Carvalho Sposito, Angela Maria Geraldo Pierin, Annelise Machado Gomes de Paiva, Antonio Carlos de Souza Spinelli, Armando da Rocha Nogueira, Nelson Dinamarco, Bruna Eibel, Cláudia Lúcia de Moraes Forjaz, Claudia Regina de Oliveira Zanini, Cristiane Bueno de Souza, Dilma do Socorro Moraes de Souza, Eduardo Augusto Fernandes Nilson, Elisa Franco de Assis Costa, Elizabete Viana de Freitas, Elizabeth da Rosa Duarte, Elizabeth Silaid Muxfeldt, Emilton Lima, Erika Maria Gonçalves Campana, Evandro José Cesarino, Fabiana Marques, Fábio Argenta, Fernanda Marciano Consolim-Colombo, Fernanda Spadotto Baptista, Fernando Antonio de Almeida, Flávio Antonio de Oliveira Borelli, Flávio Danni Fuchs, Frida Liane Plavnik, Gil Fernando Salles, Gilson Soares Feitosa, Giovanio Vieira da Silva, Grazia Maria Guerra, Heitor Moreno, Helius Carlos Finimundi, Isabela de Carlos Back, João Bosco de Oliveira, João Roberto Gemelli, José Geraldo Mill, José Marcio Ribeiro, Leda A. Daud Lotaif, Lilian Soares da Costa, Lucélia Batista Neves Cunha Magalhães, Luciano Ferreira Drager, Luis Cuadrado Martin, Luiz César Nazário Scala, Madson Q. Almeida, Marcia Maria Godoy Gowdak, Marcia Regina Simas Torres Klein, Marcus Vinícius Bolívar Malachias, Maria Cristina Caetano Kuschnir, Maria Eliete Pinheiro, Mario Henrique Elesbão de Borba, Osni Moreira, Oswaldo Passarelli, Otavio Rizzi Coelho, Priscila Valverde de Oliveira Vitorino, Renault Mattos Ribeiro, Roberto Esporcatte, Roberto Franco, Rodrigo Pedrosa, Rogerio Andrade Mulinari, Rogério Baumgratz de Paula, Rogério Toshiro Passos Okawa, Ronaldo Fernandes Rosa, Sandra Lia do Amaral, Sebastião R. Ferreira-Filho, Sergio Emanuel Kaiser, Thiago de Souza Veiga Jardim, Vanildo Guimarães, Vera H. Koch, Wille Oigman, Wilson Nadruz

**Affiliations:** 1 Universidade Federal de Goiás Universidade Federal de Goiás Goiânia GO Brasil Universidade Federal de Goiás , Goiânia , GO – Brasil; 2 Liga de Hipertensão Arterial Liga de Hipertensão Arterial Goiânia GO Brasil Liga de Hipertensão Arterial , Goiânia , GO – Brasil; 3 Pontifícia Universidade Católica de São Paulo Faculdade de Ciências Médicas e da Saúde Sorocaba SP Brasil Pontifícia Universidade Católica de São Paulo , Faculdade de Ciências Médicas e da Saúde , Sorocaba , SP – Brasil; 4 Instituto do Coração São Paulo SP Brasil Instituto do Coração (InCor), São Paulo , SP – Brasil; 5 Centro Universitário CESMAC Maceió AL Brasil Centro Universitário CESMAC , Maceió , AL – Brasil; 6 Faculdade de Ciências Médicas Universidade do Estado do Rio de Janeiro Rio de Janeiro RJ Brasil Faculdade de Ciências Médicas da Universidade do Estado do Rio de Janeiro (FCM-UERJ), Rio de Janeiro , RJ – Brasil; 7 Universidade Federal de Pernambuco Recife PE Brasil Universidade Federal de Pernambuco , Recife , PE – Brasil; 8 Pronto Socorro Cardiológico de Pernambuco Recife PE Brasil Pronto Socorro Cardiológico de Pernambuco (PROCAPE), Recife , PE – Brasil; 9 Secretaria Municipal de Saúde de Campos do Jordão Campos do Jordão SP Brasil Secretaria Municipal de Saúde de Campos do Jordão , Campos do Jordão , SP – Brasil; 10 Pontifícia Universidade Católica do Rio Grande do Sul Porto Alegre RS Brasil Pontifícia Universidade Católica do Rio Grande do Sul , Porto Alegre , RS – Brasil; 11 Universidade Federal de São Paulo São Paulo SP Brasil Universidade Federal de São Paulo (UNIFESP), São Paulo , SP – Brasil; 12 Hospital das Clínicas da Faculdade de Medicina da USP São Paulo SP Brasil Hospital das Clínicas da Faculdade de Medicina da USP , São Paulo , SP – Brasil; 13 Serviço Hipertensão e Cardiometabolismo da Santa Casa de Porto Alegre Porto Alegre RS Brasil Serviço Hipertensão e Cardiometabolismo da Santa Casa de Porto Alegre , Porto Alegre , RS – Brasil; 14 Hospital das Clínicas da Faculdade de Medicina de Ribeirão Preto da Universidade de São Paulo Ribeirão Preto SP Brasil Hospital das Clínicas da Faculdade de Medicina de Ribeirão Preto da Universidade de São Paulo , Ribeirão Preto , SP – Brasil; 15 Hospital São Francisco Ribeirão Preto SP Brasil Hospital São Francisco , Ribeirão Preto , SP – Brasil; 16 Universidade Federal da Bahia Salvador BA Brasil Universidade Federal da Bahia (UFBA), Salvador , BA – Brasil; 17 Faculdade Estadual de Medicina de São José do Rio Preto São José do Rio Preto SP Brasil Faculdade Estadual de Medicina de São José do Rio Preto , São José do Rio Preto , SP – Brasil; 18 Hospital Universitário Pedro Ernesto da Universidade do Estado do Rio de Janeiro Rio de Janeiro RJ Brasil Hospital Universitário Pedro Ernesto da Universidade do Estado do Rio de Janeiro (UERJ), Rio de Janeiro , RJ – Brasil; 19 Hospital do Coração de Goiás Goiânia GO Brasil Hospital do Coração de Goiás , Goiânia , GO – Brasil; 20 Faculdade de Medicina da Universidade Federal do Rio Grande do Sul Porto Alegre RS Brasil Faculdade de Medicina da Universidade Federal do Rio Grande do Sul (UFRGS), Porto Alegre , RS – Brasil; 21 Universidade Federal do Paraná Curitiba PR Brasil Universidade Federal do Paraná (UFPR), Curitiba , PR – Brasil; 22 Hospital Agamenom Magalhães Recife PE Brasil Hospital Agamenom Magalhães , Recife , PE – Brasil; 23 Hospital Alemão Oswaldo Cruz São Paulo SP Brasil Hospital Alemão Oswaldo Cruz , São Paulo , SP – Brasil; 24 Universidade de São Paulo São Paulo SP Brasil Universidade de São Paulo (USP), São Paulo , SP – Brasil; 25 Universidade Estadual de Campinas Campinas SP Brasil Universidade Estadual de Campinas (UNICAMP), Campinas , SP – Brasil; 26 Cardiocentro Brasil Cardiocentro , Natal, RN – Brasil; 27 Universidade Federal do Rio de Janeiro Rio de Janeiro RJ Brasil Universidade Federal do Rio de Janeiro (UFRJ), Rio de Janeiro , RJ – Brasil; 28 Universidade Estadual de Santa Cruz Ilhéus BA Brasil Universidade Estadual de Santa Cruz , Ilhéus , BA – Brasil; 29 Instituto de Cardiologia Fundação Universitária de Cardiologia Porto Alegre RS Brasil Instituto de Cardiologia , Fundação Universitária de Cardiologia (IC/FUC), Porto Alegre , RS – Brasil; 30 Centro Universitário da Serra Gaúcha Caxias do Sul RS Brasil Centro Universitário da Serra Gaúcha (FSG), Caxias do Sul , RS – Brasil; 31 Universidade Federal do Pará Belém PA Brasil Universidade Federal do Pará (UFPA), Belém , PA – Brasil; 32 Ministério da Saúde Brasília DF Brasil Ministério da Saúde , Brasília , DF – Brasil; 33 Departamento de Cardiogeriatria da Sociedade Brazileira de Cardiologia Rio de Janeiro RJ Brasil Departamento de Cardiogeriatria da Sociedade Brazileira de Cardiologia , Rio de Janeiro , RJ – Brasil; 34 Hospital Nossa Senhora da Conceição Tubarão SC Brasil Hospital Nossa Senhora da Conceição (HNSC), Tubarão , SC – Brasil; 35 Universidade Estácio de Sá Rio de Janeiro RJ Brasil Universidade Estácio de Sá (UNESA), Rio de Janeiro , RJ – Brasil; 36 Hospital de Clínicas da Universidade Federal do Paraná Curitiba PR Brasil Hospital de Clínicas da Universidade Federal do Paraná (HC/UFPR), Curitiba , PR – Brasil; 37 Universidade Iguaçu Rio de Janeiro RJ Brasil Universidade Iguaçu (UNIG), Rio de Janeiro , RJ – Brasil; 38 Faculdade de Ciências Farmacêuticas de Ribeirão Preto da Universidade de São Paulo Ribeirão Preto SP Brasil Faculdade de Ciências Farmacêuticas de Ribeirão Preto da Universidade de São Paulo , Ribeirão Preto , SP – Brasil; 39 Associação Ribeirãopretana de Ensino, Pesquisa e Assistência ao Hipertenso Ribeirão Preto SP Brasil Associação Ribeirãopretana de Ensino, Pesquisa e Assistência ao Hipertenso (AREPAH), Ribeirão Preto , SP – Brasil; 40 Faculdade de Medicina de Ribeirão Preto da Universidade de São Paulo Ribeirão Preto SP Brasil Faculdade de Medicina de Ribeirão Preto da Universidade de São Paulo , Ribeirão Preto , SP – Brasil; 41 Mediodonto Cuiabá MT Brasil Mediodonto , Cuiabá , MT – Brasil; 42 Programa de Pós-Graduação em Medicina da Universidade Nove de Julho São Paulo SP Brasil Programa de Pós-Graduação em Medicina da Universidade Nove de Julho (UNINOVE), São Paulo , SP – Brasil; 43 Instituto Dante Pazzanese de Cardiologia São Paulo SP Brasil Instituto Dante Pazzanese de Cardiologia , São Paulo , SP – Brasil; 44 Hospital de Clínicas de Porto Alegre Porto Alegre RS Brasil Hospital de Clínicas de Porto Alegre , Porto Alegre , RS – Brasil; 45 Escola Bahiana de Medicina e Saúde Pública Salvador BA Brasil Escola Bahiana de Medicina e Saúde Pública , Salvador , BA – Brasil; 46 Universidade Santo Amaro São Paulo SP Brasil Universidade Santo Amaro (UNISA), São Paulo , SP – Brasil; 47 Universidade de Caxias do Sul Caxias do Sul RS Brasil Universidade de Caxias do Sul (UCS), Caxias do Sul , RS – Brasil; 48 Universidade Federal de Santa Catarina Florianópolis SC Brasil Universidade Federal de Santa Catarina (UFSC), Florianópolis , SC – Brasil; 49 Hospital Israelita Albert Einstein São Paulo SP Brasil Hospital Israelita Albert Einstein , São Paulo , SP – Brasil; 50 Clínica Gemelli Porto Velho RO Brasil Clínica Gemelli , Porto Velho , RO – Brasil; 51 Centro de Ciências da Saúde Universidade Federal do Espírito Santo Vitória ES Brasil Centro de Ciências da Saúde , Universidade Federal do Espírito Santo , Vitória , ES – Brasil; 52 Faculdade Ciências Médicas de Minas Gerais Belo Horizonte MG Brasil Faculdade Ciências Médicas de Minas Gerais , Belo Horizonte , MG – Brasil; 53 Hospital Felício Rocho Belo Horizonte MG Brasil Hospital Felício Rocho , Belo Horizonte , MG – Brasil; 54 Hospital do Coração São Paulo SP Brasil Hospital do Coração (HCor), São Paulo , SP – Brasil; 55 Instituto Estadual de Cardiologia Aloysio de Castro Rio de Janeiro RJ Brasil Instituto Estadual de Cardiologia Aloysio de Castro , Rio de Janeiro , RJ – Brasil; 56 Centro Universitário de Tecnologia e Ciência Salvador BA Brasil Centro Universitário de Tecnologia e Ciência (UniFTC), Salvador , BA – Brasil; 57 Universidade Estadual Paulista Bauru SP Brasil Universidade Estadual Paulista (UNESP), Bauru , SP – Brasil; 58 Faculdade de Medicina da Universidade Federal de Mato Grosso Cuiabá MT Brasil Faculdade de Medicina da Universidade Federal de Mato Grosso , Cuiabá , MT – Brasil; 59 Instituto Vera Cruz São Paulo SP Brasil Instituto Vera Cruz , São Paulo , SP – Brasil; 60 Sociedade Brazileira de Nefrologia São Paulo SP Brasil Sociedade Brazileira de Nefrologia , São Paulo , SP – Brasil; 61 Cardio Clínica do Vale Lajeado RS Brasil Cardio Clínica do Vale , Lajeado , RS – Brasil; 62 Pontifícia Universidade Católica do Paraná Curitiba PR Brasil Pontifícia Universidade Católica do Paraná , Curitiba , PR – Brasil; 63 Pontifícia Universidade Católica de Goiás Goiânia GO Brasil Pontifícia Universidade Católica de Goiás , Goiânia , GO – Brasil; 64 Cardios Vita Centro de Medicina Cardiológica Brasília DF Brasil Cardios Vita Centro de Medicina Cardiológica , Brasília , DF – Brasil; 65 Hospital Pró-Cradíaco Rio de Janeiro RJ Brasil Hospital Pró-Cradíaco , Rio de Janeiro , RJ – Brasil; 66 Universidade Federal de Juiz de Fora Juiz de Fora MG Brasil Universidade Federal de Juiz de Fora , Juiz de Fora , MG – Brasil; 67 Avancor Cardiologia Maringá PR Brasil Avancor Cardiologia , Maringá , PR – Brasil; 68 Universidade Estadual de Maringá Maringá PR Brasil Universidade Estadual de Maringá , Maringá , PR – Brasil; 69 Faculdade de Ciências Médicas da Santa Casa de São Paulo São Paulo SP Brasil Faculdade de Ciências Médicas da Santa Casa de São Paulo , São Paulo , SP – Brasil; 70 Universidade Federal de Uberlândia Uberlândia MG Brasil Universidade Federal de Uberlândia , Uberlândia , MG – Brasil; 71 Hospital Getúlio Vargas Recife PE Brasil Hospital Getúlio Vargas , Recife , PE – Brasil

**Table:** 

Declaração de potencial conflito de interesses dos autores/colaboradores da Diretrizes Brazileiras de Hipertensão Arterial – 2020							
Se nos últimos 3 anos o autor/colaborador da Diretriz:							
Nomes Integrantes da Diretriz	Participou de estudos clínicos e/ou experimentais subvencionados pela indústria farmacêutica ou de equipamentos relacionados à diretriz em questão	Foi palestrante em eventos ou atividades patrocinadas pela indústria relacionados à diretriz em questão	Foi (é) membro do conselho consultivo ou diretivo da indústria farmacêutica ou de equipamentos	Participou de comitês normativos de estudos científicos patrocinados pela indústria	Recebeu auxílio pessoal ou institucional da indústria	Elaborou textos científicos em periódicos patrocinados pela indústria	Tem ações da indústria
Alexandre Alessi	Não	Não	Não	Não	Não	Não	Não
Alexandre Jorge Gomes de Lucena	Não	Não	Não	Não	Não	Não	Não
Alvaro Avezum	Não	Não	Não	Não	Não	Não	Não
Ana Luiza Lima Sousa	Não	Não	Não	Não	Não	Não	Não
Andréa Araujo Brandão	Não	Abbott, Daiichi Sankyo, EMS, Libbs, Novartis, Medley, Merck, Servier	Não	Não	Servier	Abbott, Daiichi Sankyo, EMS, Libbs, Novartis, Medley, Merck, Servier	Não
Andrea Pio-Abreu	Não	Não	Não	Não	Não	Não	Não
Andrei Carvalho Sposito	Não	Não	Não	Não	Não	Não	Não
Angela Maria Geraldo Pierin	Não	Não	Não	Não	Não	Não	Não
Annelise Machado Gomes de Paiva	Não	Não	Não	Não	Não	Não	Não
Antonio Carlos de Souza Spinelli	Não	Merck, Torrent, Boerhinger, Sandoz	Não	Não	EMS, Aché, Torrent	Não	Não
Armando da Rocha Nogueira	Não	Não	Não	Não	Não	Não	Não
Audes Diógenes de Magalhães Feitosa	Não	EMS, Servier, Sandoz, Merck, Medtronic e Omron	Omron	Não	Não	EMS, Servier e Omron	Não
Bruna Eibel	Não	Não	Não	Não	Não	Não	Não
Carlos Alberto Machado	Não	Não	Biolab, Omron	Não	Não	Não	Não
Carlos Eduardo Poli-de-Figueiredo	Não	Não	Não	Fresenius	Centro de Pesquisa Clínico da PUCRS, Baxter, Fresenius, Alexion, AstraZeneca.	Não	Não
Celso Amodeo	Não	Novartis, NovoNordisk, EMS, ACHE	Montecorp Farmasa	Não	Não	ACHE, Montecorp Farmasa	Não
Cibele Isaac Saad Rodrigues	Não	Não	Não	Não	Não	Não	Não
Cláudia Lúcia de Moraes Forjaz	Não	Não	Não	Não	Não	Não	Não
Claudia Regina de Oliveira Zanini	Não	Não	Não	Não	Não	Não	Não
Cristiane Bueno de Souza	Não	Não	Não	Não	Não	Não	Não
Decio Mion Junior	Não	Zodiac	Não	Não	Não	Zodiac	Não
Dilma do Socorro Moraes de Souza	Não	Não	Não	Não	Não	Não	Não
Eduardo Augusto Fernandes Nilson	Não	Não	Não	Não	Não	Não	Não
Eduardo Costa Duarte Barbosa	Não	Servier, EMS	Não	Não	Não	Não	Não
Elisa Franco de Assis Costa	Não	Não	Não	Não	Abbot Nutrition, Nestlé Health Sciences, Aché, Sandoz, Nutricia	Abboot Nutrition	Não
Elizabete Viana de Freitas	Não	Não	Não	Não	Não	Não	Não
Elizabeth da Rosa Duarte	Não	Não	Não	Não	Sim, Ache, Bayer, Novartis, Torrent, Servier.	Não	Não
Elizabeth Silaid Muxfeldt	Não	Não	Não	Não	Não	Não	Não
Emilton Lima Júnior	Não	Servier, Novo Nordisk, Bayer, Biolab, Amgem	Não	Servier	Não	Não	Não
Erika Maria Gonçalves Campana	Não	Não	Não	Não	Servier	Servier	Não
Evandro José Cesarino	Não	Não	Não	Não	Não	Não	Não
Fábio Argenta	Não	Não	Não	Não	Novartis, Bayer, Torrent, Lilly, Boehringer	Não	Não
Fernanda Marciano Consolim-Colombo	Não	Merck, Ache, Daiichi	Não	Não	Não	Não	Não
Fernanda Spadotto Baptista	Não	Não	Não	Não	Não	Não	Não
Fernando Antonio de Almeida	Não	Não	Não	Não	Não	Não	Não
Fernando Nobre	Não	Libbs, Cristália	Não	Não	Libbs, Novartis, Servier, Baldacci	Daichi Sankio, Libbs, Novartis, Biolab, Servier, Baldacci	Não
Flávio Antonio de Oliveira Borelli	Não	Não	Não	Não	Não	Não	Não
Flávio Danni Fuchs	Não	Não	Não	Não	Não	Não	Não
Frida Liane Plavnik	Não	Não	Não	Não	Não	Não	Não
Gil Fernando Salles	Não	Não	Não	Não	Não	Não	Não
Gilson Soares Feitosa	Não	Não	Não	Não	Não	Não	Não
Giovanio Vieira da Silva	Não	Ache	Não	Não	Não	Ache	Não
Grazia Guerra	Não	Não	Não	Não	Não	Não	Não
Heitor Moreno Júnior	Não	Não	Não	Não	Não	Não	Não
Helius Carlos Finimundi	Não	Não	Não	Não	Não	Não	Não
Isabel Cristina Britto Guimarães	Não	Não	Não	Não	Não	Não	Não
Isabela de Carlos Back	Não	Não	Não	Não	Não	Não	Não
João Bosco de Oliveira Filho	Não	Não	Não	Não	Novartis, Bristol, AztraZeneca	Não	Não
João Roberto Gemelli	Não	Não	Não	Não	Boeringher, Libbs	Não	Boeringher, Libbs
José Fernando Vilela-Martin	Não	Não	Não	Não	Não	Não	Não
Jose Geraldo Mill	Não	Não	Não	Não	Não	Não	Não
José Marcio Ribeiro	Não	Não	Não	Não	Não	Não	Não
Juan Carlos Yugar-Toledo	Não	Não	Não	Não	Não	Não	Não
Leda A. Daud Lotaif	Não	Não	Não	Não	Não	Não	Não
Lilian Soares da Costa	Não	Não	Não	Não	Não	Não	Não
Lucélia Batista Neves Cunha Magalhães	Não	Não	Não	Não	Não	Não	Não
Luciano Ferreira Drager	Não	Aché, Biolab, Boehringer, Merck	ResMed	Não	Não	Aché, Biolab, Merck	Não
Luis Cuadrado Martin	Não	Não	Não	Não	Não	Não	Não
Luiz Aparecido Bortolotto	Não	Servier, Novonordisk	Não	Não	Não	Não	Não
Luiz César Nazário Scala	Não	Não	Não	Não	Não	Não	Não
Madson Q. Almeida	Não	Não	Não	Não	Não	Não	Não
Marcia Maria Godoy Gowdak	Não	Não	Não	Não	Não	Não	Não
Marcia Regina Simas Torres Klein	Não	Não	Não	Não	Não	Não	Não
Marco Antônio Mota-Gomes	Omron, Beliva	Não	Omron, Libbs	Não	Não	Omron, Libbs	Não
Marcus Vinícius Bolívar Malachias	Não	Libbs, Biolab	Não	Não	Não	Libbs. Biolab	Não
Maria Cristina Caetano Kuschnir	Não	Não	Não	Não	Não	Não	Não
Maria Eliane Campos Magalhães	Não	Não	Não	Não	Não	Não	Não
Maria Eliete Pinheiro	Não	Não	Não	Não	Não	Não	Não
Mario Fritsch Toros Neves	Não	Servier	Não	Não	Não	Não	Não
Mario Henrique Elesbão de Borba	Não	EMS	Não	Não	Não	Não	Não
Nelson Dinamarco Ludovico	Não	Não	Não	Não	Não	Não	Não
Osni Moreira Filho	Não	Biolab, Servier	Não	Não	Não	Não	Não
Oswaldo Passarelli Júnior	Não	Não	Não	Não	Não	Não	Não
Otávio Rizzi Coelho	Não	Daichi-Sankyo, Boehringer	Não	Daichi-Sankyo, BAYER, Novo-Nordisk	Não	Sanofi, Takeda, AstraZeneca, Daichi-Sankyo, Bayer	Não
Paulo César Brandão Veiga Jardim	Não	Servier, Libbs, EMS	Não	Não	Não	Servier, Libbs	Não
Priscila Valverde Vitorino	Não	Não	Não	Não	Não	Não	Não
Renault Mattos Ribeiro Júnior	Não	Daiichi Sankyo	Não	Não	Não	Não	Não
Roberto Dischinger Miranda	Não	EMS, Boehringher	Não	Não	Não	EMS, Sanofi, Servier	Não
Roberto Esporcatte	Não	EMS	Não	Não	Não	Não	Não
Roberto Franco	Não	Não	Não	Não	Não	Não	Não
Rodrigo Pedrosa	Não	Não	Não	Não	Não	Não	Não
Rogerio Andrade Mulinari	Não	Não	Não	Não	Não	Não	Não
Rogério Baumgratz de Paula	Não	Não	Não	Não	Não	Não	Não
Rogerio Toshiro Passos Okawa	Não	Não	Não	Não	Não	Não	Não
Ronaldo Fernandes Rosa	Não	Não	Não	Não	Não	Não	Não
Rui Manuel dos Santos Póvoa	Não	Não	Não	Não	Não	Não	Não
Sandra Fuchs	Não	Não	Não	Não	Não	Não	Não
Sandra Lia do Amaral	Não	Não	Não	Não	Não	Não	Não
Sebastião R. Ferreira-Filho	Não	Não	Não	Não	Não	Não	Não
Sergio Emanuel Kaiser	Engage, Alecardio, RED-HF, Odissey-Outcomes, SELECT	Amgen, Novo Nordisk, Novartis, astrazeneca, Momenta Farma, Daiichi-Sankyo, Pfizer, Baldacci	Não	Não	Não	Novartis, Momenta Farma, Farmasa, EMS	Não
Thiago de Souza Veiga Jardim	Não	AstraZeneca, Torrent, Meck, Bayer	Não	Não	Não	Não	Não
Vanildo Guimarães	Não	Boehringer, Novartis, Sandoz	Não	Não	Não	Não	Não
Vera H. Koch	Não	Não	Não	Não	Não	Não	Não
Weimar Kunz Sebba Barroso de Souza	Ministério da Saúde, Sociedade Europeia de Hipertensão Arterial, Artery Society, EMS	EMS, Libbs, Sandoz, Servier, Cardios, Omron	Omron	Não	EMS, Servier	EMS, Servier, Omron	Não
Wille Oigman	Não	Não	Não	Não	Não	Não	Não
Wilson Nadruz	Não	Não	Não	Não	Não	Não	Não

**Table:** Lista de siglas

AAS	ácido acetilsalicílico	FEO	feocromocitona
ACS	agente comunitário de saúde	FEp	fração de ejeção preservada
AGA	avaliação geriátrica ampla	FEr	fração de ejeção reduzida
Aix	*augmentation index*	FR	fatores de risco
AMPA	automedida da pressão arterial	FRCV	fator de risco cardiovascular
AOS	apneia obstrutiva do sono	GBD	*global burden diseases* (carga global de doenças)
APA	adenomas produtores de aldosterona	GH	hormônio de crescimento
ARP	atividade da renina plasmática	GR	grau de recomendação
AVE	acidente vascular encefálico	HA	hipertensão arterial
AVEH	acidente vascular encefálico hemorrágico	HAB	hipertensão do avental branco
AVEI	acidente vascular encefálico isquêmico	HAR	hipertensão arterial resistente
AVD	atividade de vida diária	HARf	hipertensão arterial refratária
BB	betabloqueadores	HARV	hipertensão arterial renovascular
BCC	bloqueador do canal de cálcio	HDL	high density lipoprotein (lipoproteína de alta densidade)
BRA	bloqueador do receptor AT1 da angiotensina II	HELPP	*hemolysis, elevated liver enzymes, low platelets*
CB	circunferência do braço	HM	hipertensão mascarada
CH	crise hipertensiva	HO	hipotensão ortostática
CV	cardiovascular	HPLC	*high performance liquid cromatography* (cromatografia líquida de alta performance)
DAC	doença arterial coronária	HPP	hipotensão pós-prandial
DALYs	anos de vida ajustados para incapacidade	HS	hipertensão sustentada
DAOP	doença arterial obstrutiva periférica	hs-TnT	troponina T de alta sensibilidade
DC	débito cardíaco	IAM	infarto agudo do miocárdio
DCbV	doença cerebrovascular	IC	insuficiência cardíaca
DCNT	doença crônica não transmissível	ICFEP	insuficiência cardíaca com fração de ejeção preservada
DCV	doença cardiovascular	ICFER	insuficiência cardíaca com fração de ejeção reduzida
DFM	displasia fibromuscular	IECA	inibidor da enzima conversora de angiotensina
DIU	diuréticos	IGF-1	*insulin-like growth factor-I*
DM	diabetes melito	IMC	índice de massa corporal
DMF	dilatação mediada pelo fluxo	ITB	índice tornozelo braquial
DRC	doença renal crônica	IV	intravenosa
E/R	espiritualidade ou religiosidade	LOA	lesão de órgãos-alvo
EA	evento adverso	MAPA	medida ambulatorial da pressão arterial
EAB	efeito do avental branco	MDO	múltiplos danos a órgãos-alvo
EAP	edema agudo de pulmão	MDPAC	medida desacompanhada da pressão arterial no consultório
EAR	estenose de artéria renal	MEV	mudança de estilo de vida
ECR	ensaio clínico randomizado	MMII	membros inferiores
EH	emergência hipertensiva	MRPA	medida residencial da pressão arterial
EM	efeito de mascaramento	NA	noradrenalina
eNOS	produção de óxido nítrico pela sintase endotelial	NE	nível de evidência
ERG	escore de risco global	NIHSS	National Institute of Health Stroke Scale
FA	fibrilação atrial	NO	óxido nítrico
FC	frequência cardíaca	NOO-	peroxinitrito
FE	fração de ejeção	NPS	nitroprussiato de sódio
NT-proBNP	N-terminal pro-peptídio natriurético do tipo B	SM	síndrome metabólica
NTG	nitroglicerina	SNPS	sistema nervoso parassimpático
NV	normotensão verdadeira	SNS	sistema nervoso simpático
OMS	Organização Mundial da Saúde	SRAA	sistema renina-angiotensina aldosterona
ONU	Organização das Nações Unidas	SUS	Sistema Único de Saúde
PA	pressão arterial	T4	tiroxina
PAD	pressão arterial diastólica	TC	tomografia computadorizada
PAE	pressão arterial elevada	TG	triglicerídeos
PAS	pressão arterial sistólica	TNM	tratamento não medicamentoso
PCH	pseudocrise hipertensiva	TSH	hormônio tireotrófico
PCR	proteína C-reativa	UH	urgência hipertensiva
PE	pré-eclâmpsia	UTI	unidade de terapia intensiva
PNS	pesquisa nacional de saúde	VM	teste funcional de velocidade de marcha
RFG-e	ritmo de filtração glomerular estimado	VOP	velocidade de onda de pulso
RN	recém-nascido	VOPcf	velocidade de onda de pulso carotídeo femural
RNM	ressonância nuclear magnética	YLL	*years lost of life* (anos de vida perdidos)
RVP	resistência vascular periférica		

## Sumário

1. Definição, Epidemiologia e Prevenção Primária 528

1.1 Definição de Hipertensão Arterial 528

1.2. Impacto da Hipertensão Arterial nas Doenças Cardiovasculares 528

1.3. Fatores de Risco para Hipertensão Arterial 528

**1.3.1. Genética** 528

**1.3.2. Idade** 528

**1.3.3. Sexo** 528

**1.3.4. Etnia** 528

**1.3.5. Sobrepeso/Obesidade** 528

**1.3.6. Ingestão de Sódio e Potássio** 529

**1.3.7. Sedentarismo** 529

**1.3.8. Álcool** 529

**1.3.9. Fatores Socioeconômicos** 529

**1.3.10. Outros Fatores de Risco Relacionados com a Elevação da PA**

**1.3.11. Apneia Obstrutiva do Sono (AOS)** 529

**1.3.12. Dados Epidemiológicos Globais** 529

1.4. Prevalência de Hipertensão Arterial no Brazil 530

1.5. Prevenção Primária 530

**1.5.1. Introdução** 530

**1.5.2. Controle do Peso (GR: I; NE: A)** 530

**1.5.3. Dieta Saudável (GR: I; NE: A)** 530

**1.5.4. Sódio (GR: I; NE: A)** 530

**1.5.5. Potássio (GR: I; NE: A)** 531

**1.5.6. Atividade Física (GR: I; NE: A)** 531

**1.5.7. Álcool (GR: IIA; NE: B)** 531

**1.5.8. Fatores Psicossociais (GR: IIb; NE: B)** 531

**1.5.9. Suplementos Alimentares (GR: I a III; NE: A e B)** 531

**1.5.10. Tabagismo (GR: I; NE: A)** 531

**1.5.11. Espiritualidade (GR: I; NE: B)** 531

1.6. Estratégias para a Implementação de Medidas Preventivas 532

2. Pressão Arterial e Dano Vascular 535

2.1. Introdução 535

2.2. Pressão Arterial, Desfechos Clínicos e Dano Cardiovascular 535

2.3. Pressão Arterial, Inflamação e Disfunção Endotelial 536

2.4. Pressão Arterial e Rigidez Arterial 536

**2.4.1. Índice Tornozelo-Braquial (ITB)** 537

**2.4.2. Velocidade de Onda de Pulso (VOP)** 537

**2.4.3. Pressão Central** 537

3. Diagnóstico e Classificação 540

3.1. Introdução 540

3.2. Medida da Pressão Arterial no Consultório 540

3.3. Classificação 540

3.4. Medida da Pressão Arterial Fora do Consultório 541

3.5. Efeito do Avental Branco (EAB) e Efeito de Mascaramento (EM) 541

3.6. Hipertensão do Avental Branco (HAB) e Hipertensão Mascarada (HM) 541

3.7. Hipertensão Mascarada Não Controlada e Hipertensão do Avental Branco Não Controlada 542

3.8. Recomendações para Diagnóstico e Seguimento 542

3.9. Pressão Aórtica Central 542

3.10. Genética e Hipertensão Arterial 542

4. Avaliação Clínica e Complementar 548

4.1. História Clínica 548

4.2. Avaliação Clínica 548

**4.2.1. Anamnese** 548

4.3. Exame Físico 548

**4.3.1. Investigação Laboratorial Básica, Avaliação de Lesões Clínicas e subclínicas em Órgãos-Alvo** 548

5. Estratificação de Risco Cardiovascular 552

5.1. Introdução 552

5.2. Estratificação de Risco Adicional (Condições Associadas) 552

**5.2.1. Lesões em Órgãos-Alvo** 553

**5.2.2. Presença de Doença Cardiovascular e Renal** 553

5.3. Avaliação do Risco Cardiovascular Global 553

5.4. Desafios na Avaliação do Risco Cardiovascular na Hipertensão

Arterial 553

6. Decisão e Metas Terapêuticas 556

6.1. Introdução 556

6.2. O Hipertenso de Risco Baixo ou Moderado 556

6.3. O Hipertenso de Alto Risco 556

6.4. O Hipertenso com Doença Coronária 556

6.5. O Hipertenso com História de Acidente Vascular Encefálico 556

6.6. O Hipertenso com Insuficiência Cardíaca 557

6.7. Hipertenso com Doença Renal Crônica (DRC) 557

6.8. O Hipertenso Diabético 557

6.9. O Hipertenso Idoso 557

7. Equipe Multiprofissional 559

7.1. A Importância da Abordagem Multiprofissional no Controle da Hipertensão 559

7.2. Formação e Atuação da Equipe 559

**7.2.1. Profissional Médico – Ações Específicas** 559

**7.2.2. Profissional Enfermeiro – Ações Específicas** 559

**7.2.2.1. Ações Específicas da Enfermagem na Atenção Primária** 560

**7.2.3. Profissional Nutricionista – Ações Específicas** 560

**7.2.3.1. Consulta do Nutricionista** 560

**7.2.3.2. Ações Coletivas do Nutricionista** 560

**7.2.4. Profissional de Educação Física – Ações Específicas** 560

**7.2.4.1. Ações Coletivas dos Profissionais de Educação Física e Fisioterapia** 561

7.3. Ações da Equipe Multiprofissional 561

8. Tratamento não Medicamentoso 562

8.1. Introdução 562

8.2. Tabagismo 562

8.3. Padrão Alimentar 562

8.4. Sódio da Dieta 563

8.5. Potássio 563

8.6. Laticínios 563

8.7. Chocolate e Produtos com Cacau 563

8.8. Café e Produtos com Cafeína 563

8.9. Vitamina D 564

8.10. Suplementos e Substitutos 564

8.11. Perda de Peso 564

8.12. Consumo de Bebidas Alcoólicas 564

8.13. Atividade Física e Exercício Físico 564

8.14. Respiração Lenta 565

8.15. Controle de Estresse 565

8.16. Espiritualidade e Religiosidade 565

9. Tratamento Medicamentoso 568

9.1. Objetivos do Tratamento 568

9.2. Princípios Gerais do Tratamento Medicamentoso 568

9.3. Esquemas Terapêuticos 568

**9.3.1. Monoterapia** 568

**9.3.2. Combinação de Medicamentos** 569

9.4. Características Gerais das Diferentes Classes de medicamentos Anti-Hipertensivos 569

**9.4.1. Diuréticos (DIU)** 569

**9.4.1.1. Efeitos Adversos dos Diuréticos** 569

**9.4.2. Bloqueadores dos Canais de Cálcio (BCC)** 569

**9.4.2.1. Efeitos Adversos dos Bloqueadores de Canal de Cálcio** 570

**9.4.3. Inibidores da Enzima Conversora da Angiotensina (IECA)** 570

**9.4.3.1. Efeitos Adversos dos Inibidores da Enzima Conversora da Angiotensina** 570

**9.4.4. Bloqueadores dos Receptores AT1 da Angiotensina II (BRA)** 570

**9.4.4.1. Efeitos Adversos dos Bloqueadores dos Receptores AT1 da Angiotensina II** 570

**9.4.5. Betabloqueadores (BB)** 570

**9.4.5.1. Efeitos Adversos dos Betabloqueadores** 571

**9.4.6. Simpatolíticos de Ação Central** 571

**9.4.6.1. Efeitos Adversos dos Simpatolíticos de Ação Central** 571

**9.4.7. Alfabloqueadores** 571

**9.4.7.1. Efeitos Adversos dos Alfabloqueadores** 572

**9.4.8. Vasodilatadores Diretos** 572

**9.4.8.1. Efeitos Adversos dos Vasodilatadores Diretores** 572

**9.4.9. Inibidores Diretos da Renina** 572

**9.4.9.1. Efeitos Adversos dos Inibidores Diretos da Renina** 572

9.5. Associações de Fármacos Anti-hipertensivos 572

10. Hipertensão e Condições Clínicas Associadas 578

10.1. Diabetes Melito (DM) 578

**10.1.1. Objetivos do Tratamento** 578

10.2. Síndrome Metabólica (SM) 578

10.3. Doença Arterial Coronária (DAC) 578

10.4. Hipertensão na Doença Renal Crônica (DRC) 579

**10.4.1. Paciente em Tratamento Conservador – Metas e Tratamento** 579

**10.4.2. Pacientes em Terapia Renal Substitutiva (TRS): Metas e Tratamento** 579

10.5. Insuficiência cardíaca (IC) 579

10.6. Acidente Vascular Encefálico Hemorrágico (AVEH) e Acidente Vascular Encefálico Isquêmico (AVEI) 580

**10.6.1. Acidente Vascular Encefálico Hemorrágico** 580

**10.6.2. Acidente Vascular Encefálico Isquêmico** 580

11. Hipertensão Arterial na Gestação 581

11.1. Epidemiologia 581

11.2. Classificação da Hipertensão Arterial na Gestação 581

11.3. Conceito e Critérios Diagnósticos 581

11.4. Predição e Prevenção de Pré-eclâmpsia 582

11.5. Tratamento não Medicamentoso 582

11.6. Conduta Expectante 582

11.7. Tratamento Medicamentoso 583

11.8 Risco Cardiovascular Futuro 583

12. Hipertensão Arterial na Criança e no Adolescente 586

12.1. Contexto Epidemiológico e Importância da Hipertensão em

Pediatria 586

12.2. Definição e Etiologia 586

12.3. Diagnóstico 586

**12.3.1. Métodos de Medida da PA** 586

12.4. Anamnese 587

12.5. Exame Físico 587

12.6. Exames Complementares 587

12.7. Monitorização Ambulatorial de Pressão Arterial (MAPA) 587

12.8. Aspectos terapêuticos 587

12.9. Terapêutica não Farmacológica 587

12.10. Terapêutica Farmacológica 588

12.11. Seguimento de Crianças e Adolescentes com HA 588

12.12. Crise Hipertensiva 588

13. Crise Hipertensiva 596

13.1. Definição 596

13.2. Classificação 596

13.3. Principais Aspectos Epidemiológicos, Fisiopatogênicos e

Prognósticos 596

**13.3.1. Epidemiologia** 596

**13.3.2. Fisiopatogenia** 596

**13.3.3. Prognóstico** 596

13.4. Investigação Clinicolaboratorial Complementar 597

13.5. Tratamento Geral da Crise Hipertensiva 597

13.6. Emergências Hipertensivas em Situações Especiais 597

**13.6.1. Encefalopatia Hipertensiva** 597

13.7. Acidente Vascular Encefálico (AVE) 597

**13.7.1. Acidente Vascular Encefálico Isquêmico (AVEI)** 597

**13.7.2. Acidente Vascular Encefálico Hemorrágico (AVEH)** 598

**13.7.3. Síndromes Coronarianas Agudas** 598

**13.7.4. Edema Agudo de Pulmão (EAP)** 598

**13.7.4.1. Dissecção Aguda de Aorta** 598

**13.7.5. Pré-eclâmpsia/Eclâmpsia** 598

**13.7.6. EH pelo Uso de Substâncias Ilícitas** 598

**13.7.7. Hipertensão Acelerada/Maligna** 599

**13.7.8. Hipertensão com Múltiplos Danos aos Órgãos-alvo** 599

14. Hipertensão Arterial no Idoso 602

14.1. Introdução 602

14.2. Mecanismos Fisiopatológicos 602

14.3. Diagnóstico e Decisão Terapêutica 603

14.4. Tratamento 603

**14.4.1.Tratamento Não Medicamentoso** 603

**14.4.2. Tratamento Farmacológico** 604

14.5. Situações Especiais 604

**14.5.1.** ***Status*** **Funcional e Fragilidade: Avaliação e Implicações** 604

**14.5.2. Declínio Cognitivo e Demência** 604

**14.5.3. Polifarmácia e Adesão** 605

**14.5.4. Desintensificação e Desprescrição** 605

**14.5.5. Hipotensão Ortostática e Pós-prandial** 605

15. Hipertensão Arterial Secundária 607

15.1. Introdução 607

15.2. Causas Não Endócrinas 607

**15.2.1. Doença Renal Crônica (DRC)** 607

**15.2.2. Hipertensão Renovascular (HARV)** 607

15.3. Displasia Fibromuscular 608

**15.3.1. Coarctação da Aorta** 608

**15.3.2. Apneia Obstrutiva do Sono (AOS)** 608

**15.3.2.1. Conceito e Epidemiologia** 608

**15.3.2.2. Apresentação Clínica e Triagem da AOS no Contexto da HA** 609

**15.3.2.3. Impacto do Tratamento da AOS sobre a PA** 609

**15.3.2.4. Tratamento Anti-hipertensivo em Pacientes Hipertensos com AOS** 609

15.4. Causas Endócrinas 609

**15.4.1. Hiperaldosteronismo Primário (HP)** 609

**15.4.2. Feocromocitoma** 610

**15.4.3. Hipotireoidismo** 610

**15.4.4. Hipertireoidismo** 610

**15.4.5. Hiperparatireoidismo Primário** 610

**15.4.6. Síndrome de Cushing** 611

**15.4.7. Obesidade** 611

**15.4.8. Acromegalia** 611

15.5. Causas Medicamentosas, Hormônios e Substâncias Exógenas 611

16. Hipertensão Arterial Resistente e Refratária 619

16.1. Definição e Classificação 619

16.2. Epidemiologia da Hipertensão Arterial Resistente 619

16.3. Fisiopatologia 619

16.4. Investigação Diagnóstica 619

16.5. Tratamento 620

**16.5.1. Tratamento Não Farmacológico** 620

**16.5.2. Tratamento Farmacológico** 620

**16.5.3. Novos Tratamentos** 620

17. Adesão ao Tratamento Anti-Hipertensivo 624

17.1. Introdução 624

17.2. Conceito de Adesão 624

17.3. Métodos de Avaliação da Adesão ao Tratamento 624

17.4. Fatores que Inteferem na Adesão ao Tratamento 624

17.5. Estratégias para promover a adesão ao tratamento

anti-hipertensivo 625

17.6. Conclusão 625

18. Perspectivas 627

18.1. Introdução 627

18.2. Definição, Epidemiologia e Prevenção Primária 627

18.3. Pressão Arterial e Dano Vascular 628

18.4. Biomarcadores Cardíacos 628

18.5. Diagnóstico e Classificação 628

18.6. Avaliação Complementar e Estratificação do Risco

Cardiovascular 629

18.7. Metas e Tratamento 629

Referências 630

## 1. Definição, Epidemiologia e Prevenção Primária

## 1.1 Definição de Hipertensão Arterial

**Figura 1.1:**
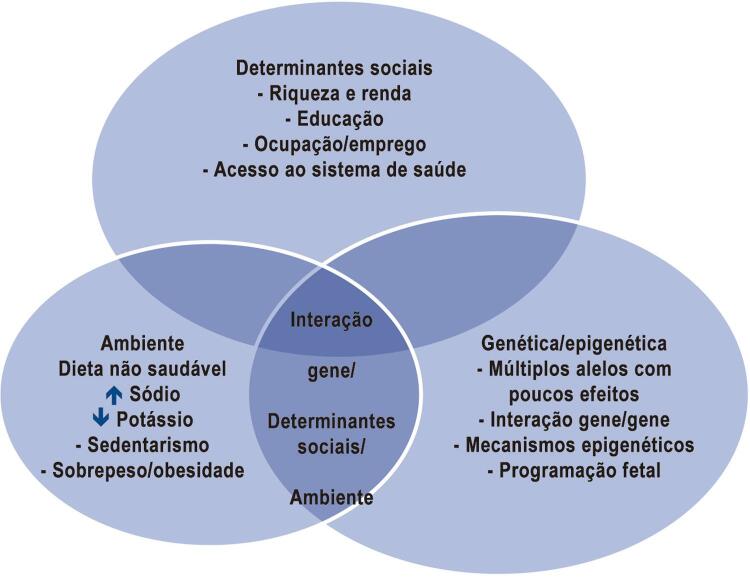
– Carey et al. 2008. ^6^

A hipertensão arterial (HA) é uma doença crônica não transmissível (DCNT) definida por níveis pressóricos, em que os benefícios do tratamento (não medicamentoso e/ou medicamentoso) superam os riscos. Trata-se de uma condição multifatorial, que depende de fatores genéticos/epigenéticos, ambientais e sociais ( Figura 1.1 ), caracterizada por elevação persistente da pressão arterial (PA), ou seja, PA sistólica (PAS) maior ou igual a 140 mmHg e/ou PA diastólica (PAD) maior ou igual a 90 mmHg, medida com a técnica correta, em pelo menos duas ocasiões diferentes, na ausência de medicação anti-hipertensiva. É aconselhável, quando possível, a validação de tais medidas por meio de avaliação da PA fora do consultório por meio da Monitorização Ambulatorial da Pressão Arterial (MAPA), da Monitorização Residencial da Pressão Arterial (MRPA) ou da Automedida da Pressão Arterial (AMPA) (ver Capítulo 3).

Determinantes genéticos/epigenéticos, ambientais e sociais interagem para elevar a PA em hipertensos e na população em geral. ↑aumentado; ↓diminuído. Fonte: Carey et al. 2008. ^6^

## 1.2. Impacto da Hipertensão Arterial nas Doenças Cardiovasculares

Por se tratar de condição frequentemente assintomática, a HA costuma evoluir com alterações estruturais e/ou funcionais em órgãos-alvo, como coração, cérebro, rins e vasos. Ela é o principal fator de risco modificável com associação independente, linear e contínua para doenças cardiovasculares (DCV), doença renal crônica (DRC) e morte prematura. Associa-se a fatores de risco metabólicos para as doenças dos sistemas cardiocirculatório e renal, como dislipidemia, obesidade abdominal, intolerância à glicose, e diabetes melito (DM). ^1^ ^-^ ^6^

Além disso, apresenta impacto significativo nos custos médicos e socioeconômicos, decorrentes das complicações nos órgãos-alvo, fatais e não fatais, como: coração: doença arterial coronária (DAC), insuficiência cardíaca (IC), fibrilação atrial (FA) e morte súbita; cérebro: acidente vascular encefálico (AVE) isquêmico (AVEI) ou hemorrágico (AVEH), demência; rins: DRC que pode evoluir para necessidade de terapia dialítica; e sistema arterial: doença arterial obstrutiva periférica (DAOP). ^3^ ^-^ ^6^

## 1.3. Fatores de Risco para Hipertensão Arterial

### 1.3.1. Genética

Os fatores genéticos podem influenciar os níveis de PA entre 30-50%. ^7^ No entanto, devido à ampla diversidade de genes, às variantes genéticas estudadas até o momento e à miscigenação em nosso país, não foram identificados dados uniformes com relação a tal fator. Mais detalhes sobre o componente genético da HA podem ser encontrados no Capítulo 3.

### 1.3.2. Idade

Com o envelhecimento, a PAS torna-se um problema mais significante, resultante do enrijecimento progressivo e da perda de complacência das grandes artérias. Em torno de 65% dos indivíduos acima dos 60 anos apresentam HA, e deve-se considerar a transição epidemiológica que o Brazil vem sofrendo, com um número ainda maior de idosos (≥ 60 anos) nas próximas décadas, o que acarretará um incremento substancial da prevalência de HA e de suas complicações. ^7^ ^,^ ^8^

### 1.3.3. Sexo

Em faixas etárias mais jovens, a PA é mais elevada entre homens, mas a elevação pressórica por década se apresenta maior nas mulheres. Assim, na sexta década de vida, a PA entre as mulheres costuma ser mais elevada e a prevalência de HA, maior. Em ambos os sexos, a frequência de HA aumenta com a idade, alcançando 61,5% e 68,0% na faixa etária de 65 anos ou mais, em homens e mulheres, respectivamente. ^7^

### 1.3.4. Etnia

A etnia é um fator de risco importante para a HA, mas condições socioeconômicas e de hábitos de vida parecem ser fatores mais relevantes para as diferenças na prevalência da HA do que a implicação étnica propriamente dita. ^7^ ^,^ ^8^ Dados do Vigitel 2018 mostraram que, em nosso país, não houve uma diferença significativa entre negros e brancos no que diz respeito à prevalência de HA (24,9% *versus* 24,2%). ^9^

### 1.3.5. Sobrepeso/Obesidade

Parece haver uma relação direta, contínua e quase linear entre o excesso de peso (sobrepeso/obesidade) e os níveis de PA. ^3^ ^-^ ^6^ Apesar de décadas de evidências inequívocas de que a circunferência de cintura (CC) fornece informações independentes e aditivas ao índice de massa corpórea (IMC) para predizer morbidade e risco de morte, tal medida não é rotineiramente realizada na prática clínica. Recomenda-se que os profissionais de saúde sejam treinados para realizar adequadamente essa simples medida e considerá-la como um importante “sinal vital” na prática clínica. ^3^ ^-^ ^6^

### 1.3.6. Ingestão de Sódio e Potássio

A ingestão elevada de sódio tem-se mostrado um fator de risco para a elevação da PA, e consequentemente, da maior prevalência de HA. A literatura científica mostra que a ingestão de sódio está associada a DCV e AVE, quando a ingestão média é superior a 2 g de sódio, o equivalente a 5 g de sal de cozinha. ^10^ Estudos de medida de excreção de sódio mostraram que naqueles com ingestão elevada de sódio, a PAS foi 4,5 mmHg a 6,0 mmHg maior e a PAD 2,3 mmHg a 2,5 mmHg em comparação com os que ingeriam as quantidades recomendadas de sódio. ^11^

Cabe destacar, ainda, que o consumo excessivo de sódio é um dos principais fatores de risco modificáveis para a prevenção e o controle da HA e das DCV e que, em 2013, US$ 102 milhões dos gastos do SUS com hospitalizações foram atribuíveis ao consumo excessivo de sódio. ^12^

De maneira inversa, o aumento na ingestão de potássio reduz os níveis pressóricos. Deve ser salientado que o efeito da suplementação de potássio parece ser maior naqueles com ingestão elevada de sódio e entre os indivíduos da raça negra. A ingestão média de sal no Brazil é de 9,3 g/dia (9,63 g/dia para homens e 9,08 g/dia para mulheres), enquanto a de potássio é de 2,7 g/dia para homens e 2,1 g/dia para mulheres. ^12^ ^,^ ^13^

### 1.3.7. Sedentarismo

Há uma associação direta entre sedentarismo, elevação da PA e da HA. ^3^ ^-^ ^6^ Chama a atenção que, em 2018, globalmente, a falta de atividade física (menos de 150 minutos de atividade física por semana ou 75 minutos de atividade vigorosa por semana) era de 27,5%, com maior prevalência entre as mulheres (31,7%) do que nos homens (23,4%). ^14^

No Brazil, o inquérito telefônico Vigitel de 2019 identificou que 44,8% dos adultos não alcançaram um nível suficiente de prática de atividade física, sendo esse percentual maior entre mulheres (52,2%) do que entre homens (36,1%). ^9^

### 1.3.8. Álcool

O impacto da ingestão de álcool foi avaliado em diversos estudos epidemiológicos. Há maior prevalência de HA ou elevação dos níveis pressóricos naqueles que ingeriam seis ou mais doses ao dia, o equivalente a 30 g de álcool/dia = 1 garrafa de cerveja (5% de álcool, 600 mL); = 2 taças de vinho (12% de álcool, 250 mL); = 1 dose (42% de álcool, 60 mL) de destilados (uísque, vodca, aguardente). Esse limite deve ser reduzido à metade para homens de baixo peso e mulheres. ^15^ ^,^ ^16^

### 1.3.9. Fatores Socioeconômicos

Entre os fatores socioeconômicos, podemos destacar menor escolaridade e condições de habitação inadequadas, além da baixa renda familiar, como fatores de risco significativo para HA. ^17^ ^,^ ^18^

### 1.3.10. Outros Fatores de Risco Relacionados com a Elevação da PA

Além dos fatores clássicos mencionados, é importante destacar que algumas medicações, muitas vezes adquiridas sem prescrição médica, e drogas ilícitas têm potencial de promover elevação da PA ou dificultar seu controle. Esse tópico será abordado em mais detalhes no Capítulo 15. Entre eles, estão: os inibidores da monoaminaoxidase e os simpatomiméticos, como descongestionantes nasais (fenilefrina), antidepressivos tricíclicos (imipramina e outros), hormônios tireoidianos, contraceptivos orais, anti-inflamatórios não esteroides, carbexonolona e liquorice, glicocorticoides, ciclosporina, eritropoietina, drogas ilícitas (cocaína, *cannabis sativa* , anfetamina e 3,4-metilenodioximetanfetamina (MDMA). ^5^ ^,^ ^19^

### 1.3.11. Apneia Obstrutiva do Sono (AOS)

Há clara evidência que sustenta a relação entre a AOS e a HA e o aumento do risco para HA resistente (ver também no Capítulo 15). Os graus leve, moderado e grave da AOS mantém uma relação dose-resposta com a HA. Existe uma associação mais forte de caucasianos e pacientes do sexo masculino à AOS. ^3^ ^-^ ^6^ ^,^ ^20^

### 1.3.12. Dados Epidemiológicos Globais

**Figura 1.2:**
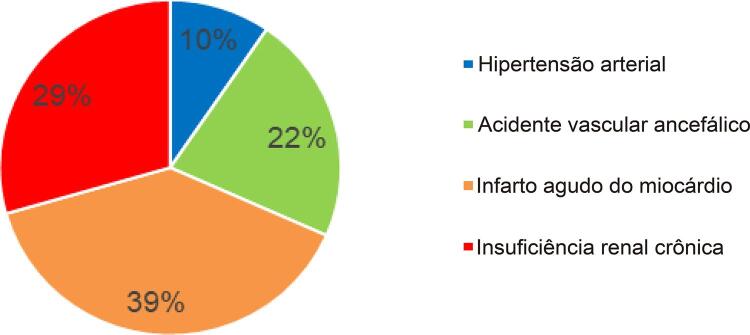
– Percentual de óbitos por hipertensão arterial, infarto agudo do miocárdio, acidente vascular encefálico e insuficiência renal crônica (Brazil, 2000).

As DCV são a principal causa de morte, hospitalizações e atendimentos ambulatoriais em todo o mundo, inclusive em países em desenvolvimento como o Brazil. ^21^ Em 2017, dados completos e revisados do Datasus mostraram a ocorrência de 1.312.663 óbitos no total, com um percentual de 27,3% para as DCV. ^22^ A HA estava associada em 45% destas mortes cardíacas: DAC e IC e de 51,0% das mortes por doença cerebrovascular (DCbV) e um percentual muito pequeno de mortes diretamente relacionadas com a HA (13,0%). Vale ressaltar que a HA mata mais por suas lesões nos órgãos-alvo. ^23^ ( Figura 1.2 ).

No ano de 2017, dados da Carga Global das Doenças (GBD) indicaram que as DCV foram responsáveis por 28,8 % do total de mortes entre as doenças crônicas não transmissíveis (DCNT). O estudo GBD revelou que, em 2017, ocorreram quase 18 milhões de mortes por causas CV (31,8% do total de mortes), representando 20,6% do total de anos de vida perdidos (YLL) e 14,7% do total de DALYs (anos de vida ajustados para a incapacidade ou, em outras palavras, anos perdidos de vida saudável). ^18^ ^,^ ^21^

Ainda, segundo o GBD, observou-se que a elevação da PAS foi o principal fator de risco, responsável por 10,4 milhões de mortes e 218 milhões de DALYs. ^21^ Foi também responsável por cerca de 40,0% das mortes em portadores de DM, 14,0% da mortalidade materno-fetal na gravidez e 14,7% do total de DALYs para a DRC. ^24^ ^-^ ^26^

Globalmente, em 2010, a prevalência de HA (≥140/90 mmHg e/ou em uso de medicação anti-hipertensiva) foi de 31,0%, sendo maior entre homens (31,9%) do que entre as mulheres (30,1%). ^17^ ^,^ ^18^

Um estudo de tendência mundial da PA entre 1975-2015, que avaliou 19,1 milhões de adultos, mostrou que em 2015 o número estimado de adultos com HA era de 1,13 bilhões, sendo 597 milhões de homens e 529 milhões de mulheres, indicando um aumento de 90% no número de pessoas com HA, principalmente nos países de baixa e média rendas. ^17^ ^,^ ^18^ O estudo mostrou que a prevalência de HA diminuiu nos países de alta renda e em alguns de média, enquanto nos países de baixa renda aumentou ou se manteve constante. Os fatores implicados nesse incremento seriam o envelhecimento da população e a maior exposição aos outros fatores de risco, como ingestão elevada de sódio e baixa de potássio, além do sedentarismo. ^17^ ^,^ ^18^

## 1.4. Prevalência de Hipertensão Arterial no Brazil

**Figura 1.3:**
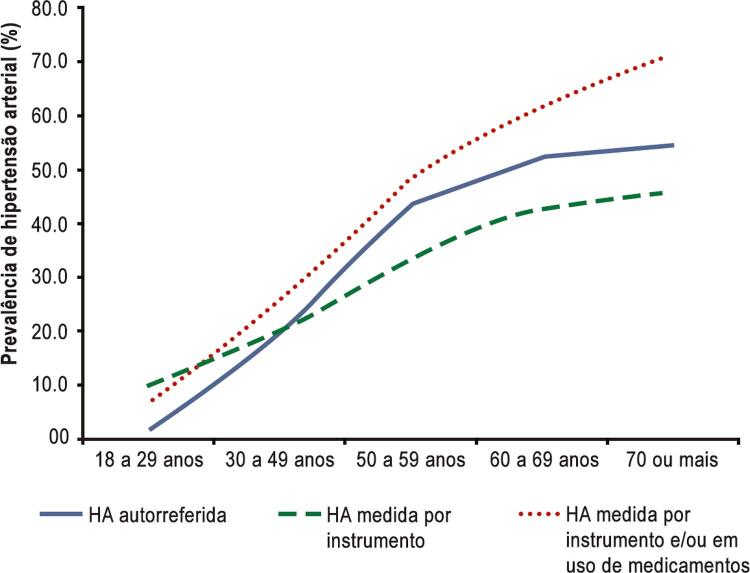
– Prevalência populacional de hipertensão arterial segundo diferentes critérios diagnósticos, em adultos com 18 anos de idade ou superior, ambos os sexos, por faixa etária (Brazil, 2013).

Os dados de prevalência no país tendem a variar de acordo com a metodologia e a casuística utilizadas. Segundo a Pesquisa Nacional de Saúde de 2013, 21,4% (IC 95% 20,8-22,0) dos adultos Brazileiros autorrelataram HA, enquanto, considerando as medidas de PA aferidas e uso de medicação anti-hipertensiva, o percentual de adultos com PA maior ou igual que 140 por 90 mmHg chegou a 32,3% (IC 95% 31,7-33,0). Detectou-se que a prevalência de HA foi maior entre homens, além de, como esperado, aumentar com a idade por todos os critérios, chegando a 71,7% para os indivíduos acima de 70 anos ( Tabela 1.1 e Figura 1.3 ). ^27^

**Tabela 1.1:** – Prevalência de hipertensão arterial e intervalo de confiança 95% de acordo com três critérios utilizados

	HA autorreferida (Vigitel)	PA medida ≥ 140/90 mmHg (PNS, 2013)	PA medida ≥ 140/90 mmHg e/ou uso de medicação anti-hipertensiva (PNS, 2013)
**Total**	21,4% (20,8-22,0)	22,8% (22,1-23,4)	32,3% (31,7-33,0)
**Sexo masculino**	18,3 (17,5-19,1)	25,8(24,8-26,7)	33,0 (32,1-34,0)
**Sexo feminino**	24,2 (23,4-24,9)	20,0 (19,3-20,8)	31,7 (30,9-32,5)

HA: hipertensão arterial; PA: pressão arterial. Fonte: Nilson et al. 2020. ^29^

Em 2017, ocorreu um total de 1.312.663 óbitos, com um percentual de 27,3% para as DCV. Essas doenças representaram 22,6% das mortes prematuras no Brazil (entre 30 e 69 anos de idade). No período de uma década (2008 a 2017), foram estimadas 667.184 mortes atribuíveis à HA no Brazil. ^21^ ^-^ ^23^

Com relação à tendência do coeficiente de mortalidade por 100.000 habitantes de 2000 a 2018, podemos observar um leve aumento no IAM e um importante aumento da HA relatada de forma direta, com incrementos, respectivamente, de 25% e 128%. ^23^

Quanto à morbidade, podemos observar no período dos últimos dez anos uma tendência de estabilidade das internações (Sistema de Internações Hospitalares do Datasus) por todas as causas e pelas DCV corrigido por habitantes ( Figura 1.3 ). ^5^ ^,^ ^23^ Em termos de custos ao SUS, a HA tem custos atribuíveis maiores do que os da obesidade e do DM. Em 2018, estimaram-se gastos de US$ 523,7 milhões no SUS, com hospitalizações, procedimentos ambulatoriais e medicamentos. ^28^

Ao longo da última década, 77% dos custos com hospitalizações no SUS com DAC são representados por DCV associadas à HA e aumentaram 32%, em reais, de 2010 a 2019, passando de R$ 1,6 bilhão para R$ 2,2 bilhões no período. ^28^ ^,^ ^29^

## 1.5. Prevenção Primária

### 1.5.1. Introdução

A HA tem alta prevalência e é um dos principais fatores de risco para as DCV e renais, apresentando determinantes genéticos, ambientais e sociais combinados. Mostra-se de fácil diagnóstico e seu tratamento é eficaz utilizando-se um arsenal terapêutico diversificado, bastante eficiente e com poucos efeitos adversos. Mesmo assim, seu controle em todo o mundo é pífio, porque se trata de doença frequentemente assintomática, o que dificulta a adesão aos cuidados.

A equação final torna o desafio do tratamento muito elevado, e a prevenção continua a ser a melhor opção em termos de custo-benefício. A abordagem adequada dos fatores de risco para o desenvolvimento da HA deve ser o grande foco do SUS. Nesse quesito, há vários pontos que merecem destaque. Muitos se confundem ou se somam ao tratamento não medicamentoso ( Quadro 1.1 ), descrito detalhadamente no Capítulo 8. ^3^ ^,^ ^5^ ^,^ ^6^ ^,^ ^30^ ^,^ ^31^

**Quadro 1.1:** – Principais intervenções que previnem hipertensão arterial

Modalidade	Intervenção NF	Dose	Diferença de PAS obtida
Controle do Peso	Peso/gordura corpórea	Alcançar peso ideal. Esperada diminuição de 1mmHg por cada quilo de peso perdido	- 2/3 mmHg
Dieta saudável	Dieta tipo DASH	Dieta rica em frutas, vegetais, grãos e baixo teor de gordura. Redução de gordura saturada e trans	- 3 mmHg
Redução da ingestão de sódio	Sódio na dieta	Ideal < 2 g ou pelo menos redução de 1,0 g/dia	- 2/3 mmHg
Aumento da ingestão de potássio	Potássio na dieta	3,5 a 5,0 g/dia em dieta rica em potássio	- 2 mmHg
Atividade física	Aeróbia	150 min/semana	-5/7 mmHg
	De resistência dinâmica	8 a 10 exercícios para os principais grupos musculares, 1 a 3 séries, 50 a 80% de 1 RM	
	De resistência isométrica	Exercício de handgrip (preensão de mão) unilateral ou 1 perna, 4 séries, 2 min de contração isométrica, 30% da contração voluntária máximo (CVM), 2-3 min de pausa entre as séries	-4/5 mmHg
Ingestão de álcool	Consumo de álcool	Para quem usa álcool Homens ≤ 2 drinques Mulheres ≤ 1 drinque	-4/5 mmHg

NF: não farmacológica; PAS: pressão arterial sistólica; RM: repetição máxima; mmHg: milímetros de mercúrio. Fonte: Adaptado de Carey et al., 2018. ^6^

### 1.5.2. Controle do Peso (GR: I; NE: A)

A obesidade geral e a obesidade abdominal foram associadas ao aumento do risco de HA. Por outro lado, a diminuição do peso promove a diminuição da PA tanto em indivíduos normotensos quanto em hipertensos. ^3^ ^,^ ^5^ ^,^ ^6^ Ser “o mais magro possível” dentro da faixa da normalidade do IMC pode ser a melhor sugestão com relação à prevenção primária da HA. ^3^ ^,^ ^5^ ^,^ ^6^ ^,^ ^32^ ^-^ ^36^

### 1.5.3. Dieta Saudável (GR: I; NE: A)

Há várias propostas de dietas para a prevenção da HA, que também favorecem o controle dos hipertensos e contribuem para a saúde como um todo. ^5^ ^,^ ^37^ Tem destaque, nesse sentido, a dieta DASH e suas variantes (baixa quantidade de gordura, mediterrânea, vegetariana/vegana, nórdica, baixo teor de carboidratos etc.). Os benefícios são ainda maiores quando ocorre em conjunto a redução de ingestão de sódio. ^5^ ^,^ ^37^ ^-^ ^40^

Todos os documentos sobre o assunto indicam a alimentação com consumo parcimonioso de frutas, verduras, legumes, cereais, leite e derivados, além de indicarem menor quantidade de gordura e sal. ^37^ ^-^ ^41^ Uma metanálise que comparou algumas variedades dessas dietas com a dieta padrão mostrou maior redução da PAS (-9,73 a -2,32 mmHg) e PAD (-4,85 a -1,27 mmHg) no grupo com dietas adequadas. ^39^ Devem ser levados em conta os aspectos socioeconômicos e culturais para que ocorra adesão a determinado tipo de recomendação alimentar. ^3^ ^,^ ^5^ ^,^ ^6^ ^,^ ^37^

### 1.5.4. Sódio (GR: I; NE: A)

O consumo excessivo de sódio é um dos principais fatores de risco modificáveis para a prevenção e o controle da HA e das DCV. ^29^ A restrição de sódio mostrou ter um efeito redutor da PA em muitos estudos. Uma metanálise apontou que uma redução de 1,75 g de sódio por dia (4,4 g de sal/dia) está associada a uma redução média de 4,2 e 2,1 mmHg na PAS e na PAD, respectivamente. O efeito redutor na PA com a restrição de sódio é maior em negros, idosos, diabéticos, naqueles que apresentam síndrome metabólica (SM) e na DRC. ^37^

Recomenda-se que a ingestão de sódio seja limitada a aproximadamente 2 g/dia (equivalente a cerca de 5 g de sal por dia) na população em geral. ^3^ ^-^ ^6^ A redução eficaz do sal não é fácil e, muitas vezes, há pouca valorização de quais alimentos contêm altos níveis de sal. Convém recomendações para se ter muito cuidado com a quantidade de sal adicionado e com os alimentos com alto teor de sal (produtos industrializados e processados). ^3^ ^-^ ^6^

A redução no consumo de sal na população Brazileira continua sendo prioridade de saúde pública, mas requer um esforço combinado entre indústria de alimentos, governos nas diferentes esferas e público em geral, já que 80% do consumo de sal envolve aquele contido nos alimentos processados. ^3^ ^-^ ^6^ ^,^ ^10^ ^,^ ^12^ ^,^ ^40^ O consumo adequado de frutas e vegetais potencializa o efeito benéfico da dieta com baixo teor de sódio sobre a PA. Os substitutos do sal contendo cloreto de potássio e menos cloreto de sódio (30% a 50%) são úteis para reduzir a ingestão de sódio e aumentar a de potássio, apesar de limitações em seu uso. ^42^

### 1.5.5. Potássio (GR: I; NE: A)

A relação entre o aumento da suplementação de potássio e a diminuição da HA está relativamente bem compreendida. ^43^ A suplementação de potássio constitui-se em uma alternativa segura, sem importantes efeitos adversos, com impacto modesto, mas significativo, na PA e pode ser recomendada para a prevenção do aparecimento da HA. ^43^ ^-^ ^47^ Uma ingestão adequada de potássio, na ordem de 90 a 120 mEq/dia, pode acarretar uma diminuição de 5,3 mmHg na PAS e 3,1 mmHg na PAD. ^45^ Sua ingestão pode ser aumentada pela escolha de alimentos pobres em sódio e ricos em potássio, como feijões, ervilha, vegetais de cor verde-escura, banana, melão, cenoura, beterraba, frutas secas, tomate, batata-inglesa e laranja. ^3^

### 1.5.6. Atividade Física (GR: I; NE: A)

O sedentarismo é um dos dez principais fatores de risco para a mortalidade global, causando cerca de 3,2 milhões de mortes a cada ano. ^48^ ^,^ ^49^ Uma metanálise com 93 artigos e 5.223 indivíduos indicou que o treinamento aeróbico, resistido dinâmico e resistido isométrico reduz a PAS/PAD em repouso em 3,5/2,5, 1,8/3,2 e 10,9/6,2 mmHg, respectivamente, em populações gerais. ^50^ ^-^ ^52^

Todos os adultos devem ser aconselhados a praticar pelo menos 150 min/semana de atividades físicas moderadas ou 75 min/semana de vigorosas. Os exercícios aeróbicos (caminhada, corrida, ciclismo ou natação) podem ser praticados por 30 minutos em 5 a 7 dias por semana. A realização de exercícios resistidos em 2 a 3 dias por semana também pode ser recomendada. ^50^ ^,^ ^52^ Para um benefício adicional, em adultos saudáveis, recomenda-se um aumento gradual da atividade física para 300 minutos por semana de intensidade moderada ou 150 minutos por semana de atividade física vigorosa, ou uma combinação equivalente de ambos, idealmente com exercício diário supervisionado. ^55^

### 1.5.7. Álcool (GR: IIA; NE: B)

Estima-se que o consumo excessivo de álcool seja responsável por cerca de 10-30% dos casos de HA e por aproximadamente 6% da mortalidade de todas as causas no mundo. ^3^ ^-^ ^6^ ^,^ ^15^ ^,^ ^56^ ^-^ ^59^ Para os consumidores de álcool, a ingestão de bebida alcoólica deve ser limitada a 30 g de álcool/dia = 1 garrafa de cerveja (5% de álcool, 600 mL); = 2 taças de vinho (12% de álcool, 250 mL); = 1 dose (42% de álcool, 60 mL) de destilados (uísque, vodca, aguardente). Esse limite deve ser reduzido à metade para homens de baixo peso, mulheres e indivíduos com sobrepeso e/ou triglicerídeos elevados. Indivíduos abstêmios não devem ser induzidos a beber. ^3^ ^-^ ^6^ ^,^ ^15^

### 1.5.8. Fatores Psicossociais (GR: IIb; NE: B)

O controle do estresse emocional, por diversas técnicas existentes, pode contribuir para a prevenção da HA. carecendo ainda de mais estudos robustos nesse sentido. ^3^ ^-^ ^6^ ^,^ ^60^ O treino desse controle resulta em: redução da reatividade CV e redução da PA e de sua variabilidade. ^61^ ^-^ ^63^

### 1.5.9. Suplementos Alimentares (GR: I a III; NE: A e B)

Os efeitos de redução da PA de suplementos alimentares são, em geral, discretos e heterogêneos. ^58^ ^-^ ^68^ As substâncias cuja suplementação tem alguma evidência de discreta redução da PA são: vitamina C, peptídeos bioativos derivados de alimentos, alho, fibras dietéticas, linhaça, chocolate amargo (cacau), soja, nitratos orgânicos e ômega 3. ^38^ ^,^ ^47^ ^,^ ^69^ As suplementações de magnésio, vitaminas combinadas, chá e coenzima Q10 não demonstraram redução significativa da PA. ^64^ ^,^ ^65^ ^,^ ^70^

### 1.5.10. Tabagismo (GR: I; NE: A)

Independentemente de seu efeito sobre a PA, abordar este tema mostra-se muito importante, porque o fumo é o único fator de risco totalmente evitável de doença e morte cardiovasculares, e seu enfrentamento precisa ser feito. ^3^ ^-^ ^6^ ^,^ ^71^ ^-^ ^75^ As estratégias recomendadas pela OMS para o controle do tabagismo seriam do ponto de vista de prevenção: evitar que o jovem experimente cigarro, pois, ao fazê-lo, terá probabilidade superior a 50% de tornar-se dependente, e aplicar a lei antifumo no país, particularmente a proibição de comercialização de produtos de tabaco para menores de idade, além de outras ações dirigidas a esse público. ^72^ O combate ao tabagismo é difícil, pelas dependências química e psíquica que causa, mas os benefícios da cessação na mortalidade CV já ocorrem a curto prazo. ^71^ ^,^ ^73^ ^-^ ^75^

O rigor no combate e no controle, a orientação contínua e o apoio psicoemocional incondicional ao tabagista, com a eventual prescrição de medicamentos, têm-se mostrado a abordagem mais eficaz. ^73^ É também importante a proteção contra a exposição ao fumo passivo que também implica maior risco. ^74^

### 1.5.11. Espiritualidade (GR: I; NE: B)

Dentro de um conceito de espiritualidade (E) que transcende religiosidade (R), mas que significa um conjunto de valores morais, emocionais, de comportamento e atitudes com relação ao mundo, temos evidências crescentes de seus benefícios em termos de risco CV, mortalidade e, particularmente, controle pressórico. ^76^

O estudo de coorte *Black Women’s Health Study* mostrou que as mulheres que lidavam com as situações de estresse ( *coping* ), usando a espiritualidade e a religiosidade, tinham um risco menor de desenvolver HA no seguimento de 10 anos (razão da taxa de incidência = 0,87; IC 95% 0,75-1,00), e essa associação era mais forte naquelas que relatavam maior nível de estresse. A pesquisa indicou que as situações de R/E contribuem com uma modulação mais suave de situações da vida cotidiana e trazem benefícios no controle da PA. ^77^

## 1.6. Estratégias para a Implementação de Medidas Preventivas

As mudanças no estilo de vida (MEV) são de difícil implementação, e a sociedade como um todo deve participar deste esforço. São importantes programas contínuos de educação em saúde dirigidos a alunos de escolas profissionalizantes; alunos de primeiro e segundo graus; equipes de instituições; empresas; e comunidade. As ações de conscientização são estratégias importantes, por meio de mídia; campanhas temáticas periódicas (Dias Municipal, Estadual e/ou Nacional de Prevenção e Combate à HA – lei federal 10.439 de 30/04/2002, Semana da HA, *May Measurement Month da International Society of Hypertension* etc.); e ações adicionais: incorporação das ações de prevenção, detecção e controle da HA nos programas de atenção primária à saúde, incluindo crianças e adolescentes e, particularmente, programas de saúde escolar; implementação de programas de assistência multiprofissional; fortalecimento de normas governamentais para reduzir o conteúdo de sódio e gorduras saturadas dos alimentos industrializados; aperfeiçoamento na rotulagem do conteúdo nutricional dos alimentos; e monitorização das ações de prevenção e controle da HA e seus resultados por meio de eficientes indicadores de saúde. ^3^ ^-^ ^6^

**Table:** 

Mensagens principais
Os números que definem a hipertensão arterial são arbitrários, mas se caracterizam como valores em que os benefícios do tratamento (não medicamentoso e/ou medicamentoso) superam os riscos.
A HA é uma condição multifatorial (genética, meio ambiente, hábitos de vida e fatores socioeconômicos).
A HA é um dos principais fatores de risco para doenças cardiovasculares e renais.
A HA tem alta prevalência, é de fácil diagnóstico e possui tratamento adequado, mas é de difícil controle pela baixa adesão.
A prevenção da HA é custo-efetiva e o melhor caminho para a diminuição da morbimortalidade cardiovascular.

## 2. Pressão Arterial e Dano Vascular

## 2.1. Introdução

Valores de pressão arterial (PA) elevados têm sido tradicionalmente associados ao risco para cardiopatia isquêmica, acidente vascular encefálico (AVE), doença renal crônica (DRC) e mortalidade precoce. Uma clássica metanálise de 61 estudos observacionais, com seguimento de 12,7 milhões de pessoas-ano e registro de 56.000 mortes por doença arterial coronária (DAC) ou AVE, produziu uma sólida evidência observacional. ^78^ Essa metanálise demonstrou que o risco inicia-se com valores de PA tão baixos quanto 115 mmHg de PA sistólica (PAS) ou 75 mmHg de PA diastólica (PAD), dobrando a cada 20 mmHg de elevação da PAS ou 10 mmHg de PAD. Apesar da consistência das evidências observacionais, não houve incorporação desses achados na definição do diagnóstico de hipertensão arterial (HA), que permanece há muitos anos em 140/90 mmHg.

Dessa forma, os pacientes continuam sendo classificados como hipertensos com níveis de PA acima de 140/90 mmHg, e indivíduos com PAS entre 120 a 139 mmHg e PAD entre 80 a 89 mmHg são classificados como portadores de PA normal ou pré-hipertensos, sendo que tais pessoas apresentam risco cardiovascular mais elevado em comparação com a PA ótima ou normal. O impacto da pré-hipertensão (sistólica entre 130-139 mmHg e diastólica entre 85-89 mmHg) sobre o risco vascular foi descrito em 2001 por Vasan et al., ^79^ que analisaram 6.859 participantes do estudo de Framingham. Neste estudo, os autores encontraram um aumento no risco absoluto para eventos cardiovasculares (CV). Vários outros estudos foram publicados a partir daí, analisando-se também pacientes considerados como níveis mais baixos (sistólica entre 120-139 e diastólica 80-89 mmHg), como no estudo de Hisayama, de Fukuhara et al., ^80^ que também encontraram um aumento no risco de doenças CV.

Diversos outros estudos foram publicados desde o estudo inicial de Vasan et al., o que levou a uma metanálise publicada em 2019 por Han et al., ^81^ analisando 47 estudos, em uma população de 491.666 indivíduos. Nessa metanálise, após o controle dos múltiplos fatores de risco CV, a pré-hipertensão aumentou em 40% o risco total de doenças, sendo que 12,09% das doenças CV; 13,26% das doenças coronarianas; 24,60% dos infartos do miocárdio (IAM); e 19,15% dos AVE poderiam ser prevenidos com seu controle efetivo.

Diante disso, os pré-hipertensos, mesmo ainda considerados como não hipertensos, deveriam ser melhor avaliados e estratificados. Alguns exames complementares não invasivos são capazes de avaliar o impacto da PA sobre o vaso, analisando o dano vascular precoce, não apenas em populações de hipertensos, mas também em pré-hipertensos, ^82^ como a dilatação mediada pelo fluxo (DMF), que verifica a função endotelial, e a velocidade de onda de pulso (VOP) e o índice tornozelo-braquial (ITB) que conferem a camada média arterial. Este capítulo tem por objetivo demonstrar o impacto do aumento da PA no risco CV, na disfunção endotelial (dano camada endotelial vascular) e na rigidez arterial (dano camada média vascular) antes mesmo do diagnóstico de HA.

## 2.2. Pressão Arterial, Desfechos Clínicos e Dano Cardiovascular

Na metanálise de Law et al., ^83^ a redução de PAS em 10 mmHg em ensaios clínicos randomizados promoveu a prevenção de IAM e AVE na proporção estimada por estudos observacionais para aquela elevação da PA. O mesmo ocorreu em uma metanálise mais recente. ^84^ Nesse estudo, a redução de risco relativo para eventos CV em ensaios em que os participantes foram tratados para meta de PAS entre 120 e 124 mmHg, em comparação com mais de 160 mmHg, foi de 64%, o que é próximo à redução de risco de 75% para uma redução estimada de 40 mmHg de PAS na metanálise realizada pela *Prospective Studies Collaboration.* ^78^ Outras metanálises têm convergido quanto a esses achados, com destaque para a maior delas, com mais de 600.000 participantes de ensaios clínicos analisados. ^85^ O ensaio clínico SPRINT acrescentou evidência maior aos estudos anteriormente comentados. ^86^ Pacientes randomizados para uma meta de PAS < 120 mm Hg (tratamento intensivo) tiveram uma redução de 25% na incidência de DCV, em comparação com queles randomizados para PA-alvo inferior a 140 mmHg. Houve redução de 43% na mortalidade por DCV e 27% na mortalidade por todas as causas. Demonstrou-se um benefício similar em um subgrupo de participantes com 75 anos ou mais na linha de base, incluindo aqueles com fragilidade ^87^ (GR: I NE: A).

Mais recentemente, foram publicados diversos estudos de coorte, muitos com grandes amostras. Eles demonstraram que a elevação da PA propicia riscos similares aos demonstrados para DAC e AVE para a incidência de outros desfechos CV. Entre eles, incluem-se insuficiência cardíaca (IC), com e sem fração de ejeção (FE) preservada, ^88^ fibrilação atrial, ^89^ cardiopatias valvares, ^90^ ^,^ ^91^ doença arterial periférica, ^92^ doença renal crônica (DRC), ^93^ ^,^ ^94^ demência ^95^ ^,^ ^96^ e doença de Alzheimer. ^97^ Provavelmente, diabetes melito, ^98^ disfunção erétil ^99^ e degeneração macular da senilidade ^100^ decorrem de elevações sustentadas da PA. Essas consequências exteriorizam-se, em geral, depois de muitos anos de exposição aos níveis pressóricos elevados, geralmente em valores que anteriormente não eram associados a risco CV. ^101^ As consequências da elevação da PA podem ser classificadas em precoces e tardias e englobam a maioria das DCV ( Quadro 2.1 ). A teoria de que a DCV decorre, predominantemente, de desvio à direita da distribuição da PA de toda humanidade foi recentemente proposta. ^102^

**Quadro 2.1:** – Consequências precoces e tardias da elevação crônica da PA **25**

Doenças de apresentação precoce e tardia
Acidente vascular encefálico Doença cardíaca coronária Insuficiência cardíaca Morte cardiovascular
**Doenças de apresentação tardia**
Cardiomiopatia hipertensiva Insuficiência cardíaca com fração de ejeção preservada Fibrilação atrial Cardiopatia valvar Síndromes aórticas Doença arterial periférica Doença renal crônica Demências Diabetes melito Disfunção erétil

Existem poucas evidências experimentais que demonstram a prevenção de longo prazo da PA elevada. A realização de ensaios clínicos para demonstrar a efetividade de intervenções ainda na fase inicial de elevação da PA e a consequente redução de desfechos é um projeto desafiador, pois exigiria longos períodos de intervenção. Apesar dessa limitação, demonstrou-se, no estudo SPRINT-Mind, que a estratégia de reduzir a PAS a valores inferiores a 120 mmHg associou-se à redução da incidência de desfecho composto por déficit cognitivo leve e demência ^103^ e de estigmas de Alzheimer na ressonância magnética. ^104^

À demonstração dos riscos da elevação da PA para a ocorrência de desfechos clínicos, adicionaram-se evidências dos danos vasculares e cardíacos pré-clínicos em valores de PA inferiores aos tradicionalmente utilizados para o diagnóstico de HA. Consequências cardíacas decorrentes dos níveis tensionais discretamente elevados, categorizadas como pré-hipertensão, foram demonstradas. ^105^ ^,^ ^106^ O estudo PREVER revelou que, nesses casos, a redução da PA leva à redução da massa ventricular estimada por eletrocardiograma, ^107^ além de reduzir em quase 50% a incidência de HA.

Estimativas de risco atribuível à elevação da PA para a incidência das doenças listadas ( Quadro 2.1 ) têm aumentado progressivamente em anos recentes, com a inclusão de valores mais baixos de PA nos modelos matemáticos. As mais conservadoras atribuem 49% dos infartos e 62% dos AVE à PA superior a 115/75 mmHg. ^108^

Muitas justificativas são aventadas para explicar sobrevidas centenárias. Curiosamente, mesmo em publicações em que se demonstra que HA e eventos CV ocorrem tardiamente nesses indivíduos, ^109^ não se atribuiu nexo causal entre aquelas condições. À luz do apresentado nesse capítulo, é natural deduzir que o envelhecimento vascular não é inexorável. ^102^ Assim, deduz-se que o fundamental para longas existências provavelmente consiste na manutenção de PA em valores realmente normais. Recentemente, demonstrou-se em um seguimento de 14 anos de participantes do estudo MESA ^110^ ( *Multi-Ethinic Study of Atherosclerosis* ), livres de outros fatores de risco CV, que a PAS superior a 100 mmHg aumentou o risco de eventos CV em três vezes, em comparação com os integrantes com PAS entre 90 e 99 mmHg.

O conjunto de evidências nos permite supor que, futuramente, os valores de referência para o diagnóstico de HA, assim como para as metas terapêuticas, possam ser modificados para níveis mais próximos do que consideramos como PA ótima ou normal. Entretanto, para que isso ocorra, ainda necessitamos de evidências mais sólidas e robustas.

## 2.3. Pressão Arterial, Inflamação e Disfunção Endotelial

A HA e suas complicações são mediadas por diversos mecanismos cujo traço comum é a disfunção endotelial, caracterizada pela baixa disponibilidade de óxido nítrico (NO) e pelo consequente desequilíbrio local entre fatores de relaxamento e constrição de arteríolas. ^111^ A disfunção endotelial decorre do desequilíbrio entre a produção de NO pela sintase endotelial (eNOS) ou a transformação do NO no radical livre peroxinitrito (NOO ^-^ ). ^112^ Nesse caso, a vasodilatação mediada por vários peptídeos, incluindo a bradicinina e angiotensina 1-7, é prejudicada, o que leva ao aumento da resistência vascular periférica e à alteração da permeabilidade endotelial. A instalação de um estado inflamatório crônico em pacientes com HA, pelo aumento na produção de citocinas pró-inflamatórias (como moléculas de adesão leucocitária, endotelina-1, angiotensina II), reduz a expressão da eNOS, ^113^ ^-^ ^115^ enquanto o aumento do estresse oxidativo acelera a degradação do NO. A baixa disponibilidade local de NO aumenta o tono do músculo liso vascular, induz a proliferação de células musculares lisas da camada média e aumenta a permeabilidade do endotélio. Isso facilita a passagem das lipoproteínas de baixa densidade (LDL-c) para o espaço subendotelial, que parece ser o evento inicial no desenvolvimento da aterosclerose. Dessa forma, a disfunção endotelial estaria na raiz de duas doenças crônicas que geralmente caminham juntas: a HA e a aterosclerose. Portanto, a identificação do grau de disfunção endotelial seria importante para a avaliação do curso clínico da HA. A nível bioquímico, a proteína C-reativa (PCR) ultrassensível parece ser o marcador mais adequado, e disponível clinicamente, para avaliar a disfunção endotelial.

Atualmente, a técnica mais utilizada em pesquisa clínica para a análise da função endotelial *in vivo* é a DMF medida na artéria braquial. ^116^ ^-^ ^119^ É um método não invasivo feito com ultrassonografia que se correlaciona com a função endotelial das coronárias ^120^ ^,^ ^121^ e prediz, de modo independente, a doença CV. ^121^ ^,^ ^123^ No entanto, sua disponibilidade é reservada. A dilatação dependente do endotélio ocorre pelo relaxamento da artéria braquial em resposta ao aumento do estresse de cisalhamento e da liberação local de NO. ^119^ A associação entre DMF e prognóstico CV é que ela reflete a biodisponibilidade do NO. ^124^ A DMF pode melhorar o poder preditivo do risco calculado pelos fatores de risco tradicionais, incluindo hipertensos jovens. ^119^ ^,^ ^123^ A opção por anti-hipertensivos que aumentam a biodisponibilidade do NO e estatinas pode representar uma opção interessante para o manejo clínico, ^5^ ^,^ ^37^ ^,^ ^125^ visando a melhorar ou preservar a função endotelial tanto de pacientes assintomáticos quanto naqueles com DAC estabelecida.

## 2.4. Pressão Arterial e Rigidez Arterial

A avaliação do dano vascular presente na HA tem sido cada vez mais incorporada na prática clínica. Tais danos envolvem alterações da microvasculatura, aterosclerose, aumento da rigidez arterial e disfunção endotelial. ^126^ No que diz respeito à rigidez arterial, esta provavelmente tem um componente genético, ^127^ mas também temos dois outros importantes determinantes: a idade e os níveis da PA. ^128^

A idade tem maior impacto nas artérias proximais (centrais), predominantemente elásticas, do que nas artérias periféricas, predominantemente musculares. As artérias centrais tornam-se mais rígidas com a idade, enquanto as musculares sofrem menos alterações. Com a idade, observamos fragmentação e degeneração da elastina e aumento progressivo do colágeno, acompanhado de depósito de cálcio na camada média arterial, com consequente aumento da rigidez arterial. ^128^ ^,^ ^129^

O aumento sustentado da PA é um gatilho para o desenvolvimento da hipertrofia da camada média da parede arterial, por promover alterações quantitativas e qualitativas de seus componentes (elastina, colágeno e células musculares lisas) que levam a adaptações mecânicas. ^127^ ^,^ ^128^ ^,^ ^130^ Tais achados foram descritos, tanto em modelos animais ^131^ quanto em estudos *in vitro* e culturas de órgãos *ex vivo,* ^132^ ^,^ ^133^ em que células mecanossensitivas responderam ao aumento de estresse com produção de matriz. Portanto, a HA acelera o envelhecimento vascular, uma resposta mecanobiológica local ao aumento de estresse induzido pelo aumento da PA e, consequentemente, da rigidez arterial (rigidez como consequência). ^128^ ^,^ ^134^

Entretanto, vários estudos demonstraram aumento da rigidez carotídea ou aórtica em indivíduos normotensos, apesar de a PA nestes ser normal. ^135^ ^-^ ^138^ Artérias mais rígidas representam maior impedância para a ejeção ventricular, o que exige maiores valores de pressão para manter constante o fluxo sanguíneo. Dessa maneira, tal aumento da rigidez arterial também é capaz de levar, a longo prazo, ao aumento da PA e, consequentemente, do risco CV. Os estudos demonstraram que a rigidez arterial poderia preceder a HA, estabelecendo a teoria da rigidez como causa. Humphey et al. (2016) ^134^ descreveram o mecanismo não como causa ou consequência, mas, sim, como causa e consequência, ou seja, um *feedback* positivo, um ciclo rigidez-HA-rigidez.

A avaliação do impacto da HA sobre a camada média das artérias pode ser feita por meio de biomarcadores que sejam capazes de detectar o dano e os diferentes graus de acometimento, o impacto na mortalidade, predizer eventos CV, adicionar informações aos fatores de risco estabelecidos, estratificar o risco suficientemente para mudar as recomendações terapêuticas e acrescentar informações que justifiquem seu custo adicional. ^139^ Os biomarcadores disponíveis para a avaliação da rigidez arterial estão relacionados nos tópicos a seguir.

### 2.4.1. Índice Tornozelo-Braquial (ITB)

O ITB é a razão entre a pressão sistólica no tornozelo e no braço. ^140^ É considerado um marcador de rigidez arterial em pacientes sem doença arterial periférica. ^141^ Pode ser realizado com o uso do Doppler, ou simplesmente através do método oscilométrico, mais barato e disponível, sendo que os valores mensurados através de ambos as técnicas têm uma boa correlação. ^142^ Segundo uma metanálise publicada em 2008, ^143^ um ITB ≤ 0,90 foi associado a aproximadamente o dobro de mortalidade idade-ajustada em 10 anos, mortalidade CV e maior taxa de eventos coronários. O uso do ITB resultou em uma reclassificação da categoria de risco CV e uma modificação da terapêutica em 19% dos homens e 36% das mulheres. ^143^ O ITB como preditor de risco cardiovascular é GR: IIa, NE: B.

### 2.4.2. Velocidade de Onda de Pulso (VOP)

A VOP tem sido considerada padrão-ouro na avaliação da rigidez arterial, não apenas pela facilidade em sua obtenção, mas também devido ao grande corpo de evidências demonstrando sua associação a doenças CV, independentemente dos fatores de risco tradicionais. ^144^ ^,^ ^145^

Calcula-se a VOP carotídeo-femoral (VOPc-f) dividindo-se a distância percorrida pelo tempo (VOPc-f= distância/tempo). O tempo pode ser medido diretamente na mesma onda de pulso ou indiretamente por meio da utilização do eletrocardiograma. A padronização de tal medida foi estabelecida pelo grupo europeu no documento de consenso publicado em 2012, ^146^ sendo um método não invasivo, robusto e validado.

Entre os métodos validados disponíveis atualmente, temos os que utilizam tonometria de pulso ^147^ ^,^ ^148^ e mecanotransdutores piezoelétricos ^149^ ^,^ ^150^ e oscilométricos. ^151^ ^,^ ^152^ Em 2015, a American Heart Association publicou um posicionamento sobre a padronização da utilização desses equipamentos para a avaliação da rigidez arterial. ^82^

O aumento da rigidez arterial é um preditor de desfechos. Isso foi demonstrado para a VOPc-f em pacientes hipertensos no início dos anos 2000 ^153^ ^,^ ^154^ e confirmado em vários estudos e subsequentemente em duas metanálises. ^155^ ^,^ ^156^ A primeira metanálise publicada em 2010, ^155^ com 15.877 pacientes de 17 estudos, demonstrou que o aumento de 1 m/s da VOP, com o risco ajustado para idade, sexo e fatores de risco, elevou em 14% os eventos CV, 15% a mortalidade CV e 15% a mortalidade por todas as causas. Além disso, um aumento de um desvio-padrão estaria associado a um aumento respectivo de 47%, 47% e 42%. A segunda metanálise, publicada em 2014, ^156^ com 17.635 pacientes, proveniente de 16 estudos, evidenciou que, para cada aumento em um desvio-padrão da VOP, ocorria aumento de risco de 35% para DAC, 54% para AVE e 45% para DCV.

Além de ser um preditor de desfechos, a adição da VOP aos fatores de risco CV tradicionais auxilia na estratificação. O primeiro estudo a demonstrar uma melhora na estratificação de risco ao se adicionar a VOP aos demais fatores de risco CV foi realizado em uma amostra da população geral da coorte de Framingham. ^157^ Posteriormente, a metanálise de Ben-Shlomo et al. (2014) ^156^ demonstrou um incremento em 13% na predição de risco em indivíduos com risco intermediário, quando a VOP foi adicionada.

Apesar da relevância da VOP na predição de eventos e na estratificação de risco, seu uso na prática clínica ainda é reduzido. Um grupo europeu publicou em 2019 um escore ^158^ baseado em variáveis clínicas que poderia identificar indivíduos com prioridade para a avaliação da VOP. Esse escore avalia variáveis clínicas facilmente disponíveis e foi denominado de SAGE, um acrônimo: S ( *systolic blood pressure* – pressão sistólica braquial), A ( *age* – idade), G ( *fasting plasma glucose* – glicemia de jejum) e E ( *estimated glomerular filtration rate* – estimativa da taxa de filtração glomerular). A elevação da VOP pode ser predita com acurácia por esse escore. Portanto, podemos priorizar a avaliação da rigidez arterial em pacientes hipertensos selecionados, melhorando a implementação do uso de tal medida na prática clínica.

O valor de corte de normalidade da VOP, na maioria dos estudos e diretrizes, é menor que 10 m/s. Entretanto, devido à influência da idade sobre a rigidez arterial, os valores de referências propostos atualmente levam em conta as diferentes faixas etárias e sexo, conforme estabelecidos pelo grupo europeu em 2010 ^144^ utilizando o método tonométrico, e mais recentemente em um trabalho nacional com o método oscilométrico ( Tabela 2.1 ). ^159^ O VOP como preditor de risco cardiovascular é GR: IIa, NE: A.

**Tabela 2.1:** – Valores de referência para pressão sistólica central e para velocidade de onda de pulso em população sem e com fatores de risco cardiovascular, Brazileira e europeia **69,84,85**

	População normal – sem fatores de risco cardiovascular				População com fatores de risco cardiovascular			
	Brazileira ^**1**^		Europeia ^**2**^		Brazileira ^**1**^		Europeia ^**2**^	
	Mulheres	Homens	Mulheres	Homens	Mulheres	Homens	Mulheres	Homens
**PASc**								
**< 30 anos**	101 (90-93-113-119)	113 (90-93-113-119)	95 (80-88-102-110)	103 (92-97-109-115)	118 (102-109-127-131)	123 (107-114-132-144)	101 (88-94-110-124)	110 (95-102-120-130)
**30-39 anos**	109 (96-102-117-123)	114 (96-102-117-123)	98 (84-90-108-119)	103 (88-05-112-120)	120 (102-110-130-143)	125 (108-116-133-141)	111 (92-100-127-141)	114 (95-103-129-144)
**40-49 anos**	110 (99-103-117-122)	116 (99-103-117-122)	102 (87-93-113-123)	106 (90-97-114-123)	121 (104-110-134-146)	123 (108-115-131-141)	116 (95-104-133-146)	118 (97-106-132-144)
**50- 59 anos**	110 (97-104-120-124)	112 (97-104-120-124)	110 (93-100-119-127)	110 (96-102-118-126)	124 (106-114-135-146	124 (105-114-134-144)	120 (100-109-134-148)	123 (102-111-137-150)
**60- 69 anos**	114 (100-103-121-126)	112 (100-105-120-125)	114 (97-105-122-129)	114 (97-105-122-128)	127 (105-115-141-154)	123 (103-112-136-149)	128 (105-115-141-154)	128 (105-115-142-155)
**≥ 70 anos**	113 (100-103-121-126)	116 (100-103-121-126)	118 (100-109-126-131)	116 (99-107-124-130)	131 (108-118-146-165)	125 (102-111-140-156)	138 (113-126-152-164)	135 (113-124-147-160)
**VOP**								
**< 30 anos**	4,9 (4,4-4,5-5,0-5,3)	5,2 (4,9-5,1-5,4-5,7)	6,1 (5,3-7,1)	5,3 (4,7-5,0-5,6-6,0)	5,3 (5,0-5,3-5,8-6,3)	6,7 (5,8-7,9)	7,2 (5,7-9,3)	7,6 (5,9-9,9)
**30- 39 anos**	5,4 (5,0-5,2-5,8-6,1)	5,7 (5,3-5,5-5,9-6,1)	6,4 (5,2-8,0)	5,8 (5,3-5,5-6,2-6,7)	6,1 (5,5-5,8-6,4-6,7)	7,0 (5,5-8,8)	7,2 (5,5-9,3)	7,6 (5,8-11,2)
**40- 49 anos**	6,4 (5,7-6,0-6,7-6,9)	6,5 (5,9-6,2-6,8-7,0)	6,9 (5,9-8,6)	6,8 (6,0-6,4-7,2-7,7)	6,8 (6,2-6,4-7,1-7,5)	7,7 (6,5-9,5)	8,1 (6,8-10,8)	9,2 (7,1-13,2)
**50- 59 anos**	7,5 (6,7-7,0-7,8-8,2)	7,4 (6,9-7,2-7,9-8,0)	8,1 (6,3-10,0)	7,9 (7,1-7,5-8,3-8,8)	7,9 (7,1-7,5-8,3-8,7)	8,4 (7,0-11,3)	9,2 (7,2-12,5)	9,7 (7,4-14,9)
**60-69 anos**	8,9 (8,1-8,5-9,2-9,4)	8,9 (8,2-8,6-9,1-9,6)	9,7 (7,9-13,1)	9,3 (8,4-8,8-9,8-10,4)	9,2 (8,4-8,7-9,7-10,2)	9,8 (7,9-13,2)	10,7 (8,4-14,1)	12,0 (8,5-16,5)
**≥ 70 anos**	11,3 (10,2-10,4-12,5-13,2)	11,0 (10,1-10,6-11,6-12,3)	10,6 (8,0-14,6)	11,8 (10,2-10,8-12,9-14,0)	11,2 (9,9-10,4-12,1-13,2)	11,2 (8,6-15,8)	12,7 (9,3-16,7)	13,5 (10,3-18,2)

^1^ referência Brazileira (oscilometria), ^2^ referência europeia de PASc, mediana (percentis 10º, 25º, 75º, 90º), ^3^ referência europeia de VOP (tonometria) mediana (pecentis 10º, 90º). Os valores de referência europeus para VOP não se dividem com relação ao sexo. PASc: pressão arterial sistólica central; VOP: velocidade de onda de pulso.

### 2.4.3. Pressão Central

A pressão central (aórtica, carotídea) não corresponde à pressão periférica (braquial), devido à amplificação de pulso que ocorre da aorta para a periferia e é mais relevante para a patogênese das doenças CV que a pressão periférica. ^160^ Atualmente, a pressão central pode ser facilmente acessada de maneira não invasiva, com os mesmos equipamentos utilizados e validados para a medida VOP. ^151^ ^,^ ^161^ ^,^ ^162^

Os índices hemodinâmicos centrais são preditores independentes de eventos CV futuros e mortalidade por todas as causas, segundo a metanálise de Vlachoupoulos et al., com 11 estudos e um total de 5.648 indivíduos, em um seguimento médio de 45 meses. ^160^ Os valores de referência para a pressão central foram estabelecidos pelo grupo europeu em 2014 usando o método tonométrico ^163^ e mais recentemente em um trabalho nacional com equipamentos oscilométricos ( Tabela 2.1 ). ^159^ A pressão central como preditor de risco cardiovascular é GR: IIa NE: B.

**Table:** 

	Grau de recomendação	Nível de evidência
Pressão arterial acima 120mmHg aumenta o dano vascular e o risco cardiovascular	I	A
Uso de marcadores séricos para a identificação de disfunção endotelial	IIb	B
Uso da DMF do sistema braquial arterial (técnica padrão-ouro para a análise da função endotelial in vivo) na identificação de disfunção endotelial	IIb	B
Uso da DMF para a estratificação de risco cardiovascular	IIb	B
Rigidez arterial avaliada pela VOP é um preditor independente de risco cardiovascular; e sua avaliação, quando possível, pode aumentar a acurácia dessa estratificação	IIa	A
ITB é preditor independente de risco cardiovascular	IIa	B
Pressão central é preditor independente de risco cardiovascular	IIa	B

**Table:** 

Mensagens principais
A pré-hipertensão aumenta o risco cardiovascular.
O dano vascular ocorre não apenas em hipertensos, mas pode estar presente também nos pré-hipertensos.
Há exames não invasivos para a avaliação precoce do dano vascular, mas nem sempre eles estão disponíveis.
A análise da rigidez arterial pela VOP é um preditor independente de risco cardiovascular; e sua avaliação pode ser realizada na prática clínica, quando disponível.
Outros métodos como o ITB e a medida central da pressão arterial também podem ser utilizados na avaliação do risco cardiovascular. A DMF é mais utilizada no âmbito da pesquisa.

## 3. Diagnóstico e Classificação

## 3.1. Introdução

A avaliação inicial de um paciente com hipertensão arterial (HA) inclui a confirmação do diagnóstico, a suspeita e a identificação de causa secundária, além da avaliação do risco cardiovascular (CV). As lesões de órgão-alvo (LOA) e as doenças associadas também devem ser investigadas. Fazem parte dessa avaliação: a medida da pressão arterial (PA) no consultório e/ou fora dele, utilizando-se técnica adequada e equipamentos validados e calibrados, a obtenção de história médica (pessoal e familiar), a realização de exame físico e a investigação clínica e laboratorial. Propõem-se avaliações gerais a todos os hipertensos e avaliações complementares apenas para grupos específicos. ^164^

## 3.2. Medida da Pressão Arterial no Consultório

A PA deve ser medida em toda avaliação por médicos, de qualquer especialidade, e por todos os profissionais da saúde devidamente capacitados. Exclusivamente aos médicos cabem o diagnóstico de HA e seus fenótipos, assim como a conduta relacionada a tais diagnósticos.

Os esfigmomanômetros auscultatórios ou oscilométricos são os métodos preferidos para medir a PA. Esses dispositivos devem ser validados de acordo com as condições e os protocolos padronizados, ^165^ e sua calibração deve ser verificada anualmente, no caso dos oscilométricos, e a cada seis meses no caso dos auscultatórios ou de acordo com as orientações do Inmetro/Ipem. ^166^ A PA deve ser inicialmente medida nos dois braços e idealmente estabelecida por medição simultânea. Caso ocorra uma diferença > 15 mmHg da PAS entre os braços, há o aumento do risco CV, ^167^ o qual pode estar relacionado com a doença vascular ateromatosa. Todas as medidas subsequentes devem ser realizadas no braço com valores mais elevados da PA. Na suspeita de HA secundária à coartação da aorta, a medida deverá ser realizada também nos membros inferiores, utilizando-se manguitos apropriados para a circunferência do braço ou da coxa ( Quadro 3.1 ). ^164^

**Quadro 3.1:** – Dimensões do manguito de acordo com a circunferência do membro

Circunferência	Denominação do manguito	Largura do manguito	Comprimento da bolsa
≤ 6 cm	Recém-nascido	3 cm	6 cm
6-15 cm	Criança	5 cm	15 cm
16-21 cm	Infantil	8 cm	21 cm
22-26 cm	Adulto pequeno	10 cm	24 cm
27-34 cm	Adulto	13 cm	30 cm
35-44 cm	Adulto grande	16 cm	38 cm
45-52 cm	Coxa	20 cm	42 cm

Fonte: Malachias et al., 2017. ^164^

**Quadro 3.2:** – Medida da pressão arterial no consultório

O paciente deve sentar-se confortavelmente em um ambiente silencioso por 5 minutos, antes de iniciar as medições da PA. Explique o procedimento ao indivíduo e oriente a não conversar durante a medição. Possíveis dúvidas devem ser esclarecidas antes ou depois do procedimento.
Certifique-se de que o paciente NÃO: •Está com a bexiga cheia; •Praticou exercícios físicos há, pelo menos, 60 minutos; •Ingeriu bebidas alcoólicas, café ou alimentos; •Fumou nos 30 minutos anteriores.
Três medidas de PA devem ser realizadas, com intervalo de 1 a 2 minutos; e medidas adicionais somente se as duas primeiras leituras diferirem em > 10 mmHg. Registre em prontuário a média das duas últimas leituras da PA, sem “arredondamentos” e o braço em que a PA foi medida.
Medidas adicionais podem ter que ser realizadas em pacientes com valores instáveis da PA devido a arritmias. Nos pacientes com FA, os métodos auscultatórios devem ser preferencialmente usados, pois a maioria dos dispositivos automáticos não foi validada para a medida da PA. ^*^
Use o manguito adequado para a circunferência do braço.
O manguito deve ser posicionado ao nível do coração. A palma da mão deve estar voltada para cima e as roupas não devem garrotear o braço. As costas e o antebraço devem estar apoiados; as pernas, descruzadas; e os pés, apoiados no chão.
Meça a PA nos dois braços na primeira visita, de preferência simultaneamente, para detectar possíveis diferenças entre os braços. Use o braço com o maior valor como referência.
Para pesquisar hipotensão ortostática, meça inicialmente a PA (de preferência, em posição supina, após o paciente estar nesta posição em repouso por 5 minutos; na impossibilidade de o indivíduo ficar na posição supina, pode-se de forma alternativa, embora não ideal, realizar a medida com o paciente sentado), e depois medir a PA 1 minuto e 3 minutos após a pessoa ficar em pé. As medições da PA em repouso e em pé devem ser realizadas em todos os pacientes na primeira consulta e também consideradas em visitas subsequentes em idosos, diabéticos, disautonômicos e pessoas em uso de anti-hipertensivo.
Registre a frequência cardíaca. Para excluir arritmia, use palpação do pulso.
Informe o valor de PA obtido para o paciente.

FA: fibrilação atrial; PA: pressão arterial. ^*^ A maioria dos dispositivos automáticos registra a forma de onda de pressão sistólica individual mais alta em vez de uma média de vários ciclos cardíacos em pacientes com FA, o que levando à superestimação da PA.

Em idosos, diabéticos, disautonômicos ou naqueles em uso de anti-hipertensivos, a PA também deve ser medida 1 minuto e 3 minutos após estar em pé (imóvel). ^168^ A hipotensão ortostática é definida como uma redução na PAS ≥ 20 mmHg ou na PAD ≥ 10 mmHg dentro do 3 ^o^ minuto em pé e está associada a um risco aumentado de mortalidade e eventos cardiovasculares. ^169^

Os Quadros 3.2 e 3.3 resumem os procedimentos e etapas recomendados para a medida adequada da PA. Enfatiza-se que a medida inadequada da PA pode levar a classificação imprecisa, superestimação ou subestimação da verdadeira PA do paciente e, consequentemente, tratamento desnecessário ou até mesmo ausência de tratamento em hipertensos mal avaliados. Diante da simplicidade da medida realizada pela técnica oscilométrica, a utilização de dispositivo oscilométrico de braço pode ser preferível ao auscultatório, quando as duas técnicas estiverem disponíveis. ^170^ As diferenças para a realização da medida da PA entre as duas técnicas são destacadas no Quadro 3.3 .

**Quadro 3.3:** – Etapas para a realização da medida da pressão arterial

Etapas
1. Determinar a circunferência do braço no ponto médio entre o acrômio e o olécrano.
2. Selecionar o manguito de tamanho adequado ao braço.
3. Colocar o manguito, sem deixar folgas, 2 a 3 cm acima da fossa cubital.
4. Centralizar o meio da parte compressiva do manguito sobre a artéria braquial.
5. Estimar o nível da PAS pela palpação do pulso radial.*
6. Palpar a artéria braquial na fossa cubital e colocar a campânula ou o diafragma do estetoscópio sem compressão excessiva.*
7. Inflar rapidamente até ultrapassar 20 a 30 mmHg o nível estimado da PAS obtido pela palpação.*
8. Proceder à deflação lentamente (velocidade de 2 mmHg por segundo).*
9. Determinar a PAS pela ausculta do primeiro som (fase I de Korotkoff) e, depois, aumentar ligeiramente a velocidade de deflação.*
10. Determinar a PAD no desaparecimento dos sons (fase V de Korotkoff).*
11. Auscultar cerca de 20 a 30 mmHg abaixo do último som para confirmar seu desaparecimento e, depois proceder, à deflação rápida e completa*.
12. Se os batimentos persistirem até o nível zero, determinar a PAD no abafamento dos sons (fase IV de Korotkoff) e anotar valores da PAS/PAD/zero.*

PAS: pressão arterial sistólica; PAD: pressão arterial diastólica. * Itens realizados exclusivamente na técnica auscultatória.

No estudo *Systolic Blood Pressure Intervention Trial* (SPRINT), ^86^ utilizou-se uma nova modalidade de medida da PA no consultório sem a presença do profissional de saúde, denominada medida desacompanhada da PA no consultório (MDPAC). Nessa modalidade, o paciente, depois de devidamente treinado, realiza sua própria medida em sala reservada para essa finalidade. Os participantes do SPRINT seguiram um protocolo no qual aguardavam em uma sala silenciosa por cinco minutos; em seguida, um aparelho automático realizava a medida a PA por três vezes, com intervalos de um minuto, registrando os valores obtidos. A MDPAC melhora a reprodutibilidade da medida da PA, e o efeito do avental branco pode ser substancialmente reduzido ou mesmo eliminado. ^171^ ^,^ ^172^ Na MDPAC, os valores obtidos são semelhantes ou mesmo inferiores aos obtidos pela medida ambulatorial da PA (MAPA) na vigília ou pela medida residencial da PA (MRPA). ^173^ Contudo, mostra-se fundamental lembrar que a medida convencional da PA no consultório é a base de todos os dados epidemiológicos e clínicos disponíveis atualmente.

Nos indivíduos obesos, o uso de um manguito com tamanho e forma ideais em relação ao braço do paciente é de importância primordial. A escolha do manguito apropriado depende não apenas da circunferência do braço, mas também de sua forma. ^174^ Manguitos mais longos e largos são necessários nesses pacientes para não haver superestimação da PA. A abordagem do antebraço deve ser considerada válida e pode ser usada em contextos clínicos para medir a PA, quando a medição do braço for desafiadora na obesidade grave (circunferência superior a 50 cm, em que não há manguito disponível). Assim, o pulso auscultado deve ser o radial, embora haja restrições a tal prática. ^175^ ^,^ ^176^ Ocorre uma especial dificuldade em braços largos e curtos, em forma de cone, nos quais manguitos de grandes dimensões não se adaptam. Nesses casos, o uso de monitores de pulso validados também pode ser considerado. ^177^ ^,^ ^178^

## 3.3. Classificação

Os limites de PA considerados normais são arbitrários. ^164^ ^,^ ^179^ Os valores que classificam o comportamento da PA em adultos por meio de medidas casuais ou de consultório estão expressos no Quadro 3.4 . São considerados hipertensos os indivíduos com PAS ≥ 140 mmHg e/ou PAD ≥ 90 mmHg. Quando utilizadas as medidas de consultório, o diagnóstico de HA deverá ser sempre validado por medições repetidas, em condições ideais, em duas ou mais visitas médicas em intervalo de dias ou semanas; ou de maneira mais assertiva, realizando-se o diagnóstico com medidas fora do consultório (MAPA ou MRPA), excetuando-se aqueles pacientes que já apresentem LOA ou doença CV. ^37^ Define-se a classificação de acordo com a PA do consultório e pelo nível mais elevado de PA, sistólica ou diastólica.

**Quadro 3.4:** – Classificação da pressão arterial de acordo com a medição no consultório a partir de 18 anos de idade

Classificação*	PAS (mHg)		PAD (mmHg)
**PA ótima**	< 120	e	< 80
**PA normal**	120-129	e/ou	80-84
**Pré-hipertensão**	130-139	e/ou	85-89
**HA Estágio 1**	140-159	e/ou	90-99
**HA Estágio 2**	160-179	e/ou	100-109
**HA Estágio 3**	≥ 180	e/ou	≥ 110

HA: hipertensão arterial; PA: pressão arterial; PAS: pressão arterial sistólica; PAD: pressão arterial diastólica. *A classificação é definida de acordo com a PA no consultório e pelo nível mais elevado de PA, sistólica ou diastólica. **A HA sistólica isolada, caracterizada pela PAS ≥ 140 mmHg e PAD < 90 mmHg, é classificada em 1, 2 ou 3, de acordo com os valores da PAS nos intervalos indicados. ***A HA diastólica isolada, caracterizada pela PAS < 140 mmHg e PAD ≥ 90 mmHg, é classificada em 1, 2 ou 3, de acordo com os valores da PAD nos intervalos indicados.

Indivíduos com PAS ≥ 140 mmHg e PAD > 90 mmHg são definidos como portadores de HA sistólica isolada, enquanto a presença de níveis de PAS > 140 mmHg e PAD ≥ 90 mmHg caracteriza a HA diastólica isolada. Tanto a HA sistólica isolada quanto a HA diastólica isolada apresentam maior prevalência de HA do avental branco (HAB). ^180^

Com relação à diretriz Brazileira anterior, ^164^ a PA normal passa a ser denominada PA ótima e a pré-hipertensão, a ser dividida em PA normal e pré-hipertensão. Os indivíduos com PAS entre 130 e 139 e PAD entre 85 e 89 mmHg passam a ser considerados pré-hipertensos, pois esta população apresenta consistentemente maior risco de doença CV, doença arterial coronária e acidente vascular encefálico do que a população com níveis entre 120 e 129 ou 80 e 84 mmHg. Há também maior risco de ser portadores de HA mascarada (HM). ^181^ ^,^ ^182^ Consequentemente, indivíduos pré-hipertensos devem ser monitorados mais de perto.

## 3.4. Medida da Pressão Arterial Fora do Consultório

A PA fora do consultório pode ser obtida através da MAPA ou da MRPA, respeitando-se suas indicações e limitações. ^183^ ^-^ ^187^ As medidas da PA fora do consultório devem ser estimuladas. As principais vantagens e desvantagens da medida da PA fora do consultório são resumidas no Quadro 3.5 , enquanto suas principais indicações, além das indicações específicas para a MRPA, são mostradas no Quadro 3.6 .

**Quadro 3.5:** – Vantagens e desvantagens da medida da pressão arterial fora do consultório

• Maior número de medidas obtidas • Refletem as atividades usuais dos examinandos • Pode identificar HA do avental branco e HA mascarada • Maior engajamento dos pacientes com o diagnóstico e o seguimento	
MAPA	MRPA
• Leituras noturnas • Permite medições em condições de vida real • Uso em pacientes com cognição prejudicada e nos raros casos de comportamento obsessivo • Permite avaliar a variabilidade da PA em períodos curtos de tempo • Evidência prognóstica mais robusta	• Baixo custo e amplamente disponível • Medição em um ambiente domiciliar, que pode ser mais relaxado do que o do consultório • Permite avaliar a variabilidade da PA no dia a dia • Envolvimento do paciente na medição da PA • Maior adesão ao tratamento
**• Custo elevado** **• Disponibilidade por vezes limitada** **• Pode ser desconfortável**	**• Somente PA em repouso** **• Potencial para erro de medição** **• Não tem leitura noturna**

HA: hipertensão arterial; MAPA: monitorização ambulatorial da pressão arterial; MRPA: monitorização residencial da pressão arterial; PA: pressão arterial.

**Quadro 3.6:** – Indicações para MAPA ou MRPA

MAPA ou MRPA
A pesquisa de HA do avental branco é mais comum, particularmente nas seguintes situações: • HA estágio 1 no consultório • Elevação acentuada da PA no consultório, com ausência de LOA
A pesquisa de HA mascarada é mais comum, particularmente nas seguintes situações: • Pré-hipertensão no consultório • PA normal no consultório em pacientes com LOA ou com alto risco CV
Confirmação do diagnóstico de HA resistente
Avaliação do controle da HA, especialmente em pacientes de alto risco CV
Indivíduos com resposta exacerbada da PA ao exercício
Presença de grande variabilidade da PA no consultório
Avaliação de sintomas sugestivos de hipotensão durante o tratamento
Indicações específicas para MAPA:
Avaliação da PA durante o sono e/o descenso vigília/sono (p. ex., suspeita de HA noturna, apneia obstrutiva do sono, doença renal crônica, diabetes, HA endócrina ou disfunção autonômica)
Investigação de hipotensão postural e pós-prandial em pacientes não tratados e tratados

HA: hipertensão arterial; PA: pressão arterial; LOA: lesão de órgão-alvo; MAPA: monitorização ambulatorial da pressão arterial; MRPA: monitorização residencial da pressão.

A MAPA e a MRPA não devem ser confundidas com a automedida da PA (AMPA), realizada com equipamento automático do próprio paciente, que não obedece a nenhum protocolo preestabelecido. As medidas são realizadas aleatoriamente e feitas por decisão do próprio paciente ou até a pedido médico. ^188^

A pandemia provocada pela covid-19 acelerou o processo de telemedicina (teleconsulta, teleorientação e telemonitoramento), que acreditamos ser irreversível. No momento presente, o SUS já realiza teleorientação sobre a covid-19, e algumas operadoras de saúde suplementar já a adotaram. Nesse cenário, a AMPA surge como uma possibilidade para contribuir no diagnóstico, no acompanhamento e no tratamento dos hipertensos. Para isso, sugere-se a utilização de equipamentos oscilométricos de boa qualidade, ou seja, validados e preferencialmente de braço. Caso seja utilizado o de punho, o que deve ser desestimulado, preferem-se aqueles validados, com sensor de altura e movimento. Sugere-se um número mínimo de sete medidas realizadas no período de 16 a 72 horas. Até o momento, sugerem-se valores de normalidade iguais aos da MRPA, embora estudos específicos ainda precisam ser realizados para comparar os valores de PA obtidos pelas diferentes técnicas. ^187^ ^,^ ^189^

A definição de HA de acordo com a PA no consultório está mostrada no Quadro 3.7 . Quando comparados com os valores da PA no consultório, os valores da MRPA são geralmente mais baixos, e o limiar de diagnóstico para HA é ≥ 130/80 mmHg (equivalente à PA no consultório ≥ 140/90 mmHg). ^180^ ^,^ ^190^ ^-^ ^192^ A MRPA fornece valores de PA mais reprodutíveis e está mais fortemente relacionada com a LOA, particularmente à hipertrofia ventricular esquerda, e a predição de morbimortalidade CV do que a PA do consultório. ^188^ ^,^ ^193^ Há também evidências de que a MRPA pode ter um efeito benéfico na adesão à medicação e no controle da PA, ^194^ ^,^ ^195^ especialmente quando combinada com orientação e aconselhamento. ^196^ O telemonitoramento e os aplicativos de *smartphone* podem oferecer vantagens adicionais à MRPA, ^197^ ^,^ ^198^ como auxílio à memória das medidas da PA e uma maneira conveniente de armazenar e editar os dados da PA em laudo digital.

**Quadro 3.7:** – Definição de hipertensão arterial de acordo com a pressão arterial de consultório, monitorização ambulatorial da pressão arterial e monitorização residencial da pressão arterial

Categoria	PAS (mmHg)		PAD (mmHg)
**PA no consultório**	≥ 140	e/ou	≥ 90
**MAPA 24 horas**	≥ 130	e/ou	≥ 80
**Vigília**	≥ 135	e/ou	≥ 85
**Sono**	≥ 120	e/ou	≥ 70
**MRPA**	≥ 130	e/ou	≥ 80

HA: hipertensão arterial; PA: pressão arterial; PAS: pressão arterial sistólica; PAD: pressão arterial diastólica; MAPA: monitorização ambulatorial da pressão arterial; MRPA: monitorização residencial da pressão arterial.

A MAPA é melhor preditor de risco CV e de LOA do que a PA do consultório. ^199^ Além disso, a média ambulatorial da PA de 24 horas demonstrou ter uma melhor relação com eventos não fatais ou fatais, ^200^ ^,^ ^201^ como eventos coronários fatais e não fatais e acidente vascular encefálico. ^202^ ^-^ ^205^

## 3.5. Efeito do Avental Branco (EAB) e Efeito de Mascaramento (EM)

A diferença da PA entre as medidas obtidas no consultório e fora dele é denominada EAB ou EM, quando seus valores são, respectivamente, positivos ou negativos. Com base em estudos de MRPA, diferenças iguais ou superiores a 15 mmHg na PAS e/ou 9 mmHg na PAD indicam EAB significativa, enquanto diferenças iguais ou inferiores a -1 mmHg na PAS e/ou PAD indicam EM significativa. ^180^ Essas situações não mudam o diagnóstico, ou seja, se o indivíduo é normotenso, permanecerá normotenso; e, se é hipertenso, continuará sendo hipertenso. Contudo, talvez seja útil para identificar indivíduos com risco de ter diferenças relevantes na PA dentro e fora do consultório, o que pode contribuir para um melhor manejo terapêutico.

## 3.6. Hipertensão do Avental Branco (HAB) e Hipertensão Mascarada (HM)

**Figura 3.1:**
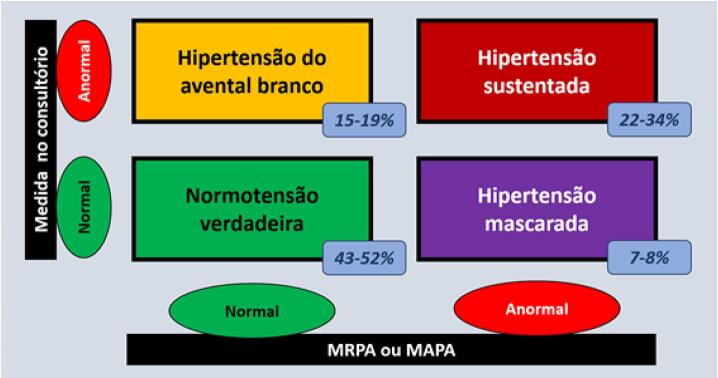
– Diagnósticos possíveis na hipertensão arterial (fenótipos).

MAPA: monitorização ambulatorial da pressão arterial; MRPA: monitorização residencial da pressão arterial.

No diagnóstico da HA, são possíveis vários fenótipos. Define-se a normotensão verdadeira (NV) como as medidas da PA no consultório e fora do consultório normais, a HA sustentada (HS) quando ambas são anormais, a HAB quando a PA é elevada no consultório, mas é normal fora dele, e HM quando a PA é normal no consultório, mas é elevada fora dele. ^206^ ^,^ ^207^ Suas prevalências estimadas no Brazil são demonstradas na Figura 3.1 . ^208^ ^,^ ^209^

Fonte: Adaptado de Datasus/MS/SVS/CGIAE 2017. ^23^

Fonte: Nilson et al., 2020.29

Embora a prevalência varie entre os estudos, a HAB pode ser detectada em cerca de 15 a 19% dos indivíduos no consultório, alcançando 30 a 40% naqueles com PA elevada no consultório. É mais comum nos pacientes com HA estágio 1. ^210^ ^-^ ^212^

A presença de LOA e o risco de eventos CV associados à HAB são menores do que na HS. ^205^ ^,^ ^213^ ^,^ ^214^ No entanto, em comparação com a NV, a HAB está associada a maior atividade adrenérgica, maior prevalência de fatores de risco metabólicos, LOA mais frequente e maior risco para desenvolver diabetes melito e progressão para HS e hipertrofia ventricular esquerda. ^215^ ^,^ ^216^ Na HAB, os valores da PA fora do consultório tendem a ser mais altos do que na NV, o que pode explicar o aumento do risco a longo prazo de eventos CV. ^217^ ^-^ ^221^

Assim como a HAB, a prevalência de HM pode variar bastante entre as populações. Contudo, de maneira geral, a HM pode ser detectada em cerca de 7 a 8% dos indivíduos no consultório, podendo atingir aproximadamente 15% dos pacientes normotensos. ^222^ ^,^ ^223^ Vários fatores podem elevar a PA fora do consultório com relação à PA do consultório, como idade avançada, sexo masculino, tabagismo, consumo de álcool, atividade física, HA induzida pelo exercício, ansiedade, estresse, obesidade, diabetes melito, doença renal crônica e história familiar de HA. A HM está associada à dislipidemia, à disglicemia, à LOA, à pré-hipertensão e à atividade adrenérgica e aumenta o risco de desenvolver diabetes melito e HS. ^183^ ^,^ ^185^ ^,^ ^198^ ^,^ ^207^ ^,^ ^224^ ^-^ ^226^ Metanálises de estudos prospectivos indicam que a incidência de eventos CV é cerca de duas vezes maior na HM do que na NV, sendo comparável à da HA. ^210^ ^,^ ^227^ ^,^ ^228^

## 3.7. Hipertensão Mascarada Não Controlada e Hipertensão do Avental Branco Não Controlada

**Figura 3.2:**
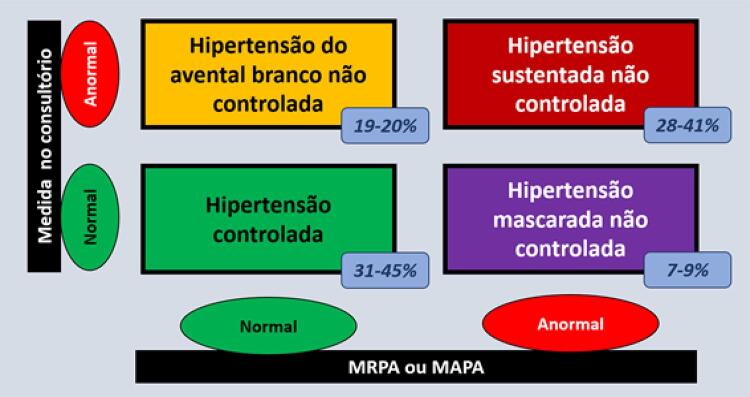
– Fenótipos em hipertensos tratados.

MAPA: monitorização ambulatorial da pressão arterial; MRPA: monitorização residencial da pressão arterial.

Os termos HAB e HM foram originalmente definidos para pessoas que não estavam sendo tratadas para HA. Entretanto, em pacientes em uso de medicações anti-hipertensivas também pode haver comportamentos discrepantes da PA dentro e fora do consultório. Nesse contexto, utilizam-se as seguintes nomenclaturas para os pacientes tratados com anti-hipertensivos: HA mascarada não controlada, quando a PA está controlada no consultório, mas elevada fora dele; HA do avental branco não controlada quando a PA está elevada no consultório, mas normal fora dele; HA sustentada não controlada, quando a PA no consultório e a fora dele estão elevadas; e HA controlada, quando a PA está normal no consultório e fora dele. ^218^ As prevalências desses fenótipos no Brazil são demonstradas na Figura 3.2 . ^213^ ^,^ ^214^

## 3.8. Recomendações para Diagnóstico e Seguimento

**Figura 3.3:**
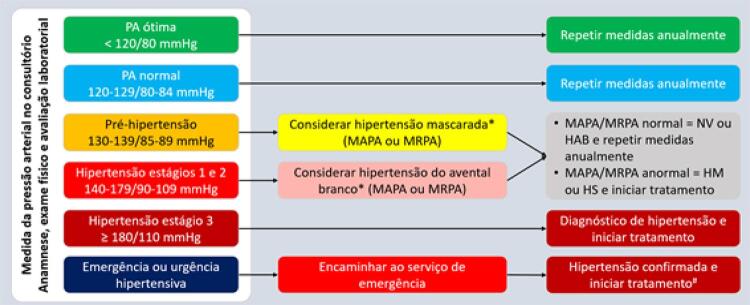
– Triagem e diagnóstico de hipertensão arterial.

PA: pressão arterial; MAPA: monitorização ambulatorial da pressão arterial; MRPA: monitorização residencial da pressão arterial; NV: normotensão verdadeira; HAB: hipertensão do avental branco; HM: hipertensão mascarada; HS: hipertensão sustentada

A HA é uma condição habitualmente assintomática. Por isso, deve ser avaliada em todo atendimento médico e em programas estruturados de triagem populacional. Nestes últimos, mais de 50% dos portadores de HA não sabiam que tinham a doença. ^229^ ^,^ ^230^ As medidas da PA devem ser realizadas em intervalos regulares, com a frequência conforme a classificação de PA ( Figura 3.3 ). Pessoas saudáveis com uma PA ótima no consultório (< 120/80 mmHg) ou com PA normal (120-129/80-84 mmHg) devem ter a PA medida novamente pelo menos anualmente e nas consultas médicas. Pacientes com pré-hipertensão (130-139/85-89 mmHg) devem ter a PA medida anualmente ou, preferencialmente antes, devido às altas taxas de progressão para HA. Além disso, nos casos suspeitos de HM, a MAPA ou a MRPA devem ser realizadas para detectar tal fenótipo.

Como a PA pode ter alta variabilidade, o diagnóstico de HA não deve se basear exclusivamente na medida da PA em apenas uma consulta médica, a menos que esteja substancialmente elevada (HA estágio 3) ou haja diagnóstico estabelecido de LOA ou de doença CV. Para os demais pacientes, as medidas repetidas da PA em visitas subsequentes no consultório devem ser utilizadas para confirmar uma elevação persistente, bem como para classificar o estágio da HA. Quanto maior o estágio da HA, maior deverá ser o número de visitas e menor o intervalo de tempo entre elas. Assim, pacientes em estágio 2 ou 3 poderão requerer mais visitas com intervalos de tempo mais curtos entre as visitas (dias ou semanas), enquanto aqueles com estágio 1 poderão requerer visitas após alguns meses, especialmente quando não há LOA e o risco CV é baixo.

A diretriz recomenda o uso de medidas de PA fora do consultório ( Figura 3.3 ) como uma estratégia alternativa às avaliações repetidas da PA dentro dele para confirmar o diagnóstico de HA, desde que sejam logística e economicamente viáveis. ^231^ Tal abordagem pode também gerar informações clínicas complementares relevantes, como a detecção de HAB e HM ^213^ ^,^ ^214^ ^,^ ^232^ ( Quadro 3.6 e Figura 3.3 ).

O teste ergométrico não é recomendado para a avaliação diagnóstica da HA, devido a várias limitações, inclusive a falta de padronização da metodologia e das definições. Atualmente, não há consenso sobre a resposta normal da PA durante o exercício.

## 3.9. Pressão Aórtica Central

Várias técnicas têm permitido a medida da PA aórtica (PA central) por meio de algoritmos específicos a partir de medidas de PA obtidas no braço. ^233^ ^,^ ^159^ Vários estudos mostraram reduções diferentes na PA central em comparação com a PA braquial com o uso de alguns anti-hipertensivos e, embora a PA central pareça ser um melhor preditor de eventos CV do que a PA braquial, o valor prognóstico incremental da medida da PA central ainda necessita de mais evidências. ^160^ ^,^ ^234^

A HA espúria (HA sistólica isolada nos jovens com PA central normal) parece ser a condição mais evidente para o uso da PA central (quando disponível) na prática clínica, o que configura a primeira indicação da medida da PA central. Isso ocorre em uma pequena fração de jovens, principalmente homens atletas, e ainda não está claro se esses pacientes estão sob menor risco CV do que o sugerido pela PA convencional. ^235^ ^-^ ^237^

É importante ressaltar que as limitações do valor prognóstico da PA central não se aplicam a parâmetros associados a tais medidas, como a velocidade de onda de pulso (VOP) e o *augmentation index* (AIx), cujos valores prognósticos estão bem estabelecidos. ^238^

## 3.10. Genética e Hipertensão Arterial

Considera-se a HA primária uma doença multifatorial, mas com forte componente genético. Estudos em famílias e em gêmeos demonstram uma herdabilidade de 30 a 50%. ^239^ ^,^ ^240^ A maioria desse risco genético é transmitido de forma poligênica, ou seja, através da contribuição de centenas de variações de DNA que, em conjunto, aumentam o risco de desenvolvimento do fenótipo hipertensivo, após interação com fatores ambientais. Um estudo recente com mais de 1 milhão de pacientes demonstrou que variações de DNA em mais de 900 genes estão associadas ao controle da PA, o que explica cerca de 27% da herdabilidade do controle da PA. ^240^ Esse estudo abre caminho para o uso futuro de painéis genéticos de avaliação de risco de HA, que poderiam ajudar a guiar os esforços preventivos.

Em contraste com a HA primária, diversas formas secundárias de HA são causadas por mutação em um gene único (HA monogênica), de herança familiar, como o hiperaldosteronismo familiar, a síndrome de Liddle, a hiperplasia adrenal congênita e as formas herdadas de feocromocitoma e paraganglioma ( Quadro 3.8 ). ^240^ ^,^ ^241^ Essas causas devem ser investigadas nos pacientes com suspeita de HA secundária. O diagnóstico genético preciso pode levar não somente ao tratamento adequado, como também permitir o aconselhamento genético familiar e o rastreio precoce em membros assintomáticos da família.

**Quadro 3.8:** – Causas de hipertensão monogênica

Condição	Modo de herança	Genes envolvidos
**Síndrome de Liddle**	Autossômico dominante	SCNN1B e SCNN1G
**Hiperplasia adrenal congênita**	Autossômico recessivo	CYP11B1
	Autossômico recessivo	CYP17A1
**Síndrome de aparente excesso mineralocorticoide**	Autossômico recessivo	HSD11B2
**Síndrome de Geller**	Autossômico dominante	NR3C2
**Síndrome de Gordon (pseudo-hipoaldosteronismo tipo II)**	Autossômico dominante	WNK4
	Autossômico dominante	WNK1
	Autossômico recessivo ou dominante	KLHL3
	Autossômico dominante	CUL3
**Hiperaldosteronismo familiar tipo I**	Autossômico dominante	CYP11B1
**Hiperaldosteronismo familiar tipo II**	Autossômico dominante	CLCN2
**Hiperaldosteronismo familiar tipo III**	Autossômico dominante	KCNJ5
**Hiperaldosteronismo familiar tipo IV**	Autossômico dominante	CACNA1H

**Table:** 

Recomendação	GR	NE
Recomenda-se que a PA seja classificada como HA ótima, normal, pré-hipertensão ou estágios 1 a 3, de acordo com a PA do consultório.	I	C
Programas de triagem para HA são recomendados. Todos os adultos (≥ 18 anos) devem ter a PA no consultório medida e registrada em seu prontuário médico e estar cientes da PA. ^160^ ^,^ ^234^	I	B
Diante da simplicidade da medida realizada pela técnica oscilométrica, a utilização de dispositivo automático de braço pode ser preferível ao auscultatório, quando as duas técnicas estiverem disponíveis.	I	C
Indica-se a medida da PA, anualmente, se a PA do consultório for > 140/90 mmHg.	I	C
Recomenda-se que a PA no consultório seja medida em ambos os braços pelo menos na primeira consulta, porque uma diferença de PAS entre os braços > 15 mmHg é sugestiva de doença ateromatosa e está associada a um risco CV aumentado. ^167^	I	A
Se uma diferença entre os braços < 15mmHg da PA for registrada, recomenda-se que todas as leituras subsequentes da PA usem o braço com uma leitura mais alta da PA.	I	C
Recomenda-se que o diagnóstico de HA seja baseado em medições repetidas da PA em consultório em mais de uma consulta, exceto quando é HA estágio 3 e especialmente em pacientes de alto risco. Em cada consulta, três medidas da PA devem ser registradas, com 1 a 2 minutos de intervalo, e medidas adicionais devem ser realizadas se as duas primeiras leituras diferirem em > 10 mmHg. A PA do paciente é a média das duas últimas leituras da PA.	I	C
Recomenda-se que o diagnóstico de HA seja baseado em medição de PA fora do consultório com MAPA e/ou MRPA , desde que essas medidas sejam viáveis (logisticamente e economicamente).	I	C
A PA fora do consultório (ou seja, MAPA ou MRPA ) é especificamente recomendada para várias indicações clínicas, como identificação da HAB e HM, quantificação dos efeitos do tratamento e identificação de possíveis causas de efeitos colaterais (p. ex., hipotensão sintomática). ^164^ ^,^ ^170^ ^,^ ^180^ ^,^ ^201^ ^,^ ^209^	I	A
A pressão de pulso, a variabilidade da PA e a PA central podem ser consideradas, mas atualmente são pouco utilizadas para o uso clínico de rotina. Podem fornecer informações adicionais úteis em algumas circunstâncias e são ferramentas valiosas para a pesquisa.	IIb	C
O teste genético deve ser considerado em centros especializados para pacientes com suspeita de causas raras monogênicas de HA secundária ou para aqueles com feocromocitoma. ^240^ ^-^ ^242^	IIa	B
O teste genético de rotina para pacientes hipertensos não é recomendado.	III	C

PA: pressão arterial; CV: cardiovascular; HAB: hipertensão do avental branco; HM: hipertensão mascarada; MAPA: monitorização ambulatorial da pressão arterial; MRPA: monitorização residencial da pressão arterial.

**Table:** 

Mensagens principais
Recomenda-se que a PA seja classificada como ótima, normal, pré-hipertensão ou estágios 1 a 3, de acordo com a PA do consultório.
Programas de triagem para HA são recomendados. Todos os adultos (≥ 18 anos) devem ter a PA medida no consultório e registrada em seu prontuário médico e estar cientes dos valores aferidos.
Indica-se a medida da PA, anualmente, se a PA do consultório > 140/90 mmHg.
Recomenda-se que o diagnóstico de HA seja baseado em medições repetidas da PA em consultório, em mais de uma consulta, ou pela medida de PA fora do consultório com MAPA e/ou MRPA, desde que essas medidas sejam viáveis.
A PA fora do consultório (ou seja, MAPA ou MRPA) é especificamente recomendada para várias indicações clínicas, como identificação da HAB e da HM, quantificação dos efeitos do tratamento e identificação de possíveis causas de efeitos colaterais (p. ex., hipotensão sintomática).

## 4. Avaliação Clínica e Complementar

## 4.1. História Clínica

A avaliação clínica do paciente hipertenso deve ser feita seguindo-se o método tradicional, constituído por anamnese, exame físico e laboratorial. O Quadro 4.1 contempla um resumo dos objetivos. O seguimento de todas as etapas permitirá o diagnóstico correto da hipertensão arterial (HA) e estratificar o risco cardiovascular e renal, contribuindo para estabelecer a estratégia terapêutica mais adequada.

**Quadro 4.1:** – Avaliação clínica e laboratorial

Realizar medidas acuradas da PA para a confirmação diagnóstica de HA (Capítulo 2)
Questionar sobre história familiar de HA
Identificar fatores de risco cardiovasculares e renais associados
Pesquisar LOA (subclínicas ou manifestas clinicamente)
Investigar a presença de outras doenças
Questionar sobre fármacos e drogas que possam interferir na PA
Aplicar escore de risco CV global (Capítulo 5)
Rastrear indícios de HA secundária (Capítulo 15)

PA: pressão arterial; HA: hipertensão arterial; LOA: lesões em órgãos-alvo; CV: cardiovascular.

## 4.2. Avaliação Clínica

### 4.2.1. Anamnese

Deve ser realizada anamnese, com história clínica completa, perguntas obrigatórias sobre o tempo de diagnóstico e tratamentos anti-hipertensivos instituídos previamente (medicamentos e doses). Além disso, convém apurar os sintomas que indiquem a evolução da doença hipertensiva, especialmente a presença de lesão de órgãos-alvo (LOA). São importantes os antecedentes pessoais e a construção de uma linha de tempo que permita melhor compreensão do quadro clínico.

A história familiar também deve ser obtida para corroborar o diagnóstico de HA primária ^243^ (GR: I; NE: B). Durante a consulta, deve ser questionada, entre outros, a existência de: fatores de risco específicos para doença cardiovascular (DCV) e renal, ^244^ ^-^ ^246^ comorbidades e aspectos biopsicossociais, culturais e socioeconômicos. ^244^ ^,^ ^245^ ^,^ ^247^ É fundamental avaliar o uso de outros medicamentos, fármacos e drogas lícitas e ilícitas, que não anti-hipertensivos, que possam interferir na PA (Capítulo 9), bem como pesquisar indícios na história clínica que sugiram causas secundárias de HA, conforme detalhado no Capítulo 15.

## 4.3. Exame Físico

Um exame físico minucioso deve ser realizado, com medida correta e repetida da PA e da frequência cardíaca (FC), como já descrito no Capítulo 3, além de se procurar sinais de LOA e de achados que possam sugerir causas secundárias de HA.

Os dados antropométricos, peso e altura, assim como o cálculo do índice de massa corporal (IMC) ^248^ e da circunferência abdominal (CA), ^248^ têm seus valores de normalidade definidos pela *World Obesity Federation* (disponíveis *on-line* em https://www.worldobesity.org/. A avaliação deve contemplar palpação e ausculta cardíaca e de carótidas, além de verificação dos pulsos. A medida do índice tornozelo braquial (ITB) também é incentivada, assim como a fundoscopia. ^249^ ^,^ ^250^ Realiza-se o cálculo do ITB por meio da razão entre a pressão arterial sistólica (PAS) do braço e a do tornozelo, tanto esquerdo quanto direito. A relação PAS braço/PAS tornozelo normal é acima de 0,90. A obstrução leve caracteriza-se por ITB entre 0,71-0,90; moderada 0,41-0,70; e grave 0,00-0,40 (GR: IIa, NE: B). É ferramenta importante tanto para o diagnóstico de doença arterial obstrutiva periférica quanto para o prognóstico de eventos cardiovasculares. ^250^

Em alguns casos, pode ser indicada a medida da pressão arterial sistólica central (PASc) com o intuito de detectar a hipertensão sistólica isolada no jovem (hipertensão espúria no jovem), pois, diferentemente da medida na artéria braquial, a PASc não se encontra elevada (GR: IIa, NE: B) (Capítulo 3). ^251^ ^,^ ^252^ No Quadro 4.2 , pode ser encontrado um resumo do exame físico.

**Quadro 4.2:** – Avaliação do exame físico

1. Obter medidas repetidas e acuradas em ambos os braços da PA (vide Capítulo 3)
2. Medir parâmetros antropométricos: peso, altura, FC, CA e cálculo do IMC
3. Procurar sinais de lesões em órgãos-alvo
4. Detectar características de doenças endócrinas como Cushing, hiper ou hipotireoidismo
5. Examinar a região cervical: palpação e ausculta das artérias carótidas, verificação de estase jugular e palpação de tireoide
6. Avaliar o aparelho cardiovascular: desvio de ictus e propulsão à palpação; na ausculta, presença de B3 ou B4, hiperfonese de segunda bulha, sopros e arritmias
7. Avaliar o sistema respiratório: ausculta de estertores, roncos e sibilos
8. Observar as extremidades: edemas, pulsos em membros superiores e inferiores (na presença de pulso femorais diminuídos, sugere coartação de aorta, doença da aorta ou ramos)
9. Palpar e auscultar o abdômen: frêmitos, sopros, massas abdominais indicativas de rins policísticos e tumores (podem sugerir causas secundárias ou LOA)
10. Detectar déficits motores ou sensoriais no exame neurológico
11. Realizar fundoscopia ou retinografia (quando disponível): identificar aumento do reflexo dorsal, estreitamento arteriolar, cruzamentos arteriovenosos patológicos, hemorragias, exsudatos e papiledema (sinais de retinopatia hipertensiva)

PA: pressão arterial, FC: frequência cardíaca; CA: circunferência abdominal; IMC: índice de massa corpórea.

### 4.3.1. Investigação Laboratorial Básica, Avaliação de Lesões Clínicas e subclínicas em Órgãos-Alvo

A avaliação complementar tem como objetivo detectar lesões clínicas ou subclínicas em órgãos-alvo, no sentido de melhor estratificar o risco cardiovascular (CV). Para a estratificação do risco CV global, deverão ser levados em conta, além dos fatores de risco clássicos ( Quadro 4.3 ), os novos fatores de risco que vêm sendo identificados, embora nem todos ainda não tenham sido incorporados em escores clínicos. ^253^ ^,^ ^254^ Nesta investigação, destacam-se alterações da glicemia ou da hemoglobina glicada, a obesidade abdominal (síndrome metabólica), a pressão de pulso > 65 mmHg em idosos, a história de pré-eclâmpsia/eclâmpsia e a história familiar de HA (em hipertensos limítrofes). ^254^

**Quadro 4.3:** – Fatores de risco cardiovascular adicionais

Idade (mulher > 65 anos e homem > 55 anos)
Tabagismo
Dislipidemia: triglicerídeos (TG) > 150 mg/dL em jejum; LDL-c > 100 mg/dL; HDL-c < 40 mg/dL
**Diabetes melito** (DM) já confirmado (glicemia de jejum de, pelo menos, 8 horas ≥ 126 mg/dL, glicemia aleatória ≥ 200 mg/dL ou HbA1c ≥ 6,5%) ou pré-diabetes (glicemia de jejum entre 100 e 125 mg/dL ou HbA1c entre 5,7 e 6,4%)
História familiar prematura de DCV: em mulher < 65 anos e homem < 55 anos
Pressão de pulso em idosos (PP = PAS – PAD) > 65 mmHg
ITB ou VOP anormais
História patológica pregressa de pré-eclâmpsia ou eclâmpsia
Obesidade central: IMC < 24,9 Kg/m ^2^ (normal); entre 25 e 29,9 Kg/m ^2^ (sobrepeso); > 30 Kg/m ^2^ (obesidade)
Relação cintura/quadril (C/Q)
Cintura abdominal = mulher < 88 cm e homem < 102 cm Cintura: C = no ponto médio entre a última costela e a crista ilíaca lateral Quadril Q = ao nível do trocanter maior Cálculo (C/Q) = mulher: C/Q ≤ 0,85; homens: C/Q ≤ 0,95
Perfil de síndrome metabólica
C: cintura, Q: quadril, C/Q: relação cintura quadril, ITB: índice tornozelo braquial, PP: pressão de pulso, IMC: índice de massa corpórea.

A alteração da velocidade de onda de pulso (VOP), quando disponível, é um exame que denota LOA, podendo reclassificar os pacientes de risco CV intermediário para risco elevado (GR: IIa, NE: A) (Capítulo 2). ^156^ A avaliação laboratorial básica (Quadros 4.4 e 4.5 ) deve fazer parte da rotina inicial de todo paciente hipertenso. ^253^ São recomendadas: dosagem sérica de potássio, ácido úrico, creatinina, glicemia e perfil lipídico; e realização de um exame sumário de urina e de eletrocardiograma, para possível detecção de hipertrofia ventricular esquerda.

**Quadro 4.4:** – Exames complementares de rotina

Análise de urina (GR: I, NE: C)
Potássio plasmático (GR: I, NE: C)
Creatinina plasmática (GR: I, NE: B)
Glicemia de jejum (GR: I, NE: C) e HbA1c (GR: I, NE: C)
Estimativa do ritmo de filtração glomerular (GR: I, NE: B)
Colesterol total, HDLc e triglicerídeos plasmáticos (GR: I, NE: C) *
Ácido úrico plasmático (GR: I, NE: C)
Eletrocardiograma convencional (GR: I, NE: B) **

* O LDLc é calculado pela seguinte fórmula: LDLc = colesterol total – (HDLc + triglicerídeos/5) (quando a dosagem de triglicerídeos for abaixo de 400 mg/dL). ^259^ * O LDLc também tem sido dosado por alguns laboratórios, fazendo parte da rotina laboratorial. ** Critério de detecção de HVE – Sokolow-Lyon: SV _1_ + RV _5,6_ > 35 mm – Cornell Voltagem: RaVL + SV _3_ > 20 mm (mulher), > 28 mm (homem). ^260^ ^,^ ^261^

**Quadro 4.5:** – Exames recomendados a populações indicadas.

Radiografia de tórax: tem indicação no acompanhamento do paciente hipertenso nas situações de suspeita clínica de acometimento cardíaco (GR: IIa, NE: C) e/ou pulmonar ou para a avaliação de hipertensos com acometimento de aorta em que o ecocardiograma não está disponível. ^262^
Ecocardiograma: é mais sensível que o eletrocardiograma quanto ao diagnóstico de hipertrofia do ventrículo esquerdo (HVE) e agrega valores na avaliação de formas geométricas de hipertrofia e tamanho do átrio esquerdo, nas funções sistólica e diastólica. Está indicado quando houver indícios de HVE no eletrocardiograma ou em pacientes com suspeita clínica de insuficiência cardíaca (GR: IIa, NE: B). Considera-se HVE quando a massa do ventrículo esquerdo indexada para a superfície corpórea é igual ou superior a 116 g/m ^2^ no homem e 96 g/m ^2^ na mulher. ^263^
Albuminuria ou relação proteinúria/creatininúria ou albuminúria/creatininúria: exame útil para os hipertensos diabéticos, com síndrome metabólica ou com dois ou mais fatores de risco, pois mostrou prever eventos cardiovasculares fatais e não fatais (valores normais < 30 mg/g de creatinina (GR: I, NE: B). ^264^
Ultrassonografia de carótidas: indicado na presença de sopro carotídeo, sinais de doença cerebrovascular ou presença de doença aterosclerótica em outros territórios. O aumento na espessura íntima-média (EIM) das carótidas e/ou a identificação de placas de aterosclerose prediz a ocorrência de acidentes vasculares cerebrais e infarto do miocárdio, independentemente de outros fatores de risco CV. Valores da EIM > 0,9 mm têm sido considerados como anormais, assim como o encontro de placas ateroscleróticas (GR: I, NE: A). ^265^ ^,^ ^266^
Ultrassonografia renal ou com Doppler: necessária em pacientes com massas abdominais ou sopro abdominal (GR: IIa, NE: B). ^267^
Hemoglobina glicada (HbA1c): indicada quando a glicemia de jejum for maior que 99 mg/dL, na presença de história familiar ou de diagnóstico prévio de DM2 e obesidade (GR: IIa, NE: B). ^268^
Teste ergométrico: está indicado na suspeita de doença coronária estável, diabetes melito ou antecedente familiar para doença coronária em pacientes com pressão arterial controlada (GR: IIa, NE: C). ^269^
MAPA/MRPA: ver as indicações dos métodos no Capítulo 3 (GR: I, NE: A). ^186^
Medida da velocidade da onda de pulso (VOP), quando disponível: indicada em hipertensos de baixo e médio risco, sendo considerado um método útil para avaliação da rigidez arterial, ou seja, do dano vascular. VOP com valores acima de 10m/s são considerados anormais na população em geral, porém já existem valores de referência ajustados para decis de idade e sexo (GR: IIa, NE: A). ^139^ ^,^ ^270^ ^,^ ^271^
Ressonância nuclear magnética (RNM) do cérebro: indicada em pacientes com distúrbios cognitivos e demência para detectar infartos silenciosos e micro-hemorragias (GR: IIa, NE: C). ^272^

Para a avaliação da função renal, devemos obter o ritmo de filtração glomerular estimado calculado pelas fórmulas do *Modification of Diet in Renal Diseases* (MDRD) ^255^ ou, preferencialmente, pelo *Chronic Kidney Diseases Epidemiology Collaboration* (CKD-EPI), ^256^ que podem ser obtidas no *site* http://mdrd.com/.

**Figura 4.1:**
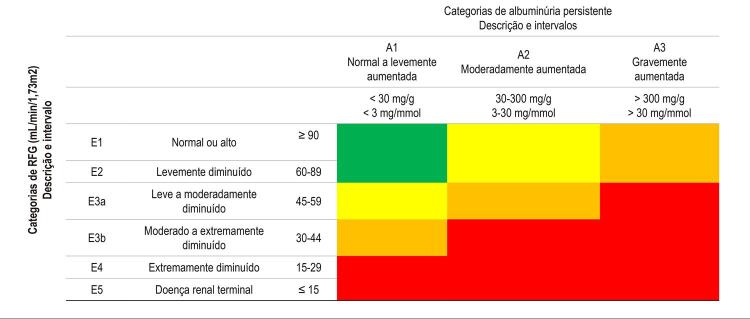
– Prognóstico da doença renal crônica de acordo com o ritmo de filtração glomerular e a albuminúria.

DRC: Doença renal crônica, RFG-e: ritmo de filtração glomerular estimado; KDIGO: Kidney Diseases Improving Global Outcomes.

Na Figura 4.1 , temos ritmo de filtração glomerular estimado (RFG-e) com a interpretação dos valores para a classificação em estágios (E1 a E5) e o prognóstico da doença renal crônica, levando-se em conta a categoria de albuminúria, de acordo com o *Kidney Diseases Improving Global Outcomes* (KDIGO). ^257^ ^,^ ^258^ As cores expressam o prognóstico renal e a conduta. O verde indica bom prognóstico e baixo risco; o amarelo, risco intermediário, devendo-se monitorizar o paciente; o laranja, alto risco, mau prognóstico, com obrigatoriedade de referenciar para o especialista; e o vermelho, risco muito alto, mau prognóstico e obrigatoriedade de referenciar para o especialista.

Com relação à avaliação renal:

• Recomenda-se que o laboratório de análises clínicas disponibilize o resultado do exame de dosagem de creatinina acompanhado do resultado do RFG-e (GR: I, NE: B);
• Não recomendamos a dosagem de *clearance* de creatinina (urina de 24h), exceto para alteração significativa da massa muscular (amputação), superfície corporal de extremos e instabilidade clínica (GR: I, NE: B);
• Recomenda-se que se investigue a proteinúria/albuminúria utilizando-se pela ordem de importância: razão albuminúria/creatininúria (RAC), razão proteinúria/creatininúria (RPC); urinálise por fita de proteinúria total com leitura automática e urinálise por fita de proteinúria total com leitura manual. Recomenda-se que os laboratórios clínicos relatem RAC e RPC de qualquer amostra de urina e não apenas suas concentrações (GR: I, NE: B).

**Table:** 

Mensagens principais
A anamnese e o exame físico devem ser completos buscando sempre a medida correta da PA, a análise dos parâmetros antropométricos e a detecção de sintomas e sinais de comprometimento em órgãos-alvo e de indícios de causas secundárias de hipertensão.
No paciente hipertenso, é importante a pesquisa de comorbidades (diabetes melito, dislipidemias e doenças renais e da tireoide, entre outras), para melhor tratamento e estratificação do risco CV.
Os exames complementares de rotina preconizados nessas diretrizes são básicos, de fácil disponibilidade e interpretação, baixo custo e obrigatórios para todos os pacientes, pelo menos na primeira consulta e anualmente. Outros exames podem ser necessários para as populações indicadas.
É fundamental pesquisar lesões em órgãos-alvo, tanto clínicas quanto subclínicas, para orientação terapêutica mais completa.

## 5. Estratificação de Risco Cardiovascular

## 5.1. Introdução

Está amplamente estabelecida a relação causal, linear e contínua entre o aumento da pressão arterial (PA) e o risco de doença cardiovascular (DCV) em ambos os sexos, todas as idades e todos os grupos étnicos. ^85^ A PA atua de forma sinérgica com outros fatores de risco (FR) para DCV, e seu efeito pró-aterogênico será tanto maior quanto maior o for o número e a intensidade desses fatores adicionais. ^78^ ^,^ ^273^ A DCV é uma condição multifatorial que depende de interações sinérgicas em todo o complexo causal responsável por seu desenvolvimento. Além disso, o aumento modesto de vários FR pode desencadear maior incremento no risco cardiovascular (CV) que a elevação acentuada de apenas um único FR. ^273^ ^,^ ^274^ Assim, quantificar o risco do paciente hipertenso, ou seja, a probabilidade de determinado indivíduo desenvolver DCV em um determinado período de tempo é parte essencial do processo e pode nortear estratégias preventivas e de tratamento. ^37^ ^,^ ^164^ ^,^ ^275^

Vale destacar que o impacto do controle da hipertensão arterial (HA) será tanto maior quanto maior for o risco absoluto individual e o risco global estimado. ^276^ ^-^ ^278^ Digno de nota é o conceito de risco residual que representa a magnitude do risco que permanece depois que os FR tradicionais são controlados. ^279^ Admite-se que o controle parcial dos FR e/ou a instituição tardia de medidas terapêuticas eficazes sejam elementos centrais na geração do risco CV residual no paciente hipertenso. Apesar da escassez de ferramentas para identificar o risco residual, está claro que a precocidade e a precisão no controle dos FR são ferramentas essenciais no controle dele. ^279^ Dessa maneira, a abordagem abrangente de todos os FR justifica-se plenamente. Nesse sentido, quando identificados, os FR potencialmente modificáveis devem ser informados aos pacientes hipertensos com o objetivo de melhorar a eficácia das medidas farmacológicas e não farmacológicas implementadas. ^37^ ^,^ ^164^ ^,^ ^275^

A estimativa de risco CV não deve ser obtida de modo intuitivo ou pela mera soma dos FR presentes, mas por métodos que considerem sua natureza complexa e multifatorial. ^4^ Deve ser feita por meio de equações ou algoritmos, ^4^ ^,^ ^280^ ^,^ ^281^ instrumentos que estimam o risco baseados em modelos de regressão com múltiplas variáveis e desenvolvidos a partir de estudos populacionais, recomendados em várias diretrizes em todo o mundo. ^4^ ^,^ ^280^ ^,^ ^281^ Mesmo médicos experientes erram em mais de 50% dos casos quando estimam o risco sem a utilização de uma equação ou um algoritmo. ^282^

Deve ser ressaltada, entretanto, a ausência de dados populacionais Brazileiros nesses modelos de estimativa de risco, o que os torna menos precisos na avaliação do risco CV em nossa população. Em outras palavras, o risco aferido pelos escores internacionais pode estar subestimado por deixar de considerar os FR mais prevalentes ou relevantes em nossa população. Para atenuar essa limitação e evitar subdiagnosticar pacientes em alto risco, a presente diretriz recomenda identificar outros marcadores, denominados fatores modificadores de risco, para reclassificar o risco em indivíduos considerados de risco moderado (a ser detalhado adiante).

A classificação do risco CV depende dos níveis da PA, dos FRCV associados, da presença de lesões em órgãos-alvo (LOA), que são lesões estruturais e/ou funcionais decorrentes da HA em vasos, coração, cérebro, rins e retina, e/ou da existência de DCV ou doença renal estabelecidas. ^37^ ^,^ ^164^ ^,^ ^275^ Diferentes escores foram desenvolvidos e vêm sendo aplicados para classificar os pacientes hipertensos em categorias de baixo, moderado e alto riscos CV. ^37^ ^,^ ^164^ ^,^ ^275^ Entretanto, novos biomarcadores, precursores e preditores de doença parecem ainda ser necessários para melhorar a predição de risco e reduzir a diferença entre o risco calculado e as taxas de eventos observadas, sobretudo nos indivíduos estratificados como de risco moderado. ^283^ ^-^ ^285^ A incorporação de ferramentas para a avaliação do risco relativo *versus* risco vitalício e períodos de avanço do risco ao longo da vida também se justifica, especialmente na avaliação de jovens com baixo risco absoluto, mas com alto risco relativo para DCV bem como no idoso, cuja estimativa de risco continua sendo um desafio pela acelerada modificação da expectativa de vida e pela funcionalidade nestes últimos anos. ^285^

## 5.2. Estratificação de Risco Adicional (Condições Associadas)

Na maioria dos pacientes hipertensos, coexistem ou agregam-se outros fatores reconhecidos como capazes, *per se,* determinar ou incrementar o aparecimento e desenvolvimento da DCV, independentemente dos valores da PA. ^37^ ^,^ ^275^ ^,^ ^286^ ^-^ ^288^ As condições associadas, quer por sua prevalência na população em geral, quer pela força de sua associação a eventos CV, devem ser identificadas no paciente hipertenso, por meio de história clínica, exame físico e exames complementares. ^37^ ^,^ ^164^ ^,^ ^275^ Assim, devem ser procurados: a) os FR coexistentes na HA ( Quadro 5.1 ); b) as LOA ( Quadro 5.2 ); c) a presença de DCV e renal estabelecida.

**Quadro 5.1:** – Fatores de risco coexistentes na hipertensão arterial

Sexo masculino
Idade: > 55 anos no homem e > 65 anos na mulher
DCV prematura em parentes de 1 ^o^ grau (homens < 55 anos e mulheres < 65 anos)
Tabagismo
Dislipidemia: LDL-colesterol ≥100mg/dL e/ou não HDL-colesterol 130 mg/dL e/ou HDL-colesterol ≤ 40mg/dL no homem e ≤ 46mg/dL na mulher e/ou TG >150 mg/dL
Diabetes melito
Obesidade (IMC ≥ 30 kg/m ^2^ )

A identificação de tais condições é importante não apenas para estimar o risco a que se expõe o paciente hipertenso em seu respectivo estágio de hipertensão, bem como para que os FR controláveis sejam alvos de intervenção terapêutica própria, com a intensidade que a gradação de risco lhe demande. ^37^ ^,^ ^164^ ^,^ ^275^ Devem ser priorizados os FR de mais fácil identificação e os mais prevalentes, bem como aqueles cuja associação ao risco CV esteja solidamente estabelecida ( Quadro 5.1 ).

Conforme exposto no Quadro 5.1 , os fatores relacionados neste devem ser considerados na estimativa do risco CV, respeitando-se os critérios diagnósticos vigentes. A idade apresenta correlação linear com HA e risco de complicações CV, como infarto do miocárdio (IAM) e acidente vascular encefálico (AVE), sendo tal linearidade mais evidente com o AVE. Para o IAM, há uma acentuação do grau de associação dependente do sexo que se inicia aos 55 anos no homem e aos 65 anos na mulher. ^287^ Tabagismo, quantificado como carga maço/ano, tabagismo passivo e outras formas de tabagismo, como charutos, cachimbos e cigarros eletrônicos, são considerados elementos centrais no risco CV. Já a dislipidemia caracteriza-se por elevações do conteúdo de colesterol da lipoproteína de baixa densidade (LDL) ou do conjunto de lipoproteínas aterogênicas obtido pela subtração da liproproteina de alta densidade (HDL) do colesterol total, ou seja, não HDL-colesterol. A consideração do valor de HDL-colesterol permanece indicada como estimativa de risco e possui limiares distintos em homens e mulheres. Os triglicerídeos (TG) elevados também caracterizam dislipidemia, particularmente quando associados a redução da HDL-colesterol ou em níveis superiores a 500 mg/dL, quando o tratamento específico está indicado e há possibilidade de pancreatite. O diabetes melito deve ser diagnosticado pelos seguintes critérios: glicemia plasmática em jejum de > 126 mg/dL; hemoglobina glicada > 6,5%, aferida por cromatografia líquida de alta performance (HPLC); ou ainda, glicemia > 200mg/dL, após 2 h de sobrecarga oral de glicose no teste oral de tolerância ou em glicemia aleatória. A obesidade deve ser considerada quando o índice de massa corporal (IMC) for > 30kg/m ^2^ ou quando a medida da cintura abdominal (CA) for > 80 cm em mulheres ou > 94 cm em homens de descendência europeia ou africana ou > 90 cm naqueles de descendência asiática. ^289^

### 5.2.1. Lesões em Órgãos-Alvo

A estimativa de risco no paciente hipertenso deve ser complementada pela identificação da presença de LOA, que são frequentes, muitas vezes subdiagnosticadas, não estando geralmente incluídas nos escores de estratificação de risco. Elas causam aumento adicional do risco CV, notadamente quando várias coexistem no mesmo indivíduo ^5^ ^,^ ^6^ ^,^ ^7^ ( Quadro 5.2 ).

**Quadro 5.2:** – Lesões em órgãos-alvo

Hipertrofia ventricular esquerda
ECG (índice Sokolow-Lyon (SV1 + RV5 ou RV6) ≥ 35 mm; RaVL > 11 mm; Cornell voltagem > 2440 mm.ms ou Cornell índice > 28 mm em homens e > 20 mm em mulheres (GR: I, NE: B)
ECO: IMVE ≥ 116 g/m ^2^ nos homens ou ≥ 96 g/m ^2^ nas mulheres (GR: IIa, NE: B)
ITB < 0,9 GR (GR: IIa, NE: B)
Doença renal crônica estágio 3 (RFG-e entre 30 e 60 mL/min/1,73m ^2^ )
Albuminúria entre 30 e 300 mg/24h ou relação albumina/creatinina urinária 30 a 300 mg/g (GR: I, NE: B)
VOP carótido-femoral > 10 m/s (GR: IIa, NE: A)

ECG: eletrocardiograma; ECO: ecocardiograma; IMVE: índice de massa ventricular esquerda; ITB: índice tornozelo-braquial; RFG-e: ritmo de filtração glomerular estimado; VOP: velocidade de onda de pulso.

### 5.2.2. Presença de Doença Cardiovascular e Renal

A presença de doenças cardiovascular e renal documentadas determina o risco aumentado de eventos CV no hipertenso. ^37^ ^,^ ^164^ ^,^ ^275^ ^,^ ^290^ ^-^ ^292^ A doença cerebrovascular deve ser considerada em quaisquer das seguintes manifestações: AVE isquêmico, hemorragia encefálico ou acidente isquêmico transitório. A doença arterial coronariana deve englobar angina, IAM, isquemia miocárdica silenciosa, cirurgia de revascularização miocárdica ou intervenção coronariana prévias. A insuficiência cardíaca (IC) com fração de ejeção preservada (FEp) ou a reduzida (FEr) devem ser igualmente consideradas como doença cardiovascular manifesta, ^86^ ^,^ ^275^ bem como a fibrilação atrial (FA). ^293^ Da mesma maneira, a doença arterial obstrutiva periférica (DAOP) sintomática ^294^ e a doença de aorta relacionada com aneurismas, hematomas ou ulcerações constituem manifestações cardiovasculares com grande impacto no risco CV global. Pela estreita associação ao risco CV, devem ser considerados como indicadores de risco alto a doença renal crônica estágio 4 ou superior, identificada como ritmo de filtração glomerular estimado (RFG-e) < 30 mL/min/1,73m ^2^ , relação albuminúria/creatininúria em amostra isolada (> 300 mg/g creatinina); e proteinúria/creatininúria em amostra isolada (> 300 mg/g creatinina). Por fim, retinopatia atribuível ao processo hipertensivo, como hemorragias; exsudatos; e papiledema também são condições indicativas de alto risco. ^37^ ^,^ ^275^

No Qaudro 5.3 , estão expostas as principais razões para que se deva realizar a estimativa de risco levando-se em conta, além do valor pressórico, a presença de fatores coexistentes de risco cardiovascular, LOA e de DCV e/ou renal. Incluem estabelecimento de prognóstico com razoável precisão de estimativa e distinção dos casos que exigem intensificação da terapêutica. ^37^ ^,^ ^275^

**Quadro 5.3:** – Razões para realizar a estimativa de avaliação de risco (GR: I NE: C)

Estimar o risco de eventos cardiovasculares em médio e longo prazos
Determinar o nível de atenção à saúde, como frequência dos atendimentos
Determinar a precocidade de início do tratamento farmacológico
Determinar a intensidade do controle dos fatores de risco modificáveis

O Quadro 5.4 é de grande valia na estratificação de risco do paciente hipertenso. Vale lembrar que os FR mencionados se referem àqueles com valor epidemiológico estabelecido, facilidade de obtenção na maioria das situações clínicas e comprovado valor prognóstico. Auxilia na compreensão do impacto da progressão de risco associado aos diferentes graus de PA, presença de FR, LOA ou doença cardiovascular e/ou renal, em indivíduos de meia-idade.

Nos pacientes considerados de risco moderado, tem sido recomendada a realização de testes, quando disponíveis e factíveis, e nunca de forma generalizada, para a identificação de marcadores subclínicos de LOA com o objetivo de incrementar a acurácia do risco estimado. ^82^ ^,^ ^295^ ^,^ ^296^ Destacam-se a avaliação da HVE através da ecocardiografia, que também registra parâmetros de função ventricular, e o índice de massa ventricular esquerda (IMVE) (GR: IIa, NE: B), a albuminúria, preferencialmente pela relação albuminúria/creatininúria e a realização do ITB (GR: IIa, NE: B). Biomarcadores vasculares de incorporação clínica mais recente, como a velocidade de onda de pulso carotídeo-femoral (VOPcf), também podem contribuir para reclassificar o risco CV desses indivíduos (GR: IIa, NE: A), ^296^ ^,^ ^297^ além de conferir pior prognóstico naqueles com doença já estabelecida. ^139^ ^,^ ^298^ Tal aspecto é de particular importância em indivíduos jovens, que têm menor risco relativo e nos quais há oportunidade de prevenção da deterioração irreversível da estrutura e da função da parede arterial.

**Quadro 5.4:** – Classificação dos estágios de hipertensão arterial de acordo com o nível de PA, presença de FRCV, LOA ou comorbidades

FR, presença de LOA ou doença	PA (mmHg)			
	Pré-hipertensão PAS 130-139 PAD 85-89	Estágio 1 PAS 140-159 PAD 90-99	Estágio 2 PAS 160-179 PAD 100-109	Estágio 3 PAS > 180 PAD > 110
Sem FR	Sem risco adicional	Risco baixo	Risco moderado	Risco alto
1 ou 2 FR	Risco baixo	Risco moderado	Risco alto	Risco alto
≥ 3 FR	Risco moderado	Risco alto	Risco alto	Risco alto
LOA, DRC estágio 3, DM, DCV	Risco alto	Risco alto	Risco alto	Risco alto

PA: pressão arterial; FR: fator de risco; PAS: pressão arterial sistólica; PAD: pressão arterial diastólica; LOA: lesão em órgão-alvo, DRC: doença renal crônica; DM: diabetes melito; DCV: doença cardiovascular.

Outras condições também têm sido reconhecidas como capazes de influenciar ou modificar o risco CV do paciente hipertenso classificado como de risco moderado, porém com menor poder discriminatório. ^37^ ^,^ ^275^ Sua identificação não é recomendada para o reconhecimento de pacientes de alto risco ou naqueles sem nenhum dos fatores coexistentes de risco acima elencados ( Quadro 5.5 ).

**Quadro 5.5:** – Fatores que podem modificar o risco do paciente hipertenso

História familiar ou nos pais de início precoce de hipertensão arterial
Nível muito elevado de um FR individual, incluindo HA estágio 3
Eclâmpsia/pré-eclâmpsia prévia
Apneia do sono
Pressão de pulso > 60 (em pacientes idosos)
Ácido úrico > 7 mg/dL (homens) e > 5,7 mg/dL (mulheres) (GR: I, nível: C)
Proteína C-reativa ultrassensível > 2mg/L (GR: I, nível: B)
FC > 80 bpm
Síndrome metabólica*
Sedentarismo
Fatores psicossociais e econômicos
Distúrbios inflamatórios crônicos

FR: fatores de risco: HA: hipertensão arterial; FC: frequência cardíaca. *Para que se defina a presença de síndrome metabólica pelos critérios da International Diabetes Federation (IDF) é necessário haver obesidade central, definida como circunferência abdominal > 80 cm em mulheres ou > 94 cm em homens de descendência europeia ou africana ou > 90 cm naqueles de descendência asiática, além de dois entre os quatro fatores a seguir: triglicerídeos > 150 mg/dL, HDL-C baixo (< 40 mg/dL em homens e < 50 mg/dL em mulheres), hipertensão arterial; glicemia de jejum ≥ 100 mg/dL ou DM tipo 2 previamente diagnosticado. ^289^

## 5.3. Avaliação do Risco Cardiovascular Global

A estratificação de risco CV global não é específica para o paciente hipertenso e tem como objetivo determinar o risco global de um indivíduo entre 30 e 74 anos de desenvolver DCV em geral nos próximos 10 anos. ^3^ ^,^ ^4^ ^,^ ^280^ ^,^ ^281^ ^,^ ^299^ Vale ressaltar que a HA é um dos FRCV com maior impacto relativo – portanto, presente em todas as equações de estimativa de risco global. Além disso, a mitigação do risco CV não será efetiva sem que se considere o conjunto de elementos que determinam o curso clínico do paciente hipertenso. Uma forma prática e ágil de calcular o risco CV global é utilizar a Calculadora para Estratificação de Risco Cardiovascular recomendada e disponibilizada pelo Departamento de Aterosclerose da SBC em seu *site* e disponível para os sistemas Android e IOS. ^3^

## 5.4. Desafios na Avaliação do Risco Cardiovascular na Hipertensão Arterial

Várias condições clínicas podem interferir na estratificação do risco CV do paciente hipertenso e, entre essas, destaca-se a idade. Em curto prazo, enquanto os pacientes idosos têm maior risco absoluto, os jovens apresentam menor risco absoluto, mesmo com perfil de risco desfavorável. ^37^ Em longo prazo ou no risco ao longo da vida, a influência da idade sobre o risco CV inverte-se, passando a ter maior perda de longevidade aqueles que manifestam os FR em idade jovem.

Outra limitação é a duração da exposição à doença ou ao FR. Escores que utilizam a opção binária (sim/não) para condições clínicas como DM e tabagismo na avaliação do risco cardiovascular não consideram a duração dessas condições. Portanto, pacientes com maior tempo de exposição terão maior risco CV quando comparados com indivíduos com menor tempo de exposição aos mesmos fatores. ^37^ Por isso, as incorporações de novas ferramentas para a avaliação do risco relativo *versus* risco vitalício e períodos de avanço do risco parecem ser necessárias, especialmente nos jovens com baixo risco absoluto, mas com alto risco relativo para DCV. ^280^ ^,^ ^299^ O conceito de “idade vascular” e “idade cardiometabólica” podem auxiliar nessa estratégia. ^300^ ^,^ ^301^

A influência da duração do tratamento anti-hipertensivo também pode interferir na estimativa de risco. Nos pacientes hipertensos com início recente de tratamento, deve ser considerada a pressão arterial antes do início do tratamento, enquanto nos pacientes com maior tempo de tratamento deverão ser consideradas as medidas atuais de pressão arterial. ^37^

O momento de aferição e o valor de PA a ser considerado na estratificação de risco CV e os diferentes fenótipos de HA também devem ser levados em consideração. Nesse sentido, a medida da PA fora do consultório tem sido cada vez mais estimulada. Medidas domiciliares, monitorização ambulatorial da pressão arterial (MAPA) e monitorização residencial da pressão arterial (MRPA) têm trazido importantes informações complementares, embora ainda se considere a medida de PA de consultório como referência para o diagnóstico e a avaliação de eficácia do tratamento. ^37^ ^,^ ^164^ ^,^ ^302^ Nesse sentido, a recomendação da realização da MAPA vem sendo ampliada para a confirmação do diagnóstico de HA, com maior correlação de suas medidas com LOA e morbidade e mortalidade cardiovascular quando comparadas com as medidas casuais de PA. ^303^ Nessa mesma direção, diferentes padrões de HA, como a hipertensão mascarada, a hipertensão sistólica isolada e a ausência de descenso noturno ou mesmo aumento da PA durante o sono, também parecem conferir risco cardiovascular distinto. ^304^ ^,^ ^305^ Desse modo, as limitações apontadas devem ser levadas em consideração na individualização da estimativa de risco CV do paciente hipertenso na prática clínica.

**Table:** 

Mensagens principais
Mais de 50% dos pacientes hipertensos têm FRCV adicionais.
A presença de um ou mais FRCV adicionais aumenta o risco de doença coronariana, cerebrovascular, renal e arterial periférica nos pacientes hipertensos.
A identificação dos FR adicionais deve fazer parte da avaliação diagnóstica do paciente hipertenso, especialmente quando há história familiar de DCV.
O risco CV deve ser estimado em todos os pacientes hipertensos por meio de escores simples, baseados nos níveis de PA e na presença de FR adicionais e comorbidades ( Quadro 5.1 ).
Uma estimativa confiável do risco CV pode ser feita de forma prática por meio da identificação de FR, como idade > 65 anos, sexo (homens > mulheres), frequência cardíaca (> 80 bpm), aumento do peso corporal, diabetes melito, elevação do LDL-c, história familiar de DCV, história familiar de HAS, tabagismo e fatores psicossociais e/ou socioeconômicos; de LOA: presença de HVE, DRC moderada a grave (RFG-e < 60 mL/min/1,73m2) ou de outra avaliação que confirme presença de LOA e de doenças prévias: DAC, IC, AVE, DAOP, FA e DRC estágio 3 ou maior.
CV: cardiovascular; ERG: escore de risco global; DCV: doença cardiovascular; DRC: doença renal crônica; FR: fator de risco; LOA: lesão de órgão-alvo; HVE: hipertrofia ventricular esquerda; RFG-e: ritmo de filtração glomerular estimado; IC: insuficiência cardíaca; AVE: acidente vascular encefálico; DAOP: doença arterial obstrutiva periférica; FA: fibrilação atrial.

## 6. Decisão e Metas Terapêuticas

## 6.1. Introdução

Um dos objetivos específicos do tratamento do paciente hipertenso é obter o controle pressórico alcançando a meta de pressão arterial (PA) previamente estabelecida. Tal meta deve ser definida individualmente, sempre considerando a idade e a presença de doença cardiovascular (DCV) ou de seus fatores de risco (FR). De forma geral, deve-se reduzir a PA visando a alcançar valores menores que 140/90 mmHg e não inferiores a 120/70 mmHg (GR: I, NE: A). Nos indivíduos mais jovens e sem FR, podem-se alcançar metas mais baixas com valores inferiores a 130/80 mmHg.

## 6.2. O Hipertenso de Risco Baixo ou Moderado

A estimativa de risco cardiovascular (CV) é extremamente importante no paciente hipertenso, pois define possíveis diferenças na meta de PA a ser alcançada. Os pacientes hipertensos sem muitos FR adicionais devem ser avaliados sob duas óticas distintas: hipertensos com níveis pressóricos significativamente elevados sem outros FR (hipertensão estágio 2 – risco moderado) e aqueles com elevações menores de PA (hipertensão estágio 1 – baixo risco).

Os benefícios de se tratar os hipertensos sem outros fatores de risco CV associados que apresentam valores de PA significativamente elevados (> 160 mmHg) já estão bem estabelecidos e vêm sendo sistematicamente recomendados por diretrizes nacionais e internacionais. ^5^ ^,^ ^37^ ^,^ ^164^ Por outro lado, há carência de evidências científicas derivadas de estudos randomizados que justifiquem o tratamento do paciente hipertenso estágio 1 com baixo risco CV. Tal fato ocorre porque o grande número de participantes e o tempo prolongado de seguimento necessários praticamente inviabilizam um estudo randomizado controlado conduzido em pacientes com essas características. Assim, metanálises de dados individuais de participantes de estudos randomizados conduzidos com hipertensos estágio 1 sem DCV prévia nos auxiliam a definir a melhor conduta. ^306^ ^-^ ^308^ Um desses estudos evidenciou que o tratamento do hipertenso de baixo risco não reduziu os desfechos de doença arterial coronariana (DAC), os eventos CV ou a mortalidade CV, em um seguimento de quatro a cinco anos. ^306^ Houve, entretanto, uma tendência para a redução de acidente vascular encefálico (AVE) e mortalidade total, sendo que tais diminuições seriam claramente obtidas com um tempo de seguimento maior ou mais participantes incluídos nos estudos. Uma segunda metanálise, que incluiu cerca de 9.000 participantes de cinco estudos randomizados, evidenciou a redução do desfecho composto (DAC e AVE) em 34% e na mortalidade por todas as causas em 19% pela redução da pressão arterial sistólica (PAS) em 7 mmHg com tratamento medicamentoso. ^307^ Uma terceira publicação demonstrou que a redução de doenças CV e de mortalidade quando a PA inicial era maior ou igual a 140/90 mmHg, sendo que o mesmo resultado não foi observado com valores iniciais menores. ^308^ Todos esses achados foram reforçados pelos resultados de uma análise de subgrupo do estudo *Heart Outcomes Prevention Evaluation* (HOPE)-3. Neste estudo, mesmo que os hipertensos estágio 1 fossem classificados como de risco CV intermediário, o tratamento anti-hipertensivo com redução média de 6 mmHg na PAS, reduziu em 27% eventos CV maiores. ^309^ Com base em tais dados, o tratamento medicamentoso pode ser iniciado em combinação com o tratamento não medicamentoso no hipertenso estágio 1 de baixo risco cardiovascular (GR: I, NE: A).

Com relação à meta pressórica no hipertenso de baixo risco CV, também faltam dados específicos provenientes de estudos randomizados. Uma metanálise recente e dados de um grande estudo observacional sugerem que metas pressóricas inferiores a 140/90 mmHg devem ser obtidas nesses pacientes, sendo que as maiores reduções em desfechos CV são obtidas com valores de PAS entre 120-130mmHg. ^85^ ^,^ ^310^ Assim, para tais pacientes recomendamos uma meta abaixo de 140/90 mmHg e, se tolerada, próximo a 120/80 mmHg ( Quadro 6.1 ) (GR: I, NE: B).

**Quadro 6.1:** – Metas pressóricas gerais a serem obtidas com o tratamento anti-hipertensivo

Meta	Risco cardiovascular	
	Baixo ou moderado	Alto
PA sistólica (mmHg)	< 140	120-129
PA diastólica (mmHg)	< 90	70-79

## 6.3. O Hipertenso de Alto Risco

Em geral, o hipertenso com três ou mais FR, diabético, com lesões em órgãos-alvo (LOA), doença CV ou renal é considerado de alto risco. Na prática clínica, os exemplos mais comuns de indivíduos com alto risco CV são os pacientes hipertensos com DAC, história prévia de AVE, com insuficiência cardíaca e doença renal crônica (DRC), e quando a HA está associada ao diabetes melito (DM). Essas comorbidades são abordadas no Capítulo 10 ( *Condições Clínicas Associadas* ), mas as metas para cada uma dessas situações clínicas estão discutidas adiante. Convém lembrar que o alto risco depende não apenas da presença de FR e de LOA, mas também do estágio da HA, conforme o Quadro 5.4 , podendo um hipertenso ter FR ou LOA, mas em estágio 3 de HA ( Quadro 6.1 ).

## 6.4. O Hipertenso com Doença Coronária

A HA consiste em um importante FR independente para o desenvolvimento de isquemia miocárdica. Antes dos 50 anos, a PA diastólica (PAD) é o principal preditor de risco de DAC, enquanto após os 60 anos de idade a PAS se mostra mais importante. ^311^ Na população mais idosa, a PAD torna-se inversamente relacionada com o risco de DAC, e a pressão de pulso torna-se o preditor mais forte para DAC. ^311^ Em uma metanálise que incluiu quase 1 milhão de adultos, a DAC fatal foi correlacionada com os níveis de PA a partir de 115/75 mmHg para todas as idades. ^78^ Nesse caso, o tratamento anti-hipertensivo em pacientes com DAC deve procurar resultar em PA < 130/80 mmHg, mas não inferior a 120/70 mmHg. Nos pacientes com evidência de isquemia miocárdica, a PAD deve ser reduzida com cautela até 70 mmHg, especialmente nos diabéticos e nos mais idosos. ^312^ Convém muito cuidado na redução da PAS nos idosos com DAC e com elevada pressão de pulso, pois isso pode resultar em valores muito baixos de PAD e propiciar isquemia miocárdica. ^313^

## 6.5. O Hipertenso com História de Acidente Vascular Encefálico

A HA é o FR mais importante para AVE isquêmico (AVEI) e hemorrágico (AVEH), demonstrando uma relação direta com os níveis pressóricos. Nos indivíduos mais jovens sem doença CV ou renal estabelecida, manter a PA na faixa ótima ou normal, com uma meta de 120/80 mmHg, pode ser mais eficaz para a prevenção cerebrovascular primária. Para aqueles com um ou mais episódios prévios de AVE, a meta mais adequada para a prevenção secundária deve ser avaliada conforme o tipo de AVE e o tempo pós-evento (Tabela 10.2). Nos casos crônicos de prevenção secundária, o preconizado é manter a PAS entre 120 e 130 mmHg (GR: I, NE: A). ^314^ Nos mais idosos ou com doença coronária associada, situação relativamente comum, o fenômeno da curva J deve ser considerado quando a PA alcança valores abaixo de 120/70 mmHg, com maior risco para eventos CV e mortalidade. ^315^

## 6.6. O Hipertenso com Insuficiência Cardíaca

A HA é considerada FR para as duas formas de apresentação de insuficiência cardíaca (IC), ou seja, com fração de ejeção reduzida e com fração de ejeção preservada. O tratamento adequado da HA reduz a incidência de IC. Ainda não houve estudos clínicos específicos na população com IC comparando diferentes metas de tratamento. As recomendações são, então, extrapoladas das evidências em outras populações de alto risco, nas quais a redução da PA mostrou maior proteção contra eventos CV, embora com potencial aumento dos efeitos colaterais. Nos pacientes que já apresentam IC com fração de ejeção reduzida, o controle pressórico diminui a mortalidade e a taxa de reinternação por descompensação cardíaca. A meta adequada nessa população é < 130/80 mmHg, mas com o cuidado de manter acima de 120/70 mmHg. ^316^ Naqueles com fração de ejeção preservada, a melhor forma de tratamento permanece incerta e, portanto, recomenda-se adotar uma estratégia de tratamento semelhante aos pacientes com fração de ejeção reduzida. ^317^ ^,^ ^318^

## 6.7. Hipertenso com Doença Renal Crônica (DRC)

A PA elevada está presente na maioria dos pacientes com DRC, o que aumenta o risco de doenças CV, DRC e morte. O estudo *Systolic Blood Pressure Intervention Trial* (SPRINT) concluiu que a PA sistólica < 120 mmHg diminuiu o risco de doença CV e a mortalidade em adultos não diabéticos com alto risco CV, muitos dos quais com DRC. Entretanto, não foi capaz de retardar a progressão da DRC. ^319^ É notório que, nesse estudo, a PA foi aferida com dispositivos automatizados e frequentemente desacompanhada, uma técnica que geralmente resulta em valores inferiores aos medidos no consultório. ^320^ ^,^ ^321^ A redução absoluta do risco pode ser maior entre aqueles com albuminúria, devido à forte associação entre albuminúria e doença CV e renal, mas os efeitos da redução intensiva da PA sobre o risco de DCV parecem ser similares pelo nível de albuminúria. ^322^ ^,^ ^323^ As evidências atuais indicam uma meta de PA < 130/80 mmHg em pacientes com DRC, independentemente da presença de DM. ^275^ ^,^ ^324^ ^,^ ^325^ Nos pacientes com DRC terminal, os benefícios do controle intensivo da PA são incertos, pois os estudos são de curta duração, e os efeitos hemodinâmicos podem levar à maior redução do ritmo de filtração glomerular (RFG). Independentemente da meta a ser alcançada, a redução da PA no paciente com DRC sempre exige atenção na medida correta da PA e monitorização dos eventos adversos, sobretudo das anormalidades eletrolíticas e da diminuição do RFG. ^325^

## 6.8. O Hipertenso Diabético

No paciente hipertenso DM, obtém-se a prevenção da morbidade e da mortalidade com o controle da glicemia, a normalização da PA e a redução dos outros fatores de risco CV. ^326^ Manter a PA controlada no indivíduo diabético é essencial para a proteção renal, o que reduz a albuminúria, além de ser importante para diminuir o risco de AVE e de hipertrofia ventricular esquerda (HVE). ^327^ ^,^ ^328^ Evidências de ensaios clínicos randomizados, metanálises e estudos observacionais em diabéticos hipertensos indicam que uma redução da PAS para 130-139 mmHg, com valores próximos a 130 mmHg, protege eficazmente contra complicações CV e renais. ^307^ ^,^ ^329^ A PAD pode ser reduzida para 70-79 mmHg sem comprometer a proteção e a segurança do indivíduo. Por outro lado, não há dados conclusivos que diminuir a PAS para < 130 mmHg leva a uma maior proteção CV e renal. Valores de PAS < 120 mmHg devem ser evitados. Portanto, a meta recomendada em diabéticos é < 130/80 mmHg (GR: IIa, NE: B).

Alcançar uma meta de PAS mais baixa implica necessidade de maior número de anti-hipertensivos, o que eleva o risco de efeitos adversos graves. ^330^ Na prática, as metas ideais de PA podem variar entre os hipertensos diabéticos, de acordo com a idade e a presença de LOA. Por exemplo, não há dados disponíveis em pacientes com diabetes de início recente, sem complicações e, portanto, com riscos CV e renal relativamente baixos. Nesses casos, níveis pressóricos mais baixos podem ser mais bem tolerados e resultar em maior benefício em médio e longo prazos. De forma geral, o controle da PA é mais difícil nos indivíduos diabéticos do que naqueles não diabéticos. Além disso, não é raro o hipertenso diabético demonstrar níveis satisfatórios de PA na consulta e valores elevados na MAPA ou na MRPA, caracterizando, assim, a hipertensão mascarada. Tal observação reforça a necessidade de medidas da PA fora do consultório para uma melhor avaliação de controle no hipertenso diabético. ^331^

## 6.9. O Hipertenso Idoso

A complexidade do hipertenso idoso é abordada no Capítulo 14. As metas de PA para a população idosa devem levar em conta, além da idade cronológica (considerando ≥ 60 anos nos países de baixa renda e ≥ 65 anos nos demais, de acordo com a maioria das sociedades internacionais), ^37^ ^,^ ^275^ o estado funcional, a fragilidade e as comorbidades presentes. Dessa forma, o alvo terapêutico deve ter equilíbrio entre os potenciais benefícios e prejuízos com os valores de PA atingidos.

Na maioria dos ensaios clínicos que mostraram benefício do tratamento da PA em pacientes idosos, o alvo para a PAS variou entre 140 e 150 mmHg, em que se observou a maior redução de eventos CV e fatais. ^37^ ^,^ ^332^ No estudo HYVET, que incluiu pacientes com idade acima de 80 anos ativos e não frágeis, além de redução da PAS > 150 mmHg (média 144 mmHg), evidenciou-se reduções significantes na mortalidade, AVE fatal e também IC. ^333^ Em metanálise e revisão sistemática incluindo nove estudos, os autores demonstraram robusta evidência de que reduções > 150/90 mmHg trazem redução de mortalidade, AVC e eventos cardíacos em idosos. ^334^ No entanto, recentes ensaios clínicos mostraram evidências de benefícios de metas mais baixas de PA em pacientes idosos. ^87^ ^,^ ^335^ No subgrupo de indivíduos do estudo SPRINT com mais de 75 anos de idade, ^87^ o grupo de tratamento intensivo que atingiu uma média de PA-alvo de 124/62 mmHg teve uma redução significativa de eventos CV e IC e também de morte por todas as causas quando comparado com a meta menos intensiva, na qual a média alcançada foi 135/67 mmHg. Neste estudo, sugere-se um benefício do tratamento mais intensivo mesmo nos idosos mais frágeis, mas houve maior incidência de quedas e maior ocorrência de prejuízo da função renal no controle mais intensivo. Outro dado relevante no estudo SPRINT é que a medida de PA foi realizada sem supervisão e costuma ser mais baixa do que a convencional. Assim, a meta atingida equivale a valores entre 130-139 mmHg, se comparada com a medida realizada nos estudos anteriores. ^321^ Em outra metanálise, Bavish et al. ^335^ demonstraram que o controle de PA mais agressivo em pacientes com idade ≥ 65 anos reduziu mais eventos CV, mas apresenta muitas limitações metodológicas e revelou maior ocorrência de insuficiência renal no grupo de controle mais intensivo.

De maneira geral, a recomendação de metas para pacientes idosos Brazileiros ≥ 60 anos é alcançar valores de acordo com sua condição global (hígidos ou frágeis), conforme o Quadro 6.2 , também constante do Capítulo 14. Em idosos, o correto é tratar metas como individuais, considerando-se a qualidade de vida do paciente, o risco de quedas, a fragilidade, a independência e a presença de comorbidades.

**Quadro 6.2:** – Metas de tratamento para idosos considerando a condição global e a medida da pressão arterial no consultório.

	PAS de consultório		PAD de consultório	
Condição global ^1^	Limiar de tratamento	Meta pressórica ^4^ ^, ^ ^5^	Limiar de tratamento	Meta ^8^
Hígidos ^2^	≥140 (I, A)	130-139 (I, A) 6	≥90	70-79
Idosos frágeis ^3^	≥160 (I, C)	140-149 (I, C) ^7^	≥90	70-79

1: mais importante a condição funcional que idade cronológica; 2: incluindo fragilidade leve; 3: fragilidade moderada a severa; 4: incluindo idosos com comorbidades: DM, DAC, DRC, ACV/EIT (não se refere à fase aguda); 5: avaliar ativamente a tolerabilidade, inclusive possíveis sintomas atípicos; 6: umna meta mais rígida (125-135 mmHg) pode ser obtida em casos selecionados, especialmente em idosos motivados, com < 80 anos, apresentando ótima tolerabilidade ao tratamento; 7: limites mais elevados em caso de sobrevida limitada e ausência de sintomas. A redução da PA deve ser gradual; 8: PAD = evitar < 65-70 mmHg em portadores de DAC clinicamente manifesta.

**Table:** 

Mensagens principais
Nos hipertensos de risco CV baixo ou moderado, a meta de tratamento é alcançar valores inferiores a 140/90 mmHg.
No hipertenso com DAC, a meta terapêutica é obter PA<130/80 mmHg, mas a PA diastólica deve ser mantida com valores acima de 70 mmHg.
Para os hipertensos com IC ou episódio prévio de AVE, o tratamento anti-hipertensivo deve ser titulado até alcançar a meta de PA<130/80 mmHg, mas a concomitância de doença DAC e idade avançada, comum em tal situação, limita a redução da PA até 120/70 mmHg.
Nos hipertensos com DRC, o objetivo do tratamento é alcançar PA < 130/80 mmHg, mas sempre com monitorização de eventos adversos, especialmente redução da função renal e alterações eletrolíticas.
O tratamento da hipertensão nos indivíduos diabéticos deve procurar manter valores < 130/80 mmHg, evitando-se a redução acentuada da PA para valores inferiores a 120/70 mmHg.

## 7. Equipe Multiprofissional

## 7.1. A Importância da Abordagem Multiprofissional no Controle da Hipertensão

A hipertensão arterial (HA) não controlada continua sendo fator de risco cardiovascular (FRCV) amplamente prevalente no Brazil e no mundo. Diversos estudos nacionais e internacionais demonstraram, de forma consistente, a superioridade do controle da pressão arterial (PA) com a abordagem multiprofissional, comparada com o tratamento convencional, com acréscimo na qualidade da assistência, melhor adesão e sucesso terapêutico, redução de FRCV, morbidade e mortalidade CV. ^336^ ^-^ ^341^ Cuidados dispensados por equipes e decisões tomadas de forma compartilhada estão associados à redução de custos e a melhores resultados no tratamento da HA. ^342^ ^,^ ^343^

Vários objetivos exigem diferentes estratégias de atuação, entre as quais assistência centrada no paciente, capacidade de integração entre os profissionais, compartilhamento de objetivos e metas e colaboração na tomada de decisões, com a participação do próprio paciente. ^344^ Um estudo longitudinal Brazileiro, retrospectivo, com o objetivo de avaliar o efeito da intervenção multiprofissional em hipertensos idosos com 80 anos ou mais (n = 71), acompanhados em serviço especializado por um tempo médio de 15,2 anos mostrou a redução dos valores da PA e o aumento das taxas de controle dela, com otimização do tratamento. ^345^

Uma revisão sistemática composta por 80 estudos realizados nos Estados Unidos, no período de 1980 a 2012, mostrou a eficácia do atendimento em equipe. Observou-se o aumento de 12% nas taxas de controle da PA, com a redução da mediana da PA sistólica (PAS) de -5,4 mmHg e da PA diastólica (PAD) de -1,8 mmHg, principalmente quando enfermeiros e farmacêuticos compunham a equipe. Tais resultados foram observados em diversas configurações multiprofissionais e em diferentes grupos populacionais norte-americanos. ^346^

Uma equipe multiprofissional forma-se a partir de ações integradas entre todos os seus membros. O que determina essa unidade não é apenas a dimensão espacial, mas principalmente o desenvolvimento de ações conjuntas, em que cada categoria profissional atua como ator independente em ações específicas de sua profissão, porém reconhecendo e associando suas ações aos demais membros da equipe.

A atuação multiprofissional tem sido utilizada com sucesso em serviços de atenção primária ^347^ , secundária ^336^ e terciária ^341^ à saúde. O nível de complexidade das ações profissionais pode distanciá-los de uma ação conjunta. Por outro lado, é na promoção da saúde e no nível de atenção primária que as atuações têm maior poder de integração, e o desempenho da equipe concretiza-se de forma efetiva. O trabalho em equipe tem vantagens como incentivar o paciente a replicar seus conhecimentos e atitudes, favorecer ações de pesquisa em serviço e proporcionar crescimento profissional aos membros da equipe e, consequentemente, da instituição. ^337^ ^,^ ^343^ ^,^ ^348^ ^-^ ^350^

A implementação da abordagem multidisciplinar exige mudanças organizacionais nos níveis de atenção do sistema de saúde, destacando sua importância também com relação a cuidados em *home care* . ^336^ A abordagem multidisciplinar da HA classifica-se com grau de recomendação I, nível de evidência A ( Quadro 7.1 ). ^351^ Portanto, a assistência à saúde passa a ter a característica central de um trabalho coletivo e complexo, em que a interdisciplinaridade e a multiprofissionalidade são necessárias. ^341^ ^,^ ^347^ ^,^ ^352^ Existem atribuições comuns a todos os membros da equipe e atribuições especificas a cada função (GR: I, NE: A).

**Quadro 7.1:** – Estratégias de atuação da equipe multiprofissional centradas no paciente

Estratégias	Descrição	Exemplos	Membro da Equipe
Educação do paciente	Abordagem didática ou interativa para informar e educar os pacientes	Sessões educativas presenciais ^389^ Materiais impressos presenciais ^389^ ^, ^ ^390^ Materiais impressos via correio ^390^ Meios audiovisuais e Educação a distância ^391^	MED, ENF, FARM, NUT, PSI, ACS
Apoio social	Envolvimento de familiares, amigos ou outros indivíduos para ajudar os pacientes a utilizarem os fármacos, conforme prescrito	Reuniões de grupos de apoio ^347^ ^,^ ^350^ Educação familiar ^348^	FAM, AM, CD, ACS, ASS
Letramento e motivação do paciente	Motivar os pacientes a tomar a medicação, conforme prescrito, e remover obstáculos que prejudiquem sua motivação	Intervenções motivacionais ^386^ ^, ^ ^389^ Implantar ações de letramento em saúde ^362^ ^,^ ^392^ ^,^ ^393^	MED, ENF, NUT, PSI EF, FIS, AM, CD, FAM
Automonitoramento da PA e uso de tecnologias	Envolver os pacientes a monitorar a PA e a adesão ao tratamento	Automedidas da PA ^391^ Monitorização residencial da PA ^394^ ^,^ ^395^ Telemonitoramento da PA ^343^ ^,^ ^390^ ^,^ ^396^ ^,^ ^397^	MED, ENF, PAC, FAM, CD, ACS
Comunicação ou interação com os prestadores de serviços e entre membros da equipe	Melhorar a comunicação entre os pacientes e a equipe multiprofissional e outros prestadores e entre membros da equipe	Treinamento de habilidades de comunicação entre pacientes e a equipe multiprofissional e entre membros da equipe ^390^ Intervenções digitais interativas ^350^ ^,^ ^358^ ^,^ ^359^ ^,^ ^398^	MED, ENF, NUT, EF, FIS, PSI, ASS. ACS
Facilitar o acesso aos serviços de saúde	Facilitar o agendamento de consultas em horários compatíveis com as necessidades dos pacientes	Pacientes de outros municípios Idosos dependentes de acompanhamento de terceiros ^345^ ^,^ ^399^	ACS, ASS

AM: Amigo; ACS: agentes comunitários de saúde; ASS: assistente social; CD: cuidadores; ENF: enfermeiro; EF: profissional de educação física; FAM: familiar; FARM: farmacêutico; FIS: fisioterapia, MED: médico; NUT: nutricionista; PAC: paciente; PS: psicólogo.

## 7.2. Formação e Atuação da Equipe

### 7.2.1. Profissional Médico – Ações Específicas

No nível primário, há a participação do médico generalista e, nos demais níveis, a presença de cardiologista, nefrologista e especialista em hipertensão. As ações específicas do médico são as seguintes: ^341^

• Consulta médica (detalhada no Capítulo 4).
• Responsável por diagnóstico, estratificação de risco e orientação das condutas terapêuticas não farmacológicas e farmacológicas.
• Avaliação clínica dos pacientes, pelo menos, duas vezes por ano.
• Referência e contra referência dentro do sistema de saúde.

Além do médico, a participação de profissionais de diversas áreas da saúde (enfermeiros, farmacêuticos, assistentes sociais, nutricionistas, profissionais de educação física, fisioterapeutas, pedagogos, psicólogos), com formação de equipes multiprofissionais no atendimento de hipertensos, ocorre há várias décadas nos países desenvolvidos. ^353^ ^,^ ^354^

### 7.2.2. Profissional Enfermeiro – Ações Específicas

As ações específicas do enfermeiro são as seguintes:

• Promover o acolhimento dos pacientes, identificando conjuntamente com o usuário os diversos obstáculos e barreiras presentes no cotidiano e incentivar apoio seu enfrentamento.
• Capacitar as pessoas para aumentar seu controle sobre os determinantes que influenciam o autocuidado e, assim, melhorar sua saúde. As habilidades avançadas de comunicação, técnicas de mudança de comportamento, educação do paciente e habilidades de aconselhamento são elementos essenciais que aprimoram os sistemas de saúde e mostram-se necessárias para auxiliar os pacientes com problemas crônicos. ^355^ O próprio Ministério da Saúde (MS) destaca que o desenvolvimento de ações referentes à promoção de saúde e à prevenção das doenças crônicas não transmissíveis (DCNT), em especial a HA e o diabetes melito (DM), é um enorme desafio. ^356^
• Incentivar o autocuidado.
• Planejar estratégias para promover e avaliar a adesão dos pacientes às condutas prescritas com técnicas educacionais, motivacionais, cognitivas e uso das tecnologias. ^344^ ^,^ ^357^ ^-^ ^361^
• Promover ações educacionais para o letramento em saúde com os usuários. ^344^ ^,^ ^362^
• Visitas domiciliares, visando a reforçar a utilização da medicação e colaborar no gerenciar de cuidados e/ou de tecnologia para promover o uso correto, como ajudar o usuário a criar rotinas e hábitos para a tomada da medicação. ^363^ ^,^ ^364^

#### 7.2.2.1. Ações Específicas da Enfermagem na Atenção Primária

As equipes que atuam na atenção básica devem procurar seguir o princípio do “cuidado centrado na pessoa”, no qual o indivíduo é o ator principal das ações de cuidado de forma singularizada. Os profissionais devem ajudar o usuário a desenvolver conhecimentos, aptidões, competências e confiança necessária para gerir e tomar decisões embasadas sobre sua própria saúde de maneira mais efetiva. O cuidado é construído com as pessoas, de acordo com suas necessidades e potencialidades na busca de uma vida independente e plena. Para tanto, o MS publicou a Portaria 2.436, de 21 de setembro de 2017, ^365^ que determina diretrizes para as ações da equipe de enfermagem, como:

• Realizar atenção à saúde aos indivíduos e famílias vinculadas às equipes e, quando indicado ou necessário, no domicílio e/ou nos demais espaços comunitários (escolas, associações entre outras), em todos os ciclos de vida.
• Realizar consulta de enfermagem e procedimentos, solicitar exames complementares e prescrever medicações conforme protocolos, diretrizes clínicas e terapêuticas ou outras normativas técnicas estabelecidas pelo gestor federal, estadual, municipal ou do Distrito Federal, observadas as disposições legais da profissão.
• Realizar e/ou supervisionar acolhimento com escuta qualificada e classificação de risco, de acordo com protocolos estabelecidos.
• Realizar estratificação de risco e elaborar plano de cuidados para as pessoas que possuem condições crônicas no território, junto aos demais membros da equipe.
• Realizar atividades em grupo e encaminhar, quando necessário, usuários a outros serviços, conforme fluxo estabelecido pela rede local.
• Planejar, gerenciar e avaliar as ações desenvolvidas por técnicos/auxiliares de enfermagem, agente comunitário de saúde (ACS) e agentes de combate a endemias em conjunto com os outros membros da equipe. ^365^
• Supervisionar as ações do técnico/auxiliar de enfermagem e ACS.
• Implementar e manter atualizados rotinas, protocolos e fluxos relacionados com sua área de competência na Unidade Básica de Saúde.
• Exercer outras atribuições conforme legislação profissional, que sejam de responsabilidade na sua área de atuação.

## 7.2.3. Profissional Nutricionista – Ações Específicas

Uma recente metanálise ^366^ mostrou que a orientação nutricional é mais efetiva na diminuição da PA quando ser realiza esta por equipe multidisciplinar composta por nutricionista. Na atenção primária, a consulta do nutricionista mostrou ser mais efetiva na melhora da qualidade da dieta. ^367^

### *7.2.3.1. Consulta do Nutricionista* ***367,368***

Na consulta com o nutricionista, devem ser contemplados os seguintes pontos:

• Anamnese alimentar com avaliação da rotina de consumo, número de refeições, horários, alimentos ingeridos e quantidades, além da frequência de consumo de alimentos cardioprotetores.
• Avaliação antropométrica: medida de peso e altura, medida da circunferência abdominal e cálculo do índice de massa corporal.
• Prescrição e orientação da dieta com base no diagnóstico médico e exames laboratoriais.
• Acompanhamento das mudanças dietéticas e evolução antropométrica.
• Participação em ações com a população.

### 7.2.3.2. Ações Coletivas do Nutricionista

As ações devem ser as seguintes:

• A orientação nutricional deve ser centrada nas mudanças de impacto na diminuição da PA: perda de peso, ^369^ ^,^ ^370^ aumento do consumo de frutas e vegetais ^371^ ^-^ ^373^ e diminuição do consumo de sódio. ^374^ ^,^ ^375^
• Atualmente, a utilização de recursos tecnológicos gratuitos na área de nutrição representa um importante recurso de informação em larga escala, e seu uso deve ser incentivado. ^376^ ^,^ ^377^

## 7.2.4. Profissional de Educação Física – Ações Específicas

O comportamento sedentário (tempo sentado ou vendo TV/celular/computador) e a inatividade física (prática de atividades físicas AF aquém do adequado para a saúde) constituem um importante problema de saúde pública, pois elevam os custos do tratamento e reduzem a expectativa de vida. ^378^ ^-^ ^380^ Cabe ao profissional de educação física aplicar as recomendações expostas no Capítulo 8 para minimizar esses comportamentos. Nesse sentido, o profissional deve:

• Recomendar a redução do comportamento sedentário nas populações adulta e adolescente.
• Incentivar o cumprimento das recomendações mínimas de atividades físicas (AF) para toda a população por meio de ações coletivas detalhadas a seguir. A prática dessas atividades contribui para reduzir a mortalidade por doenças cardiovasculares, mesmo quando há comportamento sedentário. ^381^
• Programar, ministrar e supervisionar programas de exercícios físicos (EF), presenciais ou a distância, individuais ou em grupo, adequados às realidades locais e às características específicas de cada pessoa. Convém fazer uso de recursos tecnológicos (celular, internet, *games* , vídeos etc.) para motivar a participação, controlar a frequência e a intensidade de execução e dar dicas de como aumentar a atividade física regular diária;
• Realizar avaliações pré-participação, indicar a avaliação médica prévia nos casos recomendados e fazer reavaliações regulares para verificar a efetividade da prática e ajustar sua progressão.

### 7.2.4.1. Ações Coletivas dos Profissionais de Educação Física e Fisioterapia

• Dentro da equipe multiprofissional, deve-se estimular, por meio dos pacientes, dos representantes da comunidade e da sociedade civil, o desenvolvimento de AF comunitárias, visto que as atividades de lazer são benéficas para a manutenção da qualidade de vida na comunidade. ^382^
• O profissional de educação física, dentro da equipe, deve demonstrar, por meio de resultados positivos, que a prevenção e o tratamento da HA depende da combinação da redução do comportamento sedentário e do aumento da AF com outros fatores, como uma alimentação saudável e a redução de peso corporal, estresse, consumo de sal e álcool, fumo etc. Além disso, deve incentivar a adesão ao tratamento medicamentoso e à medida frequente da PA, favorecendo melhor controle da doença.
• A criação de Ligas e Associações de portadores de HA é uma estratégia que aumenta a adesão do paciente ao tratamento, e esses profissionais podem participar da equipe de saúde que auxilia essas instituições.
• Atividades pontuais, como Campanhas de Prevenção e Combate à Hipertensão, são estratégias eficientes e importantes para que os pacientes recebam informações de saúde. Os profissionais de educação física e fisioterapia têm atuação importante nesse contexto.

## 7.3. Ações da Equipe Multiprofissional

Nos níveis primário e secundário de atenção, além do médico, a equipe multiprofissional pode ser composta por enfermeiro, técnico e auxiliar de enfermagem, nutricionista, psicólogo, assistente social, profissional de educação física, fisioterapeuta, farmacêutico, musicoterapeuta, funcionários administrativos e agentes comunitários de saúde, não sendo necessária a existência de todos componentes para o início da ação. ^383^ ^,^ ^384^

Considerando a Política Nacional de Atenção Básica (Portaria 2.436, de 21 de setembro de 2017), a qual define em seu Art. 2 ^o^ , “a atenção básica é o conjunto de ações de saúde individuais, familiares e coletivas que envolvem promoção, prevenção, proteção, diagnóstico, tratamento, reabilitação, redução de danos, cuidados paliativos e vigilância em saúde, desenvolvida por meio de práticas de cuidado integrado e gestão qualificada, realizada com equipe multiprofissional e dirigida à população em território definido, sobre as quais as equipes assumem responsabilidade sanitária”. ^365^

As superposições de funções poderão ser minimizadas pelo estabelecimento de regras claras e pela harmonia entre o grupo. Deve-se considerar que o processo educativo e as mudanças de atitudes são lentas, sendo essenciais para o alcance das metas a uniformização da linguagem e a prática de uma comunicação clara, objetiva e equilibrada. ^343^ Os membros da equipe devem atuar de acordo com as especificidades de suas formações, respeitando os limites definidos pelas diretrizes e pelos respectivos conselhos profissionais. Também é importante conhecer a ação individual dos outros membros. ^347^ ^,^ ^384^

A formação de equipes multiprofissionais no atendimento de hipertensos ocorre há várias décadas nos países desenvolvidos. ^341^ ^,^ ^348^ ^,^ ^350^ ^,^ ^385^ As ações educativas e terapêuticas podem ser desenvolvidas com grupos de pacientes, seus familiares e a comunidade, considerando que a implementação de qualquer um dos métodos deve respeitar as particularidades sociais e culturais, locais e regionais. Estratégias mais modernas podem envolver mídias sociais e técnicas de educação a distância. ^338^ ^,^ ^351^ ^,^ ^357^ ^,^ ^362^ ^,^ ^386^ ^,^ ^387^ Alguns exemplos de estratégias de atuação de equipe multiprofissional centradas no paciente, com evidências de melhor controle da PA, são expressos no Quadro 7.1 .

Em uma recente análise sobre o futuro da HA, Dzau & Balatbat ^388^ afirmaram que, até os dias atuais, o atendimento ao hipertenso tem sido feito por prestadores de cuidados não alinhados, de modo fragmentado, com informações isoladas, sendo necessárias melhores condições de coordenação e integração entre os prestadores de cuidados, em diferentes locais. Com relação ao futuro, explicam que o controle ou a prevenção da HA dependerão da convergência bem-sucedida e de avanços das ciências tecnológicas e digitais, biotecnológicas e biomédicas, com destaque para a atuação multidisciplinar. ^388^

**Table:** 

Mensagens principais
No contexto da atenção primária ao hipertenso, compete ao médico realizar: diagnóstico, estratificação de risco, conduta terapêutica não farmacológica e farmacológica, pelo menos duas vezes por ano.
O atendimento populacional deve ter como principais orientações a manutenção de peso dentro da faixa de normalidade, o aumento do consumo de frutas e vegetais e a diminuição do consumo de sódio na dieta.
Dentro da equipe multiprofissional, o profissional de educação física deve recomendar a redução do comportamento sedentário e estimular a recomendação mínima de atividade física, visando à aquisição de hábitos saudáveis para a manutenção da qualidade de vida na comunidade.
O cuidado de enfermagem deve ser centrado na pessoa, aumentando-se o acesso às informações básicas, de modo compreensível e que auxilie na decisão do autocuidado através de ações individuais e coletivas, por meio de consulta de enfermagem, visita domiciliar e atividades educacionais em grupo.
Estratégias de atuação de equipe multiprofissional centradas no paciente, com evidências de melhor controle da PA devem ser implementadas pela equipe multiprofissional.

## 8. Tratamento Não Medicamentoso

## 8.1. Introdução

Pressão arterial (PA) elevada, tabagismo, obesidade, dieta não saudável e atividade física insuficiente são fatores de risco cardiovasculares (FRCV) estabelecidos e alvos de intervenções para controle da hipertensão arterial (HA). Nos últimos anos, algumas modalidades terapêuticas não convencionais têm sido investigadas, envolvendo adoção de respiração lenta, musicoterapia e espiritualidade. Neste capítulo sobre o tratamento não medicamentoso (TNM), discutem-se as evidências que embasam as condutas recomendadas sobre tabagismo, padrão alimentar, sódio, potássio, laticínios, chocolate e produtos com cacau, vitamina D, suplementos e substitutos, perda de peso, consumo de bebidas alcoólicas, atividade e exercício físicos, respiração lenta, controle de estresse, espiritualidade e religiosidade.

## 8.2. Tabagismo

O tabagismo persiste como um dos principais FRCV, e o consumo não apenas de cigarro, mas também de charuto, cigarrilha, cachimbo, narguilé e cigarro eletrônico, permanece particularmente elevado em alguns países e associado a risco CV aumentado ^400^ (GR: I, NE: A). No Brazil, verificou-se uma tendência decrescente nos últimos 15 anos, mas não uniformemente distribuída ^401^ (GR: IIa, NE: B). O tabagismo apresenta potencial considerável de causar dano, como aceleração de processos aterotrombóticos e elevação temporária da PA. O uso de tabaco eleva a PA cerca de 5 a 10 mmHg, em média, ^402^ mas não há estudos mostrando o efeito benéfico da cessação do tabagismo sobre o controle da HA. Independentemente, deve ser enfatizada a cessação, devido ao risco CV e de neoplasias. ^403^ Os medicamentos para a cessação (como bupropiona de liberação sustentada, vareniclina, goma de nicotina, pastilha, *spray* nasal e adesivos) são eficazes para ajudar os fumantes a parar com o tabagismo ^404^ (GR: IIa, NE: B).

## 8.3. Padrão Alimentar

Os padrões alimentares considerados saudáveis têm sido associados à redução da PA. A dieta DASH ( *Dietary Approaches to Stop Hypertension* ) foi capaz de reduzir a PA, sendo o efeito atribuído ao maior consumo de frutas, hortaliças, laticínios com baixo teor de gordura e cereais integrais, além de consumo moderado de oleaginosas e redução no consumo de gorduras, doces e bebidas com açúcar e carnes vermelhas. O efeito hipotensor decorre do padrão de dieta ( Quadro 8.1 ), mais do que de seus componentes individuais – alto teor de potássio, cálcio, magnésio e fibras, com quantidades reduzidas de colesterol e gordura total e saturada ^405^ ^,^ ^406^ (GR: I, NE: A). A associação da dieta DASH com restrição de sódio ^406^ resultou em redução na PA sistólica (PAS) de 11,5 mmHg nos indivíduos hipertensos e 7,1 mmHg nos normotensos, em comparação com a dieta com alto teor de sódio. Metanálises de ensaios clínicos randomizados confirmaram o efeito redutor na PA ^406^ ^,^ ^407^ (GR: I, NE: A). Alguns estudos sugerem que a adesão à dieta DASH está associada a menor risco de acidente vascular encefálico (AVE) ^408^ ^,^ ^409^ (GR: IIa, NE: B), mortalidade cardiovascular ^410^ (GR: I, NE: A) e doença renal ^411^ (GR: I, NE: A).

**Quadro 8.1:** – Exemplo de porções e quantidades de alimentos recomendados em dieta do tipo DASH a serem consumidas diariamente ou por semana por quem dispende cerca de 2.000 kcal/dia

Grupo de alimentos	Porções diárias	Tamanho das porções/unidade
Frutas	4-5	1 fruta média 1/4 de xícara de frutas secas 1/2 xícara de frutas frescas, congeladas ou enlatadas 177 mL de suco de frutas
Vegetais	4-5	1 xícara de vegetais com folhas crus 1/2 xícara de vegetais cozidos 177 mL de suco de vegetais
Laticínios dietéticos	2-3	237 mL de leite 1 xícara de iogurte 42 g de queijo
Grãos e derivados**	7-8	1 fatia de pão 1 xícara de cereal pronto para comer* 1/2 xícara de arroz cozido, macarrão ou cereal
Carnes magras, aves e peixes	≤ 2	85 g de carne magra cozida, aves sem pele ou peixes
Nozes, sementes e leguminosas secas***	4-5 por semana	1/3 xícara ou 42 g de nozes 1 colher de sopa ou 14 g de sementes 1/2 xícara de feijão seco cozido

* Os tamanhos das porções variam entre 1/2 xícara e 1 1/4 de xícara. ** Milho, aveia, granola, arroz integral. *** Castanha-de-caju, castanha-do-pará, amêndoas, amendoim, feijão, lentilha. Adaptado de Fuchs, 2001.422

A dieta do Mediterrâneo, assim como a DASH, é rica em frutas, hortaliças e cerais integrais, além de ser pobre em carnes vermelhas. Apresenta teor elevado de gorduras, devido ao consumo de quantidades generosas de azeite de oliva (rico em ácidos graxos monoinsaturados) e inclui o consumo de peixes e oleaginosas, além da ingestão moderada de vinho tinto ^412^ (GR: IIa, NE: B). Essa dieta reduz o risco de problemas cardiovasculares ^413^ ^,^ ^414^ (GR: IIa, NE: A), mas os efeitos sobre a pressão são modestos ^414^ ^-^ ^417^ (GR: IIa, NE: B).

## 8.4. Sódio da Dieta

A ingestão habitual de sódio em todo o mundo foi estimada em 4 g/dia ^418^ (GR: IIa, NE: B), enquanto a ingestão recomendada para indivíduos hipertensos e para a população em geral é até 2 g/dia ^419^ (GR: I, NE: A). Mais do que depender de adesão individual à restrição de sódio, que diminui a longo prazo, estão em andamento iniciativas governamentais junto à indústria alimentícia para reduzir o teor de sódio dos alimentos. Dados epidemiológicos mostram que a ingestão de sódio se associa diretamente à elevação da PA, e ensaios clínicos randomizados caracterizaram o efeito hipotensor da restrição de sódio. A prova de conceito apoia-se ainda em curva dose-resposta, mostrando que mesmo uma pequena redução no consumo de sódio consegue produzir efeito, que é mais pronunciado em indivíduos hipertensos, em negros e em idosos ^420^ (GR: I, NE: A). A restrição na ingestão de sódio para ± 1.800 mg/dia associou-se à redução de 5,4 mmHg na PAS em indivíduos hipertensos ^421^ (GR: I, NE: A). São exemplos de alimentos ricos em sódio: carnes processadas (presunto, mortadela, salsicha, linguiça, salame), *bacon* , carne-seca, *nuggets* ; enlatados (extrato de tomate, conserva de milho, de ervilhas), queijos (amarelos: parmesão, provolone, prato), temperos prontos (Arisco ^®^ , Sazon ^®^ , molho de soja [ *shoyu* ], molho inglês, *ketchup* , mostarda, maionese, extrato concentrado, temperos prontos, amaciantes de carne e sopas desidratadas) e lanches industrializados ( *chips* , batata frita e salgadinhos). ^422^ Uma parte da estratégia para reduzir o sal é ler os rótulos nutricionais de todos os alimentos e escolher aqueles com baixo teor de sal (cloreto de sódio) e outras formas de sódio, destacando-se o consumo de vegetais frescos, congelados ou enlatados “sem adição de sal” e o uso de ervas, especiarias e misturas de temperos sem sal para cozinhar e à mesa. Convém cozinhar arroz, macarrão e cereal quente sem sal e escolher alimentos com baixo teor de sódio, preterindo alimentos congelados, pizza, misturas embaladas, sopas ou caldos enlatados e molhos para salada. Sempre que possível, convém lavar os alimentos enlatados, como o atum, para remover o sódio. Alguns tipos de sal de cozinha (sal rosa do Himalaia e sal marinho, entre outros) apresentam o mesmo conteúdo de cloreto de sódio que o sal de cozinha ou o sal grosso.

## 8.5. Potássio

As dietas com alto teor de sódio geralmente possuem baixo teor de potássio, as quais estão associadas à maior incidência de HA. Vários ensaios clínicos randomizados conduzidos em agregados populacionais testaram a substituição do sal de cozinha à base de cloreto de sódio por apresentações de sal com baixo teor de sódio e elevado de potássio, os quais resultaram em redução da PA ^423^ ^-^ ^428^ (GR: I, NE: A). A magnitude do efeito sobre a pressão varia com o consumo de sódio na dieta e a extensão da substituição para fontes alternativas de alimentação dos habitantes daquela população. Uma metanálise prévia ^428^ (GR: I, NE: A) teve efeito confirmado para substituição a curto e longo prazos em indivíduos adultos e idosos, embora o efeito hipotensor pareça ser mais pronunciado em indivíduos hipertensos, com uma diferença média resumida, com relação ao grupo controle, de -8,87 mmHg (IC95%: -11,19 a -6,55) na PAS e -4,04 mmHg (IC95%: -5,70 a -2,39) na PA diastólica (PAD) ^42^ (GR: I, NE: A). Uma metanálise de intervenções com restrição de sódio mostrou que seis estudos com dois meses a três anos com alta qualidade que utilizaram substitutos do sal (cloreto de potássio em substituição ao cloreto de sódio variando de 25% a 50%) reduziram significativamente a PAS (-5,7 mmHg; IC95% -8,5 a -2,8) e a PAD (-2,0 mmHg; IC95% -3,5 a -0,4) na China ^429^ (GR: I, NE: A). São alimentos ricos em potássio: damasco, abacate, melão, leite desnatado, iogurte desnatado, folhas verdes, peixes (linguado e atum), feijão, laranja, ervilha, ameixa, espinafre, tomate e uva-passa.

## 8.6. Laticínios

Os laticínios representam um grupo heterogêneo de alimentos, e seu impacto sobre a saúde deve ser avaliado considerando todos os seus componentes. Apesar de serem ricos em ácidos graxos saturados (na versão integral), podem conter constituintes com potencial efeito benéfico, como a proteína do soro do leite ( *whey protein* ), os fosfolipídios da membrana dos glóbulos de gordura, o cálcio, o magnésio, o potássio, os probióticos e as vitaminas K _1_ e K _2_ . ^430^ ^,^ ^431^ (GR: IIa, NE: B). Estudos de coorte sugerem que o consumo de laticínios apresenta associação inversa ao risco de doenças CV ^432^ ^,^ ^433^ (GR: IIa, NE: B). Alguns ensaios clínicos randomizados sugerem efeito hipotensor modesto, em especial, os realizados com laticínios pobres em gordura ^434^ ^,^ ^435^ (GR: IIa, NE: A) e com proteínas do leite ^436^ (GR: IIa, NE: B). Cabe ressaltar que as diretrizes alimentares preconizam consumo de laticínios com baixo teor de gorduras ^437^ ^,^ ^438^ (GR: IIa, NE: B).

## 8.7. Chocolate e Produtos com Cacau

Uma metanálise de dez ensaios clínicos randomizados (n = 297) identificou a redução de 4,5 mmHg (IC 95%: 3,3 a 5,9) e 2,5 mmHg (IC 95%: 1,2 a 3,9) nas pressões sistólica e diastólica, respectivamente, com o consumo aumentado de produtos com cacau. Os estudos foram muito heterogêneos e as intervenções, variadas ^439^ (GR: IIa, NE: A). Observaram-se resultados similares, mas de menor magnitude, em uma metanálise mais recente ^440^ (GR: IIa, NE: A). Dois aspectos merecem destaque, embora o número de estudos seja maior na metanálise mais recente. Um foi que persistiu a heterogeneidade entre os *trials* , com quantias variáveis de flavonóis. O segundo aspecto é que o consumo de chocolate ou produtos do cacau acrescenta calorias à dieta, que precisam ser equilibrados com algum grau de restrição alimentar.

## 8.8. Café e Produtos com Cafeína

O café, além de ser rico em cafeína ( Quadro 8.2 ), possui compostos bioativos como polifenóis, em especial os ácidos clorogênicos, o magnésio e o potássio, que podem favorecer a redução da PA. ^441^ A cafeína é capaz de elevar agudamente a PA, por mais de três horas, mas o consumo regular leva à tolerância. ^442^ A ingestão de café a longo prazo não tem sido associada a maior incidência de HA. ^443^ Pelo contrário, as metanálises de estudos de coorte mostram que o consumo de café se associou a um efeito discreto de redução no risco de hipertensão ^443^ ^,^ ^444^ (GR: IIb, NE: B). Na falta de evidências experimentais robustas, recomenda-se que o consumo de café não exceda quantidades baixas a moderadas (≤ 200 mg de cafeína) (GR: IIa, NE: B).

**Quadro 8.2:** – Conteúdo de cafeína em bebidas cafeinadas

	Volume (mL)	Cafeína (mg)
Café passado	355	235
Café instantâneo	237	63
Café expresso	30	63
Café descafeinado	237	2
Chá-preto	237	47
Chá-verde	237	28
Chá de camomila	237	0

Adaptado de van Dam RM, Hu FB, Willett WC, 2020. ^442^

## 8.9. Vitamina D

Apesar de alguns estudos observacionais sugerirem que a deficiência de vitamina D está associada à elevação da pressão ou à maior incidência de hipertensão ^445^ ^,^ ^446^ (GR: IIb, NE:A), os estudos com suplementação de vitamina D apresentam resultados inconsistentes ^447^ ^-^ ^449^ (GR: IIb, NE: A). Portanto, o papel da vitamina D no controle da pressão ainda não está estabelecido.

## 8.10. Suplementos e Substitutos

Além da redução de sódio nos alimentos processados, dispõem-se de alternativas para minimizar o efeito deletério do sódio e, ao mesmo tempo, propiciar benefício do potássio. Realizou-se um ensaio clínico randomizado em indivíduos chineses com doença cardiovascular prévia ou PAS superior a 160 mmHg, que receberam aleatoriamente uma combinação de 65% de cloreto de sódio, 25% de cloreto de potássio e 10% de sulfato de magnésio ou sal de cozinha com 100% de cloreto de sódio. A intervenção resultou em uma redução média de 3,7 mmHg (1,6 a 5,9) na pressão sistólica, a qual alcançou o efeito máximo em 12 meses, diminuindo 5,4 mmHg (2,3 a 8,5) ^426^ (GR: IIa, NE: B). Um ensaio clínico randomizado conduzido em indivíduos hipertensos e suas famílias detectou resultados similares, mas de menor magnitude, após 36 meses ^28^ (GR: I, NE: A).

Apesar de a suplementação de cálcio poder exercer um efeito discreto na prevenção de hipertensão ^450^ (GR: IIa, NE: B), não está estabelecido seu papel no tratamento. Uma metanálise de 12 ensaios clínicos randomizados identificou que o uso de multivitaminas e multiminerais foi capaz de reduzir a PA em indivíduos com doenças crônicas. Em uma análise de subgrupo de 58 indivíduos hipertensos, houve a redução de 7,98 mmHg (14,95 a 1,02) na PAS, mas, para PAD, a significância foi limítrofe ^451^ (GR: IIa, NE: B).

## 8.11. Perda de Peso

Foi bem registrado o efeito hipertensor do ganho de peso. Há uma relação praticamente linear entre PA e índices de obesidade. A adiposidade corporal excessiva, especialmente a visceral, é um fator de risco importante para a elevação da PA, que pode ser responsável por 65 a 75% dos casos de HA. ^452^ A perda ponderal reduz a PA, mesmo sem alcançar o peso corporal desejável. Em uma metanálise incluindo 25 estudos, a perda ponderal de 5,1 kg reduziu, em média, a PAS em 4,4 mmHg e a PAD em 3,6 mmHg ^453^ (GR: I, NE: A). Para indivíduos com sobrepeso ou obesidade, a perda ponderal é uma recomendação essencial no tratamento da HA. A avaliação da adiposidade corporal não deve ser limitada à análise do índice de massa corporal (IMC), mas, sim, incluir parâmetros de adiposidade corporal central, como a circunferência da cintura (CC). O ideal é alcançar e manter um peso corporal saudável, representado pelo IMC (kg/m ^2^ ) < 25 em adultos (GR: I, NE: A) e, segundo o Ministério da Saúde, IMC entre 22 e < 27 em idosos, e CC (cm) < 90 em homens e < 80 em mulheres. As evidências de uma metanálise com dados de participantes em quatro continentes mostraram que, para cada cinco unidades acima de um IMC > 25, o risco de morte prematura aumentou cerca de 31%, assim como o risco de 49% para mortalidade cardiovascular ^454^ (GR: IIa, NE: B).

## 8.12. Consumo de Bebidas Alcoólicas

Há relação linear entre consumo de bebidas alcoólicas e PA, e o consumo abusivo está associado a maior prevalência de HA. Um metanálise recente, incluindo 36 ensaios clínicos randomizados e 2.865 participantes, detectou que, em indivíduos que bebiam até dois drinques por dia, a redução no consumo de bebidas alcoólicas não se associou à redução significativa da PA. Contudo, em indivíduos que bebiam mais do que dois drinques por dia, a redução no consumo de bebidas alcoólicas associou-se à maior redução da PA, cerca de 5,5 mmHg (6,70 a 4,30) na PAS e 3,97 (4,70 a 3,25) na PAD. Essa redução foi mais pronunciada naqueles que bebiam seis drinques ou mais por dia e reduziram a ingestão em cerca de 50% ^15^ (GR: IIa, NE: B). Entre indivíduos que consomem bebidas alcoólicas, a ingestão não deve ultrapassar 30 g de álcool/dia, ou seja, uma garrafa de cerveja (5% de álcool, 600 mL), ou duas taças de vinho (12% de álcool, 250 mL) ou uma dose (42% de álcool, 60 mL) de destilados (uísque, vodca, aguardente). Esse limiar deve ser reduzido à metade para homens com baixo peso, mulheres, indivíduos com sobrepeso e/ou triglicerídeos elevados. Indivíduos abstêmios não devem ser induzidos a consumir bebidas alcoólicas. ^15^

## 8.13. Atividade Física e Exercício Físico

A atividade física (AF) refere-se a qualquer movimento corporal que aumente o gasto energético acima daquele em repouso, como locomoção e atividades laborais, domésticas e de lazer. O exercício físico (EF), por sua vez, refere-se à AF estruturada, organizada e com objetivo específico, como melhorar a saúde e/ou a aptidão física. ^455^ O comportamento sedentário é o tempo gasto em atividades de baixo dispêndio energético (≤ 1,5 MET), como aquelas executadas na posição sentada, reclinada ou deitada (assistir à TV, utilizar o computador, jogar *videogame* ou trabalhar). ^456^ A redução do tempo sedentário, mesmo que por curto período de tempo, diminui o risco de mortalidade ^457^ (GR: IIb, NE: B).

A prática regular de AF diminui a incidência de HA. ^458^ Além disso, os hipertensos que alcançam as recomendações de prática de AF para a saúde apresentam uma redução de 27 a 50% no risco de mortalidade, mas níveis menores também apresentam efeito benéfico ^53^ (GR: I, NE: A). No tratamento da HA, benefícios adicionais podem ser obtidos com EF estruturados, realizando-se o treinamento aeróbico complementado pelo resistido. O treinamento aeróbico possui comprovado efeito reduzindo a PA de consultório e ambulatorial, enquanto os treinamentos resistidos dinâmico e isométrico de *handgrip* (preensão manual) reduzem a PA de consultório, mas não há evidências de diminuição da PA ambulatorial. ^459^ O Quadro 8.3 apresenta a magnitude do efeito desses treinamentos (GR: I, NE: A). ^459^ ^-^ ^461^

**Quadro 8.3:** – Magnitude de redução da pressão arterial de indivíduos hipertensos com o treinamento físico

Treinamento	Pressão sistólica/diastólica
Aeróbico ^459^	-12,3/-6,1 mmHg*
Aeróbico ^459^	-8,8/-4,9 mmHg**
Resistido dinâmico (musculação) ^460^	-5,7/-5,2 mmHg
Resistido isométrico de preensão manual (handgrip) ^461^	-6,5/-5,5 mmHg

* Pressão de consultório. ** Monitorização ambulatorial da pressão arterial.

Outras modalidades de treinamento, como o realizado em meio líquido, ^462^ a ioga, ^463^ o tai chi chuan ^464^ e o treinamento intervalado de alta intensidade, ^465^ entre outros, também parecem reduzir a PA de consultório de hipertensos. No entanto, não há evidência documentada de efeitos sobre a pressão ambulatorial nem de seus potenciais riscos, de modo que eles ainda não são recomendados. O Quadro 8.4 apresenta as recomendações de prática de atividade física e exercício.

**Quadro 8.4:** – Recomendações de prática de atividade física e exercício físico.

Redução do Comportamento Sedentário - NE:B GR:IIb
**Levantar por 5 minutos a cada 30 minutos sentado**
Recomendação Atividade Física populacional - NE:A. GR:I
**Realizar, pelo menos, 150 minutos por semana de atividade física moderada**
Treinamento Físico - Aeróbico complementado pelo Resistido - NE:A. GR:I
Prescrição do Treinamento Aeróbico – Obrigatório
**Modalidades diversas: andar, correr, dançar, nadar, entre outras**
**Frequência: 3 a 5 vezes por semana (mais vezes - melhor)**
**Duração: 30 a 60 minutos por sessão (mais tempo - melhor)**
**Intensidade moderada definida por:**
**1) Maior intensidade conseguindo conversar (sem ficar ofegante)**
**2) Sentir-se entre “ligeiramente cansado” e “cansado” (11 a 13 na escala de Borg 20)**
**3) Manter a frequência cardíaca (FC) de treino na faixa calculada por: FCtreino = (FCmáxima – FCrepouso) x % + FCrepouso Onde:** **FC máxima** **: deve ser obtida num teste ergométrico máximo, feito em uso dos medicamentos regulares, ou pelo cálculo da FC máxima prevista pela idade (220-idade). Essa fórmula não pode ser usada em indivíduos hipertensos com cardiopatias, em uso de betabloqueadores ou inibidores de canais de cálcio não diidropiridínicos.** **FC repouso** **: deve ser medida após 5 min de repouso deitado** **%** **: utilizar 40% como limite inferior e 60% como superior**
Prescrição do Treinamento Resistido – Complementar
**2 a 3 vezes/semana**
**8 a 10 exercícios para os principais grupos musculares, dando prioridade para execução unilateral, quando possível**
**1 a 3 séries**
**10 a 15 repetições até a fadiga moderada (repetição na qual há redução da velocidade de movimento) – aproximadamente 60% de 1RM**
**Pausas longas passivas – 90 a 120 s**

Para a prescrição de AF e EF leves a moderados em indivíduos sem doença cardíaca, cerebrovascular ou renal, pode-se prescindir de uma avaliação médica prévia. Caso surjam sintomas durante a prática, deve-se interromper a atividade e procurar o médico. Indivíduos hipertensos com comorbidades, sintomas ou que pretendem fazer atividades de alta intensidade ou competitivas devem se submeter à avaliação médica prévia. ^466^ Recomenda-se o teste ergométrico para avaliar a aptidão física e prescrever exercícios físicos, ^467^ o que possibilita avaliar a resposta da PA ao esforço e confirmar a presença de doença coronariana nos indivíduos sintomáticos ou com múltiplo fatores de risco. A sessão de treinamento não deve ser realizada se a PA estiver acima de 160/105 mmHg, e recomenda-se medir a PA durante o exercício aeróbico em hipertensos hiper-reativos e diminuir a intensidade se ela estiver acima de 180/105 mmHg (GR: IIa, NE: C).

## 8.14. Respiração Lenta

A respiração lenta ou guiada requer redução da frequência respiratória para menos de 6 a 10 respirações/minuto durante 15-20 minutos/dia para promover a redução na PA casual. O ensaios clínicos randomizados utilizando respiração lenta guiada ( *device guided breathing; Resperate device* ^®^ ), analisados em metanálise prévia, não resultaram em redução significativa de PA após a exclusão de cinco estudos nos quais a indústria do equipamento estava envolvida. ^468^ Uma metanálise recente, agregando seis *trials* de exercícios de respiração lenta voluntária em comparação com a respiração natural, detectou uma redução de 6,36 mmHg (IC95%: 10,32 a 2,39) na PAS e 6,39 mmHg (IC95%: 7,30 a 5,49) na PAD, em comparação aos controles, em ensaios clínicos randomizados com até seis meses de duração. ^469^ As evidências existentes demonstram que, a curto prazo, exercícios voluntários de respiração lenta podem reduzir a PAS e a PAD de pacientes com HA portadores de doença CV (GR: IIa, NE: A). Um ensaio clínico, realizado com um número reduzido de participantes, mostrou que a respiração lenta reduz a pressão em repouso de indivíduos com HA isolada, além das respostas aos exercícios estáticos e dinâmicos ^470^ (GR: IIb, NE: B).

A associação de audição musical à respiração profunda, em comparação com ouvir música, apenas não resultou em redução estatisticamente significativa de PA. Participantes de ambos os grupos de intervenção obtiveram redução clinicamente significativa de PA ^471^ (GR: IIb, NE: B).

## 8.15. Controle de Estresse

De modo geral, as técnicas usadas no manejo de estresse não apresentam evidências robustas de eficácia, sejam terapias comportamentais, meditação transcendental (GR: IIb, NE: B), outras técnicas de meditação (GR: III, NE: C), ioga (GR: III, NE: C), terapias de relaxamento (GR: III, NE: C) e abordagens de *biofeedback* (GR: IIb, NE: B). Entre os procedimentos, a respiração lenta guiada apresentou mais evidências do que as disponíveis para acupuntura (GR: III, NE B). As indicações clínicas mostram apenas tendência à redução da PA, sejam usadas separadamente ou em conjunto ^472^ (GR: IIa, NE: B). Em duas metanálises, a musicoterapia se associou à redução significativa da PAS, ^473^ ^,^ ^474^ enquanto em outra foi observada apenas tendência à redução da PA ^475^ (GR: IIb; NE: A).

A meditação pode ser entendida como a experiência de esvaziar a mente, tornando-a livre de pensamentos; a prática de focar a concentração em um único objeto até conscientizar-se do objeto; a contemplação de um aspecto da realidade; ou ainda desenvolver determinada qualidade mental ou mesmo comportamental. ^476^ ^,^ ^477^ Uma revisão sistemática mostrou que a meditação transcendental reduziu em cerca de 4 mmHg a PAS e 2 mmHg a PAD ^478^ (GR: IIb, NE: B). Contudo, os mecanismos pelos quais a meditação reduz a PA não foram completamente elucidados, e apontaram-se limitações metodológicas nesses estudos. ^479^

## 8.16. Espiritualidade e Religiosidade

A espiritualidade está associada aos aspectos físicos, psicológicos e sociais, o que possibilita a visão integral do ser humano, colocando-o no centro da atenção e do tratamento ^480^ (GR: IIb, NE:C). Pode ser considerada como um conjunto de valores morais, mentais e emocionais que norteiam pensamentos, comportamentos e atitudes ^3^ ^,^ ^481^ (GR: I, NE: B). Diferencia-se de religião, entendida como sistema organizado de crenças, práticas e símbolos destinados a facilitar a proximidade com o transcendente ou o Divino ^3^ ^,^ ^481^ (GR: I, NE: B).

Estudos sugerem a associação entre índices de espiritualidade ou religiosidade (E/R) e taxas de mortalidade por todas as causas, mortalidade CV e câncer, bem como qualidade de vida ^482^ ^-^ ^485^ (GR: I, NE: B). Os mecanismos envolvidos envolvem modificações favoráveis de estilo de vida e em FRCV, como redução nos níveis séricos de glicose, colesterol, fibrinogênio, cortisol e citocinas inflamatórias ^481^ ^,^ ^486^ (GR: I, NE: B).

Dados os aspectos multidimensionais da E/R, assim como as características das populações estudadas, estudos observacionais avaliando a associação a PA e/ou risco de HA apresentam resultados heterogêneos, porém a maioria sugere efeitos benéficos. ^77^ ^,^ ^481^ ^,^ ^487^ ^,^ ^488^ No estudo SWAN ( *Study of Women’s Health Across the Nation* ), com mais de 1.600 mulheres de meia-idade, as práticas espirituais diárias não se revelaram protetoras para a PAS ou a HA. ^489^ Já no *Chicago Community Adult Health Study* , a frequência religiosa não se associou à HA, enquanto o hábito de orar apresentou associação positiva. A espiritualidade esteve associada à HA diastólica, enquanto o significado de perdão foi associado a menor PAD e menor probabilidade de ocorrência de HA. ^490^ Em outro estudo, a maior frequência religiosa associou-se à redução da PAD, mas não à PAS ^491^ (GR: IIa, NE: B).

Em uma revisão recente, observou-se que elementos de E/R podem interferir na adesão ao tratamento farmacológico de forma positiva, mas em outros estudos os efeitos foram opostos ou mistos, principalmente em doenças graves e crônicas ^492^ (GR: IIa, NE: C). O profissional de saúde deve identificar quais são as demandas e as expectativas do paciente, prover o apoio adequado e superar conflitos. Para tal, perguntas abertas ou questionários semiestruturados podem ser úteis ^3^ ^,^ ^493^ (GR: I, NE: B). Apesar de evidências provenientes de estudos observacionais correlacionarem E/R e HA, são poucos os estudos clínicos avaliando os efeitos de intervenções nesse domínio, principalmente em doenças CV graves, nas doenças crônicas ou em cuidados paliativos ^481^ ^,^ ^494^ (GR: IIb, NE: B).

**Table:** 

Mensagens principais
Os indivíduos hipertensos devem ser avaliados quanto ao hábito de fumar, e deve ser buscada a cessação do tabagismo, se necessário com o uso de medicamentos, pelo aumento do risco CV.
A dieta tipo DASH e semelhantes – aumento no consumo de frutas, hortaliças, laticínios com baixo teor de gordura e cereais integrais, além de consumo moderado de oleaginosas e redução no consumo de gorduras, doces e bebidas com açúcar e carnes vermelhas – deve ser prescrita.
O consumo de sódio deve ser restrito a 2 g/dia, com substituição de cloreto de sódio por cloreto de potássio, se não existirem restrições.
O peso corporal deve ser controlado para a manutenção de IMC < 25 kg/m2.
Realizar, pelo menos, 150 minutos por semana de atividade física moderada. Deve ser estimulada ainda a redução do comportamento sedentário, levantando-se por 5 minutos a cada 30 minutos sentado.

## 9. Tratamento Medicamentoso

## 9.1. Objetivos do Tratamento

A proteção cardiovascular (CV) consiste no objetivo primordial do tratamento anti-hipertensivo. A redução da pressão arterial (PA) é a primeira meta, com o objetivo maior de reduzir desfechos CV e mortalidade associados à hipertensão arterial (HA). ^5^ ^,^ ^37^ ^,^ ^164^ ^,^ ^495^ Os resultados de metanálises de estudos clínicos randomizados em pacientes hipertensos mostraram que a redução de PA sistólica de 10 mmHg e diastólica de 5 mmHg com fármacos se acompanha de diminuição significativa do risco relativo de desfechos maiores: 37% para acidente vascular encefálico (AVE), 22% para doença arterial coronariana (DAC), 46% para insuficiência cardíaca (IC) , 20% para mortalidade CV e 12% para mortalidade total. ^83^ ^,^ ^85^ ^,^ ^307^ ^,^ ^308^ ^,^ ^496^ ^,^ ^497^ Observa-se que os benefícios são maiores quanto maior o risco CV, mas ocorrem mesmo em pacientes com pequenas elevações da PA com risco CV baixo a moderado. ^307^ ^,^ ^308^ ^,^ ^496^

Destaca-se que, em sua maioria, esses achados provêm de estudos clínicos com hipertensos acima de 50 anos e de alto risco CV, em acompanhamento raramente maior que cinco anos. Portanto, os benefícios para indivíduos jovens, de risco baixo a moderado e em prazo mais longo de tratamento representam extrapolações da evidência científica disponível. ^498^ Em especial nesse grupo de pacientes, infere-se que a avaliação do impacto dos medicamentos anti-hipertensivos na proteção de órgãos-alvo pode ser útil como indicador indireto de sucesso do tratamento, notadamente a redução da massa ventricular esquerda ^499^ ^,^ ^500^ e da albuminúria. ^501^ Dessa maneira, o tratamento adequado em indivíduos abaixo de 50 anos é fortemente recomendado.

## 9.2. Princípios Gerais do Tratamento Medicamentoso

A maioria dos pacientes hipertensos necessitará de fármacos em adição às modificações do estilo de vida para alcançar a meta pressórica. ^5^ ^,^ ^37^ ^,^ ^83^ ^,^ ^164^ ^,^ ^307^ ^,^ ^307^ ^,^ ^308^ ^,^ ^495^ ^,^ ^497^ ^,^ ^502^ O Quadro 9.1 mostra as recomendações de início de tratamento com intervenções no estilo de vida e do tratamento farmacológico de acordo com a pressão arterial, a idade e o risco cardiovascular.

**Quadro 9.1:** – Início de tratamento com intervenções no estilo de vida e tratamento farmacológico de acordo com a pressão arterial, a idade e o risco cardiovascular

Situação	Abrangência	Recomendação	Classe	Nível de evidência
Início de intervenções no estilo de vida	Todos os estágios de hipertensão e pressão arterial 130-139/85-89mmHg	Ao diagnóstico	I	A
Início de terapia farmacológica	Hipertensos estágio 2 e 3	Ao diagnóstico	I	A
	Hipertensos estágio 1 de moderado e alto risco cardiovascular	Ao diagnóstico	I	B
	Hipertensos estágio 1 e risco cardiovascular baixo Indivíduos com PA 130-139/85-89 mmHg e DCV preexistente ou alto risco cardiovascular	Aguardar 3 meses pelo efeito de intervenções no estilo de vida	IIa	B
	Hipertensos idosos frágeis e/ou muito idosos	PAS≥160 mmHg	I	B
	Hipertensos idosos hígidos	PAS≥140mmHg	I	A
	Indivíduos com PA 130-139/85-89 mmHg sem DCV preexistente e risco cardiovascular baixo ou moderado	Não recomendado	III	

As cinco principais classes de fármacos anti-hipertensivos – diuréticos (DIU), bloqueadores dos canais de cálcio (BCC), inibidores da enzima conversora de angiotensina (IECA), bloqueadores dos receptores da angiotensina II (BRA) e betabloqueadores (BB) demonstraram reduções significativas da PA comparadas com placebo, acompanhadas de diminuições consideráveis dos desfechos CV fatais e não fatais, benefício relacionado fundamentalmente com a redução da PA. ^5^ ^,^ ^37^ ^,^ ^83^ ^,^ ^164^ ^,^ ^307^ ^,^ ^308^ ^,^ ^495^ ^,^ ^497^ Os BB são úteis quando há certas condições clínicas específicas: pós-infarto agudo do miocárdio (IAM) e angina do peito, IC com fração de ejeção reduzida (ICFEr), para o controle da frequência cardíaca (FC) e em mulheres com potencial de engravidar. ^5^ ^,^ ^37^ ^,^ ^164^ ^,^ ^495^ Outras classes de fármacos, como os alfabloqueadores, os simpatolíticos de ação central, os antagonistas da aldosterona e os vasodilatadores diretos, não foram amplamente estudadas em ensaios clínicos e associam-se a maior taxa de eventos adversos e devem ser usadas quando não há controle da PA em uso de combinações utilizando-se as principais classes de fármacos já mencionadas. ^37^ ^,^ ^164^ ^,^ ^495^ ^,^ ^503^ ^,^ ^504^

São características desejáveis do fármaco anti-hipertensivo:

• Ter demonstrado a capacidade de reduzir a morbidade e a mortalidade CV;
• Ser eficaz por via oral;
• Ser bem tolerado;
• Ser administrado preferencialmente em dose única diária;
• Poder ser usado em associação;
• Ter controle de qualidade em sua produção.
Além disso, recomenda-se:
• Utilizar por um período mínimo de quatro semanas, antes de modificações, salvo em situações especiais;
• Não utilizar medicamentos manipulados, pois não são submetidos a controle da farmacocinética e farmacovigilância;
• O paciente deverá ser orientado sobre a importância do uso contínuo da medicação anti-hipertensiva, da eventual necessidade de ajuste de doses, da troca ou da associação de medicamentos e ainda do eventual aparecimento de efeitos adversos;
• Não há evidências suficientes para a recomendação rotineira da administração noturna de fármacos anti-hipertensivos, exceto em condições especiais.

## 9.3. Esquemas Terapêuticos

**Figura 9.1:**
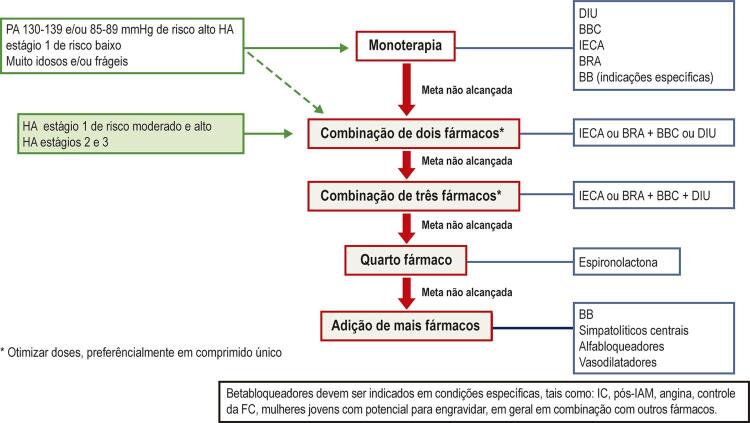
– Fluxograma de tratamento medicamentoso.

O tratamento com medicamentos pode ser iniciado com monoterapia ou com combinação de fármacos. Ênfase deve ser dada ao uso de combinação de fármacos como estratégia preferencial para a maioria dos pacientes hipertensos ( Figura 9.1 ).

### 9.3.1. Monoterapia

A monoterapia pode ser a estratégia anti-hipertensiva inicial para pacientes com HA estágio 1 com risco CV baixo ^37^ ^,^ ^164^ ^,^ ^495^ ou com PA 130-139/85-89 mmHg de risco CV alto ^307^ ou para indivíduos idosos e/ou frágeis ^4^ ( Figura 9.1 ). Nesses perfis de pacientes, a redução da PA desejada é pequena ou deve ser feita de maneira gradual, de modo a evitar eventos adversos. ^37^ ^,^ ^164^ ^,^ ^495^ ^,^ ^502^

O tratamento deve ser individualizado; e a escolha inicial do medicamento, basear-se nas características gerais desejáveis dos medicamentos anti-hipertensivos já descritas, nas particularidades individuais, na presença de doenças associadas e lesões de órgãos-alvo (LOA) e nas condições socioeconômicas. ^5^ ^,^ ^37^ ^,^ ^164^ ^,^ ^495^

As classes de anti-hipertensivos consideradas preferenciais ^5^ ^,^ ^37^ ^,^ ^164^ ^,^ ^495^ para o controle da PA em monoterapia inicial são:

• DIU tiazídicos ou similares; ^83^ ^,^ ^307^ ^,^ ^497^
• BCC; ^83^ ^,^ ^307^ ^,^ ^497^
• IECA; ^83^ ^,^ ^307^ ^,^ ^497^
• BRA. ^83^ ^,^ ^307^ ^,^ ^497^

Os BB podem ser considerados como fármaco inicial em situações específicas, ^5^ ^,^ ^37^ ^,^ ^83^ ^,^ ^164^ ^,^ ^307^ ^,^ ^495^ ^,^ ^497^ conforme já descrito anteriormente, e mais frequentemente são usados em associação a outros fármacos. A posologia pode ser ajustada na tentativa de alcançar a meta pressórica recomendada.

### 9.3.2. Combinação de Medicamentos

A combinação de fármacos é a estratégia terapêutica preferencial para a maioria dos hipertensos, independentemente do estágio da HA e do risco CV associado ^5^ ^,^ ^37^ ^,^ ^164^ ^,^ ^495^ ^,^ ^502^ ^-^ ^507^ ( Figura 9.1 ). O início do tratamento deve ser feito com combinação dupla de medicamentos que tenham mecanismos de ação distintos, sendo exceção a essa regra a associação de DIU tiazídicos com poupadores de potássio. Caso a meta pressórica não seja alcançada, ajustes de doses e/ou a combinação tripla de fármacos estarão indicados. Na sequência, mais fármacos deverão ser acrescentados até ser alcançado o controle da PA. ^502^ ^-^ ^504^

O racional para a associação de fármacos baseia-se no incremento do efeito anti-hipertensivo quando se atua em mecanismos fisiopatológicos distintos por ações sinérgicas e pela inibição da ativação dos mecanismos contrarregulatórios. ^502^ ^,^ ^503^ Além disso, a combinação de fármacos pode reduzir potencialmente a ocorrência de efeitos colaterais, pelo uso de menor dose de cada um dos fármacos envolvidos na combinação ou pela capacidade que um dos fármacos pode ter de antagonizar os efeitos adversos do outro. ^502^ ^,^ ^503^ São aspectos de interesse a maior adesão ao tratamento e a redução da inércia terapêutica. As combinações em doses fixas e em comprimido único são preferenciais por se associarem a maior adesão ao tratamento e, por consequência, melhores resultados clínicos. ^502^ ^,^ ^503^

O início do tratamento com combinação de fármacos em doses fixas associa-se à redução do risco de desfechos CV quando comparado com o tradicional início do tratamento com monoterapia, com mais rápido alcance da meta pressórica e com a proteção de órgãos-alvo e desfechos CV a longo prazo. ^502^ ^-^ ^507^

## 9.4. Características Gerais das Diferentes Classes de medicamentos Anti-Hipertensivos

A Quadro 9.2 apresenta, por classe terapêutica, a lista de medicamentos anti-hipertensivos disponíveis no Brazil.

**Quadro 9.2:** – Lista de medicamentos anti-hipertensivos disponíveis no Brazil

Classe	Classe e Medicamento	Dose diária habitual (mg)	Freq.*	Comentários e recomendações
**Diuréticos tiazídicos e similares**	Hidroclorotiazida	25-50	1	Doses mais elevadas dos tiazídicos e similares aumentam o efeito diurético sem adicionar ação anti-hipertensiva.
	Clortalidona	12,5-25	1	
	Indapamida	1,5	1	
**Diuréticos de alça**	Furosemida	20-240	1-3	Utilizada em insuficiência renal crônica (IRC), insuficiência cardíaca congestiva (ICC) e estados de retenção de líquidos (edema).
	Bumetanida	1-4	1-3	
**Diuréticos poupadores de potássio**	Espironolactona	25-100	1-2	Pode provocar hiperpotassemia, particularmente na IRC e quando associada a inibidores da ECA ou BRA.
	Amilorida	2,5-5	1	Disponível unicamente associada à hidroclorotiazida ou à clortalidona.
**Bloqueadores dos canais de cálcio (BCC) di-hidropiridínicos**	Anlodipino	2,5-10	1	Evite o uso em pacientes com insuficiência cardíaca com fração de ejeção reduzida. Pode provocar edema de membros inferiores relacionado com a dose utilizada.
	Felodipino	2,5-10	1	
	Nifedipino	10-60	1-3	
	Nitrendipino	10-30	1	
	Manidipino	10-30	1	
	Lacidipino	2-6	1	
	Lercanidipino	10-20	1	
	Levanlodipino	2,5-5	1	
**Bloqueadores dos canais de cálcio (BCC) não di-hidropiridínicos**	Verapamila	120-360	1-2	Evite o uso em pacientes com insuficiência cardíaca com fração de ejeção reduzida. Evite a associação com betabloqueadores e em pacientes com bradicardia.
	Diltiazem	80-240	1-2	
**Inibidores da enzima de conversão da angiotensina (IECA)**	Captoprila	25-150	2-3	Evite o uso em mulheres em idade fértil, pois há grande risco de malformações fetais e outras complicações na gestação. Contraindicada em associação a outros inibidores do sistema renina-angiotensina-aldosterona, exceto espironolactona na ICC. Risco de hiperpotassemia em pacientes com insuficiência renal ou que estejam recebendo suplementação de potássio.
	Enalaprila	5-40	1-2	
	Benazeprila	10-40	1-2	
	Lisinoprila	10-40	1	
	Fosinoprila	10-40	1	
	Ramiprila	2,5-20	1-2	
	Perindoprila	2,5-10	1	
**Bloqueadores dos receptores AT1 da Angiotensina II (BRA)**	Losartana	50-100	1-2	Mesmas recomendações feitas aos IECA.
	Valsartana	80-320	1	
	Irbesartana	150-300	1	
	Candesartana	8-32	1	
	Olmesartana	20-40	1	
	Telmisartana	20-80	1	
**Betabloqueadores (BB) não cardiosseletivos**	Propranolol	80-320	2-3	A retirada abrupta dos BB deve ser evitada, pois pode provocar taquicardia reflexa e mal-estar.
	Nadolol	40-160	1	
	Pindolol	10-60	1	Possui atividade simpatomimética intrínseca que proporciona menor bradicardia.
**Betabloqueadores cardiosseletivos**	Atenolol	50-100	1-2	
	Metoprolol	50-200	1	
	Bisoprolol	5-20	1	
	Nebivolol	2,5-10	1	Ação vasodilatadora via óxido nítrico.
	Carvedilol	12,5-50	1-2	Efeito alfabloqueador produz menor bradicardia.
**Simpatolíticos de ação central**	Metildopa	500-2.000	2	
	Clonidina	0,2-0,9	2	A retirada abrupta da clonidina pode provocar hipertensão rebote (crise hipertensiva) por liberação de catecolaminas na terminação sináptica.
	Rilmenidina	1-2	1-2	
**Alfabloqueadores**	Prazosina	1-20	2-3	Iniciar com dose baixa antes de deitar-se, pois pode provocar hipotensão ortostática. Aumentar progressivamente a cada 2 dias. Há outros alfabloqueadores no mercado indicados exclusivamente para a hipertrofia benigna de próstata (tansulosina, alfuzosina, silodosina).
	Doxazosina	1-16	1	
**Vasodilatadores diretos**	Hidralazina	50-200	2-3	Pode provocar retenção de sódio e água, hipervolemia e taquicardia reflexa. Deve ser usada em associação com diuréticos de alça. Síndrome lupus-like em dose alta.
**Inibidores diretos de renina**	Alisquireno	150-300	1	Mesmas recomendações feitas aos IECA e BRA.

### 9.4.1. Diuréticos (DIU)

O mecanismo da ação anti-hipertensiva dos DIU relaciona-se inicialmente a seus efeitos natriuréticos, com a diminuição do volume circulante e do volume extracelular. Após quatro a seis semanas, o volume circulante praticamente normaliza-se, e ocorre redução da resistência vascular periférica (RVP). Os DIU reduzem a PA e diminuem a morbidade e a mortalidade CV. ^508^ ^-^ ^510^ O efeito anti-hipertensivo não está diretamente ligado às doses utilizadas, porém os efeitos colaterais guardam relação com a dose e a potência da ação diurética. Deve-se dar preferência aos DIU tiazídicos (hidroclorotiazida) ou similares (clortalidona e indapamida) em doses baixas, pois são mais suaves e com maior tempo de ação, reservando-se os DIU de alça (furosemida e bumetanida) às condições clínicas com retenção de sódio e água, como a insuficiência renal (creatinina > 2,0 mg/dL ou o ritmo de filtração glomerular estimado ≤ 30 mL/min/1,73m ^2^ ) e situações de edema (IC, síndrome nefrítica). Os DIU poupadores de potássio (espironolactona e amilorida) costumam ser utilizados em associação aos tiazídicos ou DIU de alça. A espironolactona tem sido habitualmente utilizada como o quarto medicamento a ser associado aos pacientes com HA resistente e refratária. Esse aspecto é abordado de forma mais extensa no capítulo 16 dessas formas mais graves de HA.

Há maior potência diurética da clortalidona com relação à hidroclorotiazida, quando comparadas dose a dose, e sua meia-vida mais prolongada credenciou-a a ser indicada como DIU preferencial em pacientes com HA resistente ou refratária. Isso porque a retenção de sódio e volume é um mecanismo importante na resistência ao tratamento. ^504^ Já a indicação da clortalidona como DIU preferencial em função de promover maior redução de eventos CV é controversa, pois a metanálise e os estudos observacionais incluindo grande número de usuários são discordantes. ^495^ ^,^ ^511^ ^,^ ^512^ Por outro lado, como seria esperado pelo efeito diurético mais intenso, tais estudos registraram maior frequência de efeitos adversos da clortalidona, particularmente os distúrbios hidreletrolíticos e metabólicos. A indapamida, um tiazídico-símile, cujo uso tem crescido nos últimos anos, a exemplo da clortalidona, tem maior potência e efeito diurético mais prolongado e, como as medicações anteriores, apresenta comprovados efeito anti-hipertensivo e redução de eventos CV com bom perfil metabólico. ^513^ Dessa forma, não há dados definitivos que sustentem a preferência pela clortalidona para o tratamento anti-hipertensivo do indivíduo com função renal normal, mas se pode utilizá-la quando se deseja maior efeito diurético, em especial na HA resistente, pois é mais potente que a hidroclorotiazida.

#### 9.4.1.1. Efeitos Adversos dos Diuréticos

Os principais efeitos adversos dos DIU são fraqueza, cãibras, hipovolemia e disfunção erétil. A hipopotassemia é o efeito metabólico mais comum e, frequentemente acompanhada de hipomagnesemia, que podem provocar arritmias ventriculares, sobretudo a extrassistolia. A hipopotassemia também reduz a liberação de insulina, aumentando a intolerância à glicose e o risco de desenvolver diabetes melito tipo 2. O aumento do ácido úrico é um efeito quase universal dos DIU, podendo precipitar crises de gota nos indivíduos com predisposição.

O uso de DIU em doses baixas diminui o risco dos efeitos adversos, sem prejuízo da eficácia anti-hipertensiva, especialmente quando em associação a outras classes de medicamentos. A espironolactona pode causar ginecomastia e hiperpotassemia, sendo este distúrbio eletrolítico mais frequente em pacientes com déficit de função renal. Há relatos de que a indapamida pode ter um melhor perfil metabólico em comparação com a hidroclorotiazida. ^513^

## 9.4.2. Bloqueadores dos Canais de Cálcio (BCC)

Esta classe de medicamentos bloqueia os canais de cálcio na membrana das células musculares lisas das arteríolas, reduz a disponibilidade de cálcio no interior das células dificultando a contração muscular e, consequentemente, diminui a RVP por vasodilatação. ^514^ ^,^ ^515^

Os BCC são classificados em dois tipos básicos: os di-hidropiridínicos e os não di-hidropiridínicos. Os di-hidropiridínicos (anlodipino, nifedipino, felodipino, manidipino, levanlodipino, lercanidipino, lacidipino) exercem efeito vasodilatador predominante, com mínima interferência na FC e na função sistólica, sendo, por isso, mais frequentemente usados como medicamentos anti-hipertensivos.

Os BCC não di-hidropiridínicos, como as difenilalquilaminas (verapamila) e as benzotiazepinas (diltiazem), têm menor efeito vasodilatador e agem na musculatura e no sistema de condução cardíacos. Por isso, reduzem a FC, exercem efeitos antiarrítmicos e podem deprimir a função sistólica, principalmente nos pacientes que já tenham disfunção miocárdica, devendo ser evitados nessa condição.

Convém dar preferência aos BCC de ação prolongada para evitar oscilações indesejáveis na FC e na PA. São anti-hipertensivos eficazes e reduzem a morbidade e mortalidade CV. ^307^ ^,^ ^515^ ^-^ ^517^ Um estudo de desfecho reafirmou a eficácia, a tolerabilidade e a segurança do uso dessa classe de medicamentos no tratamento da HA de pacientes com DAC, ^518^ constituindo-se em alternativa aos BB quando esses não puderem ser utilizados, ou em associação, na angina refratária.

### 9.4.2.1. Efeitos Adversos dos Bloqueadores de Canal de Cálcio

O edema maleolar costuma ser o efeito colateral mais registrado e resulta da própria ação vasodilatadora (mais arterial que venosa), promovendo a transudação capilar. A cefaleia latejante e as tonturas são comuns. O rubor facial é mais comum com os BCC di-hidropiridínicos de ação rápida. A hipercromia do terço distal das pernas (dermatite ocre) e a hipertrofia gengival são efeitos adversos ocasionais.

Os efeitos adversos costumam ser dose-dependentes, podem causar intolerância aos BCC di-hidropiridínicos e, às vezes, resultam em resistência ao tratamento. Nesses casos, pode ser testada a utilização de BCC lipofílicos (manidipino, lercanidipino, lacidipino) ou o levanlodipino em baixas doses. A verapamila e o diltiazem podem agravar a IC, além de causar bradicardia e bloqueio atrioventricular. Observa-se a obstipação intestinal com a verapamila. ^516^

## 9.4.3. Inibidores da Enzima Conversora da Angiotensina (IECA)

São medicamentos anti-hipertensivos eficazes que têm como ação principal a inibição da enzima conversora de angiotensina I, responsável a um só tempo pela transformação de angiotensina I em angiotensina II (vasoconstritora) e pela redução da degradação da bradicinina (vasodilatadora). São eficazes no tratamento da HA, reduzindo a morbidade e mortalidade CV. ^307^ Mostram-se medicações comprovadamente úteis em muitas outras afecções CV, como em ICFEr e antirremodelamento cardíaco pós-IAM, além de possíveis propriedades antiateroscleróticas. Também retardam o declínio da função renal em pacientes com doença renal do diabetes ou de outras etiologias, especialmente na presença de albuminúria. ^519^

### 9.4.3.1. Efeitos Adversos dos Inibidores da Enzima Conversora da Angiotensina

Habitualmente, são bem tolerados pela maioria dos pacientes hipertensos, sendo a tosse seca seu principal efeito colateral, acometendo 5 a 20% dos usuários. O edema angioneurótico e a erupção cutânea ocorrem mais raramente. ^520^ Um fenômeno observado quando se administra a pacientes com insuficiência renal é a piora inicial da função renal, habitualmente discreta, provocada pela adaptação da hemodinâmica intraglomerular (vasodilatação da arteríola eferente e redução da pressão de filtração glomerular) que resulta em elevação da ureia e da creatinina séricas. ^521^ Entretanto, essa piora inicial da função renal é um mecanismo protetor, pois evita a hiperfiltração glomerular e reduz a progressão da doença renal crônica. ^522^ Se a perda da função renal for importante (> 30%), deve-se retirar o medicamento e investigar a possibilidade de estenose bilateral das artérias renais ou estenose de artéria renal em rim único funcionante.

Os IECA e outros bloqueadores do sistema renina-angiotensina-aldosterona (SRAA) podem provocar hiperpotassemia em pacientes com insuficiência renal, sobretudo nos diabéticos, e o uso é contraindicado na gravidez, pelo risco de complicações fetais. ^523^ ^,^ ^524^ Por isso, seu emprego deve ser cauteloso e frequentemente monitorado em adolescentes e mulheres em idade fértil.

## 9.4.4. Bloqueadores dos Receptores AT1 da Angiotensina II (BRA)

Os BRA antagonizam a ação da angiotensina II pelo bloqueio específico dos receptores AT1, responsáveis pelas ações próprias da angiotensina II (vasoconstrição, estímulo da proliferação celular e da liberação de aldosterona). No tratamento da HA, especialmente em populações de alto risco CV ou com comorbidades, proporcionam a redução da morbidade e da mortalidade CV e renal (doença renal do diabetes). ^525^ ^-^ ^531^

### 9.4.4.1. Efeitos Adversos dos Bloqueadores dos Receptores AT1 da Angiotensina II

São incomuns os efeitos adversos relacionados com os BRA, sendo o exantema raramente observado. De forma semelhante aos IECA, os BRA podem promover a redução inicial da filtração glomerular por vasodilatação das arteríolas eferentes, diminuindo a pressão de filtração glomerular, mas esse efeito é nefroprotetor a longo prazo. ^529^ ^-^ ^531^ Pelas mesmas razões dos IECA, podem causar hipercalemia, especialmente na presença de insuficiência renal, e são contraindicados na gravidez, devendo os mesmos cuidados ser tomados em mulheres em idade fértil.

## 9.4.5. Betabloqueadores (BB)

Os BB têm ações farmacológicas complexas. Promovem a diminuição inicial do débito cardíaco e da secreção de renina, com a readaptação dos barorreceptores e diminuição das catecolaminas nas sinapses nervosas. ^532^ ^,^ ^533^

Eles podem ser diferenciados em três categorias, de acordo com a seletividade para ligação aos receptores adrenérgicos: 1) não seletivos – bloqueiam tanto os receptores adrenérgicos beta-1, encontrados principalmente no miocárdio, quanto os beta-2, encontrados no músculo liso, nos pulmões, nos vasos sanguíneos e em outros órgãos (propranolol, nadolol e pindolol, este último apresentando atividade simpatomimética intrínseca, agindo como um agonista adrenérgico parcial e produzindo menos bradicardia); 2) cardiosseletivos – bloqueiam preferencialmente os receptores beta-1 adrenérgicos (atenolol, metoprolol, bisoprolol e nebivolol, que é o mais cardiosseletivo); e 3) com ação vasodilatadora – manifesta-se por antagonismo ao receptor alfa-1 periférico (carvedilol) e por produção de óxido nítrico (nebivolol). ^532^ ^-^ ^535^ O propranolol mostra-se também útil em pacientes com tremor essencial, prolapso de válvula mitral, síndromes hipercinéticas (hipertireoidismo e síndrome do pânico), cefaleia de origem vascular e hipertensão portal. ^532^ ^,^ ^533^

Uma metanálise ^536^ que incluiu mais de 130 mil pacientes com hipertensão primária comparou os BB a outras classes de medicamentos anti-hipertensivos, a placebo ou a sem tratamento. Observou-se que, comparativamente com os demais agentes anti-hipertensivos (DIU, BCC, IECA, BRA), os BB aumentam em 16% o risco de AVE. Quando comparados com placebo ou pacientes sem tratamento, os BB reduzem o AVE, mas apenas metade do que seria esperado para a redução pressórica observada. A metanálise ^534^ também identificou que o atenolol, comparado com os demais anti-hipertensivos, aumenta o risco de AVE em 26% e a mortalidade geral em 8%, ambos estatisticamente significantes. Essa é a principal razão para tal diretriz recomendar os BB como medicação anti-hipertensiva inicial apenas nos casos em que têm indicação específica.

### 9.4.5.1. Efeitos Adversos dos Betabloqueadores

Broncoespasmo, bradicardia, distúrbios da condução atrioventricular, vasoconstrição periférica, insônia, pesadelos, depressão, astenia e disfunção sexual. Os BB são contraindicados em pacientes com asma, doença pulmonar obstrutiva crônica (DPOC) e bloqueio atrioventricular de segundo e terceiro graus. Podem acarretar intolerância à glicose, induzir ao aparecimento de novos casos de diabetes melito, hipertrigliceridemia, elevação do colesterol-LDL e redução do colesterol-HDL. O impacto sobre o metabolismo da glicose é potencializado quando são utilizados em combinação com DIU. Os BB de terceira geração (carvedilol e nebivolol) têm impacto neutro ou até podem melhorar o metabolismo da glicose e lipídico, possivelmente pelo efeito vasodilatador, com diminuição da resistência à insulina e melhora da captação de glicose pelos tecidos periféricos. ^532^ ^,^ ^535^ Estudos com o nebivolol também têm encontrado menor disfunção sexual, possivelmente em decorrência do efeito sobre a síntese de óxido nítrico endotelial. ^532^ ^,^ ^535^

## 9.4.6. Simpatolíticos de Ação Central

Os alfa-agonistas de ação central agem por meio do estímulo dos receptores alfa-2 que estão envolvidos nos mecanismos simpatoinibitórios. ^537^ Os efeitos bem definidos dessa classe são: diminuição da atividade simpática e do reflexo dos barorreceptores, o que contribui para a bradicardia relativa e a hipotensão notada na posição ortostática; discreta diminuição na RVP e no débito cardíaco; redução nos níveis plasmáticos de renina; e retenção de fluidos. São representantes desse grupo: metildopa, clonidina e o inibidor dos receptores imidazolínicos rilmenidina. ^538^ ^,^ ^539^ A clonidina age também nos receptores alfa-2 pré-sinápticos, que impedem a liberação de norepinefrina. Esta se acumula na terminação nervosa e, ao ser interrompida abruptamente, pode provocar crise adrenérgica pela liberação descontrolada. ^537^ A rilmenidina, apesar de apresentar algum agonismo aos receptores alfa-2 centrais, exibe maior afinidade com os sítios de ligação dos receptores imidazolínicos do subtipo I, característica que confere menos efeitos indesejáveis que a clonidina. ^538^

Os medicamentos dessa classe não apresentam efeitos metabólicos e não interferem na resistência periférica à insulina nem no perfil lipídico. A metildopa encontra sua principal indicação na HA durante a gestação, pois é usada por um curto período da vida, com grande experiência clínica em sua utilização nesse tempo, e apresenta o melhor perfil de segurança para a gestante e para o feto. ^537^ ^,^ ^539^ O uso da clonidina pode ser favorável em situações de HA associada a síndrome das pernas inquietas, ^540^ retirada de opioides, ^541^ flushes da menopausa, ^542^ diarreia associada à neuropatia diabética ^543^ e hiperatividade simpática em pacientes com cirrose alcoólica. ^544^

### 9.4.6.1. Efeitos Adversos dos Simpatolíticos de Ação Central

A metildopa pode provocar reações autoimunes, como febre, anemia hemolítica, galactorreia e disfunção hepática, que, na maioria dos casos, desaparecem com a interrupção do uso. No desenvolvimento de uma reação adversa, a metildopa pode ser substituída por outro alfa-agonista central. ^539^

A clonidina apresenta risco do efeito rebote com a descontinuação, especialmente quando associada aos betabloqueadores, e pode ser perigosa em situações pré-operatórias. ^537^ A retirada gradual em duas a quatro semanas evita o efeito rebote. Os medicamentos dessa classe apresentam reações adversas decorrentes da ação central, como sonolência, sedação, boca seca, fadiga, hipotensão postural e disfunção erétil. ^537^ ^,^ ^539^

## 9.4.7. Alfabloqueadores

Os medicamentos dessa classe agem como antagonistas competitivos dos receptores alfa-1 pós-sinápticos, reduzindo a RVP sem mudanças no débito cardíaco. ^539^ Promovem maior redução pressórica quando na posição ortostática e na taquicardia reflexa. Por isso, é comum a hipotensão postural, comumente descrita na primeira dose. O efeito hipotensor mostra-se discreto como monoterapia, sendo a preferência pelo uso associado. Apresentam contribuição favorável e discreta no metabolismo lipídico e glicídico. ^539^ Os representantes dessa classe utilizados como medicamentos anti-hipertensivos são a doxazosina e a prazosina.

Uma ação coadjuvante benéfica dos bloqueadores alfa-1 é o relaxamento da musculatura do assoalho prostático, a qual favorece o esvaziamento da bexiga nos pacientes com hiperplasia prostática benigna (HPB). Por isso, alguns alfabloqueadores são também utilizados em homens com HPB, em particular a doxazosina, a tansulosina, a alfuzosina e a silodosina.

### 9.4.7.1. Efeitos Adversos dos Alfabloqueadores

Podem provocar hipotensão sintomática na primeira dose. O fenômeno de tolerância é frequente, necessitando aumento da dose para manter o efeito anti-hipertensivo (taquifilaxia). A incontinência urinária em mulheres pode ser causada pelo uso de alfabloqueadores. Há evidência de que os pacientes tratados com doxazosina têm maior risco de incidência de IC. ^539^

## 9.4.8. Vasodilatadores Diretos

Os medicamentos dessa classe, ativos por via oral, são a hidralazina e o minoxidil. Atuam diretamente, relaxando a musculatura lisa arterial, levando à redução da RVP. ^539^

### 9.4.8.1. Efeitos Adversos dos Vasodilatadores Diretores

Os efeitos colaterais da hidralazina são cefaleia, *flushing* , taquicardia reflexa e reação *lupus-like* (dose-dependente). ^539^ Seu uso pode também acarretar anorexia, náusea, vômito e diarreia. Os vasodilatadores podem provocar retenção de sódio e água, com o aumento do volume circulante e da taquicardia reflexa. Um efeito colateral frequente do minoxidil é o hirsutismo, que ocorre em aproximadamente 80% dos pacientes.

## 9.4.9. Inibidores Diretos da Renina

O alisquireno, único representante da classe disponível comercialmente, promove a inibição direta da ação da renina com a consequente diminuição da formação de angiotensina II. ^545^ Outras ações podem contribuir para a redução da PA e a proteção tissular, como redução da atividade plasmática de renina, ^545^ bloqueio de receptor celular próprio de renina/pró-renina ^546^ e diminuição da síntese intracelular de angiotensina II. ^547^

Estudos de eficácia anti-hipertensiva comprovam sua ação anti-hipertensiva, em monoterapia e em associação, de intensidade semelhante aos demais bloqueadores do SRAA e com aparente benefício adicional de redução da proteinúria em indivíduos com doença renal. ^548^ ^,^ ^549^ Não existem, contudo, evidências de seus benefícios sobre a morbidade e a mortalidade CV em hipertensos e pré-hipertensos. ^550^ ^,^ ^551^

### 9.4.9.1. Efeitos Adversos dos Inibidores Diretos da Renina

Apresentam boa tolerabilidade. *Rash* cutâneo, diarreia (especialmente com doses elevadas, acima de 300 mg/dia), aumento de creatinafosfoquinase e tosse podem ocorrer em menos de 1% dos usuários. Seu uso é contraindicado na gravidez pelas mesmas razões dos IECA e BRA.

## 9.5. Associações de fármacos anti-hipertensivos

A terapia anti-hipertensiva inicial com combinação de fármacos parece estar associada à redução do risco de desfechos CV quando comparada com o tradicional início do tratamento com monoterapia. ^552^ A combinação inicial de dois fármacos em comparação com a associação sequencial promove um controle mais rápido, podendo reduzir em até cinco vezes mais a PA, ^506^ com evidente impacto sobre LOA e desfechos CV a longo prazo. Uma metanálise demonstrou que a terapia de combinação em dose fixa com dois medicamentos aumentou em 24% a adesão quando comparada com a combinação em separado. ^553^ Há, contudo, poucos estudos especificamente destinados a avaliar combinações de fármacos sobre desfechos CV.

O estudo ACCOMPLISH ^554^ comparou as combinações benazeprila-hidroclorotiazida e benazeprila-anlodipino. A diferença na PA sistólica/diastólica entre os dois grupos, embora significante, foi apenas 0,9/1,1 mmHg menor no braço anlodipino. Observou-se uma redução no risco do desfecho primário composto de IAM não fatal, AVE, internação por angina instável, cirurgia de revascularização miocárdica e ressuscitação cardiopulmonar a favor do grupo benazeprila-anlodipino. A escolha da hidroclorotiazida neste estudo sofreu críticas devido à duração de seu efeito inferior a 24 horas, ao contrário do efeito prolongado do anlodipino. Todavia, uma publicação não encontrou diferenças significativas na PA de 24 horas entre os grupos. ^555^ Em pacientes com índice de massa corporal (IMC) > 30 kg/m ^2^ , não se observaram diferenças no desfecho primário entre os dois grupos. ^556^ Outra análise pré-especificada demonstrou redução adicional da progressão de doença renal com a associação benazeprila-anlodipino. ^557^

O estudo ASCOT-BPLA ^558^ comparou uma estratégia baseada em anlodipino, seguida da adição de perindoprila, se necessário, *versus* atenolol, seguido de bendroflumetiazida. Cerca de 78% dos pacientes em cada grupo usaram a terapia combinada para o controle tensional. Não houve diferenças no desfecho primário composto de IAM não fatal e DAC fatal, mas desfechos secundários como AVE, eventos coronarianos fatais, mortalidade CV e mortalidade por todas as causas foram significativamente menores no grupo baseado em anlodipino. O desenvolvimento de diabetes *de novo* foi 30% maior no grupo tratado com BB e tiazídico. O subestudo CAFE ^234^ revelou redução mais significativa da pressão aórtica central com a combinação anlodipino-perindoprila e atribuiu-se a este achado, ao menos parcialmente, a maior redução de desfechos secundários neste grupo.

No estudo multicêntrico VALUE, ^527^ pacientes de alto risco CV receberam tratamentos anti-hipertensivos baseados em valsartana ou anlodipino. Cerca de 25% dos pacientes em ambos os grupos necessitaram da adição de 12 a 25 mg de hidroclorotiazida para o controle da PA. Apesar da maior e mais precoce redução tensional no grupo anlodipino, o desfecho cardíaco primário combinado ao fim de quatro anos foi similar entre os grupos, assim como as taxas de IAM fatal e morte por todas as causas. Houve menos casos de IC com valsartana e menos AVE e IAM não fatal com anlodipino.

No HOPE-3, ^309^ primariamente dirigido ao estudo da ação de fármacos em pacientes pré-hipertensos em risco CV intermediário, o recurso de uma estratégia inicial baseada na combinação fixa candesartana-hidroclorotiazida promoveu a redução de 27% no risco do desfecho composto de morte CV, IAM não fatal e AVE nos indivíduos considerados como hipertensos estágio 1. Contudo, nenhum benefício foi observado em pré-hipertensos.

Os estudos PROGRESS, ^291^ que avaliou pacientes com doença cerebrovascular prévia; ADVANCE, ^559^ o qual analisou indivíduos com diabetes tipo 2; e HYVET, ^560^ que estudou pessoas com 80 ou mais anos de idade, utilizaram a intervenção baseada em indapamida e perindoprila, tendo demonstrado benefícios da associação de DIU a IECA na redução de problemas como AVE e disfunções vasculares; na composição de desfechos micro e macrovasculares; e em morte, AVE e IC, respectivamente.

A associação de BB a tiazídicos reduziu os desfechos CV, quando testada contra placebo em estudos antigos, envolvendo especialmente pacientes idosos, ^509^ ^,^ ^561^ ^,^ ^562^ mas foi inferior à associação de tiazídicos e losartana no estudo LIFE, ^526^ no qual ofereceu menor proteção contra AVE e favoreceu distúrbios no metabolismo da glicose. O uso de combinações fixas de tiazídicos com atenolol e outros BB deve ficar restrito às indicações específicas dessa classe de fármacos, ^5^ ^,^ ^164^ ^,^ ^495^ face à indução de potenciais distúrbios metabólicos dos DIU, como resistência a insulina, hiperglicemia, hiperuricemia e hipopotassemia.

A associação de bloqueadores de canais de cálcio di-hidropiridínicos com diuréticos tiazídicos pode revelar-se especialmente útil em idosos com hipertensão sistólica isolada ou em casos de contraindicação ao uso de bloqueadores do SRA ou de restrições a seu emprego, devido a riscos potenciais, como nas mulheres em idade fértil.

**Figura 9.2:**
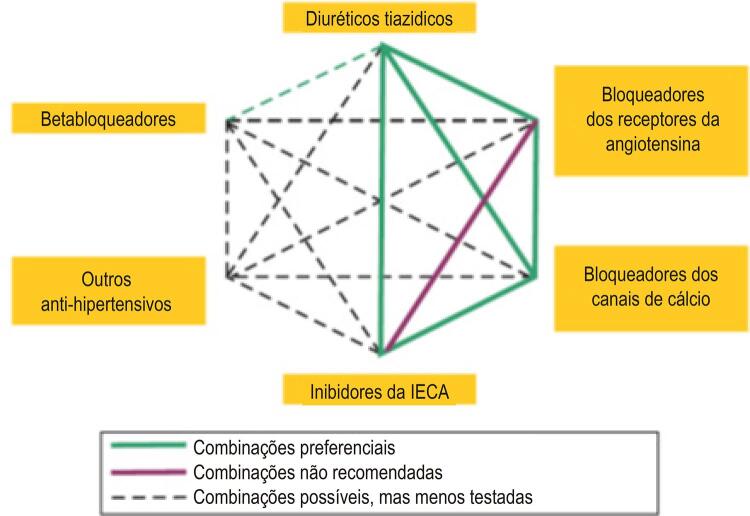
– Esquema preferencial de associações de medicamentos, de acordo com mecanismos de ação e sinergia.

O polígono da Figura 9.2 apresenta as combinações preferenciais (conectadas em traço verde), a contraindicada (em tracejado vermelho) e as combinações possíveis, porém menos estudadas (em linha pontilhada). ^1^ Em hipertensos em estágio 3 e em hipertensos resistentes, busca-se otimizar o tratamento tríplice com os fármacos preferenciais – IECA ou BRA, BCC di-hidropridínico e DIU tiazídico ou similar. ^37^ ^,^ ^503^ ^,^ ^504^ Um estudo avaliou a única combinação fixa tríplice disponível em nosso meio, valsartana-anlodipino-hidroclorotizida em pacientes com HA estágios 2 e 3, tendo demonstrado reduções médias de 39,7 mmHg na PA sistólica e de 24,7 mmHg na diastólica, significativamente maiores que combinações duplas envolvendo os mesmos fármacos. ^563^

Fonte: Malachias et al., 2016. ^164^

O não alcance da meta pressórica com o esquema tríplice exige a utilização de um quarto fármaco, cuja opção preferencial atual é a espironolactona. ^37^ ^,^ ^564^ ^-^ ^567^ BB, clonidina ^564^ e doxazosina ^567^ são opções de associações de 4 ^o^ ou 5 ^o^ fármacos, havendo ainda a possibilidade de adição de hidralazina ^164^ nos casos de intolerância a alguma das opções anti-hipertensivas anteriores e na HA resistente. ^503^ ^,^ ^504^ No estudo PATHWAY 2, ^567^ o uso de amilorida revelou-se tão eficaz quanto a espironolactona, oferecendo um tratamento substituto para a HA resistente. Contudo, o fármaco não está disponível em formulação industrializada em nosso meio. O estudo ReHOT ^564^ demonstrou eficácia da clonidina similar à da espironolactona como 4 ^o^ fármaco no controle de pacientes com HA resistente. No entanto, na análise da PA ambulatorial de 24 horas, a espironolactona revelou-se superior.

O tratamento com associações de dois bloqueadores do sistema renina-angiotensina, como IECA com BRA ou qualquer um dos dois com inibidor de renina, é contraindicado, pois promoveu o aumento de efeitos adversos, sem a redução de desfechos CV. ^568^ ^-^ ^569^

O Quadro 9.3 apresenta os principais ensaios clínicos em que foram utilizadas associações de fármacos anti-hipertensivos; e o Quadro 9.4 , os principais níveis de evidência e grau de recomendação sobre o tratamento farmacológico. A Figura 9.1 apresenta as etapas habituais da associação de fármacos para o controle da HA.

**Quadro 9.3:** – Estudos de combinações de fármacos no tratamento da hipertensão arterial

Estudo	Comparador	Perfil de pacientes	Diferença na PAS (mmHg)	Desfecho primário (redução % de risco relativo de eventos)	p
**Associação de diuréticos**					
PREVER ^179^ (amilorida + clortalidona)	Losartana	Hipertensos estágio 1	-2,2	Não avaliado. Maior redução da PAS com diuréticos sem aumento da glicemia	–
**Associação de inibidores da ECA e diuréticos**					
PROGRESS ^291^ (perindoprila + indapamida)	Placebo	AVE prévio ou AIT	-9	-28% AVE	<0,001
ADVANCE ^559^ (perindoprila + indapamida)	Placebo	Diabetes	-5,6	-9% eventos macro e microvasculares	0,04
HYVET ^560^ (indapamida + perindoprila)	Placebo	Hipertensos ≥80 anos	-15	-34% eventos CV	<0,001
**Associação de inibidores da ECA e bloqueadores de canais de cálcio (anlodipino)**					
ACCOMPLISH ^554^ (benazeprila + anlodipino)	Benazeprila + diurético	Hipertensos de alto risco	-0,9	-19,6% eventos CV compostos	<0,001
ASCOT BPLA ^558^ (anlodipino + perindoprila)	Betabloqueador + diurético	Hipertensos com 3 ou mais fatores de risco	-2,7	Diferença não significativa *	NS
**Associação de bloqueadores de receptores de angiotensina (olmesartana) e bloqueadores de canais de cálcio**					
COLM ^570^ (olmesartana + BCC)	Olmesartana + diurético	Japoneses hipertensos idosos com doença CV ou fatores de risco	0	Diferença não significativa	NS
**Associação de bloqueadores de receptores de angiotensina e diuréticos**					
LIFE ^526^ (losartana + diurético)	Betabloqueador +diurético	Hipertensos com HVE	-1,1	-13% eventos CV	0,02
**Associação de bloqueadores de canais de cálcio e diuréticos**					
FEVER ^571^ (felodipino + diurético)	Diurético + placebo	Hipertensos	-4	-34% eventos CV	<0,001
**Associação de bloqueadores de canais de cálcio e inibidores da ECA**					
SYST-EUR ^572^ (IECA + BRA + diurético)	Placebo	Idosos com HSI	-10	-31% eventos CV	<0,001
SYST-CHINA ^573^ (IECA + BRA + diurético)	Placebo	Idosos com HSI	-9	-37% eventos CV	<0,004
**Associação de betabloqueadores e diuréticos**					
Coope e Warrender ^574^ (atenolol e diurético)	Placebo	Hipertensos idosos	-18	-42% AVE	<0,003
SHEP ^509^ (clortalidona e atenolol)	Placebo	Hipertensos idosos	-13	-36% AVE	<0,001
STOP-H ^561^ (betabloqueador e diurético)	Placebo	Idosos com HSI	-23	-40% eventos CV	<0,004
STOP-H2 ^562^ (IECA e BCC)	Tratamento padrão (BB e diuretico)	Hipertensos idosos	0	Sem diferenças em eventos CV	–
**Combinação de dois antagonistas do sistema renina-angiotensina**					
ONTARGET ^568^ (telmisartana + ramipril)	IECA ou BRA	Pacientes de alto risco	–	Piora de desfechos renais	–
ALTITUDE ^569^ (alisquireno + BRA)	IECA ou BRA	Diabéticos de alto risco	–	Piora de desfechos renais	–
**Combinação em dose fixa de bloqueador de canal de cálcio, bloqueador dos receptores de angiotensina e diurético**					
Calhoun et al. ^563^ (BRA + diuretico+ BCC)	BRA + diurético ^a^ ou BCC ^b^ + diurético ou BRA + BCC ^c^	Hipertensos estágio 2 e 3	a: -7,6 b: -8,2 c: -6,2	Não avaliado	–
TRIUMPH ^575^ (telmisartana + anlodipino + clortalidona)	Tratamento habitual ao fim de 6 meses: Monoterapia em 65% e combinação de 2 fármacos em 29%	Hipertensos	-8,8	Não avaliado	–

Adaptado de ESC, 2018. ^37^ AIT: acidente isquêmico transitório; HSI: hipertensão sistólica isolada; HVE: hipertrofia ventricular esquerda; NS: não significativo. * diferenças significativas em vários desfechos secundários a favor do braço IECA + anlodipino; (a) BRA + diurético (b) BRA + diurético ou BCC (c) diurético ou BRA + BCC.

**Quadro 9.4:** – Tratamento medicamentoso: níveis de evidência e grau de recomendação

Associações de fármacos	NE	GR
As classes de fármacos preferenciais para o tratamento anti-hipertensivo são os DIU tiazídicos ou similares, BCC, IECA e BRA, por demonstrarem efetiva redução da PA e do risco de desfechos CV. Os BB devem ser considerados em situações clínicas específicas (DAC, IC e controle da FC)	A	I
O tratamento da HA pode ser iniciado com associação de duas classes de fármacos desde a HA estágio 1	B	I
O início do tratamento com combinação de dois fármacos deve ser feito com um IECA, ou BRA, associado a DIU tiazídico ou similar ou BCC	A	I
O tratamento da HA em pacientes de alto risco CV com a combinação de um IECA e um BCC di-hidropiridínico é preferencial à combinação de um IECA e um DIU tiazídico em pacientes não obesos	B	I
Quando não se atinge o controle da PA com combinação de dois fármacos, deve ser prescrita a combinação de três fármacos, habitualmente um IECA, ou BRA, associado a DIU tiazídico ou similar e BCC	A	I
Quando não se atinge o controle da PA com a combinação de três fármacos, a espironolactona deve ser acrescentada preferencialmente ao esquema terapêutico	B	I
O tratamento da HA com combinações fixas possibilita a maior adesão	B	IIa
O tratamento da HA com a combinação de dois antagonistas do sistema renina-angiotensina é contraindicado	A	III

**Table:** 

Mensagens principais
Os objetivos primordiais do tratamento anti-hipertensivo são a redução da pressão arterial e do risco de desfechos CV e mortalidade associados à hipertensão arterial.
O tratamento medicamentoso deve se associar às medidas não medicamentosas, e as classes de anti-hipertensivos preferenciais para o uso em monoterapia ou combinação são: diurético tiazídico ou similar, BCC, inibidores da enzima conversora de angiotensina, bloqueadores dos receptores de angiotensina e betabloqueadores (com indicações específicas).
A combinação de fármacos é a estratégia inicial recomendada para hipertensos estágio 1 de moderado e alto risco e estágios 2 e 3, preferencialmente em comprimido único. A monoterapia deve ser considerada para hipertensos estágio 1 de baixo risco e para muito idosos e/ou indivíduos frágeis.
O início do tratamento com combinação de dois fármacos deve ser feito com um IECA, ou BRA, associado a DIU tiazídico ou similar ou BCC. Em pacientes de alto risco não obesos, as combinações com BCC são as preferenciais.
Quando não se atinge o controle da PA com combinação de dois fármacos, deve ser prescrita a combinação de três fármacos, habitualmente um IECA, ou BRA, associado a DIU tiazídico ou similar e BCC; caso necessário, acrescentar espironolactona em seguida.

## 10. Hipertensão e Condições Clínicas Associadas

## 10.1. Diabetes Melito (DM)

A hipertensão arterial (HA) é um achado comum nos pacientes com DM, especialmente no tipo 2. As evidências mostram benefícios na redução da PA nessa população, com consequente redução de eventos macro e microvasculares e da mortalidade. Entre estes, estão: menor frequência de doença renal crônica (DRC), ^307^ ^,^ ^329^ retinopatia diabética e albuminúria. ^576^ Os dados contemporâneos mostram uma importante redução do risco cardiovascular (CV) em portadores de DM, muito embora ainda seja uma doença de elevada prevalência e importante fator de risco (FR) para as doenças cardiovasculares (DCV). ^577^ ^,^ ^578^ A relação DM e HA mostra-nos dados relevantes, como a presença de HA em 40% dos pacientes recém-diagnosticados com DM tipo 2 ^579^ e 50% dos portadores de DM tipo 2 desenvolverem HA antes do aparecimento de albuminúria. Por tratar-se de população de muito alto risco CV, a avaliação da excreção urinária de albumina, da creatinina, do fundo de olho e da presença de disautonomia deverá fazer parte da investigação. ^580^

### 10.1.1. Objetivos do Tratamento

Estudos clínicos aleatorizados mostraram benefícios no tratamento anti-hipertensivo dessa população, como menor incidência de acidente vascular encefálico (AVE), síndromes coronárias e DRC, quando alcançados níveis de PA inferiores a 140/90 mmHg. Em uma metanálise de 13 ensaios clínicos envolvendo pacientes com DM, uma pressão arterial sistólica (PAS) entre 131-135 mmHg diminuiu o risco de mortalidade por todas as causas em 13%, enquanto um controle mais intensivo da PAS ≤ 130 mmHg foi associado a maior redução de AVE. ^581^ Outra metanálise mostrou a redução significativa de mortalidade com média alcançada de PAS igual a 138 mm Hg e uma redução significativa de AVE com média alcançada de 122 mmHg. ^576^ Portanto, o controle da PA é importante na redução do risco de complicações micro e macrovasculares e deve ser mantido, se esses benefícios forem sustentados (GR: I, NE: A).

O tratamento não medicamentoso rigoroso impõe-se para todos os hipertensos diabéticos. Uma PA de consultório ≥ 140/90 mmHg indica a necessidade de tratamento medicamentoso. Todos os medicamentos utilizados na redução da PA podem ser usados em pacientes diabéticos. Evidências suportam o uso preferencial dos bloqueadores do SRAA, em particular em pacientes com lesões de órgãos-alvo (LOA). ^526^ ^,^ ^582^ ^-^ ^584^ O controle da PA frequentemente requer múltipla terapia, e um bloqueador dos canais de cálcio (BCC) e/ou um diurético (DIU) são classes recomendadas na associação aos bloqueadores do SRAA. ^506^ ^,^ ^585^ A associação de duas ou mais classes em única formulação galênica deve ser cogitada, levando-se em consideração ser essa uma população de alto risco em que a adesão ao tratamento é de fundamental importância.

## 10.2. Síndrome Metabólica (SM)

A SM caracteriza-se por uma associação de fatores de risco CV, incluindo obesidade central, elevação de glicemia e dislipidemia típica (elevação de triglicerídeos e níveis reduzidos de HDL colesterol) associada a elevação da PA. ^586^ ^-^ ^588^ Tais alterações metabólicas são encontradas em 30 a 40% dos portadores de HA, ^589^ e a presença da elevação da pressão arterial (PA) na SM eleva o risco CV global por ativar mecanismos que se associam a estados pro-trombótico e pro-inflamatório. ^590^ Assim, a investigação da presença das alterações metabólicas da SM e da obesidade central é imprescindível no paciente com HA. As mudanças no estilo de vida (MEV) visando a redução do peso, redução do consumo de sódio e controle da disglicemia e dislipidemia são recomendadas as todos os pacientes em tais condições. ^591^ O tratamento medicamentoso deve ser iniciado sempre quando PA for ≥ 140/90 mmHg, pois não existem evidências de benefícios do uso de medicamentos anti-hipertensivos na SM com PA normal. ^592^ A escolha dos medicamentos anti-hipertensivos deve priorizar as classes terapêuticas que possam melhorar ou, ao menos, não agravar a resistência insulínica, como os inibidores da enzima conversora da angiotensina (IECA), os bloqueadores dos receptores AT _1_ da angiotensina II (BRA) e os BCC. DIU e betabloqueadores (BB), com exceção dos vasodilatadores de ação direta, podem ser indicados como medicamentos adicionais. ^593^

## 10.3. Doença Arterial Coronária (DAC)

Evidências epidemiológicas robustas associam a HA à DAC. Dados do estudo INTERHEART demonstraram que 25% dos infartos (IAM) podem ser atribuídos à HA. ^286^ Um metanálise que avaliou o impacto da PA encontrou uma redução média de 17% de DAC para cada 10 mmHg de diminuição da PAS. ^85^

O tratamento da HA associada a DAC, que inclui pacientes pós-IAM, com angina de peito e revascularização miocárdica (RVM), deve contemplar preferencialmente BB, IECA ou BRA, além de estatinas e aspirina. Os BB são benéficos após IAM, especialmente no período até dois anos após o evento agudo. ^83^ Do mesmo modo, os IECA e os BRA testados nessa condição também demonstraram efeito benéfico. ^83^ ^,^ ^594^ ^,^ ^595^ Em pacientes com DAC crônica e múltiplos fatores de risco, como HA, os IECA associam-se à redução de desfechos clínicos relevantes ^596^ (NE: I; GR: A).

Com relação à meta de PA a ser atingida, deve-se considerar a possibilidade de efeito da curva J, demonstrado em diferentes estudos, ^597^ ^-^ ^600^ em que a redução excessiva, sobretudo da pressão arterial diastólica (PAD), talvez precipite eventos CV em pacientes com DAC obstrutiva. Assim, o objetivo é alcançar uma PAS < 130 mm Hg e PAD <80 mm Hg (GR: IIa; NE: B), devendo-se evitar níveis abaixo de 120/70 mmHg. ^601^ Medicamentos adicionais, como BCC e diuréticos tiazídicos, ^181^ podem ser utilizados para alcançar essas metas de PA.

## 10.4. Hipertensão na Doença Renal Crônica (DRC)

### 10.4.1. Paciente em Tratamento Conservador – Metas e Tratamento

Na DRC, a meta de PA a ser alcançada continua indefinida, e as evidências dependem de morbidades associadas. ^602^ Pacientes não diabéticos, tratados para alvos estritos (<130/80 mmHg), apresentaram retardo na progressão da doença, apenas em subgrupos com proteinúria, não sendo possível avaliar eventos CV ^603^ ^,^ ^604^ (GR: IIa; NE: A). Por outro lado, uma metanálise mostrou diminuição de mortalidade com o tratamento intensivo da HA. ^605^ Em diabéticos, observam-se redução da albuminúria, melhora da retinopatia e redução de AVE com metas estritas, porém sem efeitos em outros desfechos CV ^581^ ^,^ ^606^ ^,^ ^607^ (GR: IIa; NE: A).

Estudo em 9.361 pacientes não diabéticos, dos quais 2.646 com DRC, observou-se a redução de eventos CV da ordem de 25% no grupo tratado para a obtenção de PAS inferior a 120 mm Hg. Isso sugere um provável benefício dessa estratégia na proteção CV de pacientes com DRC ^86^ (GR: I; NE: A).

Com relação ao tratamento medicamentoso, os IECA ou os BRA estão indicados em hipertensos com ou sem albuminúria, sendo proscrita sua associação ^608^ (GR: I; NE: A). Os diuréticos tiazídicos ^83^ ou de alça, estes últimos no caso de DRC estágios G4-G5, e os BCC são eficazes, principalmente em associação a IECA ou BRA ^609^ (GR: I; NE: A). Os BB estão indicados na DAC e na insuficiência cardíaca (IC) associada. ^610^ Antagonistas dos receptores mineralocortocoides são medicamentos que diminuem a proteinúria, porém podem causar hiperpotassemia. Ensaios clínicos com novos antagonistas dessa classe são aguardados. ^611^

**Table:** 

A presente diretriz recomenda em adultos com HA e DRC, diabéticos ou não, meta de PA < 130/80 mmHg. Metas mais estritas podem ser almejadas em casos selecionados, sob estrita vigilância e após compartilhamento de riscos com o paciente.

### 10.4.2. Pacientes em Terapia Renal Substitutiva (TRS): Metas e Tratamento

O manejo da HA em pacientes sob tratamento dialítico é desafiador, especialmente devido à sobrecarga de volume que aumenta a variabilidade da PA, superestimando-a na pré-diálise e subestimando-a depois dela. ^612^ Não existem evidências acerca do nível ideal de PA em pacientes em diálise, mas os valores mais aceitos imediatamente antes e após a hemodiálise (HD) são ≤ 140/90 mmHg e ≤130/80 mmHg, respectivamente ^613^ ^,^ ^614^ (GR: IIa; NE: C). Nesses pacientes, existe uma associação (paradoxal), em U, entre a PAS medida na unidade de diálise e o risco de DCV, com valores superiores a 160 mmHg ou <110 mmHg, implicados no aumento da mortalidade ^614^ ^,^ ^615^ (GR: IIa; NE: B).

Nessa população, as medidas residenciais de PA são mais reprodutíveis, fornecem informações relevantes para decisões terapêuticas e associam-se a melhor controle da PA ^613^ (GR: IIa; NE: B). Assim, médias sistólicas obtidas por medidas domiciliares associam-se linearmente ao aumento do risco CV. ^615^ ^,^ ^616^ Além da hipervolemia, a rigidez arterial é causa importante de hipertensão sistólica em pacientes com DRC em estágio 5D. Esse fenótipo específico reflete a aceleração do processo arteriosclerótico e o envelhecimento vascular prematuro nesta população. ^616^ Outros mecanismos, como a apneia do sono, ^617^ a hiperatividade simpática ^618^ e o uso de eritropoetina, também devem ser considerados. ^616^

O tratamento da HA em pacientes em tratamento diálitico é eficaz em apenas 1/3 dos indivíduos, e ainda mais difícil de ser obtido, em especial devido à instabilidade hemodinâmica durante as sessões. Isso pode ocasionar hipo ou hipertensão intradialítica, piorando o prognóstico CV ^619^ (GR: IIa; NE: B).

O tratamento deve ser iniciado por medidas que visem à obtenção do “peso-seco”, como restrição hidrossalina e ultrafiltração na hemodiálise ^620^ (GR: IIa; NE: A). Não obstante, cerca de 60% dos pacientes sob tratamento dialítico necessitam de três ou mais anti-hipertensivos, em diversas combinações, para o controle da HA ^620^ (GR: IIa; NE: A). Nessa população, a hiperatividade do sistema nervoso simpático (SNS) tem papel importante na gênese da HA e na DCV. Em consonância, o bloqueio beta-adrenérgico foi superior à inibição da ECA na prevenção da morbidade CV e no controle da PA em pacientes com hipertrofia ventricular esquerda (HVE) sob tratamento hemodialítico. ^618^ Em indivíduos transplantados renais, os BCC e os BRA consistem na primeira opção, pois há evidências de que previnam a perda do enxerto ^621^ ^,^ ^622^ (GR: I; NE: A).

**Table:** 

A presente diretriz sugere a individualização do tratamento em TRS, respeitando-se as comorbidades, a farmacocinética e o efeito cardioprotetor do medicamento.

## 10.5. Insuficiência cardíaca (IC)

A HA tem papel fundamental na fisiopatologia da IC, levando ao aparecimento de HVE e à disfunção diastólica e sistólica do ventrículo esquerdo. ^623^ ^-^ ^625^ É o maior FR para seu desenvolvimento, geralmente antecedendo a síndrome clínica em vários anos. Na IC com fração de ejeção preservada (ICFEP), a HA é ainda mais frequente, sendo o FR mais encontrado, com prevalência de até 90%. ^626^

O diagnóstico precoce da HA e seu tratamento adequado podem reduzir significativamente o risco de desenvolvimento de IC, sobretudo em idosos. As estratégias medicamentosas para o controle da PA promovem reduções de aproximadamente 50% na incidência de IC em adultos e, na população com idade > 80 anos, diminuições de 64%. ^560^ ^,^ ^627^ ^-^ ^628^ O estudo SPRINT, com meta de redução de PAS mais intensa (< 120 mmHg) em população de alto risco CV, demonstrou uma redução de 27% em mortalidade total e de 38% no desenvolvimento de IC. ^86^ ^,^ ^629^ O impacto do tratamento anti-hipertensivo na prevenção de IC foi observado com várias classes de anti-hipertensivos, como os BB, os DIU, os BCC e os IECA. ^630^

A meta da PA no contexto de IC deve ser similar à preconizada para indivíduos com alto risco CV, ou seja < 130/80 mmHg. ^631^ O tratamento da HA no contexto de IC deve considerar o tipo de apresentação, isto é, com fração de ejeção preservada (ICFEP) ou reduzida (ICFER) ( Quadro 10.1 ). A primeira opção de terapêutica anti-hipertensiva na ICFER deve contemplar medicações que promovam bloqueio neuro-hormonal, em doses otimizadas e apresentem evidência científica comprovada de redução de mortalidade. ^632^ Tais medicações são os bloqueadores de SRAA, os BB e os antagonistas da aldosterona. A associação sacubitrila/valsartana é uma nova opção de tratamento com impacto em redução de mortalidade na ICFER, mas ainda sem evidências dos benefícios na HA. ^633^

**Quadro 10.1:** – Tratamento anti-hipertensivo nos pacientes com insuficiência cardíaca

Tratamento anti-hipertensivo em portadores de IC		
Recomendações	CR	NE
**Portadores de HA e IC (FER e FEP) devem ter meta pressórica <130/80 mmHg**	I	C
**Em ICFER, devem ser utilizados anti-hipertensivos com comprovada redução de mortalidade (BB/IECA/BRA/espironolactona)**	I	A
**Em ICFEP, todos os agentes anti-hipertensivos podem ser utilizados**	I	C
**Bloqueadores de canal de cálcio não di-hidropiridínicos e alfabloqueadores estão contraindicados na ICFER**	III	C

CR: classe de recomendação; ICFEP: insuficiência cardíaca com fração de ejeção preservada; ICFER: insuficiência cardíaca com fração de ejeção reduzida; NE: nível de evidência.

Se os níveis pressóricos ainda permanecerem elevados, apesar do bloqueio neuro-hormonal, podem ser associados a DIU; combinação dos vasodilatadores hidralazina e nitrato; ou BCC di-hidropiridínicos. Os BCC não di-hidropiridínicos, como diltiazem e verapamila, e os BB são contraindicados. ^631^

Devido à forte associação entre ICFEP e HA, o tratamento anti-hipertensivo está indicado na maioria dos pacientes. Os diuréticos devem ser utilizados para o controle da PA e de sintomas relacionados com a hipervolemia, mas os ensaios clínicos aleatorizados não demonstraram benefício na redução de mortalidade em pacientes com ICFEP. Apesar da falta de evidências de benefícios de medicações que promovam o bloqueio do SRAA e do SNS em reduzir mortalidade em ICFEP, tais substâncias devem ser utilizadas com o objetivo de controle pressórico. ^634^ ^-^ ^639^ As demais classes de anti-hipertensivos também podem ser utilizadas.

Existe uma curva em forma de J na relação entre níveis de PAS e mortalidade CV, principalmente na ICFER. ^640^ ^,^ ^641^ Dados derivados de ensaios clínicos, como *Copernicus, DigTrial, Val-HeFT e PARADIGM-HF* , ^318^ ^,^ ^642^ ^-^ ^644^ comprovaram a relação entre menores valores de PA e maiores taxas de mortalidade.

Na ICFEP, a associação entre níveis de PA e desfechos clínicos ainda permanece controversa. ^317^ ^,^ ^318^ Os níveis de PA nesse grupo de pacientes devem ser mantidos na meta de 120-129/70-79 mmHg.

10.6. Acidente Vascular Encefálico Hemorrágico (AVEH) e Acidente Vascular Encefálico Isquêmico (AVEI)

O acidente vascular encefálico isquêmico (AVEI) e o hemorrágico (AVEH) são as manifestações mais comuns da lesão vascular causada pela HA, sendo a principal causa de mortes e incapacidade nesses pacientes. ^292^ A prevenção de todos os tipos de AVE pode ser conseguida mantendo-se PA dentro das metas com os tratamentos instituídos (ver Capítulo 6) ^291^ ^,^ ^645^ ^-^ ^652^ (GR: IIa; NE: B).

### 10.6.1. Acidente Vascular Encefálico Hemorrágico

Se houver aumento da PA, podem ocorrer maior probabilidade de expansão do hematoma, aumento do risco de morte e pior prognóstico. ^653^ Estudos robustos sugerem que reduzir a PA (dentro de 6 h) para valores <140/90 mmHg não diminuiu eventos primários importantes, inclusive mortalidade. ^654^ Assim, não deve ser recomendada imediata redução da PA nos casos de AVEH, a menos que o valor da PAS esteja > 220 mmHg.

### 10.6.2. Acidente Vascular Encefálico Isquêmico

Os benefícios da redução da PA no AVE são menos claros, porém uma consideração deve ser feita para os pacientes candidatos a trombólise, pois, nesse caso, se a PA estiver com valores > 180/105 mmHg, poderá haver maior chance de hemorragias. ^655^ ^,^ ^656^ Uma metanálise sugere que a redução pressórica no AVEI poderia ter efeito neutro na mortalidade. ^657^ ^,^ ^658^

O Quadro 10.2 , modificado das Diretrizes ESC e ESH, ^37^ apresenta as recomendações para a meta e a terapêutica em pacientes com AVE agudo e doença cerebrovascular.

**Quadro 10.2:** – Recomendações para a meta e a terapêutica em pacientes com AVE agudo e doença cerebrovascular

Recomendações	CR	NE
**AVEH Não reduzir a PA em pacientes com PAS < 220 mmHg Se PAS for ≥ 220 mmHg, usar medicamentos IV, com PAS-alvo de 180 mmHg**	III IIa	A B
**AVEI Não é recomendado reduzir a PA, exceto em pacientes elegíveis para trombólise. Nesse caso, manter a PA<180/105 mmHg**	III IIa	B B
**Ataque isquêmico transitório Redução imediata**	I	A
**Meta: manter PAS entre 120-130 mmHg após o evento**	I	A
**Prevenção secundária Usar bloqueadores do sistema renina-angiotensina + bloqueadores dos canais de cálcio ou diuréticos tiazídicos**	I	A

AVEH: acidente vascular encefálico hemorrágico; AVEI: acidente vascular encefálico isquêmico; CR: classe de recomendação; NE: nível de evidência; PAS: pressão arterial sistólica.

**Table:** 

Mensagens principais
O controle da PA é importante na redução do risco de complicações micro e macrovasculares e deve ser mantido, se esses benefícios forem sustentados (GR: I, NE: A). Uma PA de consultório ≥ 140/90 mm Hg indica a necessidade de tratamento medicamentoso, preferencialmente com MEV e uso de bloqueadores do SRAA, podendo ser acrescentados DIU e BCC para alcançar a meta pressórica <140/90 mmHg.
O tratamento medicamentoso deve ser iniciado em SM sempre quando a PA estiver ≥ 140/90 mmHg, priorizando-se o emprego de anti-hipertensivos metabolicamente neutros ou que melhorem a sensibilidade à insulina, como os IECA, os BRA e os BCC.
No paciente portador de DRC, a meta de PA é <130/80 mmHg, podendo ser mais estrita em casos selecionados. Nos pacientes dialíticos, a obtenção do “peso-seco” é fundamental. Cerca de 60% dos pacientes sob tratamento dialítico necessitam de 3 ou mais anti-hipertensivos, em diversas combinações, para o controle da HA. No transplantado renal, os BCC e os BRA constituem-se a primeira opção terapêutica.
Não é recomendada a imediata redução da PA nos casos de AVEH, a menos que o valor da PAS seja ≥ 220 mm Hg, quando se deve utilizar medicamentos IV, com PAS-alvo de 180 mmHg.
Portadores de HA e IC (FER e FEP) devem ter meta pressórica <130/80 mm Hg. Em ICFER, convém realizar esse controle com BB, BRA e espironolactona, enquanto em ICFEP todos os anti-hipertensivos podem ser utilizados.
O tratamento da HA associada a DAC, que inclui pacientes pós-IAM, com angina de peito e revascularização miocárdica (RVM), deve contemplar preferencialmente os betabloqueadores, os IECA ou os BRA, além de estatinas e ácido acetilsalicílico com meta pressórica <130/80 mmHg.
As curvas J ou U são características observadas em pacientes portadores de DAC, devendo-se evitar níveis abaixo de 120/70 mm Hg. Em DRC, principalmente em dialíticos, PAS com valores superiores a 160 mmHg ou <110 mmHg são implicadas no aumento da mortalidade.

## 11. Hipertensão Arterial na Gestação

## 11.1 Epidemiologia

Os distúrbios hipertensivos da gestação constituem algumas das principais causas de mortalidade materna e perinatal em todo o mundo. A hipertensão crônica está presente em 0,9-1,5% das grávidas, e estima-se que a pré-eclâmpsia (PE) complica de 2 a 8% das gestações globalmente. ^659^ ^,^ ^660^ Tais síndromes são fatores causais relacionados com os óbitos materno e perinatal, podendo causar limitações definitivas à saúde materna e problemas graves decorrentes da prematuridade associada às indicações precoces de intervenção (prematuridade eletiva). ^660^ No Brazil, a PE é a principal causa de parto prematuro terapêutico, ^661^ e estimam-se uma incidência de 1,5% para PE e uma de 0,6% para eclâmpsia. ^662^ A prevalência da eclâmpsia em áreas mais desenvolvidas do país é de 0,2%, com mortalidade de 0,8%, ^661^ enquanto em regiões menos favorecidas essa prevalência sobe para 8,1%, com taxa de mortalidade materna correspondente a 22,0%. ^663^

## 11.2 Classificação da Hipertensão Arterial na Gestação

Recomendamos as definições e a classificação propostas pelo comitê do *American College of Obstetrician and Gynecologists* (ACOG), ^664^ ^,^ ^665^ apresentada no Quadro 11.1 (GR: IIb, NE B).

**Quadro 11.1:** – Definições e classificação dos distúrbios hipertensivos na Gestação

DEFINIÇÕES	
Hipertensão gestacional	PAS ≥140 mmHg e/ou PAD ≥90 mmHg, ou ambos, medida em duas ocasiões com pelo menos 4 horas de intervalo.
**Hipertensão gestacional grave**	PAS ≥160 mmHg e/ou PAD ≥110 mmHg, ou ambas, medidas em duas ocasiões com, pelo menos, 4 horas de intervalo.
**Proteinúria**	Proteinúria > 300 mg em 24 horas, razão proteinúria/creatinina urinária de 0,3 g/g de creatinina ou ++ em fitas reagentes (idealmente, quantificar).
**CLASSIFICAÇÃO**	
**Pré-eclâmpsia (com ou sem sinais de gravidade)**	PAS ≥140 mmHg ou PAD ≥90 mmHg, ou ambos, em geral após 20 semanas de gestação e frequentemente com proteinúria*. Na ausência de proteinúria, pode-se considerar o diagnóstico quando houver sinais de gravidade: trombocitopenia (< 100.000.109/L), creatinina > 1,1 mg/dL ou 2x creatinina basal, elevação de 2x das transaminases hepáticas, EAP, dor abdominal, sintomas visuais ou cefaleia, convulsões, sem outros diagnósticos alternativos.
**Hipertensão crônica**	HA diagnosticada ou presente antes da gestação ou antes das 20 semanas de gestação; ou HA diagnosticada pela primeira vez durante a gravidez e que não normaliza no período pós-parto.
**Hipertensão crônica com pré-eclâmpsia sobreposta**	Pré-eclâmpsia em mulher com história de HA antes da gravidez ou antes de 20 semanas de gestação.
**Hipertensão gestacional**	PAS ≥140 mmHg ou PAD ≥90 mmHg, ou ambas, em mulher com PA previamente normal, após 20 semanas de gestação, medida em duas ocasiões com, pelo menos, 4 horas de intervalo, sem proteinúria ou sinais de gravidade, e que retorna ao normal no período pós-parto.
**OUTRAS DEFINIÇÕES DIAGNÓSTICAS**	
**Eclâmpsia**	Convulsões tônico-clônicas na ausência de outras condições causais.
**Síndrome HELLP**	Hemólise, elevação de enzimas hepáticas e trombocitopenia.
**Síndrome de encefalopatia posterior reversível (PRES) e síndrome da vasoconstrição encefálico reversível**	Ocorre PRES com alteração de imagem pela presença de edema vasogênico e sinais hiperintensos na porção posterior do cérebro na RNM, além de estar associado a alterações visuais, convulsão, cefaleia e alteração de sensório. A síndrome da vasoconstrição cerebral reversível caracteriza-se por estreitamento de artérias do cérebro com cefaleia em trovoada ou sinais neurológicos focais.

PA: pressão arterial, PAS: pressão arterial sistólica, PAD: pressão arterial diastólica, HA: hipertensão arterial, EAP: edema agudo de pulmão; PRES: síndrome de encefalopatia posterior reversível

## 11.3 Conceito e Critérios Diagnósticos

Define-se hipertensão na gestação quando há pressão arterial sistólica (PAS) ≥ 140 mmHg e/ou pressão arterial diastólica (PAD) ≥ 90 mmHg, considerando-se o 5 ^o^ ruído de Korotkoff, confirmada por outra medida realizada com o intervalo de quatro horas. A medida deve ser obtida, preferencialmente, na posição sentada ou alternativamente em decúbito lateral esquerdo, com manguito de tamanho adequado. O método manual auscultatório é o padrão-ouro, pois dispositivos automatizados tendem a subestimar a pressão arterial (PA), sobretudo na pré-eclâmpsia grave. A monitorização ambulatorial da pressão arterial (MAPA) é superior à medida de consultório e a monitorização residencial da pressão arterial (MRPA) na não gestante. Na gestante, ajuda a evitar o tratamento desnecessário na hipertensão arterial (HA) do avental branco, sendo útil no manejo da hipertensão gestacional de alto risco e na detecção de HA mascarada. ^37^ O papel da MAPA e da MRPA ainda é controverso na gestação. A Sociedade Internacional para o Estudo da Hipertensão na Gestação (ISSHP) recomenda o uso da MAPA antes da vigésima semana de gestação e a MRPA para o seguimento. ^666^ O ponto de corte para diagnóstico de HA é ≥ 135/85 mmHg com a MAPA na vigília e ≥ 130/80 mmHg com a MRPA (Capítulo 3).

As definições e a classificação dos distúrbios hipertensivos na gestação estão no Quadro 11.1 .

## 11.4 Predição e prevenção de pré-eclâmpsia

Não é recomendada suplementação de cálcio (> 1g ao dia) para gestantes com ingestão normal de cálcio ^667^ (GR: III, NE: A), e, sim, naquelas com baixa ingestão de cálcio e em risco moderado e aumentado de pré-eclâmpsia ^667^ (GR: I, NE: A).

Baixas doses de ácido acetilsalicílico (AAS) (75 a 150 mg/dia) ao final do primeiro trimestre da gestação podem ser úteis na prevenção primária de pré-eclâmpsia em gestantes com risco moderado e aumentado para pré-eclâmpsia ^668^ ^-^ ^670^ (GR: I, NE: A). No entanto, o uso não é recomendado na ausência de risco ^669^ (GR: III, NE: A).

A predição de PE deve ser feita, preferencialmente, no 1 ^o^ trimestre por meio de avaliação que leva em conta a história clínica materna – fatores de risco – associada à ultrassonografia com Doppler que verifica se há resistência ao fluxo nas artérias uterinas. Existem também exames laboratoriais que avaliam a angiogênese como dosagem da endoglina solúvel, PIGF ( *placental endotelial growth factor* ), sFlt-1 ( *soluble fms-like tyrosine kinase receptor-1* ) e razão sFlt-1/PlGF que são promissores, mas ainda não estão disponíveis na prática clínica. ^666^

Nas pacientes consideradas de alto risco de PE, o uso de cálcio, na população com baixa ingesta (< 600 mg/dia), na dose 1,0 a 2g/dia, reduz risco de PE de forma efetiva. ^667^ O uso de AAS em baixas doses (75-150 mg/dia) para a prevenção de eclâmpsia encontra-se resumido no Quadro 11.2 . Preferencialmente, deve ser iniciado antes de 16 semanas, sem aumento de complicações maternas ou fetais, e encontra-se nas recomendações de diretrizes internacionais como o NICE 2019, ^671^ da OMS ^672^ e do Colégio Americano de Ginecologia e Obstetrícia (ACOG). ^664^ Um estudo ^670^ realizado com 1.776 pacientes, com dose de 150 mg de ácido acetilsalicílico comparada com placebo iniciado entre 11 e 14 semanas, mostrou um total de eventos (PE) de 1,6% no grupo do ácido acetilsalicílico contra 4,3% no grupo placebo (OR: 0,38, IC95% 0,2 a 0,74, p = 0,004). Isso ratifica o efeito protetor do ácido acetilsalicílico em gestantes de alto risco.

**Quadro 11.2:** – Recomendações para uso de AAS na prevenção de pré-eclâmpsia

Risco	Fator de risco	Recomendação
Alto	PE prévia com desfecho fetal adverso Gestação múltipla HA crônica DM tipo 1 ou 2 Doença renal Doença autoimune (LES/SAAF)	Recomenda-se ácido acetilsalicílico baixa dose para 1 ou mais desses critérios.
Moderado	Nuliparidade Obesidade (IMC ≥ 30 kg/m2) História familiar de PE (mãe ou irmã) Idade ≥ 35 anos História obstétrica prévia ruim (PIG, Prematuridade, baixo peso, mais de 10 anos de intervalo entre as gestações)	Considerar uso de ácido acetilsalicílico em baixa dose, se paciente apresentar mais de 1 fator de risco.

PE: pré-eclâmpsia; HA: hipertensão arterial; DM: diabetes melito; SAAF: síndrome do anticorpo antifosfolipídeo; LES: lúpus eritematoso sistêmico; IMC: índice de massa corporal; PIG: pequeno para a idade gestacional.

A Federação Internacional de Ginecologia e Obstetrícia (Figo) propõe em seu documento de rastreamento e prevenção de 2019 ^673^ o uso de uma calculadora de risco da *Fetal Medicine Foundation* para a indicação do ácido acetilsalicílico para a prevenção de pré-eclâmpsia. Ela é útil e pode ser acessada no *link* https://fetalmedicine.org/research/assess/preeclampsia/first-trimester.

## 11.5. Tratamento Não Medicamentoso

O tratamento não medicamentoso isoladamente não deve ser utilizado em situações de PAS acima de 160 mmHg persistente por mais de 15 minutos (GR: III, NE: B). Sugere-se o repouso relativo em hospital ou hospital-dia, com monitoramento, para pré-eclâmpsia (GR: IIa, NE: B). A internação hospitalar deve ser indicada em pacientes com hipertensão grave na gestação (GR: I, NE: B).

O tratamento não medicamentoso não deve ser utilizado isoladamente em situações de HA grave persistente por > 15 min ^674^ para evitar lesão neurológica irreversível, pois valores de PAS > 155 mmHg, em especial > 160 mmHg, são detectados imediatamente antes da ocorrência de AVE. ^675^ A hipertensão diastólica grave (> 105 ou 110 mmHg) não ocorre antes da maioria dos acidentes vasculares encefálicos (AVE) de gestantes com pré-eclâmpsia grave. ^676^

Uma revisão sistemática indica que não há diferença nos desfechos entre o repouso rígido e o repouso relativo em mulheres com hipertensão e proteinúria. ^677^ O repouso relativo no hospital, em comparação com a atividade rotineira em casa, reduz o risco de hipertensão grave. Não há indicação de repouso rotineiramente para a hipertensão na gestação. ^677^ Desfechos clínicos semelhantes entre atendimentos em unidades de cuidados pré-natais ou admissão hospitalar ocorrem para mães e recém-nascidos, mas as mulheres podem ter preferência pelo tratamento em hospital-dia. ^678^

Não há indicações específicas para os cuidados durante a internação, mas é necessário o monitoramento da condição materna e do feto. A PA deve ser medida periodicamente, com a avaliação diária do peso e da diurese, além de orientação sobre sinais premonitórios. Convém realizar exames laboratoriais (hemograma com plaquetas, enzimas hepáticas, ácido úrico, creatinina e proteinúria) uma a duas vezes por semana. O feto pode ser acompanhado pela avaliação do crescimento e dos movimentos fetais, do bem-estar fetal e do perfil biofísico fetal, além de ultrassonografia.

## 11.6. Conduta expectante

A conduta expectante não é recomendada a partir de 37 semanas de gestação em mulheres com hipertensão gestacional e pré-eclâmpsia ^679^ (GR: III, NE: B). Sugere-se conduta expectante entre 34 e 37semanas de gestação em mulheres estáveis, sem piora clínica ou hipertensão grave ^680^ (GR: IIa, NE: B).

O parto precoce em pacientes com PE pode estar associado à diminuição da mortalidade. ^681^ O momento ideal de parto antes de 32 a 34 semanas é um dilema, devido à incerteza no balanço entre a segurança da mãe (término da gestação) e a maturidade fetal (expectante). ^681^ Após 34 semanas, a sobrevida mostra-se alta, e o parto do bebê e da placenta é efetivo em países desenvolvidos. ^681^ Existe tendência de tentar postergar o parto até as 37 semanas, se for considerado seguro.

O estudo HYPTAT comparou a indução de parto com o monitoramento expectante para hipertensão grave ou PE sem sinais de gravidade (na época, considerada como leve) após 36 semanas. ^679^ Mulheres no grupo intervenção tiveram risco 29% menor de piora de desfecho materno, sem afetar o desfecho neonatal, e os autores sugerem que não se indica o tratamento expectante até as 37 semanas. ^679^ No HYPTAT-II, com gestações entre 34 a 37 semanas com HA não grave, a conduta expectante aumentou o risco materno com relação a parto imediato, mas diminuiu a ocorrência de síndrome do estresse respiratório neonatal. ^680^ Portanto, não se justifica o parto imediato; e considera-se o monitoramento expectante até que a situação clínica piore. Se o parto antes de 34 semanas for indicado e a condição clínica materna e fetal permitir a espera de 48 horas para a resolução, pode-se considerar o uso de corticosteroides para a maturação pulmonar fetal. ^682^

## 11.7. Tratamento medicamentoso

O tratamento medicamentoso urgente é indicado em caso de hipertensão grave ^674^ ^,^ ^675^ e na presença de sinais premonitórios (GR: I, NE: B). Não existe consenso com relação ao valor de PA para indicar quando o tratamento medicamentoso deve ser iniciado. Sugere-se iniciar o tratamento medicamentoso quando a PA estiver acima de 150-160/100-110 mmHg ^665^ ^,^ ^674^ ^,^ ^676^ com o objetivo de manter a PA entre 120-160/80-100 mmHg (GR: IIb, NE: B).

A escolha do medicamento anti-hipertensivo depende da experiência do médico assistente e da familiaridade com o medicamento escolhido e dos efeitos colaterais ^683^ (GR: IIb, NE: B). O uso de IECA, BRA e IDR é contraindicado na gestação (GR: I, NE: B), e atenolol e prazosin devem ser evitados se possível ^683^ ^,^ ^684^ (GR: IIa, NE: B).

Recomenda-se o uso de sulfato de magnésio para a prevenção e o tratamento da eclâmpsia (GR: I, NE: B). Para evitar mortes maternas, uma PAS > 160 mmHg deve ser considerada para o tratamento urgente, ^676^ valor este alinhando com as demais diretrizes internacionais e nacionais que utilizam o ponto de corte de 160 mmHg. ^164^

O início de medicação para gestantes com hipertensão abaixo de 160/110 mmHg é ainda controverso, exceto em gestantes com lesão de órgão-alvo (LOA). Uma revisão sistemática da Cochrane ^685^ mostrou que tratar HA leve a moderada não reduz significativamente a morbidade materna, fetal e do recém-nascido.

Entretanto, o estudo CHIPS, ^686^ ^,^ ^687^ que avaliou o tratamento agressivo (PAD até 85 mmHg) *versus* o tratamento não agressivo (PAD até 100 mmHg), em análise *post hoc* , mostrou um aumento importante de hipertensão grave e desfechos fetais desfavoráveis, como perda fetal, necessidade de UTI por mais de 48h, prematuridade e baixo peso. Desse modo, novos estudos estão avaliando se o início de medicação deveria ser a partir de 140/90 mmHg. ^665^

A meta do controle da HA, de acordo com a ACOG, deve ser a PAS > 120 e < 160 mmHg, e PAD > 80 e < 110mmhg, já que tanto a hipertensão quanto a hipotensão induzida podem prejudicar a perfusão placentária e, consequentemente, o crescimento fetal. O objetivo é prevenir a progressão de LOA, complicações cardíacas e cerebrovasculares, além das complicações obstétricas e fetais. ^665^

A terapia medicamentosa deve ser iniciada como monoterapia pelos medicamentos considerados de primeira linha (metildopa, nifedipina de ação prolongada ou betabloqueadores – exceto atenolol). Caso não ocorra o controle adequado, associar outro medicamento de primeira linha ou de segunda linha (diurético tiazídico, clonidina e hidralazina), evitando-se a combinação de medicamentos da mesma classe farmacológica. Inibidores da enzima conversora da angiotensina (ECA) e bloqueadores do receptor da angiotensina (BRA) são formalmente contraindicados na gestação, pelo risco de malformação fetal que pode levar a insuficiência renal intrauterina, assim como os antagonistas dos receptores de mineralocorticoides, pelo bloqueio hormonal, e o atenolol, pelo alto risco de restrição de crescimento fetal relacionado com seu uso. Evitar também o uso de diuréticos em pacientes com PE, pela possibilidade de piorar a depleção do volume intravascular. ^665^ ^,^ ^688^ Um estudo comparando a eficácia de labetalol, nifedipina de ação prolongada e metildopa para o manejo de HA grave na gestação sugeriu que todas as classes de medicamentos eram opções viáveis, porém a nifedipina de ação prolongada foi mais efetiva do que o labetalol e a metildopa. ^689^

O tratamento para emergência hipertensiva na gestante pode ser feito tanto com nifedipina (10 mg) por via oral quanto com hidralazina IV. Há uma tendência atual de preferência para a nifedipina 10 mg, que pode ser repetida na dose de 10 a 20 mg a cada 20 a 30 minutos VO, se não houver resposta após a terceira dose fazer hidralazina IV na dose de 5 mg a cada 20 a 30 minutos até a dose de 15 mg. ^674^

Em situações excepcionais, como a presença de edema agudo de pulmão e hipertensão grave e refratária, o uso de nitroprussiato de sódio pode ser considerado como a opção preferencial para o controle urgente da PA, ^690^ por no máximo 4 horas, pelo risco de impregnação fetal pelo cianeto.

Na HA do puerpério, nas pacientes não hipertensas crônicas, a HA costuma se resolver dentro da 1 ^a^ semana (5 a 6 dias), mas nesse período ainda há risco de complicações como AVE, edema agudo de pulmão (EAP) e insuficiência renal. O risco de eclâmpsia nesse período também existe, e entre 32% a 44% das mulheres podem ter convulsões no puerpério. ^691^

As puérperas podem receber qualquer medicação anti-hipertensiva, e o que limita a escolha é o aleitamento. Assim, devemos priorizar os anti-hipertensivos que passam em quantidade menor pelo leite materno.

O Quadro 11.3 mostra as principais medicações anti-hipertensivas disponíveis no Brazil e sua relação com o aleitamento. ^692^ ^-^ ^694^ O tratamento dos picos hipertensivos nas puérperas pode ser feito de forma convencional. Um estudo comparando captopril com a clonidina para controle de HA (PAS ≥ 180 mmHg e PAD ≥ 110 mmHg) verificou que não houve diferença significante entre eles, apenas uma tendência de a clonidina ser melhor no 3 ^o^ dia do puerpério. ^695^ Ambas foram consideradas efetivas e seguras para tratar puérperas com emergência hipertensiva. ^696^

**Quadro 11.3:** – Ações dos medicamentos no aleitamento materno

Fármacos	Excreção no leite	Aleitamento materno
**Nifedipina**	Pouca excreção	Permitido
**Anlodipino**	Estudos insuficientes	Incerto (aparentemente seguro)
**Diltiazem, verapamila**	Estudos insuficientes	Incerto (usar outro medicamento)
**Clonidina**	Excreção aumentada	Evitar
**IECA: enalaprila, captoprila**	Pouca excreção	Permitido sem restrição Permitido Permitido
**Lisinoprila, ramiprila**	Estudos insuficientes	Incerto (aparentemente seguro)
**BRA: losartana, valsartana candesartana, olmesartana, telmisartana**	Estudos insuficientes	Incerto (usar outro medicamento)
**Hidroclorotiazida**	Pouca excreção	Usar dose baixa (< 50 mg)
**Clortalidona**	Pouca excreção	Eliminação lenta no RN – evitar. Usar em dose baixa
**Furosemida**	Estudos insuficientes	Pode diminuir o leite. Somente utilizar se houver necessidade clínica
**Espironolactona**	Pouca excreção	Permitido
**Atenolol**	Excreção aumentada	Evitar
**Metoprolol**	Pouca excreção	Permitido
**Carvediolol**	Estudos insuficientes	Incerto
**Propranolol**	Pouca excreção	Permitido
**Bisoprolol**	Estudos insuficientes	Incerto (aparentemente seguro)
**Hidralazina**	Pouca excreção	Permitido
**Metildopa**	Pouca excreção	Permitido

## 11.8. Risco cardiovascular futuro

As síndromes hipertensivas da gravidez são um marcador de risco futuro (I: A), e as mulheres em tal situação devem ser abordadas de forma mais atenta e integral para a prevenção efetiva de doença cardiovascular e renal (GR: I, NE: C). Pacientes que desenvolvem qualquer tipo de HA durante a gestação, principalmente com desfechos ruins como prematuridade e PE precoce, têm aumento consistente do risco de DCV e renal no futuro. ^696^ ^-^ ^699^ O risco de ser hipertensa crônica aumenta 3 a 4 vezes; e o de AVE, 1,8 vezes. Do mesmo modo, o risco de doença arterial coronariana (DAC) dobra com o envelhecimento. ^696^ ^,^ ^697^

Em estudo prospectivo, ^698^ a HA na gestação foi associada a maior incidência de DAC (HR: 1,8; IC 95%: 1,3 a 2,6; p <0,001), IC (HR: 1,7; IC 95%: 1,04 a 2,60; p = 0,03), estenose aórtica (HR: 2,9; IC 95%: 1,5 a 5,4; p <0,001) e insuficiência mitral (HR: 5,0; IC 95%: 1,5 a 17,1; p = 0,01), o que mostra um aumento de risco CV global de 30%. Dados do registro norueguês mostram que PE está associada a risco 3 a 15 vezes maior de DRC estágio V. ^699^ As síndromes hipertensivas da gravidez são um marcador de risco futuro, e essas mulheres devem ser abordadas de forma mais atenta e integral para uma prevenção efetiva de DCV e renal.

**Table:** 

Mensagens principais	
Classificação	Pré-eclâmpsia, hipertensão crônica, pré-eclâmpsia sobreposta e hipertensão gestacional.
**Prevenção**	Cálcio e ácido acetilsalicílico em pacientes de alto risco.
**Tratamento não medicamentoso**	Não deve ser utilizado em situações de PAS acima de 160 mmHg persistente por mais de 15 minutos. Repouso relativo em hospital com monitoramento para pré-eclâmpsia. Internação em pacientes com hipertensão grave na gestação.
**Conduta expectante**	Sugere-se conduta expectante entre 34 e 37 semanas de gestação em mulheres estáveis, sem piora clínica ou hipertensão grave.
**Tratamento medicamentoso**	Indica-se tratamento medicamentoso urgente em caso de hipertensão grave e na presença de sinais premonitórios. Sugere-se iniciar tratamento medicamentoso quando a PA estiver acima de 150-160/100-110 mmHg, com o objetivo de manter a PA entre 120-160/80-100 mmHg. A escolha do medicamento anti-hipertensivo depende da experiência do médico assistente e da familiaridade com o fármaco escolhido e dos efeitos colaterais. Recomenda-se o uso de sulfato de magnésio para a prevenção e o tratamento da eclâmpsia.

## 12. Hipertensão Arterial na Criança e no Adolescente

## 12.1. Contexto Epidemiológico e Importância da Hipertensão em Pediatria

A prevalência da pressão arterial elevada (PAE) e da hipertensão arterial (HA) em crianças e adolescentes vem aumentando nos últimos anos. A prevalência atual de HA na idade pediátrica mostra-se de 3% a 5%, enquanto a de PAE é estimada entre 10-15%. ^700^ ^,^ ^701^ Na faixa etária de 7 a 12 anos, as prevalências de PAE e HA são de 4,7% e 1,9% respectivamente, ambas mais prevalentes entre os obesos. ^702^

O Estudo dos Riscos Cardiovasculares em Adolescentes (ERICA) avaliou 73.399 estudantes Brazileiros de 12 a 17 anos. A prevalência total de PAE no Brazil foi de 14,5%, taxa máxima de 29,3%, nos meninos entre 15-17 anos. A prevalência geral de HA foi de 9,6%, do mesmo modo, mais elevada entre os mais velhos. O estudo mostrou que 17,8% da prevalência de HA nos adolescentes pode ser atribuída à obesidade. ^703^

Na maioria das vezes, a HA pediátrica é assintomática, mas até 40% das crianças hipertensas apresentam hipertrofia ventricular esquerda (HVE) na ocasião de seu diagnóstico inicial. A HVE, apesar de oligossintomática na infância, é um precursor de arritmias e IC em adultos. ^704^ A HA pediátrica, também pode estar associada ao desenvolvimento de outras alterações de órgãos-alvo, como o aumento do espessamento da camada média-intimal da carótida, a redução da distensibilidade arterial e o estreitamento arteriolar na retina. Recomenda-se que a medida da PA seja realizada no mínimo anualmente, a partir dos 3 anos de idade. ^705^

## 12.2. Definição e Etiologia

Nas crianças e nos adolescentes, as definições de PAE e HA estão relacionadas com as curvas de distribuição normal da pressão arterial (PA) e sua distribuição por percentis. Utiliza-se para isso o método auscultatório, levando-se em consideração sexo, idade e percentil de altura da criança. ^164^ ^,^ ^706^ ^,^ ^707^

Em 2017, foram feitas modificações nos valores normativos da PA e nas recomendações para o diagnóstico e o manejo da HA na faixa etária pediátrica, ^5^ ^,^ ^705^ após a exclusão de crianças e adolescentes com sobrepeso e obesidade. O termo pré-hipertensão foi substituído por PAE. As novas recomendações, apresentadas adiante, redefinem o estadiamento da HA na infância e na adolescência, simplificam as recomendações para a avaliação preventiva da PA em consultas pediátricas de rotina, racionalizam o manejo inicial dos pacientes com diagnóstico de PAE ou HA e ampliam a importância da avaliação por MAPA no diagnóstico e no manejo da HA pediátrica.

O Quadro 12.1 apresenta a definição atualizada de PA normal, PA elevada, estágios 1 e 2 de HA na criança e no adolescente, de acordo com idade, sexo e percentil de altura. ^705^ Quanto mais jovem a criança e com maiores elevações da PA, maior a chance de se tratar de HA secundária. As nefropatias parenquimatosas e obstrutivas e a estenose de artéria renal são responsáveis por aproximadamente 60-90% desses casos, podendo acometer todas as faixas etárias. Distúrbios endócrinos, como o excesso de mineralocorticoide, corticoide ou catecolaminas; doenças da tireoide; e hipercalcemia – associada ao hiperparatireiodismo – correspondem a aproximadamente 5% dos casos. Diagnostica-se a coarctação da aorta em 2% dos casos, sendo 5% dos casos atribuíveis a outras etiologias, como efeitos adversos de fármacos vasoativos e imunossupressores, abuso de medicamentos esteroides, alterações no sistema nervoso central e aumento da pressão intracraniana. ^164^ ^,^ ^705^ ^-^ ^707^

**Quadro 12.1:** – Definição atualizada da pressão arterial de acordo com a faixa etária

Crianças de 1 a 13 anos de idade	Crianças com idade ≥13 anos
PA normal: < P90 para idade, sexo e altura	PA normal: < 120 / < 80 mm Hg
Pressão arterial elevada: PA ≥ P90 e < 95 percentil para idade, sexo e altura ou PA 120/80 mmHg mas < P95 (o que for menor)	Pressão arterial elevada: PA120 / <80 mmHg a PA129 / <80 mm Hg
Hipertensão estágio 1: PA ≥ P95 para idade, sexo e altura até < P95 + 12 mmHg ou PA entre 130/80 até 139/89mmHg (o que for menor)	Hipertensão estágio 1: PA 130/80 ou até 139/89 mm Hg
Hipertensão estágio 2: PA ≥ P95 + 12 mmHg para idade, sexo e altura ou PA ≥ 140/90 mmHg (o que for menor)	Hipertensão estágio 2: PA ≥ 140/90mmHg

PA: pressão arterial; P: percentil. Adaptado de Flynn et al., 2017. ^705^

**Quadro 12.2:**
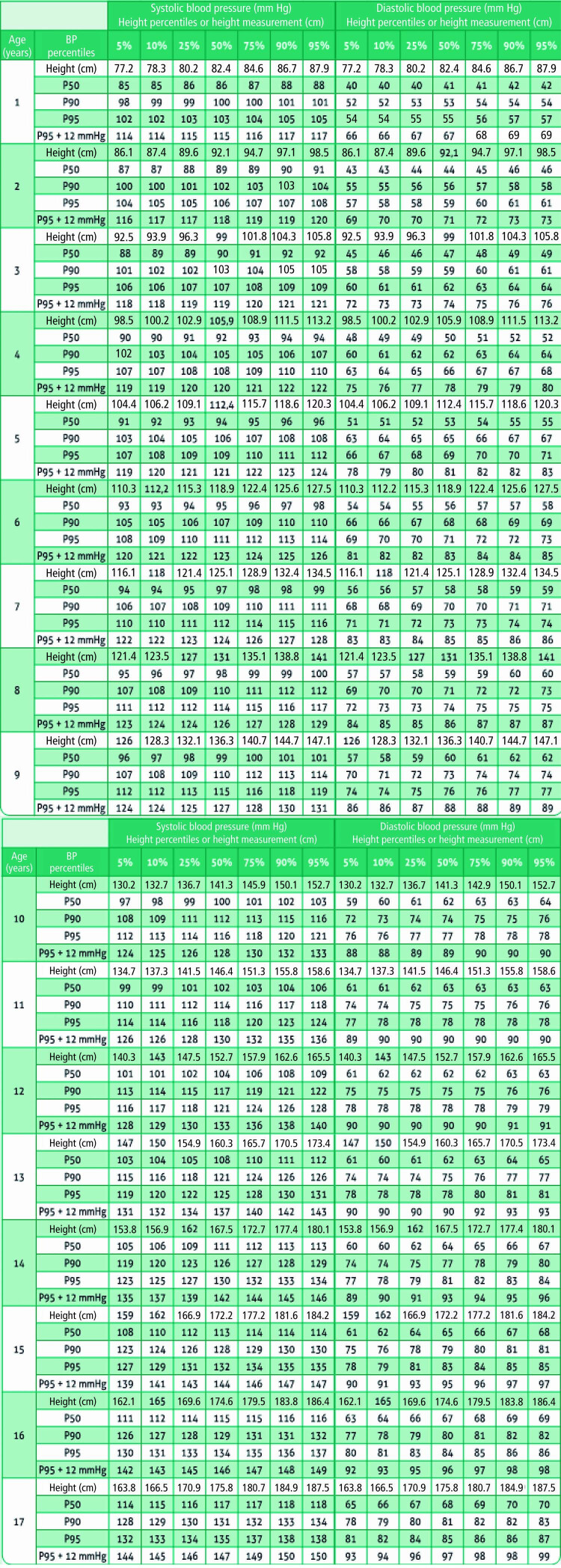
– Valores de pressão arterial para meninos de acordo com a idade e o percentil de estatura.

A HA primária parece ser a forma mais comum de HA no adolescente. É mais prevalente associada ao sobrepeso, à obesidade ou à história familiar de HA.

## 12.3. Diagnóstico

### 12.3.1. Métodos de Medida da PA

A medida da PA em crianças é recomendada em toda avaliação clínica. Convém ser medida anualmente em crianças e adolescentes ≥ 3 anos de idade, devendo-se respeitar as padronizações de medida estabelecidas. As crianças < 3 anos deverão ter a PA medida em situações específicas. A medida de PA deve ser repetida em todas as consultas no caso de condições de risco como obesidade, doença renal, coarctação de aorta, DM ou utilização crônica de medicamentos reconhecidamente associados a elevação de PA. A execução correta da técnica de medida de PA, segundo padronização previamente estabelecida, mostra-se condição obrigatória para a obtenção de valor fidedigno de medida e categorização correta da PA pediátrica. ^176^ ^,^ ^705^ Deve ser medida preferencialmente no braço direito, com o paciente deitado até os 3 anos de idade e, nas crianças maiores, em posição sentada com o braço apoiado ao nível do coração, utilizando o manguito correto. O comprimento da bolsa inflável deve ser de 80% a 100% da circunferência do braço (CB) e a largura de, pelo menos, 40% da CB. Convém a PA ser avaliada conforme descrito no Capítulo 3. Observar, pelo método auscultatório, se os sons de Korotkoff são ouvidos até 0 mmHg. Considera-se como PAD o ponto em que o som se abafa (Korotkoff fase IV). Na primeira consulta, a PA deve ser avaliada nos quatro membros e, quando realizada em membros inferiores (MMII), coloca-se o paciente em decúbito ventral, utilizando-se manguito de tamanho apropriado colocado na coxa e o estetoscópio sobre a artéria poplítea. A PAS em MMII costuma ser 10% a 20% mais elevada do que a PA medida na artéria braquial. ^164^ Os Quadros 12.2 e 12.3 apresentam os valores de PA normal, PA elevada e HA estágios 1 e 2, de acordo com sexo, idade e percentil de altura, adaptados da publicação de Flynn et al., 2017. ^705^ Alguns autores consideram o método oscilométrico como alternativa inicial de triagem adequada para crianças e adolescentes, o que justificaria a construção de quadros utilizando dispositivos validados. ^708^ ^,^ ^709^ No Brazil, Jardim et al. elaboraram uma curva de pressão arterial para adolescentes escolares de 12 a 17 anos sem excesso de peso, pelo método oscilométrico. ^710^

**Quadro 12.3:**
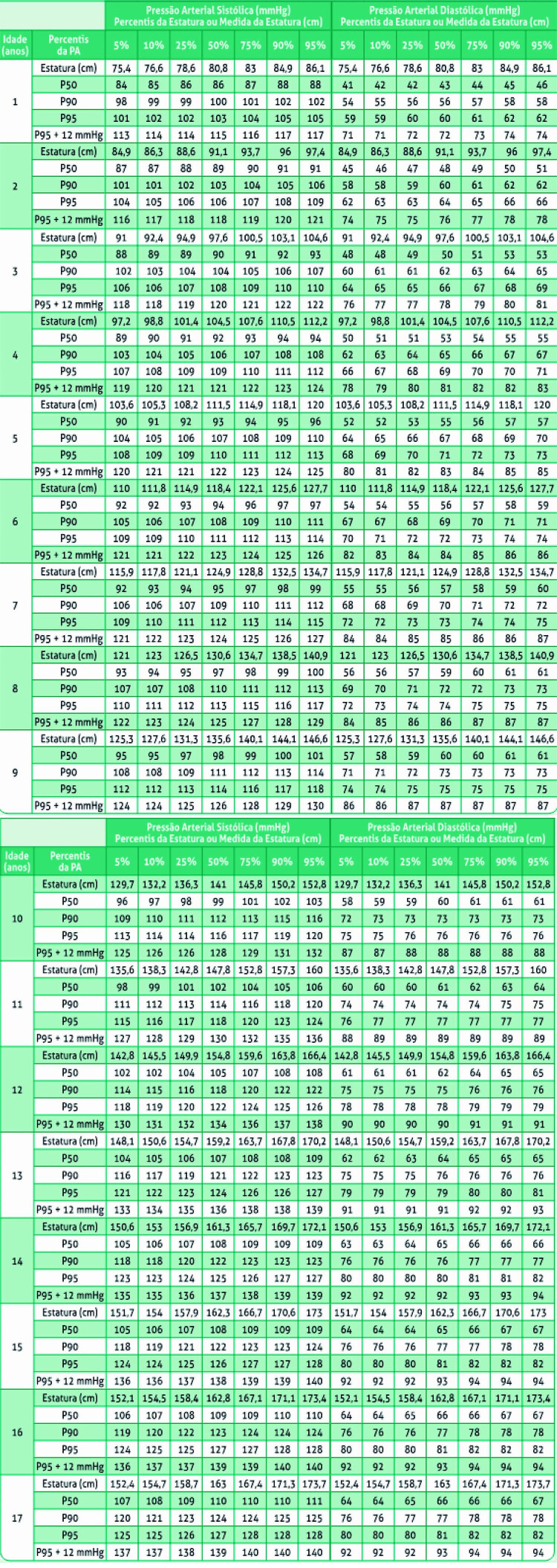
– Valores de pressão arterial para meninas de acordo com a idade e o percentil de estatura

Adaptado de Flynn et al, 2017. ^705^

As seguintes condições clínicas de risco determinam a necessidade de medida rotineira de PA em crianças < 3 anos de idade: prematuridade, muito baixo peso ao nascer, restrição de crescimento intrauterino, antecedente de internação em unidade de terapia intensiva (UTI) neonatal ou de cateterização umbilical pós-natal, cardiopatias congênitas operadas ou não, infecção urinária de repetição, hematúria ou proteinúria, nefrouropatias, transplante de órgãos sólidos, doença oncológica ou transplante de medula óssea, uso crônico de medicamentos com reconhecido efeito de elevação de PA, doenças sistêmicas associadas a HA (neurofibromatose, esclerose tuberosa, anemia falciforme, entre outras) e evidência de hipertensão intracraniana. ^705^

Com relação à medida de PA do recém-nascido (RN), sugere-se que seja avaliada por metodologia oscilométrica. Uma compilação de valores normativos para PA no período neonatal está disponível para neonatos a partir de 15 dias de vida e idade gestacional pós-natal entre 26 a 44 semanas ( Quadro 12.4 ). ^711^ Dispositivos oscilométricos, devidamente validados para a faixa etária pediátrica, podem ser usados para a avaliação inicial da PA. Se houver suspeita de PA elevada com base em leituras oscilométricas, medidas confirmatórias devem ser realizadas por metodologia auscultatória. O diagnóstico de HA pediátrica baseia-se na confirmação de valores de PA ≥ percentil 95 em três visitas diferentes por metodologia auscultatória. ^705^ O Quadro 12.5 oferece uma opção simplificada de valores de PA que sugerem a necessidade de avaliação clínica adicional. ^705^ ^,^ ^711^

**Quadro 12.4:** – Valores estimados de pressão arterial após duas semanas de vida em neonatos de 26 a 44 semanas de idade pós-concepção

Idade pós-concepção	Percentil 50	Percentil 95	Percentil 99
**44 semanas**			
**PAS**	88	105	110
**PAD**	50	68	73
**PAM**	63	80	85
**42 semanas**			
**PAS**	85	98	102
**PAD**	50	65	70
**PAM**	62	76	81
**40 semanas**			
**PAS**	80	95	100
**PAD**	50	65	70
**PAM**	60	75	80
**38 semanas**			
**PAS**	77	92	97
**PAD**	50	65	70
**PAM**	59	74	79
**36 semanas**			
**PAS**	72	87	92
**PAD**	50	65	70
**PAM**	57	72	71
**34 semanas**			
**PAS**	70	85	90
**PAD**	40	55	60
**PAM**	50	65	70
**32 semanas**			
**PAS**	68	83	88
**PAD**	40	55	60
**PAM**	50	65	70
**30 semanas**			
**PAS**	65	80	85
**PAD**	40	55	60
**PAM**	48	65	68
**28 semanas**			
**PAS**	60	75	80
**PAD**	38	50	54
**PAM**	45	58	63
**26 semanas**			
**PAS**	55	72	77
**PAD**	30	50	56
**PAM**	38	57	63

PAS: pressão arterial sistólica; PAD: pressão arterial diastólica; PAM: pressão arterial média. Adaptado de Dionne et al., 2012. ^711^

Adaptado de Flynn et al., 2017. ^705^

**Quadro 12.5:** – Valores de PA considerados como sinal de alerta para a necessidade de avaliação clínica adicional segundo a idade cronológica

Idade	Sexo masculino		Sexo feminino	
	PAS	PAD	PAS	PAD
**1**	98	52	98	54
**2**	100	55	101	58
**3**	101	58	102	60
**4**	102	60	103	62
**5**	103	63	104	64
**6**	105	66	105	67
**7**	106	68	106	68
**8**	107	69	107	69
**9**	107	70	108	71
**10**	108	72	109	72
**11**	110	74	111	74
**12**	113	75	114	75
**≥13**	120	80	120	80

PAS: pressão arterial sistólica; PAD: pressão arterial diastólica. Adaptado de Flynn et al., 2017. ^705^

## 12.4. Anamnese

Devem ser detalhados dados de nascimento, crescimento e desenvolvimento, antecedentes pessoais de doenças renais, urológicas, endócrinas, cardíacas e neurológicas e hábitos de vida, bem como o uso de medicamentos e outra substâncias que possam alterar a PA. A investigação de antecedentes familiares para HA, doenças renais e outros FRCV é fundamental. Crianças ≥ 6 anos de idade não necessitam ser submetidas a rastreamento extensivo para causas secundárias de HA, se apresentarem antecedentes familiares positivos para HA, sobrepeso ou obesidade e/ou não apresentarem anamnese ou exame físico sugestivos de causas secundárias de HA. ^705^ ^,^ ^712^

## 12.5. Exame Físico

Ao exame físico, deve-se calcular o índice de massa corporal (IMC) ^713^ e procurar indícios de HA secundária (ver Capítulo 15). ^714^

## 12.6. Exames complementares

Os exames laboratoriais e de imagem solicitados têm como objetivo definir a etiologia (primária ou secundária) e detectar lesão de órgãos-alvo (LOA) e fatores de risco cardiovasculares (FRCV) associados à HA (Quadros 12.6 e 12.7 ). ^705^ ^,^ ^715^ A avaliação de órgãos-alvo deve ser realizada em cada criança e adolescente com HA classificada nos estágios 1 e 2. O estudo do sono, por meio da polissonografia, está indicado para crianças e adolescentes com transtorno de sono detectado pela anamnese. ^705^

**Quadro 12.6:** – Investigação inicial de crianças e adolescentes com HA

Hemograma completo
Função renal e eletrólitos (incluindo cálcio, fósforo e magnésio)
Perfil lipídico
Acido úrico sérico
Glicemia de jejum
Exame de urina tipo 1 e urocultura
Fundoscopia
Radiografia de tórax
Ecodopplercardiograma
US renal e de vias urinárias com Doppler de artérias renais
US: ultrassonografia.

## 12.7. Monitorização Ambulatorial de Pressão Arterial (MAPA)

A MAPA deve ser realizada para a confirmação da HA em crianças e adolescentes com medidas de PA de consultórios compatíveis com PA elevada por, pelo menos, um ano ou com valores de PA compatíveis com HA estágio 1 em três consultas ambulatoriais. ^705^ Deve também ser considerada como parte da investigação de rotina nos casos de HA secundária, DRC, diabetes melito (DM), apneia obstrutiva do sono (AOS), obesidade, pós-operatório de coarctação de aorta, prematuridade, transplante de órgãos sólidos e HARf. O procedimento deve seguir técnicas padronizadas e utilizar monitores validados para uso pediátrico e dados normativos pediátricos. ^716^

As 6 ^as^ diretrizes de MAPA e as 4 ^as^ diretrizes de monitorização residencial da pressão arterial (MRPA) trazem as informações necessárias para a análise da MAPA em crianças e adolescentes. ^186^ A categorização da PA pela MAPA leva em conta, além da medida de PA, os parâmetros da chamada carga pressórica e a presença de *dipping* de PA durante o sono, conforme demonstrado no Quadro 12.7 . ^713^

**Quadro 12.7:** – Sugestão para estadiamento da PA ambulatorial em crianças e adolescentes

Classificação	PA no consultório	PAS/PAD ambulatorial	Carga sistólica/diastólica
PA normal	< P90	< P95	<25%
Hipertensão avental branco	≥ P95	< P95	<25%
PA elevada	≥ P90 ou > 120/80mmHg	< P 95	≥ 25%
Hipertensão mascarada	< P95	< P95	≥ 25%
Hipertensão ambulatorial	> P95	> P95 (> P90 HA 2aria)	25 a 50%
Hipertensão ambulatorial grave	>P95	> P95	> 50%

PA: pressão arterial; P: percentil. Adaptado de Flynn et al., 2017. ^705^

## 12.8. Aspectos Terapêuticos

Os principais objetivos do tratamento da HA na infância e na adolescência são evitar a LOA e a manutenção da HA na vida adulta. Seu planejamento depende da etiologia da HA, do risco cardiovascular (CV) associado ou não a outras doenças de base e da presença de LOA (NE: C). ^705^

## 12.9. Terapêutica Não Farmacológica

A terapêutica não farmacológica deve ser introduzida para todos os pacientes pediátricos com valores de PA acima do percentil 90 ou PA < 130/80 (≥ 13 anos de idade) (NE: C). ^705^ Inclui redução de peso, exercício físico, intervenção dietética e controle de estresse. A associação entre estas quatro medidas tem efeito potencializado, quando comparado com o efeito individual de cada intervenção. ^705^

A redução de peso apresenta bons resultados, sendo que a utilização da abordagem motivacional parece ser o método mais eficaz no controle da associação entre obesidade e HA na infância (NE: C). ^717^ Toda criança/adolescente deve realizar, pelo menos, 300 min/semana de atividades físicas moderadas/vigorosas para a manutenção a saúde. Além disso, deve-se limitar o tempo de comportamento sedentário (tempo sentado ou deitado) da população dessa faixa etária. O exercício físico estruturado apresenta maior impacto sobre os valores de PAS. ^717^ Recomenda-se a execução de exercícios aeróbicos (30-60 minutos) em intensidade moderada, pelo menos, três vezes por semana, se possível diariamente. ^705^ O treinamento resistido pode ser realizado em complemento. Esportes competitivos não são recomendados para pacientes com HA estágio 2 ainda não controlada (NE:C). ^718^

A intervenção dietética deve incluir a restrição do consumo de sódio, assim como pode incluir a suplementação de potássio e cálcio. Estudos observacionais demonstram o efeito positivo de polifenóis do azeite de oliva. ^705^ ^,^ ^719^ Recomenda-se a dieta DASH, com ênfase no aumento de alimentos de origem vegetal e na redução da ingestão de açúcares e doces. Tal medida é especialmente eficaz na HA associada à obesidade (NE: B). ^719^ ^-^ ^721^ Recomenda-se ainda o controle de estresse nessa faixa etária, podendo ser utilizadas diferentes formas de meditação, *mindfulness* ou ioga (NE: C). ^705^

## 12.10. Terapêutica Farmacológica

A terapêutica farmacológica deve ser iniciada para casos pediátricos com HA sintomática, secundária a DRC ou DM, presença de LOA, HA estágio 2 sem causa modificável aparente e HA persistente não responsiva à mudança de estilo de vida (MEV) (NE: B). ^705^ O tratamento tem como alvo a redução da PA abaixo do percentil 90 (NE: C). Inicia-se com um agente anti-hipertensivo em sua menor dose e aumenta-se a dose a cada duas a quatro semanas até o alvo. Caso não seja suficientemente efetivo, adicionam-se outras classes de medicamentos em sequência. Como muitas classes de medicamentos determinam retenção de sal e água, recomenda-se considerar a associação de tiazídico como segundo medicamento. Os eventos adversos associados à utilização dos agentes anti-hipertensivos em crianças e adolescentes têm-se mostrado, em geral, de grau menor (NE: B). ^705^ ^,^ ^722^

A utilização de todas as classes de anti-hipertensivos parece segura, pelo menos, a curto prazo. ^722^ No entanto, diretrizes internacionais recentes recomendam a utilização preferencial de inibidor da enzima conversora de angiotensina (IECA), BRA, BCC de ação longa ou DIU tiazídicos como fármacos de primeira linha. No caso de necessidade de um terceiro anti-hipertensivo, recomendam-se alfabloqueadores, BB, simpatolíticos de ação central ou poupadores de potássio (NE: C). ^705^ ^,^ ^722^

Sugere-se que, na HA secundária, a escolha do anti-hipertensivo seja feita em consonância com o princípio fisiopatológico envolvido, considerando as comorbidades presentes em cada caso. ^719^ ^-^ ^726^ Em pacientes com HA resistente, deve-se instituir maior redução da ingestão de sódio, além de pesquisa detalhada da utilização de substâncias ou alimentos que causam HA, detalhamento da adesão ao esquema terapêutico e otimização máxima deste esquema (NE: C). ^705^

Em caso de não resposta a monoterapia por mais de 6 meses, considera-se o encaminhamento ao especialista em HA na criança e no adolescente (NE: C). ^727^ O Quadro 12.8 apresenta os medicamentos utilizados em pediatria e suas doses. ^705^ ^,^ ^725^ ^,^ ^726^

**Quadro 12.8:** – Medicamentos anti-hipertensivos utilizados em crianças e adolescentes no Brazil

Fármaco	Idade	Dose inicial	Dose máxima	Intervalo
Clonidina	> 12 a	0,2 mg/dia	2,4 mg/dia	12h
Atenolol		0,5-1 mg/kg/dose	2 mg/kg/dia (máx. 100 mg/dia)	12-24 h
Propranolol		1-2 mg/kg/dose	4 mg/kg/dia (máx. 640 mg/dia)	8-12 h
Anlodipino	1-5 anos	0,1 mg/kg/dose	0,6 mg/kg/dia (máx. 5 mg/dia)	24 h
	> 6 anos	2,5 mg/dia	10 mg/dia	24 h
Isradipino	Criança	0,05-0,1 mg/kg/dose	0,6 mg/kg/dia (máx. 10 mg/dia)	8-12 h
Felodipino	> 6 anos	2,5 mg/dia	10 mg/dia	24 h
Nifedipino XL		0,25-0,5 mg/kg/dose	3 mg/kg/dia (máx. 120 mg/dia)	12-24 h
Candesartana	1-5 anos	0,02 mg/kg/dose (máx. 4 mg/dia)	0,4 mg/kg/dia (máx. 16 mg/dia)	12-24 h
Olmesartana	> 6 anos	< 35 kg: 10 mg/dia	< 35 kg: 20 mg/dia	24h
		> 35 kg: 20 mg/dia	> 35 kg: 40 mg/dia	24 h
Losartana	> 6 anos	0,7 mg/kg/dia (máx. 50 mg/dia)	1,4 mg/kg/dia (máx. 100 mg/dia)	24 h
Valsartana	> 6 anos	1,3 mg/kg/dia	2,7 mg/kg/dia (máx. 160 mg/dia)	24 h
Prazosina	> 12 anos	0,05-0,1 mg/kg/dose	0,5 mg/kg/dia	8 h
Furosemida		0,5-2 mg/kg/dose	6 mg/kg/dia	4-12 h
Espironolactona		1 mg/kg/dose	3,3 mg/kg/dia	6-12 h
Clortalidona	> 40 kg	0,3 (máx. 50 mg/dia)	2 mg/kg/dia	24 h
Hidroclorotiazida		1 mg/kg/dose	2 mg/kg/dia (máx. 37,5 mg/dia)	
Benazeprila	> 6 anos	0,2 (máx. 10 mg/dia)	0,6 mg/kg/dia (máx. 40 mg/dia)	24 h
Captoprila	Lactente	0,05 mg/kg/dose	6 mg/kg/dia	6-24h
	Criança	0,5 mg/kg/dose	6 mg/kg/dia	8 h
Enalaprila	> 1 mês	0,08 mg/kg/dose	0,6 mg/kg/dia (máx. 40 mg/dia)	12-24 h
Fosinoprila	> 6 anos	0,2 mg/kg/dose (máx. 10 mg/dia)	0,6 mg/kg/dia (máx. 40 mg/dia)	24 h
Lisinoprila	> 6 anos	0,07 mg/kg/dose (máx. 5 mg/dia)	0,6 mg/kg/dia (máx. 40 mg/dia)	24 h
Ramiprila		1,6 mg/m ^2^ /dia	0,6 mg/kg/dia	24 h
Hidralazina		0,75 mg/kg/dose	7,5 mg/kg/dia (máx. 200 mg/dia)	6 h
Minoxidila	< 12 anos	0,2 mg/kg/dose	50 mg/dia	6-8 h
	< 12 anos	5 mg/dia	100 mg/dia	

Máx.: máximo; h: horas.

## 12.11. Seguimento de Crianças e Adolescentes com HA

A frequência do acompanhamento de crianças e adolescentes com HA dependerá de sua gravidade e sua necessidade de tratamento. Nos pacientes submetidos apenas a terapia não farmacológica, deve-se acompanhar clinicamente a cada 3 a 6 meses, utilizando a MRPA como adjuvante do controle.

Nos que necessitarem medicação, logo após a introdução inicial, o seguimento deve ser quinzenal ou mensal até a determinação da dose ideal ou da necessidade de associação. Em uma fase intermediária, a cada 4 a 6 semanas, e trimestralmente quando a HA estiver controlada.

Na consulta de seguimento, devem-se detalhar a adesão e os efeitos colaterais. A solicitação de exames laboratoriais dependerá da medicação utilizada, da gravidade da HA e das doenças de base existentes, assim como a frequência de checagem de LOA dependerá da doença de base e da gravidade da HA. A solicitação da MAPA no seguimento está indicada quando não houver controle da HA ou nos casos de risco de hipertensão mascarada (HM), como no pós-operatório tardio de coarctação de aorta (NE: C). ^705^

## 12.12. Crise Hipertensiva

A emergência hipertensiva (EH) e a urgência hipertensiva (UH) estão definidas no Capítulo 13. ^727^ Não há consenso quanto ao nível de PA para definir EH, ^728^ embora alguns autores sugiram como ponto de corte 20% acima do estágio 2 de HA (> percentil 99). ^729^ Já a Associação Americana de Pediatria (AAP) define EH como qualquer condição em que a criança apresenta PA acima do estágio 2 de HA. A AAP alerta, no entanto, que crianças com PA > percentil 95 + 30mmHg correm maior risco de complicações. Geralmente, as EH são secundárias a doença subjacente que necessita investigação, ^705^ e seu tratamento deve ser realizado com o paciente internado, na maioria das vezes em UTI, com medicação intravenosa (IV). Pacientes com UH, sem sinais de comprometimento de órgãos-alvo, podem fazer uso inicialmente de alfa-agonistas centrais, vasodilatadores ou BCC. ^705^ O objetivo do tratamento é reduzir a PA em 25% nas primeiras 8h, seguida de uma redução lenta em torno de 24-48h, até alcançar o percentil 95, uma vez que a redução rápida poderá acarretar danos, principalmente cerebrais. ^730^ ^,^ ^731^ O Quadro Quadro 12.9 apresenta os medicamentos mais utilizados na EH pediátrica (NE: C).

**Quadro 12.9:** – Principais medicamentos e doses pediátricas utilizados para controle da emergência hipertensiva

Medicamento	Via	Dose	Início da ação	Duração
**Nitroprussiato de sódio**	IV	0,5-10 µg/kg/min	Segundos	Somente durante a infusão
**Labetamol**	IV	0,25-3 mg/kg/h ou dose em bolo de 0,2-1 mg/kg seguida de infusão de 0,25-3 mg/kg/h	2-5 min	2-4 h
**Nicardipina**	IV	1-3 µg/kg/min	2-5 min	30 min-4 h, maior quanto mais longo o uso
**Hidralalizina**	IV IM	0,2-0,6 mg/kg em bolo IV ou IM, máx. = 20mg	10-30 min	4-12 h
**Esmolol**	IV	Ataque 100-500 µg/kg seguido de infusão 50-300 µg/kg/min	Segundos	10-30 min
**Fentolamina**	IV	0,05-0,1 mg/kg em bolo máx. = 5 mg/dose	Segundos	15-20 min

IV: intravenoso; IM: intramuscular; min.: minuto; h: hora. Adaptado de Flynn et al., 2017. ^705^

**Table:** 

Mensagens principais
Toda criança e adolescente ≥ 3 anos de idade deve ter a PA verificada anualmente.
Crianças < 3 anos de idade devem ter a PA medida em caso de prematuridade, muito baixo peso ao nascer, restrição de crescimento intrauterino, antecedente de internação em UTI neonatal, cardiopatias congênitas, nefrouropatias, transplante de órgãos sólidos, doença oncológica, uso crônico de medicações que elevam a PA, doenças sistêmicas associadas a HA e evidência de hipertensão intracraniana.
Toda criança/adolescente ≥ 3 anos de idade, com excesso de peso, uso crônico de medicações que elevam a PA, doença renal, coarctação de aorta e diabetes, deve ter sua PA verificada em qualquer avaliação clínica.
Será feito o diagnóstico de HA na criança e no adolescente no ambulatório, quando pelo método auscultatório a PA estiver > percentil 95 em três visitas distintas, de acordo com idade, sexo e percentil de estatura.
Em crianças e adolescentes com diagnóstico de HA, as metas referentes à terapia não farmacológica e farmacológica devem ser a redução da PA < percentil 90 de acordo com idade, sexo e percentil de estatura e <130/80mmHg em adolescentes ≥ 13 anos de idade.

## 13. Crise Hipertensiva

## 13.1. Definição

Os termos urgência e emergência hipertensiva surgiram como proposta para uma classificação operacional da crise hipertensiva (CH), em 1993, pelo *V Joint National Committee on Detection Evaluation and Treatment of High Blood Pressure* . ^732^

As urgências hipertensivas (UH) são situações clínicas sintomáticas em que há elevação acentuada da pressão arterial (PA) (definida arbitrariamente como PA sistólica (PAS) ≥ 180 e/ou diastólica (PAD) ≥ 120 mm Hg) *sem* lesão aguda e progressiva em órgãos-alvo (LOA) e sem risco iminente de morte. ^5^ ^,^ ^164^ ^,^ ^733^ ^,^ ^734^

Já as emergências hipertensivas (EH) são situações clínicas sintomáticas em que há elevação acentuada da PA (definida arbitrariamente como PAS ≥ 180 e/ou PAD ≥ 120 mm Hg) *com* LOA aguda e progressiva, com risco iminente de morte. ^5^ ^,^ ^164^ ^,^ ^733^ ^,^ ^734^

Uma condição comum nos setores de emergência é a pseudocrise hipertensiva (PCH). Na PCH, não há LOA aguda ou risco imediato de morte. Geralmente, ocorre em hipertensos tratados e não controlados, ou em hipertensos não tratados, com medidas de PA muito elevadas, mas oligossintomáticos ou assintomáticos. Também se caracteriza como PCH a elevação da PA diante de evento emocional, doloroso, ou de algum desconforto, como enxaqueca, tontura rotatória, cefaleias vasculares e de origem musculoesquelética, além de manifestações da síndrome do pânico. ^733^ ^,^ ^734^

## 13.2. Classificação

A EH não é definida pelo nível da PA, apesar de frequentemente muito elevada, mas predominantemente pelo *status* clínico do paciente. Pode manifestar-se como um evento cardiovascular, cerebrovascular, renal ou com envolvimento de múltiplos órgãos ou mesmo na forma de pré-eclâmpsia com sinais de gravidade/eclâmpsia. Nesse contexto, o Quadro 13.1 mostra a classificação das EH. O Quadro 13.2 diferencia UH da EH com relação ao diagnóstico, prognóstico e conduta.

**Quadro 13.2:** – Diagnóstico, prognóstico e conduta nas urgências e emergências hipertensivas

Urgência	Emergência
Nível pressórico elevado acentuado	Nível pressórico elevado acentuado
Sem LOA aguda e progressiva	Com LOA aguda e progressiva
Combinação medicamentosa oral	Fármaco parenteral
Sem risco iminente de morte	Com risco iminente de morte
Acompanhamento ambulatorial precoce (7 dias)	Internação preferencial em UTI

LOA: lesão em órgãos-alvo; UTI: unidade de tratamento intensivo.

**Quadro 13.1:** – Classificação das emergências hipertensivas

EMERGÊNCIAS HIPERTENSIVAS
**Cerebrovasculares** • Encefalopatia hipertensiva • Acidente vascular encefálico isquêmico • Acidente vascular encefálico hemorrágico • Hemorragia subaracnóidea **Cardiocirculatórias** • Dissecção aguda de aorta • Edema agudo de pulmão com insuficiência ventricular esquerda • Síndromes coronarianas agudas **Renais/comprometimento de múltiplos órgãos** • Hipertensão acelerada/maligna • Hipertensão MDO
• Crises adrenérgicas graves • Crise do feocromocitoma • Dose excessiva de drogas ilícitas (cocaína, crack, LSD)
• Hipertensão na gestação • Eclâmpsia • Pré-eclâmpsia com sinais de gravidade • Síndrome “HELLP” • Hipertensão grave em final de gestação

MDO: múltiplos danos aos órgãos-alvo. HELPP: hemolysis, elevated liver enzymes, low platelets. Adaptado de Malachias et al., 2016; ^164^ Bortolotto et al., 2018; ^733^ Martion & Ribeiro, 2015; ^734^ Whelton et al., 2018 ^5^ ; Cremesp, 2004; ^746^ Williams et al., 2018; ^37^ Ma et al., 2020. ^778^

## 13.3. Principais Aspectos Epidemiológicos, Fisiopatogênicos e Prognósticos

### 13.3.1. Epidemiologia

A CH responde por 0,45% a 0,59% de todos os atendimentos de emergência hospitalar e EH por 25% de todos os casos de CH. O acidente vascular encefálico isquêmico (AVEI) e o edema agudo de pulmão (EAP) são as situações mais encontradas nas EH. ^735^ ^-^ ^737^ É condição clínica de incidência decrescente nas últimas décadas. ^738^ ^-^ ^740^

### 13.3.2. Fisiopatogenia

Tendo em vista que a PA sistêmica é resultante do produto do débito cardíaco (DC) pela resistência vascular periférica (RVP), elevações agudas da PA podem resultar de alterações nessas variáveis. Assim, aumento do volume intravascular, da RVP, produção reduzida de vasodilatadores endógenos e/ou ativação de sistemas vasoconstrictores podem precipitar maior vasorreatividade resultando em CH. ^741^ ^,^ ^742^ A autorregulação tissular é suplantada, particularmente nos leitos vasculares cerebral e renal, causando isquemia local, o que desencadeia um círculo vicioso de vasoconstrição, lesão endotelial e ativação plaquetária, do sistema da coagulação e do sistema imune, com proliferação miointimal, necrose fibrinoide de arteríolas e isquemia em órgãos-alvo. ^741^ ^-^ ^743^ A curva de autorregulação é deslocada para a direita nos hipertensos crônicos, o que faz com que tanto o nível de PA atingido quanto a velocidade da elevação sejam importantes na gênese da EH. Por outro lado, esse deslocamento da curva de autorregulação predispõe à isquemia tissular em reduções agressivas da PA no tratamento das EH. ^742^ ^,^ ^743^

### 13.3.3. Prognóstico

A letalidade da EH, caso não tratada, é de aproximadamente 80% ao final de um ano, ^739^ e o tratamento anti-hipertensivo efetivo associa-se à melhora substancial em seu prognóstico. ^740^ A sobrevida de até 5 anos é maior em indivíduos com UH do que com EH. ^735^ ^,^ ^744^

## 13.4. Investigação Clinicolaboratorial Complementar 164,733,734 

É essencial a realização de uma história clínica direcionada para a causa possível. A investigação clínica e a solicitação de exames devem prover a adequada avaliação da PA e a presença de LOA agudas. A PA deve ser medida inicialmente nos dois braços, de preferência em um ambiente calmo, e repetidamente até a estabilização (no mínimo, três medidas). Deve-se rapidamente coletar informações sobre a PA habitual do paciente e as situações que possam desencadear um aumento da PA e comorbidades; o uso de fármacos anti-hipertensivos ou sua descontinuação (particularmente inibidores adrenérgicos); ou a utilização de substâncias que aumentem a PA (ver Capítulo 15). Uma abordagem sistematizada com a avaliação de sinais/sintomas, exame físico e investigação complementar auxilia na verificação da presença de LOA aguda ou progressiva, mostrada no Quadro Quadro 13.3 .

**Quadro 13.3:** – Investigação clínico-complementar de acordo com as lesões de órgãos-alvo das emergências hipertensivas

Principais lesões nas EH	Sintomas	Exame físico	Investigação complementar a critério clínico
**Cardiovasculares**	- Dor ou desconforto no tórax, abdome ou dorso; - Dispneia; fadiga; tosse.	- FC, ritmo, alteração de pulso, galope, estase jugular, congestão pulmonar, abdominal e periférica; - Sopros cardíacos e vasculares; - Palpação de pulsos nos quatro membros.	- ECG, saturação de O2, radiografia de tórax, marcadores de necrose miocárdica, BNP, desidrogenase láctica; - Ecocardiograma; - Angiotomografia, TC de tórax e RNM de tórax.
**Neurológicas**	- Tontura; cefaleia; - Visão, audição ou fala alterada	- Nível de consciência ou coma; agitação, delírio ou confusão; convulsão; déficits focais; rigidez de nuca.	- TC crânio; RNM crânio.
**Renais**	- Alteração no volume e na frequência miccional.	- Edema ou desidratação; - Alterações no aspecto da urina (hematúria); - Massas e sopros abdominais.	- Urina I; creatinina; ureia; Na+; K+; cloro; gasometria.
**Fundo de olho**		- Papiledema; hemorragias; exsudatos. - Alterações nos vasos como espasmos, cruzamentos arteriovenosos patológicos, espessamento na parede arterial e aspecto em fio de prata ou cobre.	
**Exames complementares mínimos**	- ECG, radiografia de tórax, marcadores de necrose miocárdica, hemograma com plaquetas, creatinina, urina I e potássio.		

EH: emergência hipertensiva; FC: frequência cardíaca; ECG: eletrocardiograma; BNP: peptídeo natriurético atrial; TC: tomografia computadorizada; RNM: ressonância nuclear magnética. Adaptado de Malachias et al., 2016; ^164^ Bortolotto et al., 2018; ^733^ Martion & Ribeiro, 2015; ^734^ Whelton et al., 2018; ^5^ Vilela-Martin et al., 2020. ^747^

## 13.5. Tratamento Geral da Crise Hipertensiva

**Figura 13.1.**
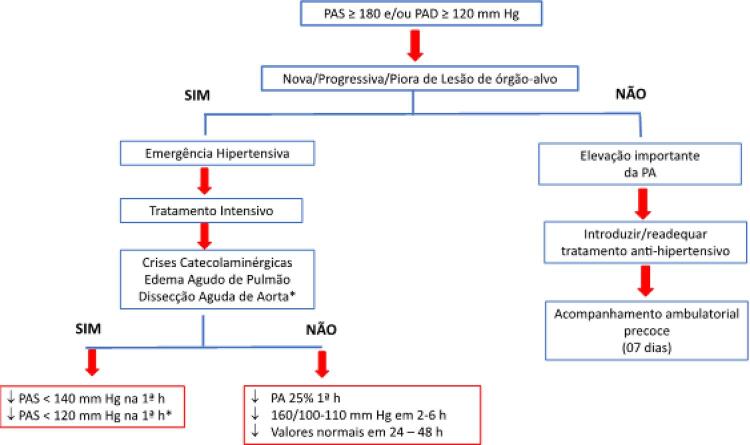
– Fluxograma de atendimento da crise hipertensiva.

O tratamento da UH ( Figura 13.1 ) deve ser iniciado após um período de observação em ambiente calmo, condição que ajuda a afastar casos de PCH (conduzidos somente com repouso ou uso de analgésicos ou tranquilizantes). Para o tratamento agudo, indicam-se a captoprila e a clonidina. A captoprila, na dose de 25-50mg, tem seu pico máximo de ação em 60 a 90 minutos, enquanto a clonidina apresenta ação rápida, em torno de 30 a 60 minutos, na dose de 0,100 a 0,200mg. O uso de cápsulas de nifedipina de liberação rápida deve ser proscrito no tratamento das UH, por não ser seguro nem eficaz, além de provocar reduções rápidas e acentuadas da PA, o que pode resultar em isquemia tecidual. ^745^ ^,^ ^746^

Adaptada de Whelton et al., 2018. ^5^

**Figura 15.3.**
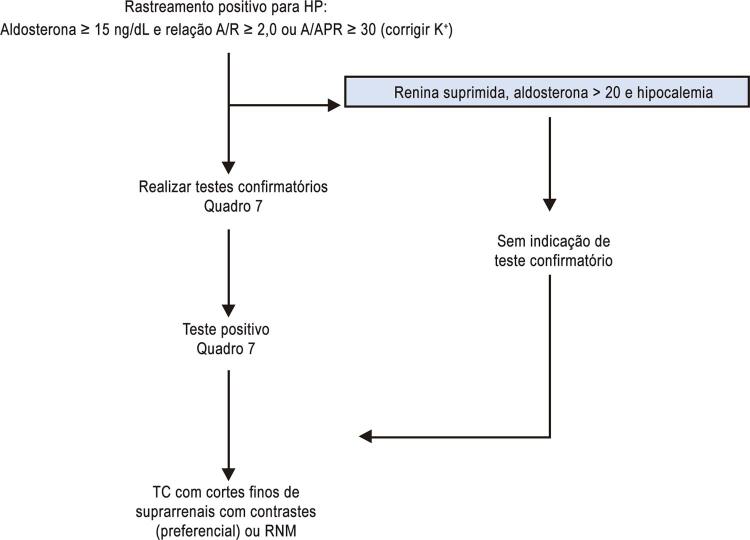
– Fluxograma de investigação diagnóstica de hiperaldosteronismo primário.

Adaptado de Vilela & Almeida, 2016. ^924^

Não há evidência de ensaios clínicos randomizados mostrando que os anti-hipertensivos reduzem a morbidade ou a mortalidade em indivíduos com EH. No entanto, baseando-se na experiência clínica e na evolução dos pacientes tratados, o tratamento anti-hipertensivo é benéfico e reduz a mortalidade. O tratamento dos pacientes com EH visa à redução rápida da PA com a finalidade de impedir a progressão das LOA. Os indivíduos devem ser admitidos preferencialmente em UTI, tratados com anti-hipertensivos intravenosos (IV) e monitorados cuidadosamente durante a terapia para evitar hipotensão. As recomendações gerais de redução da PA para EH devem ser (GR: I; NE: C): ^5^ ^,^ ^164^

• PA média ≤ 25% na 1 ^a^ hora;
• PA 160/100-110 mmHg nas próximas 2 a 6 h;
• PA 135/85 mmHg em um período de 24-48 h subsequentes.

Entretanto, as EH devem ser abordadas considerando o sistema ou o órgão-alvo acometido.

## 13.6. Emergências Hipertensivas em situações especiais

O Quadro 13.4 mostra os medicamentos indicados para as principais EH.

**Quadro 13.4:** – Medicamentos usados por via parenteral para o tratamento das emergências hipertensivas

Fármacos	Modo de administração e dosagem	Início	Duração	Indicações	Eventos adversos e precauções
Nitroprussiato de Sódio (vasodilatador arterial e venoso estimula a formação de GMPc)	Infusão contínua 0,25-10 mg/kg/min IV	Imediato	1-2 min	Maioria das emergências hipertensivas	Intoxicação por cianeto, hipotensão grave, náuseas, vômitos. Cuidado na insuficiência renal e hepática e pressão intracraniana alta. Proteger da luz.
Nitroglicerina (vasodilatador arterial e venoso)	Infusão contínua IV 5-15mg/h	2-5 min	3-5 min	Insuficiência coronariana, Insuficiência ventricular esquerda com EAP	Cefaleia, taquicardia reflexa, taquifilaxia, flushing, meta-hemoglobinemia
Metoprolol (bloqueador beta-adrenérgico seletivo)	5 mg IV (repetir 10/10 min, se necessário até 20 mg)	5-10 min	3-4 h	Insuficiência coronariana Dissecção aguda de aorta (em combinação com NPS)	Bradicardia, BAV avançado, insuficiência cardíaca, broncoespasmo
Esmolol (bloqueador beta-adrenérgico seletivo de ação ultrarrápida)	Ataque: 500μg/kg Infusão intermitente IV 25-50 μg/kg/min ↑ 25 μg/kg/min cada 10-20min. Máximo 300 μg/kg/min	1-2 min	1-20 min	Dissecção Aguda de Aorta (em combinação com NPS) Hipertensão pós-operatória grave	Náuseas, vômitos, BAV 1o grau, broncoespasmo, hipotensão
* Fentolamina (bloqueador α-adrenérgico)	Infusão contínua IV: 1-5mg. Máximo 15mg	1-2 min	3-5 min	Excesso de catecolaminas	Taquicardia reflexa, flushing, tontura, náuseas, vômitos
* Trimetafan (bloqueador ganglionar do SNS e SNPS)	Infusão contínua IV: 0,5-1,0 mg/min. ↑ 0,5mg/min até o máximo de 15mg/min	1-5 min	10 min	Excesso de catecolaminas Dissecção aguda de aorta	Taquifilaxia
Hidralazina (vasodilatador de ação direta)	10-20 mg IV ou 10-40 mg IM 6/6 h	10-30 min	3-12 h	Eclâmpsia	Taquicardia, cefaleia, vômitos. Piora da angina e do infarto. Cuidado com pressão intracraniana elevada
Diazóxido (vasodilatador da musculatura lisa arteriolar)	Infusão IV 10-15min 1-3 mg/kg Máximo 150 mg	1-10 min	3-18 h	Encefalopatia hipertensiva	Retenção de sódio, água, hiperglicemia e hiperuricemia
* Fenoldopam (agonista seletivo dopaminérgico)	Infusão contínua IV 0,1-1,6 μg/kg/min	5-10 min	10-15 min	Insuficiência renal aguda	Cefaleia, náuseas, rubor
* Nicardipina (bloqueador dos canais de cálcio)	Infusão contínua IV 5-15mg/h	5-10 min	1-4 h	Acidente vascular encefálico Encefalopatia hipertensiva Insuficiência ventricular esquerda com EAP	Taquicardia reflexa, flebite evitar em pacientes com insuficiência cardíaca ou isquemia miocárdica
* Labetalol (bloqueador alfa/beta-adrenérgico)	Ataque: 20-80mg 10-10min IV Infusão contínua IV 2mg/min (máximo 300mg/24h)	5-10 min.	2-6 h	Acidente vascular encefálico Dissecção aguda de aorta (em combinação com NPS)	Náuseas, vômitos, BAV, broncoespasmo, hipotensão ortostática
Enalaprilato (inibidor da ECA)	Infusão intermitente IV 5,0 mg 6/6h até 20 mg	15 min.	4-6 h	Insuficiência ventricular esquerda com EAP	Hipotensão, insuficiência renal, gestação
Furosemida (diurético de alça)	20-60 mg IV (repetir após 30 min)	2-5 min	30-90 min.	Insuficiência ventricular esquerda com EAP Situações de hipervolemia como DRC, GNDA	Hipopotassemia

* Não disponíveis no Brazil. NPS = nitroprussiato de sódio; IV = intravenoso; EAP = edema agudo de pulmão; BAV = bloqueio atrioventricular; SNS = sistema nervoso simpático; SNPS = sistema nervoso parassimpático; ECA = enzima de conversão da angiotensina; DRC = doença renal crônica; GNDA= glomerulonefrite difusa aguda. Adaptado de Malachias et al., 2016; ^16^ 4 Bortolotto et al., 2018; ^733^ Martion & Ribeiro, 2015; ^734^ Whelton et al., 2018; ^5^ Vilela-Martin et al., 2020. ^747^

### 13.6.1. Encefalopatia Hipertensiva 747,748 

A encefalopatia hipertensiva é uma EH neurológica caracterizada por sinais e/ou sintomas de edema cerebral secundário à elevação súbita e/ou mantida da PA. Geralmente, ocorre em hipertensos crônicos que desenvolvem hipertensão acelerada-maligna ou nos indivíduos previamente normotensos que apresentam elevações súbitas da PA, que cursam com falência dos mecanismos de autorregulação da perfusão cerebral. O início é insidioso e evolui com cefaleia, náuseas ou vômitos. Podem surgir alterações do campo visual, fotopsia, visão turva, alucinações visuais, confusão mental, coma, crises convulsivas generalizadas e hiper-reflexia. O objetivo do tratamento consiste em diminuir a PA de forma lenta porque reduções intensas e rápidas podem provocar hipoperfusão cerebral e perda do mecanismo de autorregulação cerebral. Recomenda-se o uso de nitroprussiato de sódio (NPS) em nosso meio. Em outros países, estão disponíveis e indicados nicardipina, clevidipina, labetalol ou fenoldopam. Nas primeiras 24 a 48 h, anti-hipertensivos de ação oral devem ser iniciados para o melhor controle da PA.

## 13.7. Acidente Vascular Encefálico (AVE)

A hipertensão é o principal fator de risco para o AVE, principalmente o hemorrágico (AVEH). ^749^ O diagnóstico baseia-se no exame neurológico completo; e, para avaliação da gravidade do quadro, deve ser utilizada a escala do *National Institute of Health Stroke Scale* (NIHSS). A TC e a RNM de crânio possibilitam definir o tipo do AVE (AVEI em 85% ou AVEH em 15% dos casos) e o território envolvido. ^164^ ^,^ ^750^ A RNM é mais sensível do que a TC para os infartos incipientes.

### 13.7.1. Acidente Vascular Encefálico Isquêmico (AVEI)

A PA frequentemente diminui espontaneamente em um período de 90 a 120 minutos durante a fase aguda. As recomendações são as seguintes:

1. Em caso de AVEI com indicação de trombólise, recomenda-se uma redução da PA < 185/110 mmHg antes da terapia fibrinolítica (GR: I; NE: B). ^5^ ^,^ ^652^ Se a PA permanecer > 185/110 mmHg, a terapêutica trombolítica não deverá ser administrada. Essa recomendação também se aplica a indivíduos que serão submetidos à trombectomia. ^751^ A PA deve ser mantida < 180/105 mmHg nas primeiras 24h após trombólise.
2. A redução inicial da PA em 15% pode ser aplicada nos casos de PA muito elevada (≥ 220/120 mm Hg) e com outras EH associadas (dissecção de aorta, eventos coronarianos agudos, eclâmpsia, pós-trombólise e/ou EAP) (GR: I; NE: C). ^5^ ^,^ ^652^
3. Em pacientes com PA ≥ 220/120 mmHg que não receberem trombolítico e não apresentarem outras EH que necessitem de tratamento anti-hipertensivo, o benefício de iniciar ou reiniciar tratamento da hipertensão nas primeiras 48 a 72h é incerto. Parece ser prudente reduzir PA em 15% durante as primeiras 24 h após o início do AVEI (GR: IIb; NE: C). ^5^ ^,^ ^652^
4. Iniciar ou reiniciar a terapia anti-hipertensiva durante a hospitalização em pacientes com PA ≥ 140/90 mmHg, que estejam neurologicamente estáveis, é seguro para melhorar o controle de PA a longo prazo (NE: B; CR: IIa). ^5^ ^,^ ^652^
5. Nos demais casos de AVEI, a redução da PA em 5 a 7 dias após o evento tem efeitos neurológicos controversos, sendo necessária a individualização do tratamento (GR: I; NE: A). ^652^

### 13.7.2. Acidente Vascular Encefálico Hemorrágico (AVEH)

A elevação da PA aumenta o risco de expansão do hematoma e o risco de morte, além de piorar o prognóstico da recuperação neurológica. No entanto, as evidências não são conclusivas para a rápida redução da PA. O edema cerebral ocorre em 30% dos casos e normalmente ocorre nas primeiras 24 h. Nesses casos, a craniectomia descompressiva e a transferência para centros especializados deve ser realizada (GR: I; NE: B). ^5^ ^,^ ^752^

Para indivíduos com apresentação aguda (< 6 h do início do AVEH):

1. PAS > 220 mmHg – considerar a redução da PA com infusão IV contínua e o monitoramento frequente da PA (GR: IIa; NE: C). ^5^ ^,^ ^752^
2. PAS entre 150 a 220 mmHg – reduzir a PA abaixo de 140 mmHg não apresenta benefícios para diminuir mortalidade ou incapacidade grave e é potencialmente perigoso (GR: III; NE: A). ^5^ ^,^ ^752^ Considerar alvo de PAS < 180 mmHg. ^37^

### 13.7.3. Síndromes Coronarianas Agudas

As síndromes coronarianas podem estar acompanhadas de elevação da PA, devido a um reflexo desencadeado pelo miocárdio isquêmico. Em consequência, o aumento da RVP eleva a demanda de oxigênio pelo miocárdio. O objetivo é reduzir a pós-carga sem aumentar a FC ou sem reduzir exageradamente a pré-carga, pois isso levaria a um incremento no consumo de oxigênio pelo miocárdio. A meta de PAS < 140 mmHg (evitar < 120 mmHg) e PAD entre 70-80 mmHg deve ser buscada, utilizando-se esmolol, metoprolol ou nitroglicerina (GR: I; NE: A). Os nitratos IV reduzem a RVP, melhoram a perfusão coronariana e têm importante efeito venodilatador sistêmico, diminuindo a pré-carga e o consumo de oxigênio pelo miocárdio. O uso de hidralazina, NPS ou nifedipina não está indicado, pois pode promover o roubo de fluxo. ^164^ ^,^ ^733^ Recomendam-se:

- a) A nitroglicerina (NTG) IV é indicada nas primeiras 48 horas para o tratamento de hipertensão, isquemia persistente e IC, desde que não haja hipotensão, infarto do ventrículo direito ou uso de inibidores da fosfodiesterase tipo 5 nas 48 horas anteriores (GR: I; NE: B). O uso de NTG não deve excluir a terapêutica com outras intervenções de redução de mortalidade comprovadas, como betabloqueadores (BB) ou IECA. ^753^ ^,^ ^754^
- b) BB IV em indivíduos com hipertensão que não apresentem: 1) sinais de IC; 2) evidência clínica de baixo DC; 3) aumento do risco para choque cardiogênico; ou 4) outras contraindicações relativas ao bloqueio beta (GR: IIa, NE: B). ^753^ ^,^ ^756^

### 13.7.4. Edema Agudo de Pulmão (EAP)

Cerca de 1/3 dos pacientes admitidos com EAP e EH têm a função ventricular esquerda preservada, e a isquemia miocárdica também pode estar envolvida na fisiopatogenia do EAP associado à EH. ^755^ ^,^ ^756^ A EH com EAP deve ser preferencialmente controlada em UTI, com medicação IV, monitoramento e diminuição gradativa da PA. NTG e NPS são utilizados com a finalidade de reduzir a pré e a pós-carga. O uso de diurético de alça também diminui a sobrecarga de volume e, consequentemente, a PA. Em alguns casos, o uso de pressão positiva contínua não invasiva de vias respiratórias pode ser indicado para reduzir o edema pulmonar e o retorno venoso. ^747^ ^,^ ^748^ ^,^ ^757^

#### 13.7.4.1. Dissecção Aguda de Aorta

Em pacientes com dor precordial e elevação da PA, sempre considerar a dissecção aguda de aorta. A progressão da dissecção está relacionada com o valor de PA e a velocidade de ejeção ventricular. ^758^ É importante obter o controle adequado da dor (analgesia por opiáceos IV), o FC < 60 bpm e a PAS entre 100 e 120mmHg (GR: I; NE: B). ^5^ ^,^ ^747^ ^,^ ^758^ A PAS < 120 mmHg deve ser alcançada em 20 min. O uso isolado de NPS não é ideal, pois promove o aumento da FC e da velocidade de ejeção aórtica, podendo piorar a dissecção. ^5^ ^,^ ^747^ ^,^ ^758^ Assim, o NPS deve ser associado ao BB, inicialmente IV, de ação rápida e titulável (metoprolol, labetalol ou esmolol), para diminuir a FC. Em pacientes asmáticos, os bloqueadores dos canais de cálcio (BCC) não dihidropiridínicos podem ser usados como alternativa.

## 13.7.5. Pré-eclâmpsia/Eclâmpsia (ver Capítulo 11)

## 13.7.6. EH pelo Uso de Substâncias Ilícitas

As substâncias ilícitas que elevam a PA têm ação simpaticomimética, potencializando o efeito das catecolaminas, entre elas as anfetaminas e seu derivado ilegal *ecstasy,* além da cocaína e sua forma para fumar, o *crack* . ^5^ ^,^ ^759^ ^,^ ^760^ As anfetaminas aumentam a PA em dose-dependente, ^761^ causando taquicardia, palpitações, sudorese e arritmias, enquanto o *ecstasy* tem outros efeitos, além do aumento da FC e PA (síndrome serotoninérgica). ^762^

Quando a cocaína é utilizada por via intranasal, ocorre o aumento repentino e perigoso dos níveis da PA, observado 15 minutos após o uso. Em caso de hipertensão preexistente, pode haver maiores elevações da PA. ^763^ A vasoconstrição induzida pela cocaína depende da descarga simpática central, que se encontra suprimida com função barorreceptora intacta. Quando o tamponamento barorreflexo está prejudicado, ocorrem vasoconstrição adrenérgica excessiva e CH. ^764^

Nos casos mais leves, podem-se usar benzodiazepínicos e NTG sublingual. Nos mais graves, provavelmente será necessária terapia IV, e os agentes de escolha são NTG, NPS ou fentolamina. ^759^ ^,^ ^760^ É importante evitar os BB, pois podem levar à estimulação do receptor alfa-adrenérgico na presença do bloqueio beta-adrenérgico, causando espasmo de coronária. ^763^ Uma exceção pode ser o carvedilol, capaz de atenuar aumentos da FC e PA induzidos por cocaína na forma de *crack* . ^765^ Os BCC também podem ser usados nos casos de IAM devido ao uso de cocaína, situação em que a vasoconstrição coronariana seja a causa presumida. ^760^

Um complicador dessas intoxicações, seja como UH ou EH, é a ingestão concomitante de altas doses de cafeína (presente em energéticos), nicotina ou álcool, fato que aumenta o nível de NA plasmática. ^766^ Particularmente, a associação de álcool mais cocaína tem efeito tóxico maior do que o uso isolado de cada um, ^767^ ^,^ ^768^ aumentando o risco de morte súbita em 18 a 25 vezes, ^769^ por elevar a biodisponibilidade da cocaína. ^770^ O tratamento inclui o uso de BB, alfabloqueadores e BCCa, este administrado antes ou depois do consumo da cocaína. ^760^ ^,^ ^771^

## 13.7.7. Hipertensão Acelerada/Maligna

A hipertensão maligna caracteriza-se pela presença de hipertensão em geral grave, retinopatia com papiledema, com ou sem insuficiência renal e/ou cardíaca, necrose fibrinoide de arteríolas renais e endarterite obliterante, podendo apresentar evolução clínica rapidamente progressiva e fatal. A elevação da PA na presença de hemorragias retinianas e exsudatos ao fundo de olho, mas sem papiledema, é denominada hipertensão acelerada. Atualmente, os termos “maligna” e “acelerada” são considerados intercambiáveis, sendo o termo hipertensão acelerada/maligna mais usado para definir essa EH que, apesar de menos frequente, representa uma forma devastadora de elevação aguda da PA. ^747^ ^,^ ^772^ ^,^ ^773^ Seu prognóstico mostra-se quase sempre fatal, se não for reconhecida ou não tratada adequadamente, com uma mortalidade de cerca de 80% em dois anos, principalmente em decorrência de IC e DRC. ^774^ ^,^ ^775^ O tratamento eficaz da hipertensão maligna melhorou consideravelmente a sobrevida, mas ainda cursa com alto índice de complicações. ^776^ O modo mais racional de manuseá-la é preveni-la, instituindo-se tratamento precoce e eficiente para a hipertensão. Indivíduos com hipertensão grave que apresentem HVE importante e insuficiência renal devem ser acompanhados como previamente portadores de hipertensão acelerada/maligna.

Os pacientes devem ser internados para controle intensivo da PA com fármacos vasodilatadores de ação imediata, como NPS, que promove controle rápido da PA e torna os indivíduos mais responsíveis à terapêutica anti-hipertensiva clássica. ^164^ ^,^ ^732^ Durante o controle agudo, deve-se instituir anti-hipertensivos de uso oral, incluindo diuréticos, bloqueadores do sistema renina-angiotensina, BB, vasodilatadores de ação direta (hidralazina), agonistas adrenérgicos centrais (clonidina e metildopa) e BCC, quando necessário o uso de vários farmacos. ^747^ ^,^ ^774^ O uso de BB é melhor indicado nos casos de congestão pulmonar, devido à disfunção diastólica decorrente de grave HVE. A redução da PA deve ser gradual, mantendo-se níveis de PAD não inferiores a 100 mmHg nos primeiros dias de tratamento. Pode ocorrer deterioração inicial da função renal com elevação de creatinina, devido ao mecanismo de autorregulação do fluxo renal estar desviado para níveis muito superiores aos dos hipertensos leves ou dos normotensos. Assim, deve-se aguardar um período de readaptação para o retorno da creatinina aos valores basais. Às vezes, o tratamento dialítico pode ser necessário na fase mais aguda. O tratamento anti-hipertensivo dessa condição modificou a sobrevida de forma impactante (GR: IIa; NE: B).

## 13.7.8. Hipertensão com Múltiplos Danos aos Órgãos-alvo

A hipertensão com múltiplos danos aos órgãos-alvo (MDO), conhecida em inglês como MOD ( *hypertension with multi organ damage* ), é definida pela presença concomitante de envolvimento de três dos quatro sistemas a seguir: ^777^

• Renal (rápida deterioração da função renal ou proteinúria);
• Cardíaco (HVE importante ou disfunção sistólica, ou anormalidades da repolarização ventricular, ou aumento de troponina);
• Neurológico (AVE ou encefalopatia hipertensiva);
• Hematológico (hemólise microangiopática).

A definição da hipertensão MDO (na presença de acometimento de múltiplos órgãos-alvo) dispensa a presença de lesões fundoscópicas graus III ou IV de Keith-Wagener, que podem ocorrer tardiamente. ^778^ ^,^ ^779^ Ao comparar a hipertensão MDO com a hipertensão acelerada-maligna, observa-se que apresentam patogenia, significado clínico e prognóstico análogos, o que implica manejo clínico semelhante (GR: IIa; NE: B). ^777^ ^,^ ^780^

**Table:** 

Mensagens principais
Crise hipertensiva: elevação aguda da pressão arterial (PA) sistólica ≥ 180 mm Hg e/ou PA diastólica ≥120 mmHg, que pode resultar ou não em lesões de órgãos-alvo (LOA), que é dividida em urgência hipertensiva (elevação da PA sem LOA e sem risco de morte iminente. Isso permite a redução da PA em 24 a 48 h) e emergência hipertensiva (elevação da PA com LOA aguda ou em progressão e risco imediato de morte; requer redução rápida e gradual da PA em minutos a horas, com medicamentos intravenosos).
A emergência hipertensiva pode se manifestar como um evento cardiovascular, cerebrovascular, renal e com envolvimento de múltiplos órgãos ou também na forma de pré-eclâmpsia com sinal de gravidade/eclâmpsia.
A elevação da PA sem LOA aguda e progressiva afasta EH.
Hipertensão arterial mal controlada por falta de adesão, hipertensão de difícil controle, urgência hipertensiva e pseudocrise hipertensiva são situações comuns de elevação da PA sem LOA aguda ou progressiva.
A gravidade da condição clínica não é determinada pelo nível absoluto da PA, e, sim, pela magnitude e tempo de sua elevação. A definição numérica serve como um parâmetro, mas não deve ser usada como critério diagnóstico absoluto.

## 14. Hipertensão Arterial no Idoso

## 14.1. Introdução

A Organização das Nações Unidas (ONU) e a Organização Mundial da Saúde (OMS) consideram idoso todo indivíduo com 60 anos ou mais. Nos países de alta renda, onde a expectativa de vida é mais alta, o limite foi aumentado para 65 anos. ^781^ Existe uma faixa etária especial chamada de “muito idosos”, composta por aqueles com idade igual ou superior a 80 anos que representam o grupo de maior crescimento percentual populacional. ^782^

A prevalência de multimorbidade aumenta com a idade, de forma que mais de 2/3 dos muito idosos têm duas ou mais doenças crônicas. ^783^ ^,^ ^784^ Em estudo de base nacional da população idosa (ELSI-Brazil), mais de 60% tinham múltiplas doenças crônicas; e a hipertensão arterial (HA) foi a segunda mais prevalente, ficando atrás apenas da dor lombar crônica. ^785^ Esses pacientes geralmente recebem múltiplos fármacos em esquemas terapêuticos de difícil gerenciamento que aumentam o custo e o risco de interações medicamentosas.

Existe relação direta e linear da pressão arterial (PA) com a idade, sendo a prevalência de HA de aproximadamente 7% na população de 18 a 39 anos, chegando a mais de 60% na faixa etária acima de 65 anos. O estudo de Framingham demonstrou que quase 2/3 dos homens e 3/4 das mulheres apresentam HA aos 70 anos. ^786^ ^,^ ^787^

Embora estudos epidemiológicos tenham sugerido maior sobrevida em pessoas com 80 anos ou mais que apresentem níveis mais altos de PA, isso pode ser causado, em parte, pelo fato daqueles com PA baixa terem maior multimorbidade e fragilidade e, portanto, menor probabilidade de sobreviver. Na população geriátrica, a HA é o principal fator de risco (FR) modificável para morbidade e mortalidade cardiovascular, ^786^ mesmo nas idades mais avançadas. É fundamental ressaltar que a HA é FR modificável para declínio cognitivo, demência e perda de funcionalidade. ^787^ ^,^ ^788^

Na avaliação de sobrevida média em idosos, não se deve usar a expectativa de vida “ao nascer”, mas, sim, a expectativa de vida “em vida”. Dessa forma, as expectativas de vida ao alcançar 80 anos, em 2018, foram de 10,4 anos para mulheres e 8,6 para homens, tempo mais que suficiente para obter os benefícios com o tratamento da HA.

## 14.2. Mecanismos Fisiopatológicos

A pressão arterial diastólica (PAD) aumenta até cerca de 50 anos, estabiliza-se dos 50 aos 60 anos e diminui posteriormente, enquanto a pressão arterial sistólica (PAS) tende a aumentar durante toda a vida. Assim, a pressão de pulso (PP = PAS – PAD), um indicador hemodinâmico útil de rigidez vascular arterial, aumenta com a idade. Essas alterações são consistentes com a noção de que, em pessoas mais jovens, a PA é determinada em grande parte pela resistência vascular periférica (RVP), enquanto em indivíduos mais velhos se mostra determinada pela rigidez dos grandes vasos arteriais centrais. ^789^ ^-^ ^791^

O espessamento da parede arterial e a disfunção endotelial observadas durante o envelhecimento são acompanhadas de aumento na rigidez e redução na complacência vasculares, atribuídas a uma série de fatores, como maior sensibilidade ao sal, estresse hemodinâmico crônico, fragmentação e desalinhamento das fibras de elastina, com substituição por fibras colágenas, o que facilita a deposição de íons cálcio. ^792^

O enrijecimento aórtico, em decorrência do envelhecimento vascular, aumenta as velocidades de propagação da onda de pulso arterial (VOP) em direção à circulação periférica (centrífuga) e das ondas reflexas que retornam ao coração (centrípeta). A superposição dessas duas ondas na fase protomesossistólica causa o aumento da PAS e o alargamento da PP observadas nos idosos. ^793^

Atualmente, considera-se a medida da VOP carotídeo-femoral padrão-ouro para avaliar a rigidez arterial dos vasos centrais. Idosos com velocidades menores que 7,6 m/s, na ausência de comorbidades, são considerados portadores de boa saúde vascular e corresponderam, em amostra isolada, menos de 4% da população de indivíduos com mais de 60 anos. ^794^ ^,^ ^795^ Em determinada área urbana no Brazil, os valores da VOP encontrados em idosos, ajustados para a PA, idade e gênero, foram em média de 9,1 m/s para normotensos e de 9,4 m/s para os hipertensos não controlados. ^796^ Por outro lado, em muito idosos, a amplificação da PP pode ser melhor preditora de eventos e de mortalidade que a VOP. ^797^

## 14.3. Diagnóstico e Decisão Terapêutica

A investigação da HA nos idosos pode ser dificultada pela presença de múltiplas comorbidades e polifarmácia. As orientações do Capítulo 3 referentes à medida da PA e aos exames físico e laboratorial devem ser seguidas também para essa faixa etária. No entanto, a investigação de causas secundárias de HA deve ser conduzida com cuidado, avaliando-se os riscos e os benefícios de cada procedimento (ver Capítulo 15). ^798^

A avaliação clínica dos pacientes idosos e, especialmente, dos muito idosos difere da tradicional. Primeiramente, o médico deve reconhecer que a consulta exigirá um tempo maior, devido a vários fatores, como: complexidade das múltiplas condições associadas, lentidão física e cognitiva do paciente e presença de familiares e cuidadores, com os quais o médico deverá discutir pontos inerentes às condições clínicas e terapêuticas propostas. ^799^ Os muito frágeis podem necessitar de consultas adicionais, devido à exaustão do paciente. ^800^

Em face da maior variabilidade pressórica e de algumas peculiaridades, a medida da PA pode resultar em valores inexatos. Os principais fatores que interferem na medida da PA em idosos são: 1. hiato auscultatório; 2. pseudo-hipertensão; e 3. variações posturais e pós-prandiais. ^801^ (ver Capítulo 3)

A monitorização da PA fora do consultório, ambulatorial (MAPA) ou residencial (MRPA), é cada vez mais valorizada e indicada no diagnóstico da HAS no idoso. ^180^ ^,^ ^186^ A automedida da PA, apesar das limitações, também deve ser considerada (ver Capítulo 3).

O tratamento e o controle pressórico adequados da HA em idosos e muito idosos têm benefícios inequívocos, com redução expressiva de AVE, IAM, IC e mortalidade, ^87^ ^,^ ^509^ ^,^ ^572^ ^,^ ^802^ ^,^ ^803^ além da prevenção do declínio cognitivo e, provavelmente, de demência. ^103^ ^,^ ^804^ ^-^ ^806^ Por outro lado, os níveis exatos de PA considerados para tratamento nos idosos, assim como as metas com o tratamento, têm sido foco de debates, ^180^ com divergências entre diferentes diretrizes. ^5^ ^,^ ^37^ ^,^ ^807^ Todas, porém, assim como esta diretriz, consideram fundamental a avaliação individualizada. Recomenda-se que, até mesmo acima da idade cronológica, convém ponderar condição funcional, cognição, grau de fragilidade, expectativas do paciente, comorbidades, lesão de órgãos-alvo e risco CV global, polifarmácia e tolerabilidade ao tratamento. Os valores pressóricos recomendados em idosos, tanto para o início de tratamento quanto para as metas a serem obtidas estão descritos no Quadro 14.1 .

**Quadro 14.1:** – Recomendações para o tratamento da hipertensão em idosos

	PAS de consultório		PAD de consultório	
Condição global ^1^	Limiar de tratamento	Meta pressórica ^4^ ^, ^ ^5^	Limiar de tratamento	Meta ^8^
Hígidos ^2^	≥140 (I, A)	130-139 (I, A) ^6^	≥90	70-79
Idosos frágeis ^3^	≥160 (I, C)	140-149 (I, C) ^7^	≥90	70-79

1: mais importante a condição funcional que a idade cronológica; 2: incluindo fragilidade leve; 3: fragilidade moderada a severa; 4: incluindo idosos com comorbidades: DM, DAC, DRC, ACV/EIT (não se refere à fase aguda); 5: avaliar ativamente a tolerabilidade, inclusive possíveis sintomas atípicos: 6: a meta mais rígida (125-135 mmHg) pode ser obtida em casos selecionados, especialmente em idosos motivados, < 80 anos, apresentando ótima tolerabilidade ao tratamento; 7: limites mais elevados em caso de sobrevida limitada e ausência de sintomas. Redução da PA deve ser gradual; 8: PAD = evitar < 65-70 mmHg em portadores de DAC clinicamente manifesta. Obs.: o monitoramento fora do consultório (MAPA/MRPA) deve ser realizado às mudanças de terapia ou anualmente devido à maior variabilidade da PA com envelhecimento, maior risco de hipotensão ortostática e menor tolerabilidade ao tratamento inadequado da hipertensão do avental branco e mascarada.

## 14.4. Tratamento

A estratégia terapêutica no idoso, especialmente naqueles com mais de 80-85 anos, não pode ser única ( Quadro 14.2 ). Por isso, mais importante que a idade, deve-se considerar: a presença de comorbidades, a autonomia, o *status* funcional e o grau de fragilidade para planejar o tratamento (GR: I; NE: C). Tal estratificação é capaz de predizer melhor as possíveis complicações, no curto e no longo prazos, em diferentes comorbidades. ^808^ ^,^ ^809^ Nenhuma intervenção terapêutica deve ser negada ou retirada apenas com base na idade (GR: I; NE: C).

**Quadro 14.2:** – Desafios no tratamento da HAS no idoso.

A maioria dos idosos é de hipertensos, com alta prevalência de HSI.
Os desafios não se restringem à idade, mas principalmente às condições funcional, social, nutricional e mental.
A sobrevida está mais relacionada com o status funcional global que a idade per se.
O diagnóstico da HA no idoso requer o conhecimento de suas peculiaridades e o uso frequente de monitorização fora do consultório.
As dificuldades terapêuticas estão ligadas a adesão, presença ou não de polifarmácia, hipotensão ortostática e comorbidades, como incontinência urinária e fadiga, entre outros, comuns nos idosos.
A avaliação clínica deve incluir testes funcionais, como a velocidade de marcha e a Escala de Fragilidade Clínica.
O tratamento previne eventos CV, morte e declínio cognitivo, mesmo em idades avançadas.
As MEV funcionam, mas exigem um cuidado maior.
DIU, BCC, IECA/BRA devem ser usados em monoterapia ou em combinação, como terapia inicial; BB, quando houver indicação formal.
A perda de peso e de reserva orgânica nas idades avançadas pode estar associada à diminuição gradativa da PA e implicar a desintensificação do tratamento.
Em idosos em cuidados paliativos por doenças avançadas ou fragilidade severa, o objetivo principal do tratamento é o controle de sintomas.

BB: betabloqueadores; BC: bloqueadores dos canais de cálcio; BRA: bloqueadores dos receptores AT1 da angiotensina II; CV: cardiovasculares; DIU: diuréticos; HA: hipertensão arterial; HSI: hipertensão sistólica isolada; IECA: inibidores da enzima conversora da angiotesina; MEV: mudanças de estilo de vida.

### 14.4.1.Tratamento Não Medicamentoso

Todas as medidas de mudanças de estilo de vida (MEV) que se aplicam no indivíduo jovem (ver capítulo 8) são válidas para o idoso (GR: I; NE: B), porém convém um cuidado maior e considerar o real benefício – e potencial risco – de cada uma. Os idosos são mais sal-sensíveis, sendo a restrição salina mais eficaz nessa faixa etária. ^420^ O estudo TONE mostrou que, para cada 80 mmol de sódio (=2,0 g de sal) de diminuição de consumo de sal por dia, houve a redução de 4,3 mmHg na PAS e de 2,0 mmHg na PAD. Quando ocorreu queda simultânea de peso, o efeito de redução da PA foi potencializado. ^810^ A diminuição excessiva da ingestão de sal pode induzir hiponatremia e perda de apetite, levando a desnutrição. As dietas ricas em potássio devem ser incentivadas, ^811^ porém convém ter maior cautela com o risco de hipercalemia, devido à presença frequente de doença renal crônica (DRC) e do uso de fármacos que reduzem a excreção de potássio.

Exercícios físicos, aeróbicos e resistidos são fundamentais nos idosos e devem ser orientados. ^52^ Em idosos, especialmente nos frágeis ou sarcopênicos, a redução do peso sem exercício físico e do consumo adequado de proteínas pode reduzir a massa muscular e piorar a funcionalidade.

O tabagismo e o uso consumo inadequado de álcool ainda têm certa prevalência na idade avançada e devem ser abordados. Do mesmo modo, todos os medicamentos em uso pelo paciente devem ser revisados, pois alguns deles podem elevar a pressão.

Na indicação de MEV, o médico deve levar em consideração a presença e o grau de fragilidade, a capacidade funcional e demais aspectos clínicos e sociais do paciente. O acompanhamento por equipe multidisciplinar (ver Capítulo 7) e o envolvimento de seus familiares/cuidadores são ainda mais importantes nos pacientes idosos.

### 14.4.2. Tratamento Farmacológico

Na escolha do(s) anti-hipertensivo(s) para idosos, devem-se considerar elevada prevalência de comorbidades, contraindicações específicas, prováveis interações medicamentosas e custo, bem como a disponibilidade do fármaco e e a experiência clínica com o mesmo (GR: I; NE: C). É prudente iniciar com monoterapia ou combinação em doses baixas e, se necessário, realizar aumento ou combinação gradual de anti-hipertensivos, com intervalo mínimo de duas semanas (GR: I; NE: C).

No Capítulo 9, estão descritos os detalhes sobre quando dar preferência ou evitar determinados anti-hipertensivos e sobre suas combinações. Destacamos aqui as peculiaridades para o idoso.

O anti-hipertensivo inicial pode ser um diurético tiazídico (ou similar), um bloqueador dos canais de cálcio (BCC) ou um bloqueador do sistema renina-angiotensina-aldosterona (SRAA): inibidor da enzima conversora da angiotensina (IECA) ou bloqueador do receptor AT _1_ da angiotensina II (BRA). As quatro classes têm um grande número de estudos clínicos e são referência em diretrizes de idosos. ^807^ ^-^ ^809^ As indicações e cuidados no monitoramento são, em geral, semelhantes aos adultos (ver Capítulo 9).

Os betabloqueadores (BB) não devem ser utilizados como monoterapia inicial em idosos, ^809^ exceto na presença de algumas comorbidades, em que podem ter, inclusive, indicação obrigatória, como insuficiência cardíaca (IC) ou insuficiência coronariana aguda (GR; I; NE: A). ^812^ ^,^ ^813^ Pacientes com asma brônquica ou doença pulmonar obstrutiva crônica (DPOC), mas com indicação clínica para o uso de BB, devem ser tratados com BB cardiosseletivo de forma cuidadosa e após compensação respiratória, não devendo ser privados dos benefícios do medicamento. ^814^ Quando usados em combinação com fármacos inibidores da acetilcolinesterase, comumente utilizados na doença de Alzheimer, podem induzir bradicardia severa. ^815^

Outras classes de anti-hipertensivos (medicamentos de ação central, antagonistas da aldosterona e vasodilatadores diretos), bem como outras modalidades de tratamento invasivo no sistema simpático, devem ser vistas como exceção e não habituais para o tratamento do idoso (GR: III; NE: C) (ver Capítulo 9).

O risco de quedas em idosos pode aumentar nas primeiras semanas de tratamento com o uso dos DIU e, no primeiro dia, com as demais classes. A longo prazo, o uso de anti-hipertensivos pode até mesmo ter um efeito protetor. ^816^ ^,^ ^817^

## 14.5. Situações Especiais

Existe certa divergência de resultados entre estudos observacionais e ensaios clínicos randomizados (ECR). Isso se deve, principalmente, ao fato de os idosos frágeis e multimórbidos estarem pouco representados nos ECR e pelo alto risco de vieses nos estudos observacionais e não randomizados, nos quais a melhor sobrevida naqueles com PA elevada pode ser explicada porque estes apresentam melhor reserva orgânica. ^818^ ^-^ ^822^

### 14.5.1. *Status* Funcional e Fragilidade: Avaliação e Implicações

Em idosos e, especialmente nos muito idosos, deve-se ter atenção especial ao *status* funcional e à fragilidade. A Avaliação Geriátrica Ampla (AGA), utilizando escalas e testes de forma sistematizada, possibilita identificar com precisão a condição do global do idoso e montar estratégias para a abordagem terapêutica. ^823^ ^,^ ^824^ Apesar de ser a forma ideal de avaliação, pode ser necessária a presença de um geriatra ou um gerontólogo. Na assistência cotidiana do hipertenso idoso, o clínico deve realizar a avaliação da funcionalidade e da capacidade para a execução das atividades de vida diária. ^825^ ^,^ ^826^

Recomenda-se o uso rotineiro do teste funcional da velocidade de marcha (VM) por ser facilmente aplicado na rotina de atendimento e por ter demonstrado clara discriminação prognóstica de sobrevida. ^827^ ^,^ ^828^ São considerados como frágeis ou em risco de fragilidade aqueles com VM < 0,8 m/s (ou incapacidade de caminhar 6 metros em menos de 8 segundos), devendo-se aprofundar a investigação. ^820^ ^,^ ^829^ Para complementação, sugere-se utilizar a “Escala Clínica de Fragilidade” já traduzida e validada para o Brazil, ^830^ a partir da *Clinical Frailty Scale* do Canadá, amplamente testada e utilizada, por sua simplicidade, sua confiabilidade e sua visão global do paciente, além de determinar o prognóstico. ^827^ ^,^ ^828^ ^,^ ^831^ ^,^ ^832^

A fragilidade está associada a maior risco de HA, doença subclínica, eventos CV e mortalidade. ^821^ ^,^ ^833^ ^-^ ^835^ O controle adequado da HA pode influenciar a trajetória da fragilidade. Por outro lado, graus avançados de fragilidade estão associados a menores valores de PA, menor índice de massa corporal (IMC), menor massa muscular, pior cognição e maior mortalidade. ^335^ ^,^ ^836^

Aqueles funcionalmente ativos, independentes e sem comorbidades graves apresentam reserva orgânica e sobrevida média suficientes para obter os maiores benefícios de tratamento anti-hipertensivo e devem, desde que bem toleradas, ter metas pressóricas semelhantes aos idosos mais jovens (GR: I; NE: B). ^335^ ^,^ ^805^ ^,^ ^825^ ^,^ ^831^ No outro extremo, aqueles com perda funcional importante, sarcopenia, fragilidade ou demência avançada e incapacidade para as atividades de autocuidado, todo o tratamento deve ser reavaliado e as metas, pressóricas revistas. ^821^ ^,^ ^825^ ^,^ ^831^ ^,^ ^837^ O objetivo primordial é o de melhora de sintomas e de qualidade de vida. Os idosos mais frágeis foram sistematicamente excluídos dos diversos estudos clínicos, sendo, portanto, fundamentais estudos clínicos específicos para tal população. ^307^ ^,^ ^821^

Entre esses extremos, estão os idosos em situação funcional intermediária, com múltiplas comorbidades não CV, que podem gerar grandes desafios para a decisão terapêutica. Neles, uma avaliação mais aprofundada pode ser fundamental para definir o real risco/benefício e a individualização das várias estratégias terapêuticas. ^307^ ^,^ ^825^ ^,^ ^831^ ^,^ ^838^ ^,^ ^839^

### 14.5.2. Declínio Cognitivo e Demência

Além de bem estabelecida como principal causa de AVE, a HA também foi implicada como fator patogênico no comprometimento cognitivo, tanto de origem vascular quanto da doença de Alzheimer, que são as principais causas de demência no idoso, de forma mais marcante a longo prazo. ^840^ ^-^ ^842^

Em vários estudos epidemiológicos, o uso de anti-hipertensivos esteve associado a menor declínio cognitivo e demência, especialmente no longo prazo. ^843^ Os ECR mostraram redução de lesão de substância branca e do declínio cognitivo com tratamento da HA, sendo o tratamento intensivo ainda mais eficiente. ^5^ ^,^ ^37^ ^,^ ^844^ A redução de quadros demenciais ainda não foi claramente comprovada por ECR. Isso pode ocorrer porque nos ECR a cognição não era o desfecho primário, pela falta de uniformidade na definição de demência e nos testes utilizados, assim como pela curta duração dos estudos. ^806^ ^,^ ^845^

### 14.5.3. Polifarmácia e Adesão

A polifarmácia, definida pelo uso regular de cinco ou mais medicações, é mais frequente quanto maior a idade. ^846^ Está associada a maior chance de eventos adversos (EA), interações farmacológicas e piora na adesão ao tratamento. ^845^

A falta de adesão ao tratamento farmacológico mostra-se um problema frequente nos idosos e uma das principais causas do controle inadequado da PA. Alguns determinantes da má adesão à terapêutica instituída são a baixa compreensão da doença, a polifarmácia, as inúmeras tomadas diárias e os efeitos colaterais. ^847^ Nesse sentido, recomendamos, especialmente nos idosos sob polifarmácia, a revisão periódica de cada um dos fármacos em uso, a avaliação de EA ^848^ e que o tratamento anti-hipertensivo apresente o menor número possível de comprimidos ao dia, com a utilização de anti-hipertensivos em combinações fixas de dose única diária, além da ênfase às medidas não farmacológicas (GR: I; NE: A) (ver Capítulo 17).

### 14.5.4. Desintensificação e Desprescrição

Em diferentes situações clínicas, pode ser necessária a diminuição gradativa da dose ou até a suspensão de anti-hipertensivos, como: na hipotensão sintomática; em reações adversas; na PAS abaixo da meta de forma persistente detectada dentro ou fora do consultório; ^822^ ^-^ ^824^ na alteração da meta pressórica para valores menos rígidos (lembrando que a PA tende diminuir na idade muito avançada, resultante da diminuição progressiva da reserva orgânica e maior fragilidade); e nos cuidados paliativos no fim de vida. ^837^

Um ponto-chave no tratamento da HA em idosos e, especialmente, em muito idosos, é o monitoramento criterioso dos EA e da tolerabilidade, com atenção aos sinais e sintomas atípicos. A descontinuação de anti-hipertensivos parece ser segura a curto prazo, porém sem benefícios demonstrados na cognição ou na funcionalidade para atividades de vida diária (AVD). ^838^ ^,^ ^849^ ^,^ ^850^

### 14.5.5. Hipotensão Ortostática e Pós-prandial

Devido à rigidez arterial, as variações de volume interferem de forma significativa no controle da HA. Os idosos têm menor resposta dos barorreceptores à hipotensão; por isso, são mais propensos a hipotensão ortostática (HO) e a hipotensão pós-prandial (HPP). Associa-se a isso maior frequência de doenças neurodegenerativas. ^851^ Em torno de 20% dos idosos apresentam HO e aproximadamente 30% dos idosos institucionalizados têm hipotensão após as refeições. ^852^ ^,^ ^853^ Em razão diso, os idosos devem ser cuidadosamente monitorados para HO e HPP (GR: I; NE: B).

Em ECR, o controle da HA mostrou a diminuição de eventos CV sem aumento do no risco de HO ou quedas com lesão. ^854^ ^-^ ^856^ A HA mal controlada e determinados anti-hipertensivos, como os alfabloqueadores, podem provocar ou piorar a HO. A melhor opção para seu controle é o uso de medidas não medicamentosas como hidratação adequada, dieta normossódica, mudança lenta de decúbito, elevação da cabeceira e uso de meias elásticas. ^853^

Nos casos de hipotensão pós-prandial, o idoso deve evitar refeições copiosas e grande consumo de carboidratos e de álcool. Também não deve realizar exercícios após as refeições. Além disso, a prescrição farmacológica deve ser revisada, reduzindo-se ao máximo a polifarmácia e tendo atenção especial a medicamentos que possam estar contribuindo para a HO ou a HPP, como DIU, simpatolíticos, nitratos e antidepressivos tricíclicos.

**Table:** 

Mensagens principais
A prevalência de HA aumenta progressivamente com a idade, assim como de outros FR, elevando acentuadamente o risco CV entre os idosos.
Deve-se ter atenção às peculiaridades na medida da pressão para o diagnóstico correto, sendo que a avaliação da PA fora do consultório (AMPA, MRPA, MAPA) é fundamental no idoso, pelo maior risco no caso de tratamento inapropriado.
Os status funcional e cognitivo devem ser avaliados. A decisão terapêutica e a meta de PA devem se basear mais na condição funcional e na sobrevida do que na idade cronológica.
O tratamento reduz o risco CV e de declínio cognitivo. As comorbidades, que se apresentam em maior frequência no idoso, devem nortear os fármacos de escolha ou a ser evitados.
Um atenção especial deve ser direcionada à rede de suporte familiar, à presença de polifarmácia, à adesão e ao maior risco de HO.

## 15. Hipertensão Arterial Secundária

## 15.1. Introdução

**Figure 15.1.**
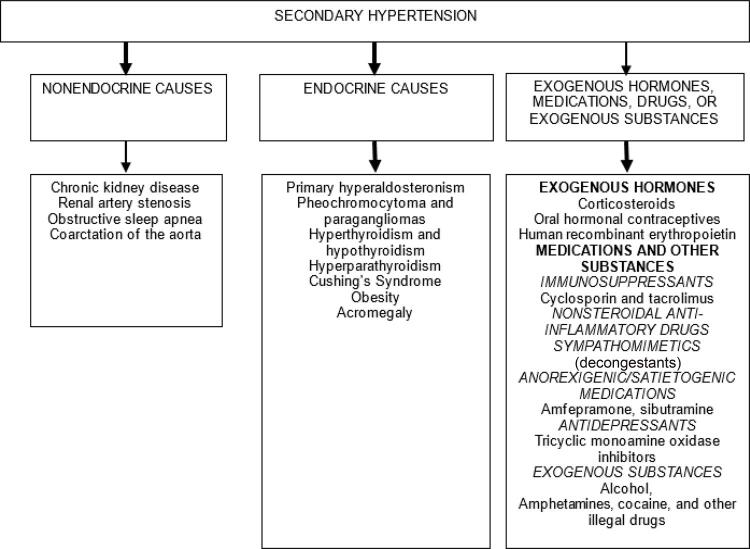
– Causes of secondary HT: nonendocrine, endocrine, and due to use of exogenous hormones, medications, drugs, or exogenous substances.

A hipertensão arterial secundária é a forma de hipertensão arterial (HA) decorrente de uma causa identificável, que pode ser tratada com uma intervenção específica, a qual determina a cura ou a melhora do controle pressórico. A real prevalência de HA secundária é desconhecida, mas se estima entre 10 e 20%, ^857^ podendo ser maior ou menor, conforme a população avaliada (especialmente idade), os recursos diagnósticos disponíveis e a experiência do médico responsável. Deve ser investigada diante de indícios (história clínica, exame físico ou exames de rotina) que levem à sua suspeita clínica ^258^ ^-^ ^860^ ( Quadro 15.1 ).

**Quadro 15.1:** – Indícios de hipertensão arterial secundária

1	Hipertensão estágio 3 antes dos 30 anos ou após os 55 anos
**2**	Hipertensão resistente ou refratária
**3**	Utilização de hormônios exógenos, fármacos ou demais substâncias que possam elevar a PA (ver Quadro 15.7 )
**4**	Tríade do feocromocitoma: crises de palpitações, sudorese e cefaleia
**5**	Indícios de apneia obstrutiva do sono
**6**	Fácies típica ou biótipo de doenças que cursam com hipertensão arterial
**7**	Presença de sopros em territórios arteriais ou massas abdominais
**8**	Assimetria ou ausência de pulsos em MMII
**9**	Hipopotassemia espontânea ou severa induzida por diuréticos (< 3,0 mEq/L)
**10**	Exame de urina anormal (hematúria glomerular (dismórfica) ou presença de albuminúria/proteinúria), diminuição do RFG estimado, aumento de creatinina sérica ou alterações de imagem renal

As principais causas de hipertensão arterial secundária, que serão exploradas neste capítulo estão demonstradas na Figura 15.1 . A investigação diagnóstica pode ser direcionada pela idade do paciente e pelo tipo de indício Quadro 15.2 . Os portadores de HA secundária estão sob maior risco CV e renal e apresentam maior impacto nos órgãos-alvo, devido a níveis mais elevados e sustentados de PA, bem como por ativação de mecanismos hormonais e moleculares. ^859^ ^,^ ^861^

**Figura 15.1.**
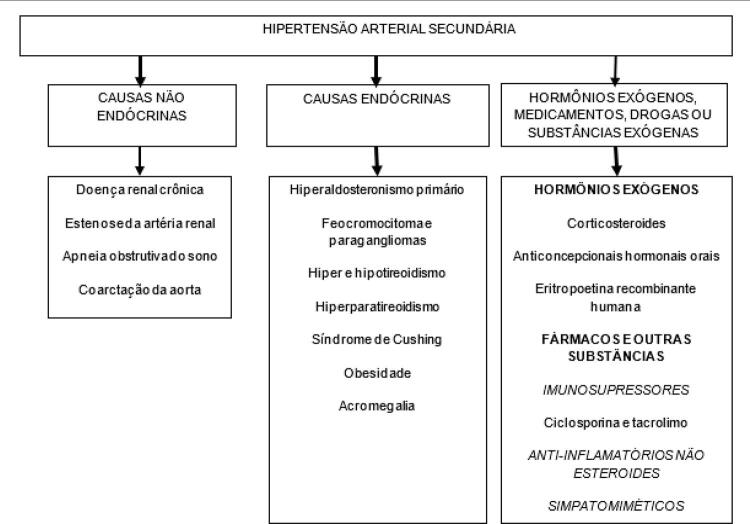
– Causas de HA secundária: não endócrinas, endócrinas e por uso de hormônios exógenos ou de medicamentos, drogas e substâncias exógenas.

**Quadro 15.2:** – Principais causas de HA secundária, não endócrinas e endócrinas, sinais indicativos e rastreamento diagnóstico

Achados clínicos	Suspeita diagnóstica	Achados adicionais
**Causas não endócrinas**		
**Edema, anemia, anorexia, fadiga, creatinina e ureia elevados e alterações do sedimento urinário ou nos exames de imagem**	Doença renal parenquimatosa	Creatinina e cálculo da RFG-e (I: B), US renal, exame sumário de urina (I: C) para pesquisa de proteinúria/hematúria dismórfica. Razão albuminúria ou proteinúria/creatininúria nos casos indicados (GR: I; NE: B)
**HA de aparecimento súbito ou com piora sem causa aparente antes dos 30 anos ou após 55 anos, HAR ou HA refratária ou HAM, sopro abdominal, EAP súbito, alteração da função renal inexplicável ou por medicamentos que bloqueiam o SRAA, assimetria entre o tamanho dos rins > 1,5 cm**	Estenose de artéria renal	US com Doppler renal com medida de velocidade de fluxo e índice de resistividade (rastreio, mas observador dependente) (GR: I; NE: B) e/ou renograma radioisotópico com captoprila (GR: III; NE: C), angiografia por RNM (RFG-e >30 mL/min, por subtração digital ou BOLD) (GR; I; GE: B) ou TC espiral (RFG-e > 60 mL/min (GR: I; NE: B) Padrão-ouro: arteriografia renal convencional (GR: I, NE: A)
**Maior frequência em homens ou mulheres pós menopausa, ronco na maioria das noites, fragmentação do sono com pausas ou engasgos, sonolência excessiva diurna, sono não reparador, fadiga, nicturia, cefaleia matinal, SM**	Apneia obstrutiva do sono (AOS)	Questionários apresentam baixa precisão para triagem Padrão-ouro: polissonografia ou poligrafia residencial. IAH< 5 eventos/h: sem AOS; IAH 5-14,9 eventos/h: AOS leve; IAH 15-29,9 eventos/h: AOS moderada; IAH ≥30 eventos/h: AOS importantes (ou grave)
**Fraqueza em MMII, pulsos em MMII ausentes ou de amplitude diminuída, HA com PAS 10 mmHg > em MMSS com relação aos MMSS, sopro sistólico interescapular e no tórax**	Coarctação de aorta	Radiografia de tórax, ecocardiograma para rastreio Angiografia de tórax por TC ou preferencialmente, RNM aorta (padrão-ouro). Angiografia invasiva, apenas quando necessários dados adicionais
**Causas endócrinas**		
**HAR ou HARf e/ou com hipopotassemia (não obrigatória) e/ou com nódulo adrenal**	Hiperaldosteronismo primário (hiperplasia ou adenoma)	Determinações de aldosterona (>15 ng/dL) e atividade/ concentração de renina plasmática; cálculo da relação aldosterona/renina > 30 Testes confirmatórios (ver Quadro 15.7 ) Exames de imagem: TC com cortes finos ou RNM. Coleta seletiva de aldosterona e cortisol em veias adrenais, para identificar subtipo, quando indicado (GR: I; NE: B)
**HA paroxística com a tríade composta por cefaleia, sudorese e palpitações**	Feocromocitoma e paragangliomas	Metanefrinas plasmáticas livres e/ou metanefrinas fracionadas urinárias (GR: I, NE: A). TC (GR: IIa, NE: B) (rastreamento), RNM (GR: I; NE: B) e cintilografia (GR: IIa, NE: C) nos casos indicados
**Fadiga, ganho de peso, perda de cabelo, HA diastólica, fraqueza muscular, sonolência**	Hipotireoidismo	Rastreamento: TSH e T4 livre (GR: I, NE: B)
**Intolerância ao calor, perda de peso, taquicardia/palpitações, exoftalmia, hipertermia, reflexos exaltados, tremores, bócio**	Hipertireoidismo	Rastreamento: TSH e T4 livre (GR: I; NE: B).
**Litíase urinária, osteoporose, depressão, letargia, fraqueza ou espasmos musculares, sede, poliúria, polidpsia, constipação**	Hiperparatireoidismo (hiperplasia ou adenoma)	Cálcio total e/ou iônico, fósforo, PTH, calciúria de 24h e dosagem de vitamina D (GR: I; NE: B)
**Ganho de peso, diminuição da libido, fadiga, hirsutismo, amenorreia, “fácies em lua cheia”, “giba dorsal”, estrias purpúreas, obesidade central, hipopotassemia**	Síndrome de Cushing (hiperplasia, adenoma e excesso de produção de ACTH)	Cortisol basal, cortisol salivar à meia-noite, cortisol urinário livre de 24h e teste de supressão com dexametasona ou betametasona: tomar dexametasona 1 mg às 23-24h e dosar cortisol matinal (7-8h) do dia seguinte. TC, RNM (GR: I; NE: B)
**Aumento da gordura central ou generalizada**	Obesidade classe 1: IMC de 30 a < 35 kg/m ^2^ classe 2: IMC de 35 a < 40 kg/m ^2^ classe 3: IMC ≥ 40 kg/m ^2^	IMC (peso em kg/altura em m2) e da circunferência abdominal (>102 cm homem e 88 cm mulher) Exames de imagem: DEXA (padrão-ouro), TC, RNM (estudos clínicos) (GR; I; NE: B)
**HA em até 30% dos casos, além de diabetes, HVE e AOS. Outros sintomas: defeitos visuais, paralisia de nervos cranianos, cefaleia, macrognatia, crescimento de pés e mãos, hipertrofia de tecidos moles, macroglossia, complicações musculoesqueléticas**	Acromegalia	Dosagem de IGF-1 (I, B), nível sérico de GH e GH pós sobrecarga oral de glicose (I, B) Localização: RNM de sela túrcica (preferencial) ou TC de sela túrcica

AOS: apneia obstrutiva do sono; HAR: hipertensão arterial resistente; HAM: hipertensão arterial maligna; RFG-e: ritmo de filtração glomerular estimado; EAP: edema agudo de pulmão; SRAA: sistema renina-angiotensina-aldosterona; TC: tomografia computadorizada; RNM: ressonância nuclear magnética; BOLD: blood oxygen level-dependent; DEXA: dual-energy x-ray absorptiometry scanning; IAH: índice de apneia e hipopneia; IMC: índice de massa corporal; ACTH: adrenocorticotropina; TSH: hormônio tireoestimulante; PTH: paratormônio; IGF-1: fator de crescimento insulina-símile tipo 1; GH: hormônio do crescimento; HVE: hipertrofia ventricular esquerda. MMII: membros inferiores; MMSS: membros superiores.

## 15.2. Causas Não Endócrinas

**Figure 15.2.**
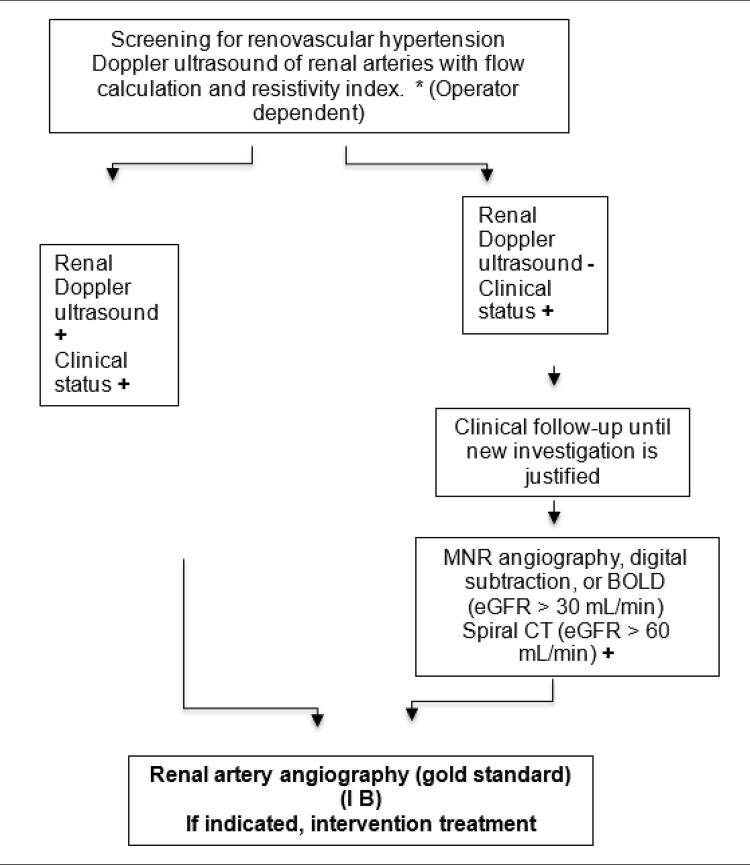
– Flow chart for investigation of patient suspected of having renal artery stenosis.

### 15.2.1. Doença Renal Crônica (DRC)

Define-se DRC por sua causa e por anormalidades de função ou morfologia persistentes por mais de três meses, com implicações para a saúde. Caracteriza-se por ritmo de filtração glomerular estimado (RFG-e) < 60 mL/min. ou alterações no exame de urina, especialmente albuminúria (30 mg/24 h ou razão albuminúria/creatininúria 30 mg/g), e/ou na morfologia renal (GR:I; NE: C). ^862^ A classificação e o prognóstico da DRC dão-se pelo RFG-e e pelos valores de albuminúria (capítulo 4). A HA é causa e consequência de DRC e aumenta progressivamente de acordo com o declínio da função renal, atingindo 90% dos pacientes em estágio 5 (GR: I; NE: A). ^863^ ^,^ ^864^

Os pacientes hipertensos devem dosar a creatinina sérica, acompanhada do cálculo do RFG-e (GR: I; NE: B) e realizar um exame de urina (GR: I; NE: C) para o rastreamento de DRC. ^859^ A ultrassonografia renal, a tomografia computadorizada (TC) ou a ressonância nucler magnética (RNM) podem ser necessárias. Reserva-se a biópsia renal quando há, além da HA, declínio rápido do RFG-e ou presença de hematúria glomerular e/ou proteinúria/albuminúria. ^865^ A HA acelera a progressão da DRC, e a redução da PA atenua sua evolução. ^863^ ^,^ ^864^ As metas de tratamento e a terapêutica indicada para o controle da PA em pacientes com DRC estão descritas nos Capítulos 6 a 9.

### 15.2.2. Hipertensão Renovascular (HARV)

A HARV é causa comum e potencialmente reversível de HA secundária, devido a uma estenose parcial ou total, uni ou bilateral da artéria renal (EAR) ou de seus ramos, desencadeando e mantendo isquemia renal significante. Isso geralmente ocorre com obstruções superiores a 70%. ^164^ Sua prevalência e sua etiologia variam conforme a idade e o nível pressórico. Em adultos jovens, especialmente do sexo feminino, a HARV é mais frequentemente causada por displasia fibromuscular (DFM). Em idosos, a causa mais comum consiste em aterosclerose, geralmente acompanhada por aterosclerose periférica e/oucoronariana. ^866^ Os indicadores clínicos de HARV são apresentados no Quadro 15.3 .

**Quadro 15.3:** – Indicadores clínicos de hipertensão renovascular

Início de hipertensão arterial antes de 30 anos
Início de hipertensão arterial grave após 55 anos, associada à doença renal crônica e à insuficiência cardíaca congestiva
Hipertensão arterial e sopro abdominal
Hipertensão arterial com piora rápida e persistente em paciente previamente controlado
Hipertensão arterial resistente ou refratária
Crise hipertensiva com lesões em órgãos-alvo (insuficiência renal aguda, insuficiência cardíaca congestiva, encefalopatia hipertensiva, retinopatia hipertensiva grau 3-4)
Piora da função renal após tratamento com bloqueadores do sistema renina angiotensina
Atrofia renal não justificada, assimetria renal ou piora de função renal inexplicada
Edema agudo de pulmão

Aboyans et al., 2018.867

Em pacientes com potenciais sinais de HARV, recomenda-se considerar testes diagnósticos em pacientes com menos morbidades nos quais esteja indicado o tratamento de revascularização. ^867^ ^,^ ^868^ As manifestações clínicas da doença renovascular são heterogêneas. Muitas lesões evoluem com repercussão hemodinâmica mínima e são silenciosas, até progredirem para níveis críticos associados a ativação de mecanismos fisiopatológicos hipertensivos e de isquemia renal. Os procedimentos de revascularização são indicados para os portadores de DFM e para os portadores de etiologia aterosclerótica que não conseguem controlar a PA ou tenham perda progressiva da função renal ou descompensação clínica cardiológica (edema agudo de pulmão, insuficiência cardíaca e angina refratária). ^869^ Uma investigação custo-efetiva sugere a seleção apropriada do candidato e exames de avaliação anatômica e funcional da estenose. ^866^

O padrão-ouro ainda é a arteriografia renal convencional, porém ela se mostra invasiva e não deve ser utilizada como procedimento inicial (GR: I; NE: B). A angiografia por RNM por subtração digital ou método BOLD (GR: II; NE: B) ou a TC espiral têm igual acurácia e maior sensibilidade e especificidade que a ultrassonografia (GR: I; NE: B). A US com Doppler renal é o método não invasivo recomendado para o rastreamento com sensibilidade e especificidade estimadas respectivamente em 75% e 90%. ^267^ ^,^ ^869^ ^-^ ^871^

Os objetivos do tratamento da HARV são a redução da morbidade e da mortalidade associadas à elevação da PA e a proteção da circulação e da função dos rins. Estudos clínicos randomizados ^872^ e metanálise ^873^ demonstraram ^874^ ^,^ ^875^ que o tratamento medicamentoso é igual ao da revascularização, com taxas similares de controle da PA e mortalidade cardiovascular.

Recomenda-se a utilização de fármacos que bloqueiam o SRAA para reduzir a hiperfiltração no rim contralateral e a proteinúria na HARV unilateral com monitorização adequada do potássio e da creatinina. A eficácia da otimização farmacológica é importante elemento para a decisão sobre a indicação de procedimento invasivo. ^874^ A HARV aterosclerótica demanda mudança do estilo de vida, cessação do tabagismo, controle glicêmico e prescrição de estatinas e antiagregantes, a menos que contraindicados. ^874^ ^,^ ^876^

Se a meta pressórica não puder ser alcançada e/ou outras condições clínicas estiverem associadas, como HAR ou HARf, disfunção renal progressiva, episódios de EAP, o procedimento invasivo pode ser recomendado, com a devida concordância do paciente. O real benefício do tratamento invasivo é controverso, e ensaios clínicos são necessários para identificar a população específica que se beneficiaria com este tipo de tratamento. ^877^ ^,^ ^878^ As recomendações para diagnóstico de doença renovascular são apresentadas no Quadro 15.4 .

**Quadro 15.4:** – Recomendações para diagnóstico de doença renovascular

Recomendação	GR	NE
Doppler de artérias renais (triagem), tomografia computadorizada espiral, angiografia por ressonância magnética nuclear	I	B
A angiografia por subtração digital ou BOLD pode ser indicada para confirmar o diagnóstico de estenose de artéria renal detectada por outros métodos em paciente com alta probabilidade de doença renovascular	IIB	C
Cintilografia renal, dosagem de renina antes e pós-administração de captoprila e dosagem de renina em veia renal não indicados para o screening de estenose de artéria renal	III	C

Aboyans et al., 2018. ^867^

## 15.3. Displasia Fibromuscular

A displasia fibromuscular (DFM) é uma doença idiopática, segmentar, estenosante, não aterosclerótica e não inflamatória da musculatura das artérias pequenas e médias. Tais lesões podem ser sintomáticas ou clinicamente silenciosas, hemodinamicamente significativas ou não. Aproximadamente 80 a 90% dos pacientes acometidos são do sexo feminino. O primeiro consenso internacional ^879^ recomenda uma classificação angiográfica em DFM focal e multifocal. O Doppler de artérias renais é o exame preconizado de rastreamento. Os demais exames de imagem coincidem com aqueles utilizados para a HARV de origem aterosclerótica: TC espiral, se RFG-e > 60 mL/min ou RNM, se RFG-e > 30 mL/min. ^879^ A angiografia das artérias renais é o padrão-ouro para a identificação da lesão na artéria renal. Recomenda-se mensuração do gradiente translesional para a determinação do significado hemodinâmico da estenose, sobretudo nas lesões multifocais. A identificação de outros segmentos vasculares acometidos pela doença e a pesquisa de aneurismas e dissecções são recomendáveis ^880^ ( Figura 15.2 ).

**Figura 15.2.**
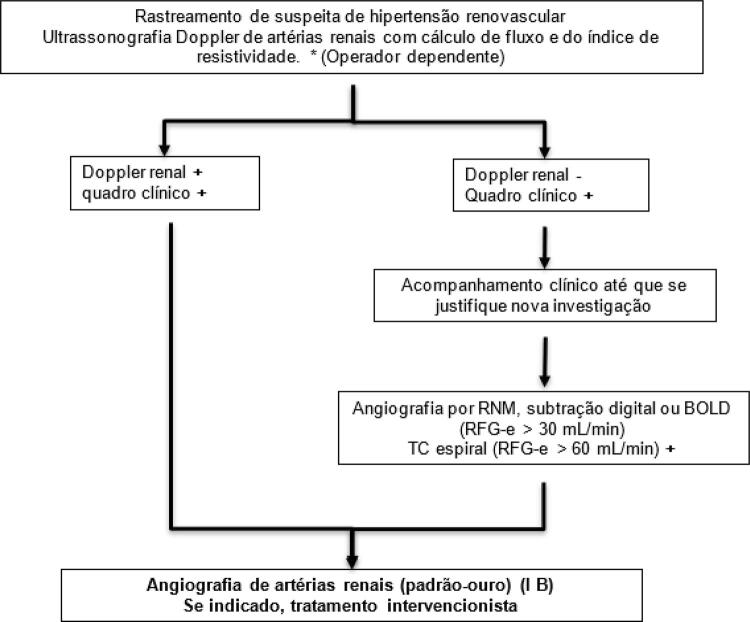
– Fluxograma de investigação do paciente com suspeita de estenose de artéria renal.

A angioplastia isolada é o procedimento indicado; e a utilização do *stent* , para os casos de complicações (dissecção ou ruptura arterial). Na ausência de contraindicação, a terapia contínua antiplaquetária com ácido acetilsalicílico na posologia de 75 a 100 mg/dia está indicada para a prevenção de complicações trombóticas, podendo por um período curto de quatro a seis semanas ser utilizada terapia antiplaquetária dupla. ^881^ Recomenda-se a realização do Doppler de artérias renais após 30 dias da angioplastia, repetindo a cada seis meses durante os primeiros dois anos e, depois, anualmente para detectar reestenose. ^879^ Todos os pacientes devem ser seguidos de rotina, a rigor, anualmente por avaliação clínica e de imagem.

### 15.3.1. Coarctação da Aorta

A coarctação da aorta é uma alteração congênita que leva a constrição da aorta geralmente justaductal, proximal ao canal arterial ou ao ligamento. Costuma ser subdiagnosticada, com apresentação clínica variada, desde sintomas precoces ao nascimento (crítica) até assintomática na fase adulta, ^882^ que depende da localização e da severidade da coarctação, bem como da presença frequente de outras doenças cardíacas congênitas que modificam seu prognóstico. ^883^ A definição de coarctação significante requer gradiente de pressão pré e pós-coarctação > 20 mmHg. A suspeita clínica baseia-se em sintomas (HA resistente ou refratária, epistaxes, cefaleia, fraqueza nas pernas aos esforços, manifestações de IC, angina, dissecção da aorta ou hemorragia cerebral) e no exame físico (presença de HA em membros superiores [MMSS] com PAS pelo menos 10 mmHg maior na artéria braquial com relação à artéria poplítea; ausência ou diminuição dos pulsos em membros inferiores [MMII]; sopro sistólico interescapular e no tórax). ^164^ O diagnóstico é feito por meio de exames de imagem: radiografia do tórax (aorta torácica com dilatações pré e pós-estenose, corrosão de costelas); ecocardiograma, principal exame de rastreio (protuberância posterior, istmo expandido, arco aórtico transverso e jato contínuo de alta velocidade no local da coarctação); e angiografia por TC ou RNM ^884^ quando a janela acústica for inadequada. A RNM é considerada o padrão-ouro para a avaliação e o seguimento pós-intervenção e, em adultos jovens, dispensa a realização da angiografia invasiva no pré-operatório, que está indicada quando as imagens de outros métodos não conseguem visualizar a coarctação, e em indivíduos mais velhos que podem ter DAC. O tratamento intervencionista inclui: angioplastia, implante de endoprótese vascular ou cirurgia aberta (hipoplasia do arco aórtico e/ou necessidade de ressecção da coarctação). A mortalidade operatória é muito baixa e o prognóstico mostra-se relativamente bom, embora pacientes portadores de coarctação da aorta tenham uma maior e mais precoce incidência de doenças CV que a população geral, devendo fazer acompanhamento contínuo. ^885^ A resposta da PA ao tratamento intervencionista depende da duração da HA antes da cirurgia e da idade do paciente. ^886^ Embora muitos reduzam a PA após o procedimento invasivo, uma grande proporção desenvolve HA exercício induzida. Os medicamentos anti-hipertensivos de escolha tanto para o periodo pré-operatório quanto para a PA residual após a cirurgia são os betabloqueadores (BB) e os inibidores da enzima conversora de angiotensina (IECA) ou o bloqueador do receptor AT1 da angiotensina II (BRA). ^885^ ^,^ ^887^ Os bloqueadores do sistema renina-angiotensina-aldosterona (SRAA) devem ser utilizados com cuidado no pré-operatório para não ocorrer diminuição importante de fluxo sanguíneo distal à coarctação desencadeando lesão renal aguda. ^885^ ^,^ ^887^

### 15.3.2. Apneia Obstrutiva do Sono (AOS)

#### 15.3.2.1. Conceito e Epidemiologia

A AOS é uma condição clínica caracterizada pelo colapso intermitente das vias aéreas superiores durante o sono, acarretando obstruções totais (apneias) e parciais (hipopneias). ^888^ As pausas respiratórias levam a maior esforço respiratório e geram redução da pressão intratorácica que aumentam a pressão transmural do ventrículo esquerdo, as quedas cíclicas da saturação de oxigênio (hipóxia intermitente), a hipercapnia (usualmente discreta) e a fragmentação do sono. ^889^ Entre os mecanismos envolvidos com a HA, podemos citar a ativação do sistema nervoso simpático, a inflamação sistêmica, o aumento na produção de espécies reativas de oxigênio e a disfunção endotelial, entre outras. ^889^

Classicamente, dividimos a gravidade da AOS pela soma dos eventos de apneia e hipopneia (o chamado índice de apneia e hipopneia, IAH) determinados pelo exame objetivo do sono: IAH < 5 eventos/h: sem AOS; IAH 5-14,9 eventos/h: AOS leve; IAH 15-29,9 eventos/h: AOS moderada; e IAH ≥ 30 eventos/h: AOS importante (ou grave). A prevalência da AOS é alta na população geral e depende dos critérios diagnósticos adotados. Em adultos, acomete cerca de 9,6% das mulheres e 24,8% dos homens. ^890^ Nos pacientes com HA em geral, estima-se que 56% tenham algum grau de AOS. ^891^ ^-^ ^894^ Em hipertensos resistentes, a prevalência revela-se > 60% e é, provavelmente, a causa mais comumente associada à HA secundária, ^895^ não significando que ela seja a causa isolada na maioria dos casos. Embora tenhamos evidências de que pacientes com AOS normotensos evoluem com maior incidência de HA independente de outros fatores de risco, ^896^ ^,^ ^897^ na prática clínica a AOS frequentemente surge em um indivíduo previamente hipertenso. No entanto, isso não minimiza a importância da AOS: existem evidências de que a associação da AOS e HA está relacionada com a maior frequência de lesão de órgãos-alvo do que pacientes hipertensos sem AOS. ^895^ ^,^ ^896^

#### 15.3.2.2. Apresentação Clínica e Triagem da AOS no Contexto da HA

Na população geral, alguns fatores predisponentes, sintomas clínicos e sinais devem ser avaliados durante o exame do paciente e podem reforçar a suspeita diagnóstica de AOS ^888^ ( Quadro 15.5 ). A prevalência de AOS é duas a três vezes maior nos homens que nas mulheres, mas também comum entre as mulheres, sobretudo após a menopausa.

**Quadro 15.5:** – Frequência dos principais fatores de risco e de sintomas/sinais clínicos sugestivos da apneia obstrutiva do sono (AOS)

Características	Medidas
Fatores de risco	Razão de chances (odds ratio)
Sobrepeso vs. eutrófico	2,3-3,4
Obeso vs. eutrófico	4,0-10,5
Sexo masculino vs. feminino	1,7-3,0
Idade (a cada aumento de 10 anos)	1,4-3,2
Período pós-menopausa em mulheres	2,8-4,3
Sintomas clínicos e sinais	Prevalência, %
Sonolência excessiva, fadiga ou sono não reparador	73-90
Relato de ronco na maioria das noites	50-60
Pausas respiratórias e engasgos observadas por outra pessoa	10-15
Nocturia (2 ou mais vezes por noite)	30
Refluxo gastresofágico durante à noite	50-75
Cefaleia matinal	12-18

Adaptado de Gottlieb et al., 2020.888

No entanto, muitos desses sintomas e sinais podem ser menos destacados na HA. Por exemplo, a sonolência diurna muitas vezes não está presente na HA, especialmente nos pacientes com HA resistente. ^899^ Os questionários para triar a AOS população geral não apresentam um bom desempenho, sobretudo nos pacientes com HA resistente. ^900^ ^-^ ^903^ É importante mencionar que alguns achados no padrão da PA podem ajudar na triagem de pacientes com AOS. Dados recentes sugerem que as alterações do padrão de descenso noturno, especialmente a forma ascensão da PA (média da PA no sono mais alta do que na vigília), aumentam a chance para a presença da AOS em cerca de três a quatro vezes. ^904^

#### 15.3.2.3. Impacto do Tratamento da AOS sobre a PA

O tratamento considerado de escolha para AOS, especialmente nos casos moderados a grave, é o uso de um aparelho gerador de pressão positiva contínua na via aérea, o CPAP (derivado do inglês *continuous positive airway pressure* ”). ^888^ Outros tratamentos da AOS como o avanço mandibular, os exercícios orofaríngeos, a terapia posicional e as cirurgias são boas opções para casos menos graves e selecionados. ^905^

Em linhas gerais, o impacto do tratamento da AOS sobre a PA é modesto (em torno de 2 a 3 mmHg). ^906^ Esses resultados são parcialmente justificados por alguns fatores: 1) muitos estudos e metanálises misturaram pacientes normotensos, hipertensos controlados e hipertensos não controlados; ^906^ e 2) a adesão nem sempre adequada ao uso do CPAP. ^906^ Estudos randomizados mostraram que o impacto do tratamento da AOS sobre a PA é maior em pacientes com HAR (em torno de 5 mmHg em média), mas, em geral, não levam ao controle pressórico desses pacientes. ^907^ ^-^ ^909^ Um estudo mostrou que a presença de alteração do descenso noturno foi um preditor de melhor resposta do CPAP na redução da PA em pacientes com AOS. ^910^ Outro achado ainda pouco compreendido é que indivíduos com sonolência excessiva apresentam uma maior redução da PA. ^911^ A Quadro 15.6 detalha algumas características preditoras de melhor resposta pressórica com o CPAP. ^911^

**Quadro 15.6:** – Preditores de melhor resposta pressórica com o CPAP

Características clínicas
Pacientes com melhor adesão ao CPAP (usualmente > 4 horas por noite)
Pacientes com sonolência excessiva diurna
Pacientes com hipertensão resistente
Pacientes com alteração do descenso noturno da PA

#### 15.3.2.4. Tratamento Anti-hipertensivo em Pacientes Hipertensos com AOS

Até o momento, não há evidências conclusivas de que haja alguma classe de anti-hipertensivos preferencial para pacientes hipertensos com AOS. ^905^ Alguns pontos sobre este tópico merecem destaque:

1. O efeito de anti-hipertensivos, em geral, parece ser mais eficaz do que o CPAP na redução da PA, mas a associação de CPAP mais anti-hipertensivo tem alguns benefícios adicionais – particularmente na PA noturna; ^912^
2. O tratamento farmacológico da HA, embora mais eficaz do que o CPAP isoladamente, não melhora na maioria das vezes a gravidade da AOS e os sintomas com ela relacionados. Mesmo o efeito de alguns diuréticos e da restrição de sal sobre a gravidade da AOS (motivada pela teoria do deslocamento rostral de fluidos à noite favorecendo o colapso das vias aéreas superiores) ^913^ tem resultado muito modesto na severidade da AOS. ^914^ ^,^ ^915^

## 15.4. Causas Endócrinas

## 15.4.1. Hiperaldosteronismo Primário (HP)

A HA acompanhada de supressão da atividade da renina plasmática (ARP) e aumento da excreção de aldosterona caracteriza a síndrome de aldosteronismo primário. ^916^ O HP era considerado uma forma rara de HA secundária (1%) e, na atualidade, pode estar presente em 22% dos pacientes com HAR. ^917^ ^,^ ^918^ Gordon et al. observaram que a incidência de HP na população de hipertensos primários está entre 5 a 15%, provavelmente cerca de 12 %. ^919^ A hiperplasia adrenal cortical bilateral é a causa mais frequente de HP (50-60%), enquanto os adenomas produtores de aldosterona (APA) são responsáveis por 40% dos casos de HP. ^920^ O carcinoma adrenal cortical produtor de aldosterona e a hiperplasia adrenal cortical unilateral são causas mais raras de HP.

Os principais testes confirmatórios para HP são apresentados no Quadro 15.7 . ^920^ ^-^ ^924^ O fluxograma de investigação diagnóstica é apresentado na Figura 15.3 .

**Quadro 15.7:** – Testes confirmatórios para hiperaldosteronismo primário

Teste	Procedimento	Dosagens	Resultado	Desvantagens
**Teste da infusão salina**	Infusão de 2L de soro fisiológico 0,9% em 4h (iniciar entre às 8 e 9h30). Na posição sentada, apresenta maior sensibilidade para o diagnóstico de HP.	K+, Aldosterona (A), renina (R) no tempo 0 e após 4h.	Sentado: valores de A > 6,0 a 10 ng/dL ao final do teste são positivos. Valor de corte de maior sensibilidade/especificidade é 6,0 ng/dL. Deitado: valores de A > 6,8 a 10 ng/dL ao final do teste são positivos. Valor de corte de maior sensibilidade / especificidade é 6,8 ng/dL.	Efeitos colaterais: crise hipertensiva, hipervolemia e hipocalemia. Contraindicado em pacientes com HA grave, IC descompensada, insuficiência renal e hipocalemia grave.
**Teste da Captoprila**	Captoprila 50 mg oral com o paciente permanecendo sentado por 2h.	A, R, K+ e cortisol nos tempos 0, 1h e 2h.	Valor de A > 8,5-13,9 ng/dL ou supressão da A > 30%. Subtrair a queda percentual do cortisol da queda percentual da A.	Efeito colateral: hipotensão Teste seguro, mas com baixa reprodutibilidade. Indicado para pacientes com insuficiência renal.
**Teste da fludrocortisona**	Fludrocortisona 0,1 mg 6/6h por 4 dias.	Controle de K+ a cada 6h. Dosar A e R às 10h do 5o dia.	Positivo de A > 6 ng/dL com R suprimida.	Efeitos colaterais: Crise hipertensiva, hipervolemia e hipocalemia. Contraindicado em pacientes com HA grave, IC descompensada, insuficiência renal e hipocalemia grave. Considerado padrão-ouro, mas precisa ser realizado sob internação sendo pouco viável na prática clínica.
**Teste da furosemida intravenosa**	Administrar furosemida 40 mg EV e estimular deambulação por 2h.	Dosar A, R e K+ antes e após 2h de deambulação intermitente.	Positivo se APR <2 ng/mL/h ou Renina <13 uUI/mL.	Efeito colateral: hipocalemia e hipotensão. Vantagem: bem tolerado e de fácil execução, é ideal para os pacientes com contraindicação a sobrecarga de sódio.

HP: hiperaldosteronismo primário; A: aldosterona; APR: atividade plasmática de renina; R: renina.

O exame de imagem de melhor acurácia é a TC de cortes finos, e a RM não mostra vantagens. O cateterismo de veias adrenais com coleta sanguínea simultânea de aldosterona e cortisol tem por objetivo identificar a origem da secreção de aldosterona, sendo considerado o exame de maior acurácia na diferenciação dos diferentes subtipos de HP. Está indicado nos pacientes com adrenais normais ou com alterações bilaterais na TC.

Além disso, o cateterismo de veias adrenais está indicado nos pacientes com nódulos adrenais pequenos (<1,5 cm) e idade de diagnóstico da HA superior a 40 anos, devido à possibilidade de tratar-se de um adenoma não funcionante. ^920^ ^-^ ^924^ Trata-se de método invasivo e dependente de experiência do radiologista. O tratamento preferencial em APA é a adrenalectomia unilateral, preferencialmente laparoscópica, a menos que contraindicada.

Em HP por hiperplasia, o tratamento é realizado com antagonistas mineralocorticoides (espironolactona 50 a 400 mg/dia). ^920^ ^-^ ^924^ O alvo principal do tratamento medicamentoso deve ser o desbloqueio da renina (além do controle pressórico e da correção da hipcalemia), a fim de reduzir a incidência cumulativa de eventos cardiovasculares. ^925^

### 15.4.2. Feocromocitoma

Os feocromocitomas (FEO) são tumores de células cromafins do eixo simpático-adrenomedular secretores de catecolaminas. ^926^ De 10% a 15% são extradrenais (paragangliomas); 10%, bilaterais; e 15-20%, malignos (podendo variar de 2 a 50%, conforme o defeito genético). ^927^ A incidência de feocromocitoma e paraganglioma é de cerca de 0,6 casos por 100.000 pessoas-ano.

Os sintomas são a tríade clássica de cefaleia, sudorese profusa e palpitações com HAR/HARf ou paroxística (50%; picos hipertensivos alternados com momentos de PA normal). A concomitância da tríade clássica com crise hipertensiva tem sensibilidade de 89% e especificidade de 67% para o diagnóstico. ^926^

O diagnóstico de feocromocitoma ou paraganglioma requer a comprovação do excesso de liberação de catecolaminas e a documentação anatômica do tumor. O diagnóstico laboratorial baseia-se na dosagem dos metabólitos de catecolaminas no sangue e na urina. A elevação das metanefrinas plasmáticas livres (metanefrina e normetanefrina) tem sensibilidade de 97% e especificidade de 93% ^926^ (GR: I; NE: A), mas, devido a seu maior custo, indica-se metanefrina urinária isolada ou associada às catecolaminas urinárias (epinefrina, norepinefrina e dopamina). Embora menos sensível, os valores aumentados (> 2 vezes o limite superior da normalidade) das catecolaminas urinárias indicam alta probabilidade diagnóstica. ^242^ A dosagem de metanefrinas urinárias tem sensibilidade superior às catecolaminas urinárias e ao ácido vanilmandélico para o diagnóstico de FEO e paragangliomas (recomendação não graduada). ^928^

As situações clínicas de estresse agudo (doença aguda, sepse, IAM, IC descompensada) e o uso de antidepressivos tricíclicos, agentes antipsicóticos e levodopa, entre outros, podem cursar com o aumento das catecolaminas (normalmente < 2x o limite superior da normalidade). Tais fármacos devem ser suspensos 2 semanas antes das dosagens para evitar resultados falso-positivos. Os métodos de imagem para a localização são TC, preferencialmente (GR: 2; NE: B), ou RNM (FEO apresenta hipersinal em T2), com sensibilidade de 89% e 98% respectivamente, para tumores adrenais. ^929^ A RM é superior na identificação de paragangliomas ou doença metastática linfonodal (GR: I; NE: B). A cintilografia de corpo inteiro com 123I-MIBG ou 68Ga DOTATE-PET-CT é muito eficaz em localizar FEO e paragangliomas, doença metastática ou múltiplos tumores cromafins (GR: IIa; NE: C). ^930^ ^,^ ^931^

O tratamento preferencial é cirúrgico, minimamente invasivo (GR: I; NE: B), devendo-se fazer preparo pré-operatório com alfa-1-bloqueadores (doxazosina ou prazosina) e hidratação adequada com o aumento da ingesta oral de sódio por, pelo menos, duas semanas antes da cirurgia. ^932^ O tratamento medicamentoso crônico inclui alfa-1-bloqueadores, BB (apenas após início de alfa-1-bloqueadores, quando houver taquicardia sintomática), BCC, IECA e agonistas de ação central. ^932^ A crise hipertensiva paroxística do FEO é uma emergência e deve ser tratada com nitroprussiato de sódio ou fentolamina injetável e reposição volêmica, se necessária. ^926^

A remoção total e precoce da neoplasia proporciona, em geral, remissão total dos sintomas e cura da HA, além de prevenir a doença metastática. ^927^ ^,^ ^929^ Em FEO malignos, com metástases não passíveis de ressecção, indica-se terapia sistêmica com MIBG-131. A quimioterapia citotóxica está indicada na progressão de doença após uma dose cumulativa elevada de MIBG-131 ou na doença metastática que não capta MIBG. O ácido zoledrônico está indicado para reduzir a dor e o risco de fratura na doença óssea metastática. ^927^ ^,^ ^929^ O acompanhamento clínico, bioquímico e radiológico dos pacientes é essencial para a detecção de recorrências ou metástases na forma maligna e de outro tumor nas síndromes familiares. ^242^

### 15.4.3. Hipotireoidismo

Na maior parte das vezes, o quadro clínico do hipotireoidismo é inespecífico, com fadiga, sonolência e ganho de peso (discreto na maioria dos casos). Os pacientes com hipotireoidismo apresentam níveis baixos de tiroxina (T4) livre e elevação do hormônio tireotrófico (TSH), que são exames de rastreamento (GR: IIa; NE: B). ^933^ No hipotireoidismo subclínico, o T4 livre está normal e o TSH, elevado. No hipotireoidismo, existe um risco maior de HA diastólica. ^934^ O hipotireoidismo aumenta a resistência vascular e o volume extracelular, mas a elevação na PA costuma ser discreta (<150/100 mmHg).

### 15.4.4. Hipertireoidismo

O hipertireoidismo eleva o débito cardíaco em consequência do aumento do consumo periférico de oxigênio e do aumento da contratilidade cardíaca. ^935^ A HA sistólica é comum, mas a prevalência da HA depende da gravidade do hipertireoidismo. Ocorre fibrilação atrial em 10-20% dos pacientes com hipertireoidismo, sendo mais comum acima dos 60 anos de idade. ^936^ O quadro clínico é mais proeminente na doença de Graves (palpitação, perda de peso, exoftalmia, bócio, tremores de extremidades, pele quente e intolerância ao calor, entre outros sintomas) ou adenoma tóxico, enquanto pode ser mais oligossintomático em indivíduos idosos com bócio multinodular tóxico. O diagnóstico baseia-se na dosagem da tiroxina (T4) livre e do hormônio tireotrófico (TSH) (GR: IIa; NE: B). Tipicamente, o T4L está elevado com o TSH suprimido. ^937^ No hipertireoidismo subclínico, o T4 livre está normal e o TSH suprimido. A presença do anticorpo antirreceptor de TSH é diagnóstico da doença de Graves, mas pode estar ausente em aproximadamente 10% dos casos.

### 15.4.5. Hiperparatireoidismo Primário

A frequência de HA em pacientes com hiperparatireoidismo primário varia de 10 a 60%. ^938^ A maioria dos pacientes com hiperparatireoidismo primário é composta por assintomáticos, enquanto o restante pode apresentar poliúria e polidipsia, osteoporose, constipação, litíase renal e HA. Os mecanismos envolvidos na HA não estão definidos, não havendo uma correlação direta entre níveis de PTH e calcemia com a gravidade da HA. A HA no hiperparatireoidismo primário agrava-se com o comprometimento da função renal pela hipercalcemia. A investigação laboratorial é feita pela dosagem de calcemia (cálcio total e/ou iônico), fósforo, PTH e cálcio total em urina de 24h. ^939^ É importante dosar e repor vitamina D (principalmente se < 20 ng/dL) para excluir hiperparatireoidismo secundário a deficiência de vitamina D do hiperparatireoidismo primário normocalcêmico.

### 15.4.6. Síndrome de Cushing

A síndrome de Cushing iatrogênica (pelo uso de corticoide exógeno) é relativamente comum, ao contrário da síndrome de Cushing endógena que se mostra rara. Entre as causas endógenas, a doença de Cushing (adenoma hipofisário produtor de ACTH) é responsável por 85% dos casos, enquanto 15% dos casos são causados por tumores ou hiperplasia adrenal (causas ACTH independentes). A HA ocorre em 75-80% dos pacientes com síndrome de Cushing. Os mecanismos da HA são estímulo pelo cortisol da ação vasopressora das catecolaminas, ação do cortisol nos receptores mineralocorticoides e ativação do SRAA pelo aumento da produção hepática de angiotensinogênio. O diagnóstico laboratorial do hipercortisolismo é feito pela dosagem de cortisol basal (útil para excluir uso exógeno de dexametasona ou betametasona), cortisol salivar a meia-noite e cortisol em urina de 24h, além de teste de depressão com dexametasona 1 mg (tomar dexametasona 1 mg às 23h e dosar o cortisol sérico entre 7-8 h na manhã do dia seguinte). A investigação radiológica deve ser feita com TC de adrenal ou RM de hipófise nos casos de hipercortisolismo ACTH-dependente. Os exames de imagem só devem ser realizados após o diagnóstico clínico e laboratorial de hipercortisolismo. O tratamento do Cushing endógeno depende da etiologia do hipercortisolismo. A conduta pode ser cirúrgica ou medicamentosa. ^940^

### 15.4.7. Obesidade

A distribuição excessiva de gordura visceral é acompanhada de importantes alterações hormonais, inflamatórias e endoteliais. ^941^ Todos estes mecanismos ativam uma cascata de eventos que liberam citocinas e adipocinas, aumentam a resistência à insulina e determinam a hiperatividade do SRAA e do SNS, causando retenção de sódio e água, com consequente HA e aumento do risco CV e renal. Inúmeros estudos demonstraram uma estreita associação entre o aumento da PA e o ganho de peso. A estratégia de redução de peso (ver Capítulo 8) é uma recomendação fundamental para a diminuição da PA e do risco CV, bem como de doenças associadas, como a AOS. ^942^ ^,^ ^943^

Do ponto de vista prático, embora haja críticas por desconsiderar raça/etnia, idade, sexo e outros parâmetros, categoriza-se a obesidade, de acordo com o IMC (kg/m ^2^ ), em classe 1: IMC de 30 a < 35; classe 2: IMC de 35 a < 40; e classe 3: IMC ≥ 40. A medida da circunferência abdominal também pode ajudar no diagnóstico de obesidade central. Estudos complementares como bioimpedância e exames de imagem mais fidedignos e muito caros podem ser realizados, principalmente em estudos clínicos, como *dual-energy x-ray absorptiometry scanning* (DEXA), TC e RNM. ^944^ ^-^ ^945^

### 15.4.8. Acromegalia

A acromegalia, esporádica ou familiar, em aproximadamente 98% dos casos, gerada por adenomas hipofisários secretores do hormônio de crescimento (GH). O excesso de GH estimula a secreção hepática de *insulin-like growth factor-I* (IGF-1), que causa a maioria das manifestações clínicas.

São mais comuns entre os 30 e 50 anos e classificam-se como microadenomas (com menos de 1 cm) ou macroadenomas (com 1 cm ou mais). Mais de 70% dos tumores causadores de acromegalia são do segundo tipo. A HA pode ocorrer em aproximadamente 30% dos casos e é de natureza multifatorial, com componente de retenção hidrossalina, efeito direto antinatriurético do GH, hiperatividade do SRAA, do SNS e disfunção endotelial, além de disglicemia, hipertrofia ventricular esquerda (HVE) e AOS. Outros sintomas podem ser visualizados no Quadro 15.2 . ^947^

A avaliação laboratorial inicia-se com a dosagem de IGF-1 e GH séricos (GR: I; NE: B). Um valor muito baixo de GH (abaixo de 0,4 ng/mL) exclui o diagnóstico de acromegalia, especialmente se associado a nível sérico de IGF-1 normal. A dosagem de GH após sobrecarga de glicose (75 g) possibilita a demonstração da não supressão da secreção de GH (GR: I; NE: B). A dosagem de IGF-1 e o teste de supressão de GH após sobrecarga de glicose são também empregados para a avaliação de resposta ao tratamento. A RNM de sela túrcica é o melhor exame de imagem para a identificação do tumor e, se contraindicada, pode ser substituída por TC de sela túrcica (GR: IIa; NE: B). ^946^ ^-^ ^948^ O tratamento da acromegalia pode envolver procedimentos cirúrgicos, radioterapia e terapia medicamentosa com análogos da somatostatina, sendo que a octreotida, a lanreotida e a cabergolina estão disponíveis no Sistema Único de Saúde (SUS). ^949^

## 15.5. Causas Medicamentosas, Hormônios e Substâncias Exógenas

É causa relativamente comum e subestimada de agravamento ou mesmo indução de HA, frequentemente contornável ou reversível. Uma anamnese completa de todos os fármacos, drogas e suplementos em uso deve ser realizada em todo hipertenso. ^859^

Os mecanismos hipertensores são bastante diversos, por vezes multifatoriais, como retenção de volume (glicocorticoides, cetoconazol, anticoncepcionais orais, terapia andrógena, anti-inflamatório não esteroide [AINE]), hiperatividade simpática (descongestionantes, anfetaminas, Inibidor da monoamina oxidase [IMAO], antidepressivos, outros medicamentos utilizados em psiquiatria e cocaína, inibidores de calcineurina) e hiperatividade do SRAA (imunossupressores). ^950^ É boa prática clínica informar aos pacientes hipertensos quando os medicamentos associados podem resultar em piora do controle pressórico. ^951^

A inibição da angiogênese por inibição do fator de crescimento vascular endotelial é uma estratégia antineoplásica em uso em variados contextos oncológicos. Apresenta como comum efeito colateral elevações de pressão arterial, inclusive agudamente. ^952^ Os mecanismos envolvidos são ativação do sistema da endotelina, disfunção endotelial e rarefação capilar. Recomenda-se que a pressão arterial esteja abaixo de 140/90 mmHg para iniciar essa forma de tratamento e que seja monitorado o comportamento da pressão arterial durante a terapia ^953^ (ver Quadro 15.8 ).

**Quadro 15.8:** – Medicamentos, hormônios, substâncias exógenas ilícitas e lícitas relacionadas com o desenvolvimento ou agravamento de HA

MEDICAMENTOS		MECANISMOS
Imunossupressores (inibidores de calcineurina)	Ciclosporina e tacrolimo	Aumentam síntese de prostaglandinas e diminuem excreção de Na+, H2O e K+
**Anti-inflamatórios não esteroides (AINE) e analgésicos**	Inibidores da cicloxigenase 1 e 2 Acetaminofeno	Diminuição de prostaglandinas e retenção de Na+ e volume
**Simpaticomiméticos**	Descongestionantes nasais (efedrina, pseudoefedrina, fenilefrina	Estimulam sistema nervoso central
**Anorexígenos/sacietógenos**	Anfepramona, sibutramina	Aumentam secreção de noradrenalina
**Antidepressivos e fármacos de uso psiquiátrico**	Tricíclicos, inibidores da monoamino-oxidase (IMAO), lítio, fluoxetina, selegilina, carbamazepina, clozapina, buspirona, duloxetina, velafaxina e desvenlafaxina	Aumentam secreção de norepinefrina, causando hiperatividade simpática
**Antifúngicos**	Cetoconazol, anfotericina B	Retenção de volume
**Alcaloides do ergot**	Bromocriptina	
**Terapia antirretrovirais combinada (TARV)**		
**Antineoplásicos inibidores do VEGF (vascular endothelial growth fator)**	Axitinibe, bevacizumabe, ponatinibe, pazopanibe, regorafenibe, sorafenibe, sunitinibe	Disfunção endotelial e diminuição de óxido nítrico
**HORMÔNIOS EXÓGENOS**		
**Glicocorticoides**		Retenção de Na+ e volume
**Eritropoetina recombinante humana**		Alteração em produção e sensibilidade dos agentes vasopressores endógenos, ação vasopressora direta e remodelamento arterial
**Hormônios sexuais (terapia de reposição estrogênica (estrogênios conjugados e estradiol; anticoncepcionais orais)**		Estimulam a produção de angiotensinogênio
**Hormônio de crescimento (GH)**		Multifatorial
**SUBSTÂNCIAS EXÓGENAS**		
**Álcool**		Hiperatividade simpática
**Anfetaminas**		Hiperatividade simpática
**Cocaína**		Hiperatividade simpática
**Suplementos advindos de plantas**		
**Alcaçuz (liquorice)**		
**Ginseng**		
**Ginkgo biloba**		

**Table:** 

Mensagens principais
Na ausência de sinais clínicos sugestivos de hipertensão secundária em adultos, as indicações para avaliação adicional são hipertensão resistente e início precoce ou tardio de hipertensão arterial e/ou elevação súbita da PA.
As principais causas da hipertensão secundária não endócrina e endócrina, os sinais indicativos e os métodos de rastreamento e diagnóstico são apresentados na Tabela 15.2.
A causa mais comum de hipertensão secundária é o aldosteronismo primário (AP). A razão aldosterona/renina mostra-se o melhor teste inicial para a indicação de avaliação adicional de AP. A HA paroxística com a tríade composta por cefaleia, sudorese e palpitações ocorre no feocromocitoma
A estenose da artéria renal deve ser investigada quando ocorre o aumento da creatinina ≥ 50% após o uso de IECA ou BRA. A HA grave de início recente ocorre em indivíduos > 55 anos, com sopro abdominal e diferença de tamanho do rim contralateral > 1,5 cm. A HA é grave em pacientes com aterosclerose ou edema pulmonar recorrente. Em adultos jovens com HA grave, deve ser pesquisada displasia fibromuscular da artéria renal.
Outras causas de hipertensão secundária requerem métodos, de diagnóstico específico, conhecimento especializado e experiência na interpretação de resultados. O tratamento deve ser orientado por especialistas em centros de referência.

## 16. Hipertensão Arterial Resistente e Refratária

## 16.1. Definição e Classificação

**Figura 16.1:**
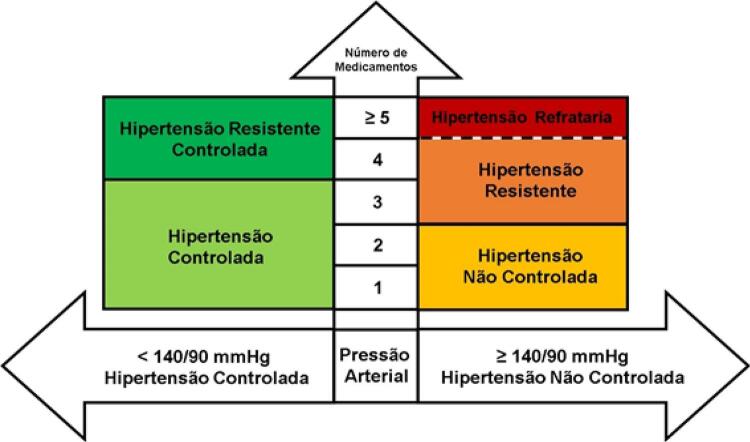
– Classificação da hipertensão arterial de acordo com o número de medicamentos anti-hipertensivos e controle da pressão arterial.

Define-se hipertensão arterial resistente (HAR) como a PA de consultório que permanece com valores ≥140/90 mmHg, com o uso de três ou mais classes de fármacos anti-hipertensivos com ações sinérgicas, em doses máximas preconizadas ou toleradas, sendo um deles preferencialmente um diurético tiazídico. Quando o paciente necessita do uso de quatro ou mais fármacos anti-hipertensivos para alcançar o controle da PA, ele também é considerado um hipertenso resistente, porém controlado (PA < 140/90 mmHg) ( Figura 16.1 ). ^164^ ^,^ ^504^ ^,^ ^564^ ^,^ ^954^

**Figura 16.2:**
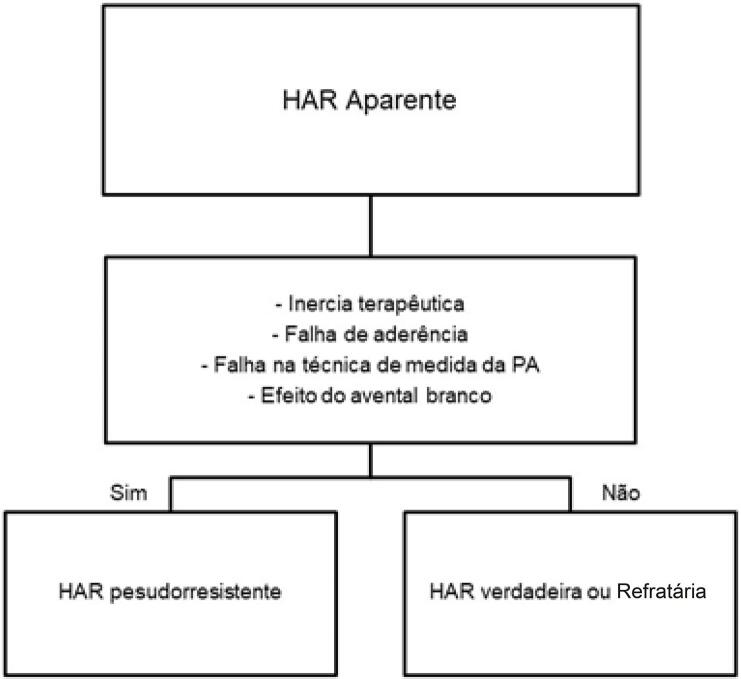
– Classificação da hipertensão arterial resistente.

Já a hipertensão refratária (HARf) é definida como um subgrupo de pacientes com HAR verdadeira, que mantém a PA não controlada (PA ≥ 140/90 mmHg), mesmo estando em uso de cinco ou mais fármacos anti-hipertensivos, incluindo a espironolactona e um diurético de longa ação ( Figura 16.1 ). ^955^ Define-se a hipertensão arterial pseudorresistente como a falha no controle da PA relacionada com hipertensão do avental branco, falha na técnica de verificação da PA, inércia terapêutica ou falha na adesão ao tratamento farmacológico e não farmacológico propostos ( Figura 16.2 ). ^164^ ^,^ ^504^ ^,^ ^564^ ^,^ ^954^ A identificação dos pacientes com HAR verdadeira impõe, portanto, o afastamento da pseudorresistência e das condições a ela associadas ( Figura 16.2 ), sendo fundamental para estabelecer abordagens específicas. ^164^ ^,^ ^504^ ^,^ ^564^ ^,^ ^954^

Adaptado de: Malachias et al., 2016; ^164^ Carey et al., 2018; ^954^ Yugar-Toledo, 2020; ^504^ Krieger et al., 2018. ^564^ HAR: hipertensão arterial resistente.

## 16.2. Epidemiologia da Hipertensão Arterial Resistente

Em estudos populacionais, a prevalência de HAR está estimada entre 12 a 15% da população hipertensa. ^164^ ^,^ ^504^ ^,^ ^564^ ^,^ ^954^ No Brazil, o estudo multicêntrico ReHOT ^564^ encontrou uma prevalência de 11,7% de HAR. A HARf corresponde a 3,6% dos hipertensos resistentes. ^164^

As principais características clínicas e condições associadas aos portadores de HAR estão destacadas no Quadro 16.1 . ^164^ ^,^ ^504^ ^,^ ^564^ ^,^ ^954^ ^,^ ^956^ A piora no prognóstico desses pacientes está associada especialmente aos seguintes fatores: exposição prolongada a níveis pressóricos elevados, danos em órgãos-alvo, excesso de mineralocorticoides (aldosterona) e consumo elevado de sódio. ^164^ ^,^ ^504^ ^,^ ^564^ ^,^ ^954^ ^,^ ^956^

**Quadro 16.1:** – Características clínicas e condições associadas a HAR

Características clínicas	Condições associadas
• Idade mais avançada • Afrodescendentes • Obesidade • PAS mais elevada • Não dipper na MAPA • Hipervolemia (mesmo em uso de diuréticos) • Ingestão excessiva de sal • Sedentarismo • EAB (30%)	• Presença de HVE • DM • Síndrome metabólica • IRC • Albuminúria

Adaptado de: Malachias et al., 2016; ^164^ Carey et al., 2018; ^954^ Yugar-Toledo, 2020; ^504^ Krieger et al., 2018; ^564^ Gaddan et al., 2008; ^957^ Shimosawa, 2013. ^958^ DM: diabetes melito; EAB: efeito do avental branco; HAR: hipertensão arterial resistente; HVE: hipertrofia ventricular esquerda; IRC: insuficiência renal crônica; MAPA: monitorização ambulatorial da pressão arterial; PAS: pressão arterial sistólica.

## 16.3. Fisiopatologia

Assim como a fisiopatologia da hipertensão arterial primária é multifatorial, na HAR e na HARf múltiplos fatores também estão envolvidos na sua gênese. Isso determina diferentes graus de refratariedade aos fármacos anti-hipertensivos ( Figura 16.1 ).

**Figura 16.3:**
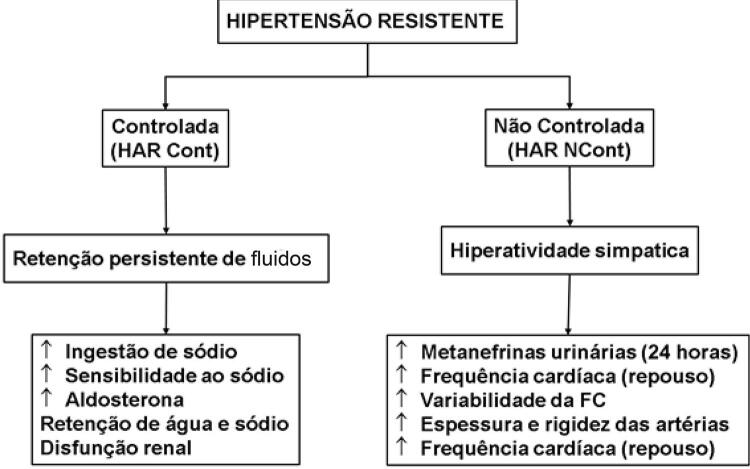
– Mecanismos fisiopatológicos predominantes na hipertensão resistente.

A HAR depende mais do aumento de volemia do que a HARf, devido à importante persistência de retenção de fluidos, sensibilidade aumentada ao sódio, hiperaldosteronismo e disfunção renal. Além disso, maior expansão de conteúdo plasmático torácico, concentração de aldosterona urinária, discreta supressão da atividade de renina ^957^ e elevada relação aldosterona/renina plasmática, assim como altos níveis de peptídeos natriuréticos atrial e cerebral (BNP) são observados nesses indivíduos. ^958^ ^-^ ^960^ Essa relação entre volume e pressão elevada é a principal base fisiopatológica demonstrada em vários estudos ^955^ ^,^ ^957^ ^-^ ^960^ ( Figura 16.3 ) e justifica o uso de diuréticos na terapêutica de pacientes com HAR. ^961^

FC: Frequência cardíaca. Indivíduos refratários (não controlados) com cinco classes de anti-hipertensivos estão incluídos no grupo refratário (3-5%).

**Figura 16.4:**
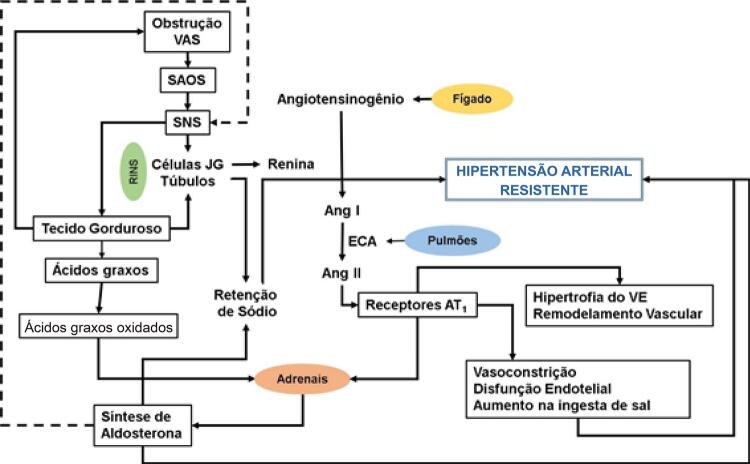
– Sistemas biomoleculares mediando o desequilíbrio entre a síntese aumentada de aldosterona, a retenção de sódio, a AOS, a atividade aumentada do SRAA (receptores AT1 e AT2) e a cardiopatia hipertensiva e resistência vascular total aumentada, induzidas principalmente pela expansão do volume plasmático (retenção de sal e excesso de aldosterona) e pela hiperatividade simpática.

SAOS: síndrome da apneia obstrutiva do sono; SNS: sistema nervoso simpático; Células JG: células juxta-glomerulares; FA oxidados: ácidos graxos oxidados; VE: ventrículo esquerdo; VAS: vias aéreas superiores; SRAA: sistema renina-angiotensina-aldosterona.

Em contraste, os portadores de HARf tem predominantemente hiperatividade do sistema nervoso simpático e maior rigidez vascular. ^962^ Valores mais elevados de velocidade de onda de pulso denotando rigidez vascular exacerbada e níveis elevados de citocinas, como o fator de necrose tumoral-α, ^963^ podem mediar o dano vascular em hipertensos refratários. ^964^ Outros fatores, como idade, obesidade, apneia obstrutiva do sono (AOS), descendência afro-americana, adipocinas alteradas, disfunção endotelial e maior atividade das metaloproteinases-2 e -9 e das moléculas de adesão, também estão envolvidos no processo. ^504^ ^,^ ^954^ ^,^ ^895^ Polimorfismos genéticos, especialmente do SRAA e da enzima óxido nítrico sintase endotelial, vêm sendo correlacionados com a HARf ^965^ (Figuras 16.4 e 16.5).

## 16.4. Investigação Diagnóstica

A rigor, os hipertensos resistentes devem ser acompanhados em serviços especializados em HA capazes de oferecer uma abordagem multidisciplinar. A investigação diagnóstica baseia-se em quatro pilares: ^341^ ^,^ ^504^ ^,^ ^954^ ^,^ ^966^

- **a) Pseudorresistência** : afastar aferição incorreta da PA (em especial, a largura do manguito em obesos), inércia clínica terapêutica, má adesão ao tratamento e uso de medicamentos que elevam a PA (ver Capítulo 15). ^504^ ^,^ ^954^ ^,^ ^967^
- **b) Avaliação de fatores de risco cardiovasculares (CV), lesões de órgãos-alvo (LOA) e doença CV estabelecida** : uma vez confirmada a HAR, deve ser iniciada a investigação diagnóstica com exames específicos, conforme a orientação desta diretriz de hipertensão com relação ao comprometimento de LOA e à hipertensão secundária. A ocorrência de comorbidades associadas deve ser detectada com exames especializados de acordo com a suspeita clínica.
- **c) Monitorização ambulatorial da pressão arterial (MAPA) e monitorização residencial da pressão arterial (MRPA):** o diagnóstico da HAR baseia-se na PA de consultório, ^504^ ^,^ ^954^ porém a avaliação da pressão fora do consultório (MAPA ou MRPA) é fundamental para a exclusão do efeito do avental branco e da hipertensão mascarada. ^504^ ^,^ ^954^ ^,^ ^968^ A conduta diagnóstica e terapêutica deve basear-se nas pressões da MAPA ou MRPA. ^504^ ^,^ ^954^ ^,^ ^968^ ^,^ ^969^ Pacientes com níveis pressóricos elevados na vigília e/ou no sono (HAR verdadeira ou HA mascarada) deverão ter sua medicação ajustada e a MAPA repetida após o ajuste terapêutico. ^504^ ^,^ ^954^ ^,^ ^968^ ^,^ ^969^ Os pacientes com a MAPA controlada devem ter seu esquema terapêutico mantido, independentemente dos valores da PA de consultório. Nesses indivíduos, a MAPA deve ser repetida semestral ou anualmente. ^968^ ^,^ ^969^ A MRPA também pode ser utilizada quando disponível. Apesar de não avaliar o período noturno e superestimar os níveis pressóricos, apresenta uma concordância moderada no diagnóstico, com alta especificidade e baixa sensibilidade. ^970^
- **d) Investigação de causas secundárias:** as causas secundárias são mais comuns nos hipertensos resistentes do que nos não resistentes, sendo a mais prevalente a AOS (80%), seguida do hiperaldosteronismo (6-23%), renovascular (estenose de artéria renal) (2,5-20%) e doença do parênquima renal (2 a 10%). ^504^ ^,^ ^954^ ^,^ ^895^ A investigação de alterações da função tireoidiana (1-3%) também se justifica. ^504^ ^,^ ^954^

## 16.5. Tratamento ( Quadro 16.2 )

### 16.5.1. Tratamento Não Farmacológico

Todos os pacientes com HAR devem ser orientados e incentivados a adotar modificações de estilo de vida ^971^ (ver Capítulo 8).

**Quadro 16.2:** – Tratamento da hipertensão arterial resistente

Intervenção	GR	NE
Institua e incentive MEV	I	B
Otimize tratamento com três medicações: hidroclorotiazida, clortalidona ou indapamida,* IECA ou BRA, e BCC†	I	B
Adicione espironolactona como 4a medicação	I	A
Adicione BB e/ou clonidina como 5 ^a^ /6 ^a^ medicação†	IIA	B
Adicione sequencialmente vasodilatadores de ação direta	IIB	C
Prescreva uma ou mais das medicações à noite ao deitar-se	IIB	B
Verifique e melhore a adesão ao tratamento	I	C
Não utilize tratamentos invasivos, exceto em protocolos de pesquisa	III	B

*Com taxa de filtração glomerular ≤ 30 mL/min ou ICC, usar diurético de alça. †Com doença arterial coronariana, insuficiência cardíaca e taquiarritmias, os BBs substituem os BCCs no tratamento triplo inicial. BB: betabloqueador; BCC: bloqueadores de canais de cálcio; BRA: bloqueadores dos receptores AT1 da angiotensina II; IECA: inibidores da enzima conversora de angiotensina; MEV: mudanças de estilo de vida.

### 16.5.2. Tratamento Farmacológico

O princípio básico do tratamento farmacológico é a associação de medicamentos anti-hipertensivos que tenham ação sobre a maioria dos mecanismos fisiopatológicos de elevação da PA: a expansão do volume intravascular, a ativação simpática e do SRAA e a resistência vascular periférica aumentada. ^504^ ^,^ ^954^ ^,^ ^972^ O melhor tratamento triplo deve incluir um diurético (DIU) tiazídico, um bloqueador do SRAA (inibidor da ECA ou bloqueador dos receptores AT1 da angiotensina (BRA) II) e um bloqueador dos canais de cálcio (BCC) di-hidropiridínico de longa duração, em doses plenas e toleradas a intervalos adequados. Na presença de doença arterial coronariana, insuficiência cardíaca ou taquiarritmias, um betabloqueador (BB) deve substituir o BCC no esquema terapêutico inicial com três medicações.

O uso correto de DIU é essencial no tratamento da HAR: a clortalidona (25 mg/dia) ou a indapamida (1,5mg/dia) são os diuréticos de escolha desde que haja um ritmo de filtração glomerular estimado (RFG-e) superior a 30 mL/min. Entretanto, no momento da elaboração deste texto, somente a hidroclorotiazida está disponível na rede pública de saúde no Brazil. Em pacientes com doença renal crônica estágios 4 ou 5 e insuficiência cardíaca com retenção de volume, o DIU de alça (furosemida) deve ser utilizado em lugar dos tiazídicos e administrados de acordo com a necessidade com relação ao controle volêmico e pressórico. A espironolactona (antagonista da aldosterona, 25 a 50 mg/dia) é a medicação de escolha a ser acrescentada como 4 ^o^ fármaco nos pacientes aderentes ao tratamento e com HAR verdadeira. ^564^ ^,^ ^567^ Nos intolerantes à espironolactona, a amilorida (5 a 10 mg/dia) pode ser utilizada. ^973^

Nos pacientes sem controle pressórico após a adição de espironolactona, os BB (principalmente aqueles com efeito vasodilatador) ou os alfa-agonistas de ação central (clonidina) ^564^ são as medicações de 5 ^a^ /6 ^a^ linhas. Caso o controle pressórico ainda não seja alcançado, os vasodilatadores diretos (hidralazina e minoxidila) podem ser utilizados como medicações de 7 ^a^ opção ^974^ ^,^ ^975^

A cronoterapia dirigida pela MAPA, com pelo menos uma das medicações anti-hipertensivas administrada à noite (sobretudo os bloqueadores do SRAA e os BB), melhorou o controle pressórico e reverteu o padrão não *dipper* em pacientes com HAR, além de reduzir a morbimortalidade cardiovascular. ^976^

A adesão ao tratamento é fundamental para o controle pressórico. Entretanto, até 50% dos pacientes com HAR são parcial ou completamente não aderentes ao tratamento farmacológico. ^977^

### 16.5.3. Novos Tratamentos

Diversos tratamentos invasivos, como denervação simpática renal endovascular, terapia de ativação e de modulação do barorreflexo carotídeo, ablação do corpo carotídeo e anastomose arteriovenosa ilíaca central, não estão aprovados e não devem ser utilizados no tratamento de hipertensos resistentes, exceto em protocolos de pesquisa. ^5^ ^,^ ^978^

**Table:** 

Mensagens principais
São considerados hipertensos resistentes os indivíduos aderentes ao tratamento em uso de três ou mais classes de fármacos anti-hipertensivos em doses otimizadas que não apresentam pressão arterial controlada.
Os hipertensos refratários são os aderentes não controlados com 5 ou mais classes de fármacos anti-hipertensivos em doses otimizadas.
No Brazil, a prevalência de hipertensão arterial resistente verdadeira é de 11,7% (Estudo ReHot).
A hipertensão resistente depende mais de volume, enquanto na hipertensão refratária predomina a hiperatividade simpática.
Fundamental nas três classes de fármacos iniciais no tratamento da hipertensão resistente usar diurético, bloquear o sistema renina-angiotensina-aldosterona e utilizar vasodilatadores de ação direta.

## 17. Adesão ao Tratamento Anti-Hipertensivo

## 17.1. Introdução

A implementação do tratamento anti-hipertensivo com medidas farmacológicas e não farmacológicas visa, principalmente, a reduzir a morbidade e a mortalidade decorrentes dos valores elevados da pressão arterial (PA). Apesar da efetividade e da eficácia comprovadas do tratamento, os índices de controle da hipertensão arterial (HA) ainda são insatisfatórios na maioria dos países, inclusive o Brazil. ^979^ Um estudo nacional de revisão sistemática e metanálise realizado na atenção primária à saúde mostrou que a taxa de controle da HA variou de 43,7% a 67,5%. ^980^ Os motivos para a falta de controle dos hipertensos são diversos, mas um dos fatores de maior peso nesse cenário é certamente a falta de adesão ao tratamento por diferentes motivos.

## 17.2. Conceito de Adesão

Um documento da Organização Mundial da Saúde (OMS) publicado em 2003 ^981^ definiu a adesão como o “grau em que o comportamento de uma pessoa – tomar o medicamento,seguir uma dieta e/ou executar mudanças no estilo de vida (MEV) – corresponde às recomendações acordadas com um prestador de assistência à saúde”. O principal motivo do controle inadequado da HA parece ser o não cumprimento do tratamento a longo prazo, tanto em MEV quanto no que se refere à observação da prescrição médica medicamentosa. Em 2012, em um novo documento da OMS, os autores diferenciam os processos, como a adesão aos medicamentos e o manejo da adesão. ^982^ De acordo com tal diretriz, a adesão ao medicamento é um processo caracterizado por três grandes componentes: o início, a implementação e a descontinuação. O início é o tempo desde a prescrição até a tomada da primeira dose do medicamento; a implementação corresponde à coincidência entre a dose que o paciente toma e a dosagem prescrita; e a descontinuação marca a interrupção, quando se omite a próxima dose a ser tomada e se interrompe o tratamento posteriormente. ^982^

Apesar de várias terminologias utilizadas como sinônimos de adesão, como aderência, observância, complacência, fidelidade, *compliance* , a palavra mais apropriada para o tratamento proposto da HA é adesão. Os problemas de adesão nem sempre são fáceis de se detectar e quantificá-los é ainda mais difícil. Para melhorar o controle da HA, é importante reunir esforços no sentido de identificar os pacientes não aderentes ao tratamento proposto. O abandono mostra-se frequente nos primeiros meses do tratamento, além de os pacientes poderem tomar os medicamentos em desacordo com a prescrição médica. Tal situação é descrita na literatura desde a década de 1970 ^983^ e mantém-se em publicações mais recentes. ^984^ ^,^ ^985^ A falta de adesão ao tratamento é frequentemente definida quando os hipertensos fazem uso de menos de 80% dos medicamentos prescritos. No entanto, pode variar ao longo de um contínuo de zero e até ultrapassar 100% naqueles que usam mais do que foi prescrito, o que também se considera não adesão ao tratamento.

## 17.3. Métodos de Avaliação da Adesão ao Tratamento

Existem várias formas para medir a adesão ao tratamento medicamentoso anti-hipertensivo na prática clínica e em pesquisa, sendo classificados como métodos diretos os que há comprovação objetiva da tomada do medicamento pelo paciente; e métodos indiretos aqueles em que estratégias diversas estimam ou não a tomada do medicamento prescrito. A escolha do melhor método dependerá da finalidade do uso da informação obtida, dos recursos disponíveis para a avaliação, da aceitação, da conveniência para o paciente do método a ser utilizado e dos custos envolvidos. ^986^ Medir a adesão ao tratamento é uma tarefa complexa. Não há um método considerado padrão-ouro que represente as várias dimensões que envolvem o processo. ^344^

A OMS sugere o uso de um método indireto associado a um método direto para a medida da adesão ^984^ para doenças crônicas. No contexto da HA, os métodos indiretos acabam sendo os mais utilizados, pois os métodos diretos ainda carecem de validação, são caros e estão somente disponíveis em ambientes de pesquisa.

As escalas estruturadas de autorrelato são muito empregadas em pesquisa clínica, como a escala de adesão terapêutica de Morisky-Green. A Escala de Adesão Terapêutica de Oito Itens de Morisky ^987^ foi criada a partir da escala anterior de quatro itens, ^988^ apresentando maior confiabilidade (α= 0,83 *versus* α= 0,61) e possui validação para o português Brazileiro. ^989^ O escore total é classificado de acordo com a seguinte pontuação: 8 pontos indicam alta adesão; entre 6 e 7, moderada adesão; e inferior a 6, baixa adesão. Outro instrumento é o Questionário de Adesão a Medicamentos – Qualiaids (QAM-Q), criado em nosso meio, com três perguntas. As medidas de precisão para detectar a não adesão mostraram sensibilidade de 62,5% e especificidade de 85,7%, área sob a curva ROC de 74,1% e valor preditivo positivo de 90,9%. ^990^

Um artigo de revisão sobre adesão e HA salienta a importância de aumentar a disponibilidade e a acessibilidade de medidas mais precisas de avaliação da adesão. Destaca, ainda, que essa é a razão pela qual diretrizes recentes enfatizam a necessidade de abordar a adesão aos medicamentos como uma questão importante no tratamento da HA. ^991^ O Quadro 17.1 esclarece as vantagens e desvantagens dos vários métodos de avaliação da adesão à terapia medicamentosa. ^992^

**Quadro 17.1:** – Vantagens e desvantagens dos vários métodos de avaliação da adesão à terapia medicamentosa

MÉTODOS	VANTAGENS	DESVANTAGENS
**MÉTODOS DIRETOS**		
**Análise biológica em sangue ou urina**	Objetivo e permite a determinação da concentração do medicamento.	Custo elevado. Pode ser afetado por fatores biológicos e pela “adesão do avental branco”*
**Adição de marcador**	Objetivo e pode ser usado no placebo em pesquisa clínica.	Requer ensaios quantitativos de alto custo e coleta de amostragem de fluidos corporais.
**Tomada supervisionada**	Preciso.	Pacientes podem esconder os comprimidos embaixo da língua e depois descartá-los. Difícil aplicação na rotina ambulatorial de pacientes com hipertensão, podendo ser reservado para os casos de hipertensão resistente e refratária.
**MÉTODOS INDIRETOS**		
**Questionários estruturados de adesão (escalas de autorrelato)**	Simples, fácil, barato e muito utilizado.	Suscetível a erros com aumento do tempo de intervalo entre as consultas. Os resultados podem ser distorcidos pelos pacientes.
**Impressão do médico**	Fácil e barato.	Baixa sensibilidade.
**Contagem manual de comprimidos**	Objetivo, quantificável e de fácil execução.	Requer a colaboração do paciente em retornar os medicamentos. Dados podem ser alterados pelos indivíduos.
**Reabastecimento de receitas**	Objetivo e de fácil obtenção de dados.	Requer programa de computação e centralização dos registros e das farmácias.
**Resposta clínica**	Simples e de fácil execução.	Outros fatores, além da adesão, podem afetar a resposta clínica.
**Dispositivos eletrônicos**	Preciso e identifica padrões nas tomadas. Os resultados são facilmente quantificáveis.	Método de alto custo, o qual requer visitas de retorno e processamento dos dados gerados.

* “Adesão do avental branco”: situação em que o paciente se torna mais aderente ao tratamento recomendado antes da coleta de exames laboratoriais ou da consulta médica.

## 17.4 Fatores que Inteferem na Adesão ao Tratamento

A adesão ao tratamento é um processo complexo e multidimensional no qual se identificam barreiras reunidas em cinco dimensões ( Quadro 17.2 ) ^985^ ^,^ ^993^ ^-^ ^996^ que podem fornecer uma visão mais abrangente para os profissionais de saúde, visando a intervenções eficazes para o melhor controle da PA. Fatores como idade, renda, escolaridade e etnia/raça destacam-se principalmente em locais com predomínio de baixo nível socioeconômico. O sistema de saúde ofertado e as características da equipe de saúde também podem influenciar a adesão dos hipertensos. Quanto à doença e ao tratamento, ressaltam-se a cronicidade e a ausência de sintomatologia da HA, o tratamento para toda a vida e o esquema medicamentoso complexo em alguns casos, além de efeitos indesejáveis e interações dos fármacos. Nos aspectos relacionados com o paciente, acrescentam-se a falta de envolvimento com sua problemática de saúde e o esquecimento do uso de medicamentos.

**Quadro 17.2:** – Fatores que interferem na adesão ao tratamento anti-hipertensivo


**FATORES SOCIODEMOGRÁFICOS** • Sexo; • Idade; • Baixa escolaridade; • Baixa renda; • Minorias raciais/etnicidade; • Acesso a transporte, distância e moradia em zona rural; • Situações de desastres e pandemia.
**FATORES RELACIONADOS COM O TRATAMENTO MEDICAMENTOSO** • Falta de medicamentos nos serviços de saúde; • Custo de aquisição dos medicamentos; • Efeitos adversos; • Esquemas posológicos complexos; • Esquema terapêutico inadequado; • Tratamento contínuo e prolongado.
**FATORES RELACIONADOS COM AS EQUIPES E O SISTEMA DE SAÚDE** • Relacionamento médico/paciente inadequado; • Ausência de atendimento em equipe multiprofissional; • Tratamento não individualizado; • Falha de identificar a não adesão; • Comunicação ineficaz; • Sobrecarga de trabalho da equipe de saúde; • Falta de atualização.
**FATORES RELACIONADOS COM O PACIENTE** • Negação do diagnóstico; • Falta de percepção do benefício do tratamento; • Conhecimento inadequado da doença e de seu tratamento; • Esquecimento de tomar a medicação; • Baixa motivação e autoestima; • Medo de dependência e dos efeitos adversos dos medicamentos.
**FATORES RELACIONADOS COM A DOENÇA** • Ausência de sintomas; • Complicações a longo prazo; • Presença de outras comorbidades associadas; • Abuso de álcool e drogas ilícitas; • Interferência na qualidade de vida.

## 17.5. Estratégias para promover a adesão ao tratamento anti-hipertensivo

A falta de adesão ao tratamento tem como principal consequência a falta de controle da HA e, portanto, o aumento de lesões em órgãos-alvo (LOA) e da morbimortalidade cardiovascular (CV). Essas consequências, por sua vez, têm grande impacto econômico, devido a maiores gastos de atendimentos de saúde e aposentadorias precoces. Por isso, a adoção de estratégias com o objetivo de promover uma melhor adesão ao tratamento anti-hipertensivo, seja de forma isolada ou em conjunto, sintetizadas no Quadro 17.3 , tem como finalidade a mudança desse cenário. ^197^ ^,^ ^997^ ^-^ ^1006^

**Quadro 17.3:** – Estratégias para promover a adesão ao tratamento anti-hipertensivo


**INTERVENÇÕES NO PACIENTE** • Estratégias motivacionais; • Monitorização da pressão arterial domiciliar (medir a PA em casa); • Serviços de telemonitoramento a distância; • Educação em saúde para promover autocuidado; • Usos de lembretes e caixas organizadoras de medicamentos; • Incentivar apoio familiar e social; • Sessões de educação em grupo; • Envio de mensagem por telefonia móvel.
**INTERVENÇÕES NO TRATAMENTO MEDICAMENTOSO** • Evitar doses elevadas em monoterapia; • Escolha de medicamentos com menor perfil de eventos adversos; • Esquemas com melhor comodidade posológica: •Dose única diária; •Dois ou três anti-hipertensivos combinados no mesmo comprimido; • Receituário de fácil entendimento (escrito ou impresso); • Tratamento diferenciado de acordo com características clínicas e demográficas (negros, idosos, mulheres, obesos, diabéticos).
**INTERVENCÕES NAS EQUIPES E SISTEMAS DE SAÚDE** • Estabelecer vínculo com o paciente (fixar equipe de atendimento); • Comunicação clara; • Convocação de faltosos às consultas; • Visitas domiciliares; • Atuação de equipe multidisciplinar (médico, enfermeiro, farmacêutico, educador físico, nutricionista, psicólogo, assistente social, agentes comunitários de saúde); • Facilitar acesso aos medicamentos.

Entre todas as estratégias disponíveis, as mais factíveis de serem implementadas no Brazil e com maiores evidências, destacam-se:

• Automedida da PA (Grau de Recomendação I/Nível de Evidência B);
• Esquemas posológicos com maior comodidade: menores doses possíveis, tomada em dose única diária, associação de anti-hipertensivos em um mesmo comprimido (Grau de Recomendação I/Nível de Evidência A);
• Implementação de equipes multiprofissionais no cuidado de pacientes hipertensos com médico, enfermeiro, farmacêutico, educador físico, nutricionista, psicólogo, assistente social e agentes comunitários de saúde (Grau de Recomendação I/Nível de Evidência B).

## 17.6. Conclusão

Otimizar os índices de adesão ao tratamento anti-hipertensivo contribui para a diminuição dos custos de morbidade, mortalidade e com a assistência à saúde. Atualmente, dispõe-se de arsenal terapêutico por meio de tratamento medicamentoso e não medicamentoso com eficácia comprovada. A adesão às propostas de tratamento e consequente controle dos hipertensos ainda é um grande desafio para todos profissionais de saúde. Dessa maneira, reunir esforços para atender às reais necessidades da população hipertensa mostra-se tarefa primordial para a mudança do atual panorama no contexto da HA.

**Table:** 

Mensagens principais
Os índices de controle da HA ainda são insatisfatórios no Brazil. Os motivos para a falta de controle dos hipertensos são diversos, mas um dos fatores de maior peso neste cenário é a falta de adesão ao tratamento.
A adesão ao tratamento é um processo complexo e multidimensional no qual se identificam barreiras relacionadas com as condições sociodemográficas, o tratamento medicamentoso, os sistemas de saúde, o paciente e a doença propriamente dita.
Os problemas de adesão nem sempre são fáceis de se detectar, e quantificá-los é ainda mais difícil.
Medir a adesão ao tratamento é uma tarefa complexa. Não há um método considerado padrão-ouro que represente as várias dimensões que envolvem o processo.
Entre todas as estratégias disponíveis para a melhora da adesão, as mais factíveis de serem implementadas no Brazil e com maiores evidências são: Automedida da pressão arterial (GR: I, NE: B); Esquemas posológicos com maior comodidade: menores doses possíveis, tomada em dose única diária, associação de anti-hipertensivos em um mesmo comprimido (GR: I, NE: A); Implementação de equipes multiprofissionais no cuidado de pacientes hipertensos com médico, enfermeiro, farmacêutico, educador físico, fisioterapeuta, nutricionista, psicólogo, musicoterapeuta, assistente social e agentes comunitários de saúde (GR: I / NE: B).

## 18. Perspectivas

## 18.1. Introdução

O objetivo deste capítulo é discutir, com base na evolução do conhecimento científico relativo à doença hipertensiva nas últimas décadas e nas evidências mais atuais, os possíveis avanços e ajustes que permearão nosso dia a dia no enfrentamento dos desafios do diagnóstico, do tratamento e do acompanhamento do paciente hipertenso. Ressalta-se que, diferentemente dos capítulos anteriores desta diretriz, que foram rigorosamente fundamentados em níveis de evidências cientificas e graus de recomendação, este foi idealizado visando a apresentar possíveis horizontes racionais, com base no conhecimento que temos até esse momento, dessa doença multifatorial e complexa, cujas consequências cardiovasculares (CV), cerebrais e renais determinam morbidade e mortalidade impactantes, a ponto de ser a principal causadora de morte em todo o mundo.

## 18.2. Definição, Epidemiologia e Prevenção Primária

**Figura 18.1:**
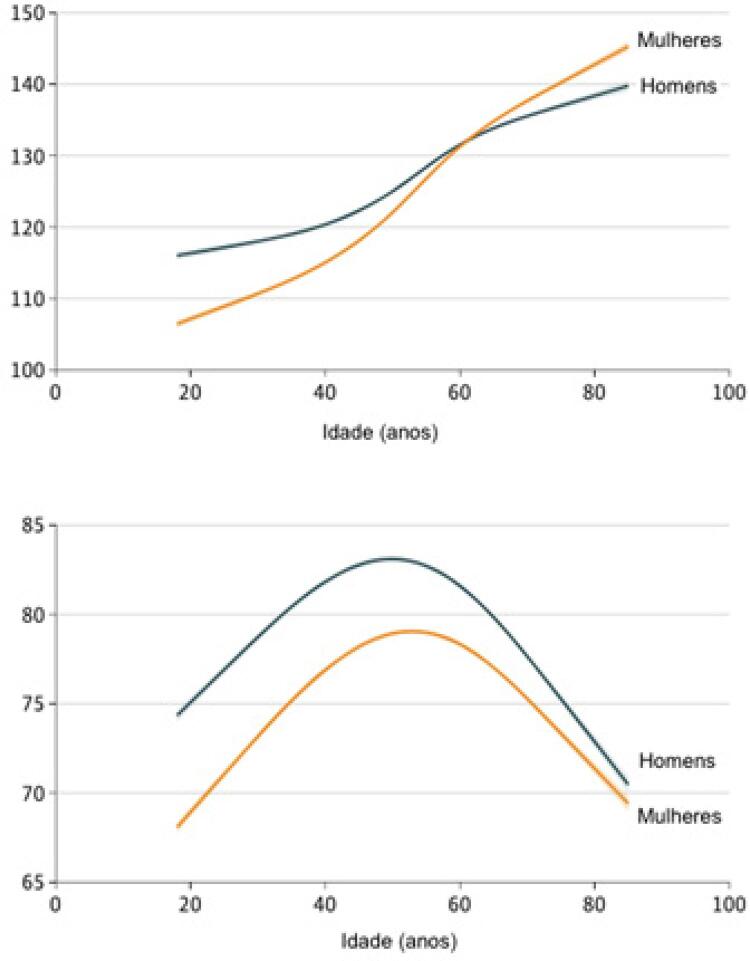
– Comportamento da pressão arterial sistólica e diastólica ao longo da vida e por sexo.

Com o aumento gradativo na expectativa de vida nos países desenvolvidos e em desenvolvimento, a prevalência da hipertensão arterial (HA) tende a aumentar ainda mais. Em média, observou-se o ganho de 1,4 ano por década de vida após os 60 anos em países desenvolvidos e 1,2 ano nos países latino-americanos no período de 1980 até 2011. ^1007^ Sabidamente, à medida que envelhecemos, observa-se um aumento nas cifras tensionais e, a partir dos 60 anos, prevalece o incremento da pressão arterial sistólica (PAS) com descenso da pressão diastólica (PAD). Isso proporciona maior pressão de pulso (PP). Todos esses aspectos são importantes a serem considerados na avaliação do risco relacionado e para as estratégias de tratamento a serem adotadas ( Figura 18.1 ). ^180^ ^,^ ^1008^

Fonte: Ji H et al., 2020. ^1008^

Esses aspectos epidemiológicos reforçam a importância do diagnóstico precoce e da identificação de lesões subclínicas associadas à HA, assim como o controle adequado da pressão arterial (PA) já nas fases iniciais da doença hipertensiva com o objetivo de diminuir ainda mais o risco CV. ^1009^ No futuro, conforme novas e robustas evidências epidemiológicas, os valores de referência para o diagnóstico, assim como para a estratégia de início de tratamento e metas de controle da PA, poderão ser revisados.

## 18.3. Pressão Arterial e Dano Vascular

O aumento do risco CV a partir de valores de 115 mmHg para a PAS e 75 mmHg para a PAD é algo bem conhecido, ^78^ e as medidas de PA continuam a ser o marcador diagnóstico da doença hipertensiva. No entanto, a ciência tenta avançar com o melhor entendimento e a aplicabilidade clínica de biomarcadores capazes de identificar precocemente o dano vascular no contexto da doença hipertensiva, antes mesmo da maior elevação dos valores de PA. O objetivo é aumentar a precisão na estratificação de risco CV em indivíduos considerados de risco baixo ou intermediário. ^82^ ^,^ ^139^ ^,^ ^1010^

As avaliações da pressão sistólica central e da rigidez arterial pela medida da velocidade de onda de pulso (VOP) ancoram-se em evidências robustas a fim de identificar precocemente dano vascular e ser capazes de identificar e reestratificar aqueles indivíduos que, inicialmente considerados como de risco baixo e intermediário, na verdade podem apresentar risco mais elevado. Além disso, os valores de VOP superiores a 10 m/s podem caracterizar a presença de lesão subclínica em órgão-alvo, ^156^ ^,^ ^298^ ^,^ ^1011^ e o aumento na pressão sistólica central é preditor do desenvolvimento de HA. ^1012^ Outra forma de avaliar o dano vascular é a capacidade de identificar a perda ou a diminuição da funcionalidade do endotélio, bem como entender a fisiopatogenia relacionada, que perpassa pela predisposição genética e pelo envelhecimento cronológico, além de alterações nas atividades inflamatória e imunológica, na sensibilidade à insulina e nas lipoproteínas ricas em colesterol. ^112^ ^,^ ^114^

Atualmente, o método mais empregado para analisar a função endotelial *in vivo* é a dilatação fluxo mediada (DFM), mas permanece ainda restrito no campo da pesquisa. ^118^ É possível que, à medida que novas evidências sejam produzidas no contexto da doença hipertensiva e das doenças CV, esse método venha a ser mais fidedigno e seguro para ser incorporado à prática clínica, objetivando identificar ainda mais precocemente o dano vascular. ^1013^ ^,^ ^1014^

## 18.4. Biomarcadores Cardíacos

Embora muito se tenha evoluído na busca por marcadores que estimem o dano arterial, não podemos subestimar exames identificadores de risco CV, como a demonstração de hipertrofia ventricular esquerda (HVE) por eletrocardiograma (ECG), ecocardiograma, ressonância magnética ou, mais modernamente, escore de cálcio coronariano, entre outros. ^1015^ ^,^ ^1016^ Há robustas evidências que incentivam a utilização dos peptídeos natriuréticos do tipo B, N-terminal pro-peptídio natriurético do tipo B (NT-proBNP) e do peptídeo natriurético do tipo B (BNP), assim como a troponina T de alta sensibilidade (hs-TnT) na estratificação de risco de eventos CV fatais ou não fatais, ou morte por todas as causas. Os peptídeos natriuréticos do tipo B são secretados pelos miócitos em resposta contrarregulatória às sobrecargas de volume ou de pressão na parede miocárdica, ao aumento do tônus simpático e à vasoconstrição, mas também integram o estresse CV e hemodinâmico de várias fontes. ^1017^ As troponinas cardíacas são proteínas estruturais do aparelho contrátil dos miócitos cardíacos que são liberadas na circulação após o dano celular. ^1018^

Um estudo recente revelou que a simples elevação de NT-proBNP e/ou hs-TnT, em pacientes com pré-hipertensão, foi capaz de discriminar cerca de 1/3 daqueles que apresentariam desfechos CV e internações por insuficiência cardíaca (IC) em 10 anos e que, possivelmente, poderiam se beneficiar de tratamento medicamentoso. ^1019^ Também se demonstrou que o NT-proBNP conseguiu estimar o risco de morte por todas as causas e desfechos CV não fatais em pacientes diabéticos hipertensos de alto risco, em 2,6 anos, com a mesma habilidade preditiva que todo o conjunto das 20 mais significativas variáveis clínicas e laboratoriais frequentemente utilizadas, como hs-TnT, idade, albumina, história de IC, frequência cardíaca, história de acidente vascular encefálico (AVE), HbA1c, tabagismo, HVE no ECG, onda Q no ECG, história de fibrilação atrial, qualquer bloqueio de ramo no ECG, relação albumina/creatinina na urina, PAS, sexo, história de doença arterial coronária, colesterol de lipoproteína de baixa densidade, ritmo de filtração glomerular estimado, uso de insulina e PAD. ^1020^

## 18.5. Diagnóstico e Classificação

O diagnóstico da HA, baseado nos resultados da medida de consultório e atendendo aos preceitos básicos de técnica e aparelhos adequadamente utilizados, é definido como ≥ 140 mmHg para a PAS e ≥ 90 mmHg para a PAD. ^164^ Nas últimas diretrizes internacionais de HA, tem sido recomendado que o diagnóstico da HA, sempre que possível, seja baseado na medida do consultório, preferencialmente realizada de forma desacompanhada, ou então por meio de medidas fora do consultório (MAPA e MRPA). Além disso, discute-se se os valores de referência para definir a presença de HA deveriam ser ainda mais baixos. ^37^ ^,^ ^164^ ^,^ ^186^ ^,^ ^1021^

Parece claro que identificar a HA, com ou sem tratamento, de acordo com seus fenótipos, permite uma estratificação de risco e uma definição de estratégia de tratamento mais individualizadas. ^180^ ^,^ ^212^ Outro aspecto interessante a ser abordado é o uso da automedida da pressão arterial (AMPA) como método capaz de aumentar a atenção do paciente para com sua doença e propiciar maior adesão ao tratamento, além de oferecer ao profissional de saúde maiores informações sobre o comportamento da PA no dia a dia do indivíduo. ^1022^

Com a pandemia da covid-19, é fundamental reconhecer o desenvolvimento mundial de técnicas de telemedicina. Aparentemente, o monitoramento a distância por meio de plataformas digitais e aplicativos para hipertensos surgiu para ficar, facilitando o diálogo entre a equipe de saúde e o paciente e a consequente troca de informações e ajustes benéficos nas mudanças no estilo de vida e mesmo no tratamento, com foco na prevenção e no melhor controle da doença. No entanto, a tecnologia digital pode expandir ainda mais no âmbito da hipertensão, assim como ocorreu no cenário do diabetes melito, possibilitando o desenvolvimento cada vez mais preciso de ferramentas de monitorização contínua da PA, sem *cuffs* , e em sincronia com *smartphones* , hoje disponíveis para a maioria da população mundial. ^388^ ^,^ ^1023^ ^,^ ^1024^

Por fim, é possível que nos anos vindouros o olhar mais atento ao paciente com níveis pressóricos ≥ 130 mmHg para a PAS e ≥ 85 mmHg para a PAD (hoje considerado como pré-hipertenso nesta e em outras diretrizes) mude o entendimento sobre o conceito do diagnóstico da HA. ^1025^

## 18.6. Avaliação Complementar e Estratificação do Risco Cardiovascular

A utilização de biomarcadores para a identificação precoce de lesões subclínicas, bem como do maior risco CV, mesmo em fases iniciais de elevação da PA, traz a expectativa de que, em indicações específicas, convém iniciar precocemente os cuidados para com esse indivíduo. ^139^ Entre os biomarcadores, existem aqueles que avaliam o dano vascular. Alguns deles, como a medida do índice tornozelo-braquial (ITB), o escore de cálcio ou a medida da VOP, ^298^ ^,^ ^1011^ apresentam evidências para as proposições anteriormente descritas, embora não largamente disponíveis na prática clínica. Enquanto isso, outros, como a DFM, ainda estão aplicados apenas no campo da pesquisa. ^118^ Ademais, estuda-se atualmente uma série de substâncias relacionadas com a inflamação e que, em última análise, estão intimamente ligadas ao processo de disfunção do endotélio e à aterosclerose, mas ainda necessitam de evidências mais robustas para uma eventual aplicação na prática clínica. ^180^ ^,^ ^271^ ^,^ ^1014^ ^,^ ^1022^ ^,^ ^1026^

No contexto da estratificação de risco CV, a incorporação progressiva de biomarcadores permitirá, especialmente nos hipertensos de risco intermediário, separar com maior precisão o verdadeiro nível de risco desse indivíduo. Esse tipo de abordagem traz a possibilidade de uma prática médica mais personalizada, com maior assertividade nas decisões relacionadas com a classificação e o tratamento. ^156^ ^,^ ^298^ ^,^ ^1011^

Ainda, pensando na medicina de precisão, geralmente baseada em genômica e também metabolômica, ressalta-se que já existem escores clínicos validados capazes de identificar pacientes com maior chance da doença hipertensiva precoce, bem como valores de referência para a população Brazileira de alguns desses biomarcadores, corrigidos para sexo e idade. Certamente, a possibilidade de marcadores menos sofisticados vai ao encontro da melhora da precisão desejada para indicar avaliações específicas em pacientes clinicamente pré-selecionados, como habitualmente já é realizado na investigação de HA secundária, quando existem indícios clínicos e exame de rastreamento positivo. ^158^ ^,^ ^159^

## 18.7. Metas e Tratamento

**Figura 18.2:**
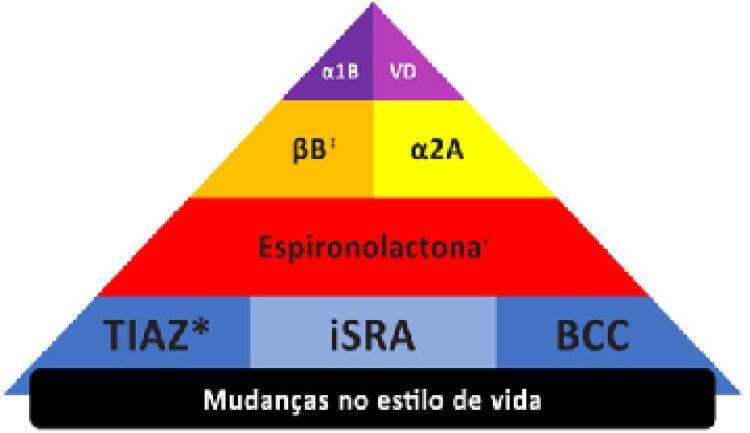
– O octeto medicamentoso para o tratamento da hipertensão arterial.

Conforme todos os avanços e comprovações científicas sobre o racional apontado nos tópicos anteriores, é plausível pensar que, para situações peculiares, o início precoce do tratamento e a busca por metas mais baixas de controle da PA possam ser indicados para prevenir os desfechos relacionados com o aumento da PA e minimizar o chamado risco residual. ^307^ ^,^ ^1027^ Além disso, a estratégia de tratamento medicamentoso baseado em combinações duplas ou mesmo triplas de anti-hipertensivos (em doses baixas), mesmo nas fases iniciais da doença, deve ganhar cada vez mais espaço nas recomendações de diretrizes, e a monoterapia talvez se mostre uma estratégia interessante como abordagem para os indivíduos hoje considerados como pré-hipertensos de risco alto ou naqueles com alterações em biomarcadores ( Figura 18.2 ). ^307^ ^,^ ^1023^ ^,^ ^1028^ ^,^ ^1029^

Fonte: Feitosa et al., 2020.1028 TIAZ*: diurético de longa duração tiazídico ou tiazídico-símile até 30 mL/minuto de ritmo de filtração glomerular estimado e na ausência de hipervolemia, senão trocar por diurético de alça; iSRA: inibidores do sistema renina-angiotensina-aldosterona; BCC: bloqueador do canal de cálcio di-hidropiridínico; BB: betabloqueador; α2A: agonista alfa-2 de ação central; α1B: bloqueador alfa-1-adrenérgico; VD: vasodilatador arterial direto; BB, que pode ser indicado antes em condições clínicas específicas.

Pode ser que, no horizonte da doença hipertensiva, conforme os estudos atualmente em curso, tenhamos como objetivo o controle dos parâmetros periféricos e centrais da PA, desde que se comprove nessa estratégia uma capacidade de maximizar a redução dos principais desfechos CV e renais. ^1030^ ^,^ ^1031^ Por fim, embora pareça longínquo, há a possibilidade real no tratamento da HA do uso cada vez maior de ferramentas moleculares altamente específicas, caso da interferência mediada por RNA. Nada mais é que do que o silenciamento gênico pós-transcricional ( *post transcription gene silencing* – PTGS) da expressão excessiva de determinada proteína de interesse. ^388^ ^,^ ^1032^ A terapia gênica, no caso da HA, aparece como promissora e já comprovada por estudos experimentais, em que o gene-alvo codifica o angiotensinogênio hepático. No entanto, há um caminho a ser perseguido para sua aplicação clínica, após se comprovar seletiva, eficaz e principalmente segura. Embora tantas perspectivas importantes estejam em foco, aparentemente o maior desafio de todos, no Brazil e no mundo, é bem mais simples de ser ultrapassado e inclui metas de melhorar o diagnóstico e tratar adequadamente e em equipe, com melhor controle da pressão arterial, visando a alcançar uma redução significativa na morbidade e na mortalidade cardiovascular e renal.

## References

[1] Forouzanfar MH, Liu P, Roth GA, Ng M, Biryukov S, Marczak L (2017). Global burden of hypertension and systolic blood pressure of at least 110 to 115 mm Hg, 1990–2015. JAMA.

[2] Anderson AH, Yang W, Townsend RR, Pan Q, Chertow GM, Kusek JW (2015). Time-updated systolic blood pressure and the progression of chronic kidney disease: a cohort study. Ann. Intern. Med..

[3] Précoma DB, Oliveira GMM, Simão AF, Dutra OP, Coelho OR, Izar MCO (2019). Atualização da Diretriz de Prevenção Cardiovascular da Sociedade Brazileira de Cardiologia – 2019. Arq Bras Cardiol.

[4] Arnett DK, Blumenthal RS, Albert MA, Buroker AB, Goldberger ZD, Hahn EJ (2019). 2019, ACC/AHA Guideline on the Primary Prevention of Cardiovascular Disease. JACC.

[5] Whelton PK, Carey RM, Aronow WS, Casey DE, Collins KJ, Himmelfarb CD (2017). Guideline for Prevention, Detection, Evaluation and Management of High Blood Pressure in Adults. J Am Coll Cardiol.

[6] Carey RM, Muntner P, Bosworth HB, Whelton PK (2018). Prevention and Control of Hypertension. JACC Health Promotion Series. J Am Coll Cardiol.

[7] Menni C, Mangino M, Zhang F, Clement G, Snieder H, Padmanabhan S (2013). Heritability analyses show visit-to-visit blood pressure variability reflects different pathological phenotypes in younger and older adults: evidence from UK twins. J Hypertens.

[8] Singh GM, Danaei G, Pelizzari PM, Lin JK, Cowan MJ, Stevens GA (2012). The age associations of blood pressure, cholesterol, and glucose: analysis of health examination surveys from international populations. Circulation.

[9] Brazil, Ministério da Saúde (2016). Vigitel Brazil, 2016: Vigilância de Fatores de Risco e Proteção para Doenças Crônicas por Inquérito Telefônico.

[10] Mente A, O’Donnell M, Rangarajan S, McQueen M, Dagenais G, Wielgosz A (2018). Urinary sodium excretion, blood pressure, cardiovascular disease, and mortality: a community-level prospective epidemiological cohort study. Lancet.

[11] Elliott P, Stamler J, Nichols R, Dyer AR, Stamler R, Kesteloot H (1996). Intersalt revisited: further analyses of 24 hours sodium excretion and blood pressure within and across populations. BMJ.

[12] Mill JG, Malta DC, Machado ÍE, Pate A, Pereira CA, Jaime PC (2019). Estimativa do consumo de sal pela população Brazileira: resultado da Pesquisa Nacional de Saúde 2013. Rev Bras Epidemiol.

[13] Araujo MC, Bezerra IN, Barbosa F dos S, Junger WL, Yokoo EM, Pereira RA (2013). Consumo de macronutrientes e ingestão inadequada de micronutrientes em adultos. Rev Saude Publica.

[14] Guthold R, Stevens GA, Riley LM, Bull FC (2018). Worldwide trends in insufficient physical activity from 2001 to 2016: a pooled analysis of 358 population-based surveys with 1.9 million participants. Lancet Glob. Health..

[15] Roerecke M, Kaczorowski J, Tobe SW, Gmel G, Hasan OSM, Rehm J (2017). The effect of a reduction in alcohol consumption on blood pressure: a systematic review and meta-analysis. Lancet Public Health.

[16] Fuchs FD, Chambless LE, Whelton PK, Nieto FJ, Heiss G (2001). Alcohol consumption and the incidence of hypertension: the Atherosclerosis Risk in Communities Study. Hypertension.

[17] Mills KT, Bundy JD, Kelly TN, Reed JE, Kearney PM, Reynolds K (2016). Global disparities of hypertension prevalence and control: a systematic analysis of population-based studies from 90 countries. Circulation.

[18] NCD Risk Factor Collaboration (NCD-RisC) (2017). Worldwide trends in blood pressure from 1975 to 2015: a pooled analysis of 1479 population-based measurement studies with 19·1 million participants. Lancet.

[19] Plavnik FL (2002). Hipertensão arterial induzida por drogas: como detectar e tratar. Rev Bras Hipertens.

[20] Ahmad M, Makati D, Akbar S (2017). Review of and Updates on Hypertension in Obstructive Sleep Apnea. Int J Hypertens.

[21] GBD 2016 Causes of Death Collaborators (2017). Global, regional, and national age-sex specific mortality for 264 causes of death, 1980- 2016: a systematic analysis for the Global Burden of Disease Study. Lancet.

[22] (2016). Causes of Death. Lancet.

[23] Brazil, Ministério da Saúde DATASUS/MS/SVS/CGIAE - Sistema de Informações sobre Mortalidade SIM.

[24] Khan KS, Wojdyla D, Say L, Gulmezoglu AM, Van Look PFA (2006). WHO analysis of causes of maternal death: a systematic review. Lancet.

[25] Udani S, Lazich I, Bakris GL (2011). Epidemiology of hypertensive kidney disease. Nat Rev Nephrol.

[26] Brazil, Ministério da Saúde, DATASUS Sistema de Informações Hospitalares do SUS(SIH/SUS).

[27] Malta DC, Gonçalves RPF, Machado IE, Freitas MIF, Azeredo C, Szwarcwald CL (2018). Prevalência da hipertensão arterial segundo diferentes critérios diagnósticos. Pesquisa Nacional de Saúde. Rev Bras Epidemiol.

[28] Nilson EAF, Andrade RCS, Brito DA, Oliveira ML (2020). Custos atribuíveis à obesidade, hipertensão e diabetes no Sistema Único de Saúde em 2018. Rev Panam Salud Publica.

[29] EAF, Silva EN, Jaime PC (2020). Developing and applying a costing tool for hypertension and related cardiovascular disease: attributable costs to salt/sodium consumption. J Clin Hypertens.

[30] Dickey RA, Janick JJ (2001). Lifestyle modifications in the prevention and treatment of hypertension. Endocr Pract.

[31] Perumareddi P (2019). Prevention of hypertension related to cardiovascular disease. Prim Care.

[32] Jiang SZ, Lu W, Zong XF, Ruan HY, Liu Y (2016). Obesity and hypertension (Review). Exp Ther Med.

[33] Jayedi A, Rashidy-Pour A, Khorshidi M, Shab-Bidar S (2018). Body mass index, abdominal adiposity, weight gain and risk of developing hypertension: a systematic review and dose-response meta-analysis of more than 2.3 million participants. Obes Rev.

[34] Zhao Y, Qin P, Sun H, Liu Y, Liu D, Zhou Q (2020). Metabolically healthy general and abdominal obesity are associated with increased risk of hypertension. Br J Nutr.

[35] Ross R, Neeland IJ, Yamashita S, Shai I, Seidell J, Magni P (2020). Waist circumference as a vital sign in clinical practice: a Consensus Statement from the IAS and ICCR Working Group on Visceral Obesity. Nat Rev Endocrinol.

[36] Semlitsch T, Jeitler K, Berghold A, Horvath K, Posch N, Poggenburg S (2016). Long-term effects of weight-reducing diets in people with hypertension. Cochrane Database Syst Rev.

[37] Williams B, Mancia G, Spiering W, Agabiti RE, Azizi M, Burnier M (2018). 2018 ESC/ESH Guidelines for the management of arterial hypertension: The Task Force for the management of arterial hypertension of the European Society of Cardiology and the European Society of Hypertension: The Task Force for the management of arterial hypertension of the European Society of Cardiology and the European Society of Hypertension. J Hypertens.

[38] Schwingshackl L, Chaimani A, Schwedhelm C, Toledo E, Pünsch M, Hoffmann G (2019). Comparative effects of different dietary approaches on blood pressure in hypertensive and pre-hypertensive patients: A systematic review and network meta-analysis. Crit Rev Food Sci Nutr.

[39] Pergola G, D’Alessandro A (2018). Influence of Mediterranean Diet on Blood Pressure. Nutrients.

[40] Ozemek C, Laddu DR, Arena R, Lavie CJ (2018). The role of diet for prevention and management of hypertension. Curr Opin Cardiol.

[41] Grillo A, Salvi L, Coruzzi P, Salvi P, Parati G (2019). Sodium Intake and Hypertension. Nutrients.

[42] Jafarnejad S, Mirzaei H, Clark CCT, Taghizadeh M, Ebrahimzadeh (2020). The hypotensive effect of salt substitutes in stage 2 hypertension: a systematic review and meta-analysis. BMC Cardiovasc Disord.

[43] Whelton PK, He J, Cutler JA, Brancati FL, Appel LJ, Follmann (1997). Effects of oral potassium on blood pressure. Meta-analysis of randomized controlled clinical trials. J Am Med Assoc.

[44] Stone MS, Martyn L, Weaver CM (2016). Potassium Intake, Bioavailability, Hypertension, and Glucose Control. Nutrients.

[45] Filippini T, Violi F, D’Amico R, Vinceti M (2017). The effect of potassium supplementation on blood pressure in hypertensive subjects: A systematic review and meta-analysis. Int J Cardiol.

[46] Poorolajal J, Zeraati F, Soltanian AR, Sheikh V, Hooshmand E, Maleki A (2017). Oral potassium supplementation for management of essencial hypertension: A meta–analysis of randomized controlled trials. Plos One.

[47] Caligiuri SPB, Pierce GN (2017). A review of the relative efficacy of dietary, nutritional supplements, lifestyle, and drug therapies in the management of hypertension. Crit Rev Food Sci Nutr.

[48] World Health Organization. (WHO) (2014). Global status report on noncommunicable diseases.

[49] Lim SS, Vos T, Flaxman AD, Danaei G, Shibuya K, Adair-Rohani H (2012). A comparative risk assessment of burden of disease and injury attributable to 67 risk factors and risk factor clusters in 21 regions, 1990– 2010: a systematic analysis for the Global Burden of Disease Study 2010. Lancet.

[50] Cornelissen VA, Smart NA (2013). Exercise training for blood pressure: a systematic review and meta-analysis. J Am Heart Assoc.

[51] Carlson DJ, Dieberg G, Hess NC, Millar PJ, Smart NA (2014). Isometric exercise training for blood pressure management: a systematic review and meta-analysis. Mayo Clin Proc.

[52] Inder JD, Carlson DJ, Dieberg G, McFarlane JR, Hess NC, Smart NA (2016). Isometric exercise training for blood pressure management: a systematic review and meta-analysis to optimize benefit. Hypertens Res.

[53] Leitzmann MF, Park Y, Blair A, Ballard-Barbash R, Mouw T, Hollenbeck AR (2007). Physical activity recommendations and decreased risk of mortality. Arch Intern Med.

[54] Rossi A, Dikareva A, Bacon SL, Daskalopoulou SS (2012). The impact of physical activity on mortality in patients with high blood pressure: a systematic review. J Hypertens.

[55] Piepoli MF, Hoes AW, Agewall S, Albus C, Brotons C, Catapano AL (2016). 2016 European Guidelines on cardiovascular disease prevention in clinical practice. The Sixth Joint Task Force of the European Society of Cardiology and Other Societies on Cardiovascular Disease Prevention in Clinical Practice (constituted by representatives of 10 societies and by invited experts). Eur Heart J.

[56] MacMahon S (1987). Alcohol consumption and hypertension. Hypertension.

[57] Lang T, Cambien F, Richard JL, Bingham A (1987). Mortality in cerebrovascular diseases and alcoholism in France. Presse Med.

[58] Fuchs FD, Chambless LE, Folsom AR, Eigenbrodt ML, Duncan BB, Gilbert A (2004). Association between alcoholic beverage consumption and incidence of coronary heart disease in whites and blacks: the Atherosclerosis Risk in Communities Study. Am J Epidemiol.

[59] World Health Organization.(WHO) (2014). Global status report on alcohol and health.

[60] Johnson HM (2019). Anxiety and Hypertension: Is There a Link? A Literature Review of the Comorbidity Relationship Between Anxiety and Hypertension. Curr Hypertens Rep.

[61] Dalmazo AL, Fetter C, Goldmeier S, Irigoyen MC, Pelanda LC, Barbosa ECD (2019). Stress and Food Consumption Relationship in Hypertensive Patients. Arq Bras Cardiol.

[62] Denollet J, Gidron Y, Vrints CJ, Conraads VM (2010). Anger, suppressed anger, and risk of adverse events in patients with coronary artery disease. Am J Cardiol.

[63] Bai Z, Chang J, Chen C, Li P, Yang K, Chi I (2015). Investigating the effect of transcendental meditation on blood pressure: a systematic review and meta-analysis. J Hum Hypertens.

[64] Tankeu AT, Agbor VN, Noubiap JJ (2017). Calcium supplementation and cardiovascular risk: A rising concern. J Clin Hypertens.

[65] Manson JE, Cook NR, Lee IM, Christen W, Bassuk SS, Mora S (2018). Vitamin D Supplements and Prevention of Cancer and Cardiovascular Disease. N Engl J Med.

[66] Amoh-Mensah K, Ankomah SE, KariKari AK, Arthur JA (2017). Prevention of Hypertension: A critical review of the Health benefits of Salt, Garlic, Fish oil, Chocolate and Vitamin D. Int J Med Sci Tech.

[67] Paula TP, Kramer CK, Viana LV, Azevedo MJ (2017). Effects of individual micronutrients on blood pressure in patients with type 2 diabetes: a systematic review and meta-analysis of randomized clinical trials. Sci Rep.

[68] Gröber U, Schimidt J, Kisters K (2015). Magnesium in Prevention and Therapy. Nutrients.

[69] Padwal R, Hackam D, Khan N, Tobe S (2016). Primary prevention of CVD: modification of diet in people with hypertension. BMJ Clin Evid.

[70] Flowers N, Hartley L, Todkill D, Stranges S, Rees K (2014). Co-enzyme Q10 supplementation for the primary prevention of cardiovascular disease. Cochrane Database Syst Rev.

[71] National Center for Chronic Disease Prevention and Health Promotion (US) Office on Smoking and Health (2014). The Health Consequences of Smoking-50 Years of Progress: A Report of the Surgeon General.

[72] World Health Organization. (WHO) (2017). Report on the global tobacco epidemic.

[73] Center for Disease Control and Prevention (2010). How Tobacco Smoke Causes Disease: The Biology and Behavioural Basis for Smoking-attributable Disease. A Report of the Surgeon General.

[74] Jha P, Ramasundarahettige C, Landsman V, Rostron B, Thun M, Anderson RN (2013). 21st-centuryhazards of smoking and benefits of cessation in the United States. N Engl J Med.

[75] Khoramdad M, Vahedian-azimi A, Karimi L, Rahimi-Bashar F, Amini H, Sahebkar A (2020). Association between passive smoking and cardiovascular disease: A systematic review and meta-analysis. IUBMB Life.

[76] VanderWeele TJ, Balboni TA, Koh HK (2017). Health and Spirituality. JAMA.

[77] Cozier YC, Yu J, Wise LA, VanderWeele TJ, Balboni TA, Argentieri MA (2018). Religious and Spiritual Coping and Risk of Incident Hypertension in the Black Women’s Health Study. Ann Behav Med.

[78] Lewington S, Clarke R, Qizilbash N, Peto R, Collins C (2002). Age-specific relevance of usual blood pressure to vascular mortality: a meta-analysis of individual data for one million adults in 61 prospective studies. Lancet.

[79] Vasan RS, Larson MG, Leip EP, Evans JC, O’Donnell CJ, Kannel WB (2001). Impact of high-normal blood pressure on the risk of cardiovascular disease. N Engl J Med.

[80] Fukuhara M, Arima H, Ninomiya T, Hata J, Yonemoto K, Doi Y (2012). Impact of lower range of prehypertension events in a general population: the Hysayama Study. J Hypertens.

[81] Han M, Li Q, Liu L, Zhang D, Ren Y, Zhao Y (2019). Prehypertension and risk of cardiovascular diseases: a meta-analysis of 47 cohort studies. J Hypertens.

[82] Townsend RR, Wilkinson IB, Schiffrin EL, Avolio AP, Chirinos JA, Cockcroft JR (2015). Recommendations for Improving and Standardizing Vascular Research on Arterial Stiffness. Hypertension.

[83] Law MR, Morris JK, Wald NJ (2009). Use of BP lowering drugs in the prevention of cardiovascular disease: meta-analysis of 147 randomised trials in the context of expectations from prospective epidemiological studies. BMJ.

[84] Bundy JD, Li C, Stuchlik P, Bu X, Bu X, Kelly TN (2017). Systolic blood pressure reduction and risk of cardiovascular disease and mortality: a systematic review and network meta-analysis. JAMA Cardiol.

[85] Ettehad D, Emdin CA, Kiran A, Anderson SG, Callender T, Emberson J (2016). Blood pressure lowering for prevention of cardiovascular disease and death: a systematic review and meta- analysis. Lancet.

[86] Wright JT, Williamson JD, Whelton PK, Snyder JK, Sink KM, Rocco MV, SPRINT Research Group (2015). A randomized trial of intensive versus standard blood-pressure control. N Engl J Med.

[87] Williamson JD, Supiano MA, Applegate WB, Berlowitz DR, Campbell RC, Chertow GM (2016). Intensive vs standard blood pressure control and cardiovascular disease outcomes in adults aged ≥75 years: a randomized clinical trial. JAMA.

[88] Ho JE, Enserro D, Brouwers FP, Kizer JR, Shah SJ, Psaty BM (2016). Predicting heart failure with preserved and reduced ejection fraction: The International Collaboration on Heart Failure Subtypes. Circ Heart Fail.

[89] Emdin CA, Anderson SG, Salimi-Khorshidi G, Woodward M, MacMahon S, Dwyer T (2017). Usual blood pressure, atrial fibrillation and vascular risk: evidence from 4.3 million adults. Int J Epidemiol.

[90] Rahimi K, Mohseni H, Kiran A, Tran J, Nazarzadeh M, Rahimian F (2018). Elevated blood pressure and risk of aortic valve disease: a cohort analysis of 5.4 million UK adults. Eur Heart J.

[91] Rahimi K, Mohseni H, Otto CM, Conrad N, Tran J, Nazarzadeh M (2017). Elevated blood pressure and risk of mitral regurgitation: A longitudinal cohort study of 5.5 million United Kingdom adults. PLoS Med.

[92] Emdin CA, Anderson SG, Callender T, Conrad N, Salimi-Khorshidi G, Mohseni H (2015). Usual blood pressure, peripheral arterial disease, and vascular risk: cohort study of 4.2 million adults. BMJ.

[93] Hsu CY, McCulloch CE, Darbinian J, Go AS, Iribarren C (2005). Elevated blood pressure and risk of end-stage renal disease in subjects without baseline kidney disease. Arch Intern Med.

[94] Kanno A, Kikuya M, Ohkubo T, Hashimoto T, Satoh M, Hirose T (2012). Pre-hypertension as a significant predictor of chronic kidney disease in a general population: the Ohasama Study. Nephrol. Dial Transplant.

[95] Emdin CA, Rothwell PM, Salimi-Khorshidi G, Kiran A, Conrad N, Callender T (2016). Blood pressure and risk of vascular dementia: evidence from a primary care registry and a cohort study of transient ischemic attack and stroke. Stroke.

[96] Walker KA, Sharrett R, Wu A, Schneider AL, Alber M, Lutsey PL (2019). Association of midlife to late-life blood pressure patterns with incident dementia. JAMA.

[97] Joas E, Bäckman K, Gustafson D, Ostling S, Waern M, Guo X (2012). Blood pressure trajectories from midlife to late life in relation to dementia in women followed for 37 years. Hypertension.

[98] Emdin CA, Anderson SG, Woodward M, Rahimi K (2015). Usual blood pressure and risk of new-onset diabetes. J Am Coll Cardiol.

[99] Ning L, Yang L (2017). Hypetension might be a risk factor for erectile dysfunction: a meta-analysis. Andrologia.

[100] Chakravarthy U, Wong TY, Fletcher A, Piault E, Evans C, Zlateva G (2010). Clinical risk factors for age-related macular degeneration: a systematic review and meta-analysis. BMC Ophthalmol.

[101] Fuchs FD (2018). Essentials of hypertension.

[102] Fuchs FD, Whelton PK (2020). High Blood Pressure and Cardiovascular Disease. Hypertension.

[103] Williamson JD, Pajewski NM, Auchus AP, Bryan RN, Chelune G, Cheung AK (2019). Effect of Intensive vs Standard Blood Pressure Control on Probable Dementia: A Randomized Clinical Trial. JAMA.

[104] Nasrallah IM, Pajewski NM, Auchus AP, Chelune G, Cheung AK, Cleveland ML, SPRINT Research Group (2019). Association of intensive versus standard blood pressure control with cerebral write matter lesions. JAMA.

[105] Markus MR, Stritzke J, Lieb W, Mayer B, Luchner A, Döring A (2008). Implications of persistent prehypertension for ageing-related changes in left ventricular geometry and function: the MONICA/KORA Augsburg study. J Hypertens.

[106] Santos AB, Gupta DK, Bello NA, Gori M, Claggett B, Fuchs FD (2016). Prehypertension is associated with abnormalities of cardiac structure and function in the atherosclerosis risk in communities Study. Am J Hypertens.

[107] Fuchs SC, Poli-de-Figueiredo Carlos E, Figueiredo JA, Scala LC, Whelton PK, Mosele F (2016). Effectiveness of chlorthalidone plus amiloride for the prevention of hypertension: the PREVER-Prevention randomized clinical trial. J Am Heart Assoc.

[108] World Health Organization. (WHO) (2002). The world health report 2002 - Reducing Risks, Promoting Healthy Life (Internet).

[109] Andersen SL, Sebastiani P, Dworkis DA, Feldman L, Perls TT (2012). Health span approximates life span among many supercentenarians: compression of morbidity at the approximate limit of life span. J Gerontol A Biol Sci Med Sci..

[110] Whelton SP, McEvoy JW, Shaw L, Psaty BM, Lima JAC, Budoff M (2020). Association of Normal Systolic Blood Pressure Level with Cardiovascular Disease in the Absence of Risk Factors. JAMA Cardiol.

[111] Kanel WB, Larson M (1993). Long Term epidemiologic prediction of coronary disease. The Framingham Experience. Cardiology.

[112] Konukoglu D, Uzun H (2017). Endothelial Dysfunction and Hypertension. Adv Exp Med Biol.

[113] Bautista LE, Lopez-Jaramillo P, Vera LM, Casas JP, Otero AP, Guaracao AI (2001). Is C-reactive protein an independent risk factor for essential hypertension?. J Hypertens.

[114] Boos CJ, Lip GY (2006). Is hypertension an inflammatory process?. Curr Pharm Des.

[115] Grundy SM (2003). Inflammation, hypertension, and the metabolic syndrome. JAMA.

[116] Corretti MC, Anderson TJ, Benjamin EJ, Celermajer D, Charbonneau F, Creager MA (2002). Guidelines for the ultrasound assessment of endothelial-dependent flow-mediated vasodilation of the brachial artery: a report of the International Brachial Artery Reactivity Task Force. J Am Coll Cardiol.

[117] Thijssen DHJ, Black MA, Pyke KE, Padilla J, Atkinson G, Harris RA (2011). Assessment of flow-mediated dilation in humans: a methodological and physiological guideline. Am J Physiol Heart Circ Physiol.

[118] Thijssen DHJ, Bruno RM, van Mil ACCM, Holder SM, Faita F, Greyling A (2019). Expert consensus and evidence-based recommendations for the assessment of flow-mediated dilation in humans. Eur Heart J.

[119] Moens AL (2005). Flow-mediated vasodilation: a diagnostic instrument, or an experimental tool?. Chest.

[120] Celermajer DS, Sorensen KE, Gooch VM, Spiegelhalter DJ, Miller OI, Sullivan ID (1992). Non-invasive detection of endothelial dysfunction in children and adults at risk of atherosclerosis. Lancet.

[121] Takase B, Uehata A, Akima T, Nagai T, Nishioka T, Hamabe A (1998). Endothelium-dependent flow-mediated vasodilation in coronary and brachial arteries in suspected coronary artery disease. Am J Cardiol.

[122] Ras RT, Streppel MT, Draijer R, Zock PL (2013). Flow-mediated dilation and cardiovascular risk prediction: a systematic review with meta-analysis. Int J Cardiol.

[123] Inaba Y, Chen JA, Bergmann SR (2010). Prediction of future cardiovascular outcomes by flow-mediated vasodilatation of brachial artery: a meta-analysis. Int J Cardiovasc Imaging.

[124] Green D, Jones H, Thijssen D, Cable NT, Atkinson G (2011). Flow-Mediated Dilation and Cardiovascular Event Prediction: Does Nitric Oxide Matter?. Hypertension.

[125] Soloviev MA (2011). Correction of endothelial dysfunction in patients with arterial hypertension. Bull Exp Biol Med.

[126] Persu A, Plaen JF (2004). Recent insights in the development of organ damage caused by hypertension. Acta Cardiologica.

[127] Laurent S, Boutouyrie P, Lacolley P (2005). Structural and Genetic Bases of Arterial Stiffness. Hypertension.

[128] Safar ME, Asmar R, Benetos A, Blacher J, Boutouyrie P, Lacolley P (2018). Interaction Between Hypertension and Arterial Stiffness. An Expert Reappraisal. Hypertension.

[129] Laurent S, Cockcroft J, Van Bortel L, Boutouyrie P, Giannattasio C, Hayoz D (2006). Expert consensus document on arterial stiffness: methodological issues and clinical applications. Eur Heart J.

[130] Fridez P, Makino A, Kakoi D, Miyazaki H, Meister J, Hayashi K (2002). Adaptation of Conduit Artery Vascular Smooth Muscle Tone to Induced Hypertension. Ann Biomed Eng.

[131] Bardy N, Merval R, Benessiano J, Samuel JL, Tedgui A (1996). Pressure and angiotensin-II synergistically induce aortic fibronectin expression in organ culture model of rabbit aorta. Circ Res.

[132] Humphrey JD, Dufrense E, Schwartz MA (2014). Mechanotransduction and extracellular matrix homeostasis. Nat Rev Mol Cell Biol.

[133] Liao D, Arnett DK, Tyroler HA, Riley WA, Chambless LE, Szklo M (1999). Arterial stiffness and the development of hypertension. Hypertension.

[134] Humphrey JD, Harrison DG, Figueroa CA, Lacolley P, Laurent S (2016). Central Artery Stiffness in Hypertension and Aging: A Problem with Cause and Consequence. Circ Res.

[135] Dernellis J, Panaretou M (2005). Aortic stiffness is an independent predictor of progression to hypertension in nonhypertensive subjects. Hypertension.

[136] Kaess BM, Rong J, Larson MG, Hamburg NM, Vita JA, Levy D (2012). Aortic stiffness, blood pressure progression, and incident hypertension. JAMA.

[137] Weisbrod RM, Shiang T, Al Sayah L, Fry JL, Bajpai S, Reinhart-King CA (2013). Arterial stiffening precedes systolic hypertension in diet-induced obesity. Hypertension.

[138] Van Gorp AW, van Ingen Schenau DS, Hoeks APG, Struijker Boudier HAJ, Mey JGR, Reneman RS (2000). In spontaneously hypertensive rats alterations in rat aortic wall properties precede development of hypertension. Am J Physiol.

[139] Vlachopoulos C, Xaplanteris P, Aboyans V, Brodmann M, Cífkova R, Cosentino F (2015). The role of vascular biomarkers for primary and secondary prevention. A position paper from the European Society of Cardiology Working Group on peripheral circulation Endorsed by the Association for Research into Arterial Structure and Physiology (ARTERY) Society. Atherosclerosis.

[140] Aboyans V, Criqui MH, Abraham P, Allison MA, Creager MA, Diehm C (2012). Measurement and interpretation of the ankle-brachial index: a scientific statement from the American Heart Association. Circulation.

[141] Rabkin SW, Him S, Sweeney C (2012). Ankle-Brachial Index as an Indicator of Arterial Stiffness in Patients Without Peripheral Artery Disease. Angiology.

[142] Umemura S, Arima H, Arima S, Asayama K, Dohi Y, Hirooka Y (2019). The Japanese Society of Hypertension Guidelines for the Management of Hypertension (JSH 2019). Hypertens Res.

[143] Fowkes FGR, Murray GD, Butcher I, Heald CL, Lee RJ, Chambless LE (2008). Ankle Brachial Index Combined with Framingham Risk Score to Predict Cardiovascular Events and Mortality: A Meta-Analysis. JAMA.

[144] Mattace-Raso FUS, Hofman A, Verwoert GC, Wittemana JCM, Wilkinson I, Cockcroft J (2010). Determinants of Pulse Wave Velocity in Healthy People and in the Presence of Cardiovascular Risk Factors: ‘Establishing Normal and Reference Values’. Eur Heart J.

[145] Boutouyrie P, Bruno RM (2019). The Clinical Significance and Application of Vascular Stiffness Measurements. Am J Hypertens.

[146] Van Bortel L, Laurent S, Boutouyrie P, Chowienczyk P, Cruickshank JK, Backer T (2012). Expert Consensus Document on the Measurement of Aortic Stiffness in Daily Practice Using Carotid-Femoral Pulse Wave Velocity. J Hypertens.

[147] Butlin M, Qasem A (2016). Large Artery Stiffness Assessment Using SphygmoCor Technology. Pulse.

[148] Weber T, Ammer M, Rammer M, Adji A, O’Rourke MF, Siegfried Wassertheurer S (2009). Noninvasive determination of carotid–femoral pulse wave velocity depends critically on assessment of travel distance: a comparison with invasive measurement. Journal of Hypertension.

[149] Stea F, Bozec E, Millasseau S, Khettab H, Boutouyrie P, Laurent S (2014). Comparison of the Complior Analyse device with Sphygmocor and Complior SP for pulse wave velocity and central pressure assessment. J Hypertens.

[150] Sztrymf B, Jacobs F, Chemla D, Richard C, Millasseau S (2013). Validation of the new Complior sensor to record pressure signals non-invasively. J Clin Monit Comput.

[151] Jones CR, Taylor K, Chowienczyk P, Poston L, Shennan AH (2000). A validation of the Mobil O Graph (version 12) ambulatory blood pressure monitor. Blood Pressure Monitoring.

[152] Hametner B, Wassertheurer S, Kropf J, Mayer C, Eber B, Weber T (2013). Oscillometric estimation of aortic pulse wave velocity: comparison with intra-aortic catheter measurements. Blood Press Monit.

[153] Laurent S, Boutouyrie P, Asmar R, Gautier I, Laloux B, Guize L (2001). Aortic stiffness is an independent predictor of all-cause and cardiovascular mortality in hypertensive patients. Hypertension.

[154] Boutouyrie P, Tropeano AI, Asmar R, Gautier I, Benetos A, Lacolley P (2002). Aortic stiffness is an independent predictor of primary coronary events in hypertensive patients: a longitudinal study. Hypertension.

[155] Vlachopoulos C, Aznaouridis K, Stefanadis C (2010). Prediction of cardiovascular events and all-cause mortality with arterial stiffness: a systematic review and meta-analysis. J Am Coll Cardiol.

[156] Ben-Shlomo Y, Spears M, Boustred C, May M, Anderson SG, Benjamin EJ (2014). Aortic pulse wave velocity improves cardiovascular event prediction: an individual participant meta-analysis of prospective observational data from 17,635 subjects. J Am Coll Cardiol.

[157] Mitchell GF, Hwang SJ, Vasan RS, Larson MG, Pencina MJ, Hamburg NM (2010). Arterial stiffness and cardiovascular events: the Framingham Heart Study. Circulation.

[158] Xaplanteris P, Vlachopoulos C, Protogerou AD, Aznaouridis K, Terentes-Printzios D, Argyris AA (2019). A clinical score for prediction of elevated aortic stiffness: derivation and validation in 3943 hypertensive patients. J Hypertens.

[159] Paiva AMG, Mota-Gomes MA, Brandão AA, Silveira FS, Silveira MS, Okawa RTP (2020). Reference values of office central blood pressure, pulse wave velocity, and augmentation index recorded by means of the Mobil-O-Graph PWA monitor. Hypertens Res.

[160] Vlachopoulos C, Aznaouridis K, O’Rourke MF, Safar M, Baou K, Stefanadis C (2013). Prediction of cardiovascular events and all-cause mortality with central haemodynamics: a systematic review and meta-analysis. Eur Heart J.

[161] Ding FH, Fan WX, Zhang RY, Zhang Q, Li Y, Wang JG (2011). Validation of the Noninvasive Assessment of Central Blood Pressure by the SphygmoCor and Omron Devices Against the Invasive Catheter Measurement. American Journal of Hypertension.

[162] Pereira T, Maldonado J, Coutinho R, Cardoso E, Laranjeiro M, Andrade I (2014). Invasive validation of the Complior Analyse in the assessment of central artery pressure curves: a methodological study. Blood Press Monit.

[163] Herbert A, Cruickshank JK, Laurent S, Boutouyrie P, Collaboration on behalf of The Reference Values for Arterial Measurements (2014). Establishing reference values for central blood pressure andi ts amplification in a general healthy population and according to cardiovascular risk factors. Eur Heart J.

[164] Malachias MVB, Souza WKSB, Plavnik FL, Rodrigues CIS, Brandão AA, Neves MFT (2016). 7ª Diretriz Brazileira de Hipertensão Arterial. Arq Bras Cardiol.

[165] Stergiou GS, Alpert B, Mieke S, Asmar R, Atkins N, Eckert S (2018). A universal standard for the validation of blood pressure measuring devices: Association for the Advancement of Medical Instrumentation/European Society of Hypertension/International Organization for Standardization (AAMI/ESH/ISO) Collaboration Statement. J Hypertens.

[166] Brazil, Ministério do Desenvolvimento, Indústria e Comércio Exterior, Instituto Nacional de Metrologia, Qualidade e Tecnologia Portaria n.46 de 22 de janeiro de 2016. Esfigmomanometros.

[167] Clark CE, Taylor RS, Shore AC, Ukoumunne OC, Campbell JL (2012). Association of a difference in systolic blood pressure between arms with vascular disease and mortality: a systematic review and meta-analysis. Lancet.

[168] Saedon NI, Pin Tan M, Frith J (2020). The Prevalence of Orthostatic Hypotension: A Systematic Review and Meta-Analysis. J Gerontol A Biol Sci Med Sci.

[169] Fagard RH, Cort P (2010). Orthostatic hypotension is a more robust predictor of cardiovascular events than nighttime reverse dipping in elderly. Hypertension.

[170] Leung AA, Daskalopoulou SS, Dasgupta K, McBrien K, Butalia S, Zarnke KB. (2017). Hypertension Canada’s 2017 Guidelines for Diagnosis, Risk Assessment, Prevention, and Treatment of Hypertension in Adults. Can J Cardiol.

[171] Myers MG (2016). A short history of automated office blood pressure - 15 years to SPRINT. J Clin Hypertens (Greenwich).

[172] Parati G, Pomidossi G, ParatiR Casadei, Mancia G (1985). Lack of alerting reactions to intermitent cuff inflations during noninvasive blood pressure monitoring. Hypertension.

[173] Myers MG, Godwin M, Dawes M, Kiss A, Tobe SW, Kaczorowski J (2010). Measurement of blood pressure in the office: recognizing the problem and proposing the solution. Hypertension.

[174] Palatini P, Asmar R (2018). Cuff challenges in blood pressure measurement. J Clin Hypertens.

[175] Leblanc MÈ, Auclair A, Leclerc J, Bussières J, Agharazii M, Hould FS (2019). Blood pressure measurement in severely obese patients: validation of the forearm approach in different arm positions. Am J. Hypertens.

[176] Pickering TG, Hall JE, Appel LJ, Falkner BE, Graves J, Hill MN (2005). Recommendations for blood pressure measurement in humans and experimental animals: part 1: blood pressure measurement in humans: a statement for professionals from the Subcommittee of Professional and Public Education of the American Heart Association Council on High Blood Pressure Research. Circulation.

[177] Senarclens (2008). Brachial or wrist blood pressure in obese patients:which is the best?. Blood Pressure Monitoring.

[178] Irving G, Holden J, Stevens R, McManus RJ (2016). Which cuff should I use? Indirect blood pressure measurement for the diagnosis of hypertension in patients with obesity: a diagnostic accuracy review. BMJ Open.

[179] Fuchs FD, Scala LC, Vilela-Martin JF, de Mello RB, Mosele F, Whelton PK (2016). Effectiveness of chlorthalidone/amiloride versus losartan in patients with stage I hypertension: results from the PREVER-treatment randomized trial. J Hypertens.

[180] Feitosa ADM, Mota-Gomes MA, Barroso WS, Miranda RD, Barbosa ECD, Pedrosa RP (2020). Relationship between office isolated systolic or diastolic hypertension and white-coat hypertension across the age spectrum: a home blood pressure study. J Hypertens.

[181] Weber MA, Schiffrin EL, White WA, Mann S, Lindbolm LH, Venerson JG (2014). Clinical practice guidelines for the management of hypertension in the community: a statement by the American Society of Hypertension and the International Society of Hypertension. J Hypertens.

[182] Egan BM, Stevens-Fabry S (2015). Prehypertension-prevalence, health risks, and management strategies. Nat Rev Cardiol.

[183] Parati G, Stergiou G, O’Brien E, Asmar R, Beilin L, Bilo G, European Society of Hypertension Working Group on Blood Pressure Monitoring and Cardiovascular Variability (2014). European Society of Hypertension practice guidelines for ambulatory blood pressure monitoring. J Hypertens.

[184] Stergiou GS, Parati G, Vlachopoulos C, Achimastos A, Andreadis E, Asmar R (2016). Methodology and technology for peripheral and central blood pressure and blood pressure variability measurement: current status and future directions - Position statement of the European Society of Hypertension Working Group on blood pressure monitoring and cardiovascular variability. J Hypertens.

[185] O’Brien E, Parati G, Stergiou G, Asmar R, Beilin L, Bilo G, European Society of Hypertension Working Group on Blood Pressure M. (2013). European Society of Hypertension position paper on ambulatory blood pressure monitoring. Hypertens.

[186] Nobre F, Mion D, Gomes MAM, Barbosa ECD, Rodrigues CIS, Neves MFT (2018). 6ª Diretrizes de Monitorização Ambulatorial da Pressão Arterial e 4ª Diretrizes de Monitorização Residencial da Pressão Arterial. Arq Bras Cardiol.

[187] Souza WK, Jardim PC, Porto LB, Araújo FA, Sousa AL, Salgado CM (2011). Comparison and correlation between self-measured blood pressure, casual blood pressure measurement and ambulatory blood pressure monitoring. Arq Bras Cardiol.

[188] Bliziotis IA, Destounis A, Stergiou GS (2012). Home versus ambulatory and office blood pressure in predicting target organ damage in hypertension: a systematic review and meta-analysis. J Hypertens.

[189] WK, Jardim PC, Brito LP, Araújo FA, Sousa AL (2012). Self measurement of blood pressure for control of blood pressure levels and adherence to treatment. Arq Bras Cardiol.

[190] Park JS, Rhee MY, Namgung J, Lee SY, Cho DK, Choi TY (2017). Comparison of Optimal Diagnostic Thresholds of Hypertension With Home Blood Pressure Monitoring and 24-Hour Ambulatory Blood Pressure Monitoring. Am J Hypertens.

[191] Niiranen TJ, Asayama K, Thijs L, Johansson JK, Ohkubo T (2012). Outcome-Driven Thresholds for Home Blood Pressure Measurement: International Database of HOme blood pressure in relation to Cardiovascular Outcome. Hypertension.

[192] D, Asayama K, Ohkubo T, Kikuya M, Kanno A, Hara A (2010). Stroke Risk in Treated Hypertension Based on Home Blood Pressure: the Ohasama Study. Am J Hypertens.

[193] Ward AM, Takahashi O, Stevens R, Heneghan C (2012). Home measurement of blood pressure and cardiovascular disease: systematic review and meta-analysis of prospective studies. J Hypertens.

[194] McManus RJ, Mant J, Bray EP, Holder R, Jones MI, Greenfield S (2010). Telemonitoring and selfmanagement in the control of hypertension (TASMINH2): a randomised controlled trial. Lancet.

[195] McManus RJ, Mant J, Haque MS, Bray EP, Bryan S, Greenfield SM (2014). Effect of self-monitoring and medication self-titration on systolic blood pressure in hypertensive patients at high risk of cardiovascular disease: the TASMIN-SR randomized clinical trial. JAMA.

[196] Tucker KL, Sheppard JP, Stevens R, Bosworth HB, Bove A, Bray EP (2017). Self-monitoring of blood pressure in hypertension: a systematic review and individual patient data meta-analysis. PLoS One.

[197] Omboni S, Gazzola T, Carabelli G, Parati G (2013). Clinical usefulness and cost effectiveness of home blood pressure telemonitoring: meta-analysis of randomized controlled studies. J Hypertens.

[198] Parati G, Omboni S (2010). Role of home blood pressure telemonitoring in hypertension management: an update. Blood Press Monit.

[199] Gaborieau V, Delarche N, Gosse P (2008). Ambulatory blood pressure monitoring versus self-measurement of blood pressure at home: correlation with target organ damage. J Hypertens.

[200] Clement DL, Buyzere ML, Bacquer DA, Leeuw PW, Duprez DA, Fagard RH (2003). Prognostic value of ambulatory blood-pressure recordings in patients with treated hypertension. N Engl J Med.

[201] Sega R, Facchetti R, Bombelli M, Cesana G, Corrao G, Grassi G (2005). Prognostic value of ambulatory and home blood pressures compared with office blood pressure in the general population: follow-up results from the Pressioni Arteriose Monitorate e Loro Associazioni (PAMELA) study. Circulation.

[202] ABC-H Investigators, Roush GC, Fagard RH, Salles GF, Pierdomenico SD, Reboldi G, Verdecchia P (2014). Prognostic impact from clinic, daytime, and night-time systolic blood pressure in nine cohorts of 13,844 patients with hypertension. J Hypertens.

[203] Fagard RH, Celis H, Thijs L, Staessen JA, Clement DL, Buyzere ML (2008). Daytime and nighttime blood pressure as predictors of death and cause-specific cardiovascular events in hypertension. Hypertension.

[204] Parati G, Ochoa JE, Bilo G, Agarwal R, Covic A, Dekker FW (2016). Hypertension in chronic kidney disease part 2: role of ambulatory and home blood pressure monitoring for assessing alterations in blood pressure variability and blood pressure profiles. Hypertension.

[205] Piper MA, Evans CV, Burda BU, Margolis KL, O’Connor E, Whitlock EP (2015). Diagnostic and predictive accuracy of blood pressure screening methods with consideration of rescreening intervals: a systematic review for the U.S. Preventive Services Task Force. Ann Intern Med.

[206] Mancia G, Zanchetti A (1996). White-coat hypertension: misnomers, misconceptions and misunderstandings. What should we do next?. J Hypertens.

[207] Bobrie G, Clerson P, Menard J, Postel-Vinay N, Chatellier G, Plouin PF (2008). Masked hypertension: a systematic review. J Hypertens.

[208] Feitosa ADM, Mota-Gomes MA, Miranda RD, Barroso WS, Barbosa ECB, Pedrosa RP (2018). Impact of 2017 ACC/AHA hypertension guidelines on the prevalence of white-coat and masked hypertension: A home blood pressure monitoring study. J Clin Hypertens.

[209] Paiva AMG, Gomes MICM, Campana EMG, Feitosa ADM, Sposito AC, Mota-Gomes MA (2019). Impact of hypertension phenotypes on the office and 24-h pulse wave velocity and augmentation index in individuals with or without antihypertensive medication use. Hypertens Res.

[210] Fagard RH, Cornelissen VA (2007). Incidence of cardiovascular events in whitecoat, masked and sustained hypertension versus true normotension: a meta-analysis. J Hypertens.

[211] Staessen JA, O’Brien ET, Amery AK, Atkins N, Baumgart P, Cort P (1994). Ambulatory blood pressure in normotensive and hypertensive subjects: results from an international database. J Hypertens.

[212] Barroso WKS, Feitosa ADM, Barbosa ECD, Miranda RD, Vitorino PVO, Brandão AA (2019). Prevalence of Masked and White-Coat Hypertension in Pre-Hypertensive and Stage 1 Hypertensive patients with the use of TeleMRPA. Arq Bras Cardiol.

[213] Huang Y, Huang W, Mai W, Cai X, An D, Liu Z (2017). White-coat hypertension is a risk factor for cardiovascular diseases and total mortality. J Hypertens.

[214] Briasoulis A, Androulakis E, Palla M, Papageorgiou N, Tousoulis D (2016). White-coat hypertension and cardiovascular events: a meta-analysis. J Hypertens.

[215] Kikuya M, Ohkubo T, Metoki H, Asayama K, Hara A, Obara T (2008). Day-by-day variability of blood pressure and heart rate at home as a novel predictor of prognosis: the Ohasama study. Hypertension.

[216] Rassi G, Seravalle G, Trevano FQ, Dell’oro R, Bolla G, Cuspidi C, Arenare F, Mancia G (2007). Neurogenic abnormalities in masked hypertension. Hypertension.

[217] Parati G, Omboni S, Staessen J, Thijs L, Fagard R, Ulian L, Syst-Eur investigators (1998). Limitations of the difference between clinic and daytime blood pressure as a surrogate measure of the ‘white-coat’ effect. J Hypertens.

[218] Banegas JR, Ruilope LM, de la Sierra A, de la Cruz JJ, Gorostidi M, Segura J (2014). High prevalence of masked uncontrolled hypertension in people with treated hypertension. Eur Heart J.

[219] Mancia G (2016). Clinical significance of white-coat hypertension. J Hypertens.

[220] Mancia G (2017). White-coat hypertension: growing evidence in favour of its adverse prognostic significance. J Hypertens.

[221] Mancia G, Grassi G (2016). The heterogeneous nature of white-coat hypertension. J Am Coll Cardiol.

[222] Mancia G, Bombelli M, Cuspidi C, Facchetti R, Grassi G (2017). Cardiovascular risk associated with white-coat hypertension: pro side of the argument. Hypertension.

[223] Mancia G, Fagard R, Narkiewicz K, Redon J, Zanchetti A, Bohm M (2013). 2013 ESH/ESC guidelines for the management of arterial hypertension: the Task Force for the Management of Arterial Hypertension of the European Society of Hypertension (ESH) and of the European Society of Cardiology (ESC). Eur Heart J.

[224] Pierdomenico SD, Cuccurullo F (2011). Prognostic value of white-coat and masked hypertension diagnosed by ambulatory monitoring in initially untreated subjects: an updated meta analysis. Am J Hypertens.

[225] Lurbe E, Torro I, Alvarez V, Nawrot T, Paya R, Redon J, Staessen JA (2005). Prevalence, persistence, and clinical significance of masked hypertension in youth. Hypertension.

[226] Mancia G, Facchetti R, Bombelli M, Grassi G, Sega R (2006). Long-term risk of mortality associated with selective and combined elevation in office, home, and ambulatory blood pressure. Hypertension.

[227] Bobrie G, Chatellier G, Genes N, Clerson P, Vaur L, Vaisse B (2004). Cardiovascular prognosis of “masked hypertension” detected by blood pressure self-measurement in elderly treated hypertensive patients. JAMA.

[228] Franklin SS, Thijs L, Li Y, Hansen TW, Boggia J, Liu Y (2013). Response to masked hypertension in untreated and treated patients with diabetes mellitus: attractive but questionable interpretations and response to Is masked hypertension related to diabetes mellitus?. Hypertension.

[229] Chow CK, Teo KK, Rangarajan S, Islam S, Gupta R, Avezum A (2013). Prevalence, awareness, treatment, and control of hypertension in rural and urban communities in high-, middle-, and low-income countries. JAMA.

[230] Lindholt JS, Sogaard R (2017). Population screening and intervention for vascular disease in Danish men (VIVA): a randomised controlled trial. Lancet.

[231] Hodgkinson J, Mant J, Martin U, Guo B, Hobbs FD, Deeks JJ (2011). Relative effectiveness of clinic and home blood pressure monitoring compared with ambulatory blood pressure monitoring in diagnosis of hypertension: systematic review. BMJ.

[232] Vinyoles E, Felip A, Pujol E, de la Sierra A, Dura R, del Rey RH (2008). Clinical characteristics of isolated clinic hypertension. J Hypertens.

[233] Picone DS, Schultz MG, Otahal P, Aakhus S, Al-Jumaily AM, Black JA (2017). Accuracy of cuff-measured blood pressure: systematic reviews and meta-analyses. J Am Coll Cardiol.

[234] Williams B, Lacy PS, Thom SM, Cruickshank K, Stanton A, Collier D (2006). Differential impact of blood pressure-lowering drugs on central aortic pressure and clinical outcomes: principal results of the Conduit Artery Function Evaluation (CAFE) study. Circulation.

[235] Lurbe E, Redon J (2016). Isolated systolic hypertension in young people is not spurious and should be treated: con side of the argument. Hypertension.

[236] McEniery CM, Franklin SS, Cockcroft JR, Wilkinson IB (2016). Isolated systolic hypertension in young people is not spurious and should be treated: pro side of the argument. Hypertension.

[237] Miall WE, Oldham PD (1963). The hereditary factor in arterial blood-pressure. BMJ.

[238] Snieder H, Hayward CS, Perks U, Kelly RP, Kelly PJ, Spector TD SO (2000). Heritability of central systolic pressure augmentation: a twin study. Hypertension.

[239] Evangelou E, Warren HR, Mosen-Ansorena D, Mifsud B, Pazoki R, Gao H (2018). Genetic analysis of over 1 million people identifies 535 new loci associated with blood pressure traits. Nat Genet.

[240] Raina R, Krishnappa V, Das A, Amin H, Radhakrishnan Y, Nair NR (2019). Overview of Monogenic or Mendelian Forms of Hypertension. Front Pediatr.

[241] Favier J, Amar L, Gimenez-Roqueplo AP (2015). Paraganglioma and phaeochromocytoma: from genetics to personalized medicine. Nat Rev Endocrinol.

[242] Lenders JW, Duh QY, Eisenhofer G, Gimenez-Roqueplo AP, Grebe SK, Murad MH (2014). Pheochromocytoma and paraganglioma: an endocrine society clinical practice guideline. J Clin Endocrinol Metab.

[243] Murabito JM, Nam BH, D’Agostino RB, Lloyd-Jones DM, O’Donnell CJ, Wilson PW (2004). Accuracy of offspring reports of parenteral cardiovascular disease history: the Framingham Offspring Study. Ann Intern Med.

[244] Chobanian AV, Bakris GL, Black HR, Cushman WC, Green LA, Izzo J (2003). Seventh Report of the Joint National Committee on Prevention, Detection, Evaluation, and Treatment of High Blood Pressure. Hypertension.

[245] Berry JD, Dyer A, Cai X, Garside DB, Ning H, Thomas A (2012). Lifetime risks of cardiovascular disease. N Engl J Med.

[246] Wilson PW, Kannel WB, Silbershatz H, D’Agostino RB (1999). Clustering of metabolic factors and coronary heart disease. Arch Intern Med.

[247] Egan BM, Li J, Hutchison FN, Ferdinand KC (2014). Hypertension in United States, 1999 to 2012: progress toward Healthy People 2020 goals. Circulation.

[248] Obesity Classification – World Obesity Federation https://www.worldobesity.org/about/about-obesity/obesity-classification?_ga=2.27200504.476223329.1582981112-571126236.1582981112.

[249] Carter SA (1968). Indirect systolic pressures and pulses waves in arterial occlusive disease of the lower extremities. Circulation.

[250] Newman AB, Siscovick DS, Manolio TA, Polak J, Fried LP, Borhani NO (1993). Ankle-arm index as a marker of atherosclerosis in the Cardiovascular Health Study. Cardiovascular Health Study (CHS) Collaborative Reserch Group. Circulation.

[251] Rourke MF, Adji A (2013). Guidelines on guidelines: focus on isolated systolic hypertension in youth. J Hypertens.

[252] Brandão AA, Amodeo C, Alcantara C, Barbosa E, Nobre F, Pinto F (2017). I Posicionamento Luso-Brazileiro de Pressão Arterial Central. Arq Bras Cardiol.

[253] Mancia G, Narkiewiczk, Redon J, Zanchetti A, Bohm M, Christiaens T (2013). 2013 ESH/ESC Guidelines for the management of arterial hypertension: theTask Force for the management of arterial hypertension of the European Society of Hypertension (ESH) and of the European Society of Cardiology (ESC). J Hypertens.

[254] D’Agostino RB, Vasan RS, Pencina MJ, Wolf PA, Cobain M, Massaro JM, WBl Kannel (2008). General cardiovascular risk profile for use in primary care: the Framingham Heart Study. Circulation.

[255] Levey AS, Bosch JP, Lewis JB, Greene LT, Rogers N, Roth D, Modification of Diet in Renal Disease Study Group (1999). A more accurate accurate method to estimate GFR from serum creatinine: a new prediction equation. Ann Intern Med.

[256] (2009). A New Equation to Estimate Glomerular Filtration Rate. Chronic Kidney Disease Epidemiology Collaboration (CKD-EPI). Ann Intern Med.

[257] Levey AS, Eckardt K-U, Dorman NM, Christiansen SL, Hoorn EJ, Ingelfinger JR (2020). Nomenclature for kidney function and disease: report of a Kidney Disease: Improving Global Outcomes (KDIGO) Consensus Conference. Kidney Inter.

[258] KDIGO CKD Work Group (2013). KDIGO 2012 clinical practice guideline for the evaluation and management of chronic kidney disease. Kidney Int.

[259] Friedewald WT, Levi RI, Fredrickson DS (1972). Estimation of the concentration of low density lipoproteins cholesterol in plasma without use of the ultracentrifuge. Clin Chem.

[260] Sokolow M, Lyon TP (1949). The ventricular complex in left ventricular hypertrophy as obtained by unipolar precordial and limb leads. Am Heart J.

[261] Casalle PN, Devereux RB, Alonso DR, Campo E, Kligfield P (1987). Improved sex specific criteria of left ventricular hypertrophy for clinical and computer interpretation of electrocadiograms: validation with autopsy findings. Circulation.

[262] Rayner BL, Goodman H, Opie LH (2004). The Chest Radiograph. A useful investigationin the evaluation of hypertensive patients. Am J Hypertens.

[263] Marwick TH, Gillebert TC, Aurigemma G, Chirinos J, Derumeaux G, Galderisi M (2015). Recommendations on the Use of Echocardiography in Adult Hypertension: A Report from the European Association of Cardiovascular Imaging (EACVI) and the American Society of Echocardiography (ASE). J Am Soc Echocardiogr.

[264] Tsioufis C, Kokkinos P, Macmanus C, Thomopoulos C, Faselis C, Doumas M (2010). Left ventricular hypertrophy as a determinant of renal outcome in patients with high cardiovascular risk. J Hypertens.

[265] Nambi V, Chambless L, Folsom AR, He M, Hu Y, Mosley T (2010). Carotid intima-media thickness and presence or absence of plaque improves prediction of coronary heart disease risk: the ARIC (Atherosclerosis Risk In Communities) study. J Am Coll Cardiol.

[266] Polak JF, Szklo M, O’Leary DH (2017). Carotid intima-media thickness score, positive coronary artery calcium score, and incident coronary heart disease: the Multi-Ethnic Study of Atherosclerosis. J Am Heart Assoc.

[267] Vasbinder GB, Nelemans PJ, Kessels AG, Kroon AA, Leeuw PW, van Engelshoven JM (2001). Diagnostic tests for renal artery stenosis in patients suspected of having renovascular hypertension: a meta-analysis. Ann Intern Med.

[268] Selvin E, Steffes MW, Zhu H, Matsushita K, Wagenknecht L, Pankow J (2010). Brancati Glycated hemoglobin, diabetes, and cardiovascular risk in nondiabetic adults. N Engl J Med.

[269] Chin D, Battistoni A, Tocci G, Passerini J, Parati G, Volpe M (2012). Non-invasive diagnostic testing for coronary artery disease in the hypertensive patient: potential advantages of a risk estimation-based algorithm. Am J Hypertens.

[270] Safar ME, Levy BI, Struijker-Boudier H (2003). Current perspectives on arterial stiffness and pulse pressure in hypertension and cardiovascular diseases. Circulation.

[271] Mikael LR, Paiva AMG, Mota-Gomes M, Sousa ALL, Jardim PCBV, Vitorino PVO (2017). Envelhecimento vascular e rigidez arterial. Arq Bras Cardiol.

[272] Leeuw FE, Groot JC, Oudkerk M, Witteman JC, Hofman A, van Gijn J, Breteler MMB (2002). Hypertension and cerebral white matter lesions in a prospective cohort study. Brain.

[273] NCD Risk Factor Collaboration (2017). Worldwide trends in blood pressure from 1975 to 2015: a pooled analysis of 1479 population-based measurement studies with 19.1 million participants. Lancet.

[274] Kearney PM, Whelton M, Reynolds K, Muntner P, Whelton PK, He J (2005). Global Burden of hypertension: analysis of worldwide data. Lancet.

[275] Ungera T, Borghib C, Charcharc F, Khanf NA, Poulterh NR, Prabhakarani D (2020). International Society of Hypertension global hypertension practice guidelines. Hypertension.

[276] Wilson PW, D’Agostino RB, Levy D, Belanger AM, Silbershatz H, Kannel WB (1998). Prediction of coronary heart disease using risk factor categories. Circulation.

[277] Neaton JD, Wentworth D (1992). Serum cholesterol, blood pressure, cigarette smoking, and death from coronary heart disease. Overall findings and differences by age for 316,099 white men. Multiple Risk Factor Intervention Trial Research Group. Arch Intern Med.

[278] Blood Pressure Lowering Treatment Trialists C (2014). Blood pressure-lowering treatment based on cardiovascular risk: a meta-analysis of individual patient data. Lancet.

[279] Matsura Y, Kanter JE, Bornfeldt KE (2019). Highlighting Residual Atherosclerotic Cardiovascular Disease. Arterioscler Thromb Vasc Biol.

[280] Grundy SM, Stone NJ, Bailey AL, Beam C, Birtcher KK, Blumenthal RS (2019). AHA/ACC/AACVPR/AAPA/ABC/ACPM/ADA/AGS/APhA/ASPC/NLA/PCNA Guideline on the Management of Blood Cholesterol: A Report of the American College of Cardiology/American Heart Association Task Force on Clinical Practice Guidelines. Circulation.

[281] Mach F, Baigent C, Catapano AL, Koskinas KC, Casula M, Badimon L (2020). ESC/EAS Guidelines for the management of dyslipidaemias: lipid modification to reduce cardiovascular risk 2020. Eur Heart J.

[282] Sposito AC, Ramires JA, Jukema JW, Molina JC, Silva PM, Ghadanfar MM, Wilson PW (2009). Physicians’ attitudes and adherence to use of risk scores for primary prevention of cardiovascular disease: cross-sectional survey in three world regions. Curr Med Res Opin.

[283] Hlatky MA, Greenland P, Arnett DK, Ballantyne CM, Criqui MH, Elkin (2009). Criteria for evaluation of novel markers of cardiovascular risk: a scientific statement from the American Heart Association. Circulation.

[284] Cooney MT, Dudina AL, Graham IM (2009). Value and Limitations of Existing Scores for the Assessment of Cardiovascular Risk A Review for Clinicians. J Am Coll Cardiol.

[285] Rapsomaniki E, Timmis A, George J (2014). Blood pressure and incidence of twelve cardiovascular diseases: lifetime risks, healthy life-years lost, and age-specific associations in 1.25 million people. Lancet.

[286] Yusuf S, Hawken S, Ounpuu S, Dans T, Avezum A, Lanas F (2004). Effect of potentially modifiable risk factors associated with myocardial infarction in 52 countries (the INTERHEART study): case-control study. Lancet.

[287] Wang OJ, Wang Y, Chen J, Krumholz HM (2012). Recent Trends in Hospitalization for Acute Myocardial Infarction. Am J Cardiol.

[288] Tunstall-Pedoe H, Kuulasmaa K, Amouyel P, Arveiler D, Rajakangas AM, Pajak A (1994). Myocardial infarction and coronary deaths in the World Health Organization MONICA Project. Registration procedures, event rates, and case-fatality rates in 38 populations from 21 countries in four continents. Circulation.

[289] International Diabetes Federation (2006). IDF Consensus Worldwide Definition of the Metabolic Syndrome.

[290] Perrone-Filardi P, Coca A, Galderisi M, Paolillo S, Alpendurada F, Simone G (2017). Noninvasive cardiovascular imaging for evaluating subclinical target organ damage in hypertensive patients: a consensus article from the European Association of Cardiovascular Imaging, the European Society of Cardiology Council on Hypertension and the European Society of Hypertension. J Hypertens.

[291] PROGRESS Collaborative Group (2001). Randomised trial of a perindopril-based blood-pressure-lowering regimen among 6,105 individuals with previous stroke or transient ischaemic attack. Lancet.

[292] Manning LS, Mistri AK, Potter J, Rothwell PM, Robinson TG (2015). Short-term blood pressure variability in acute stroke: post hoc analysis of the controlling hypertension and hypotension immediately post stroke and continue or stop post-stroke antihypertensives collaborative study trials. Stroke.

[293] Grundvold I, Skretteberg PT, Liestol K, Erikssen G, Kjeldsen SE, Arnesen H, Erikssen J, Bodegard J (2012). Upper normal blood pressures predict incident atrial fibrillation in healthy middle-aged men: a 35-year follow-up study. Hypertension.

[294] Singer DR, Kite A (2008). Management of hypertension in peripheral arterial disease: does the choice of drugs matter?. Eur J Vasc Endovasc Surg.

[295] Sehestedt T, Jeppesen J, Hansen TW, Wachtell K, Ibsen H, Torp-Petersen C (2010). Risk prediction is improved by adding markers of subclinical organ damage to SCORE. Eur Heart J.

[296] Orlova IA, Nuraliev EY, Yarovaya EB, Ageev FT (2010). Prognostic value of changes in arterial stiffness in men with coronary artery disease. Vasc Health Risk Manag.

[297] Cecelja M, Chowienczyk P (2009). Dissociation of aortic pulse wave velocity with risk factors for cardiovascular disease other than hypertension: a systematic review. Hypertension.

[298] Chirinos JA, Segers P, Hughes T, Townsend R (2019). Large-Artery Stiffness in Health and Disease JACC State-of-the-Art Review. J Am Coll Cardiol.

[299] Bertoluci MC, Moreira RO, Faludi A, Izar MC, Schaan BD, Valerio CM (2017). Brazilian guidelines on prevention of cardiovascular disease in patients with diabetes: a position statement from the Brazilian Diabetes Society (SBD), the Brazilian Cardiology Society (SBC) and the Brazilian Endocrinology and Metabolism Society (SBEM). Diabetol Metab Syndr.

[300] Daskalopoulou SS, Rabi DM, Zarnke KB, Dasgupta K, Nerenberg K, Cloutier L (2015). The 2015 Canadian Hypertension Education Program recommendations for blood pressure measurement, diagnosis, and assessment of risk, prevention, and treatment of hypertension. Can J Cardiol.

[301] British Cardiac Society, British Hypertension Society, Diabetes UK, HEART UK, Primary Care Cardiovascular Society, Stroke Association (2005). JBS 2: Joint British Societies’ guidelines on prevention of cardiovascular disease in clinical practice. Heart.

[302] Sánchez RA, Boggia J, Peñaherrera E, Barroso WS, Barbosa E, Villar R (2020). Ambulatory blood pressure monitoring over 24 h: A Latin American Society of Hypertension position paper-accessibility, clinical use and cost effectiveness of ABPM in Latin America in year 2020. J Clin Hypertens.

[303] O’Brien E, Parati G, Stergiou G, Asmar R, Beilin L, European Society of Hypertension Working Group on (2013). Blood Pressure Monitoring. J Hypertens.

[304] Banegas JR, Ruilope LM, de la Sierra A, Vinyoles E, Gorostidi M (2018). Relationship between clinic and ambulatory blood-pressure measurements and mortality. N Engl J Med.

[305] Hansen TW, Li Y, Boggia J, Thijs L, Richart T, Staessen JA (2011). Predictive role of the nighttime blood pressure. Hypertension.

[306] Diao D, Wright JM, Cundiff DK, Gueyffier F (2012). Pharmacotherapy for mild hypertension. Cochrane Database Syst Rev.

[307] Thomopoulos C, Parati G, Zanchetti A (2014). Effects of blood pressure lowering on outcome incidence in hypertension. 1. Overview, meta-analyses, and meta-regression analyses of randomized trials. J Hypertens.

[308] Brunstrom M, Carlberg B (2018). Association of blood pressure lowering with mortal- ity and cardiovascular disease across blood pressure levels: a systematic review and meta-analysis. JAMA Intern Med.

[309] Lonn EM, Bosch J, López-Jaramillo P, Zhu J, Liu L, Pais P (2016). Blood-pressure lowering in intermediate-risk persons without cardiovascular disease. N Engl J Med.

[310] Lee CJ, Ryu J, Kim HC, Ryu DR, Ihm SH, Kim YJ (2018). Clinical benefit of treatment of stage-1, low-risk hypertension Korean national health insurance database analysis. Hypertension.

[311] Franklin SS, Larson MG, Khan SA, Wong ND, Leip EP, Kannel WB (2001). Does the relation of blood pressure to coronary heart disease risk change with aging?: The Framingham Heart Study. Circulation.

[312] Mahtta D, Elgendy IY, Pepine CJ (2018). Optimal medical treatment of hypertension in patients with coronary artery disease. Expert Rev Cardiovasc..

[313] Rosendorff C, Lackland DT, Allison M, Aronow WS, Black HR, Blumenthal RS (2015). Treatment of hypertension in patients with coronary artery disease: A scientific statement from the American Heart Association, American College of Cardiology, and American Society of Hypertension. J Am Coll Cardiol.

[314] Yannoutsos A, Dreyfuss CT, Safar ME, Blacher J (2017). Optimal blood pressure target in stroke prevention. Curr Opin Neurol.

[315] Béjot Y (2019). Targeting blood pressure for stroke prevention: current evidence and unanswered questions. J Neurol.

[316] Pinho-Gomes AC, Rahimi K (2019). Management of blood pressure in heart failure. Heart.

[317] Tsimploulis A, Lam PH, Arundel C, Singh SN, Morgan CJ, Faselis C (2018). Systolic Blood Pressure and Outcomes in Patients With Heart Failure With Preserved Ejection Fraction. JAMA Cardiol.

[318] Tsujimoto T, Kajio H (2018). Low diastolic blood pressure and adverse outcomes in heart failure with preserved ejection fraction. Int J Cardiol.

[319] Cheung AK, Rahman M, Reboussin DM, Craven TE, Greene T, Kimmel PL (2017). Effects of intensive BP control in CKD. J Am Soc Nephrol.

[320] Filipovsky J, Seidlerova J, Kratochvil Z, Karnosova P, Hronova M, Mayer O (2016). Automated compared to manual office blood pressure and to home blood pressure in hypertensive patients. Blood Press.

[321] Kjeldsen SE, Lund-Johansen P, Nilsson PM, Mancia G (2016). Unattended blood pressure measurements in the systolic blood pressure intervention trial: implications for entry and achieved blood pressure values compared with other trials. Hypertension.

[322] Heerspink HJ, Ninomiya T, Perkovic V, Woodward M, Zoungas S, Cass A (2010). Effects of a fixed combination of perindopril and indapamide in patients with type 2 diabetes and chronic kidney disease. Eur Heart J.

[323] Ku E, Gassman J, Appel LJ, Smogorzewski M, Sarnak MJ, Glidden DV (2017). BP control and long-term risk of ESRD and mortality. J Am Soc Nephrol.

[324] Chang AR, Appel LJ (2018). Target Blood Pressure for Cardiovascular Disease Prevention in Patients with CKD. Clin J Am Soc Nephrol.

[325] Chang AR, Lóser M, Malhotra R, Appel LJ (2019). Blood pressure goals in patients with CKD: A review of evidence and guidelines. Clin J Am Soc Nephrol.

[326] Associati American Diabetes (2020). on. Cardiovascular Disease and Risk Management: Standards of Medical Care in Diabetes-2020. Diabetes Care.

[327] Toklu B, Bangalore S (2015). Blood pressure lowering in patients with type 2 diabetes improves cardiovascular events including mortality, but more intensive lowering to systolic blood pressure less than 130 mm Hg is associated with further reduction in stroke and albuminuria without further reduction in cardiac events. Evid Based Med.

[328] Soliman EZ, Byington RP, Bigger JT, Evans G, Okin PM, Goff DC (2015). Effect of Intensive Blood Pressure Lowering on Left Ventricular Hypertrophy in Patients With Diabetes Mellitus: Action to Control Cardiovascular Risk in Diabetes Blood Pressure Trial. Hypertension.

[329] Brunström M, Carlberg B (2016). Effect of antihypertensive treatment at different blood pressure levels in patients with diabetes mellitus: Systematic review and meta-analyses. BMJ.

[330] Cushman WC, Evans GW, Byington RP, Goff DC, Grimm RH, Cutler JA (2010). Effects of intensive blood-pressure control in type 2 diabetes mellitus. N Engl J Med.

[331] Boer IH, Bangalore S, Benetos A, Davis AM, Michos ED, Muntner P (2017). Diabetes and hypertension: A position statement by the American diabetes association. Diabetes Care.

[332] Wright JT, Fine LJ, Lackland DT, Ogedegbe G, Dennison Himmelfarb CR (2014). Evidence supporting a systolic blood pressure goal of less than 150 mmHg in patients aged 60 years or older: the minority view. Ann Intern Med.

[333] Beckett N, Peters R, Leonetti G (2014). Subgroup and per-protocol analyses from the Hypertension in the Very Elderly Trial. J Hypertens.

[334] Weiss J, Freeman M, Low A, Fu R, Kerfoot A, Paynter R (2017). Benefits and harms of intensive blood pressure treatment in adults aged 60 years or older: A systematic review and meta-analysis. Ann Intern Med.

[335] Bavishi C, Bangalore S, Messerli FH (2017). Outcomes of Intensive Blood Pressure Lowering in Older Hypertensive Patients. J Am Coll Cardiol.

[336] Community Preventive Services Task F (2014). Team-based care to improve blood pressure control: recommendation of the Community Preventive Services Task Force. Am J Prev Med.

[337] Weinstein E, Rucker LM (2016). Team-based care to improve control of hypertension in an inner city practice. Healthc (Amst)..

[338] Mansoor SM, Krass I, Aslani P (2013). Multiprofessional interventions to improve patient adherence to cardiovascular medications. J Cardiovasc Pharmacol Ther.

[339] Carter BL, Rogers M, Daly J (2009). The potency of team-based care interventions for hypertension: a meta-analysis. Arch Intern Med.

[340] Walsh JM, McDonald KM, Shojania KG, Sundaram V, Nayak S, Lewis R (2006). Quality improvement strategies for hypertension management: a systematic review. Med Care.

[341] Potthoff SA, Vonend O (2017). Multidisciplinary Approach in the Treatment of Resistant Hypertension. Curr Hypertens Rep.

[342] David G, Gunnarsson C, Saynisch PA, Chawla R, Nigam S (2015). Do patient-centered medical homes reduce emergency department visits?. Health Serv Res.

[343] Jacob V, Chattopadhyay SK, Thota AB (2015). Economics of Team-based Care in Controlling Blood Pressure: A Community Guide Systematic Review. Am J Prev Med.

[344] Peacock E, Krousel-Wood M (2017). Adherence to Antihypertensive Therapy. Med Clin North Am.

[345] Jardim LM, Jardim TV, Souza WK, Barroso de Souza WKS, Pimenta CD, Sousa AL (2017). Multiprofessional Treatment of High Blood Pressure in Very Elderly Patients. Arq Bras Cardiol.

[346] Proia KK, Thota AB, Njie GJ, Finnie R, Hopkins D, Mukhtar Q (2014). Team-based care and improved blood pressure control: a community guide systematic review. Am J Prev Med.

[347] Kuhmmer R, Lazzaretti RK, Guterres CM, Raimundo FV, Leite LE, Delabary TS (2016). Effectiveness of multidisciplinary intervention on blood pressure control in primary health care: a randomized clinical trial. BMC Health Serv Res.

[348] Strumpf E, Ammi M, Diop M, Laniel JF, Tousignant P (2017). The impact of team-based primary care on health care services utilization and costs: Quebec’s family medicine groups. J Health Econ.

[349] Norouzi Z, Jafarnejad F, Khadivzadeh T, Esmaily H, Headjazi A (2019). Comparison of the effect of standardized patient-based training with team-based learning on the knowledge of midwifery students in providing services to victims of rape. J Educ Health Promot.

[350] Kravetz JD, Walsh RF (2016). Team-Based Hypertension Management to Improve Blood Pressure Control. J Prim Care Community Health.

[351] Boulware LE, Daumit GL, Frick KD, Minkovitz CS, Lawrence RS, Powe NR (2001). An evidence-based review of patient-centered behavioral interventions for hypertension. Am J Prev Med.

[352] Overwyk KJ, Dehmer SP, Roy K, Maciosek MV, Hong Y, Baker-Goering MM (2019). Modeling the Health and Budgetary Impacts of a Team-based Hypertension Care Intervention That Includes Pharmacists. Med Care.

[353] Tagliacozzo DM, Luskin DB, Lashof JC, Ima K (1974). Nurse intervention and patient behavior: an experimental study. Am J Public Health.

[354] Dickey FF, Mattar ME, Chudzik GM (1975). Pharmacist counsling increases drug regimen compliance. Hospitals.

[355] Brazil, Ministério da Saúde (2018). Política Nacional de Educação Permanente em Saúde: o que se tem produzido para o seu fortalecimento.

[356] Brazil, Ministério da Saúde (2018). Síntese de evidências para políticas de saúde: adesão ao tratamento medicamentoso por pacientes portadores de doenças crônicas.

[357] Marquez Contreras E, Marquez Rivero S, Rodriguez Garcia E, Ramos LLG, Vilas JCP, Suarez AB (2019). Specific hypertension smartphone application to improve medication adherence in hypertension: a cluster-randomized trial. Curr Med Res Opin.

[358] Hallberg I, Ranerup A, Bengtsson U (2018). Experiences, expectations and challenges of an interactive mobile phone-based system to support self-management of hypertension: patients’ and professionals’ perspectives. Patient Prefer Adherence.

[359] Hallberg I, Ranerup A, Bengstsson U, Kjellgren K (2016). Supporting the self-management of hypertension: Patients’ experiences of using a mobile phone-based system. J Hum Hypertens.

[360] Schoenthaler A, de la Calle F, Pitaro M, Lum A, Chaplin W, Mogavero J (2020). A Systems-Level Approach to Improving Medication Adherence in Hypertensive Latinos. J Gen Intern Med.

[361] Schoenthaler A, La Calle F, Barrios-Barrios M, Garcia A, Pitaro M, Lum A (2015). A practice-based randomized controlled trial to improve medication adherence among Latinos with hypertension: study protocol for a randomized controlled trial. Trials.

[362] Delavar F, Pashaeypoor S, Negarandeh R (2020). The effects of self-management education tailored to health literacy on medication adherence and blood pressure control among elderly people with primary hypertension: A randomized controlled trial. Patient Educ Couns.

[363] Serene Olin S, Kutash K, Pollock M (2014). Developing quality indicators for family support services in community team-based mental health care. Adm Policy Ment Health.

[364] Ranerup A, Hallberg I (2015). Actors and intentions in the development process of a mobile phone platform for self-management of hypertension. Inform Health Soc Care.

[365] Brazil, Ministério da Saúde (2017). Política Nacional de Atenção Básica. Portaria no 2.436, de 21 de setembro de 2017.

[366] Riegel GR, Ribeiro PAB, Rodrigues MP (2018). Efficacy of nutritional recommendations given by registered dietitians compared to other healthcare providers in reducing arterial blood pressure: Systematic review and meta-analysis. Clin Nutr.

[367] Mitchell LJ, Ball LE, Ross LJ (2017). Effectiveness of Dietetic Consultations in Primary Health Care: A Systematic Review of Randomized Controlled Trials. J Acad Nutr Diet.

[368] Sladdin I, Chaboyer W, Ball L (2018). Patients’ perceptions and experiences of patient-centred care in dietetic consultations. J Hum Nutr Diet.

[369] Riaz H, Khan MS, Siddiqi TJ, Usman MS, Shah N, Goyal A (2018). Association Between Obesity and Cardiovascular Outcomes: A Systematic Review and Meta-analysis of Mendelian Randomization Studies. JAMA Netw Open.

[370] Dwivedi AK, Dubey P, Cistola DP (2020). Association Between Obesity and Cardiovascular Outcomes: Updated Evidence from Meta-analysis Studies. Curr Cardiol Rep.

[371] Zhao CN, Meng X, Ya Li, Li S, Tang GY, Li HB (2017). Fruits for Prevention and Treatment of Cardiovascular Diseases. Nutrients.

[372] Mellendick K, Shanahan L, Wideman L, calkins S, Keane S, Lovelady C. (2018). Diets Rich in Fruits and Vegetables Are Associated with Lower Cardiovascular Disease Risk in Adolescents. Nutrients.

[373] Alissa EM, Ferns GA (2017). Dietary fruits and vegetables and cardiovascular diseases risk. Crit Rev Food Sci Nutr.

[374] Aminde LN, Cobiac LJ, Veerman JL (2019). Potential impact of a modest reduction in salt intake on blood pressure, cardiovascular disease burden and premature mortality: a modelling study. Open Heart.

[375] He FJ, Tan M, Ma Y (2020). Salt Reduction to Prevent Hypertension and Cardiovascular Disease: JACC State-of-the-Art Review. J Am Coll Cardiol.

[376] Graham GN, Ostrowski M, Sabina AB (2016). Population health-based approaches to utilizing digital technology: a strategy for equity. J Public Health Policy.

[377] Grahame JA (2016). Digital Note-Taking: Discussion of Evidence and Best Practices. J Physician Assist Educ.

[378] Zhao R, Bu W, Chen Y, Chen X (2020). The Dose-Response Associations of Sedentary Time with Chronic Diseases and the Risk for All-Cause Mortality Affected by Different Health Status: A Systematic Review and Meta-Analysis. J Nutr Health Aging.

[379] Ozemek C, Lavie CJ, Rognmo O (2019). Global physical activity levels - Need for intervention. Prog Cardiovasc Dis.

[380] Rezende LF, Rabacow FM, Viscondi JY, Luiz O, Matsudo V (2015). Effect of physical inactivity on major noncommunicable diseases and life expectancy in Brazil. J Phys Act Health.

[381] Ekelund U, Brown WJ, Steene-Johannessen J, Wang M, Owen N, Powell KE (2019). Do the associations of sedentary behaviour with cardiovascular disease mortality and cancer mortality differ by physical activity level? A systematic review and harmonised meta-analysis of data from 850 060 participants. Br J Sports Med.

[382] Ferreira T, Cipolotti M, Marques B, Miranda M (2016). A inserção do Profissional de Educação Física nos Núcleos de Apoio a Saúde da Família: visão dos profissionais. Rev Bras Ativ Fís Saúde.

[383] Balamurugan A, Adolph S, Faramawi M, George M (2017). Community Team-Based Care for Hypertension Management: A Public-Private Partnership in Rural Arkansas. J Ark Med Soc.

[384] Sidney S (2015). Team-Based Care: A Step in the Right Direction for Hypertension Control. Am J Prev Med.

[385] Santschi V, Wuerzner G, Chiolero A, Brurnand B (2017). Team-based care for improving hypertension management among outpatients (TBC-HTA): study protocol for a pragmatic randomized controlled trial. BMC Cardiovasc Disord.

[386] Al-Rubaey MG, Shwaish MI (2019). Impact of hypertension education on treatment compliance among hypertensive patients in Baghdad 2017. J Pak Med Assoc.

[387] Chen Y, Li X, Jing G, Pan B, Long B, Zhi Tong B (2020). Health education interventions for older adults with hypertension: A systematic review and meta-analysis. Public Health Nurs.

[388] Dzau VJ, Balatbat CA (2019). Future of Hypertension. Hypertension.

[389] Conn VS, Ruppar TM, Chase JA, Enriquez M, Cooper P (2015). Interventions to Improve Medication Adherence in Hypertensive Patients: Systematic Review and Meta-analysis. Curr Hypertens Rep.

[390] Conn VS, Ruppar TM, Chase JD (2016). Blood pressure outcomes of medication adherence interventions: systematic review and meta-analysis. J Behav Med.

[391] Mistry N, Keepanasseril A, Wilczynski NL, Nieuwlaat R, Ravall M, Haynes B (2015). Technology-mediated interventions for enhancing medication adherence. J Am Med Inform Assoc.

[392] Lindahl B, Norberg M, Johansson H, Lindvall K, Ng N, Nordin M (2020). Health literacy is independently and inversely associated with carotid artery plaques and cardiovascular risk. Eur J Prev Cardiol.

[393] Earl GL, Harris EM, Dave M, Jiang JE (2019). Implementing a health literacy module fostering patient-centered written communication in a cardiovascular prevention elective course. Curr Pharm Teach Learn.

[394] Fletcher BR, Hartmann-Boyce J, Hinton L, McManus RJ. 10 (2015). The Effect of Self-Monitoring of Blood Pressure on Medication Adherence and Lifestyle Factors: A Systematic Review and Meta-Analysis. Am J Hypertens.

[395] Fletcher BR, Hinton L, Hartmann-Boyce J (2016). Self-monitoring blood pressure in hypertension, patient and provider perspectives: A systematic review and thematic synthesis. Patient Educ Couns.

[396] Bengtsson U, Kjellgren K, Hallberg I, Lundin M, Makitalo A (2018). Patient contributions during primary care consultations for hypertension after self-reporting via a mobile phone self-management support system. Scand J Prim Health Care.

[397] McLean G, Band R, Saunderson K, Murray HP, Little P, McManus RJ (2016). Digital interventions to promote self-management in adults with hypertension systematic review and meta-analysis. J Hypertens.

[398] Thakkar J, Kurup R, Laba TL (2016). Mobile Telephone Text Messaging for Medication Adherence in Chronic Disease: A Meta-analysis. JAMA Intern Med.

[399] John JR, Tannous WK, Jones A (2020). Effectiveness of a patient-centered medical home model of primary care versus standard care on blood pressure outcomes among hypertensive patients. Hypertens Res.

[400] Yan R, Li W, Yin L, Wang Y, Bo J, PURE-China Investigators (2017). Cardiovascular Diseases and Risk-Factor Burden in Urban and Rural Communities in High-, Middle-, and Low-Income Regions of China: A Large Community-Based Epidemiological Study. J Am Heart Assoc.

[401] Malta DC, Silva AGD, Machado ÍE, Sá ACMGN, Santos FMD, Prates EJS, Cristo EB (2019). Trends in smoking prevalence in all Brazilian capitals between 2006 and 2017. J Bras Pneumol.

[402] Piper MA, Evans CV, Burda BU, Margolis KL, O’Connor E, Smith N (2014). Screening for High Blood Pressure in Adults: A Systematic Evidence Review for the U.S..

[403] Bhatnagar A, Maziak W, Eissenberg T, Ward KD, Thurston G, King BA (2019). Water Pipe (Hookah) Smoking and Cardiovascular Disease Risk: A Scientific Statement from the American Heart Association. Circulation.

[404] Leone FT, Zhang Y, Evers-Casey S, Evins AE, Eakin MN, Fathi J (2020). Initiating Pharmacologic Treatment in Tobacco-Dependent Adults. An Official American Thoracic Society Clinical Practice Guideline. Am J Respir Crit Care Med.

[405] Appel LJ, Moore TJ, Obarzanek E, Vollmer WM, Svetkey LP, Sacks FM (1997). A clinical trial of the effects of dietary patterns on blood pressure. N Engl J Med.

[406] Sacks FM, Svetkey LP, Vollmer WM, Appel LJ, Bray GA, Harsha D (2001). Effects on blood pressure of reduced dietary sodium and the Dietary Approaches to Stop Hypertension (DASH) diet. N Engl J Med.

[407] Filippou CD, Tsioufis CP, Thomopoulos CG, Mihas CC, Dimitriadis KS, Sotiropoulou LI (2020). Dietary Approaches to Stop Hypertension (DASH) diet and blood pressure reduction in adults with and without hypertension: A systematic review and meta-analysis of randomized controlled trials. Adv Nutr.

[408] Larsson SC, Wallin A, Wolk A (2016). Dietary Approaches to Stop Hypertension diet and incidence of stroke: results from 2 prospective cohorts. Stroke.

[409] Mertens E, Markey O, Geleijnse JM, Lovegrove JA, Gibens DI (2018). Adherence to a healthy diet in relation to cardiovascular incidence and risk markers: evidence from the Caerphilly Prospective Study. Eur J Nutr.

[410] Soltani S, Arablou T, Jayedi A, Salehi-Abargouei A (2020). Adherence to the dietary approaches to stop hypertension (DASH) diet in relation to all-cause and cause-specific mortality: a systematic review and dose-response meta-analysis of prospective cohort studies. Nutr J.

[411] Mozaffari H, Ajabshir S, Alizadeh S (2020). Dietary Approaches to Stop Hypertension and risk of chronic kidney disease: A systematic review and meta-analysis of observational studies. Clin Nutr.

[412] Martínez-González MA, Gea A, Ruiz-Canela M (2019). The mediterranean diet and cardiovascular health. Circ Res.

[413] Dinu M, Pagliai G, Casini A, Sofi F (2018). Mediterranean diet and multiple health outcomes: an umbrella review of meta-analyses of observational studies and randomised trials. Eur J Clin Nutr.

[414] Rosato V, Temple NJ, La Vecchia C, Castellan G, Tavani A, Guercio V (2019). Mediterranean diet and cardiovascular disease: a systematic review and meta-analysis of observational studies. Eur J Nutr.

[415] Nissensohn M, Román-Viñas B, Sánchez-Villegas A, Piscopo S, Serra-Majem L (2016). The effect of the mediterranean diet on hypertension: a systematic review and meta-analysis. J Nutr Educ Behav.

[416] Jennings A, Berendsen AM, de Groot LCPGM, Feskens EJM, Brzozowska A, Sicinska E (2019). Mediterranean-style diet improves systolic blood pressure and arterial stiffness in older adults. Hypertension.

[417] Domenech M, Roman P, Lapetra J, Garcia de la Corte FJ, Sala-Vila A, de la Torre R (2014). Mediterranean diet reduces 24-hour ambulatory blood pressure, blood glucose, and lipids: one-year randomized, clinical trial. Hypertension.

[418] Fuchs SF (2011). Estudo PREVER: Mudanças de Estilo de Vida.

[419] Powles J, Gahimi S, Micha R, Khatibzadeh S, Shi P, Ezzati M (2013). Global, regional and national sodium intake in 1990 and 2010: a systematic analysis of 24h urinary sodium excretion and dietary surveys worldwide. BMJ Open.

[420] World Health Organization, WHO (2012). Guideline: Sodium intake for adults and children.

[421] Huang L, Trieu K, Yoshimura S, Neal B, Woodward M, Campbell NRC (2020). Effect of dose and duration of reduction in dietary sodium on blood pressure levels: systematic review and meta-analysis of randomised trial. BMJ.

[422] He FJ, Li J, Macgregor GA (2013). Effect of longer term modest salt reduction on bloodpressure: Cochrane systematic review and meta-analysis of randomised trials. BMJ.

[423] Bernabe-Ortiz A, Sal Y Rosas VG, Ponce-Lucero V, Cárdenas MK, Carrillo-Larco RM, Diez-Canseco F (2020). Effect of salt substitution on community-wide blood pressure and hypertension incidence. Nat Med.

[424] Marklund M, Singh G, Greer R, Cudhea F, Matsushita K, Micha R, Brady T (2020). Estimated population wide benefits and risks in China of lowering sodium through potassium enriched salt substitution: modelling study. BMJ.

[425] Binia A, Jaeger J, Hu Y, Singh A, Zimmermann D (2015). Daily potassium intake and sodium-to-potassium ratio in the reduction of blood pressure: a meta-analysis of randomized controlled trials. J Hypertens.

[426] China Salt Substitute Study Collaborative Group (2007). Salt substitution: a low-cost strategy for blood pressure control among rural Chinese. A randomized, controlled trial. J Hypertens.

[427] Zhou B, Wang HL, Wang WL, Wu XM, Fu LY, Shi JP (2013). Long-term effects of salt substitution on blood pressure in a rural north Chinese population. J Hum Hypertens.

[428] Peng Y-G, Li W, Wen X-X, Li Y, Hu J-H, Zhao L-C (2014). Effects of salt substitutes on blood pressure: a meta-analysis of randomized controlled trials. Am J Clin Nutr.

[429] Jin A, Xie W, Wu Y (2020). Effect of salt reduction interventions in lowering blood pressure in Chinese populations: a systematic review and meta-analysis of randomised controlled trials. BMJ Open.

[430] Thorning TK, Bertram HC, Bonjour JP, Groot L, Dupont D, Feeney R (2017). Whole dairy matrix or single nutrients in assessment of health effects: current evidence and knowledge gaps. Am J Clin Nutr.

[431] Mozaffarian D, Wu JHY (2018). Flavonoids, dairy foods, and cardiovascular and metabolic health: a review of emerging biologic pathways. Circ Res.

[432] Dehghan M, Mente A, Rangarajan S, Sheridan P, Mohan V, Igbal R (2018). Association of dairy intake with cardiovascular disease and mortality in 21 countries from five continents (PURE): a prospective cohort study. Lancet.

[433] Buendia JR, Li Y, Hu FB, Cabral HJ, Bradlee ML, Quatromoni PA (2018). Regular yougurt intake and risk of cardiovascular disease among hypertensive adults. Am J Hypertens.

[434] Machin DR, Park W, Alkatan M, Mouton M, Tanaka H (2014). Hypotensive effects of solitary addition of conventional nonfat dairy products to the routine diet: a randomized controlled trial. Am J Clin Nutr.

[435] Rietsema S, Eelderink C, Joustra ML, van Vliet IMY, van Londen M, Corpeleijn E (2019). Effect of high compared with low dairy intake on blood pressure in overweight middle-aged adults: results of a randomized crossover intervention study. Am J Clin Nutr.

[436] Hidayat K, Du HZ, Yang J, Chen GC, Zhang Z, Lin ZN, Qin LQ (2017). Effects of milk proteins on blood pressure: a meta-analysis of randomized control trials. Hypertens Res.

[437] Brazil (2014). Ministério da Saúde.

[438] Dietary Guidelines Advisory Committee (2015). Scientific Report of the 2015 Dietary Guidelines Advisory Committee: Advisory Report to the Secretary of Health and Human Services and the Secretary of Agriculture.

[439] Desch S, Schmidt J, Kobler D, Sonnabend M, Eitel I, Sareban M (2010). Effect of cocoa products on blood pressure: systematic review and meta-analysis. Am J Hypertens.

[440] (2017). Effect of cocoa on blood pressure. Cochrane Database Syst Rev.

[441] Mesas AE, Leon-Munoz LM, Rodriguez-Artalejo F, Lopez-Garcia E (2011). The effect of coffee on blood pressure and cardiovascular disease in hypertensive individuals: a systematic review and meta-analysis. Am J Clin Nutr.

[442] van Dam RM, Hu FB, Willett WC (2020). Coffee, caffeine, and health. N Engl J Med.

[443] D´Elia L, La Fata E, Galletti F, Scalfi L, Strazzullo P (2019). Coffee consumption and risk of hypertension: a dose-response meta-analysis of prospective studies. Eur J Nutr.

[444] Grosso G, Micek A, Godos J, Pajak A, Sciacca S, Bes-Rastrollo M, Galvano F, Martinez-Gonzalez MA (2017). Long-Term Coffee Consumption Is Associated with Decreased Incidence of New-Onset Hypertension: A Dose-Response Meta-Analysis. Nutrients.

[445] Ke L, Mason RS, Mpofu E, Vingren JL, Li Y, Graubard BI (2017). Hypertension and other cardiovascular risk factors are associated with vitamin D deficiency in an urban Chinese population: a short report. J Steroid Biochem Mol Biol.

[446] Zhang D, Cheng C, Wang Y, Sun H, Yu S, Xue Y (2020). Effect of vitamin D on blood pressure and hypertension in the general population: an update meta-analysis of cohort studies and randomized controlled trials. Prev Chronic Dis.

[447] Pilz S, Gaksch M, Kienreich K, Grubler M, Verheyen N, Fahrleitner-Pammer A (2015). Effects of vitamin D on blood pressure and cardiovascular risk factors: a randomized controlled trial. Hypertension.

[448] Arora P, Song Y, Dusek J, Plotnikoff G, Sabatine MS, Cheng S (2015). Vitamin D therapy in individuals with prehypertension or hypertension: the DAYLIGHT trial. Circulation.

[449] Shu L, Huang K (2018). Effect of vitamin D supplementation on blood pressure parameters in patients with vitamin D deficiency: a systematic review and meta-analysis. J Am Soc Hypertens.

[450] Cormick G, Ciapponi A, Cafferata ML, Belizán JM (2015). Calcium Supplementation for Prevention of Primary Hypertension. Cochrane Database Syst Rev.

[451] Li K, Liu C, Kuang X, Deng Q, Zhao F, Li D (2018). Effects of Multivitamin and Multimineral Supplementation on Blood Pressure: A Meta-Analysis of 12 Randomized Controlled Trials. Nutrients.

[452] Hall JE, do Carmo JM, Silva AA, Wang Z, Hall ME (2019). Obesity, kidney dysfunction and hypertension: mechanistic links. Nat Rev Nephrol.

[453] Neter JE, Stam BE, Kok FJ, Grobbee DE, Geleijnse JM (2003). Influence of weight reduction on blood pressure: a meta-analysis of randomized controlled trials. Hypertension.

[454] Di Angelantonio E, ShN Bhupathiraju, Wormser D, Gao P, Kaptoge S, Berrington de Gonzalez A, Global BMI Mortality Collaboration (2016). Body-mass index and all-cause mortality: individual-participant-data meta-analysis of 239 prospective studies in four continents. Lancet.

[455] Caspersen CJ, Powell KE, Christenson GM (1985). Physical activity, exercise, and physical fitness: definitions and distinctions for health-related research. Public Health Rep.

[456] US Department of Health and Human Services Physical Activity Guidelines for Americans 2018.

[457] Ekelund U, Steene-Johannessen J, Brown WJ (2016). Does physical activity attenuate, or even eliminate, the detrimental association of sitting time with mortality? A harmonized meta-analysis of data from more than 1 million men and women. Lancet.

[458] Liu X, Zhang D, Liu Y, Sun X, Han C, Wang B (2017). Dose-response associations between physical activity and incident hypertension: a systematic review and meta-analysis of cohort studies. Hypertension.

[459] Cao L, Li X, Yan P, Wang X, Li M, Li R (2019). The effectiveness of aerobic exercise for hypertensive population: A systematic review and meta-analysis. J Clin Hypertens.

[460] MacDonald HV, Johnson BT, Huedo-Medina TB, Livingston J, Forsyth KC, Kraemer WJ (2016). Dynamic Resistance Training as Stand- Alone Antihypertensive Lifestyle Therapy: A Meta-Analysis. J Am Heart Assoc.

[461] Jin YZ, Yan S, Yuan WX (2017). Effect of isometric handgrip on resting blood pressure in adults: a meta-analysis of randomized controlled trials. J Sports Med Phys Fitness.

[462] Igarashi Y, Nogami Y (2018). The effects of regular aquatic exercise on blood pressure: a meta-analysis of randomized controlled trials. Eur J Prev Cardiol.

[463] Cramer H, Langhorst J, Dobos G, Lauche R (2016). Yoga for metabolic syndrome: a systematic review and meta-analysis. Eur J Prev Cardiol.

[464] Zhong D, Li J, Yang H, Li Y, Huang Y, Xiao Q (2020). Tai Chi for Essential Hypertension: a Systematic Review of Randomized Controlled Trials. Curr Hypertens Rep.

[465] Costa EC, Hay JL, Kehler DS, Boreskie KF, Arora RC, Umpierre D (2018). Effects of High-Intensity Interval Training Versus Moderate- Intensity Continuous Training on Blood Pressure in Adults with Pre- to Established Hypertension: a Systematic Review and Meta-Analysis of Randomized Trials. Sports Med.

[466] Riebe D, Franklin BA, Thompson PD, Garber CE, Whitfield GP, Magal M, Pescatello LS (2015). Updating ACSM’s Recommendations for Exercise Preparticipation Health Screening. Med Sci Sports Exerc.

[467] Meneguelo RS (2010). III Diretrizes da Sociedade Brazileira de Cardiologia sobre Teste Ergométrico. Arq Bras Cardiol.

[468] Mahtani KR, Nunan D, Heneghan CJ (2012). Device-guided breathing exercises in the control of human blood pressure: systematic review and meta-analysis. J Hypertens.

[469] Zou Y, Zhao X, Hou YY, Liu T, Wu Q, Huang YH (2017). Meta-Analysis of Effects of Voluntary Slow Breathing Exercises for Control of Heart Rate and Blood Pressure in Patients with Cardiovascular Diseases. Am J Cardiol.

[470] Ubolsakka-Jones C, Tongdee P, Jones DA (2019). The effects of slow loaded breathing training on exercise blood pressure in isolated systolic hypertension. Physiother Res Int.

[471] Kow FP, Adlina B, Sivasangari S, Punithavathi N, Ng KK, Ang AH (2018). The impact of music guided deep breathing exercise on blood pressure control - A participant blinded randomised controlled study. Med J Malaysia.

[472] Brook RD, Appel LJ, Rubenfire M, Ogedegbe G, Bisognano JD, Elliott WJ (2013). Beyond medications and diet: alternative approaches to lowering blood pressure: a scientific statement from the American Heart Association. Hypertension.

[473] Bradt J, Dileo C, Potvin N (2013). Music for stress and anxiety reduction in coronary heart disease patients. Cochrane Database of Systematic Reviews.

[474] Do Amaral MAS, Neto MG, Queiroz JG, Martins PRS, Saquetto M B, Carvalho VO (2016). Effect of music therapy on blood pressure of individuals with hypertension: A systematic review and Meta-analysis. Int J Cardiol.

[475] Kühlmann AYR, Etnel JRG, Roos-Hesselink JW, Jeekel J, Bogers AJJC, Takkenberg JJM (2016). Systematic review and meta-analysis of music interventions in hypertension treatment: a quest for answers. BMC Cardiovasc Disord.

[476] Maynard BR, Solis MR, Miller VL, Brendel KE (2017). Mindfulness-based interventions for improving cognition, academic achievement, behavior, and socioemotional functioning of primary and secondary school students. Campbell Systematic Reviews.

[477] Goyal M, Singh S, Sibinga EMS, Gould NF, Rowland-Seymour A, Sharma R (2014). Meditation Programs for Psychological Stress and Well-being A Systematic Review and Meta-analysis. JAMA Intern Med.

[478] Ooi SL, Giovino M, Pak SC (2017). Transcendental meditation for lowering blood pressure: An overview of systematic reviews and meta-analyses. Complement Ther Med.

[479] GN, Lange RA, Bairey-Merz CN, Davidson RJ, Jamerson K, Mehta PK, American Heart Association Council on Clinical Cardiology, Council on Cardiovascular and Stroke Nursing, Council on Hypertension (2017). Meditation and Cardiovascular Risk Reduction: A Scientific Statement From the American Heart Association. J Am Heart Assoc.

[480] Steinhauser KE, Fitchett G, Handzo GF, Johnson KS, Koenig HG, Pargament KI (2017). State of the Science of Spirituality and Palliative Care Research Part I: Definitions, Measurement, and Outcomes. J Pain Symptom Manag.

[481] Lucchese FA, Koenig HG (2013). Religion, spirituality and cardiovascular disease: research, clinical implications, and opportunities in Brazil. Rev Bras Cir Cardiovasc.

[482] Chida Y, Steptoe A, Powell LH (2009). Religiosity/Spirituality and Mortality. Psychother Psychosom.

[483] Li S, Stampfer MJ, Williams DR, VanderWeele TJ (2016). Association of Religious Service Attendance With Mortality Among Women. JAMA Intern Med.

[484] VanderWeele TJ, Yu J, Cozier YC, Wise L, Argentieri MA, Rosenberg L (2017). Attendance at Religious Services, Prayer, Religious Coping, and Religious/Spiritual Identity as Predictors of All-Cause Mortality in the Black Women’s Health Study. Am J Epidemiol.

[485] Abu HO, Ulbricht C, Ding E, Allison JJ, Salmoirago-Blotcher E, Goldberg RJ (2018). Association of religiosity and spirituality with quality of life in patients with cardiovascular disease: a systematic review. Qual Life Res.

[486] Shattuck EC, Muehlenbein MP (2020). Religiosity/Spirituality and Physiological Markers of Health. J Relig Health.

[487] Spence ND, Farvid MS, Warner ET (2020). Religious Service Attendance, Religious Coping, and Risk of Hypertension in Women Participating in the Nurses’ Health Study II. Am J Epidemiol.

[488] Holt-Lunstad J, Steffen PR, Sandberg J, Jensen B (2011). Understanding the connection between spiritual well-being and physical health: an examination of ambulatory blood pressure, inflammation, blood lipids and fasting glucose. J Behav Med.

[489] Fitchett G, Powell LH (2009). Daily spiritual experiences, systolic blood pressure, and hypertension among midlife women in SWAN. Ann Behav Med.

[490] Buck AC, Williams DR, Musick MA, Sternthal MJ (2009). An examination of the relationship between multiple dimensions of religiosity, blood pressure, and hypertension. Soc Sci Med.

[491] Suh H, Hill TD, Koenig HG (2019). Religious Attendance and Biological Risk: A National Longitudinal Study of Older Adults. J Relig Health.

[492] Badanta-Romero B, Diego-Cordero R, Rivilla-García E (2018). Influence of Religious and Spiritual Elements on Adherence to Pharmacological Treatment. J Relig Health.

[493] Oliveira JA, Anderson MI, Lucchetti G, Pires EV, Gonçalves LM (2019). Approaching Spirituality Using the Patient-Centered Clinical Method. J Relig Health.

[494] Balboni TA, Fitchett G, Handzo GF (2017). State of the Science of Spirituality and Palliative Care Research Part II: Screening, Assessment, and Interventions. J Pain Symptom Manage.

[495] Hypertension Task Force of the Latin American Society of (2017). Guidelines on the management of arterial hypertension and related comorbidities in Latin America. Journal of Hypertension.

[496] Collaboration Blood Pressure Lowering Treatment Trialists’ (2014). Blood pressure-lowering treatment based on cardiovascular risk: a meta-analysis of individual patient data. Lancet.

[497] Turnbull F, Collaboration Blood Pressure Lowering Treatment Trialists’ (2003). Effects of different blood-pressure-lowering regimens on major cardiovascular events: results of prospectively-designed overviews of randomised trials. Lancet.

[498] Sytkowski PA, D’Agostino RB, Belanger AJ, Kannel WB (1996). Secular trends in long-term sustained hypertension, long-term treatment, and cardiovascular mortality. Circulation.

[499] Bang CN, Devereux RB, Okin PM (2014). Regression of electrocardiographic left ventricular hypertrophy or strain is associated with lower incidence of cardiovascular morbidity and mortality in hypertensive patients independent of blood pressure reduction - A LIFE review. J Electrocardiol.

[500] Fagard RH, Celis H, Thijs L, Wouters S (2009). Regression of left ventricular mass by antihypertensive treatment: a meta-analysis of randomized comparative studies. Hypertension.

[501] Ibsen H, Olsen MH, Wachtell K, Borch-Johnsen K, Lindholm LH, Mogensen CE (2005). Reduction in albuminuria translates to reduction in cardiovascular events in hypertensive patients: losartan intervention for endpoint reduction in hypertension study. Hypertension.

[502] Mancia G, Rea F, Corrao G, Grassi G (2019). Two-Drug Combinations as First-Step Antihypertensive Treatment. Circ Res.

[503] Póvoa R, Barroso WS, Brandão AA, Jardim PC, Barroso O, Passarelli O (2014). I Brazilian position paper on antihypertensive drug combination. Arq Bras Cardiol.

[504] Yugar-Toledo JC, Moreno H, Gus M, Rosito GBA, Scala LCN, Muxfeldt ES (2020). Brazilian Position Statement on Resistant Hypertension - 2020. Arq Bras Cardiol.

[505] Gradman AH, Basile JN, Carter BL, Bakris GL, American Society of Hypertension Writing Group (2010). Combination therapy in hypertension. J Am Soc Hypertens.

[506] Wald DS, Law M, Morris JK, Bestwick JP, Wald NJ (2009). Combination therapy versus monotherapy in reducing blood pressure: metaanalysis on 11,000 participants from 42 trials. Am J Med.

[507] Law MR, Wald NJ, Morris JK, Jordan RE (2003). Value of low dose combination treatment with blood pressure lowering drugs: analysis of 354 randomised trials. BMJ.

[508] Psaty BM, Smith NL, Siscovick DS, Koepsell TD, Weiss NS, Heckbert SR (1997). Health outcomes associated with antihypertensive therapies used as first-line agents: a systematic review and meta-analysis. JAMA.

[509] SHEP-Cooperative Research Group (1991). Prevention of stroke by antihypertensive drug treatment in older persons with isolated systolic hypertension: final results of the Systolic Hypertension in the Elderly Program (SHEP). JAMA.

[510] ALLHAT Officers and Coordinators for the ALLHAT Collaborative Research Group, The Antihypertensive and Lipid-Lowering Treatment to Prevent Heart Attack Trial (2002). Major outcome in high-risk hypertensive patients to angiotensin-converting enzyme inhibitor or calcium channel blocker vs. diuretic: the Antihypertensive and Lipid-Lowering Treatment to Prevent Heart Attack Trial (ALLHAT). JAMA.

[511] Dhalla IA, Gomes T, Yao Z, Nagge J, Persaud N, Hellings C (2013). Chlorthalidone versus hydrochlorothiazide for the treatment of hypertension in older adults: a population-based cohort study. Ann Intern Med.

[512] Hripcsak G, Suchard MA, Shea S, Chen R, You SC, Pratt N (2020). Comparison of Cardiovascular and Safety Outcomes of Chlorthalidone vs Hydrochlorothiazide to Treat Hypertension. JAMA Intern Med.

[513] Roush GC, Ernst ME, Kostis JB, Tandon S, Sica DA (2015). Head-to-Head Comparisons of Hydrochlorothiazide With Indapamide and Chlorthalidone. Hypertension.

[514] Elliott WJ, Ram CV (2011). Calcium channel blockers. J Clin Hypertens.

[515] Messerli FH (2002). Calcium antagonists in hypertension: from hemodynamics to outcomes. Am J Hypertens.

[516] Elliott WJ, Bandari A (2005). The role of calcium antagonists in stroke prevention. J Clin Hypertens.

[517] Nathan S, Pepine CJ, Bakris GL (2005). Calcium antagonists: effects on cardiorenal risk in hypertensive patients. Hypertension.

[518] Rollins G (2004). Calcium antagonist and beta blocker regimens found equally effective in hypertensive patients with coronary artery disease. Rep Med Guidel Outcomes Res.

[519] Vejakama P, Thakkinstian A, Lertrattananon D, Ingsathit A, Ngarmukos C, Attia J (2012). Reno-protective effects of renin-angiotensin system blockade in type 2 diabetic patients: a systematic review and network meta-analysis. Diabetologia.

[520] Baram M, Kommuri A, Sellers SA, Cohn JR (2013). ACE inhibitor-induced angioedema. J Allergy Clin Immunol Pract.

[521] Ryan MJ, Tuttle KR (2008). Elevations in serum creatinine with RAAS blockade: why isn’t it a sign of kidney injury?. Curr Opin Nephrol Hypertens.

[522] Maschio G, Alberti D, Janin G, Locatelli F, Mann JF, Motolese M, The Angiotensin-Converting-Enzyme Inhibition in Progressive Renal Insufficiency Study Group (1996). Effect of the angiotensin-converting-enzyme inhibitor benazepril on the progression of chronic renal insufficiency. N Engl J Med.

[523] Polifka JE (2012). Is there an embryopathy associated with first-trimester exposure to angiotensin-converting enzyme inhibitors and angiotensin receptor antagonists? A critical review of the evidence. Birth Defects Res A Clin Mol Teratol.

[524] Laube GF, Kemper MJ, Schubiger G, Neuhaus TJ (2007). Angiotensin-converting enzyme inhibitor fetopathy: long-term outcome. Arch Dis Child Fetal Neonatal Ed.

[525] Dahlof B, Devereux R, Kjeldsen S, Julius S, Beevers G, Faire U (2002). Cardiovascular morbidity and mortality in the losartan intervention or endpoint reduction in hypertension study (LIFE): a randomized trial against atenolol. Lancet.

[526] Lindholm LH, Ibsen H, Dahlof B, Devereux RB, Beevers G, Faire U (2002). Cardiovascular morbidity and mortality in patients with diabetes in the Losartan Intervention For Endpoint reduction in hypertension study (LIFE): a randomized trial against atenolol. Lancet.

[527] Julius S, Kejdelsen SE, Weber M, Brunner HR, Ekman S, Hansson L (2004). Outcomes in hypertensive patients in high cardiovascular risk treated with regimens based on valsartan and amlodipine: the VALUE randomised trial. Lancet.

[528] Pfeffer MA, Swedberg K, Granger CB, Held P, McMurray JJ, Michelson EL (2003). Effects of candesartan on mortality and morbidity in patients with chronic heart failure: the CHARM Overall programme. Lancet.

[529] Brenner BM, Cooper ME, Zeeuw D, Keane WF, Mitch WE, Parving HH (2001). Effects of losartan on renal and cardiovascular outcomes in patients with type 2 diabetes and nephropathy. N Engl J Med.

[530] Lewis EJ, Hunsicker LG, Clarke WR, Berl T, Pohl MA, Lewis JB (2001). Renoprotective effect of the angiotensin receptor antagonist irbersartan in patients with nephropathy due to type 2 diabetes. N Eng J Med.

[531] Parving HH, Lehnert H, Brochner-Mortensen J, Gomis R, Andersen S, Arner P, Irbesartan in Patients with Type 2 Diabetes and Microalbuminuria Study Group (2001). The effect of irbersartan on the development of diabetic nephropathy in patients with type 2 diabetes. N Engl J Med.

[532] Helfand M, Peterson K, Christensen V, Dana T, Thakurta S (2009). Drug class review: Beta adrenergic blockers. Update 4.

[533] López-Sendón J, Swedberg K, McMurray J, Tamargo J, Maggioni AP, Dargie H (2004). Expert consensus document on beta-adrenergic receptor blockers. Eur Heart J.

[534] Dulin B, Abraham WT (2004). Pharmacology of carvedilol. Am J Cardiol.

[535] Pedersen ME, Cockcroft JR (2007). The vasodilatory beta-blockers. Curr Hypertens Rep.

[536] Lindholm LH, Carlberg B, Samuelsson O (2005). Should beta blockers remain first choice in the treatment of primary hypertension? A meta-analysis. Lancet.

[537] Vongpatanasin W, Kario K, Atlas SA, Victor RG (2011). Central sympatholitic drugs. J Clin Hypertens.

[538] Atlas D, Diamant S, Zonnenschein R (1992). Is the imidazoline site a unique receptor? A correlation with clonidine-displacing substance activity. Am J Hypertens.

[539] Kaplan NM, Victor RG (2015). Clinical hypertension.

[540] Wagner ML, Walters AS, Coleman RG, Hening WA, Grasing K, Chokroverty S (1996). Randomized double-blind, placebo-controlled study of clonidine in restless legs syndrome. Sleep.

[541] Bond WS (1986). Psychiatric indications for clonidine. J Clin Psychopharmacol.

[542] Pandya KJ, Raubertas RF, Flynn PJ, Hynes HE, Rosenbluth RJ, Kirshner JJ (2000). Oral clonidine in postmenopausal patients with breast cancer experiencing tamoxifen-induced hot flashes: a University of Rochester Cancer Center Community Clinical Oncology Program study. Ann Intern Med.

[543] Fedorak RN, Field M, Chang EB (1985). Treatment of diabetic diarrhea with clonidine. Ann Intern Med.

[544] Esler M, Dudley F, Jennings G, Debinski H, Lambert G, Jones P (1992). Increased sympathetic nervous activity and the effects of its inhibition with clonidine in alcoholic cirrohosis. Ann Intern Med.

[545] Müller DN, Derer W, Dechend R (2008). Aliskiren-mode of action and preclinical data. J Mol Med.

[546] Danser AH (2009). (Pro)renin receptors: are they biologically relevant?. Curr Opin Nephrol Hypertens.

[547] Singh VP, Le B, Khode R, Baker KM, Kumar R (2008). Intracellular angiotensin II production in diabetic rats is correlated with cardiomyocyte apoptosis, oxidative stress, and cardiac fibrosis. Diabetes.

[548] Musini VM, Fortin PM, Bassett K, Wright JM (2008). Blood pressure lowering efficacy of renin inhibitors for primary hypertension. Cochrane Database Syst Rev.

[549] Heerspink HJL, Persson F, Brenner BM, Chaturvedi N, Brunel P, McMurray JJ (2016). Renal outcomes with aliskiren in patients with type 2 diabetes: a prespecified secondary analysis of the ALTITUDE randomised controlled trial. Lancet Diabetes Endocrinol.

[550] Bjerre HL, Christensen JB, Buus NH, Simonsen U, Su J (2019). The role of aliskiren in the management of hypertension and major cardiovascular outcomes: a systematic review and meta-analysis. J Hum Hypertens.

[551] Zhang JT, Chen KP, Guan T, Zhang S (2015). Effect of aliskiren on cardiovascular outcomes in patients with prehypertension: a meta-analysis of randomized controlled trials. Drug Des Devel Ther.

[552] Gradman AH, Parisé H, Lefebvre P, Falvey H, Lafeuille MH, Duh MS (2013). Initial combination therapy reduces the risk of cardiovascular events in hypertensive patients: a matched cohort study. Hypertension.

[553] Bangalore S, Kamalakkannan G, Parkar S, Messerli FH (2007). Fixed-Dose Combinations Improve Medication Compliance: A Meta-Analysis. Am J Med.

[554] Jamerson K, Weber MA, Bakris GL, Dahlöf B, Pitt B, Shi V (2008). Benazepril plus Amlodipine or Hydrochlorothiazide for Hypertension in High-Risk Patients. N Engl J Med.

[555] Jamerson KA, Bakris GL, Weber MA (2010). 24-Hour Ambulatory Blood Pressure in the ACCOMPLISH Trial. N Engl J Med.

[556] Weber MA, Jamerson K, Bakris GL, Weir MR, Zappe D, Zhang Y (2013). Effects of body size and hypertension treatments on cardiovascular event rates: subanalysis of the ACCOMPLISH randomised controlled trial. Lancet.

[557] Bakris GL, Sarafidis PA, Weir MR, Dahlöf B, Pitt B, Jamerson K (2010). Renal outcomes with different fixed-dose combination therapies in patients with hypertension at high risk for cardiovascular events (ACCOMPLISH): a prespecified secondary analysis of a randomised controlled trial. Lancet.

[558] Dahlöf B, Sever PS, Poulter NR, Wedel H, Beevers DG, Caulfield M (2005). Prevention of cardiovascular events with an antihypertensive regimen of amlodipine adding perindopril as required versus atenolol adding bendroflumethiazide as required, in the Anglo-Scandinavian Cardiac Outcomes Trial-Blood Pressure Lowering Arm (ASCOT-BPLA): a multicentre randomised controlled trial. Lancet.

[559] Patel A, MacMahon S, Chalmers J, Neal B, Woodward M, ADVANCE Collaborative Group (2007). Effects of a fixed combination of perindopril and indapamide on macrovascular and microvascular outcomes in patients with type 2 diabetes mellitus (the ADVANCE trial): a randomised controlled trial. Lancet.

[560] Beckett NS, Peters R, Fletcher AE, Staessen JA, Liu L, Dumitrascu D (2008). Treatment of Hypertension in Patients 80 Years of Age or Older. N Engl J Med.

[561] Dahlöf B, Hansson L, Lindholm LH, Scherstén B, Ekbom T, Wester P-O (1991). Morbidity and mortality in the Swedish Trial in Old Patients with Hypertension (STOP-Hypertension). Lancet.

[562] Hansson L, Lindholm LH, Ekbom T, Dahlöf B, Lanke J, Scherstén B (1999). Randomised trial of old and new antihypertensive drugs in elderly patients: cardiovascular mortality and morbidity the Swedish Trial in Old Patients with Hypertension-2 study. Lancet.

[563] Calhoun DA, Lacourcière Y, Chiang YT, Glazer RD (2009). Triple antihypertensive therapy with amlodipine, valsartan, and hydrochlorothiazide: a randomized clinical trial. Hypertension.

[564] Krieger EM, Drager LF, Giorgi DMA, Pereira AC, Barreto JAS, Nogueira AR (2018). Spironolactone Versus Clonidine as a Fourth-Drug Therapy for Resistant Hypertension: The ReHOT Randomized Study (Resistant Hypertension Optimal Treatment). Hypertension.

[565] Dahal K, Kunwar S, Rijal J, Alqatahni F, Panta R, Ishak N (2015). The Effects of Aldosterone Antagonists in Patients With Resistant Hypertension: A Meta-Analysis of Randomized and Nonrandomized Studies. Am J Hypertens.

[566] Liu G, Zheng XX, Xu YL, Lu J, Hui RT, Huang XH (2015). Effect of aldosterone antagonists on blood pressure in patients with resistant hypertension: a meta-analysis. J Hum Hypertens.

[567] Williams B, MacDonald TM, Morant S, Webb DJ, Sever P, McInnes G (2015). Spironolactone versus placebo, bisoprolol, and doxazosin to determine the optimal treatment for drug-resistant hypertension (PATHWAY-2): a randomised, double-blind, crossover trial. Lancet.

[568] Mann JF, Schmieder RE, McQueen M, Dyal L, Schumacher H, Pogue J (2008). Renal outcomes with telmisartan, ramipril, or both, in people at high vascular risk (the ONTARGET study): a multicentre, randomised, double-blind, controlled trial. Lancet.

[569] Parving H-H, Brenner BM, McMurray JJV, Zeeuw D, Haffner SM, Solomon SD (2012). Cardiorenal End Points in a Trial of Aliskiren for Type 2 Diabetes. N Engl J Med.

[570] Ogihara T, Saruta T, Rakugi H, Saito I, Shimamoto K, Matsuoka H (2014). Combinations of olmesartan and a calcium channel blocker or a diuretic in elderly hypertensive patients: a randomized, controlled trial. J Hypertens.

[571] Liu L, Zhang Y, Liu G, Li W, Zhang X, Zanchetti A (2005). The Felodipine Event Reduction (FEVER) Study: a randomized long-term placebo-controlled trial in Chinese hypertensive patients. J Hypertens.

[572] Staessen JA, Fagard R, Thijs L, Celis H, Arabidze GG, Birkenhäger WH, The Systolic Hypertension in Europe (Syst-Eur) Trial Investigators (1997). Randomised double-blind comparison of placebo and active treatment for older patients with isolated systolic hypertension. Lancet.

[573] Liu L, Wang JG, Gong L, Liu G, Staessen JA, Systolic Hypertension in China (Syst-China) Collaborative Group (1998). Comparison of active treatment and placebo in older Chinese patients with isolated systolic hypertension. J Hypertens.

[574] Coope J, Warrender TS (1986). Randomised trial of treatment of hypertension in elderly patients in primary care. Br Med J Clin Res.

[575] Webster R, Salam A, Silva HA, Selak V, Stepien S, Rajapakse S (2018). Fixed Low-Dose Triple Combination Antihypertensive Medication vs Usual Care for Blood Pressure Control in Patients With Mild to Moderate Hypertension in Sri Lanka: A Randomized Clinical Trial. JAMA.

[576] Emdin CA, Rahimi K, Neal B, Callender T, Perkovic V, Patel A (2015). Blood pressure lowering in type 2 diabetes: a systematic review and meta-analysis. JAMA.

[577] Benjamin EJ, Virani SS, Callaway CW, Chamberlain AM, Chang AR, Cheng S (2018). Heart disease and stroke statistics – 2018 update: a report from the American Heart Association. Circulation.

[578] Gaede P, Vedel P, Larsen N, Jensen Gunnar VH, Parving H-H, Pedersen O (2003). Multifactorial intervention and cardiovascular disease in patients with type 2 diabetes. N Engl J Med.

[579] (1993). Hypertension in Diabetes Study (HDS): I. Prevalence of hypertension in newly presenting type 2 diabetic patients and the association with risk factors for cardiovascular and diabetic complications. J Hypertens.

[580] James PA, Oparil S, Carter BL, Cushman WC, Dennison-Himmelfarb C, Handler J (2014). 2014 evidence-based guideline for the management of high blood pressure in adults: report from the panel members appointed to the Eighth Joint National Committee (JNC 8). JAMA.

[581] Bangalore S, Kumar S, Lobach I, Messerli FH (2011). Blood pressure targets in subjects with type 2 diabetes mellitus/impaired fasting glucose: observations from traditional and Bayesian random-effects meta-analyses of randomized trials. Circulation.

[582] Niskanen L, Hedner T, Hansson L, Lanke J, Niklason A, CAPPP Study Group (2001). Reduced cardiovascular morbidity and mortality in hypertensive diabetic patients on first-line therapy with an ACE inhibitor compared with a diuretic/ beta-blocker-based treatment regimen: a sub analysis of the Captopril Prevention Project. Diabetes Care.

[583] Ostergren J, Poulter NR, Sever PS, Dahlof B, Wedel H, Beevers G (2008). The Anglo-Scandinavian Cardiac OutcomesTrial: blood pressure-lowering limb: effects in patients with type II diabetes. J Hypertens.

[584] Weber MA, Bakris GL, Jamerson K, Weir M, Kjeldsen SE, Devereux RB (2010). Cardiovascular events during differing hypertension therapies in patients with diabetes. J Am Coll Cardiol.

[585] Tocci G, Paneni F, Palano F, Sciarretta S, Ferrucci A, Kurtz T (2011). Angiotensin-converting enzyme inhibitors, angiotensin II receptor blockers and diabetes: a meta-analysis of placebo-controlled clinical trials. Am J Hypertens.

[586] Yanai H, Tomono Y, Ito K, Furutani N, Yoshida H, Tada N (2008). The underlying mechanisms for development of hipertension in the metabolic syndrome. Nutr J.

[587] Alberti KG, Zimmet P, Shaw J (2006). Metabolic Syndrome - a new world-wide definition. A consensus statement from the International Diabetes Federation. Diabet Med.

[588] Lebovitz HE, Banerji MA (2005). Point: visceral adiposity is causally related to insulin resistance. Diabetes Care.

[589] Kahn R, Buse J, Ferrannini E, Stern M (2005). The metabolic Syndrome: Time for a critical appraisal: Joint statement from American Diabetes Association and European Association for the Study of Diabetes. Diabetes Care.

[590] Gagliardi ART (2004). Obesidade central, bases hormonais e moleculares da síndrome metabólica. Rev Soc Cardiol Estado de São Paulo.

[591] Tuomilehto J, Lindstrom J, Eriksson JG, Valle TT, Hamalainen H, Ilanne-Parikka P (2001). Prevention of type 2 diabetes mellitus by changes in life style among subjects with impaired glucose tolerance. N Engl J Med.

[592] Grundy SM, Cleeman JI, Daniels SR, Donato KA, Eckel RH, Franklin BA (2005). Diagnosis and management of the metabolic syndrome: an American Heart Association/National Heart, Lung, and Blood Institute Scientific Statement. Circulation.

[593] Stears AJ, Woods SH, Watts MM, Burton TJ, Graggaber J, Mir FA, Brown MJ (2012). A double-blind, placebo-controlled, crossover trial comparing the effects of amiloride and hydrochlorothiazide on glucose tolerance in patients with essential hypertension. Hypertension.

[594] Borghi C, Bacchelli S, Degli Esposti D, Bignamini A, Magnani B, Ambrosioni E (1999). Effects of the administration of an angiotensin-converting enzyme inhibitor during the acute pHAe of myocardial infarction in patients with arterial hypertension. SMILE Study Investigators. Survival of Myocardial Infarction Long-term Evaluation. Am J Hypertens.

[595] Gustafsson F, Kober L, Torp-Pedersen C, Hildebrandt P, Ottesen MM, Sonne B, TRACE study group (1998). Long-term prognosis after acute myocardial infarction in patients with a history of arterial hypertension. Eur Heart J.

[596] Arnold JM, Yusuf S, Young J (2003). Prevention of heart failure in patients in the heart outcomes prevention evaluation (HOPE) study. Circulation.

[597] Messerli FH, Mancia G, Conti CR, Hewkin AC, Kupfer S, Champion A (2006). Dogma disputed: can aggressively lowering blood pressure in hypertensive patients with coronary artery disease be dangerous?. Ann Intern Med.

[598] Sleight P, Redon J, Verdecchia P, Mancia G, Gao P, Fagard R, ONTARGET investigators (2009). Prognostic value of blood pressure in patients with high vascular risk in the Ongoing Telmisartan Alone and in combination with Ramipril Global Endpoint Trial study. J Hypertens.

[599] Zanchetti A, Mancia G (2012). Longing for clinical excellence: a critical outlook into the NICE recommendations on hypertension management--is nice always good?. J Hypertens.

[600] Mancia G, Parati G, Bilo G, Gao P, Fagard R, Redon J (2012). Ambulatory blood pressure values in the Ongoing Telmisartan Alone and in Combination with Ramipril Global Endpoint Trial (ONTARGET). Hypertension.

[601] Vidal-Petiot E, Ford I, Greenlaw N, Ferrari R, Fox KM, Tardif JC (2016). Cardiovascular event rates and mortality according to achieved systolic and diastolic blood pressure in patients with stable coronary artery disease: an international cohort study. Lancet.

[602] Xie X, Atkins E, Lv J, Bennett A, Neal B, Ninomiya T (2016). Effects of intensive blood pressure lowering on cardiovascular and renal outcomes: updated systematic review and meta-analysis. Lancet.

[603] Wright JT, Bakris G, Greene T, Agodoa LY, Appel LJ, Charleston J (2002). African American Study of Kidney Disease and Hypertension Study Group: Effect of blood pressure lowering and antihypertensive drug class on progression of hypertensive kidney disease: Results from the AASK trial. JAMA.

[604] Klahr S, Levey AS, Beck GJ, Caggiula AW, Hunsicker L, Kusek JW (1994). Modification of Diet in Renal Disease Study Group: The effects of dietary protein restriction and blood-pressure control on the progression of chronic renal disease. N Engl J Med.

[605] Malhotra R, Nguyen HA, Benavente O, Mete M, Howard BV, Mant J (2017). Association between more intensive vs less intensive blood pressure lowering and risk of mortality in chronic kidney disease stages 3 to 5: a systematic review and meta-analysis. JAMA Intern Med.

[606] Reboldi G, Gentile G, Angeli F, Ambrosio G, Mancia G, Verdecchia P (2011). Effects of intensive blood pressure reduction on myocardial infarction and stroke in diabetes: a meta-analysis in 73,913 patients. J Hypertens.

[607] Cushman WC, Evans GW, Byington RP, Goff DC, Grimm RH, Cutler JA, ACCORD Study Group (2010). Effects of intensive blood-pressure control in type 2 diabetes mellitus. N Engl J Med.

[608] Fried LF, Emanuele N, Zhang JH, Brophy M, Conner TA, Duckworth W, VA NEPHRON-D Investigators (2013). Combined angiotensin inhibition for the treatment of diabetic nephropathy. N Engl J Med.

[609] Bakris GL, Sarafidis PA, Weir MR, Dahlöf B, Pitt B, Jamerson K, ACCOMPLISH Trial Investigators (2010). Renal outcomes with different fixed-dose combination therapies in patients with hypertension at high risk for cardiovascular events (ACCOMPLISH): a prespecified secondary analysis of a randomized controlled trial. Lancet.

[610] Foody JM, Farrell MH, Krumholz HM (2002). beta-blocker therapy in heart failure: Scientific review. JAMA.

[611] Clinical Trial (2020). Efficacy and Safety of Finerenone in Subjects with Type 2 Diabetes Mellitus and the Clinical Diagnosis of Diabetic Kidney Disease (NCT02540993).

[612] Agarwal R, Peixoto AJ, Santos SF, Zoccali C (2006). Pre- and post-dialysis blood pressures are imprecise estimates of interdialytic ambulatory blood pressure. CJASN.

[613] Silva GV, Barros S, Abensur H, Ortega KC, Mion D, Cochrane Renal Group Prospective Trial Register: CRG060800146 (2009). Home blood pressure monitoring in monitoring in blood pressure control among hemodialysis patients: an open randomized clinical trial. Nephrol Dial Transplant.

[614] Georgianos PI, Agarwal R (2017). Blood pressure and mortality in long-term hemodialysis-time to move forward. Am J Hypertens.

[615] Bansal N, McCulloch CE, Lin F, Alper A, Anderson AH, Cuevas M (2017). Blood pressure and risk of cardiovascular events in patients on chronic hemodialysis: the CRIC study (Chronic Renal Insufficiency Cohort). Hypertension.

[616] Georgianos PI, Agarwal R (2019). Systolic and diastolic hypertension among patients on hemodialysis: Musings on volume overload, arterial stiffness, and erythropoietin. Semin Dial.

[617] Tada T, Kusano KF, Ogawa A, Iwasaki J, Sakuragi J, Kusano I (2007). The predictors of central and obstructive sleep apnoea in haemodialysis patients. Nephrol Dial Transplant.

[618] Agarwal R, Sinha AD, Pappas MK, Abraham TN, Tegegne GG (2014). Hypertension in hemodialysis patients treated with atenolol or lisinopril: A randomized controlled trial. Nephrol Dial Transplant.

[619] Kishnan N, Peixoto AJ (2016). We hold antihypertensives prior to dialysis. Semin Dial.

[620] Agarwal R, Sinha AD (2019). Clinical pharmacology of antihypertensive therapy for the treatment of hypertension in chronic kidney disease. Clin J Am Soc of Nephrol.

[621] Cross NB, Webster AC, Masson P, O’ Connel PJ, Craig JC (2009). Antihypertensive treatment for kidney transplant recipients. Transplantation.

[622] Ibrahim HN, Jackson S, Connaire J, Matas A, Ney A, Najafian B (2013). Angiotensin II blockade in kidney transplant recipients. J Am Soc Nephrol.

[623] Stokes J, Kannel WB, Wolf PA, D’Agostino RB, Cupples LA (1989). Blood pressure as a risk factor for cardiovascular disease the framingham study-30 years of follow-up. Hypertension.

[624] Kannel WB (2000). Incidence and epidemiology of heart failure. Heart Fail Rev.

[625] Goyal D, Macfadyen RJ, Watson RD, Lip GYH (2005). Ambulatory blood pressure monitoring in heart failure: A systematic review. Eur J Heart Fail.

[626] Pfeffer MA (2017). Heart Failure and Hypertension Importance of Prevention Heart failure Randomized clinical trials Antihypertensive agents Prevention.

[627] Moser M, Hebert PR (1996). Prevention of disease progression, left ventricular hypertrophy and congestive heart failure in hypertension treatment trials. J Am Coll Cardiol.

[628] Tadic M, Cuspidi C, Frydas A, Grassi G (2018). The role of arterial hypertension in development heart failure with preserved ejection fraction: just a risk factor or something more?. Heart Fail Rev.

[629] Kostis JB, Davis BR, Cutler J, Grimm RH, Berge KG, Cohen JD (1997). Prevention of heart failure by antihypertensive drug treatment in older persons with isolated systolic hypertension. J Am Med Assoc.

[630] Thomopoulus C, Parati G, Zanchetti A (2016). Effects of blood pressure-lowering treatment .6.Prevention of heart failure and new-onset heart failure–meta-analyses of randomized trials. J Hypertens.

[631] Yancy CW, Jessup M, Bozkurt B, Butler J, Casey DE, Colvin MM (2017). 2017 ACC/AHA/HFSA Focused Update of the 2013 ACCF/AHA Guideline for the Management of Heart Failure: A Report of the American College of Cardiology / American Heart Association Task Force on Clinical Practice Guidelines and the Heart Failure Society of America. Circulation.

[632] Rohde LEP, Montera MW, Bocchi EA, Clausell NO, Albuquerque DC, Rassi S (2018). Diretriz Brazileira de insuficiência cardíaca crônica e aguda. Arq Bras Cardiol.

[633] McMurray JJ, Packer M, Desai AS, Gong J, Lefkowitz MP, Rizkala AR, PARADIGM-HF Investigators and Committees (2014). Angiotensin-neprilysin inhibition versus enalapril in heart failure. N Engl J Med.

[634] Yusuf S, Pfeffer MA, Swedberg K, Granger CB, Held P, McMurray JJV (2003). Effects of candesartan in patients with chronic heart failure and preserved left-ventricular ejection fraction: The CHARM-preserved trial. Lancet.

[635] Forman D, Gaziano JM (2009). Irbesartan in patients with heart failure and preserved ejection fraction. Curr Cardiovasc Risk Rep.

[636] Cleland JGF, Tendera M, Adamus J, Freemantle N, Polonski L, Taylor J (2006). The perindopril in elderly people with chronic heart failure (PEP-CHF) study. Eur Heart J.

[637] Yamamoto K, Origasa H, Hori M (2013). Effects of carvedilol on heart failure with preserved ejection fraction: The Japanese Diastolic Heart Failure Study (J-DHF). Eur J Heart Fail.

[638] Pitt B, Pfeffer MA, Assmann SF, Boineau R, Anand IS, Claggett B (2014). Spironolactone for heart failure with preserved ejection fraction. N Engl J Med.

[639] Solomon SD, McMurray JJV, Anand IS, Ge J, Lam CSP, Maggioni AP (2019). Angiotensin–neprilysin inhibition in heart failure with preserved ejection fraction. N Engl J Med.

[640] Lee TT, Chen J, Cohen DJ, Tsao L (2006). The association between blood pressure and mortality in patients with heart failure. Am Heart J.

[641] Kalantar-Zadeh K, Block G, Horwich T, Fonarow GC (2004). Reverse epidemiology of conventional cardiovascular risk factors in patients with chronic heart failure. J Am Coll Cardiol.

[642] Rouleau JL, Roecker EB, Tendera M, Mohacsi P, Krum H, Katus HA (2004). Influence of pretreatment systolic blood pressure on the effect of carvedilol in patients with severe chronic heart failure: The Carvedilol Prospective Randomized Cumulative Survival (COPERNICUS) study. J Am Coll Cardiol.

[643] Anand IS, Rector TS, Kuskowski M, Thomas S, Holwerda NJ, Cohn JN (2008). Effect of baseline and changes in systolic blood pressure over time on the effectiveness of valsartan in the Valsartan Heart FailureTrial. Circ Heart Fail.

[644] Böhm M, Young R, Jhund PS, Solomon SD, Gong J, Lefkowitz MP (2017). Systolic blood pressure, cardiovascular outcomes and efficacy and safety of sacubitril/ valsartan (LCZ696) in patients with chronic heart failure and reduced ejection fraction: Results from PARADIGM-HF. Eur Heart J.

[645] Rashid P, Leornardi-Bee J, Bath P (2003). Blood pressure reduction and secondary prevention of stroke and other vascular events: a systematic review. Stroke.

[646] PATS Collaborating Group (1995). Poststroke antihypertensive treatment study: a preliminary result. Chin Med J (Engl)..

[647] Gueyffier F, Boissel JP, Boutitie F, Pocock S, Coope J, Cutler J, The INDANA (IN-dividual Data Analysis of Antihypertensive intervention trials) Project Collaborators (1997). Effect of antihypertensive treatment in patients having already suffered from stroke. Gathering the evidence. Stroke.

[648] Liu L, Wang Z, Gong L, Zhang Y, Thijs L, Staessen JA (2009). Blood pressure reduction for the secondary prevention of stroke: a Chinese trial and a systematic review of the literature. Hypertens Res.

[649] Schrader J, Luders S, Kulschewski A, Hammersen F, Plate K, Berger J, MOSES Study Group (2005). Morbidity and mortality after stroke, eprosartan compared with nitrendipine for secondary prevention: principal results of a prospective randomized controlled study (MOSES). Stroke.

[650] Yusuf S, Diener HC, Sacco RL, Cotton D, Ounpuu S, Lawton WA, ProFESS Study Group (2008). Telmisartan to prevent recurrent stroke and cardiovascular events. N Engl J Med.

[651] White CL, Pergola PE, Szychowski JM, Talbert R, Cervantes-Arriaga A, Clark HD, SPS3 Investigators (2013). Blood pressure after recent stroke: baseline findings from the secondary prevention of small subcortical strokes trial. Am J Hypertens.

[652] Powers WJ, Rabinstein AA, Ackerson T, Adeoye OM, Bambakidis NC, Becker K (2018). 2018 Guidelines for the Early Management of Patients With Acute Ischemic Stroke: A Guideline for Healthcare Professionals From the American Heart Association/American Stroke Association. Stroke.

[653] Rodriguez-Luna D, Pineiro S, Rubiera M, Ribo M, Coscojuela P, Pagola J (2013). Impact of blood pressure changes and courseon hematoma growth in acute intracerebral hemorrhage. Eur J Neurol.

[654] Anderson CS, Heeley E, Huang Y, Wang J, Stapf C, Delcourt C, INTERACT2 Investigators (2013). Rapid blood-pressure lowering in patients with acute intracerebral hemorrhage. N Engl J Med.

[655] Ahmed N, Wahlgren N, Brainin M, Castillo J, Ford GA, Kaste M (2009). Relationship of blood pressure, antihypertensive therapy, and outcome inischemic stroke treated with intravenous thrombolysis: retrospective analysisfrom Safe Implementation of Thrombolysis in Stroke-International StrokeThrombolysis Register (SITS-ISTR). Stroke.

[656] Wu W, Huo X, Zhao X, Liao X, Wang C, Pan Y (2016). Relationship between blood pressure and outcomes in acute ischemic strokepatients administered lytic medication in the TIMS-China Study. PLoS One.

[657] Lee M, Ovbiagele B, Hong KS, Wu YL, Lee JE, Rao NM (2015). Effect of blood pressure lowering in early ischemic stroke: meta-analysis. Stroke.

[658] Zhao R, Liu FD, Wang S, Peng JL, Tao XX, Zheng B (2015). Blood pressure reduction in the acute phase of an ischemic stroke does not improve short- or longtermdependency or mortality: a meta-analysis of current literature. Medicine(Baltimore).

[659] Bateman BT, Bansil P, Hernandez-Diaz S, Mhyre JM, Callaghan WM, Kuklina EV (2012). Prevalence, trends, and out-comes of chronic hypertension: a nationwide sample of delivery admissions. Am J Obstet Gynecol.

[660] Steegers EA, von Dadelszen P, Duvekot JJ, Pijnenborg R (2010). Pre-eclampsia. Lancet.

[661] Ramos JGL, Sass N, Costa SHM (2017). Preeclampsia. Rev Bras Ginecol Obstet.

[662] Abalos E, Cuesta C, Grosso AL, Chou D, Say L (2013). Global and regional estimates of preeclampsia and eclampsia: a systematic review. Eur J Obstet Gynecol Reprod Biol.

[663] Giordano JC, Parpinelli MA, Cecatti JG, Haddad SM, Costa ML, Surita FG (2014). The burden of eclampsia: results from a multicenter study on surveillance of severe maternal morbidity in Brazil. PLoS One.

[664] American College of Obstetricians and Gynecologists (ACOG) (2019). ACOG Practice Bulletin No. 202: Gestational Hypertension and Preeclampsia. Obstet Gynecol.

[665] American College of Obstetricians and Gynecologists (ACOG) (2019). ACOG Practice Bulletin No. 203: Chronic Hypertension in Pregnancy. Obstet Gynecol.

[666] Brown MA, Magee LA, Kenny LC, Karumanchi SA, McCarthy FP, Saito S (2018). The hypertensive disorders of pregnancy: ISSHP classification, diagnosis & management recommendations for international practice. Pregnancy Hypertens.

[667] Hofmey GJ, Lawrie TA, Atallah ÁN, Duley L (2014). Calcium supplementation during pregnancy for preventing hypertensive disorders and related problems. Cochrane Database Syst Rev.

[668] CLASP (Collaborative Low dose Aspirin Study in Pregnancy) Collaborative Group (1994). CLASP: a randomized trial of low-dose aspirin for the prevention and treatment of pre-eclampsia among 9364 pregnant women. Lancet.

[669] Duley L, Henderson-Damart DJ, Meher S, King JF (2004). Antiplatelet agents for preventing pre-eclampsia and its complications. Cochrane Database of Syst Rev.

[670] Rolnik DL, Wright D, Poon L, O’Gorman N, Syngelaki A, Paco Matallana C (2017). Aspirin versus placebo in pregnancies at high risk of preterm preeclampsia. N Engl J Med.

[671] (2019). Hypertension in pregnancy: diagnosis and management NICE guideline Published.

[672] World Health Organization. WHO (2011). Recommendations for Prevention and Treatment of Pre-Eclampsia and Eclampsia.

[673] Poon LC, Shennan A, Hyett JA, Kapur A, Hadar E, Divakar H (2019). The International Federation of Gynecology and Obstetrics (FIGO) initiative on pre-eclampsia: A pragmatic guide for first-trimester screening and prevention. Int J Gynaecol Obstet.

[674] American College of Obstetricians and Gynecologists (ACOG) (2019). ACOG Committee Opinion No. 767: Emergent Therapy for Acute-Onset, Severe Hypertension During Pregnancy and the Postpartum Period. Obstet Gynecol.

[675] Martin JN, Thigpen BD, Moore RC, Rose CH, Cushman J, May W (2005). Stroke and severe preeclampsia and eclampsia: a paradigm shift focusing on systolic blood pressure. Obstet Gynecol.

[676] Cantwell R, Clutton-Brock T, Cooper G, Dawson A, Drife J, Garrod D (2011). Saving Mothers’ Lives: Reviewing maternal deaths to make motherhood safer: 2006-2008. The Eighth Report of the Confidential Enquiries into Maternal Deaths in the United Kingdom. BJOG.

[677] Meher S, Abalos E, Carroli G (2005). Bed rest with or without hospitalisation for hypertension during pregnancy. Cochrane Database Syst Rev.

[678] Dowswell T, Middleton P, Weeks A (2009). Antenatal day care units versus hospital admission for women with complicated pregnancy. Cochrane Database of Syst Rev.

[679] Koopmans CM, Bijlenga D, Groen H, Vijgen SMC, Aarnoudse JG, Bekedam DJ (2009). Induction of labour versus expectant monitoring for gestational hypertension or mild pre-eclampsia after 36 weeks’ gestation (HYPITAT): a multicentre, open-label randomised controlled trial. Lancet.

[680] Broekhuijsen K, van Baaren GJ, van Pampus MG, Ganzevoort W, Sikkema JM, Woiski MD (2015). Immediate delivery versus expectant monitoring for hypertensive disorders of pregnancy between 34 and 37 weeks of gestation (HYPITAT-II): an open-label, randomised controlled trial. Lancet.

[681] Ronsmans C, Campbell O (2011). Quantifying the fall in mortality associated with interventions related to hypertensive diseases of pregnancy. BMC Public Health.

[682] Peraçoli JC, Borges VTM, Ramos JG, Cavalli RC, Costa SHAM, Oliveira LG (2019). Pre-eclampsia/Eclampsia. Rev Bras Ginecol Obstet.

[683] Duley L, Henderson-Smart DJ, Meher S (2006). Drugs for treatment of very high blood pressure during pregnancy. Cochrane Database Syst Rev.

[684] control Centers for disease, prevention (1997). Postmarketing surveillance for angiotensin-converting enzyme inhibitor use during the first trimester of pregnancy – United States, Canada and Israel, 1987-1995. JAMA.

[685] Abalos E, Duley L, Steyn DW (2014). Antihypertensive drug therapy for mild to moderate hypertension during pregnancy. Cochrane Database of Syst Rev.

[686] Magee LA, von Dadelszen P, Rey E, Ross S, Asztalos E, Murphy KE (2015). Less-tight versus tight control of hypertension in pregnancy. N Engl J Med.

[687] Magee LA, von Dadelszen P, Singer J, Lee T, Rey E, Ross S (2016). The CHIPS randomized controlled trial (Control of Hypertension in Pregnancy Study): is severe hypertension just an elevated blood pressure?. Hypertension.

[688] Butalia S, Audibert F, Côté AM, Firoz T, Logan AG, Magee LA (2018). Hypertension Canada’s 2018. Guidelines for the Management of Hypertension in Pregnancy. Can J Cardiol.

[689] Easterling T, Mundle S, Bracken H, Parvekar S, Mool S, Magee LA (2019). Oral antihypertensive regimens (nifedipine retard, labetalol, and methyldopa) for management of severe hypertension in pregnancy: an open-label, randomised controlled trial. Lancet.

[690] Sass N, Itamoto CH, Silva MP, Torloni MR, Atallah NA (2007). Does sodium nitroprusside kill babies? A systematic review. Sao Paulo Med J.

[691] Townsend R, O’Brien P, Khalil A (2016). Current best practice in the management of hypertensive disorders in pregnancy. Integr Blood Press Control.

[692] (2006). Drugs and Lactation Database (LactMed).

[693] Regit-Azgrosek V, Roos-Hesselink JW, Bauersachs J, Blomström-Lundqvist C, Cífková R, Bonis M (2018). 2018 ESC guidelines for the management of cardiovascular diseases during pregnancy. Eur Heart J.

[694] Halpern DG, Weinberg CR, Pinnelas R, Mehta-Lee S, Economy KE, Valente AM (2019). Use of Medication for Cardiovascular Disease During Pregnancy: JACC State-of-the-Art Review. J Am Coll Cardiol.

[695] Noronha C C, Maia SSB, Katz L, Coutinho IC, Souza AR, Amorim MM (2017). Clonidine versus Captopril for Severe Postpartum Hypertension: A Randomized Controlled Trial. Plos One.

[696] Ray JG, Vermeulen MJ, Schull MJ, Redelmeier DA (2005). Cardiovascular health after maternal placental syndromes (CHAMPS): population-based retrospective cohort study. Lancet.

[697] Bellamy L, Casas JP, Hingorani AD, Williams DJ (2007). Pre-eclampsia and risk of cardiovascular disease and cancer in later life: systematic review and meta-analysis. BMJ.

[698] Honigberg MC, Zekavat SM, Aragam K, Klarin D, Bhatt DL, Scott NS (2019). Long-Term Cardiovascular Risk in Women With Hypertension During Pregnancy. J Am Coll Cardiol.

[699] Vikse BE, Irgens LM, Leivestad T, Skjaerven R, Iversen BM (2008). Preeclampsia and the risk of end-stage renal disease. N Engl J Med.

[700] Sorof JM, Lai D, Turner J, Poffenbarger T, Portman RJ (2004). Overweight, ethnicity, and the prevalence of hypertension in school-aged children. Pediatrics.

[701] McNiece KL, Poffenbarger TS, Turner JL, Franco KD, Sorof JM, Portman RJ (2007). Prevalence of hypertension and prehypertension among adolescents. J Pediatr.

[702] Brian KK, Elena K, Margaret DC, Yachim O, David SF, Cynthia LO (2015). Prevalence of and trends in dyslipidemia and blood pressure among us child and adolescents 1999-2012. Jama Pediatr.

[703] Bloch VK, Klein CH, Szklo M, Kuschnir MCC (2016). Prevalências de hipertensão arterial e obesidade Brazileiros. Rev. Saúde Pública.

[704] Brady TM, Redwine KM, Flynn JT, American Society of Pediatric Nephrology (2014). Screening blood pressure measurement in children: are we saving lives?. Pediatr Nephrol.

[705] Flynn JT, Kaelber DC, Baker-Smith CM, Blowey D, Carroll AE, Subcommittee on Screening and Management of High Blood Pressure in Children (2017). Clinical Practice Guideline for Screening and Management of High Blood Pressure in Children and Adolescents. Pediatrics.

[706] National High Blood Pressure Education Program Working Group on Hypertension Control in Children and Adolescents (1996). Update on the 1987 Task Force Report on High Blood Pressure in Children and Adolescents: a working group report from the National High Blood Pressure Education Program.. Pediatrics.

[707] National High Blood Pressure Education Program Working Group on High Blood Pressure in Children and Adolescents (2004). The Fourth Report on the Diagnosis, Evaluation, and Treatment of High Blood Pressure in Children and Adolescents. Pediatrics.

[708] Kulaga Z, Litwin M, Grajda A, Kułaga K, Gurzkowska B, Gó´zd´ zM (2012). Oscillometric blood pressure percentiles for Polish normal-weight school-aged children and adolescents. J Hyper-tens.

[709] Neuhauser HK, Thamm M, Ellert U, Hense HW, Rosario AS (2011). Blood pressure percentiles by age and height from nonover-weight children and adolescents in Germany. Pediatrics.

[710] Jardim TV, Rosner B, Bloch KV, Kuschnir MC, Szklo M, Jardim PC (2020). Blood pressure reference values for Brazilian adolescents: data from the Study of Cardiovascular Risk in Adolescents (ERICA Study). J Pediatr.

[711] Dionne JM, Abitbol CL, Flynn JT (2012). Hypertension in infancy: diagnosis, management and outcome. Pediatr Nephrol.

[712] (1987). Report of the second task force on blood pressure control in children–1987. Task force on blood pressure control in children. National Heart, Lung, and Blood Institute, Bethesda, Maryland. Pediatrics.

[713] Guimarães IC, Almeida AM, Santos AS, Barbosa DB, Guimarães AC (2008). Blood pressure: effect of body mass index and of waist circumference on adolescents. Arq Bras Cardiol.

[714] Sr Daniels, Moss & Adams (2013). Heart disease in infants, children and adolescents.

[715] Lurbe E, Cifkova R, Cruickshank JK, Dillon MJ, Ferreira I, Invitti C (2009). Management of high blood pressure in children and adolescents: recommendations of the European Society of Hypertension. J Hypertens.

[716] Flynn JT, Daniels SR, Hayman LL, Maahs DM, McCrindle BW, Mitsnefes M, Zachariah JP, Urbina EM (2014). Update: ambulatory blood pressure monitoring in children and adolescents: a scientific statement from the American Heart Association. Hypertension.

[717] Hansen HS, Hyldebrandt N, Froberg K, Nielsen JR (1990). Blood pressure and physical fitness in a population of children—the Odense Schoolchild Study. J Hum Hypertens.

[718] McCambridge TM, Benjamin HJ, Brenner JS, Cappetta CT, Demorest RA, Gregory AJ (2010). Athletic participation by children and adolescents who have systemic hypertension. Pediatrics.

[719] Rios-Leyvraz M, Bloetzer C, Chatelan A, Bochud M, Burnier M (2019). Sodium intake and blood pressure in children with clinical conditions: A systematic review with meta-analysis. J Clin Hypertens (Greenwich)..

[720] Bricarello P L, Poltronieri F, Fernandes R, Retondario A, Morais EBST, Vasconcelos FAG (2018). Effects of the Dietary Approach to Stop Hypertension (DASH) diet on blood pressure, overweight and obesity in adolescents: A systematic review. Clin Nutr ESPEN.

[721] Miller JZ, Wienberger MH, Christian JC (1987). Blood pressure response to potassium supplement in normotensive adults and children. Hypertension.

[722] Chaturvedi S, Lipszyc DH, Licht C, Craig JC, Parekh P (2014). Pharmacological interventions for hypertension in children. Evid Based Child Health.

[723] Prichard BN, Cruickshank JM, Graham BR (2001). Beta-adrenergic blocking drugs in the treatment of hypertension. Blood Press.

[724] Bullo M, Tschumi S, Bucher BS, Bianchetti MG, Simonetti GD (2012). Pregnancy outcome following exposure to angiotensin converting enzyme inhibitors or angiotensin receptor antagonists: a systematic review. Hypertension.

[725] Ferguson MA, Flynn JT (2014). Rational use of antihypertensive medications in children. Pediatr Nephrol.

[726] Blowey DL (2012). Update on the pharmacologic treatment of hypertension in pediatrics. J Clin Hypertens (Greenwich)..

[727] Nerenberg KA, Zarnke KB, Leung AA, Dasgupta K, Butalia S, Hypertension Canada (2018). Hypertension Canada’s 2018 Guidelines for Diagnosis, Risk Assessment, Prevention, and Treatment of Hypertension in Adults and Children. Can J Cardiol.

[728] (2016). 2016 European Society of Hypertension guidelines for the management of high blood pressure in children and adolescents. J Hypertens.

[729] Wu HP, Yang WC, Wu YK, Zhao L, Chen CY, Fu YC (2012). Clinical significance of blood pressure ratios in hypertensive crisis in children. Arch Dis Child.

[730] Chandar J, Zilleruelo G (2012). Hypertensive crisis in children. Pediatr Nephrol.

[731] Yang WC, Zhao LL, Chen CY, Wu YK, Chang YJ, Wu HP (2012). First-attack pediatric hypertensive crisis presenting to the pediatric emergency department. BMC Pediatrics.

[732] (1993). The fifth report of the Joint National Committee on Detection, Evaluation, and Treatment of High Blood Pressure (JNC V). Arch Intern Med.

[733] Bortolotto LA, Silveira JV, Vilela-Martin JF (2018). Crises Hipertensivas: Definindo a gravidade e o tratamento. Rev Soc Cardiol Estado de São Paulo.

[734] Martin JFV, Ribeiro JM, Moreira MC, Montenegro ST, Paola AAV (2015). Livro Texto da Sociedade Brazileira de Cardiologia.

[735] Martin JF, Higashiama E, Garcia E, Luizon MR, Cipullo JP (2004). Hypertensive crisis profile. Prevalence and clinical presentation. Arq Bras Cardiol.

[736] Pinna G, Pascale C, Fornengo P, Arras S, Piras C, Panzarasa P (2014). Hospital admissions for hypertensive crisis in the emergency departments: a large multicenter Italian study. PLoS One.

[737] Vilela-Martin JF, Vaz-de-Melo RO, Kuniyoshi CH, Abdo AN, Yugar-Toledo JC (2011). Hypertensive crisis: clinical-epidemiological profile. Hypertens Res.

[738] Pierin AMG, Flórido CF, Santos JD (2019). Hypertensive crisis: clinical characteristics of patients with hypertensive urgency, emergency and pseudocrisis at a public emergency department. Einstein (Sao Paulo).

[739] Keith NM, Wagener HP, Barker NW (1974). Some different types of essential hypertension: their course and prognosis. Am J Med Sci.

[740] Velasco I, Cuadrado L, Fontana A, Reijaili WA, Balbi AL, Barretti P, Franco RJS (1993). Cuadro clínico y evolución de 77 pacientes con hipertensión arterial maligna: comparación de dos épocas y de diferentes niveles de creatinina. Nefrologia.

[741] Almeida FA, Stella RC, Voos A, Ajzen H, Ribeiro AB (1981). Malignant hypertension: a syndrome associated with low plasma kininogen and kinin potentiating factor. Hypertension.

[742] Ault MJ, Ellrodt AG (1985). Pathophysiologic events leading to the end-organ effects of acute hypertension. Am J Emerg Med.

[743] Strandgaard S, Paulson O (1984). Cerebral autoregulation. Stroke.

[744] Martin JFV, Kuniyoshi CH, Andrade LG, Yugar-Toledo JC, Loureiro AC, Cipullo JP (2007). Fatores Preditores de Mortalidade em Pacientes com Crise Hipertensiva. Arq Bras Cardiol.

[745] Grossman E, Messerli FH, Grodzicki T, Kowey P (1996). Should a moratorium be placed on sublingual nifedipine capsules given for hypertensive emergencies and pseudoemergencies?. JAMA.

[746] (2004). CREMESP elabora parecer sobre uso de nifedipina.

[747] Vilela-Martin JF, Yugar-Toledo JF, Rodrigues MC, Barroso WS, Bronze L, Torres F (2020). Luso-Brazilian Position Statement on Hypertensive Emergencies – 2020. Arq Bras Cardiol.

[748] van den Born BH, Lip GYH, Brguljan-Hitij J, Cremer A, Segura J, Morales E (2019). ESC Council on hypertension position document on the management of hypertensive emergencies. Eur Heart J Cardiovasc Pharmacother.

[749] O’Donnell MJ, Chin SL, Rangarajan S, Xavier D, Liu L, Zhang H (2016). Global and regional effects of potentially modifiable risk factors associated with acute stroke in 32 countries (INTERSTROKE): a case-control study. Lancet.

[750] Truelsen T, Heuschmann PU, Bonita R, Arjundas G, Dalal P, Damasceno A (2007). Standard method for developing stroke registers in low-income and middle-income countries: experiences from a feasibility study of a stepwise approach to stroke surveillance (STEPS Stroke). Lancet Neurol.

[751] Jovin TG, Chamorro A, Cobo E, Miquel MA, Molina CA, Rovira A (2015). Thrombectomy within 8 hours after symptom onset in ischemic stroke. N Engl J Med.

[752] Hemphill J.C. (2015). Guidelines for the Management of Spontaneous Intracerebral Hemorrhage: A Guideline for Healthcare Professionals From the American Heart Association/American Stroke Association. Stroke.

[753] Ibanez B, James S, Agewall S, Antunes MJ, Bucciarelli-Ducci C, Bueno H (2018). 2017 ESC Guidelines for the management of acute myocardial infarction in patients presenting with ST-segment elevation. The Task Force for the management of acute myocardial infarction in patients presenting with ST-segment elevation of the European Society of Cardiology (ESC). Eur Heart J..

[754] Amsterdam EA, Wenger NK, Brindis RG, Casey DE, Ganiats TG, Holmes DR, American Heart Association Task Force on Practice Guidelines, Society for Cardiovascular Angiography and Interventions, Society of Thoracic Surgeons, American Association for Clinical Chemistry (2014). 2014 AHA/ACC Guideline for the management of patients with non–ST-elevation acute coronary syndromes. A Report of the American College of Cardiology/American Heart Association Task Force on Practice Guidelines. J Am Coll Cardiol.

[755] Gandhi SK, Powers JC, Nomeir AM, Fowle K, Kitzman DW, Rankin KM (2001). The pathogenesis of acute pulmonary edema associated with hypertension. N Engl J Med.

[756] Kumar R, Gandhi SK, Little WC (2008). Acute heart failure with preserved systolic function. Crit Care Med.

[757] Rohde LEP, Montera MW, Bocchi EA, Albuquerque DC, Clausell NO, Rassi S, Comitê Coordenador da Diretriz de Insuficiência Cardíaca da Sociedade Brazileira de Cardiologia (2018). Diretriz Brazileira de Insuficiência Cardíaca Crônica e Aguda. Arq Bras Cardiol.

[758] Bossone E, La Bounty TM, Eagle KA (2018). Acute aortic syndromes: Diagnosis and management, an update. Eur Heart J.

[759] Frishman WH, Del Vecchio A, Sanal S, Ismail A (2003). Cardiovascular manifestations of substance abuse part 2: alcohol, amphetamines, heroin, cannabis, and caffeine. Heart Dis.

[760] Frishman WH, Del Vecchio A, Sanal S, Ismail A (2003). Cardiovascular manifestations of substance abuse part 1: cocaine. Heart Dis.

[761] Lester SJ, Baggott M, Welm S (2000). Cardiovascular effects of 3, 4-methylenedioxy-methamphetamine. A double-blind, placebo-controlled trial. Ann Intern Med.

[762] Gahlinger PM (2004). Club drugs: MDMA, gamma-hydroxybutyrate (GHB), Rohypnol, and ketamine. Am Fam Physician.

[763] Lange RA, Ciggarroa RG, Flores ED, McBride W, Kim AS, Wells PJ (1990). Potentiation of cocaine induced coronary vasoconstriction by beta adrenergic blockade. Ann Intern Med.

[764] Tuncel M, Wang Z, Arbique D, Fadel PJ, Victor RG, Vongpatanasin W (2002). Mechanism of the blood pressure-raising effect of cocaine in humans. Circulation.

[765] Sofuoglu M, Brown S, Babb DA, Pentel PR, Hatsukami DK (2000). Carvedilol affects the physiological and behavioral response to smoked cocaine in humans. Drug Alcohol Depend.

[766] Sordo L, Indave BI, Barrio G, Degenhardt L, de la Fuente L, Bravo MJ (2014). Cocaine use and risk of stroke: a systematic review. Drug Alcohol Depend.

[767] Wilson LD, Jeromin J, Garvey L, Dorbandt A (2001). Cocaine, ethanol, and cocaethylene cardiotoxicity in an animal model of cocaine and ethanol abuse. Acad Emerg Med.

[768] Mehta MC, Jain AC, Billie M (2002). Effects of cocaine and alcohol alone and in combination on cardiovascular performance in dogs. Am J Med Sci.

[769] Wilkerson RD (1989). Cardiovascular effects of cocaine: enhancement by yohimbine and atropine. J Pharmacol Exp Ther.

[770] Perez-Reyes M, Jeffcoat AR (1992). Ethanol/cocaine interactions: cocaine and cocaethylene plasma concentrations and their relationship to subjective and cardiovascular effects. Life Sci.

[771] Melchert RB, Eselin JA, O’Dell JF, Welder AA (1991). Effects of nitrendipine on cocaine induced toxicity evaluated in primary myocardial cell cultures. J Pharmaceut Sci.

[772] Bortolotto LA (2014). Hipertensão acelerada-maligna. Rev Bras Hipertens.

[773] Ahmed ME, Walker JM, Beevers DG, Beevers M (1986). Lack of difference between malignant and accelerated hypertension. Br Med J (Clin Res Ed)..

[774] Kincaid-Smith P, McMichael J, Murphy EA (1958). The clinical course and pathology of hypertension with papilloedema (malignant hypertension). Q J Med.

[775] Clough C, Beevers D, Beevers M (1990). The survival of malignant hypertension in blacks, whites and Asians in Britain. J Hum Hypertens.

[776] Lip G, Beevers M, Beevers D (1995). Complications and survival of 315 patients with malignant-phase hypertension. J Hypertens.

[777] Cremer A, Amraoui F, Lip GY, Morales E, Rubin S, Segura J (2016). From malignant hypertension to hypertension-MOD: a modern definition for an old but still dangerous emergency. J Hum Hypertens.

[778] Ma H, Jiang M, Fu Z, Wang Z, Shen P, Shi H (2020). Clinical value of multiorgan damage in hypertensive crises: A prospective follow-up study. J Clin Hypertens. (Greenwich)..

[779] Lip GY, Beevers M, Dodson PM, Beevers DG (1995). Severe hypertension with lone bilateral papilloedema: a variant of malignant hypertension. Blood Press.

[780] Amraoui F, van Montfrans GA, van den Born BJ (2010). Value of retinal examination in hypertensive encephalopathy. J Hum Hypertens.

[781] World Health Organization, (WHO) World Report on Ageing and Health 2015.

[782] United Nations (2015). Department of Economic and Social Affairs Population Division. World Population Ageing.

[783] Barnett K, Mercer SW, Norbury M, Watt G, Wyke S, Guthrie B (2012). Epidemiology of multimorbidity andimplications for health care, research, and medical education: a cross-sectional study. The Lancet.

[784] Yarnall AJ, Sayer AA, Clegg A, Rockwood K, Parker S, Hindle JV (2017). New horizons in multimorbidity in older adults. Age Ageing.

[785] Nunes BP, Batista SRR, Andrade FB, Souza PRB, Lima-Costa MF, Facchini LA (2018). Multimorbidity: The Brazilian Longitudinal Study of Aging (ELSI-Brazil). Rev Saude Publica.

[786] Peters R, Beckett N, McCormack T, Fagard R, Fletcher A, Bulpitt C (2014). Treating hypertension in the very elderly—benefits, risks, and future directions, a focus on the hypertension in the very elderly trial. Eur Heart J.

[787] Iadecola C (2014). Hypertension and Dementia. Hypertension.

[788] Costa AM, Mambrini JVM, Malta DC, Lima-Costa MF, Peixoto SV (2018). Contribution of chronic diseases to the prevalence of disability in basic and instrumental activities of daily living in elderly Brazilians: the National Health Survey (2013). Cad. Saúde Pública (Online).

[789] Kelly R, Hayward C, Avolio A, O’Rourke M (1989). Noninvasive determination of age-related changes in the human arterial pulse. Circulation.

[790] Franklin SS, Wt Gustin, Wong ND, Larson MG, Weber MA, Kannel WB, The Framingham Heart Study (1997). Hemodynamic patterns of age-related changes in blood pressure. Circulation.

[791] Pearson JD, Morrell CH, Brant LJ, Landis PK, Fleg JL (1997). Age-associated changes in blood pressure in a longitudinal study of healthy men and women. J Gerontol A Biol Sci Med Sci..

[792] Lakatta EG (2007). Central arterial aging and the epidemic of systolic hypertension and atherosclerosis. J Am Soc Hypertens.

[793] O’Rourke MF, Nichols WW (2005). Aortic diameter, aortic stiffness, and wave reflection increase with age and isolated systolic hypertension. Hypertension.

[794] Niiranen TJ, Lyass A, Larson MG, Hamburg NM, Benjamin EJ, Mitchell GF (2017). Prevalence, Correlates, and Prognosis of Healthy Vascular Aging in a Western Community-Dwelling Cohort: The Framingham Heart Study. Hypertension.

[795] Freitas EGB, Souza DF, Ferreira SR (2018). Probability of At Least One High Arterial Blood Pressure Measurement in Elderly Patients with Healthy Vascular Aging in Two Years of Follow-Up. Kidney Blood Press Res.

[796] Mendonca GS, Souza DF, Alvarenga Cunha Brunelli AC, Oliveira Peres CI, Freitas EGB, Lacerda GN (2018). Arterial stiffness in elderly patients with normotension and hypertension in Brazil. J Clin Hypertens (Greenwich)..

[797] Benetos A, Gautier S, Labat C (2012). Mortality and Cardiovascular Events Are Best Predicted by Low Central/Peripheral Pulse Pressure Amplification But Not by High Blood Pressure Levels in Elderly Nursing Home Subjects: the PARTAGE (Predictive Values of Blood Pressure and Arterial Stiffness in Institutionalized Very Aged Population) study. J Am Coll Cardiol.

[798] Rimoldi SF, Scherrer U, Messerli FH (2014). Secondary arterial hypertension: when, who, and how to screen?. Eur Heart J.

[799] Quinn TJ, McArthur K, Ellis G, Stott DJ (2011). Functional assessment in older people. BMJ.

[800] Benetos A, Petrovic M, Strandberg T (2019). Hypertension Management in Older and Frail Older Patients. Circ Res.

[801] Campana EMG, Freitas EV, Brandão AA, Freitas EV, Py L (2016). Tratado de Geriatria e Gerontologia.

[802] Beckett NS, Peters R, Fletcher AE (2008). Treatment of hypertension in patients 80 years of age or older. N Engl J Med.

[803] Corrao G, Rea F, Monzio Compagnoni M, Merlino L, Mancia G (2017). Protective effects of antihypertensive treatment in patients aged 85 years or older. J Hypertens.

[804] Hansson L, Lithell H, Skoog I, Baro F, Banki CM, Carbonin PU (1999). Study on COgnition and Prognosis in the Elderly (SCOPE). Blood Press.

[805] Peters R, Beckett N, Forette F (2008). Incident dementia and blood pressure lowering in the Hypertension in the Very Elderly Trial cognitive function assessment (HYVET-COG): a double-blind, placebo controlled trial. Lancet Neurol.

[806] White WB, Wakefield DB, Moscufo N (2019). Effects of Intensive Versus Standard Ambulatory Blood Pressure Control on Cerebrovascular Outcomes in Older People (INFINITY). Circulation.

[807] Feitosa GS, Peixoto JM, Pinheiro JES (2019). Updated Geriatric Cardiology Guidelines of the Brazilian Society of Cardiology - 2019. Arq Bras Cardiol.

[808] Butrous H, Hummel SL (2016). Heart Failure in Older Adults. Can J Cardiol.

[809] Stortecky S, Schoenenberger AW, Moser A, Kalesan B, Juni P, Carrel T (2012). Evaluation of multidimensional geriatric assessment as a predictor of mortality and cardiovascular events after transcatheter aortic valve implantation. JACC Cardiovasc Interv.

[810] Whelton PK, Appel LJ, Espeland MA, TONE Collaborative Research Group (1998). Sodium reduction and weight loss in the treatment of hypertension in older persons: a randomized controlled trial of nonpharmacologic interventions in the elderly (TONE). JAMA.

[811] Mente A, O’Donnell MJ, Rangarajan S (2014). Association of urinary sodium and potassium excretion with blood pressure. N Engl J Med.

[812] Bangalore S, Messerli FH, Kostis JB, Pepine CJ (2007). Cardiovascular protection using beta-blockers: a critical review of the evidence. J Am Coll Cardiol.

[813] Wiysonge CS, Bradley HA, Volmink J, Mayosi BM, Opie LH (2017). Beta-blockers for hypertension. Cochrane Database Syst Rev.

[814] Finks SW, Rumbak MJ, Self TH (2020). Treating Hypertension in Chronic Obstructive Pulmonary Disease. N Engl J Med.

[815] Isik AT, Soysal P, Stubbs B (2018). Cardiovascular Outcomes of Cholinesterase Inhibitors in Individuals with Dementia: A Meta-Analysis and Systematic Review. J Am Geriatr Soc.

[816] Kahlaee HR, Latt MD, Schneider CR (2018). Association Between Chronic or Acute Use of Antihypertensive Class of Medications and Falls in Older Adults. A Systematic Review and Meta-Analysis. Am J Hypertens.

[817] Ang HT, Lim KK, Kwan YH, Há YC, Lim JY (2018). A Systematic Review and Meta-Analyses of the Association Between Anti-Hypertensive Classes and the Risk of Falls Among Older Adults. Drugs Aging.

[818] Mühlbauer V, Dallmeier D, Brefka S, Bollig C, Voigt-Radloff S, Denkinger M (2019). The pharmacological treatment of arterial hypertension in frail, older patients— a systematic review. Dtsch Arztebl Int.

[819] Vetrano DL, Palmer KM, Galluzzo L (2018). Hypertension and frailty: a systematic review and meta-analysis. BMJ Open.

[820] Delgado J, Masoli JAH, Bowman K (2017). Outcomes of Treated Hypertension at Age 80 and Older: Cohort Analysis of 79,376 Individuals. J Am Geriatr Soc.

[821] Masoli JAH, Delgado J, Pilling L, Strain D, Melzer D (2020). Blood pressure in frail older adults: associations with cardiovascular outcomes and all-cause mortality. Age Ageing.

[822] Benetos A, Labat C, Rossignol P (2015). Treatment with multiple blood pressure medications, achieved blood pressure, and mortality in older nursing home residents: the PARTAGE Study. JAMA Intern Med.

[823] Wu C, Smit E, Peralta CA, Sarathy H, Odden MC (2017). Functional Status Modifies the Association of Blood Pressure with Death in Elders: Health and Retirement Study. J Am Geriatr Soc.

[824] Soobiah C, Daly C, Blondal E, Ewusie J, Ho J, Elliott MJ, Yue R, Holroyd-Leduc J, Liu B, Marr S, Basran J, Tricco AC, Hamid J, Straus SE (2017). An evaluation of the comparative effectiveness of geriatrician-led comprehensive geriatric assessment for improving patient and healthcare system outcomes for older adults: a protocol for a systematic review and network meta-analysis. Syst Rev.

[825] Rockwood K, Song X, MacKnight C (2005). A global clinical measure of fitness and frailty in elderly people. CMAJ.

[826] Rockwood K, Song X, Mitnitski A (2011). Changes in relative fitness and frailty across the adult lifespan: evidence from the Canadian National Population Health Survey. CMAJ.

[827] Afilalo J, Eisenberg MJ, Morin JF (2010). Gait speed as an incremental predictor of mortality and major morbidity in elderly patients undergoing cardiac surgery. J Am Coll Cardiol.

[828] Studenski S, Perera S, Patel K, Rosano C (2011). Gait speed and survival in older adults. JAMA.

[829] Odden MC, Moran AE, Coxson PG, Peralta CA, Goldman L, Bibbins-Domingo K (2016). Gait Speed as a Guide for Blood Pressure Targets in Older Adults: A Modeling Study. J Am Geriatr Soc.

[830] Rodrigues M.K., Nunes Rodrigues I., Vasconcelos Gomes da Silva D.J. (2020). Clinical Frailty Scale: Translation and Cultural Adaptation Into the Brazilian Portuguese Language. J Frailty Aging.

[831] Darvall JN, Greentree K, Braat MS, Story DA, Lim WK (2019). Contributors to frailty in critical illness: Multi-dimensional analysis of the Clinical Frailty Scale. J Crit Care.

[832] Chong E, Ho E, Baldevarona-Llego J, Chan M, Wu L, Tay L (2017). Frailty and Risk of Adverse Outcomes in Hospitalized Older Adults: A Comparison of Different Frailty Measures. J Am Med Dir Assoc.

[833] Atkins JL, Delgado J, Pilling LC (2019). Impact of Low Cardiovascular Risk Profiles on Geriatric Outcomes: Evidence From 421,000 Participants in Two Cohorts. J Gerontol A Biol Sci Med Sci.

[834] Nadruz W, Kitzman D, Windham BG (2017). Cardiovascular Dysfunction and Frailty Among Older Adults in the Community: The ARIC Study. J Gerontol A Biol Sci Med Sci.

[835] Aprahamian I, Sassaki E, dos Santos MF (2018). Hypertension and frailty in older adults. J Clin Hypertens.

[836] Ravindrarajah R, Hazra NC, Hamada S (2017). Systolic Blood Pressure Trajectory, Frailty, and All-Cause Mortality >80 Years of Age: Cohort Study Using Electronic Health Records. Circulation.

[837] Gulla C, Flo E, Kjome RL, Husebo BS (2018). Deprescribing antihypertensive treatment in nursing home patients and the effect on blood pressure. J Geriatr Cardiol.

[838] Warwick J, Falaschetti E, Rockwood K (2015). No evidence that frailty modifies the positive impact of antihypertensive treatment in very elderly people: an investigation of the impact of frailty upon treatment effect in the HYpertension in the Very Elderly Trial (HYVET) study, a double-blind, placebo-controlled study of antihypertensives in people with hypertension aged 80 and over. BMC Med.

[839] Russo G., Liguori I., Aran L. (2018). Impact of SPRINT results on hypertension guidelines: implications for “frail” elderly patients. J Hum Hypertens.

[840] Scheltens P, Blennow K, Breteler MM (2016). Alzheimer’s disease. Lancet.

[841] Abell JG, Kivimäki M, Dugravot A (2018). Association between systolic blood pressure and dementia in the Whitehall II cohort study: role of age, duration, and threshold used to define hypertension. Eur Heart J.

[842] Gottesman RF, Schneider AL, Albert M, Alonso A, Bandeen-Roche K, Coker L, Coresh J, Knopman D, Power MC, Rawlings A, Sharrett AR, Wruck LM, Mosley TH (2014). Midlife hypertension and 20-year cognitive change: the atherosclerosis risk in communities neurocognitive study. JAMA Neurol.

[843] Iadecola C, Gottesman RF (2019). Neurovascular and Cognitive Dysfunction in Hypertension. Circ Res.

[844] Ngandu T, Lehtisalo J, Solomon A (2015). A 2 year multidomain intervention of diet, exercise, cognitive training, and vascular risk monitoring versus control to prevent cognitive decline in at-risk elderly people (FINGER): a randomised controlled trial. Lancet.

[845] Flores LM, Mengue SS (2005). Drug use by the elderly in Southern Brazil. Rev Saúde Pública.

[846] Fulton MM, Allen ER (2005). Polypharmacy in the elderly: a literature review. J Am Acad Nurse Pract.

[847] Gellad WF, Grenard JL, Marcum ZA (2011). A systematic review of barriers to medication adherence in the elderly: looking beyond cost and regimen complexity. Am J Geriatr Pharmacother.

[848] Scott IA, Hilmer SN, Reeve E (2015). Reducing inappropriate polypharmacy: the process of deprescribing. JAMA Intern Med.

[849] Moonen JE, Foster-Dingley JC, Ruijter W (2015). Effect of Discontinuation of Antihypertensive Treatment in Elderly People on Cognitive Functioning--the DANTE Study Leiden: A Randomized Clinical Trial. JAMA Intern Med.

[850] Luymes CH, Poortvliet RKE, van Geloven N (2018). Deprescribing preventive cardiovascular medication in patients with predicted low cardiovascular disease risk in general practice - the ECSTATIC study: a cluster randomised non-inferiority trial. BMC Med.

[851] Gibbons CH, Schmidt P, Biaggioni I (2017). The recommendations of a consensus panel for the screening, diagnosis, and treatment of neurogenic orthostatic hypotension and associated supine hypertension. J Neurol.

[852] Rutan GH, Hermanson B, Bild DE, Kittner SJ, LaBaw F, Tell GS (1992). Orthostatic hypotension in older adults. The Cardiovascular Health Study. Hypertension.

[853] Jansen RW, Lipsitz LA (1995). Postprandial hypotension: epidemiology, pathophysiology, and clinical management. Ann Intern Med.

[854] Gangavati A, Hajjar I, Quach L (2011). Hypertension, orthostatic hypotension, and the risk of falls in a community-dwelling elderly population: the maintenance of balance, independent living, intellect, and zest in the elderly of Boston study. J Am Geriatr Soc..

[855] Margolis KL, Palermo L, Vittinghoff E (2014). Intensive blood pressure control, falls, and fractures in patients with type 2 diabetes: the ACCORD trial. J Gen Intern Med.

[856] Juraschek SP, Taylor AA, Wright JT (2020). Orthostatic Hypotension, Cardiovascular Outcomes, and Adverse Events: Results From SPRINT. Hypertension.

[857] Hirsch AT, Haskal ZJ, Hertzer NR, Bakal CW, Creager MA, Halperin JL (2006). ACC/AHA 2005 guidelines for the management of patients with peripheral arterial disease (lower extremity, renal, mesenteric, and abdominal aortic): executive summary a collaborative report from the American Association for Vascular Surgery/Society for Vascular Surgery, Society for Cardiovascular Angiography and Interventions, Society for Vascular Medicine and Biology, Society of Interventional Radiology, and the ACC/AHA Task Force on Practice Guidelines (Writing Committee to Develop Guidelines for the Management of Patients With Peripheral Arterial Disease) endorsed by the American Association of Cardiovascular and Pulmonary Rehabilitation; National Heart, Lung, and Blood Institute; Society for Vascular Nursing; TransAtlantic Inter-Society Consensus; and Vascular Disease Foundation. J Am Coll Cardiol.

[858] Charles L, Triscott J, Dobbs B (2017). Secondary Hypertension: Discovering the Underlying Cause. Am Fam Physician.

[859] Hirsch JS, Hong S (2019). The Demystification of Secondary Hypertension: Diagnostic Strategies and Treatment Algorithms. Current Treatment Options in Cardiovascular Medicine.

[860] Siddiqui MA, Mittal PK, Little BP, Miller FH, Akduman EI, Ali K (2019). Secondary Hypertension and Complications: Diagnosis and Role of Imaging. Radiographics : a review publication of the Radiological Society of North America, Inc..

[861] Cingolani OH (2019). Cardiovascular Risks and Organ Damage in Secondary Hypertension. Endocrinol Metab Clin North Am.

[862] (2013). Summary of Recommendation Statements. Kidney Int.

[863] Pugh D, Gallacher PJ, Dhaun N (2019). Management of Hypertension in Chronic Kidney Disease. Drugs.

[864] Sinha AD, Agarwal R (2019). Clinical Pharmacology of Antihypertensive Therapy for the Treatment of Hypertension in CKD. Clinical Journal of the American Society of Nephrology.

[865] (2013). Chapter 2: Definition, identification, and prediction of CKD progression. Kidney Int.

[866] Herrmann SM, Textor SC (2018). Current Concepts in the Treatment of Renovascular Hypertension. Am J Hypertens.

[867] Aboyans V, Ricco JB, Bartelink MEL, Bjorck M, Brodmann M, Cohnert T (2018). 2017 ESC Guidelines on the Diagnosis and Treatment of Peripheral Arterial Diseases, in collaboration with the European Society for Vascular Surgery (ESVS): Document covering atherosclerotic disease of extracranial carotid and vertebral, mesenteric, renal, upper and lower extremity arteriesEndorsed by: the European Stroke Organization (ESO)The Task Force for the Diagnosis and Treatment of Peripheral Arterial Diseases of the European Society of Cardiology (ESC) and of the European Society for Vascular Surgery (ESVS). Eur Heart J.

[868] Anderson JL, Halperin JL, Albert NM, Bozkurt B, Brindis RG, Curtis LH (2013). Management of patients with peripheral artery disease (compilation of 2005 and 2011 ACCF/AHA guideline recommendations): a report of the American College of Cardiology Foundation/American Heart Association Task Force on Practice Guidelines. Circulation.

[869] Parikh SA, Shishehbor MH, Gray BH, White CJ, Jaff MR (2014). SCAI expert consensus statement for renal artery stenting appropriate use. Catheter Cardiovasc Interv.

[870] Harvin HJ, Verma N, Nikolaidis P, Hanley M, Dogra VS, Goldfarb S (2017). ACR Appropriateness Criteria((R)) Renovascular Hypertension. Journal of the American College of Radiology.

[871] Rountas C, Vlychou M, Vassiou K, Liakopoulos V, Kapsalaki E, Koukoulis G (2007). Imaging Modalities for Renal Artery Stenosis in Suspected Renovascular Hypertension: Prospective Intraindividual Comparison of Color Doppler US, CT Angiography, GD-Enhanced MR Angiography, and Digital Substraction Angiography. Renal Failure.

[872] Zeller T, Krankenberg H, Erglis A, Blessing E, Fuss T, Scheinert D (2017). A randomized, multi-center, prospective study comparing best medical treatment versus best medical treatment plus renal artery stenting in patients with hemodynamically relevant atherosclerotic renal artery stenosis (RADAR) – one-year results of a pre-maturely terminated study. Trials.

[873] Raman G, Adam GP, Halladay CW, Langberg VN, Azodo IA, Balk EM (2016). Comparative Effectiveness of Management Strategies for Renal Artery Stenosis: An Updated Systematic Review. Ann Intern Med.

[874] Cooper CJ, Murphy TP, Cutlip DE, Jamerson K, Henrich W, Reid DM (2014). Stenting and medical therapy for atherosclerotic renal-artery stenosis. N Engl J Med.

[875] Wheatley K, Ives N, Gray R, Kalra PA, Moss JG, Baigent C (2009). Revascularization versus medical therapy for renal-artery stenosis. N Engl J Med.

[876] Nicholson J, Alderman M, Pickering T, Teichman S, Sos T, Laragh J (1983). CIGARETTE SMOKING AND RENOVASCULAR HYPERTENSION. The Lancet.

[877] Piaggio D, Bracale U, Pecchia L, Di Taranto MD, Sodo M, Bracale UM (2019). Endovascular Treatment versus Medical Therapy for Hypertensive Patients with Renal Artery Stenosis: An Updated Systematic Review. Annals of vascular surgery.

[878] Noory E, Sritharan K, Zeller T (2016). To Stent or Not to Stent? Update on Revascularization for Atherosclerotic Renovascular Disease. Curr Hypertens Rep.

[879] Gornik HL, Persu A, Adlam D, Aparicio LS, Azizi M, Boulanger M (2019). First International Consensus on the diagnosis and management of fibromuscular dysplasia. Vascular medicine (London, England)..

[880] Bolen MA, Brinza E, Renapurkar RD, Kim ESH, Gornik HL (2017). Screening CT Angiography of the Aorta, Visceral Branch Vessels, and Pelvic Arteries in Fibromuscular Dysplasia. JACC Cardiovascular imaging.

[881] Weinberg I, Gu X, Giri J, Kim SE, Bacharach MJ, Gray BH (2015). Anti-platelet and anti-hypertension medication use in patients with fibromuscular dysplasia: Results from the United States Registry for Fibromuscular Dysplasia. Vascular medicine (London, England)..

[882] Rao PS (1995). Coarctation of the aorta. Seminars in nephrology.

[883] Godart F (2007). Management of aortic coarctation at the adult age. Archives des maladies du coeur et des vaisseaux.

[884] Dijkema EJ, Leiner T, Grotenhuis HB (2017). Diagnosis, imaging and clinical management of aortic coarctation. Heart.

[885] Torok RD, Campbell MJ, Fleming GA, Hill KD (2015). Coarctation of the aorta: Management from infancy to adulthood. World journal of cardiology.

[886] Martins JD, Zachariah J, Selamet Tierney ES, Truong U, Morris SA, Kutty S (2019). Impact of Treatment Modality on Vascular Function in Coarctation of the Aorta: The LOVE - COARCT Study. J Am Heart Assoc.

[887] Cangussú LR, Lopes MR, RHdA Barbosa (2019). The importance of the early diagnosis of aorta coarctation. Revista da Associação Médica Brazileira.

[888] Gottlieb DJ, Punjabi NM (2020). Diagnosis and Management of Obstructive Sleep Apnea: A Review. JAMA.

[889] Drager LF, Togeiro SM, Polotsky VY, Lorenzi G (2013). Obstructive sleep apnea: a cardiometabolic risk in obesity and the metabolic syndrome. J Am Coll Cardiol.

[890] Tufik S, Santos-Silva R, Taddei JA, Bittencourt LR (2010). Obstructive sleep apnea syndrome in the Sao Paulo Epidemiologic Sleep Study. Sleep Med.

[891] Fletcher EC, DeBehnke RD, Lovoi MS, Gorin AB (1985). Undiagnosed sleep apnea in patients with essential hypertension. Ann Intern Med.

[892] Williams AJ, Houston D, Finberg S, Lam C, Kinney JL, Santiago S (1985). Sleep apnea syndrome and essential hypertension. Am J Cardiol.

[893] Sjostrom C, Lindberg E, Elmasry A, Hagg A, Svardsudd K, Janson C (2002). Prevalence of sleep apnoea and snoring in hypertensive men: a population based study. Thorax.

[894] Drager LF, Genta PR, Pedrosa RP, Nerbass FB, Gonzaga CC, Krieger EM (2010). Characteristics and predictors of obstructive sleep apnea in patients with systemic hypertension. Am J Cardiol.

[895] Pedrosa RP, Drager LF, Gonzaga CC, Sousa MG, Paula LK, Amaro AC (2011). Obstructive sleep apnea: the most common secondary cause of hypertension associated with resistant hypertension. Hypertension.

[896] Peppard PE, Young T, Palta M, Skatrud J (2000). Prospective study of the association between sleep-disordered breathing and hypertension. N Engl J Med.

[897] Marin JM, Agusti A, Villar I, Forner M, Nieto D, Carrizo SJ (2012). Association between treated and untreated obstructive sleep apnea and risk of hypertension. JAMA.

[898] Drager LF, Bortolotto LA, Figueiredo AC, Silva BC, Krieger EM, Lorenzi G (2007). Obstructive sleep apnea, hypertension, and their interaction on arterial stiffness and heart remodeling. Chest.

[899] Drager LF, Bortolotto LA, Krieger EM, Lorenzi G (2009). Additive effects of obstructive sleep apnea and hypertension on early markers of carotid atherosclerosis. Hypertension.

[900] Gus M, Goncalves SC, Martinez D, Abreu Silva EO, Moreira LB, Fuchs SC (2008). Risk for Obstructive Sleep Apnea by Berlin Questionnaire, but not daytime sleepiness, is associated with resistant hypertension: a case-control study. Am J Hypertens.

[901] Netzer NC, Stoohs RA, Netzer CM, Clark K, Strohl KP (1999). Using the Berlin Questionnaire to identify patients at risk for the sleep apnea syndrome. Ann Intern Med.

[902] Margallo VS, Muxfeldt ES, Guimaraes GM, Salles GF (2014). Diagnostic accuracy of the Berlin questionnaire in detecting obstructive sleep apnea in patients with resistant hypertension. J Hypertens.

[903] Giampa SQC, Pedrosa RP, Gonzaga CC, Bertolami A, Amodeo C, Furlan SF (2018). Performance of NoSAS score versus Berlin questionnaire for screening obstructive sleep apnoea in patients with resistant hypertension. J Hum Hypertens.

[904] Genta-Pereira DC, Furlan SF, Omote DQ, Giorgi DMA, Bortolotto LA, Lorenzi G (2018). Nondipping Blood Pressure Patterns Predict Obstructive Sleep Apnea in Patients Undergoing Ambulatory Blood Pressure Monitoring. Hypertension.

[905] Drager LF, Lorenzi G, Cintra FD, Pedrosa RP, Bittencourt LRA, Poyares D (2018). Arq Bras Cardiol.

[906] Fatureto-Borges F, Lorenzi G, Drager LF (2016). Effectiveness of continuous positive airway pressure in lowering blood pressure in patients with obstructive sleep apnea: a critical review of the literature. Integr Blood Press Control.

[907] Pedrosa RP, Drager LF, Paula LKG, Amaro ACS, Bortolotto LA, Lorenzi G (2013). Effects of OSA treatment on BP in patients with resistant hypertension: a randomized trial. Chest.

[908] Martinez-Garcia MA, Capote F, Campos-Rodriguez F, Lloberes P, Diaz de Atauri MJ, Somoza M (2013). Effect of CPAP on blood pressure in patients with obstructive sleep apnea and resistant hypertension: the HIPARCO randomized clinical trial. JAMA.

[909] Oliveira AC, Martinez D, Massierer D, Gus M, Goncalves SC, Ghizzoni F (2014). The antihypertensive effect of positive airway pressure on resistant hypertension of patients with obstructive sleep apnea: a randomized, double-blind, clinical trial. Am J Respir Crit Care Med.

[910] Castro-Grattoni AL, Torres G, Martínez-Alonso M, Barbé F, Turino C, Sánchez-de-la-Torre A (2017). Blood pressure response to CPAP treatment in subjects with obstructive sleep apnoea: the predictive value of 24-h ambulatory blood pressure monitoring. Eur Respir J.

[911] Montesi SB, Edwards BA, Malhotra A, Bakker JP (2012). The effect of continuous positive airway pressure treatment on blood pressure: a systematic review and meta-analysis of randomized controlled trials. J Clin Sleep Med.

[912] Pepin JL, Tamisier R, Barone-Rochette G, Launois SH, Levy P, Baguet JP (2010). Comparison of continuous positive airway pressure and valsartan in hypertensive patients with sleep apnea. Am J Respir Crit Care Med.

[913] Yumino D, Redolfi S, Ruttanaumpawan P, Su MC, Smith S, Newton GE (2010). Nocturnal rostral fluid shift: a unifying concept for the pathogenesis of obstructive and central sleep apnea in men with heart failure. Circulation.

[914] Gaddam K, Pimenta E, Thomas SJ, Cofield SS, Oparil S, Harding SM (2010). Spironolactone reduces severity of obstructive sleep apnoea in patients with resistant hypertension: a preliminary report. J Hum Hypertens.

[915] Fiori CZ, Martinez D, Montanari CC, Lopez P, Camargo R, Sezera L (2018). Diuretic or sodium-restricted diet for obstructive sleep apnea-a randomized trial. Sleep.

[916] Conn JW (1955). Primary aldosteronism. J Lab Clin Med.

[917] Calhoun DA (2007). Is there an unrecognized epidemic of primary aldosteronism? Pro. Hypertension.

[918] Kline GA, Prebtani APH, Leung AA, Schiffrin EL (2017). Primary aldosteronism: a common cause of resistant hypertension. CMAJ.

[919] Gordon RD, Ziesak MD, Tunny TJ, Stowasser M, Klemm SA (1993). Evidence that primary aldosteronism may not be uncommon: 12% incidence among antihypertensive drug trial volunteers. Clin Exp Pharmacol Physiol.

[920] Funder JW, Carey RM, Mantero F, Murad MH, Reincke M, Shibata H (2016). The Management of Primary Aldosteronism: Case Detection, Diagnosis, and Treatment: An Endocrine Society Clinical Practice Guideline. J Clin Endocrinol Metab.

[921] Morera J, Reznik Y (2018). MANAGEMENT OF ENDOCRINE DISEASE: The role of confirmatory tests in the diagnosis of primary aldosteronism. Eur J Endocrinol.

[922] Stowasser M, Ahmed AH, Cowley D, Wolley M, Guo Z, McWhinney BC (2018). Comparison of Seated With Recumbent Saline Suppression Testing for the Diagnosis of Primary Aldosteronism.

[923] Nanba K, Tamanaha T, Nakao K, Kawashima ST, Usui T, Tagami T (2012). Confirmatory testing in primary aldosteronism. The Journal of clinical endocrinology and metabolism.

[924] Vilela LAP, Almeida MQ (2017). Diagnosis and management of primary aldosteronism. Arch Endocrinol Metab.

[925] Hundemer GL, Curhan GC, Yozamp N, Wang M, Vaidya A (2018). Cardiometabolic outcomes and mortality in medically treated primary aldosteronism: a retrospective cohort study. Lancet Diabetes Endocrinol.

[926] van Berkel A, Lenders JW, Timmers HJ (2014). Diagnosis of endocrine disease: Biochemical diagnosis of phaeochromocytoma and paraganglioma. European journal of endocrinology.

[927] Martucci VL, Pacak K (2014). Pheochromocytoma and paraganglioma: diagnosis, genetics, management, and treatment. Current problems in cancer.

[928] Lenders JW, Pacak K, Walther MM, Linehan WM, Mannelli M, Friberg P (2002). Biochemical diagnosis of pheochromocytoma: which test is best?. Jama.

[929] Tsirlin A, Oo Y, Sharma R, Kansara A, Gliwa A, Banerji MA (2014). Pheochromocytoma: a review. Maturitas.

[930] Pacak K, Eisenhofer G, Ahlman H, Bornstein SR, Gimenez-Roqueplo AP, Grossman AB (2007). Pheochromocytoma: recommendations for clinical practice from the First International Symposium. October 2005. Nature clinical practice Endocrinology & metabolism.

[931] Calabrò D, Allegri V, Fanti S, Ambrosini V (2019). 68Ga-DOTANOC and 18F-DOPA PET/CT: a site-specific approach to the imaging of parangliomas of the head and neck and of the abdomen. European Journal of Nuclear Medicine and Molecular Imaging.

[932] Bravo EL (2002). Pheochromocytoma: an approach to antihypertensive management. Annals of the New York Academy of Sciences.

[933] Jonklaas J, Bianco AC, Bauer AJ, Burman KD, Cappola AR, Celi FS (2014). Guidelines for the treatment of hypothyroidism: prepared by the american thyroid association task force on thyroid hormone replacement. Thyroid.

[934] Jian WX, Jin J, Qin L, Fang WJ, Chen XR, Chen HB (2013). Relationship between thyroid-stimulating hormone and blood pressure in the middle-aged and elderly population. Singapore Med J.

[935] Klein I, Ojamaa K (1994). Thyroid hormone and the cardiovascular system: from theory to practice. J Clin Endocrinol Metab.

[936] Frost L, Vestergaard P, Mosekilde L (2004). Hyperthyroidism and risk of atrial fibrillation or flutter: a population-based study. Arch Intern Med.

[937] Ross DS, Burch HB, Cooper DS, Greenlee MC, Laurberg P, Maia AL (2016). 2016 American Thyroid Association Guidelines for Diagnosis and Management of Hyperthyroidism and Other Causes of Thyrotoxicosis. Thyroid.

[938] Richards AM, Espiner EA, Nicholls MG, Ikram H, Hamilton EJ, Maslowski AH (1988). Hormone, calcium and blood pressure relationships in primary hyperparathyroidism. J Hypertens.

[939] Bilezikian JP (2018). Primary Hyperparathyroidism. The Journal of clinical endocrinology and metabolism.

[940] Nieman LK, Biller BMK, Findling JW, Murad MH, Newell-Price J, Savage MO (2015). Treatment of Cushing’s Syndrome: An Endocrine Society Clinical Practice Guideline. The Journal of Clinical Endocrinology & Metabolism.

[941] Fantin F, Giani A, Zoico E, Rossi AP, Mazzali G, Zamboni M (2019). Weight Loss and Hypertension in Obese Subjects. Nutrients.

[942] Saliba LJ, Maffett S (2019). Hypertensive Heart Disease and Obesity: A Review. Heart Fail Clin.

[943] Seravalle G, Grassi G (2017). Obesity and hypertension. Pharmacol Res.

[944] Ping Z, Pei X, Xia P, Chen Y, Guo R, Hu C (2018). Anthropometric indices as surrogates for estimating abdominal visceral and subcutaneous adipose tissue: A meta-analysis with 16,129 participants. Diabetes research and clinical practice.

[945] Nimptsch K, Konigorski S, Pischon T (2019). Diagnosis of obesity and use of obesity biomarkers in science and clinical medicine. Metabolism.

[946] Katznelson L, Atkinson J, Cook D, Ezzat S, Hamrahian A, Miller K (2011). American Association of Clinical Endocrinologists Medical Guidelines for Clinical Practice for the Diagnosis and Treatment of Acromegaly-2011 Update. Endocrine Practice.

[947] Pablos-Velasco P, Venegas EM, Alvarez Escola C, Fajardo C, Miguel P, Gonzalez N (2020). Diagnosis, treatment and follow-up of patients with acromegaly in a clinical practice setting in Spain: the ACROPRAXIS program Delphi survey. Pituitary.

[948] Silverstein JM, Roe ED, Munir KM, Fox JL, Emir B, Kouznetsova M (2018). Use of electronic health records to characteristize a rare disease in the U.S. : treatment comorbidities and follow-up trends among patients with a confirmed diagnosis of acromegaly. Endocr Pract.

[949] Brazil, Ministério daSaúde, Secretaria da Ciência e Tecnologia e Insumos Estratégicos (2019). Portaria Conjunta n.2, de 07 de janeiro de 2019. Aprova o protocolo clínico e diretrizes terapêuticas da Acromeagalia.

[950] Grossman E, Messerli FH (2012). Drug-induced hypertension: an unappreciated cause of secondary hypertension. Am J Med.

[951] Diaconu CC, Dediu GN, Iancu MA (2018). Drug-induced arterial hypertension - a frequently ignored cause of secondary hypertension: a review. Acta cardiologica.

[952] Versmissen J, Mirabito Colafella KM, Koolen SLW, Danser AHJ (2019). Vascular Cardio-Oncology: Vascular Endothelial Growth Factor inhibitors and hypertension. Cardiovasc Res.

[953] Rizzoni D, Ciuceis C, Porteri E, Agabiti-Rosei C, Agabiti-Rosei E (2017). Use of Antihypertensive Drugs in Neoplastic Patients. High Blood Press Cardiovasc Prev.

[954] Carey RM, Calhoun DA, Bakris GL, Brook RB, Daugherty SL, Dennison-Himmelfarb CR (2018). Resistant hypertension: detection, evaluation, and management: a scientific statement from the American Heart Association. Hypertension.

[955] Calhoun DA, Booth JN, Oparil S, Irvin MR, Shimbo D, Lackland DT (2014). Refractory hypertension: determination of prevalence, risk factors, and comorbidities in a large, population-based cohort. Hypertension.

[956] (2014). Resistant hypertension: patient characteristics, risk factors, co-morbidities and outcomes. J Hum Hypertens.

[957] Gaddam KK, Nishizaka MK, Pratt-Ubunama MN, Pimenta E, Aban I, Oparil S (2008). Characterization of resistant hypertension: association between resistant hypertension, aldosterone, and persistent intravascular volume expansion. Arch Intern Med.

[958] Shimosawa T (2013). Salt, the renin-angiotensin-aldosterone system and resistant hypertension. Hypertens Res.

[959] Calhoun DA (2016). Refractory and Resistant Hypertension: Antihypertensive Treatment Failure versus Treatment Resistance. Korean Circ J.

[960] Eirin A, Textor SC, Lerman LO (2016). Emerging concepts for patients with treatment-resistant hypertension. Trends Cardiovasc Med.

[961] Judd EK, Calhoun DA, Warnock DG (2014). Pathophysiology and treatment of resistant hypertension: the role of aldosterone and amiloride-sensitive sodium channels. Semin Nephrol.

[962] Dudenbostel T, Acelajado MC, Pisoni R, Li P, Oparil S, Calhoun DA (2015). Refractory hypertension: evidence of heightened sympathetic activity as a cause of antihypertensive treatment failure. Hypertension.

[963] Barbaro NR, Araújo TM, Tanus-Santos JE, Anhê GF, Fontana V, Moreno H (2015). Vascular Damage in Resistant Hypertension: TNF-Alpha Inhibition Effects on Endothelial Cells. Biomed Res Int.

[964] Barbaro NR, Fontana V, Modolo R, Faria AP, Sabbatini AR, Fonseca FH (2015). Increased arterial stiffness in resistant hypertension is associated with inflammatory biomarkers. Blood Press.

[965] Yugar-Toledo JC, Martin JF, Krieger JE, Pereira AC, Demacq C, Coelho OR (2011). Gene variation in resistant hypertension: multilocus analysis of the angiotensin 1-converting enzyme, angiotensinogen, and endothelial nitric oxide synthase genes. DNA Cell Biol.

[966] Aronow WS (2020). Approaches for the management of resistant hypertension in 2020. Curr Hypertens Rep.

[967] Corrêa NB, Faria AP, Ritter AM, Sabbatini AR, Almeida A, Brunelli V (2016). A practical approach for measurement of antihypertensive medication adherence in patients with resistant hypertension. J Am Soc Hypertens.

[968] Lazaridis AA, Sarafidis PA, Ruilope LM (2015). Ambulatory Blood Pressure Monitoring in the Diagnosis, Prognosis, and Management of Resistant Hypertension: Still a Matter of our Resistance?. Curr Hypertens Rep.

[969] Muxfeldt ES, Salles GF (2013). How to use ambulatory blood pressure monitoring in resistant hypertension. Hypertens Res.

[970] Muxfeldt ES, Barros GS, Viegas BB, Carlos FO, Salles GF (2015). Is home blood pressure monitoring useful in the management of patients with resistant hypertension?. Am J Hypertens.

[971] Ozemek C, Tiwari S, Sabbahi A, Carbone S, Lavie CJ (2020). Impact of therapeutic lifestyle changes in resistant hypertension. Prog Cardiovasc Dis.

[972] Acelajado MC, Hughes ZH, Oparil S, Calhoun DA (2019). Treatment of resistant and refractory hypertension. Circ Res.

[973] Williams B, MacDonald TM, Morant SV, Webb DJ, Sever P, McInnes GT (2018). Endocrine and haemodynamic changes in resistant hypertension, and blood pressure responses to spironolactone or amiloride: the PATHWAY-2 mechanisms substudies. Lancet . Diabetes Endocrinol..

[974] Hermida RC, Crespo JJ, Domínguez-Sardiña M, Otero A, Moyá A, Ríos MT (2019). Bedtime hypertension treatment improves cardiovascular risk reduction: the Hygia Chronotherapy Trial. Eur Heart J.

[975] Berra E, Azizi M, Capron A, Høieggen A, Rabbia F, Kjeldsen SE (2016). Evaluation of adherence should become an integral part of assessment of patients with apparently treatment-resistant hypertension. Hypertension.

[976] Kunz M, Lauder L, Ewen S, Böhm M, Mahfoud F (2020). The current status of devices for the treatment of resistant hypertension. Am J Hypertens.

[977] Muxfeldt ES, Chedier B, Rodrigues CIS (2019). Hipertensão resistente e refratária: duas faces de uma mesma doença?. J Bras Nefrol.

[978] Alessi A, Brandao AA, Coca A, Cordeiro AC, Nogueira AR, Diogenes de Magalhaes F (2012). First Brazilian position on resistant hypertension. Arq Bras Cardiol.

[979] Geldsetzer P, Manne-Goehler J, Marcus ME (2019). The state of hypertension care in 44 low-income and middle-income countries: a cross- sectional study of nationally representative individual-level data from 1·1 million adults. Lancet.

[980] Picon RV, Dias-da-Costa JS, Fuchs FD, Olinto MTA, Choudhry NK, Fuchs SC (2017). Hypertension Management in Brazil: Usual Practice in Primary Care. A Meta-Analysis. Int J Hypertens.

[981] World Health Organization (WHO). (2003). Adherence to long-term therapies: evidence for action.

[982] Vrijens B, Geest S, Hughes DA, Przemyslaw K, Demonceau J, Ruppar T, ABC Project Team (2012). A new taxonomy for describing and defining adherence to medications. Br J Clin Pharmacol.

[983] Haynes RB, Sackett DL, Gibson ES, Taylor DW, Hackett BC, Roberts RS (1976). Improvement of medication compliance in uncontrolled hypertension. Lancet.

[984] Gialamas A, Yelland LN, Ryan P, Willson K, Laurence CO, Bubner TK (2009). Does point-of-care testing lead to the same or better adherence to medication? A randomised controlled trial: the PoCT in General Practice Trial. Med J Austr.

[985] Naderi SH, Bestwick JP, Wald DS (2012). Adherence to drugs that prevent cardiovascular disease: meta-analysis on 376,162 patients. Am J Med.

[986] Gupta P, Patel P, Horne R, Buchanan H, Williams B, Tomaszewski M (2016). How to Screen for Non-Adherence to Antihypertensive Therapy. Curr Hypertens Rep.

[987] Morisky DE, Ang A, Krousel-Wood M, Ward HJ (2008). Predictive validity of a medication adherence measure in an outpatient setting. J Clin Hypertens.

[988] Morisky DE, Green LW, Levine DM (1986). Concurrent and predictive validity of a self-reported measure of medication adherence.

[989] Oliveira AD, Morisky DE, Neves SJF, Costa FA, Lyra DP (2014). The 8-item Morisky Medication Adherence Scale: Validation of a Brazilian–Portuguese version in hypertensive adults. Res Social Adm Pharm.

[990] Santa Helena ET, Nemes MIB, Eluf J (2008). Desenvolvimento e validação de questionário multidimensional para medir não-adesão ao tratamento com medicamentos.

[991] Burnier M, Egan BM (2019). Adherence in Hypertension A Review of Prevalence, Risk Factors, Impact, and Management. Circ Res.

[992] Osterberg L, Blaschke T (2005). Adherence to medication. N Engl J Med.

[993] Dhar L, Dantas J, Ali M (2017). A systematic review of factors influencing medication adherence to hypertension treatment in developing countries. Open J Epidemiol.

[994] Abegaz TM, Shehab A, Gebreyohanne EA (2017). Nonadherence to Antihypertensive Drugs: A Systematic Review Meta-Analysis. Medicine.

[995] Glynn LG, Murphy AW, Smith SM, Schroeder K, Fahey T (2010). Interventions used to improve control of blood pressure in patients with hypertension. Cochrane Database Syst Rev.

[996] van der Laan DM, Elders PJM, Boons CCLM, Beckeringh JJ, Nijpels G, Hugtenburg JG (2017). Factors associated with antihypertensive medication non-adherence: a systematic review. J Hum Hypertens.

[997] Gupta AK, Arshad S, Poulter NR (2010). Compliance, safety, and effectiveness of fixed-dose combinations of antihypertensive agents: a meta-analysis. Hypertension.

[998] Santschi V, Chiolero A, Colosimo AL, Platt RW, Taff. P, Burnier M, Burnand B, Paradis G. (2014). Improving blood pressure control through pharmacist interventions: a meta-analysis of randomized controlled trials. J Am Heart Assoc.

[999] Rudd P, Miller NH, Kaufman J, Kraemer HC, Bandura A, Greenwald G, Debusk RF (2004). Nurse management for hypertension. A system approaches. Am J Hypertens.

[1000] Checchi KD, Huybrechts KF, Avorn J, Kesselheim AS (2014). Electronic medication packaging devices and medication adherence: a systematic review. JAMA.

[1001] Burnier M, Wuerzner G, Struijker-Boudier H, Urquhart J (2013). Measuring, analyzing, and managing drug adherence in resistant hypertension. Hypertension.

[1002] Christensen A, Osterberg LG, Hansen EH (2009). Electronic monitoring of patient adherence to oral antihypertensive medical treatment: a systematic review. J Hypertens.

[1003] Parati G, Torlasco C, Omboni S, Pellegrini D (2017). Smartphone applications for hypertension management: a potential game-changer that needs more control. Curr Hypertens Rep.

[1004] Naik AD, Kallen MA, Walder A, Street RL (2008). Improving hypertension control in diabetes mellitus: the effects of collaborative and proactive health communication. Circulation.

[1005] Conn VS, Ruppara TM, Enriqueza M, Cooper P (2016). Medication adherence interventions that target subjects with adherence problems: Systematic Review and Meta-analysis. Res Social Adm Pharm.

[1006] (2017). Medication Adherence Outcomes of 771 Intervention Trials: Systematic Review and Meta-Analysis. Prev Med.

[1007] Mathers CD, Stevens GA, Boerma T, White RA, Tobias MI (2015). Causes of international increases in older age life expectancy. Lancet.

[1008] Ji H, Kim A, Ebinger JE, Niiranen TJ, Claggett BL, Merz NB (2020). Sex Differences in Blood Pressure Trajectories Over the Life Course. JAMA Cardiol.

[1009] Olsen MH, Angell S, Asmar S, Boutouyrie P, Burger D, Chirinos JA (2016). A call to action and a lifecourse strategy to address the global burden of raised blood pressure on current and future generations: The Lancet Commission on hypertension. Lancet.

[1010] Brandão AB, Amodeo C, Alcantara C, Barbosa E, Nobre F (2017). I Luso Brazilian Positioning Paper on Central Arterial Pressure. Arq Bras Cardiol.

[1011] Hamczyk Nevado RM, Barettino A, Fuster V, Andrés V (2020). Biological Versus Chronological Aging. J Am Coll Cardiol.

[1012] Sugiura T, Takase H, Machii M, Nonaka D, Ohno K (2020). Central blood pressure predicts the development of hypertension in the general population. Hypertens Res.

[1013] Matsuzawa Y, Kwon TG, Lennon RJ, Lerman LO, Lerman A (2015). Prognostic Value of Flow-Mediated Vasodilation in Brachial Artery and Fingertip Artery for Cardiovascular Events: A Systematic Review and Meta-Analysis. J Am Heart Assoc.

[1014] Wu MY, Li CJ, Hou MF, Chu PY (2017). New Insights into the Role of Inflammation in the Pathogenesis of Atherosclerosis. Int J Mol Sci.

[1015] Grossman C, Levin M, Koren-Morag N, Bornstein G, Leibowitz A, Ben-Zvi I (2018). Left Ventricular Hypertrophy Predicts Cardiovascular Events in Hypertensive Patients with Coronary Artery Calcifications. Am J Hypertens.

[1016] McEvoy JW, Martin SS, Dardari ZA, Miedema MD, Sandfort V, Yeboah J (2017). Coronary artery calcium to guide a personalized risk-based approach to initiation and intensification of antihypertensive therapy. Circulation.

[1017] Kerkelä R, Ulvila J, Magga J (2015). Natriuretic Peptides in the Regulation of Cardiovascular Physiology and Metabolic Events. J Am Heart Assoc.

[1018] Willeit P, Welsh P, Evans JDW, Tschiderer I, Boachie C, Jukema JW (2017). High-Sensitivity Cardiac Troponin Concentration and Risk of First-Ever Cardiovascular Outcomes in 154,052 Participants. J Am Coll Cardiol.

[1019] Pandey A, Patel KV, Vongpatanasin W, c Ayers, Berritz JD, Mentz RJ (2019). Incorporation of Biomarkers Into Risk Assessment for Allocation of Antihypertensive Medication According to the 2017 ACC/AHA High Blood Pressure Guideline: A Pooled Cohort Analysis. Circulation.

[1020] Malachias MVB, Jhund PS, Claggett BL, Wijkman MO, Bentley-Lewis R, Chaturvedi N (2020). NT-pro BNP by Itself Predicts death and cardiovascular events in high-risk patients with type 2 Diabetes Mellitus. J Am Heart Assoc.

[1021] lar R, Sánchez R, Boggia R, Peñaherrera, Lopez J, Barroso WS (2020). Recommendations for home blood pressure monitoring in Latin American countries: A Latin American Society of Hypertension position paper. J Clin Hypertens(Greenwich)..

[1022] Shimbo D, Artenian N, Basile JN, Krakoff LR, Margolis K, Rackotz MK (2020). Self-measured blood pressure monitoring at home. A Joint Policy Statement From the American Heart Association and American Medical Association. Circulation.

[1023] Mukherjee R, Ghosh S, Gupta B, Chakravarty T (2018). A Universal Noninvasive Continuous Blood Pressure Measurement System for Remote Healthcare Monitoring. Telemed J E Health..

[1024] Bard DM, Joseph JI, van Helmond N (2019). Cuff-Less Methods for Blood Pressure Telemonitoring. Front Cardiovasc Med.

[1025] Fuchs FD, Whelton PK (2020). High Blood Pressure and Cardiovascular Disease. Hypertension.

[1026] (2016). Arterial stiffness, atherosclerosis and cardiovascular risk: Pathophysiologic mechanisms and emerging clinical indications. Vasc Pharmacol.

[1027] Morales SA, Coca A, Olsen MH, Sanchez RA, Sebba-Barroso WK, Kones R (2017). Clinical Perspective on Antihypertensive Drug Treatment in Adults With Grade 1 Hypertension and Low-to-Moderate Cardiovascular Risk: An International Expert Consultation. Curr Probl Cardiol.

[1028] Feitosa AD, Gomes MM, Passarelli O, Barroso WKS, Miranda RDS, Barbosa EDB (2020). Pharmacological Treatment of Hypertension: From the Golden Trio. Octet Arq Bras Cardiol.

[1029] Campana E, Cunha V, Glaveckaite S, Gruev I, Lamirault G, Lehmann E (2020). The use of single-pill combinations as first-line treatment for hypertension: translating guidelines into clinical practice. J Hypertensi.

[1030] Laurent S, Chatellier G, Azizi M, Calvet D, Choukroun G, Danchin N A Strategy for Preventing Cardiovascular and Renal Events based on Arterial Stiffness. Protocol of the SPARTE Study.

[1031] Barroso WKS, Inuzuka S, Guimarães GC, Pacífico RP, Melo VA, Oliveira LF (2020). Pharmacological Management of Hypertension Guided by Central or Peripheral Blood Pressure Measurement: Comparison of Two Strategies on the Incidence of Intermediate Outcome. Artery Research.

[1032] Arendse LB, Danser AHJ, Poglitsch M, Touyz RM, Burnett JC, Llorens-Cortesc (2019). novel therapeutic approaches targeting the renin-angiotensin system and associated peptides in hypertension and heart failure. Pharmacol Rev.

