# Supplementary material for: Diretrizes Brasileiras de Hipertensão Arterial – 2020
Source: Arq Bras Cardiol. 2021 Mar 3;116(3):516–658. [Article in Portuguese] doi: 10.36660/abc.20201238 (PMC9949730; doi:10.36660/abc.20201238)
Supplement: Translation

# Translation: Brazilian Guidelines of Hypertension – 2020

**Authors:** Weimar Kunz Sebba Barroso, Cibele Isaac Saad Rodrigues, Luiz Aparecido Bortolotto, Marco Antônio Mota-Gomes, Andréa Araujo Brandão, Audes Diógenes de Magalhães Feitosa, Carlos Alberto Machado, Carlos Eduardo Poli-de-Figueiredo, Celso Amodeo, Décio Mion, Eduardo Costa Duarte Barbosa, Fernando Nobre, Isabel Cristina Britto Guimarães, José Fernando Vilela-Martin, Juan Carlos Yugar-Toledo, Maria Eliane Campos Magalhães, Mário Fritsch Toros Neves, Paulo César Brandão Veiga Jardim, Roberto Dischinger Miranda, Rui Manuel dos Santos Póvoa, Sandra C Fuchs, Alexandre Alessi, Alexandre Jorge Gomes de Lucena, Alvaro Avezum, Ana Luiza Lima Sousa, Andrea Pio-Abreu, Andrei Carvalho Sposito, Angela Maria Geraldo Pierin, Annelise Machado Gomes de Paiva, Antonio Carlos de Souza Spinelli, Armando da Rocha Nogueira, Nelson Dinamarco, Bruna Eibel, Cláudia Lúcia de Moraes Forjaz, Claudia Regina de Oliveira Zanini, Cristiane Bueno de Souza, Dilma do Socorro Moraes de Souza, Eduardo Augusto Fernandes Nilson, Elisa Franco de Assis Costa, Elizabete Viana de Freitas, Elizabeth da Rosa Duarte, Elizabeth Silaid Muxfeldt, Emilton Lima, Erika Maria Gonçalves Campana, Evandro José Cesarino, Fabiana Marques, Fábio Argenta, Fernanda Marciano Consolim-Colombo, Fernanda Spadotto Baptista, Fernando Antonio de Almeida, Flávio Antonio de Oliveira Borelli, Flávio Danni Fuchs, Frida Liane Plavnik, Gil Fernando Salles, Gilson Soares Feitosa, Giovanio Vieira da Silva, Grazia Maria Guerra, Heitor Moreno, Helius Carlos Finimundi, Isabela de Carlos Back, João Bosco de Oliveira, João Roberto Gemelli, José Geraldo Mill, José Marcio Ribeiro, Leda A. Daud Lotaif, Lilian Soares da Costa, Lucélia Batista Neves Cunha Magalhães, Luciano Ferreira Drager, Luis Cuadrado Martin, Luiz César Nazário Scala, Madson Q. Almeida, Marcia Maria Godoy Gowdak, Marcia Regina Simas Torres Klein, Marcus Vinícius Bolívar Malachias, Maria Cristina Caetano Kuschnir, Maria Eliete Pinheiro, Mario Henrique Elesbão de Borba, Osni Moreira, Oswaldo Passarelli, Otavio Rizzi Coelho, Priscila Valverde de Oliveira Vitorino, Renault Mattos Ribeiro, Roberto Esporcatte, Roberto Franco, Rodrigo Pedrosa, Rogerio Andrade Mulinari, Rogério Baumgratz de Paula, Rogério Toshiro Passos Okawa, Ronaldo Fernandes Rosa, Sandra Lia do Amaral, Sebastião R. Ferreira-Filho, Sergio Emanuel Kaiser, Thiago de Souza Veiga Jardim, Vanildo Guimarães, Vera H. Koch, Wille Oigman, Wilson Nadruz

**Affiliations:** 1 Brasil Universidade Federal de Goiás, Goiânia, GO – Brazil; 2 Brasil Liga de Hipertensão Arterial, Goiânia, GO – Brazil; 3 Brasil Pontifícia Universidade Católica de São Paulo, Faculdade de Ciências Médicas e da Saúde, Sorocaba, SP – Brazil; 4 Brasil Instituto do Coração (InCor), São Paulo, SP – Brazil; 5 Brasil Centro Universitário CESMAC, Maceió, AL – Brazil; 6 Brasil Faculdade de Ciências Médicas da Universidade do Estado do Rio de Janeiro (FCM-UERJ), Rio de Janeiro, RJ – Brazil; 7 Brasil Universidade Federal de Pernambuco, Recife, PE – Brazil; 8 Brasil Pronto Socorro Cardiológico de Pernambuco (PROCAPE), Recife, PE – Brazil; 9 Brasil Secretaria Municipal de Saúde de Campos do Jordão, Campos do Jordão, SP – Brazil; 10 Brasil Pontifícia Universidade Católica do Rio Grande do Sul, Porto Alegre, RS – Brazil; 11 Brasil Universidade Federal de São Paulo (UNIFESP), São Paulo, SP – Brazil; 12 Brasil Hospital das Clínicas da Faculdade de Medicina da USP, São Paulo, SP – Brazil; 13 Brasil Serviço Hipertensão e Cardiometabolismo da Santa Casa de Porto Alegre, Porto Alegre, RS – Brazil; 14 Brasil Hospital das Clínicas da Faculdade de Medicina de Ribeirão Preto da Universidade de São Paulo, Ribeirão Preto, SP – Brazil; 15 Brasil Hospital São Francisco, Ribeirão Preto, SP – Brazil; 16 Brasil Universidade Federal da Bahia (UFBA), Salvador, BA – Brazil; 17 Brasil Faculdade Estadual de Medicina de São José do Rio Preto, São José do Rio Preto, SP – Brazil; 18 Brasil Hospital Universitário Pedro Ernesto da Universidade do Estado do Rio de Janeiro (UERJ), Rio de Janeiro, RJ – Brazil; 19 Brasil Hospital do Coração de Goiás, Goiânia, GO – Brazil; 20 Brasil Faculdade de Medicina da Universidade Federal do Rio Grande do Sul (UFRGS), Porto Alegre, RS – Brazil; 21 Brasil Universidade Federal do Paraná (UFPR), Curitiba, PR – Brazil; 22 Brasil Hospital Agamenom Magalhães, Recife, PE – Brazil; 23 Brasil Hospital Alemão Oswaldo Cruz, São Paulo, SP – Brazil; 24 Brasil Universidade de São Paulo (USP), São Paulo, SP – Brazil; 25 Brasil Universidade Estadual de Campinas (UNICAMP), Campinas, São Paulo – Brazil; 26 Brasil Cardiocentro, Natal, RN – Brazil; 27 Brasil Universidade Federal do Rio de Janeiro (UFRJ), Rio de Janeiro, RJ – Brazil; 28 Brasil Universidade Estadual de Santa Cruz, Ilhéus, BA – Brazil; 29 Brasil Instituto de Cardiologia, Fundação Universitária de Cardiologia (IC/FUC), Porto Alegre, RS – Brazil; 30 Brasil Centro Universitário da Serra Gaúcha (FSG), Caxias do Sul, RS – Brazil; 31 Brasil Universidade Federal do Pará (UFPA), Belém, PA – Brazil; 32 Brasil Ministério da Saúde, Brasília, DF – Brazil; 33 Brasil Departamento de Cardiogeriatria da Sociedade Brazileira de Cardiologia, Rio de Janeiro, RJ – Brazil; 34 Brasil Hospital Nossa Senhora da Conceição (HNSC), Tubarão, SC – Brazil; 35 Brasil Universidade Estácio de Sá (UNESA), Rio de Janeiro, RJ – Brazil; 36 Brasil Hospital de Clínicas da Universidade Federal do Paraná (HC/UFPR), Curitiba, PR – Brazil; 37 Brasil Universidade Iguaçu (UNIG), Rio de Janeiro, RJ – Brazil; 38 Brasil Faculdade de Ciências Farmacêuticas de Ribeirão Preto da Universidade de São Paulo, Ribeirão Preto, SP – Brazil; 39 Brasil Associação Ribeirãopretana de Ensino, Pesquisa e Assistência ao Hipertenso (AREPAH), Ribeirão Preto, SP – Brazil; 40 Brasil Faculdade de Medicina de Ribeirão Preto da Universidade de São Paulo, Ribeirão Preto, SP – Brazil; 41 Brasil Mediodonto, Cuiabá, MT – Brazil; 42 Brasil Programa de Pós-Graduação em Medicina da Universidade Nove de Julho (UNINOVE), São Paulo, SP – Brazil; 43 Brasil Instituto Dante Pazzanese de Cardiologia, São Paulo, SP – Brazil; 44 Brasil Hospital de Clínicas de Porto Alegre, Porto Alegre, RS – Brazil; 45 Brasil Escola Bahiana de Medicina e Saúde Pública, Salvador, BA – Brazil; 46 Brasil Universidade Santo Amaro (UNISA), São Paulo, SP – Brazil; 47 Brasil Universidade de Caxias do Sul (UCS), Caxias do Sul, RS – Brazil; 48 Brasil Universidade Federal de Santa Catarina (UFSC), Florianópolis, SC – Brazil; 49 Brasil Hospital Israelita Albert Einstein, São Paulo, SP – Brazil; 50 Brasil Clínica Gemelli, Porto Velho, RO – Brazil; 51 Brasil Centro de Ciências da Saúde, Universidade Federal do Espírito Santo, Vitória, ES – Brazil; 52 Brasil Faculdade Ciências Médicas de Minas Gerais, Belo Horizonte, MG – Brazil; 53 Brasil Hospital Felício Rocho, Belo Horizonte, MG – Brazil; 54 Brasil Hospital do Coração (HCor), São Paulo, SP – Brazil; 55 Brasil Instituto Estadual de Cardiologia Aloysio de Castro, Rio de Janeiro, RJ – Brazil; 56 Brasil Centro Universitário de Tecnologia e Ciência (UniFTC), Salvador, BA – Brazil; 57 Brasil Universidade Estadual Paulista (UNESP), Bauru, SP – Brazil; 58 Brasil Faculdade de Medicina da Universidade Federal de Mato Grosso, Cuiabá, MT – Brazil; 59 Brasil Instituto Vera Cruz, São Paulo, SP – Brazil; 60 Brasil Sociedade Brazileira de Nefrologia, São Paulo – Brazil; 61 Brasil Cardio Clínica do Vale, Lajeado, RS – Brazil; 62 Brasil Pontifícia Universidade Católica do Paraná, Curitiba, PR – Brazil; 63 Brasil Pontifícia Universidade Católica de Goiás, Goiânia, GO – Brazil; 64 Brasil Cardios Vita Centro de Medicina Cardiológica, Brasília, DF – Brazil; 65 Brasil Hospital Pró-Cradíaco, Rio de Janeiro, RJ – Brazil; 66 Brasil Universidade Federal de Juiz de Fora, Juiz de Fora, MG – Brazil; 67 Brasil Avancor Cardiologia, Maringá, PR – Brazil; 68 Brasil Universidade Estadual de Maringá, Maringá, PR – Brazil; 69 Brasil Faculdade de Ciências Médicas da Santa Casa de São Paulo, São Paulo – Brazil; 70 Brasil Universidade Federal de Uberlândia, Uberlândia, MG – Brazil; 71 Brasil Hospital Getúlio Vargas, Recife, PE – Brazil

**Table:** 

Declaration of potential conflict of interests of authors/collaborators of the Brazilian Guidelines of Hypertension – 2020							
If, within the last 3 years, the author/collaborator of the guideline:							
Names of guideline collaborators	Participated in clinical and/or experimental studies sponsored by pharmaceutical or equipment companies related to this statement	Spoke at events or activities sponsored by industry related to this statement	Was (is) a member of a board of advisors or a board of directors of a pharmaceutical or equipment industry	Participated in normative committees of scientific research sponsored by industry	Received personal or institutional funding from industry	Wrote scientific papers in journals sponsored by industry	Owns stocks in industry
Alexandre Alessi	No	No	No	No	No	No	No
Alexandre Jorge Gomes de Lucena	No	No	No	No	No	No	No
Alvaro Avezum	No	No	No	No	No	No	No
Ana Luiza Lima Sousa	No	No	No	No	No	No	No
Andréa Araujo Brandão	No	Abbott, Daiichi Sankyo, EMS, Libbs, Novartis, Medley, Merck, Servier	No	No	Servier	Abbott, Daiichi Sankyo, EMS, Libbs, Novartis, Medley, Merck, Servier	No
Andrea Pio-Abreu	No	No	No	No	No	No	No
Andrei Carvalho Sposito	No	No	No	No	No	No	No
Angela Maria Geraldo Pierin	No	No	No	No	No	No	No
Annelise Machado Gomes de Paiva	No	No	No	No	No	No	No
Antonio Carlos de Souza Spinelli	No	Merck, Torrent, Boerhinger, Sandoz	No	No	EMS, Aché, Torrent	No	No
Armando da Rocha Nogueira	No	No	No	No	No	No	No
Audes Diógenes de Magalhães Feitosa	No	EMS, Servier, Sandoz, Merck, Medtronic e Omron	Omron	No	No	EMS, Servier e Omron	No
Bruna Eibel	No	No	No	No	No	No	No
Carlos Alberto Machado	No	No	Biolab, Omron	No	No	No	No
Carlos Eduardo Poli-de-Figueiredo	No	No	No	Fresenius	Centro de Pesquisa Clínico da PUCRS, Baxter, Fresenius, Alexion, AstraZeneca.	No	No
Celso Amodeo	No	Novartis, NovoNordisk, EMS, ACHE	Montecorp Farmasa	No	No	ACHE, Montecorp Farmasa	No
Cibele Isaac Saad Rodrigues	No	No	No	No	No	No	No
Cláudia Lúcia de Moraes Forjaz	No	No	No	No	No	No	No
Claudia Regina de Oliveira Zanini	No	No	No	No	No	No	No
Cristiane Bueno de Souza	No	No	No	No	No	No	No
Decio Mion Junior	No	Zodiac	No	No	No	Zodiac	No
Dilma do Socorro Moraes de Souza	No	No	No	No	No	No	No
Eduardo Augusto Fernandes Nilson	No	No	No	No	No	No	No
Eduardo Costa Duarte Barbosa	No	Servier, EMS	No	No	No	No	No
Elisa Franco de Assis Costa	No	No	No	No	Abbot Nutrition, Nestlé Health Sciences, Aché, Sandoz, Nutricia	Abboot Nutrition	No
Elizabete Viana de Freitas	No	No	No	No	No	No	No
Elizabeth da Rosa Duarte	No	No	No	No	Sim, Ache, Bayer, Novartis, Torrent, Servier.	No	No
Elizabeth Silaid Muxfeldt	No	No	No	No	No	No	No
Emilton Lima Júnior	No	Servier, Novo Nordisk, Bayer, Biolab, Amgem	No	Servier	No	No	No
Erika Maria Gonçalves Campana	No	No	No	No	Servier	Servier	No
Evandro José Cesarino	No	No	No	No	No	No	No
Fábio Argenta	No	No	No	No	Novartis, Bayer, Torrent, Lilly, Boehringer	No	No
Fernanda Marciano Consolim-Colombo	No	Merck, Ache, Daiichi	No	No	Não	No	No
Fernanda Spadotto Baptista	No	No	No	No	Não	No	No
Fernando Antonio de Almeida	No	No	No	No	Não	No	No
Fernando Nobre	No	Libbs, Cristália	No	No	Libbs, Novartis, Servier, Baldacci	Daichi Sankio, Libbs, Novartis, Biolab, Servier, Baldacci	No
Flávio Antonio de Oliveira Borelli	No	No	No	No	No	No	No
Flávio Danni Fuchs	No	No	No	No	No	No	No
Frida Liane Plavnik	No	No	No	No	No	No	No
Gil Fernando Salles	No	No	No	No	No	No	No
Gilson Soares Feitosa	No	No	No	No	No	No	No
Giovanio Vieira da Silva	No	Ache	No	No	No	Ache	No
Grazia Guerra	No	No	No	No	No	No	No
Heitor Moreno Júnior	No	No	No	No	No	No	No
Helius Carlos Finimundi	No	No	No	No	No	No	No
Isabel Cristina Britto Guimarães	No	No	No	No	No	No	No
Isabela de Carlos Back	No	No	No	No	No	No	No
João Bosco de Oliveira Filho	No	No	No	No	Novartis, Bristol, AztraZeneca	No	No
João Roberto Gemelli	No	No	No	No	Boeringher, Libbs	No	Boeringher, Libbs
José Fernando Vilela-Martin	No	No	No	No	No	No	No
Jose Geraldo Mill	No	No	No	No	No	No	No
José Marcio Ribeiro	No	No	No	No	No	No	No
Juan Carlos Yugar-Toledo	No	No	No	No	No	No	No
Leda A. Daud Lotaif	No	No	No	No	No	No	No
Lilian Soares da Costa	No	No	No	No	No	No	No
Lucélia Batista Neves Cunha Magalhães	No	No	No	No	No	No	No
Luciano Ferreira Drager	No	Aché, Biolab, Boehringer, Merck	ResMed	No	No	Aché, Biolab, Merck	No
Luis Cuadrado Martin	No	No	No	No	No	No	No
Luiz Aparecido Bortolotto	No	Servier, Novonordisk	No	No	No	No	No
Luiz César Nazário Scala	No	No	No	No	No	No	No
Madson Q. Almeida	No	No	No	No	No	No	No
Marcia Maria Godoy Gowdak	No	No	No	No	No	No	No
Marcia Regina Simas Torres Klein	No	No	No	No	No	No	No
Marco Antônio Mota-Gomes	Omron, Beliva	No	Omron, Libbs	No	No	Omron, Libbs	No
Marcus Vinícius Bolívar Malachias	No	Libbs, Biolab	No	No	No	Libbs. Biolab	No
Maria Cristina Caetano Kuschnir	No	No	No	No	No	No	No
Maria Eliane Campos Magalhães	No	No	No	No	No	No	No
Maria Eliete Pinheiro	No	No	No	No	No	No	No
Mario Fritsch Toros Neves	No	Servier	No	No	No	No	No
Mario Henrique Elesbão de Borba	No	EMS	No	No	No	No	No
Nelson Dinamarco Ludovico	No	Não	No	No	No	No	No
Osni Moreira Filho	No	Biolab, Servier	No	No	No	No	No
Oswaldo Passarelli Júnior	No	No	No	No	No	No	No
Otávio Rizzi Coelho	No	Daichi-Sankyo, Boehringer	No	Daichi-Sankyo, BAYER, Novo-Nordisk	No	Sanofi, Takeda, AstraZeneca, Daichi-Sankyo, Bayer	No
Paulo César Brandão Veiga Jardim	No	Servier, Libbs, EMS	No	No	No	Servier, Libbs	No
Priscila Valverde Vitorino	No	No	No	No	No	No	No
Renault Mattos Ribeiro Júnior	No	Daiichi Sankyo	No	No	No	No	No
Roberto Dischinger Miranda	No	EMS, Boehringher	No	No	No	EMS, Sanofi, Servier	No
Roberto Esporcatte	No	EMS	No	No	No	No	No
Roberto Franco	No	No	No	No	No	No	No
Rodrigo Pedrosa	No	No	No	No	No	No	No
Rogerio Andrade Mulinari	No	No	No	No	No	No	No
Rogério Baumgratz de Paula	No	No	No	No	No	No	No
Rogerio Toshiro Passos Okawa	No	No	No	No	No	No	No
Ronaldo Fernandes Rosa	No	No	No	No	No	No	No
Rui Manuel dos Santos Póvoa	No	No	No	No	No	No	No
Sandra Fuchs	No	No	No	No	No	No	No
Sandra Lia do Amaral	No	No	No	No	No	No	No
Sebastião R. Ferreira-Filho	No	No	No	No	No	No	No
Sergio Emanuel Kaiser	Engage, Alecardio, RED-HF, Odissey-Outcomes, SELECT	Amgen, Novo Nordisk, Novartis, astrazeneca, Momenta Farma, Daiichi-Sankyo, Pfizer, Baldacci	No	No	No	Novartis, Momenta Farma, Farmasa, EMS	No
Thiago de Souza Veiga Jardim	No	AstraZeneca, Torrent, Meck, Bayer	No	No	No	No	No
Vanildo Guimarães	No	Boehringer, Novartis, Sandoz	No	No	No	No	No
Vera H. Koch	No	No	No	No	No	No	No
Weimar Kunz Sebba Barroso de Souza	Ministério da Saúde, Sociedade Europeia de Hipertensão Arterial, Artery Society, EMS	EMS, Libbs, Sandoz, Servier, Cardios, Omron	Omron	No	EMS, Servier	EMS, Servier, Omron	No
Wille Oigman	No	No	No	No	No	No	No
Wilson Nadruz	No	No	No	No	No	No	No

**Table:** List of Abbreviations

ABI	ankle-brachial index	GBD	*global burden diseases*
ABPM	ambulatory blood pressure monitoring	GH	growth hormone
AC	arm circumference	GRS	global risk score
ACEI	angiotensin-converting enzyme inhibitor	GST	gait speed test
ADL	activity of daily living	HBP	high blood pressure
AE	adverse event	HBPM	home blood pressure monitoring
AF	atrial fibrillation	HC	hypertensive crisis
Aix	*augmentation index*	HDL	high-density lipoprotein
AMI	acute myocardial infarction	HE	hypertensive emergency
APA	aldosterone-producing adenomas	HELPP	*hemolysis, elevated liver enzymes, low platelets*
APE	acute pulmonary edema	HF	heart failure
ARB	angiotensin II AT1 receptor blocker	HFpEF	heart failure with preserved ejection fraction
ASA	acetylsalicylic acid	HFrEF	heart failure with reduced ejection fraction
BB	beta-blockers	HPLC	*high-performance liquid chromatography*
BE	blinding effect	HR	heart rate
BMI	body mass index	hs-TnT	high-sensitivity troponin T
BP	blood pressure	HT	hypertension
CAD	coronary artery disease	HTPC	hypertensive pseudocrisis
CCB	calcium channel blocker	HU	hypertensive urgency
cfPWV	carotid-femoral pulse wave velocity	ICU	intensive care unit
CGA	comprehensive geriatric assessment	IGF-1	*insulin-like growth factor-1*
CHW	community health worker	IV	intravenous
CKD	chronic kidney disease	LE	level of evidence
CO	cardiac output	LLs	lower limbs
CRP	C-reactive protein	LR	level of recommendation
CT	computed tomography	LSC	lifestyle change
CV	cardiovascular	MH	masked hypertension
CVD	cerebrovascular disease	MNR	magnetic nuclear resonance
CVD	cardiovascular disease	MOD	multi-organ damage
CVRF	cardiovascular risk factor	MS	metabolic syndrome
DALYs	disability-adjusted life years	NB	newborn
DBP	diastolic blood pressure	NCD	noncommunicable disease
DIU	diuretics	NE	norepinephrine
DM	diabetes mellitus	NIHSS	National Institute of Health Stroke Scale
EF	ejection fraction	NO	nitric oxide
eGFR	estimated glomerular filtration rate	NOO-	peroxynitrite
eNOS	endothelial nitric oxide synthase	NPT	nonpharmacological treatment
EOD	end-organ damage	NT-proBNP	N-terminal pro b-type natriuretic peptide
FMD	fibromuscular dysplasia	NTG	nitroglycerin
FMD	flow-mediated dilation	OH	orthostatic hypotension
OSA	obstructive sleep apnea	RHT	resistant hypertension
PE	pre-eclampsia	RVH	renovascular hypertension
PEF	preserved ejection fraction	SBP	systolic blood pressure
PHEO	pheochromocytoma	SHT	sustained hypertension
PNS	Brazilian National Health Survey	SMBP	self-measured blood pressure
PNS	parasympathetic nervous system	SNP	sodium nitroprusside
POAD	peripheral occlusive atherosclerotic disease	SNS	sympathetic nervous system
PPH	postprandial hypotension	SUS	Brazilian Unified Health System
PRA	plasma renin activity	T4	thyroxine
PVR	peripheral vascular resistance	TG	triglycerides
PWV	pulse wave velocity	TNT	true normotension
R/S	religiosity and spirituality	TSH	thyroid-stimulating hormone
RAAS	renin-angiotensin-aldosterone system	UAOBPM	unobserved automated office blood pressure measurement
RAS	renal artery stenosis	UN	United Nations
RCT	randomized controlled trial	WCE	white-coat effect
REF	reduced ejection fraction	WCH	white coat hypertension
RF	risk factors	WHO	World Health Organization
RfHT	refractory hypertension	YLL	*years of life lost*

## Content

1. Definition, Epidemiology, and Primary Prevention 528

1.1 Definition of Hypertension 528

1.2. Impact of Hypertension on Cardiovascular Diseases 528

1.3. Risk Factors for Hypertension 528

**1.3.1. Genetics** 528

**1.3.2. Age** 528

**1.3.3. Sex** 528

**1.3.4. Race/Ethnicity** 528

**1.3.5. Overweight/Obesity** 528

**1.3.6. Sodium and Potassium Intake** 528

**1.3.7. Sedentary lifestyle** 529

**1.3.8. Alcohol** 529

**1.3.9. Socioeconomic Factors** 529

**1.3.10. Other Risk Factors for High BP** 529

**1.3.11. Obstructive Sleep Apnea (OSA)** 529

**1.3.12. Global Epidemiological Data** 529

1.4. Prevalence of Hypertension in Brazil 529

1.5. Primary Prevention 530

**1.5.1. Introduction** 530

**1.5.2. Weight Control (LR: I; LEE: A)** 530

**1.5.3. Healthy Diet (LR: I; LE: A)** 530

**1.5.4. Sodium (LR: I; LE: A)** 530

**1.5.5. Potassium (LR: I; LE: A)** 530

**1.5.6. Physical Activity (LR: I; LE: A)** 530

**1.5.7. Alcohol (LR: IIA; LE: B)** 531

**1.5.8. Psychosocial Factors (LR: IIb; LE: B)** 531

**1.5.9. Dietary Supplements (LR: I to III; LE: A and B)** 531

**1.5.10. Smoking (LR: I; LE: A)** 531

**1.5.11. Spirituality (LR: I; LE: B)** 531

1.6. Strategies for the Implementation of Preventive Measures 531

2. Blood Pressure and Vascular Damage 535

2.1. Introduction 535

2.2. Blood Pressure, Clinical Outcomes, and Cardiovascular Damage 535

2.3. Blood Pressure, Inflammation, and Endothelial Dysfunction 536

2.4. Blood Pressure and Arterial Stiffness 536

**2.4.1. Ankle-Brachial Index (ABI)** 536

**2.4.2. Pulse Wave Velocity (PWV)** 537

**2.4.3. Central Blood Pressure** 537

3. Diagnosis and Classification 540

3.1. Introduction 540

3.2. Blood pressure measurement at the physician's office 540

3.3. Classification 540

3.4. Out-of-Office Blood Pressure Measurement 541

3.5. White-Coat Effect (WCE) and Masking Effect (ME) 541

3.6. White Coat Hypertension (WCH) and Masked Hypertension (MH) 541

3.7. Uncontrolled Masked and White Coat Hypertension 541

3.8. Diagnosis and Follow-Up Recommendations 541

3.9. Central Aortic Pressure 542

3.10. Genetics and Hypertension 542

4. Clinical and Complementary Assessment 548

4.1. Clinical History 548

4.2. Clinical Assessment 548

**4.2.1. History-Taking** 548

4.3. Physical Examination 548

**4.3.1. Basic Laboratory Investigation, Assessment of Subclinical and Clinical End-Organ Damage** 548

5. Cardiovascular Risk Stratification 552

5.1. Introduction 552

5.2. Additional Risk Stratification (Associated Conditions) 552

**5.2.1. End-Organ Damage** 553

**5.2.2. Presence of Cardiovascular and Renal Disease** 553

5.3. Assessment of Global Cardiovascular Risk 553

5.4. Challenges of Cardiovascular Risk Assessment in Hypertension 553

6. Therapeutic Decision and Targets 556

6.1. Introduction 556

6.2. Low- or Moderate-Risk Hypertensive Patients 556

6.3. High-Risk Hypertensive Patients 556

6.4. Hypertensive Patients with Coronary Disease 556

6.5. Hypertensive Patients with History of Stroke 556

6.6. Hypertensive Heart Failure Patients 557

6.7. Hypertensive Patients with Chronic Kidney Disease (CKD) 557

6.8. Diabetic Hypertensive Patients 557

6.9. Older Hypertensive Patients 557

7. Multidisciplinary Team 559

7.1. The Importance of a Multidisciplinary Approach to Hypertension Control 7.2. Team Composition and Work 559

**7.2.1. Medical Professional: Specific Actions** 559

**7.2.2. Nursing Professional: Specific Actions** 559

***7.2.2.1. Nursing-Specific Actions in Primary Care*** 559

**7.2.3. Nutrition Professional: Specific Actions** 560

***7.2.3.1. Dietetic Consultation*** 560

***7.2.3.2. Collective Actions by Nutritionists*** 560

**7.2.4. Physical Education Professional: Specific Actions** 560

***7.2.4.1. Collective Actions by Physical Education and Physical Therapy Professionals*** 560

7.3. Multidisciplinary Team Actions 561

8. Nonpharmacological Treatment 562

8.1. Introduction 562

8.2. Smoking 562

8.3. Dietary Patterns 562

8.4. Sodium Intake 563

8.5. Potassium 563

8.6. Dairy Products 563

8.7. Chocolate and Cocoa Products 563

8.8. Coffee and Caffeinated Products 563

8.9. Vitamin D 563

8.10. Supplements and Substitutes 564

8.11. Weight Loss 564

8.12. Alcohol Consumption 564

8.13. Physical Activity and Physical Exercise 564

8.14. Slow Breathing 565

8.15. Stress Control 565

8.16. Religiosity and Spirituality 565

9. Pharmacological Treatment 568

9.1. Treatment Objectives 568

9.2. General Principles of Pharmacological Treatment 568

9.3. Therapy Regimens 568

**9.3.1. Monotherapy** 568

**9.3.2. Drug Combinations** 568

9.4. General Characteristics of Different Classes of Antihypertensive Medications 569

**9.4.1. Diuretics (DIUs)** 569

***9.4.1.1. Adverse Effects of Diuretics*** 569

**9.4.2. Calcium Channel Blockers (CCBs)** 569

***9.4.2.1. Adverse Effects of Calcium Channel Blockers*** 570

**9.4.3. Angiotensin-Converting Enzyme Inhibitors (ACEIs)** 570

***9.4.3.1. Adverse Effects of Angiotensin-Converting Enzyme Inhibitors* 9.4.4. Angiotensin II AT1 Receptor Blockers (ARBs)** 570

***9.4.4.1. Adverse Effects of Angiotensin II AT1 Receptor Blockers*** 570

**9.4.5. Beta-Blockers (BBs)** 570

***9.4.5.1. Adverse Effects of Beta-Blockers*** 571

**9.4.6. Centrally Acting Sympatholytics** 571

***9.4.6.1. Adverse Effects of Centrally Acting Sympatholytics*** 571

**9.4.7. Alpha-blockers** 571

***9.4.7.1. Adverse Effects of Alpha-Blockers*** 571

**9.4.8. Direct-Acting Vasodilators** 571

***9.4.8.1. Adverse Effects of Direct-Acting Vasodilators*** 571

**9.4.9. Direct Renin Inhibitors** 571

***9.4.9.1. Adverse Effects of Direct Renin Inhibitors*** 572

9.5. Antihypertensive Drug Combinations 572

10. Hypertension and Associated Clinical Conditions 578

10.1. Diabetes Mellitus (DM) 578

**10.1.1. Treatment Objectives** 578

10.2. Metabolic Syndrome (MS) 578

10.3. Coronary Artery Disease (CAD) 578

10.4. Hypertension in Chronic Kidney Disease (CKD) 578

**10.4.1. Patient in Conservative Treatment: Goals and Treatment** 578

**10.4.2. Patients in Renal Replacement Therapy (RRT): Goals and Treatment** 579

10.5. Heart Failure (HF) 579

10.6. Hemorrhagic Stroke and Ischemic Stroke 580

**10.6.1. Hemorrhagic Stroke** 580

**10.6.2. Ischemic Stroke** 580

11. Hypertension in Pregnancy 581

11.1. Epidemiology 581

11.2. Classification of Hypertension in Pregnancy 581

11.3. Concept and Diagnostic Criteria 581

11.4. Prediction and prevention of pre-eclampsia 581

11.5. Nonpharmacological treatment 582

11.6. Expectant management 582

11.7. Pharmacological treatment 582

11.8 Future Cardiovascular Risk 583

12. Hypertension in Children and Adolescents 586

12.1. Epidemiological Context and Importance of Hypertension in Pediatrics 586 12.2. Definition and Etiology 586

12.3. Diagnostic 586

**12.3.1. BP Measurement Methods** 586

12.4. History-Taking 587

12.5. Physical Examination 587

12.7. Ambulatory Blood Pressure Monitoring (ABPM) 587

12.8. Therapeutic Aspects 587

12.9. Nonpharmacological Therapy 587

12.10. Pharmacological Therapy 587

12.11. Follow-up of Children and Adolescents with HT 588

12.12. Hypertensive Crisis 588

13. Hypertensive Crisis 596

13.1. Definition 596

13.2. Classification 596

13.3. Major Epidemiological, Pathophysiological, and Prognostic Aspects 596

**13.3.1. Epidemiology** 596

**13.3.2. Pathophysiology** 596

**13.3.3. Prognosis** 596

13.4. Complementary Clinical and Laboratory Investigation 597

13.5. General Treatment of Hypertensive Crisis 597

13.6. Hypertensive Emergencies in Special Situations 597

**13.6.1. Hypertensive Encephalopathy** 597

13.7. Stroke 597

**13.7.1. Ischemic Stroke** 597

**13.7.2. Hemorrhagic Stroke** 598

**13.7.3. Acute Coronary Syndromes** 598

**13.7.4. Acute Pulmonary Edema (APE)** 598

***13.7.4.1. Acute Aortic Dissection*** 598

**13.7.5. Pre-eclampsia/Eclampsia** 598

**13.7.6. HE from Illicit Drug Use** 598

**13.7.7. Accelerated/Malignant Hypertension** 598

**13.7.8. Hypertension with Multi-Organ Damage** 599

14. Hypertension in Older Adults 602

14.1. Introduction 602

14.2. Physiopathological Mechanisms 602

14.3. Diagnosis and Therapeutic Decision 603

14.4. Treatment 603

**14.4.1. Nonpharmacological Treatment** 603

**14.4.2. Pharmacological Treatment** 603

14.5. Special Situations 604

**14.5.1. Functional Status and Frailty: Assessment and Implications** 604

**14.5.2. Cognitive Decline and Dementia** 604

**14.5.3. Polypharmacy and Adherence** 604

**14.5.4. Deintensification and Deprescription** 605

**14.5.5. Orthostatic and Postprandial Hypotension** 605

15. Secondary Hypertension 606

15.1. Introduction 606

15.2. Nonendocrine Causes 606

**15.2.1. Chronic Kidney Disease (CKD)** 606

**15.2.2. Renovascular Hypertension (RVH)** 606

15.3. Fibromuscular Dysplasia 607

**15.3.1. Coarctation of the Aorta** 607

**15.3.2. Obstructive Sleep Apnea (OSA)** 607

***15.3.2.1. Concept and Epidemiology*** 607

***15.3.2.2. Clinical Presentation and Screening of OSA in Hypertension*** 608

***15.3.2.3. Impact of Treatment of OSA on BP*** 608

***15.3.2.4. Antihypertensive Treatment in Hypertensive Patients with OSA*** 608

15.4. Endocrine Causes 608

**15.4.1. Primary Hyperaldosteronism (PH)** 608

**15.4.2. Pheochromocytoma** 609

**15.4.3. Hypothyroidism** 609

**15.4.4. Hyperthyroidism** 609

**15.4.5. Primary Hyperparathyroidism** 609

**15.4.6. Cushing's Syndrome** 610

**15.4.7. Obesity** 610 **15.4.8. Acromegaly** 610 15.5. Pharmacological Causes, Hormones, and Exogenous Substances 610

16. Resistant and Refractory Hypertension 618 16.1. Definition and Classification 618 16.2. Epidemiology of Resistant Hypertension 618 16.3. Pathophysiology 618 16.4. Diagnostic Investigation 618 16.5. Treatment 618 **16.5.1. Nonpharmacological Treatment** 618 **16.5.2. Pharmacological Treatment** 619 **16.5.3. New Treatments** 619

17. Adherence to Antihypertensive Treatment 622

17.1. Introduction 622

17.2. Concept and Adherence 622

17.3. Treatment Adherence Assessment Methods 622

17.4. Factors Interfering in Adherence to Treatment 623

17.5. Strategies to promote adherence to antihypertensive treatment 623

17.6. Conclusion 623

18. Perspectives 625

18.1. Introduction 625

18.2. Definition, Epidemiology, and Primary Prevention 625

18.3. Blood Pressure and Vascular Damage 625

18.4. Cardiac Biomarkers 625

18.5. Diagnosis and Classification 625

18.6. Complementary Assessment and Cardiovascular Risk Stratification 626

18.7. Goals and Treatment 626

Reference 628

## 1. Definition, Epidemiology, and Primary Prevention

## 1.1. Definition of Hypertension

Hypertension (HT) is a chronic noncommunicable disease (NCD) defined by blood pressure levels for which the benefits of treatment (nonpharmacological and/or pharmacological) outweigh the risks. HT is a multifactorial condition, depending on genetic/epigenetic, environmental, and social factors ( Figure 1.1 ), characterized by persistent high blood pressure (BP), ie, systolic blood pressure (SBP) equal to or greater than 140 mm Hg and diastolic blood pressure (DBP) equal to or greater than 90 mm Hg, measured using the appropriate technique, on at least two different occasions, in the absence of antihypertensive medication. When possible, it is advised that these measurements be validated by assessing BP outside the physician's office using ambulatory blood pressure monitoring (ABPM), home blood pressure monitoring (HBPM), or self-measured blood pressure (SMBP) (see Chapter 3).

**Figure 1.1:** – Schematic description of major determinants of blood pressure and hypertension and their interactions n adults. Genetic/epigenetic, environmental, and social determinants interact to increase the BP of hypertensive patients and in the general population. ↑increased; ↓decreased.

Source: Carey et al. 2008. ^6^

**Figure 1.2:** – Percentage of deaths from hypertension, acute myocardial infarction, stroke, and chronic renal failure (Brazil, 2000).

## 1.2. Impact of Hypertension on Cardiovascular Diseases

As an often asymptomatic condition, BP usually progresses to structural and/or functional change to end organs, such as the heart, brain, kidneys, and blood vessels. It is the primary modifiable risk factor, independently, linearly, and continuously associated, for cardiovascular disease (CVD), chronic kidney disease (CVD), and early death. It is associated with metabolic risk factors for cardiocirculatory and renal diseases, such as dyslipidemia, abdominal obesity, glucose intolerance, and diabetes mellitus (DM). ^1^ ^-^ ^6^

In addition, it has a significant impact on socioeconomic and medical costs due to fatal and nonfatal complications to end organs, such as: heart: coronary artery disease (CAD), heart failure (HF), atrial fibrillation (AF), and sudden death; brain: stroke, ischemic stroke, hemorrhagic stroke, and dementia; kidneys; CKD that may require dialysis therapy; and arterial system: peripheral occlusive atherosclerotic disease (POAD). ^3^ ^-^ ^6^

## 1.3. Risk Factors for Hypertension

### 1.3.1. Genetics

Genetic factors may influence BP levels from 30 to 50%. ^7^ However, due to wide genetic diversity, the gene variants we have studied thus far and Brazilian miscigenation, uniform data for this factor have yet to be identified. Further details about the genetic component of HT can be found in Chapter 3.

### 1.3.2. Age

SBP becomes a more significant problem with age, the result of the progressive hardening and decreased compliance of the great arteries. Approximately 65 percent of people age 60 or older have HT, and we should take into consideration Brazil's ongoing epidemiological transition, with an even greater number of older adults (age ≥ 60) in the coming decades leading to a substantial increase in the prevalence of HT and its complications. ^7^ ^,^ ^8^

### 1.3.3. Sex

Among younger cohorts, BP is higher in men, but rises faster by decade in women. Therefore, in their sixth decades, women's BP is usually higher than men's, as is the prevalence of HT. For both sexes, the frequency of HT rises with age, reaching 61.5% and 68.0% for men and women age 65 or older, respectively. ^7^

### 1.3.4. Race/Ethnicity

Race and ethnicity are important risk factors for HT, but socioeconomic status and lifestyle seem to be more relevant for the differing prevalence of HT than race and ethnicity themselves. ^7^ ^,^ ^8^ The Vigitel 2018 data show that, in Brazil, there was no significant differences between blacks and whites regarding the prevalence of HT (24.9% *versus* 24.2%). ^9^

### 1.3.5. Overweight/Obesity

There seems to be a direct, continuous, and almost linear relationship between overweight/obesity and BP levels. ^3^ ^-^ ^6^ Despite decades of unequivocal evidence that waist circumference (CC) provides both independent and additive information to body mass index (BMI) for predicting morbidity and risk of death, this parameter is not routinely measured in clinical practice. It is recommended that health professionals be trained to properly perform this simple measurement and consider it as an important “vital sign” in clinical practice. ^3^ ^-^ ^6^

### 1.3.6. Sodium and Potassium Intake

High sodium intake has been shown to be a risk factor for high BP and consequently for the greater prevalence of HT. The literature shows that sodium intake is associated with CVD and stroke when mean intake is greater than 2 g of sodium, equivalent to 5 g of table salt. ^10^ Sodium excretion studies show that, for those with high sodium intake, SBP was 4,5-6.0 mm Hg higher, and DBP 2.3-2.5 mm Hg higher, than for those at recommended sodium intake levels. ^11^

It should also be stressed that excess sodium intake is one of the main modifiable risk factors for preventing and controlling HT and CVD, and that the Brazilian Unified Health System (SUS) spent USD 102 million in 2013 alone on hospitalizations attributable to excess sodium intake. ^12^

Conversely, increased sodium intake reduces blood pressure levels. It is worth highlighting that the effects of potassium supplementation seems to be greater for those with high sodium intake and for black people. Mean salt intake in Brazil is 9.3 g/day (9.63 g/day for men and 9.08 g/day for women), while potassium intake is 2.7 g/day for men and 2.1 g/day for women. ^12^ ^,^ ^13^

### 1.3.7. Sedentary lifestyle

There is a direct association between a sedentary lifestyle, high BP, and HT. ^3^ ^-^ ^6^ It should be noted that, globally in 2018, the rate of lack of physical activity (less than 150 minutes of physical activity per week or 75 minutes of vigorous physical activity per week) was 27.5%, with greater prevalence among women (31.7%) than men (23.4%). ^14^

In Brazil, the 2019 Vigitel phone survey found that 44.8% of adults did not perform sufficient levels of physical activity, and rates were worse for women (52.2%) than for men (36,1%). ^9^

### 1.3.8. Alcohol

The impact of alcohol intake has been investigated in various epidemiological studies. There is greater prevalence of HT or high blood pressure levels for those taking six or more doses per day, equivalent to 30 g of alcohol/day = 1 bottle of beer (5% alcohol, 600 mL); = 2 glasses of wine (12% alcohol, 250 mL); = 1 dose (42% alcohol, 60 mL) of distilled beverages (whiskey, vodka, spirits). That threshold should be cut in half for low-weight men and for women. ^15^ ^,^ ^16^

### 1.3.9. Socioeconomic Factors

Socioeconomic factors include lower educational levels, inadequate living conditions, and low family income as significant risk factors for HT. ^17^ ^,^ ^18^

### 1.3.10. Other Risk Factors for High BP

In addition to the classic factors listed above, it is important to stress that some medications, often acquired without prescription, have the potential to promote high BP or make it harder to control, as do illicit drugs. The subject will be discussed in more detail in Chapter 15. These include monoamine oxidase inhibitors and sympathomimetic, such as decongestants (phenylephrine), tricyclic antidepressants (imipramine and others), thyroid hormones, oral contraceptives, nonsteroidal anti-inflammatory drugs, carbenoxolone and liquorice, glucocorticoids, cyclosporine, erythropoietin, and illicit drugs (cocaine, cannabis, amphetamine and 3,4-methylenedioxymethamphetamine (MDMA)). ^5^ ^,^ ^19^

### 1.3.11. Obstructive Sleep Apnea (OSA)

There is clear evidence behind the relation between OSA and HT and increased risk of resistant HT (see also Chapter 15). Mild, moderate, and severe OSA has a dose-response relationship with HT. There is a stronger association for Caucasian and male patients with OSA. ^3^ ^-^ ^6^ ^,^ ^20^

### 1.3.12. Global Epidemiological Data

CVD are the main cause of death, hospitalization, and outpatient medical visits worldwide, including developing countries such as Brazil. ^21^ In 2017, complete and revised data from Datasus showed a total of 1 312 663 deaths, 27.3% of which from CVD. ^22^ HT was associated with 45% of cardiac deaths (CAD and HF), 51.0% of deaths from cerebrovascular disease (CVD), and a small percentage of deaths directly related to HT (13.0%). It should be stressed that HT kills more by causing end-organ damage ^23^ ( Figure 1.2 ).

In 2017, data from Global Burden of Disease (GBD) indicated that CVD accounted for 28.8% of total deaths from noncommunicable diseases (NCD). The GBD study found that there were almost 18 million deaths from CV causes in 2017 (31.8% of total deaths), accounting for 20.6% of total years of life lost (YLL) and 14,7% total DALYs (disability-adjusted life years, ie, years of healthy life lost). ^18^ ^,^ ^21^

Also according to GBD, SBP increase was found to be the main risk factor, responsible for 10.4 million deaths and 218 million DALYs. ^21^ It also accounts for approximately 40.0% of deaths of DM patients, 14.0% of maternal and fetal mortality during pregnancy, and 14.7% of total DALYs from CKD. ^24^ ^-^ ^26^

Globally, in 2010, HT prevalence (≥140/90 mm Hg and/or use of antihypertensive medication) was 31.0%, higher for men (31.9%) than for women (30.1%). ^17^ ^,^ ^18^

A study on worldwide trends in blood pressure from 1975 to 2015 assessing 19.1 million adults found that, in 2015, there was an estimated 1.13 billion adults with HT (597 million men and 529 million women), suggesting a 90% increase in the number of people with HT, especially in low- and medium-income countries. ^17^ ^,^ ^18^ The study found that HT prevalence decreased in high-income countries and some medium-income ones, but increased or held steady in lower-income nations. The factors implicated in that increase are likely population aging and greater exposure to other risk factors, such as high sodium and low potassium intake, in addition to sedentary lifestyles. ^17^ ^,^ ^18^

## 1.4. Prevalence of Hypertension in Brazil

Countrywide prevalence data tend to vary according to the methodology and sample chosen. According to the 2013 Brazilian National Health Survey, 21.4% (95% CI 20.8-22.0) of Brazilian adults self-report HT, while BP readings and the use of antihypertensive medications indicate that the share of adults with BP at or above 140/90 mm Hg is approximately 32.3% (95% CI 31.7-33.0). HT prevalence was found to be higher among men and, as expected, to increase with age regardless of other parameters, reaching 71.7% for individuals age 70 and older ( Table 1.1 and Figure 1.3 ). ^27^

**Table 1.1:** – Prevalence of hypertension and 95% confidence interval according to three criteria

	Self-reported HT (Vigitel)	Measured BP ≥ 140/90 mm Hg (PNS, 2013)	Measured BP ≥ 140/90 mm Hg and/or use of antihypertensive medication (PNS, 2013)
Total	21;4% (20;8-22;0)	22;8% (22.1-23.4)	32.3% (31.7-33.0)
Male	18.3 (17.5-19.1)	25.8 (24.8-26.7)	33.0 (32.1-34.0)
Female	24.2 (23.4-24.9)	20.0 (19.3-20.8)	31.7 (30.9-32.5)

HT: hypertension; BP: blood pressure. Source: Nilson et al. 2020. ^29^

In 2017, there was a total of 1 312 663 deaths, 27.3% of which from CVD, accounting for 22.6% of all early deaths in Brazil (ages 30 to 69). In one ten-year period (2008 through 2017), it is estimated that 667 184 could be attributed to HT in Brazil. ^21^ ^-^ ^23^

In the death rate per 100 000 inhabitants from 2000 to 2018, we can see a slight uptick in AMI and a jump in direct HT, with 25% and 128% increases, respectively. ^23^

As for morbidity, we can see the population-adjusted hospitalization trend has been stable over the last ten years (Datasus Hospitalization System) both for all causes and for CVD ( Figure 1.3 ). ^5^ ^,^ ^23^ More of the Brazilian health system's costs can be attributed to HT than to obesity and DM. In 2018, it is estimated that SUS spent USD 523.7 million in hospitalizations, outpatient procedures, and medications. ^28^

Over the last decade, CVD associated with HT account for 77% of the Brazilian Unified Health System's (SUS) hospitalization costs from CAD, and they increased 32% from 2010 to 2019 in Brazilian reais, from R$ 1.6 billion to R$ 2.2 billion over the same period. ^28^ ^,^ ^29^

## 1.5. Primary Prevention

## 1.5.1. Introduction

HT is highly prevalent and a major risk factor for CVD and kidney disease, combining genetic, environmental, and social determinants. It is easily diagnosable and effectively treatable by a diverse and highly efficiency therapeutic arsenal with few adverse effects. Even so, globally, the fact that it is an often asymptomatic disease means adherence to care is difficult and it remains mostly uncontrolled worldwide.

That equation makes treatment extremely challenging, and prevention remains the best option from a cost-benefit perspective. An adequate approach to risk factors for HT should be a major point of focus for SUS (the Brazilian Unified Health System). Several aspects of that issue deserve further consideration. Many are interwoven or ad to nonpharmacological treatment ( Chart 1.1 ), detailed in Chapter 8. ^3^ ^,^ ^5^ ^,^ ^6^ ^,^ ^30^ ^,^ ^31^

**Chart 1.1:** – Main interventions that prevent hypertension

Modality	NP Intervention	Dose	Difference in SBP measured
Weight control	Body weight/fat	Reach ideal weight. Expected decrease of 1mm Hg per kg of body weight lost	- 2/3 mm Hg
Healthy diet	DASH diet	Diet rich in fruits, vegetables, grain, and low fat content Reduction in saturated and trans fats	- 3 mm Hg
Lower sodium intake	Sodium intake	Ideal < 2 g or at least 1.0 g/day less	- 2/3 mm Hg
Increased potassium intake	Potassium intake	3.5 to 5,0 g/day in a potassium-rich diet	- 2 mm Hg
Physical activity	Aerobic	150 min/week	- 5/7 mmHg
	Dynamic resistance	8 to 10 exercises for the major muscle groups, 1 to 3 sets, 50 to 80% of 1 RM	
	Isometric resistance	Unilateral handgrip exercise or 1 leg, 4 sets, 2 min isometric contraction, 30% of maximum voluntary contraction (MVC), 2-3 min break between sets	- 4/5 mm Hg
Alcohol intake	Alcohol consumption	For alcohol drinkers Men ≤ 2 drinks Women ≤ 1 drink	- 4/5 mm Hg

mm Hg: millimeters of mercury; NP: nonpharmacological; RM: repetition maximum; SBP: systolic blood pressure. Source: Adapted from Carey et al., 2018. ^6^

## 1.5.2. Weight Control (LR: I; LEE: A)

Overall and central obesity are associated with increased risk of HT. On the other hand, weight loss promoted lower BP both for normotensive and for hypertensive individuals. ^3^ ^,^ ^5^ ^,^ ^6^ Being “as lean as possible” within the normal BMI range may be the best suggestion for primary prevention of HT. ^3^ ^,^ ^5^ ^,^ ^6^ ^,^ ^32^ ^-^ ^36^

## 1.5.3. Healthy Diet (LR: I; LE: A)

Several diets have been proposed for HT prevention which also favor hypertension control and contribute to health as a whole. ^5^ ^,^ ^37^ One major proposal to that end is the DASH diet and its variants (low fat, Mediterranean, vegetarian/vegan, Nordic, low carbohydrate content, etc.). The benefits are even greater when combined with lower sodium intake. ^5^ ^,^ ^37^ ^-^ ^40^

Every report on the subject recommends eating healthy amounts of fruits, greens, vegetables, cereal, milk, and dairy products, as well as lowering salt and fat intake. ^37^ ^-^ ^41^ A meta-analysis compared varieties of these diets with the standard diet and found a greater decrease in SBP (-9.73 to -2.32 mm Hg) and DBP (-4.85 to -1.27 mm Hg) in the proper diet group. ^39^ Socioeconomic and cultural aspects have to be taken into account to ensure adherence to a given kind of dietary recommendation. ^3^ ^,^ ^5^ ^,^ ^6^ ^,^ ^37^

## 1.5.4. Sodium (LR: I; LE: A)

Excess sodium intake is one of the main modifiable risk factors for preventing and controlling HT and CVD. ^29^ Sodium restriction has been shown to lower BP in several studies. A meta-analysis found that a 1.75 g decrease in daily sodium intake (4.4 g of salt/day) is associated with a mean decrease of 4.2 and 2.1 mm Hg in SBP and DBP, respectively. The BP decrease from sodium restriction is greater in blacks, older adults, diabetic patients, and individuals suffering from metabolic syndrome (MS) and CKD. ^37^

In the general population, individuals are recommended to restrict their sodium intake to approximately 2 g/day (equivalent to about 5 g of salt per day). ^3^ ^-^ ^6^ Effectively lowering salt intake is not easy, and low-salt foods are often underappreciated. Patients should be advised to take care with how much salt they add to their food and not to eat high-salt items (industrialized and processed foods). ^3^ ^-^ ^6^

Decreasing Brazilian salt intake remains a high public health priority, but requires combined efforts from the food industry, all levels of government, and the public in general, since 80% of salt comes from processed foods. ^3^ ^-^ ^6^ ^,^ ^10^ ^,^ ^12^ ^,^ ^40^ Adequate intake of fruits and vegetables leverages the beneficial effects of a low-sodium diet on BP. Salt substitutes with potassium chloride and less sodium chloride (30 to 50%) are useful to help lower sodium intake and increase potassium intake, despite their restrictions. ^42^

### 1.5.5. Potassium (LR: I; LE: A)

The relationship between potassium supplementation and lowering HT is relatively well understood. ^43^ Potassium supplementation represents a safe alternative, with no major adverse effects and modest but significant impact on BP, and can be recommended to help prevent the onset of HT. ^43^ ^-^ ^47^ Adequate potassium intake, on the order of 90 to 120 mEq/day, may lead to a 5.3 mm Hg decrease in SBP and a 3.1 mm Hg decrease in DBP. ^45^ Its intake can increase by opting for sodium-poor and potassium-rich foods, such as beans, peas, dark leafy greens, bananas, melons, carrots, beets, dried fruit, tomatoes, potatoes, and oranges. ^3^

### 1.5.6. Physical Activity (LR: I; LE: A)

A sedentary lifestyle is one of the ten most important risk factors for global mortality, causing approximately 3.2 million deaths per year. ^48^ ^,^ ^49^

A meta-analysis of 93 papers and 5223 individuals showed that aerobic, dynamic resistance and isometric resistance training lower SBP and DBP at rest by 3.5/2.5, 1.8/3.2 and 10.9/6.2 mm Hg, respectively, in the general population. ^50^ ^-^ ^52^

All adults should be advised to practice at least 150 min/week of moderate physical activity or 75 min/week of vigorous activity. Aerobic exercises (walking, running, bicycling, or swimming) may be practiced for 30 minutes 5 to 7 times per week. Resistance training two to three days per week is also recommended. ^50^ ^,^ ^52^ For additional benefits, in healthy adults, a gradual increase in physical activity to 300 minutes per week of moderate-intensity physical activity or 150 minutes per week of vigorous physical activity, or an equivalent combination of the two, ideally with supervised daily physical exercise. ^55^

### 1.5.7. Alcohol (LR: IIA; LE: B)

Alcohol consumption is estimated to account for approximately 10 to 30% of HT cases and approximately 6% of all-cause mortality worldwide. ^3^ ^-^ ^6^ ^,^ ^15^ ^,^ ^56^ ^-^ ^59^ Among drinkers, intake should not exceed 30 g of alcohol/day, ie, 1 bottle of beer (5% alcohol, 600 mL), two glasses of wine (12% alcohol, 250 mL), or one 1 dose (42% alcohol, 60 mL) of distilled beverages (whiskey, vodka, spirits). That threshold should be cut in half for low-weight men, women, the overweight, and/or those with high triglycerides. Teetotalers should not be encouraged to drink. ^3^ ^-^ ^6^ ^,^ ^15^

### 1.5.8. Psychosocial Factors (LR: IIb; LE: B)

There is a wide variety of techniques used to control emotional stress and contribute to HT prevention, but there is still a dearth of robust studies on the subject. ^3^ ^-^ ^6^ ^,^ ^60^ Practicing emotional stress control can help CV reactivity, BP itself, and BP variability. ^61^ ^-^ ^63^

### 1.5.9. Dietary Supplements (LR: I to III; LE: A and B)

The effects of dietary supplements on lowering BP are usually small and heterogeneous. ^58^ ^-^ ^68^ There is evidence that the following supplements can help lower BP to a small degree: vitamin C, food-derived bioactive peptides, garlic, dietary fiber, flaxseed, dark chocolate (cocoa), soy, organic nitrates, and Omega-3 fatty acids. ^38^ ^,^ ^47^ ^,^ ^69^ Magnesium supplements, multivitamins, tea, and coenzyme Q10 have not been shown to lead to significant decreases in BP. ^64^ ^,^ ^65^ ^,^ ^70^

### 1.5.10. Smoking (LR: I; LE: A)

Regardless of its impact on BP, tackling this issue is critical, since smoking is the only completely avoidable risk factor for cardiovascular disease and death, and fighting it is paramount. ^3^ ^-^ ^6^ ^,^ ^71^ ^-^ ^75^ From a prevention standpoint, the WHO recommends the following strategies for tobacco control: prevent the young from trying cigarettes, since the odds of becoming addicted for those who try are over 50%; and enforce the country's tobacco legislation, particularly the prohibition of marketing tobacco products to minors, in addition to other activities directed at this age group. ^72^ Chemical and psychological addiction makes the fight against smoking hard, but the benefits of cessation for CV mortality are apparent in the short run. ^71^ ^,^ ^73^ ^-^ ^75^

Rigor in fighting and controlling tobacco use, continuous guidance and unconditional psycho-emotional support for smokers, and occasionally prescribing medication have been shown to be the most effective approach. ^73^ It is also important to protect individuals against exposure to secondhand smoking, which also implies greater risk. ^74^

### 1.5.11. Spirituality (LR: I; LE: B)

There is growing evidence that spirituality (S), a concept transcending religiosity (R), signifying a set of moral, emotional, behavioral, and attitudinal values toward the world, provides benefits in terms of CV risk, mortality, and, in particular, blood pressure control. ^76^

The Black Women’s Health Study showed that women who coped with stressful situations through spirituality and religiosity had lower risk of developing HT over a 10-year follow-up period (incidence ratio = 0.87; 95% CI 0.75-1.00), and the association was stronger for those reporting higher levels of stress. The survey also found that R/S situations helped modulate and smooth out the challenges of daily life and brought benefits in terms of BP control. ^77^

## 1.6. Strategies for the Implementation of Preventive Measures

Lifestyle changes (LSCs) are hard to implement, and society as a whole should work together to support that effort. It is important to establish and support ongoing health education programs directed at K-12 and vocational school students, staff, corporations, and the community. Using the media to raise awareness is an important strategy; periodic focused campaigns (City, State and/or National Hypertension and Prevention Day—Federal Law 10.439 from April 30, 2002, HT Week, the International Society of Hypertension's May Measurement Month, etc.); and additional actions: incorporating HT prevention, detection, and control to primary health care programs, including children and adolescents, and particularly in school health programs; deploying multidisciplinary care programs; strengthening government norms to lower the saturated fat and sodium content of industrialized foods; enhancing nutrition fact labels; and using efficient health indicators to monitor HT prevention and control actions and their results. ^3^ ^-^ ^6^

**Table:** 

Key Takeaways
The numbers defining hypertension are arbitrary, but represent values for which the benefits of treatment (nonpharmacological and/or pharmacological) outweigh the risks.
HT is a multifactorial condition (genetics, environment, life habits and socioeconomic factors).
HT is a major risk factor for cardiovascular and kidney diseases.
HT is highly prevalent, easily diagnosed and can be properly treated, but low adherence means it is hard to control.
HT prevention is cost-effective and also the best way to decrease cardiovascular morbidity and mortality.

## 2. Blood Pressure and Vascular Damage

## 2.1. Introduction

High blood pressure (BP) values are traditionally associated with risk for ischemic heart disease, stroke, chronic kidney disease (CKD), and early death. A classic meta-analysis of 61 prospective studies, tracking 12.7 million persons-year and a record of 56 000 deaths from coronary artery disease (CAD) or stroke, produced solid observational evidence. ^78^ That meta-analysis showed that the risk begins with BP values as low as 115 mm Hg for systolic BP (SBP) or 75 mm Hg for diastolic BP (DBP), doubling for every 20 mm Hg increase in SBP or 10 mm Hg increase in DBP. Despite observational evidence, these findings have not been integrated with the definition of hypertension (HT) diagnosis, which has remained at 140/90 mm Hg for many years.

Thus, patients are still classified as hypertensive with BP levels above 140/90 mm Hg, and individuals with SBP from 120 to 139 mm Hg and DBP from 80 to 89 mm Hg are classified as having normal BP or as prehypertensives, but these have higher cardiovascular risk in comparison with their peers with normal or optimum BP levels. The impact of prehypertension (systolic BP 130-139 mm Hg, diastolic BP 85-89 mm Hg) on vascular risk was described in 2001 by Vasan et al., ^79^ who analyzed 6859 participants in the Framingham Heart Study. In that study, the authors found an increase in absolute risk for cardiovascular (CV) events. Several other studies have since been published, and their analysis included lower risk patients (systolic BP 120-139, diastolic BP 80-89 mm Hg), such as the Hisayama study, by Fukuhara et al., ^80^ which also found increased risk for CV disease.

Several other studies have been published since the early work by Vasan et al., which has led to a meta-analysis by Han et al. ^81^ in 2019, which analyzed 47 studies and a total population of 491 666 individuals. In that meta-analysis, after controlling for multiple CV risk factors, prehypertension increased the total risk of disease by 40%, including that 12.09% of CV disease, 13.26% of coronary diseases, 24;60% of myocardial infarctions (MIs), and 19.15% of strokes could have been prevented if prehypertension was effectively controlled.

That leads to the conclusion that prehypertensive individuals, even if not considered actually hypertensives, should be better assessed and stratified. Noninvasive complementary examinations can evaluate the impact of BP on vessels and analyze early vascular damage both in hypertensive and prehypertensive patients, ^82^ such as flow-mediated dilation (FMD), which checks endothelial function, and pulse wave velocity (PWV) and ankle-brachial index (ABI), which check the medial layer. The goal of this chapter is to show the impact of increased BP on CV risk, on endothelial dysfunction (damage to the vascular endothelial layer) and arterial stiffness (damage to the vascular medial layer) before HT is diagnosed.

## 2.2. Blood Pressure, Clinical Outcomes, and Cardiovascular Damage

In the meta-analysis by Law et al., ^83^ lowering SBP by 10 mm Hg in randomized controlled trials led to AMI and stroke prevention at the same rate estimated by observational studies for the same BP increase. The same was true for a more recent meta-analysis. ^84^ In this study, the relative risk reduction for CV events in trials where participants were treated to achieve SBP targets from 120 to 124 mm Hg, compared to over 160 mm Hg, was 64%, close to the 75% risk reduction for an estimated 40 mm Hg decrease in SBP from the Prospective Studies Collaboration meta-analysis. ^78^ Other meta-analyses have converged for these findings, and the largest included 600 000 participants from clinical trials. ^85^ The SPRINT clinical trial added more evidence to the studies discussed above. ^86^ The incidence of CVD decreased by 25% in patients randomized for a SBP < 120 mm Hg (intensive treatment), compared to those randomized for a target BP level below 140 mm Hg. There was a 43% decrease in CVD mortality and a 27% decrease in all-cause mortality. A similar benefit was found for participants 75 or older over the baseline, including frail individuals ^87^ (LR: I LE: A).

More recently, several cohort studies with large sample sizes have come out showing that increased BP creates similar risks for other CV outcomes as those found for CAD and stroke. These include heart failure (HF), with and without preserved ejection fraction (EF), ^88^ atrial fibrillation, ^89^ valvular heart disease, ^90^ ^,^ ^91^ peripheral arterial disease, ^92^ chronic kidney disease (CKD), ^93^ ^,^ ^94^ dementia, ^95^ ^,^ ^96^ and Alzheimer's disease. ^97^ Diabetes mellitus, ^98^ erectile dysfunction, ^99^ and age-related macular degeneration ^100^ are likely consequences of sustained high BP. In general, these consequences are externalized after many years of exposure to high blood pressure levels, usually to values previously not associated with CV risk. ^101^ The consequences of high BP can be classified by onset as early or late and comprise most CVD ( Chart 2.1 ). Recently, authors have theorized that CVD is predominantly caused by the rightward shift of the BP distribution curve on a global scale. ^102^

**Chart 2.1:** – Early and late-onset consequences of chronic high BP **25**

Early- and late-onset diseases
Stroke Coronary heart disease Heart failure Cardiovascular death
**Late-onset disease**
Hypertensive cardiomyopathy Heart failure with preserved ejection fraction Atrial fibrillation Valvular heart disease Aortic syndromes Peripheral arterial disease Chronic kidney disease Dementia Diabetes mellitus Erectile Dysfunction

There is little experimental evidence showing the long-term prevention of high BP. Running clinical trials to show the effectiveness of interventions in the early stages of high BP and, consequently, in decreasing outcomes is a major challenge, since it would require long intervention periods. Despite this limitation, the SPRINT-Mind trials showed that the strategy of lowering SBP to below 120 mm Hg was associated with the decreased incidence of mild cognitive impairment and dementia ^103^ as well as Alzheimer's markers in magnetic resonance imaging. ^104^

In addition to increased risk of clinical outcomes from high BP, there was also evidence of preclinical vascular and cardiac damage from BP readings lower than those traditionally used to diagnose HT. Cardiac consequences have also been found from mildly elevated blood pressure levels, categorized as prehypertension. ^105^ ^,^ ^106^ The PREVER trial found that, in these cases, lowering BP leads to smaller ECG-estimated ventricular mass, ^107^ as well as an almost 50% decrease in HT.

Risk estimates for increased incidence for the diseases listed in Chart 2.1 have increased significantly in recent years as lower BP values were included in mathematical models. The most conservative attribute 49% of infarctions and 62% of strokes to BP above 115/75 mm Hg. ^108^

Many justifications have been offered to explain centenarians' life spans. Curiously, even reports that found late onset of HT and CV events for these individuals ^109^ did not attribute a causal relationship between those conditions. Considering the discussion above, it is natural to conclude that vascular aging is not inexorable. ^102^ Therefore, it can be deduced that the key for very long life spans probably consists of maintaining BP at actually normal values. More recently, the 14-year follow-up of participants in the MESA (Multi-Ethinic Study of Atherosclerosis) study ^110^ with no other CV risk factors found that SBP above 100 mmGg increased the risk of CV events threefold compared to participants with SBP between 90 and 99 mm Hg.

The evidence available allow us to hypothesize that, in the future, reference values for a HT diagnosis, as well as therapeutic targets, may be changed and brought closer to levels we now consider normal or optimum BP. More solid and robust evidence is still needed before that change can occur, however.

## 2.3. Blood Pressure, Inflammation, and Endothelial Dysfunction

HT and its complications are mediated by various mechanisms sharing one common trait, ie, endothelial dysfunction, characterized by the low availability of nitric oxide (NO) and the consequent local imbalance between arteriole relaxing and contracting factors. ^111^ Endothelial dysfunction is a consequence of the imbalance between endothelial NO synthase (eNOS) or the transformation of NO into the free radical peroxynitrite (NOO^-^). ^112^ In that case, vasodilation mediated by several peptides, including bradykinin and angiotensin 1-7, is impaired, leading to increased peripheral vascular resistance and alterations in endothelial permeability. The onset of a chronic inflammatory state in HT patients via the increased production of proinflammatory cytokines (eg, leukocyte adhesion molecules such as endothelin-1 and angiotensin II) decreases eNOS expression, ^113^ ^-^ ^115^ while increased oxidative stress accelerates NO degradation. Reduced local NO availability increases smooth vascular muscle tone, induces smooth muscle cell proliferation in the medial layer, and increases endothelial permeability, facilitating the passage of low-density lipoprotein (LDL-c) into the subendothelial space, which seems to be the initial event behind the onset of atherosclerosis. Thus, endothelial dysfunction would be at the root of two chronic diseases that usually go together, ie, HT and atherosclerosis. Therefore, identifying the degree of endothelial dysfunction might be an important step in assessing the clinical course of HT. At the biochemical level, ultrasensitive C-reactive protein (CRP) seems to be the most adequate clinically available marker to assess endothelial dysfunction.

Currently, the most widely used technique for analyzing endothelial function in vivo in clinical settings is brachial artery FMD, ^116^ ^-^ ^119^ a noninvasive ultrasound method correlated with coronary endothelial function ^120^ ^,^ ^121^ and an independent predictor of CV disease. ^121^ ^,^ ^123^ However, its availability is limited. Endothelium-dependent dilation is a consequence of brachial artery relaxation in response to increased shear stress and local release of NO. ^119^ The association between FMD and CV prognosis is that it reflects the bioavailability of NO. ^124^ FMD may improve the predictive power of risk calculations based on traditional risk factors, including for young hypertensive patients. ^119^ ^,^ ^123^ Antihypertensive medications that increase the bioavailability of NO and statins may be an interesting option for clinical management ^5^ ^,^ ^37^ ^,^ ^125^ in order to maintain or preserve endothelial function for both asymptomatic patients and those with established CAD.

## 2.4. Blood Pressure and Arterial Stiffness

Assessments of vascular damage, a common finding in HT, are increasingly part of clinical practice. The damage involves microvascular alterations, atherosclerosis, increased arterial stiffness, and endothelial dysfunction. ^126^ There is probably a genetic component to arterial stiffness, ^127^ but there are also two other important determinants: age and BP levels. ^128^

Age has greater impact on proximal (central) arteries, predominantly elastic, than on peripheral arteries, predominantly muscular. Central arteries grow stiffer with age, while muscular arteries change less. With age comes elastin fragmentation and generation and progressive accumulation of collagen, accompanied by calcium deposits in the medial layer of arteries, and consequently increased arterial stiffness. ^128^ ^,^ ^129^

Sustained BP increases trigger the onset of arterial medial hypertrophy, as it causes quantitative and qualitative alterations in the components of the arterial walls (elastin, collagen, and smooth muscle cells), leading to mechanical adaptations. ^127^ ^,^ ^128^ ^,^ ^130^ These findings have been described both for animal models ^131^ and for in vitro studies and ex vivo organ cultures, ^132^ ^,^ ^133^ where mechanosensitive cells respond to increased stress with extracellular matrix production. Therefore, HT accelerates vascular aging, a local mechanobiological response to increased induced stress from higher BP and, consequently, greater arterial stiffness (stiffness as a consequence). ^128^ ^,^ ^134^

However, several studies have found increased carotid or aortic stiffness in normotensive individuals, despite normal BP levels. ^135^ ^-^ ^138^ Stiffer arteries create higher impedance for ventricular ejection, requiring higher blood pressure to keep blood flow constant. Thus, increased arterial stiffness may also lead to increased BP in the long run and, consequently, to CV risk. Studies have shown that arterial stiffness may precede HT, theorizing stiffness as a cause. Humphey et al. (2016) ^134^ described the mechanism as neither cause nor consequence alone, but rather as both, ie, a positive feedback loop where stiffness leads to HT and HT leads to stiffness.

The impact of HT on the medial layer of arteries may be assessed using biomarkers capable of detecting damage and various levels of impairment, determining impact on mortality, predicting CV events, adding information to known risk factors, sufficiently stratifying risk to change therapeutic recommendations, and adding information to justify additional costs. ^139^ Biomarkers available for assessing arterial stiffness can be found in the following subsections.

### 2.4.1. Ankle-Brachial Index (ABI)

ABI is the ratio between systolic pressure in the ankle and the arm, ^140^ considered a marker for arterial stiffness in patients without peripheral arterial disease. ^141^ It may be measured using Doppler ultrasound or simply applying the oscillometric method, cheaper and more easily available, and readings obtained using both techniques are strongly correlated. ^142^ According to a 2008 meta-analysis, ^143^ ABI ≤ 0.90 is associated with approximately twice the 10-year age-adjusted mortality, CV mortality, and higher rate of coronary events. Using ABI has led to the reclassification of CV risk categories and changing therapies for 19% of men and 36% of women. ^143^ ABI as a predictor of cardiovascular risk is LR: IIa, LE: B.

**Table:** 

	Level of recommendation	Level of evidence
Blood pressure above 120 mm Hg increases vascular damage and cardiovascular risk	I	A
Use of serum markers to identify endothelial dysfunction	IIb	B
Use of brachial artery FMD (the gold standard technique for in vivo endothelial function analysis) in identification of endothelial dysfunction	IIb	B
Use of FMD for cardiovascular risk stratification	IIb	B
Arterial stiffness assessed with PWV is an independent predictor of cardiovascular risk, and its assessment, when possible, may make the risk stratification more accurate	IIa	A
ABI is an independent predictor of cardiovascular risk	IIa	B
Central blood pressure is an independent predictor of cardiovascular risk	IIa	B

**Table:** 

Key Takeaways
Prehypertension increases cardiovascular risk.
Vascular damage is not found only in the hypertensive and may also be found in prehypertensives.
There are noninvasive tests to assess early vascular damage, but they are not always available.
Arterial stiffness analysis with PWV is an independent predictor of cardiovascular risk, and it may be assessed in clinical practice, when available.
Other methods, such as ABI and central blood pressure, may also be used to assess cardiovascular risk. FMD is more widely used in research settings.

### 2.4.2. Pulse Wave Velocity (PWV)

PWV is considered the gold standard for arterial stiffness assessments due both to how easy it is to obtain and to the large body of evidence showing its association with CV disease regardless of traditional risk factors. ^144^ ^,^ ^145^

Carotid-femoral PWV (cfPWV) is determined by dividing traveled distance by travel time (cfPWV = distance/time). The time may be measured directly in the same pulse wave or indirectly using an electrocardiogram. This noninvasive, robust and validated measure was standardized in an expert consensus document published by a European group in 2012. ^146^

Currently available validated methods include pulse tonometry ^147^ ^,^ ^148^ and piezoelectric ^149^ ^,^ ^150^ and oscillometric mechanotransducers. ^151^ ^,^ ^152^ In 2015, the American Heart Association published a position paper on standardizing the use of these devices to assess arterial stiffness. ^82^

Increased arterial stiffness is predictive of outcomes. This was shown for cfPWV in hypertensive patients in the early 2000s ^153^ ^,^ ^154^ and confirmed in several studies and two subsequent meta-analyses. ^155^ ^,^ ^156^ The first meta-analysis, from 2010, ^155^ included 15877 patients from 17 trials, and showed that, risk-adjusted for age, sex, and risk factors, a 1 m/s increase in PWV led to a 14% increase in CV events, 15% in CV mortality, and 15% in all-cause mortality. In addition, a one standard deviation increase was associated to increases of 47%, 47%, and 42%, respectively. The second meta-analysis, published in 2014, ^156^ featuring 17635 patients from 16 trials, found that, for every one standard deviation increase in PWV, the risk increased 35% for CAD, 54% for stroke, and 45% for CVD.

As well as predicting outcomes, adding PWV to traditional CV risk factors helps with stratification. The first study to show improved risk stratification from adding PWV to other CV risk factors was performed on a population sample from the Framingham cohort. ^157^ Later, the meta-analysis by Ben-Shlomo et al. (2014) ^156^ showed a 13% increase in risk prediction for individuals at intermediate risk when PWV was added.

Though PWV is relevant for event prediction and risk stratification, it is still little used in clinical practice. In 2019, a European group published a score ^158^ based on clinical variables to prioritize individuals for PWV assessments. The score assesses easily available clinical variables and is known by the acronym SAGE: S ( *systolic blood pressure* ), A ( *age* ), G ( *fasting plasma glucose* ), and E ( *estimated glomerular filtration rate* ). PWV increases can be predicted accurately from that score. Therefore, we can prioritize arterial stiffness assessments for select hypertensive patients, improving its deployment in clinical practice.

The cutoff value for normal PWV, in most studies and guidelines, is under 10 m/s. However, due to the influence of age on arterial stiffness, current proposed reference values take into account the various age ranges and sex, as established by the European group in 2010 ^144^ using tonometry, and more recently in a Brazilian study using oscillometric devices ( Table 2.1 ). ^159^ PWV as a predictor of cardiovascular risk is LR: IIa, LE: A.

**Table 2.1:** – Reference values for central systolic pressure and pulse wave velocity in Brazilian and European populations with and without cardiovascular risk factors69,84,85

	Normal population — no cardiovascular risk factors				Population with cardiovascular risk factors					Normal population — no cardiovascular risk factors			Population with cardiovascular risk factors				
	Brazilian ^1^		European^2^		Brazilian ^1^		European^2^			Brazilian ^1^		European^3^	Brazilian ^1^		European^3^		
CBP	Women	Men	Women	Men	Women	Men	Women	Men	PWV	Women	Men		Women	Men	High normal	Stage I HT	Stage II HT
**< 30 years**	101 (90-93-113-119)	113 (90-93-113-119)	95 (80-88-102-110)	103 (92-97-109-115)	118 (102-109-127-131)	123 (107-114-132-144)	101 (88-94-110-124)	110 (95-102-120-130)	< 30 years	4.9 (4.4-4.5-5.0-5.3)	5.2 (4.9-5.1-5.4-5.7)	6.1 (5.3-7.1)	5.3 (4.7-5.0-5.6-6.0)	5.3 (5.0-5.3-5.8-6.3)	6.7 (5.8-7.9)	7.2 (5.7-9.3)	7.6 (5.9-9.9)
**30-39 years old**	109 (96-102-117-123)	114 (96-102-117-123)	98 (84-90-108-119)	103 (88-05-112-120)	120 (102-110-130-143)	125 (108-116-133-141)	111 (92-100-127-141)	114 (95-103-129-144)	30-39 years old	5.4 (5.0-5.2-5.8-6.1)	5.7 (5.3-5.5-5.9-6.1)	6.4 (5.2-8.0)	5.8 (5.3-5.5-6.2-6.7)	6.1 (5.5-5.8-6.4-6.7)	7.0 (5.5-8.8)	7.2 (5.5-9.3)	7.6 (5.8-11.2)
**40-49 years old**	110 (99-103-117-122)	116 (99-103-117-122)	102 (87-93-113-123)	106 (90-97-114-123)	121 (104-110-134-146)	123 (108-115-131-141)	116 (95-104-133-146)	118 (97-106-132-144)	40-49 years old	6.4 (5.7-6.0-6.7-6.9)	6.5 (5.9-6.2-6.8-7.0)	6.9 (5.9-8.6)	6.8 (6.0-6.4-7.2-7.7)	6.8 (6.2-6.4-7.1-7.5)	7.7 (6.5-9.5)	8.1 (6.8-10.8)	9.2 (7.1-13.2)
**50- 59 years old**	110 (97-104-120-124)	112 (97-104-120-124)	110 (93-100-119-127)	110 (96-102-118-126)	124 (106-114-135-146	124 (105-114-134-144)	50-59 years old00-109-134-148)	123 (102-111-137-150)	60-69 years old	4.9 (4.4-4.5-5.0-5.3)	5.2 (4.9-5.1-5.4-5.7)	6.1 (5.3-7.1)	5.3 (4.7-5.0-5.6-6.0)	5.3 (5.0-5.3-5.8-6.3)	6.7 (5.8-7.9)	7.2 (5.7-9.3)	7.6 (5.9-9.9)
**60- 69 years old**	114 (100-103-121-126)	112 (100-105-120-125)	114 (97-105-122-129)	114 (97-105-122-128)	127 (105-115-141-154)	123 (103-112-136-149)	128 (105-115-141-154)	128 (105-115-142-155)	60-69 years old	8.9 (8.1-8.5-9.2-9.4)	8.9 (8.2-8.6-9.1-9.6)	9.7 (7.9-13.1)	9.3 (8.4-8.8-9.8-10.4)	9.2 (8.4-8.7-9.7-10.2)	9.8 (7.9-13.2)	10.7 (8.4-14.1)	12.0 (8.5-16.5)
**≥ 70 years**	113 (100-103-121-126)	116 (100-103-121-126)	118 (100-109-126-131)	116 (99-107-124-130)	131 (108-118-146-165)	125 (102-111-140-156)	138 (113-126-152-164)	135 (113-124-147-160)	70 years	11.3 (10.2-10.4-12.5-13.2)	11.0 (10.1-10.6-11.6-12.3)	10.6 (8.0-14.6)	11.8 (10.2-10.8-12.9-14.0)	11.2 (9.9-10.4-12.1-13.2)	11.2 (8.6-15.8)	12.7 (9.3-16.7)	13.5 (10.3-18.2)

### 2.4.3. Central Blood Pressure

Central (aortic, carotid) blood pressure does not correspond to peripheral (brachial) blood pressure due to pulse amplification from the aorta to the periphery; the former is more relevant for CV pathogenesis than the latter. ^160^ Currently, central blood pressure can be easily measured by noninvasive methods using the same equipment utilized and validated for measuring PWV. ^151^ ^,^ ^161^ ^,^ ^162^

Central hemodynamic indices are independent predictors of future CV events and all-cause mortality according to the meta-analysis by Vlachoupoulos et al., which included 11 studies and a total of 5648 individuals with a mean follow-up period of 45 months. ^160^ Central blood pressure reference values were established by the European group in 2014 using tonometry, ^163^ and more recently in a Brazilian study with oscillometric devices ( Table 2.1 ). ^159^ Central blood pressure as a predictor of cardiovascular risk is LR: IIa LE: B.

## 3. Diagnosis and Classification

## 3.1. Introduction

The initial assessment of a patient with hypertension (HT) comprises diagnostic confirmation, suspicion, and identification of the secondary cause, and assessment of cardiovascular (CV) risks. End-organ damage (EOD) and associated diseases should also be investigated. The assessment comprises blood pressure (BP) measurement in and/or out of the office, using proper techniques and validated and well calibrated equipment, taking the patient's medical history (personal and family), physical examination, and clinical and laboratory investigation. All hypertensive patients should undergo general assessments, in addition to complementary assessments for specific groups. ^164^

## 3.2. Blood Pressure Measurement at the Physician's Office

All medical assessments should include blood pressure measurements, whatever their specialty, and all other health care professionals should be properly trained in the process. Diagnosing HT and its phenotypes, as well as the management of the diagnosis, is the exclusive province of physicians.

Auscultatory or oscillometric sphygmomanometers are the preferred instruments for BP measurement. These devices should be validated according to standardized protocols and conditions, ^165^ and their calibration checked annually (for oscillometric devices) or every six months (for auscultatory devices), or following Inmetro/Ipem recommendations. ^166^ Initially, BP should be measured in both arms, preferably by simultaneous double arm measurement. If the difference between arms is > 15 mm Hg for SBP, there is increased CV risk, ^167^ which may be connected to atheromatous vascular disease. All subsequent measurements should be performed on the arm with the highest BP values. If HT secondary to coarctation of the aorta is suspected, blood pressure should also be measured in the lower limbs, using properly sized cuffs for arm or thigh circumference ( Chart 3.1 ). ^164^

**Chart 3.1:** – Cuff dimensions by limb circumference

Circumference	Cuff denomination	Cuff width	Bladder length
≤ 6 cm	Newborn	3 cm	6 cm
6-15 cm	Toddler	5 cm	15 cm
16-21 cm	Child	8 cm	21 cm
22-26 cm	Small adult	10 cm	24 cm
27-34 cm	Adult	13 cm	30 cm
35-44 cm	Large adult	16 cm	38 cm
45-52 cm	Thigh	20 cm	42 cm

Source: Malachias et al., 2017. ^164^

**Chart 3.2:** – Blood pressure measurement at the physician's office

The patient should seat comfortably in a quiet environment for 5 minutes before BP measurement can begin. Explain the procedure to the individual and instruct them not to talk during the measurement. Possible doubts should be clarified before or after the procedure.
Make sure the patient does or has NOT: • Have a fuller bladder; • Exercised within the last 60 minutes; • Had coffee or alcohol or eaten; • Smoked within the last 30 minutes.
Three BP measurements should be taken, at 1 to 2 minute intervals; additional measurements should only be performed if the first two readings differ by > 10 mm Hg. Record in the patient's chart the mean of the last two BP readings, without rounding it up or down, and the arm used for the measurement.
Additional measurements may have to be performed for patients with unstable BP due to heart arrhythmias. In patients with AF, auscultatory methods are preferable, since most automated devices have not been validated for BP measurement.^*^
Use properly sized cuffs for arm circumference.
The cuff should be positioned at heart level. The patient should have their palm up and their clothing should not compress their arm. Patients should have back and forearm supported, legs uncrossed, and feet planted on the ground.
Measure BP in both arms during the first visit, preferably both simultaneously, to detect possible differences between arms. The arm with the higher reading provides the reference value.
In investigating orthostatic hypotension, first measure BP (preferably in supine position, after the patient has been supine for 5 minutes; if the individual is unable to remain in supine position, the measurement may alternately be taken with the patient sitting, though that position is not ideal), then take additional BP readings 1 minute and 3 minutes after the person stands up. BP should measured at rest and standing for all patients in their first visit and also considered in subsequent visits for older adults, diabetes patients and dysautonomic patients, as well as those on any antihypertensive medication.
Record the heart rate. To rule out arrhythmia, use palpation of the pulse.
Inform the patient of the BP reading.

AF: atrial fibrillation; BP: blood pressure.^*^Most automated devices register the highest individual systolic blood pressure reading instead of averaging out several cardiac cycles for AF patients, leading BP to be overestimated.

In older adults, diabetic patients, dysautonomia patients, or individuals taking antihypertensive medications, BP should also be measured 1 minute and 3 minutes after standing up (motionless). ^168^ Orthostatic hypotension is defined as a SBP decrease ≥ 20 mm Hg or a DBP decrease PAD ≥ 10 mm Hg within the 3^rd^minute standing up and is associated with higher risk of mortality and cardiovascular events. ^169^

Charts 3.2 and 3.3 summarize the procedures and steps recommended for proper BP measurement. It should be stressed that improper BP measurements may lead to inaccurate classification, overestimating or underestimating the patient's true BP, and consequently to unnecessary treatment or lack of treatment for misassessed hypertensive patients. Given the simplicity of measuring BP by oscillometry, using a brachial oscillometric device may be preferable to auscultation when both techniques are available. ^170^ The differences between both techniques for measuring BP are highlighted in Chart 3.3 .

**Chart 3.3:** – Steps of blood pressure measurement

Steps
1. Measure arm circumference at the midpoint between the acromion and the olecranon.
2. Choose cuff sized to match the arm.
3. Place cuff snugly 2 to 3 cm from the cubital fossa.
4. Centralize the compressive part of the cuff on the brachial artery.
5. Estimate BP level based on palpation of the radial pulse.*
6. Palpate the brachial artery on the cubital fossa and place the stethoscope’s diaphragm without excessive compression;*
7. Inflate cuff rapidly until the estimated SBP level obtained on palpation is exceeded by 20-30 mm Hg;*
8. Proceed to deflate slowly (2 mm Hg per second).*
9. Determine SBP by auscultation of the first sound (Korotkoff phase I), then slightly increase the deflation velocity.*
10. Determine DBP when the sounds disappear (Korotkoff phase V).*.
11. Auscultate until 20-30 mm Hg below the last sound to confirm its disappearance, then proceed to rapid and complete deflation.*.
12. If heart beats persist until zero, determine DBP on the muffling of sounds (Korotkoff phase IV) and write down the values of SBP/DBP/zero.*

DBP: diastolic blood pressure; SBP: systolic blood pressure. * Items performed exclusively in the auscultatory technique

In the *Systolic Blood Pressure Intervention Trial* (SPRINT), ^86^ a new mode of measuring BP at the physician's office without an attending health care professional was used, known as unobserved automated office blood pressure measurement (UAOBPM). In this technique, after duly instructed, the patient measures their own blood pressure in a room set aside for that purpose. In SPRINT, participants followed a protocol where they waited in a quiet room for five minutes, then an automated device measured their BP three times, with one minute intervals, and recorded the readings. UAOBPM improves BP measurement reproducibility, and the white-coat effect may be significantly lowered or even eliminated. ^171^ ^,^ ^172^ In UAOBPM, readings are similar or lower than those obtained via ambulatory blood pressure monitoring (ABPM) or home blood pressure monitoring (HBPM). ^173^ However, one cannot forget that conventional office BP measurement is the basis for all currently available clinical and epidemiological data.

For obese individuals, optimum cuff size and shape to fit the patient's arm is critical. Proper cuff choice depends on both the circumference and the shape of the arm. ^174^ Longer and wider cuffs are required for measurements in these patients to avoid overestimating BP. The forearm approach should be considered valid and may be used in clinical settings for BP measurement when severe obesity makes measurement in the upper arm too challenging (arm circumference greater than 50 cm, for which there are no cuffs available). In these situations, the radial pulse should be auscultated, though there are limitations to that practice. ^175^ ^,^ ^176^ Cone-shaped, wide, short arms that do not fit large cuffs represent a particular challenge for BP measurement. The use of validated heart rate monitors should also be considered in these cases. ^177^ ^,^ ^178^

## 3.3. Classification

The BP limits considered normal are arbitrary. ^164^ ^,^ ^179^ The values used to classify BP in adults by using casual or office measurements are shown in Chart 3.4 . Individuals are considered hypertensives if SBP ≥ 140 mm Hg and/or DBP ≥ 90 mm Hg. When using office measurements, the hypertension diagnosis should always be validated with repeated readings, under ideal conditions, on at least two visits made days or weeks apart; or more assertively by using out-of-office measurements (ABPM or HBPM), except for patients already presenting with EOD or CV disease. ^37^ Patient classification follows the office BP measure and the highest BP level, either systolic or diastolic.

**Chart 3.4:** – Classification of blood pressure from in-office measurement, ages 18 and up.

Classification*	SBP (mm Hg)		DBP (mm Hg)
Optimum BP	< 120	and	< 80
Normal BP	120-129	and/or	80-84
Prehypertension	130-139	and/or	85-89
Stage 1 HT	140-159	and/or	90-99
Stage 2 HT	160-179	and/or	100-109
Stage 3 HT	≥ 180	and/or	≥ 110

BP: blood pressure; DBP: diastolic blood pressure; HT: hypertension; SBP: systolic blood pressure. * Classification follows office BP and the highest BP level, either systolic or diastolic. ** Isolated systolic HT, characterized by SBP ≥ 140 mm Hg and DBP < 90 mm Hg, is classified as 1, 2 or 3, according to SBP values at the intervals indicated. *** Isolated diastolic HT, characterized by SBP < 140 mm Hg and DBP ≥ 90 mm Hg, is classified as 1, 2 or 3, according to SBP values at the intervals indicated.

Individuals with SBP ≥ 140 mm Hg and DBP < 90 mm Hg are classified as having isolated systolic HT, while SBP < 140 mm Hg and DBP ≥ 90 mm Hg is characteristic of isolated diastolic HT. Both isolated systolic HT and isolated diastolic HT have higher rates of white-coat HT (WCH). ^180^

In the previous Brazilian guidelines, ^164^ what was then called normal BP is now known as optimum BP, while prehypertension is now divided into normal BP and prehypertension. Individuals with SBP from 130 to 139 and DBP from 85 to 89 mm Hg are now considered prehypertensive, since that population has shown a consistently higher risk of CV disease, coronary artery disease, and stroke than populations with BP between 120 and 129 or 80 and 84 mm Hg. They are also at greater risk of having masked hypertension (MH). ^181^ ^,^ ^182^ Consequently, prehypertensive individuals should be monitored closely.

## 3.4. Out-of-Office Blood Pressure Measurement

Out-of-office BP may be measured using ABPM or HBPM, following its indications and restrictions. ^183^ ^-^ ^187^ Out-of-office BP measurements should be encouraged. The major advantages and disadvantages of out-of-office BP measurement are summarized in Chart 3.5 , while its primary indications, as well as specific indications for HBPM, can be found in Chart 3.6 .

**Chart 3.5:** – Advantages and disadvantages of out-of-office blood pressure measurement

• Greater number of measurements • Reflects usual activities of patients • May identify white-coat HT and masked HT • Greater patient engagement with diagnosis and follow-up	
ABPM	HBPM
• Night readings • Allows measurement in real-life conditions • Use in patients with cognitive impairments and in rare cases of obsessive behavior • Allows short-term assessment of BP variability • More robust prognostic evidence	• Low cost and widely available • Measurement at home may be more relaxed than at the physician's office • Allows assessment of day-to-day BP variability • Patient engagement in BP measurement • Higher adherence to treatment
**• High cost** **• Potentially limited availability** **• May be uncomfortable**	**• Only BP at rest** **• Potential measurement error** **• No night reading**

ABPM: ambulatory blood pressure monitoring; BP: blood pressure; HBPM: home blood pressure monitoring; HT: hypertension.

**Chart 3.6:** – Indications for ABPM or HBPM

ABPM or HBPM
White-coat HT investigation is more frequent, particularly in the following situations: • Stage 1 HT at the physician's office • Very high BP at the physician's office in the absence of EOD
Investigating masked HT is more frequent, particularly in the following situations: • Prehypertension at the physician's office • Normal BP at the physician's office for patients with EOD or high-risk CV
Confirmation of resistant HT diagnosis
HT control assessment, especially for high CV risk patients
Individuals with exaggerated BP response to physical exercise
Presence of high variability in office BP
Assessment of symptoms of hypotension during treatment
Specific indications for ABPM:
BP assessment during sleep and/or wakefulness dip/dip (eg suspected noctunal hypertension, obstructive sleep apnea, chronic kidney disease, diabetes, endocrine HT, or autonomic dysfunction)
Investigation of postural and postprandial hypotension in treated and untreated patients

ABPM: ambulatory blood pressure monitoring; BP: blood pressure; DBP: diastolic blood pressure; HBPM: home blood pressure monitoring; HT: hypertension; SBP: systolic blood pressure.

ABPM and HBPM should not be mistaken for self-measured blood pressure (SMBP), performed by patients themselves using automated devices, which do not follow any pre-established protocol. The measurements are made at random, at the patient's discretion or as requested by the physician. ^188^

The COVID-19 pandemic has accelerated the development of telemedicine (televist, telecounseling, and telemonitoring), a change we believe to be irreversible. Currently, the Brazilian Unified Health System (SUS) already provides COVID-19 telecounseling, and supplementary health care services have already adopted it as well. Here, SMBP has the possibility of contributing to diagnosis, follow-up and treatment for hypertensive patients. To that end, this guideline recommends the use of high-quality oscillometric devices, ie, preferably brachial cuff-based devices that have been validated. Wrist blood pressure monitors should be discouraged, but where used, give preference to validated devices that include height and motion sensors. A minimum of seven measurements, performed during a 16- to 72-hour period, is recommended. Thus far, recommended normal values are the same as for HBPM, though specific studies and trials are still needed to compare BP values observed using each technique. ^187^ ^,^ ^189^

The definition of hypertension by BP at the physician's office is shown on Chart 3.7 . Compared to office BP readings, HBPM values are usually low, and the diagnostic threshold for BP is ≥ 130/80 mm Hg (equivalent to office BP ≥ 140/90 mm Hg). ^180^ ^,^ ^190^ ^-^ ^192^ HBPM offers more reproducible BP values and is more strongly related to EOD, particularly left ventricular hypertrophy, and to CV morbidity and mortality than office BP. ^188^ ^,^ ^193^ There is also evidence that HBPM may offer a beneficial effect in terms of adherence to medication and BP control, ^194^ ^,^ ^195^ especially when combined with guidance and counseling. ^196^ Telemonitoring and smartphone applications may offer additional advantages for HBPM, ^197^ ^,^ ^198^ such as BP measurement reminders and a convenient way to store and edit BP data in a digital report.

**Chart 3.7:** – Definition of home blood pressure monitoring according to office blood pressure, ambulatory blood pressure monitoring, and home blood pressure monitoring

Category	SBP (mm Hg)		DBP (mm Hg)
**Office BP**	≥ 140	and/or	≥ 90
**24-hour ABPM**	≥ 130	and/or	≥ 80
**Daytime**	≥ 135	and/or	≥ 85
**Sleep**	≥ 120	and/or	≥ 70
**HBPM**	≥ 130	and/or	≥ 80

ABPM: ambulatory blood pressure monitoring; BP: blood pressure; DBP: diastolic blood pressure; HBPM: home blood pressure monitoring; HT: hypertension; SBP: systolic blood pressure.

ABPM is a better predictor of CV risk and EOD than office BP. ^199^ In addition, 24-hour ambulatory BP means are better correlated with fatal or nonfatal events, ^200^ ^,^ ^201^ such as fatal and nonfatal coronary events and strokes. ^202^ ^-^ ^205^

## 3.5. White-Coat Effect (WCE) and Masking Effect (ME)

The difference in BP between measurements taken at the physician's office and out of it is known as WCE or ME, when the values are positive or negative, respectively. Based on HBPM trials, differences equal to or higher than 15 mm Hg for SBP and/or 9 mm Hg for DBP indicate significant WCE, while differences equal to or lower than -1 mm Hg for SBP and/or DBP indicate significant ME. ^180^ These scenarios do not change the diagnosis; ie, if the individual is normotensive, they remain normotensive, and if hypertensive, they remain hypertensive. However, it may be useful in identifying individuals at risk of significant BP differences at and out of the physician's office, which may contribute to better therapeutic management.

## 3.6. White Coat Hypertension (WCH) and Masked Hypertension (MH)

Several different phenotypes are possible for an HT diagnosis. True normotension (TNT) is defined as normal attended and unattended BP measurements, sustained HT (SHT) when both are abnormal, WCH when BP is high at the physician's office but normal outside it, and MH when BP is normal at the office, but high outside it. ^206^ ^,^ ^207^ Estimated prevalence rates in Brazil can be found in Figure 3.1 . ^208^ ^,^ ^209^

**Figure 1.3:** – Population prevalence of hypertension according to various diagnostic criteria, in adults 18 or older, both genders, by age group (Brazil, 2013).

Source: Nilson et al., 2020. ^29^

Though the prevalence varies between studies, WCH can be found in approximately 15 to 19% of individuals at the office and up to 30 to 40% of individuals with high BP at the office. It is more common among patients with stage 1 hypertension. ^210^ ^-^ ^212^

The presence of EOD and the risk of CV events associated with WCH are lower than in SHT. ^205^ ^,^ ^213^ ^,^ ^214^ However, compared to TNT, WCH is associated with higher adrenergic activity, higher prevalence of metabolic risk factors, more frequent EOD and higher of developing diabetes mellitus, and progressing to SHT and left ventricular hypertrophy. ^215^ ^,^ ^216^ In WCH, out-of-office BP values tend to be higher than in TNT, which might explain the increased long-term risk of CV events. ^217^ ^-^ ^221^

Like WCH, the prevalence of MH may vary significantly across populations. However, overall, MH may be found in approximately 7 to 8% of individuals at the physician's office, and may total circa 15% of normotensives. ^222^ ^,^ ^223^ Several factors can elevate out-of-office BP compared to office BP, such as being older, male, smoking, alcohol consumption, physical activity, exercise-induced hypertension, anxiety, stress, obesity, diabetes mellitus, chronic kidney disease, and family history of HT. MH is associated with dyslipidemia, dysglycemia , EOD, prehypertension, and adrenergic activity and increases the risk of progression to diabetes mellitus and SHT. ^183^ ^,^ ^185^ ^,^ ^198^ ^,^ ^207^ ^,^ ^224^ ^-^ ^226^ Meta-analyses of prospective studies report that the incidence of CV events is approximately twice as high in MH than in TNT, and comparable to that in HT. ^210^ ^,^ ^227^ ^,^ ^228^

## 3.7. Uncontrolled Masked and White Coat Hypertension

WCH and MH were originally defined for people who were not being treated for HT. However, patients on antihypertensive medications may also have divergent BP behaviors in and out of the physician's office. The following terms are used for patients treated with antihypertensives: uncontrolled masked HT, when BP is controlled at the office, but high out of it; uncontrolled white-coat HT, when BP is high at the office, but normal out of it; uncontrolled sustained HT, when BP is high at and out of the office; and controlled HT, when BP is normal at and out of the office. ^218^ Figure 3.2 shows the prevalence rates for these four phenotypes in Brazil. ^213^ ^,^ ^214^

**Figure 3.2:** – Fenótipos em hipertensos tratados.

MAPA: monitorização ambulatorial da pressão arterial; MRPA: monitorização residencial da pressão arterial.

## 3.8. Diagnosis and Follow-Up Recommendations

HT is a habitually asymptomatic condition. Therefore, it should be assessed during doctor's visits and as part of structured population-based screening programs. In the latter, over 50% of HT patients did not known they had the disease. ^229^ ^,^ ^230^ BP should be measured at regular intervals, with frequency determined by BP classification ( Figure 3.3 ). Healthy individuals with optimum office BP (< 120/80 mm Hg) or normal BP (120-129/80-84 mm Hg) should have their BP measured at least one a year and during medical appointments. Patients suffering from prehypertension (130-139/85-89 mm Hg) should have their BP measured annually, or preferably more often than that, due to the high rates of progression to HT. In addition, if MH is suspected, ABPM or HBPM should be deployed to investigate the phenotype.

**Figure 3.3:** – Screening and diagnosis of hypertension.

ABPM: ambulatory blood pressure monitoring; BP: blood pressure; HBPM: home blood pressure monitoring; MH: masked hypertension; SHT: sustained hypertension; TNT: true normotension; WCH: white coat hypertension.

Since BP may be highly variable, a diagnosis of HT should not be based exclusively on BP values at a single visit, unless it is significantly elevated (stage 3 HT) or there is an established diagnosis of EOD or CV disease. For other patients, repeated BP measurements in subsequent visits to the physician's office should be used to verify persistent high BP as well as to stage the disease. The higher the stage, the more frequent the appointments should be and the shorter the interval between them. Therefore, stages 2 and 3 patients may require more frequent visits (days or weeks apart), while stage 1 patients may require visits after a few months, especially when there is no EOD and CV risk is low.

The guideline recommends the use of out-of-office BP measurements ( Figure 3.3 ) as an alternate strategy for repeated office BP measurements to confirm the HT diagnosis, as long as they are logistically and economically feasible. ^231^ This approach may also generate relevant supplementary clinical information, such as detecting WCH and MH ^213^ ^,^ ^214^ ^,^ ^232^ ( Chart 3.6 and Figure 3.3 ).

The exercise stress test is not recommended for the diagnostic assessment of HT due to several limitations, including lack of standardization in methods and definitions. Currently, there is no consensus about normal BP response to physical exercise.

## 3.9. Central Aortic Pressure

Several techniques have enabled the measurement of aortic BP (central BP) using algorithms based on brachial BP readings. ^233^ ^,^ ^159^ Several studies show different reductions for central BP compared to brachial BP for select antihypertensive medications, and though central BP seems to be a better predictor of CV events than brachial BP, the added prognostic value of measuring central BP still requires more evidence. ^160^ ^,^ ^234^

Spurious HT (isolated systolic HT in young individuals with normal central BP) seems to be clearest case for use of central BP (when available) in clinical practice, making it the first indication for central BP measurement. It is found in a small fraction of young individuals, especially male athletes, but it remains unclear whether these patients are at lower CV risk than suggested by conventional BP measurements. ^235^ ^-^ ^237^

It should be stressed that the prognostic limitations of central BP are not applicable to other parameters associated with said measurements, such as pulse wave velocity (PWV) and augmentation index (AIx), which have well-established prognostic values. ^238^

## 3.10. Genetics and Hypertension

Primary hypertension is a multifactorial, but with a strong genetic component. Family and twin studies have shown 30 to 50% heritability levels. ^239^ ^,^ ^240^ Most of the genetic risk is polygenic, ie, it comes from the contribution of hundreds of DNA variants that, taken together and after interacting with the environment, increase the risk of developing a hypertensive phenotype. A recent study of over 1 million patients showed that DNA variations in over 900 genes are associated with BP control, explaining approximately 27% of the heritability of BP control. ^240^ The study paves the way for the use of gene panels to assess HT risk, which might help guide preventive efforts.

In contrast with primary HT, various forms of secondary HT are caused by heritable single-gene mutations (monogenic HT), such as familial hyperaldosteronism, Liddle's syndrome, congenital adrenal hyperplasia, and hereditary pheochromocytoma and paraganglioma ( Chart 3.8 ). ^240^ ^,^ ^241^ These causes should be investigated in patients suspected of secondary HT. Genetic diagnoses need to take into consideration proper treatment as well as allow genetic counseling for families and early screening for asymptomatic family members.

**Table:** 

Recommendation	LR	LE
BP should be classified as optimum, normal, prehypertension or stages 1 to 3, depending on BP measurement at the physician's office.	I	C
HT screening programs are recommended. All adults (≥ 18 years old) should have their BP measured at the physician's office, have their values recorded in their files, and be made aware of their BP. ^160^ ^,^ ^234^	I	B
Given the simplicity of measuring BP by oscillometry, using an automated brachial oscillometric device may be preferable to auscultation when both techniques are available.	I	C
Annual BP measurement is indicated if the office BP is < 140/90 mm Hg.	I	C
It is recommended that BP be measured in both arms, at least on the first visit, since differences in SBP greater than 15 mm Hg across arms might suggest atheromatous disease and is associated with increased CV risk. ^167^	I	A
If a difference in BP < 15mm Hg is found, it is recommended that all subsequent BP readings use the arms with the highest BP value.	I	C
It is recommended that the HT diagnosis be based on repeated office BP measurements, on multiple visits, except for stage 3 HT and especially for high-risk patients. Three BP measurements should be taken at each appointment, at 1 to 2 minute intervals; additional measurements should only be performed if the first two readings differ by > 10 mm Hg. The patient's BP is the mean of the last two BP readings.	I	C
It is recommended that the HT diagnosis be based on out-of-office BP measurements using ABPM and/or HBPM, as long as these measurement techniques are feasible (logistically and economically).	I	C
Out-of-office BP (ie, ABPM or HBPM) is specifically recommended for various clinical indications, such as identifying WCH and MH, quantifying treatment effects and identifying possible causes of side effects (eg, symptomatic hypotension). ^164^ ^,^ ^170^ ^,^ ^180^ ^,^ ^2^ 01,209	I	A
Pulse pressure, BP variability and central BP may be considered, but are currently little used in routine clinical practice. They may provide useful additional information in certain circumstances and stand as valuable research instruments.	IIb	C
Genetic testing should be considered in specialized centers for patients suspected of rare monogenic causes of secondary HT or for those with pheochromocytoma. ^240^ ^-^ ^242^	IIa	B
Routine genetic testing for hypertensive patients is not recommended.	III	C

*ABPM: ambulatory blood pressure monitoring; BP: blood pressure; CV: cardiovascular; HBPM: home blood pressure monitoring; MH: masked hypertension; WCH: white coat hypertension.*

**Table:** 

Key Takeaways
BP should be classified as optimum, normal, prehypertension or stages 1 to 3, depending on office BP.
HT screening programs are recommended. All adults (≥ 18 years old) should have their BP measured at the physician's office, have their values recorded in their files, and be made aware of their BP.
Annual BP measurement is indicated if the office BP is < 140/90 mm Hg.
It is recommended that the HT diagnosis be based on repeated office BP measurements, on multiple visits, or on out-of-office BP measured by ABPM and/or HBPM when either or both are feasible.
Out-of-office BP (ie, ABPM or HBPM) is specifically recommended for various clinical indications, such as identifying WCH and MH, quantifying treatment effects and identifying possible causes of side effects (eg, symptomatic anemia hypotension).

**Chart 3.8:** – Causes of monogenic hypertension

Condition	Mode of inheritance	Genes involved
**Liddle's Syndrome**	Autosomal dominant	*SCNN1B and SCNN1G*
**Congenital adrenal hyperplasia**	Autosomal recessive	*CYP11B1*
	Autosomal recessive	*CYP17A1*
**Apparent mineralocorticoid excess syndrome**	Autosomal recessive	*HSD11B2*
**Geller Syndrome**	Autosomal dominant	*NR3C2*
**Gordon syndrome (pseudohypoaldosteronism type II)**	Autosomal dominant	*WNK4*
	Autosomal dominant	*WNK1*
	Autosomal recessive or dominant	*KLHL3*
	Autosomal dominant	*CUL3*
**Familial hyperaldosteronism type I**	Autosomal dominant	*CYP11B1*
**Familial hyperaldosteronism type II**	Autosomal dominant	*CLCN2*
**Familial hyperaldosteronism type III**	Autosomal dominant	*KCNJ5*
**Familial hyperaldosteronism type IV**	Autosomal dominant	*CACNA1H*

## 4. Clinical and Complementary Assessment

## 4.1. Clinical History

The clinical assessment of hypertensive patients should follow the traditional methodology, consisting of taking their hypertensive patient, physical examination, and laboratory tests. Chart 4.1 summarizes the objectives. Following every step of the process will enable physicians to correctly diagnose hypertension (HT) and stratify cardiovascular and renal risks, contributing to a more adequate therapeutic strategy.

**Chart 4.1:** – Clinical and laboratory assessment

Perform accurate BP measurements for diagnostic confirmation of HT (Chapter 2)
Ask about family history of HT
Identify associated renal and cardiovascular risk factors
Investigate EOD (subclinical or clinically manifested)
Investigate presence of other diseases
Ask about drugs and medications that may interfere with BP
Apply global CV risk score (Chapter 5)
Screen for signs of secondary HT (Chapter 15)

*BP: blood pressure; CV: cardiovascular; EOD: end-organ damage; HT: hypertension.*

## 4.2. Clinical Assessment

### 4.2.1. History-Taking

A full patient history should be taken, including mandatory questions about timing of diagnosis and prior antihypertensive treatments (medications and dosage). In addition, symptoms indicating the progress of hypertension, especially the presence of end-organ damage (EOD), should be investigated. It is also important to establish the patient's personal history and to build a timeline to better understand their clinical condition.

Family histories should also be taken to corroborate a diagnosis of primary HT ^243^ (LR: I; LE: B). During the appointment, the patient should be asked, among others, about the presence of specific cardiovascular disease (CVD) and kidney disease risk factors, ^244^ ^-^ ^246^ comorbidities and biopsychosocial, cultural, and socioeconomic aspects. ^244^ ^,^ ^245^ ^,^ ^247^ Assessing the use of other nonantihypertensive medications, whether legal or illegal drugs, that might interfere with BP (Chapter 9) is critical, as is investigating the patient's clinical history for signs suggesting secondary causes of HT, as detailed in Chapter 15.

## 4.3. Physical Examination

A detailed physical examination should be performed, with proper and repeated BP and heart rate (HR) measurements, as described in Chapter 3, as well as the search for signs of EOD and findings that might suggest secondary causes of HT.

Anthropometric data, such as weight, height, as well as body mass index (BMI) calculation, ^248^ and abdominal circumference (AC) , ^248^ have normal reference values defined by the *World Obesity Federation* (available online at https://www.worldobesity.org/ . The assessment should include palpation and auscultation of the heart, carotid arteries, and pulses. Measuring the ankle-brachial index (ABI) is also encouraged, as is fundoscopy. ^249^ ^,^ ^250^ To calculate ABI, divide brachial systolic blood pressure (SBP) by ankle SBP for both the left and the right side. The normal arm/ankle SBP ratio is higher than 0.90. Mild obstruction is characterized by ABI from 0.71 to 0.90; moderate, from 0.41 to 0.70; and severe, from 0.00 to 0.40 (LR: IIa, LE: B). It is an important tool for diagnosing peripheral occlusive atherosclerotic disease and to determine the prognosis for cardiovascular events. ^250^

In some cases, measuring central blood pressure (CBP) in order to detect isolated systolic hypertension in young individuals (spurious hypertension of youth) may be recommended, since, unlike brachial artery readings, CBP is not high in these situations (LR: IIa, LE: B) (Chapter 3). ^251^ ^,^ ^252^ Chart 4.2 shows a summary of the physical examination.

**Chart 4.2:** – Physical examination assessment

1. Take repeated, accurate BP measurements in both arms (see Chapter 3)
2. Measure anthropometric parameters: weight, height, HR, AC, and BMI.
3. Look for signs of end-organ damage
4. Investigate signs of endocrine disease, such as Cushing's syndrome and hyper or hypothyroidism
5. Examine cervical region: palpation and auscultation of carotid arteries, check of jugular stasis, and thyroid palpation
6. Cardiovascular system assessment: displaced apex beat and propulsion to palpation; in auscultation, presence of S3 or S4, hyperphonetic second heart sound, murmurs, and arrhythmias
7. Assess respiratory system: listen for rales, rhonchi, and wheezing
8. Observe extremities: edemas, upper and lower limb pulses (decreased femoral pulse suggests coarctation of the aorta, aortic disease or aortic arch disease)
9. Abdominal palpation and auscultation: fremitus, bruit, abdominal masses suggestive of polycystic kidney disease and tumors (may suggest secondary causes or EOD)
10. Detect sensory and motor deficits in neurological examination
11. Perform fundoscopy or retinography (when available0: identify increased dorsal reflex, arterioral narrowing, pathological arteriovenous crossings, hemorrhages, exudates, and papilledema (signs of hypertensive retinopathy)

*AC: abdominal circumference; BMI: body mass index; BP: blood pressure; HR: heart rate.*

### 4.3.1. Basic Laboratory Investigation, Assessment of Subclinical and Clinical End-Organ Damage

The goal of complementary assessment is to detect subclinical or clinical end-organ damage to better stratify cardiovascular (CV) risk. To stratify global CV risk, in addition to classical risk factors ( Chart 4.3 ), other, recently-identified risk factors should also be considered, although not all have been incorporated into clinical scores. ^253^ ^,^ ^254^ Important elements in this investigations include altered glycemia or glycated hemoglobin, abdominal obesity (metabolic syndrome), pulse pressure > 65 mm Hg in older adults, history of pre-eclampsia/eclampsia, and family history of HT (for borderline hypertensive patients). ^254^

**Chart 4.3:** – Additional cardiovascular risk factors

Age (women > 65 years old and men > 55 years old)
Smoking
Dyslipidemia: fasting triglycerides (TG) > 150 mg/dL; LDL-c > 100 mg/dL; HDL-c < 40 mg/dL
Confirmed diabetes mellitus (DM) (fasting plasma glucose of at least 8 hours ≥ 126 mg/dL, random blood glucose ≥ 200 mg/dL or HbA1c ≥ 6.5%) or pre-diabetes (fasting plasma glucose from 100 to 125 mg/dL or HbA1c from 5.7 to 6.4%)
Family history of premature CVD: in women < 65 and men < 55
Pulse pressure in older adults (PP = SBP – DBP) > 65 mm Hg
Abnormal ABI or PWV
Past pathological history of pre-eclampsia or eclampsia
Central obesity: BMI < 24.9 Kg/m ^2^ (normal); from 25 to 29.9 Kg/m ^2^ (overweight); > 30 Kg/m ^2^ (obesity)
Waist-hip ratio (WHR)
Abdominal circumference = women < 88 cm and men < 102 cm Waist: C = at the midpoint between the last rib and the iliac crest Hip H = at the level of the greater trochanter Calculation (WHR) = women: WHR ≤ 0.85; men: WHR ≤ 0.95
Metabolic syndrome profile

ABI: ankle-brachial index; BMI: body mass index; H: hip; PP: pulse pressure; W: waist; WHR: waist-hip ratio.

Changes in pulse wave velocity (PWV), when available, are indicative of EOD, and may reclassify intermediate CV risk patients as high-risk (LR: IIa, LE: A) (Chapter 2). ^156^ Basic laboratory assessment ( Chart 4.4 and 4.5 ) should be part of the initial routine for all hypertensive patients. ^253^ The recommended tests are serum potassium, uric acid, creatinine, glycemia, and lipid profile, as well as urinalysis and an electrocardiogram for possible left ventricular hypertrophy.

**Chart 4.4:** – Routine complementary examinations

Urinalysis (LR: I, LE: C)
Plasma potassium (LR: I, LE: C)
Plasma creatinine (LR: I, LE: B)
Fasting plasma glucose (LR: I, LE: C) and HbA1c (LR: I, LE: C)
Estimated glomerular filtration rate (LR: I, LE: B)
Total cholesterol, HDL-C and serum triglycerides (LR: I, LE: C) *
Plasma uric acid (LR: I, LE: C)
Conventional electrocardiogram (LR: I, LE: B) **

* LDL-C is calculated using the following formula: LDL-C = total cholesterol - (HDL-C + triglycerides/5) (when triglycerides < 400 mg/dL). ^259^ * LDL-C may also be measured in routine laboratory work. ** Sokolow-Lyon criteria for LVH: SV_1_+ RV_5.6_> 35 mm – Cornell voltage: RaVL + SV_3_> 20 mm (women), > 28 mm (men). ^260^ ^,^ ^261^

**Chart 4.5:** – Recommended tests for indicated populations.

Chest X-ray: indicated for follow-up of hypertensive patient in case of clinical suspicion of cardiac involvement (LR: IIa, LE: C) and/or pulmonary involvement and to assess hypertensive patients with aortic involvement for whom an echocardiogram is not available. ^262^
Echocardiogram: more sensitive than an electrocardiogram to diagnose left ventricular hypertrophy (LVH), adds value in the assessment of the geometry of left atrial hypertrophy and size of the left atrium, as well as analysis of systolic and diastolic function. Indicated when there are signs of LVH in the electrocardiogram or for patients with clinical suspicion of heart failure (LR: IIa, LE: B). LVH is diagnosed when the left ventricular mass adjusted for body surface area is equal to or greater than 116 g/m ^2^ for men and 96 g/m ^2^ for women. ^263^
Albuminuria or urine protein/creatinine ratio or urine albumin/creatinine ratio: useful test for diabetic hypertensive patients, patients with metabolic syndrome or two or more risk factors, since it is predictive of fatal and nonfatal cardiovascular events (normal values < 30 mg/g of creatinine (LR: I, LE: B). ^264^
Carotid ultrasound: indicated in the presence of carotid bruit, signs of cerebrovascular disease, or presence of atherosclerotic disease in other areas. Increased carotid intima-media thickness (IMT) and/or the identification of atherosclerosis plaque formation is predictive of stroke and myocardial infarction, regardless of other CV risk factors. IMT values > 0.9 mm are considered abnormal, as is the finding atherosclerotic plaques (LR: I, LE: A). ^265^ ^,^ ^266^
Doppler or non-Doppler renal ultrasound: necessary for patients with abdominal masses or abdominal murmur (LR: IIa, LE: B). ^267^
Glycated hemoglobin (HbA1c): indicated when fasting plasma glucose is higher than 99 mg/dL, family history or previous diagnosis of type 2 DM and obesity (LR: IIa, LE: B). ^268^
Exercise stress test: indicated in suspected stable coronary disease, diabetes mellitus or family history of coronary disease in patients with controlled blood pressure (LR: IIa, LE: C). ^269^
ABPM/HBPM: see indications for methods in Chapter 3 (LR: I, LE: A). ^186^
Pulse wave velocity (PWV) measurement, when available: indicated for low- to moderate-risk hypertensives, considered a useful method to assess arterial stiffness, ie, vascular damage. PWV values higher than 10m/s are considered abnormal for the general population, but there are adjusted reference values for age and sex deciles available (LR: IIa, LE: A). ^139^ ^,^ ^270^ ^,^ ^271^
Brain magnetic nuclear resonance (MNR): indicated in patients with cognitive disorders and dementia to detect silent cerebral infarcts and microbleeds (LR: IIa, LE: C). ^272^

To assess renal function, one should obtain the estimated glomerular filtration rate using the *Modification of Diet in Renal Diseases* (MDRD) ^255^ formula or, preferably, the *Chronic Kidney Diseases Epidemiology Collaboration* (CKD-EPI) ^256^ formula, available at http://mdrd.com/.

Figure 4.1 shows the estimated glomerular filtration rate (eGFR) accompanied by staging (stages 1 through 5) and prognosis for chronic kidney disease and considering albuminuria levels, according to the *Kidney Diseases Improving Global Outcomes* (KDIGO) guidelines. ^257^ ^,^ ^258^ The colors indicate renal prognosis and management. Green means low risk and good prognosis; yellow, intermediate risk, patient should be monitored; orange, high risk, poor prognosis, mandatory referral to specialist; red, very high risk, poor prognosis, mandatory referral to specialist.

**Figure 3.1:** – Possible diagnoses in hypertension (phenotypes).

ABPM: ambulatory blood pressure monitoring; HBPM: home blood pressure monitoring.

**Figure 4.1:** – Prognosis for chronic kidney disease by glomerular filtration rate and albuminuria.

CKD: Chronic kidney disease, eGFR: estimated glomerular filtration rate; KDIGO: Kidney Diseases Improving Global Outcomes.

In terms of renal assessment:

It is recommended that the clinical laboratory make the creatinine test results available along with the eGFR results (LR: I, LE: B);

Creatinine clearance results (24 h urine) are not recommended, except for significant changes in muscle mass (amputation), body surface at the extremities and clinical instability (LR: I, LE: B);

It is recommended that the urine protein to albumin ratio be analyzed, in order of importance: urine albumin to creatinine ratio (ACR), urine protein to creatinine ratio (PCR), protein urine test strips with automated or manual reading. It is recommended that clinical laboratories report ACR and PCR for all urine samples and not just their concentrations (LR: I, LE: B).

**Table:** 

Key Takeaways
A full medical history and physical examination should always include proper BP measurement, analysis of anthropometric parameters and investigation of signs and symptoms of end-organ impairment and secondary causes of hypertension.
In hypertensive patients, it is important to investigate comorbidities (diabetes mellitus, dyslipidemia, and kidney and thyroid disease, among others) to improve CV risk stratification and treatment.
The routine supplementary tests recommended in this guideline are basic, easily available and easy to interpret, low-cost and mandatory for all patients, at least in their first visit and once a year. Other tests are required for indicated populations.
Investigating end-organ damage, both clinical and subclinical, is essential for fuller therapeutic guidance.

## 5. Cardiovascular Risk Stratification

## 5.1. Introduction

There is a widely established causal, linear, and continuous relation between increased blood pressure (BP) and risk of cardiovascular disease (CVD) for both sexes, all ages, and all ethnic groups. ^85^ BP interacts synergically with other CVD risk factors (RFs), and its pro-atherogenic effect is proportional to the number and intensity of these additional factors. ^78^ ^,^ ^273^ CVD is a multifactorial condition, dependent on synergic interactions in the whole causal system responsible for its development. In addition, modest increases in several RFs may trigger greater increases in cardiovascular (CV) risk than sharp increases in a single RF. ^273^ ^,^ ^274^ Therefore, quantifying risk for hypertensive patients, ie, the probability of a given individual developing CVD during a given period, is an essential part of the process and can help guide preventive and treatment strategies. ^37^ ^,^ ^164^ ^,^ ^275^

It should be stressed that the impact of hypertension (HT) control is proportional to the absolute individual risk and to the global estimated risk. ^276^ ^-^ ^278^ It is noteworthy that the concept of residual risk represents the magnitude of the risk remaining after traditional risk factors are under control. ^279^ Partial RF control and/or late onset of effective therapeutic measures may be key elements for residual CV risk in hypertensive patients. Despite the lack of tools for the identification of residual risk, it is clear that early and accurate RF control is essential. ^279^ Thus, a comprehensive approach to all RFs is fully justified. To that end, when identified, hypertensive patients should be told about risk factors potentially subject to change in order to improve the efficacy of both pharmacological and nonpharmacological measures. ^37^ ^,^ ^164^ ^,^ ^275^

CV risk should not be estimated intuitively or by simply adding up the RFs observed, but rather through the application of methods that take into consideration its complex and multifactorial nature. ^4^ The process should be based on equations or algorithms, ^4^ ^,^ ^280^ ^,^ ^281^ instruments that estimate risk based on multivariate regression models based on population-level studies and are recommended by multiple guidelines worldwide. ^4^ ^,^ ^280^ ^,^ ^281^ Even experienced physicians make mistakes in over 50% of cases when estimating risk without the aid of equations or algorithms. ^282^

However, one cannot forget the lack of Brazilian population data for these risk estimate models, making them less accurate for assessing CV in Brazil. In other words, international scores may underestimate risk by failing to consider the most prevalent or relevant RFs in Brazil. To mitigate that limitation and prevent the underdiagnosis of high-risk patients, this guideline recommends the identification of other markers, known as risk-modifying factors, to reassess risk in individuals classified as being at moderate risk (see details below).

CV risk classification depends on BP levels, associated CVRFs, presence of end-organ damage (EOD), structural and/or functional damage from HT to vessels, heart, brain, kidneys, and retina, and/or presence of established CVD or kidney disease. ^37^ ^,^ ^164^ ^,^ ^275^ Different scoring systems have been developed and applied to classify hypertensives as low, moderate and high CV risk patients. ^37^ ^,^ ^164^ ^,^ ^275^ However, new biomarkers, precursors, and predictors still seem necessary to improve risk prediction and lower the difference between calculated risk and event rates, especially in individuals classified as being at moderate risk. ^283^ ^-^ ^285^ Adding relative versus lifetime risk assessment tools risk advancement periods throughout the life cycle, especially for assessing young individuals at low absolute risk but high relative risk of CVD, as well as in older adults, for whom risk estimates are still challenging due to rapid changes in life expectancy and function in latter years. ^285^

## 5.2. Additional Risk Stratification (Associated Conditions)

Several factors known to, by themselves, determine or accelerate the onset of CVD, regardless of BP values, can coexist or add to each other in most hypertensive patients. ^37^ ^,^ ^275^ ^,^ ^286^ ^-^ ^288^ Associated conditions, whether from their prevalence in the general population or the strength of their association with CV events, should be identified in hypertensive patients by their clinical history, physical examination, and complementary testing. ^37^ ^,^ ^164^ ^,^ ^275^ Therefore, the examination should look for: a) coexisting RFs in HT ( Chart 5.1 ); b) EOD ( Chart 5.2 ); c) presence of established kidney disease and CVD.

**Chart 5.1:** – Coexisting risk factors for hypertension

Male
Age: > 55 years for men and > 65 years for women
Premature CVD in 1st degree relatives (men < 55 years old and women < 65 years old)
Smoking
Dyslipidemia: LDL cholesterol ≥100 mg/dL and/or non-HDL cholesterol 130 mg/dL and/or HDL cholesterol ≤ 40 mg/dL for men and ≤ 46 mg/dL for women and/or TG >150 mg/dL
Diabetes mellitus
Obesity (BMI ≥ 30 kg/m ^2^ )

**Chart 5.2:** – End-organ damage

**Left ventricular hypertrophy**
ECG (Sokolow-Lyon index (SV1 + RV5 or RV6) ≥ 35 mm; RaVL > 11 mm; Cornell voltage > 2440 mm.ms or Cornell index > 28 mm in men and > 20 mm in women (LR: I, LE: B)
ECHO: LVMI ≥ 116 g/m ^2^ in men or ≥ 96 g/m ^2^ in women (LR: IIa, LE: B)
ABI < 0.9 GR (LR: IIa, LE: B)
Stage 3 chronic kidney disease (eGFR between 30 and 60 mL/min/1.73m ^2^ )
Albuminuria from 30 to 300 mg/24 h or urinary albumin to creatinine ratio of 30 to 300 mg/g (LR: I, LE: B)
Carotid-femoral PWV > 10 m/s (LR: IIa, LE: A)

ABI: ankle-brachial index; ECG: electrocardiogram; ECHO: echocardiogram; eGFR: estimated glomerular filtration rate; LVMI: left ventricular mass index; PWV: pulse wave velocity.

Identifying these conditions is important both to develop an estimate of the risk to which the hypertensive patient is exposed in their current stage and to make sure that controllable RFs will be targeted by adequate therapeutic interventions, as intensive as their degree of risk requires. ^37^ ^,^ ^164^ ^,^ ^275^ The most prevalent and most easily identifiable RFs should be prioritized, as well as those for which there is solid evidence of association with CV risk ( Chart 5.1 ).

The factors listed in Chart 5.1 need to be taken into accounting when estimating CV risk, in accordance with current diagnostic criteria. Age is linearly correlated with HT and risk of CV complications, such as myocardial infarction (AMI) and stroke, and that linearity is more evident for strokes. For AMI, the level of association rises sharply with sex, beginning at age 55 for men and 65 for women. ^287^ Smoking, quantified by pack-year load, secondhand smoking and other forms of tobacco use, such as cigars, pipes, and vapes, are considered central elements for CV risk. Dyslipidemia is characterized by increased low-density lipoprotein (LDL) or the atherogenic lipoprotein profile obtained by subtracting high-density lipoprotein (HDL) from total cholesterol, ie, non-HDL cholesterol. HDL cholesterol levels are still indicated for risk estimates and has separate thresholds for men and women. High triglycerides (TG) are also characteristic of dyslipidemia, particularly when associated with decreased HDL cholesterol or at levels above 500 mg/dL; in this case, there is indication for specific treatment and pancreatitis should be considered. The criteria for diagnosing diabetes mellitus are fasting plasma glucose > 126 mg/dL; glycated hemoglobin > 6.5%, measured by high-performance liquid chromatography (HPLC); or glucose > 200mg/dL 2 h after oral glucose overload in oral glucose tolerance test or random blood glucose. Obesity should be considered when body mass index (BMI) is > 30kg/m ^2^ or when abdominal circumference (AC) is > 80 cm in women or > 94 cm in men of European or African descent or > 90 cm in men of Asian descent. ^289^

### 5.2.1. End-Organ Damage

Risk estimates for hypertensive patients should be supplemented with the investigation of EOD, which is frequent, often underdiagnosed, and usually not included in risk stratification scores. They cause additional increases in CV risk, especially when coexisting in a single individual ^5^ ^,^ ^6^ ^,^ ^7^ ( Chart 5.2 ).

### 5.2.2. Presence of Cardiovascular and Renal Disease

The presence of documented cerebrovascular and renal disease determines the increased risk of CV events in hypertensive patients. ^37^ ^,^ ^164^ ^,^ ^275^ ^,^ ^290^ ^-^ ^292^ Cerebrovascular disease should be considered in case of ischemic stroke, brain hemorrhage, or transient ischemic attack. Coronary artery disease includes angina, AMI, silent myocardial ischemia, myocardial revascularization surgery, or prior coronary interventions. Heart failure (HF) with preserved ejection fraction (pEF) or reduced ejection fraction (rEF) should also be considered in manifested cardiovascular disease, ^86^ ^,^ ^275^ as well as atrial fibrillation (AF). ^293^ Likewise, symptomatic peripheral occlusive atherosclerotic disease (POAD) ^294^ and aortic disease related with aneurysms, hematomas, or ulcerations represent cardiovascular manifestations with major impact on global CV risk. Because of its close association with CV risk, stage 4 or higher chronic kidney disease should be considered an indicator of high risk, identified by estimated glomerular filtration rate (eGFR) < 30 mL/min/1.73m ^2^ , urine albumin/creatinine ratio in an isolated sample (> 300 mg/g creatinine); and urine protein/creatinine in an isolated sample (> 300 mg/g creatinine). Finally, retinopathy attributable to the hypertensive process, such as hemorrhages, exudates, and papilledema, also indicates high risk. ^37^ ^,^ ^275^

Chart 5.3 shows the main reasons for performing risk estimates, taking into consideration, in addition to blood pressure levels, the presence of coexisting cardiovascular risk factors, EOD, and kidney and/or cardiovascular disease. These include establishing a reasonably accurate prognostic estimate and distinguishing cases requiring more intensive therapeutic regimens. ^37^ ^,^ ^275^

**Chart 5.3:** – Reasons to perform risk assessments (LR: I LE: C)

Estimating medium- and long-term risk of cardiovascular events
Determining health care level, such as frequency of service
Determining early timing of pharmacological treatment onset
Determining intensity of control of modifiable risk factors

Chart 5.4 is especially important for risk stratification for hypertensive patients. It is worth considering that the RFs mentioned above are only those with established epidemiological value, easily obtainable in most clinical settings, and with proven prognostic value. It helps us understand how the progression of risks associated with the presence of different BP levels and presence of RFs, EOD, or cardiovascular and/or kidney disease impact middle-aged individuals.

**Chart 5.4:** – Hypertension staging by BP level, presence of CVRFs, EOD, or comorbidities

RR, presence of EOD or disease	BP (mm Hg)			
	Prehypertension SBP 130-139 DBP 85-89	Stage 1 SBP 140-159 DBP 90-99	Stage 2 SBP 160-179 DBP 100-109	Stage 3 SBP > 180 DBP > 110
No RF	No additional risk	Low risk	Moderate risk	High Risk
1 or 2 RFs	Low risk	Moderate risk	High Risk	High Risk
≥ 3 RFs	Moderate risk	High Risk	High Risk	High Risk
EOD, stage 3 CVD, DM, CVD	High Risk	High Risk	High Risk	High Risk

*BP: blood pressure; CKD: chronic kidney disease; CVD: cardiovascular disease; DBP: diastolic blood pressure; DM: diabetes mellitus; EOD: end-organ damage; RF: risk factor; SBP: systolic blood pressure.*

In moderate-risk patients, tests are recommended when feasible and available, but never in an overgeneralized process, to identify subclinical EOD markers in order to make risk estimates more accurate. ^82^ ^,^ ^295^ ^,^ ^296^ Echocardiograms to assess LVH, which also record ventricular function parameters, and left ventricular mass index (LVMI) are important (LR: IIa, LE: B), as is albuminuria, preferably in the urine albumin/creatinine ratio, and ABI calculation (LR: IIa, LE: B). Vascular biomarkers added more recently to clinical practice, such as carotid-femoral pulse wave velocity (cfPWV), may also contribute for the reclassification of CV risk for these individuals (LR: IIa, LE: A), ^296^ ^,^ ^297^ as well as establish a worse prognosis for patients with established CV disease. ^139^ ^,^ ^298^ This aspect is particularly important for young individuals, who have lower relative risk and for whom there is the opportunity to prevent irreversible structural and functional damage to the arterial walls.

Other conditions are also known to influence or change CV risk for hypertensive patients classified as at moderate risk, but have lower discriminative power. ^37^ ^,^ ^275^ Its identification is not recommended for the identification of high-risk patients or for those without any of the coexisting risk factors discussed above ( Chart 5.5 ).

**Chart 5.5:** – Factors modifying risks for hypertensive patients

Family history or parental history of early-onset hypertension
Very high individual RF, including stage 3 HT
Prior eclampsia/pre-eclampsia
Sleep apnea
Pulse pressure > 60 (in older patients)
Uric acid > 7 mg/dL (men) and > 5.7 mg/dL (women) (LR: I, LE: C)
High-sensitivity C-reactive protein > 2mg/L (LR: I, LE: B)
HR > 80 bpm
Metabolic syndrome*
Sedentary lifestyle
Psychosocial and economic factors
Chronic inflammatory disease

HR: heart rate; HT: hypertension; RF: risk factor. * According to the criteria established by the International Diabetes Federation (IDF), metabolic syndrome requires central obesity, defined as abdominal circumference > 80 cm in women or > 94 cm in men of European or African descent or > 90 cm in men of Asian descent, as well as any two of the following four factors: triglycerides > 150 mg/dL, low HDL-C (< 40 mg/dL in men and < 50 mg/dL in women), hypertension; fasting plasma glucose ≥ 100 mg/dL or previously diagnosed type 2 diabetes mellitus. ^289^

## 5.3. Assessment of Global Cardiovascular Risk

Global CV risk stratification is not specific for hypertensive patients, and its objective is to determine the risk of a given individual ages 30 to 74 to develop CVD in general over the next 10 years. ^3^ ^,^ ^4^ ^,^ ^280^ ^,^ ^281^ ^,^ ^299^ It should be stressed that the relative impact of HT is one of the highest for any CVRFs, and therefore used in all global risk estimate equations. In addition, CV risk cannot be effectively mitigated without taking into consideration all the elements that determine the clinical course of hypertensive patients. A quick and practical way of calculating global CV risk is to use the Cardiovascular Risk Stratification Calculator recommended and provided by the SBC Atherosclerosis Department on its website, available for Android and iOS devices. ^3^

## 5.4. Challenges of Cardiovascular Risk Assessment in Hypertension

Several clinical conditions can impact CV risk stratification for hypertensive patients, and age is one of the most important. In the short run, while older patients have higher absolute risk, the young have lower absolute risk even when they have an unfavorable risk profile. ^37^ In the long run or for their lifetime risk, the influence of age on CV risk flips, and longevity loss becomes highest for those who had RFs when young.

Another limitation is the duration of exposure to the disease or RF. Scoring instruments that use binary (yes or no) choices for clinical conditions such as DM and smoking to assess cardiovascular risk do not take into consideration the duration of these conditions. Therefore, patients with longer exposure times have higher CV risk compared to individuals exposed to the same factors for shorter periods. ^37^ This means that incorporating new instruments to assess relative versus lifetime risk and periods of risk increase seem to be necessary, especially for young adults with low absolute risk but high relative risk for CVD. ^280^ ^,^ ^299^ The concepts of “vascular age” and “cardiometabolic age” can be helpful in that strategy. ^300^ ^,^ ^301^

The influence of the duration of antihypertensive treatment can also impact the risk estimate. In hypertensive patient who have recently started treatment, blood pressure before onset of treatment should be considered, while in patients who have been in treatment for longer periods, current blood pressure readings should be used. ^37^

The timing and value of BP readings to be considered in CV risk stratification and different HT phenotypes should also be taken into account. Therefore, out-of-office BP measurements are increasingly encouraged. Home measurements, ambulatory blood pressure monitoring (ABPM), and home blood pressure monitoring (HBPM) have led to important complementary information, though office BP readings are still the reference for diagnosis and to assess treatment efficacy. ^37^ ^,^ ^164^ ^,^ ^302^ To that end, the recommendation for ABPM has been widened to include confirming the diagnosis of HT, considering the higher correlation of ABPM readings with EOD and cardiovascular morbidity and mortality compared to casual BP measurements. ^303^ Likewise, different HT patterns, such as masked hypertension, isolated systolic hypertension, and absence of nighttime dipping, or even increased BP during sleep, also seem to lead to different degrees of cardiovascular risk. ^304^ ^,^ ^305^ Thus, these limitations should be taken into consideration when customizing CV risk estimates for hypertensive patients in clinical practice.

**Table:** 

Key Takeaways
Over 50% of hypertensive patients have additional CVRFs.
The presence of one or more additional CVRFs increases the risk of coronary, cerebrovascular, renal, and peripheral artery disease in hypertensive patients.
Identifying additional RFs should be part of diagnostic assessments of hypertensive patients, especially when there is family history of CVD.
CV risk should be estimated for all hypertensive patients using a simple scoring system, based on BP levels and the presence of additional RFs and comorbidities ( Chart 5.1 ).
CV risk can be estimated practically and reliably by identifying RFs, such as age > 65, sex (men > women), heart rate (> 80 bpm), increased body weight, diabetes mellitus, high LDL-c, family history of CVD, family history of SHT, smoking, and psychosocial and/or socioeconomic factors; for EOD: presence of LVH, moderate to severe CKD (eGFR < 60 mL/min/1.73m ^2^ ) or other assessment confirming presence of EOD and prior diseases: CAD, HF, stroke, POAD, AF, and stage 3 or higher CKD.

AF: atrial fibrillation; CKD: chronic kidney disease; CV: cardiovascular; CVD: cardiovascular disease; EOD: end-organ damage; eGFR: estimated glomerular filtration rate; GRS: global risk score; HF: heart failure; LVH: left ventricular hypertrophy; POAD: peripheral occlusive atherosclerotic disease; RF: risk factor.

## 6. Therapeutic Decision and Targets

## 6.1. Introduction

One of the specific goals of treatment for hypertensive patients is to achieve blood pressure control by hitting a previously established blood-pressure (BP) target. That target should be defined on an individual basis, and always take into account age and presence of cardiovascular disease (CVD) or risk factors (RFs). In general, BP decreases should target BP levels below 140/90 mm Hg, but not lower than 120/70 mm Hg (LR: I, LE: A). In younger individuals without RFs, lower targets, with values below 130/80 mm Hg, are achievable.

## 6.2. Low- or Moderate-Risk Hypertensive Patients

Cardiovascular (CV) risk estimates are extremely important in hypertensive patients, as they determine possible differences in BP targets. Hypertensive patients with few additional FR should be assessed from two perspectives: hypertensives with significantly high blood pressure levels without other RFs (stage 2 hypertension: moderate risk) and those with smaller BP increases (stage 1 hypertension: low risk).

The benefits of treating hypertensive patients without other associated CV risk factors with significantly high BP readings (> 160 mm Hg) are well established and have long been systematically recommended by Brazilian and international guidelines. ^5^ ^,^ ^37^ ^,^ ^164^ On the other hand, there is too little scientific evidence from randomized trials justifying treatment for stage 1 hypertensive patients with low CV risk. The reason is that the large number of participants and long follow-up period required mean that a controlled randomized trial with participants with those characteristics would be infeasible. Therefore, meta-analyses of individual data from participants in randomized trials with stage 1 hypertensive patients with no prior CVD can help us determine the best course of action. ^306^ ^-^ ^308^ One such study found that treating low-risk hypertensive patients did not lead to a decrease in coronary artery disease (CAD) outcomes, CV events, or CV mortality in a four- to five-year follow-up period. ^306^ There was, however, a trend of low stroke and total mortality rates, with both decreases clearly achieved as follow-up times grew longer or as more patients were added to the studies. A second meta-analyses, including approximately 9 000 participants from five randomized trials, found that lowering systolic blood pressure (SBP) by 7 mm Hg with pharmacological treatment led to a 34% decrease in composite outcomes (CAD and stroke) and a 19% decrease in all-cause mortality. ^307^ A third study found lower CV disease and mortality when the initial BP was equal to or higher than 140/90 mm Hg, and the same result was not found for lower initial values. ^308^ All these outcomes are supported by a subgroup analysis from the Heart Outcomes Prevention Evaluation (HOPE)-3 trial. In that study, even if stage 1 hypertensive patients were classified as having intermediate CV risk, antihypertensive treatment with a 6 mm Hg mean decrease in SBP led to a 27% decrease in major CV events. ^309^ Based on this date, pharmacological treatment can be initiated for stage 1 hypertensive patient with low cardiovascular risk, combined with nonpharmacological treatment (LR: I, LE: A).

In terms of blood-pressure targets for low CV risk hypertensive patients, there is also too little specific data from randomized trials. A recent meta-analysis and data from a large observational study suggest that blood-pressure targets below 140/90 mm Hg should be set and achieved for these patients, with larger decreases in CV outcomes attained from SBP readings between 120 and 130mm Hg. ^85^ ^,^ ^310^ Therefore, for these patients, targets below 140/90 mm Hg are recommended, and closer to 120/80 mm Hg, if tolerated ( Chart 6.1 ) (LR: I, LE: B).

**Chart 6.1:** – General blood-pressure targets for antihypertensive treatment

Target	Cardiovascular risk	
	Low or moderate	High
Systolic blood pressure (mm Hg)	< 140	120-129
Diastolic blood pressure (mm Hg)	< 90	70-79

## 6.3. High-Risk Hypertensive Patients

In general, hypertensive patients with three or more RFs, diabetic patients, those with end-organ damage (EOD), CV disease, or kidney disease are considered to be at high risk. In clinical practice, the most frequent examples of individuals with high CV risk are hypertensive patients with CAD, prior history of stroke, heart failure and chronic renal failure (CRF), and HT associated with diabetes mellitus (DM). These comorbidities are discussed in Chapter 10 ( *Associated Clinical Conditions* ), but the targets for each of these clinical situations are discussed below. Keep in mind that high risk depends not only on RFs and EOD, but also on HT staging, as shown in Chart 5.4 , and a patient may have RF or EOD but also have stage 3 HT ( Chart 6.1 ).

## 6.4. Hypertensive Patients with Coronary Disease

HT is an important independent RF for the onset of myocardial ischemia. Before age 50, diastolic BP (DBP) is the main predictor for CAD risk, while SBP is more important after age 60. ^311^ In older populations, DBP is inversely related with CAD risk, and pulse pressure becomes a stronger predictor of CAD. ^311^ In a meta-analysis including nearly 1 million adults, fatal CAD was correlated with BP levels equal to and higher than 115/75 mm Hg for all ages. ^78^ In that case, antihypertensive treatment for CAD patients should result in BP < 130/80 mm Hg, but no lower than 120/70 mm Hg. In patients with evidence of myocardial ischemia, DBP should be cautiously lowered to 70 mm Hg, especially in diabetic patients and in the very old. ^312^ Lowering SBP in older patients with CAD and high pulse pressure requires great care, since they may lead to very low DBP values and trigger myocardial ischemia. ^313^

## 6.5. Hypertensive Patients with History of Stroke

HT is the most important RF for ischemic and hemorrhagic stroke and is directly related to blood pressure levels. In younger individuals with no history of established CV or renal disease, keeping BP within the normal or optimum range, targeting a BP level of 120/80 mm Hg, may be the most effective for of primary prevention for cerebrovascular disease. For those with one or more prior strokes, the most adequate target for secondary prevention should be assessed according to type of stroke and post-event time (Table 10.2). In chronic cases of secondary prevention, keeping SBP from 120 to 130 mm Hg is recommended (LR: I, LE: A). ^314^ For older adults or those with associated coronary disease, a relatively common scenario, the J-curve phenomenon should be taken into consideration when BP falls below 120/70 mm Hg, with higher risk of CV events and mortality. ^315^

## 6.6. Hypertensive Heart Failure Patients

Hypertension is considered a risk factor for both forms of heart failure, ie, with reduced ejection fraction and with preserved ejection fraction. Adequate treatment for HT lowers the incidence of HF. No clinical trials specifically for the HF population have compared different treatment goals. Therefore, recommendations are extrapolated from the evidence from other high-risk populations, for which lowering BP has been shown to be most protective against CV events, though with potentially increased side effects. In patients already suffering from HF with reduced ejection fraction, blood pressure control decreases mortality and readmission rates for cardiac decompensation. The proper target for this population is < 130/80 mm Hg, but taking care to keep it above 120/70 mm Hg. ^316^ In patients with preserved ejection fraction, the best form of treatment remains uncertain, so the recommended treatment strategy is similar to that for patients with reduced ejection fraction. ^317^ ^,^ ^318^

## 6.7. Hypertensive Patients with Chronic Kidney Disease (CKD)

Most CKD patients have high BP, which increases the risk of CV diseases, CKD, and death. The Systolic Blood Pressure Intervention Trial (SPRINT) study concluded that systolic BP < 120 mm Hg lowered the risk of CV disease and mortality for nondiabetic adults with high CV risk, many of whom have CKD. However, it could not halt the progression of CKD. ^319^ Notoriously, in that study, BP was measured using automated devices, frequently unattended, which usually results in lower readings than office BP measurements. ^320^ ^,^ ^321^ The absolute decrease in risk may be greater for patients with albuminuria due to the strong association between albuminuria and kidney and CV disease, but the effects of intensive decreases in BP on the risk of CVD seem to be similar by albuminuria level. ^322^ ^,^ ^323^ Current evidence indicates a BP target of < 130/80 mm Hg for CKD patients, regardless of DM. ^275^ ^,^ ^324^ ^,^ ^325^ In patients with end-stage CKD, the benefits of intensive BP control are uncertain due to their short duration and to hemodynamic effects possibly leading to greater decreases in glomerular filtration rate (GFR). Regardless of targets, BP decreases in CKD patients always requires attention to proper BP measurements and monitoring adverse events, especially electrolyte abnormalities and decreases in GFR. ^325^

## 6.8. Diabetic Hypertensive Patients

In hypertensive DM patients, morbidity and mortality prevention comes from glycemic control, BP normalization, and lowering other CV risk factors. ^326^ Keeping BP under control is keep for renal protection in diabetic individuals, as it lowers albuminuria, in addition to its importance in lowering the risk of stroke and left ventricular hypertrophy (LVH). ^327^ ^,^ ^328^ Evidence from randomized controlled trials, meta-analyses and observational studies with hypertensive diabetes patients shows that lowering SBP to 130-139 mm Hg, with values closer to the lower bound of 130 mm Hg, effectively protects against CV and renal complications. ^307^ ^,^ ^329^ DBP may be lowered to 70-79 mm Hg without compromising individual protection and safety. On the other hand, there is no conclusive data indicating that lowering SBP to < 130 mm Hg leads to higher CV and renal protection. SBP values < 120 mm Hg should be avoided. Therefore, for diabetic patients, the recommended target is < 130/80 mm Hg (LR: IIa, LE: B).

Achieving a lower SBP target implies the need for a larger number of antihypertensive medications, increasing the risk of severe adverse effects. ^330^ In practice, ideal BP targets can vary across diabetic hypertensive patients by age and presence of EOD. For instance, there is no data available for recent-onset diabetes patients with no complications and, therefore, relatively low CV and renal risks. In these cases, very low blood pressure levels may be more easily tolerated and result in greater medium- and long-term benefits. Overall, BP control is harder for patients with diabetes than for patients without diabetes. In addition, diabetic hypertensive patients often have satisfactory office BP levels, but high ABPM or HBPM readings, characterizing masked hypertension. This reinforces the need for out-of-office BP measurement in order to better assess BP control in diabetic hypertensive patients. ^331^

## 6.9. Older Hypertensive Patients

The complexities of older hypertensive patients are discussed in Chapter 14. BP targets for older populations should consider functional status, frailty and comorbidities in addition to chronological age (≥ 60 in low-income countries and ≥ 65 in all others, according to most international associations). ^37^ ^,^ ^275^ Therefore, the therapeutic goal should balance the potential benefits and harm from BP targets.

In most clinical trials that show the benefit of treating BP in older patients, the SBP target ranged from 140 to 150 mm Hg, with greater decreases in deaths and CV events. ^37^ ^,^ ^332^ In the HYVET trial, which included active and nonfrail patients over 80 years old, in addition to lowering SBP < 150 mm Hg (mean: 144 mm Hg), there were significant decreases in mortality, fatal strokes, and HF. ^333^ In a meta-analysis and systematic review of nine studies, the authors found robust evidence that decreases < 150/90 mm Hg lower mortality and fewer strokes and cardiac events in older adults. ^334^ However, recent clinical trials have found evidence for benefits from lower BP targets for older patients. ^87^ ^,^ ^335^ In the SPRINT trial subgroup consisting of individuals over age 75, ^87^ the intensive treatment group that achieved a mean target BP level of 124/62 mm Hg had significant decreases in CV events and HF as well as all-cause mortality compared to the group with a less intensive target, for which the average BP level achieved was 135/67 mm Hg. That study suggests that more intensive treatment may be beneficial even for frailer older adults, but the incidence of falls was higher, as was the incidence of impaired kidney function, in the more intensive blood pressure control group. Another relevant data point from the SPRINT trial is that BP measurements were unsupervised, and these readings tend to be lower than those obtained with conventional methods. Therefore, the target achieved is equivalent to BP values from 130 to 139 mm Hg when compared to readings from previous studies. ^321^ In another meta-analysis, Bavish et al. ^335^ showed that more aggressive BP control for patients ≥ 65 years old led to greater decreases in CV events, but it has several methodological restrictions and found greater rates of renal failure in the more intensive control group.

Overall, recommended targets for Brazilian patients ≥ 60 years old is to achieve levels matching their global condition (healthy or frail), as shown in both Chart 6.2 and Chapter 14. In older adults, targets should be treated individually, taking into account patients' quality of life, risk of falls, frailty, independence, and presence of comorbidities.

**Table:** 

Key Takeaways
In low to moderate CV risk hypertensive patients, the treatment goal is to achieve values below 140/90 mm Hg.
In hypertensives with CAD, the therapeutic target is to have BP < 130/80 mm Hg, but diastolic BP should be kept above 70 mm Hg.
For hypertensive patients with HF or who have had a stroke, antihypertensive treatment should be titrated until achieving the target of BP < 130/80 mm Hg, but the presence of CAD and advanced age, both common in that scenario, limits the ability to lower BP down to 120/70 mm Hg.
In hypertensive patients with CKD, the treatment goal is BP < 130/80 mm Hg, but always monitoring patients for adverse events, especially impaired kidney function and electrolyte abnormalities.
Hypertension treatment in diabetic patients should try to keep BP levels < 130/80 mm Hg, but avoid sharp decreases in BP to levels below 120/70 mm Hg.

**Chart 6.2:** – Treatment goals for older adults considering global condition and office blood pressure measurement.

	Office SBP		Office DBP	
Global condition ^1^	Treatment threshold	Blood-pressure target ^4^ ^,^ ^5^	Treatment threshold	Target ^8^
Healthy ^2^	≥140 (I, A)	130-139 (I, A) ^6^	≥90	70-79
Frail older adults ^3^	≥160 (I, C)	140-149 (I, C) ^7^	≥90	70-79

*1: functional status is more important than chronological age; 2: including light frailty; 3: moderate to severe frailty; 4: including older adults with comorbidities: DM, CAD, CKD, stroke/TIA (not acute stage); 5: actively assess tolerability, including possible atypical symptoms; 6: stricter target (125-135 mm Hg) may be achieved in selected cases, especially for motivated older adults, < 80 years old, with optimum treatment tolerability; 7: higher limits in case of limited survival and absence of symptoms. BP reductions should be gradual; 8: DBP = avoid < 65-70 mm Hg in clinically manifested CAD patients.*

## 7. Multidisciplinary Team

## 7.1. The Importance of a Multidisciplinary Approach to Hypertension Control

Uncontrolled hypertension (HT) remains a widely prevalent cardiovascular risk factor (CVRF) in Brazil and throughout the world. Various Brazilian and international studies have consistently shown the superiority of blood pressure (BP) control using a multidisciplinary team approach when compared to conventional treatment, including higher quality care, higher adherence and therapeutic success rates, fewer CVRFs, and lower CV morbidity and mortality. ^336^ ^-^ ^341^ Shared patient care and decision-making are associated with lower costs and better results in HT treatment. ^342^ ^,^ ^343^

Different objectives require different strategies, including patient-centered care, integration between multiple professionals, shared goals and targets, and collaborative decision-making with patient participation. ^344^ A Brazilian retrospective longitudinal study with the goal of evaluating the effect of multidisciplinary care in hypertensive patients age 80 and older (n = 71), treated by a specialized service for an average of 15.2 years, found lower BP values, increased BP control rates and treatment optimization. ^345^

A systematic review of 80 U.S. studies from 1980 to 2012 showed the efficacy of team-based care. There was a 12% increase in BP control rates, with median systolic BP (SBP) decrease of -5.4 mm Hg and median diastolic BP (DBP) decrease of -1.8 mm Hg, especially when the team included nurses and pharmacists. These results were found in various multidisciplinary setups and for various American population groups. ^346^

A multidisciplinary team is established through actions that integrate the contributions from all of its members. The spatial element is not the single determinant of this unit; more important is the practice of developing joint actions, where each discipline acts as an independent agent in its own realm, but always acknowledging and collaborating with the actions of other team members.

Multidisciplinary work has been used successfully in primary ^347^ , secondary, ^336^ and tertiary ^341^ health care settings. The high complexity of professional activities may drive them away from joint efforts. On the other hand, it is in health promotion and in the level of primary care that we find the greatest potential for integration and where team performance is most effectively embodied. Teamwork has advantages such as encouraging patients to reproduce knowledge and attitudes, favoring research activities in care, and providing opportunities for growth for team members and, consequently, for the institutions themselves. ^337^ ^,^ ^343^ ^,^ ^348^ ^-^ ^350^

Deploying a multidisciplinary approach requires organizational change at the health care system level, also important in home care settings. ^336^ A multidisciplinary approach to HT has level of recommendation I and level of evidence A ( Chart 7.1 ). ^351^ Therefore, health care becomes a fundamentally collective and complex form of work, requiring interdisciplinarity and multidisciplinarity. ^341^ ^,^ ^347^ ^,^ ^352^ Some duties and responsibilities are shared by all team members, others are specific to each role (LR: I, LE: A).

**Chart 7.1:** – Strategies for patient-centered multidisciplinary teams

Strategies	Description	Examples	Team Member
Patient Education	Educational or interactive approach to inform and educate patients	In-person educational sessions ^389^ In-person print material ^389^ ^,^ ^390^ Mailed print material ^390^ Audiovisual media and Distan^ce^education ^391^	PHY, NUR, PHARM, NUT, PSY, CHW
Social support	Engagement of family members, friends, or other individuals to help patients take medications as prescribed	Support group meetings ^347^ ^,^ ^350^ Fami^ly^education ^348^	FAM, FR, CT, CHW, SS
Patient literacy and motivation	Motivating patients to take their medication as prescribed and eliminate obstacles that negatively impact that motivation	Motivational interventions ^386^ ^,^ ^389^ Implement health literacy initiatives ^362^ ^,^ ^392^ ^,^ ^393^	PHY, NUR, NUT, PSY PE, PT, FR, CT, FAM
BP self-monitoring and technology use	Engage patients in monitoring BP and adhering to treatment	Self-measured BP ^391^ Home BP monitoring ^394^ ^,^ ^395^ BP telemonitoring ^343^ ^,^ ^390^ ^,^ ^396^ ^,^ ^397^	PHY, NUR, PAT, FAM, CT, CHW
Communication or interaction with service providers and among team members	Improving communication between patients and multidisciplinary team and other service providers and among team members	Training communication skills between patients and multidisciplinary team and among team members ^390^ Interactive digital interventions ^350^ ^,^ ^358^ ^,^ ^359^ ^,^ ^398^	PHY, NUR, NUT, PE, PT, PST, SS. CHW
Facilitating access to health care services	Facilitating scheduling of appointments at times compatible with patient needs	Patients from out of town Older adults depending on accompaniment by third parties ^345^ ^,^ ^399^	CHW, SS

*CHW: community health worker; CT: caretaker; FAM: family member; FR: Friend; NUR: nurse; NUT: nutritionist; PE: physical education professional; PHARM: pharmacist; PHY: physician; PSY: psychologist; PT: physical therapist; SS: social worker; PAT: patient. Source: Peacock & Krousel-Wood, 2016.^*344*^*

## 7.2. Team Composition and Work

### 7.2.1. Medical Professional: Specific Actions

General practitioners are involved in primary care, while cardiologists, nephrologists, and hypertension specialists are present at the other levels. The activities specific to physicians are as follows: ^341^

• Medical visit (detailed in Chapter 4).
• Responsible for diagnosis, risk stratification, and prescription of pharmacological and nonpharmacological therapy.
• Clinical assessment of patients at least twice a year.
• Referral and counter-referral within the health care system.

In addition to physicians, professionals from various health care disciplines (nurses, pharmacists, social workers, nutritionists, physical education professionals, physical therapists, educators, psychologists) have established multidisciplinary teams to provide care for hypertensive patients for several decades in developed countries. ^353^ ^,^ ^354^

### 7.2.2. Nursing Professional: Specific Actions

The activities specific to nurses are as follows:

• Performing patient intake, identifying alongside users the various obstacles and barriers in their daily lives, and encouraging the coping process.
• Enabling people to increase their control over factors impacting self-care, improving their health. Advanced communication skills, behavior change techniques, patient education, and counseling skills are key elements to improve and enhance health care systems, necessary to help patients with chronic conditions. ^355^ The Brazilian Ministry of Health stresses that developing actions focused on health promotion and prevention of noncommunicable disease (NCD), especially HT and diabetes mellitus (DM), is an enormous challenge. ^356^
• Encouraging self-care.
• Planning strategies to promote and assess patient adherence to prescribed behaviors using educational, motivational, cognitive and technological approaches. ^344^ ^,^ ^357^ ^-^ ^361^
• Promoting educational health-literacy initiatives for users. ^344^ ^,^ ^362^
• Home visits to reinforce medication use and help manage care and/or technologies to promote proper use, such as helping users establish medication intake habits and routines. ^363^ ^,^ ^364^

#### 7.2.2.1. Nursing-Specific Actions in Primary Care

Teams working in basic care should try to follow the principle of “person-centered care,” in which individuals are the main agents of personalized care. Professionals should help users develop the knowledge, aptitudes, competences and trust needed to more effectively manage and make informed decisions about their own health. Management plans are designed for people, according to their needs and their possibilities to pursue a full and independent lifestyle. To that end, the Ministry of Health published Ordinance 2.436, dated September 21, 2017, ^365^ establishing guidelines for the actions and activities of nursing staff, such as:

• Providing health care services to the individuals and families serve by the team and, when indicated or required, at homes and/or other community spaces (schools, associations, etc.) at every step of patients' life cycles.
• Performing nursing visits and procedures, requesting supplementary tests and prescribing medication in accordance with protocol, clinical and therapeutic guidelines and other technical standards, as established by federal, state, municipal or Federal District managers, in accordance with legislation and regulations.
• Performing and/or supervising patient intake with qualified listening and risk classification, following established procedures.
• Performing risk stratification and developing a care plan for individuals with chronic conditions in their territory alongside other team members.
• Performing group activities and referring users to other services, when necessary, following the flow rules established by the local health care network.
• Planning, managing and assessing the actions of nursing technicians and assistants, community health workers (CHW), and endemic disease control agents together with other team members. ^365^
• Supervising the actions of nursing technicians/assistants and CHW.
• Implementing and updating routines, protocols, and flows connected to their area of expertise at their primary health care unit.
• Performing other duties under their purview in accordance with legislation.

## 7.2.3. Nutrition Professional: Specific Actions

A recent meta-analysis ^366^ showed that nutritional counseling is more effective in lowering BP when provided by a multidisciplinary team including a nutritionist. In primary care, dietetic consultations were found to be the most effective in improving diet quality. ^367^

### *7.2.3.1. Dietetic Consultation* ***367,368***

Visits to nutritionists should include the following items:

• Nutritional history with an assessment of eating routine, number of meals, meal times, types and quantity of food, and frequency of cardioprotective foods.
• Anthropometric assessment: height, weight, and abdominal circumference measurement and body mass index calculation.
• Prescribe and guide diet based on medical diagnosis and laboratory examinations.
• Monitor diet changes and anthropometric evolution.
• Take part in actions involving the population.

### 7.2.3.2. Collective Actions by Nutritionists

The following actions are recommended:

• Nutritional guidance should center of impactful changes in BP reduction: weight loss, ^369^ ^,^ ^370^ increased intake of fruits and vegetables, ^371^ ^-^ ^373^ and lower sodium intake. ^374^ ^,^ ^375^
• Currently, the use of free technological resources in nutrition represent an important large-scale information resource and should be encouraged. ^376^ ^,^ ^377^

## 7.2.4. Physical Education Professional: Specific Actions

Sedentary behavior (time sitting, watching TV or at the cellphone or computer) and physical inactivity (physical activity habits below recommended levels) represent a major public health issue, as they increase treatment costs and lower life expectancy. ^378^ ^-^ ^380^ Physical education professionals have the task of applying the recommendations found in Chapter 8 to minimize these behaviors. To that end, the professional should:

• Recommend less sedentary behavior in adult and adolescent populations.
• Encourage meeting minimum physical activity (PA) recommendations for the whole population through the collective actions detailed below. Practicing these activities contributes to lowering cardiovascular mortality even in case of sedentary behavior. ^381^
• Plan, teach and supervise physical exercise (PE) programs, in person or at a distance, individually or in groups, matching local networks and the specific characteristics of each individual. Professionals should make use of technological resources (cellphones, Internet, video games, videos, etc.) to encourage participation, monitor the frequency and intensity of exercises, and teach individuals how to increase their daily regular physical activity levels;
• Perform pre-participation assessments, indicate prior medical evaluation for recommended cases, and perform regular reassessments to verify the effectiveness of physical activity practices and adjust them as they evolve.

### 7.2.4.1. Collective Actions by Physical Education and Physical Therapy Professionals

• Within multidisciplinary teams, developing community-based PA should be encouraged, through patients and representatives from the community and civil society organizations, given that leisure activities improve quality of life for the community.382
• As team members, physical education professionals should use positive results to show that treating and preventing HT depends on a combination of decreasing sedentary behavior and increasing physical activity with other factors, such as a healthy diet, weight loss, less stress, decreased salt and alcohol intake, smoking cessation, etc. In addition, adherence to pharmacological treatment and frequent BP readings should be encouraged in order to help control the disease.
• The strategy of establishing Leagues and Associations of people who suffer from HT helps increase patients' adherence to treatment, and these professionals could join the health care teams working alongside these institutions.
• One-off activities, such as Hypertension Prevention Campaigns and Fight Against Hypertension Campaigns, are important and efficient strategies to help patients learn about their health. Physical education and physical therapy professionals have an important role in this setting.

## 7.3. Multidisciplinary Team Actions

At the primary and secondary care level, in addition to physicians, the multidisciplinary teams can include nurses, nursing technician/assistants, nutritionists, psychologists, social workers, physical education professionals, physical therapists, pharmacists, music therapists, managerial staff, and community health workers, though not all role are required before the team can act. ^383^ ^,^ ^384^

According to the National Primary Care Policy (Ordinance 2.436, dated September 21, 2017), defines, in Article 2, that “primary care is the set of individual, familial and collective actions that involve the promotion, prevention, protection, diagnosis, treatment, rehabilitation, damage reduction, palliative care, and surveillance, based on practices of comprehensive care and qualified management, performed by a multidisciplinary team and directed toward the population of a given territory over which the teams assume responsibility.” ^365^

Role overlaps can be minimized by establishing clear rules and working on group harmony. Keep in mind that the education and attitude changes are slow process, and that standardized, clear, objective and balanced communication is critical to achieve one's goals. ^343^ Team members should work within the boundaries of their specific roles and education, as determined by guidelines and their respective boards. The individual actions of other team members should also be acknowledged. ^347^ ^,^ ^384^

Multidisciplinary teams have been used to treat hypertensive patients in developed countries for several decades. ^341^ ^,^ ^348^ ^,^ ^350^ ^,^ ^385^ Educational and therapeutic actions may involve groups of patients, family members and the community as a whole, and the methods deployed should always take into consideration the specificities of local and regional societies and cultures. Modern approaches may involve social media and distance education techniques. ^338^ ^,^ ^351^ ^,^ ^357^ ^,^ ^362^ ^,^ ^386^ ^,^ ^387^ Examples of the work of patient-centered multidisciplinary teams, with evidence of better BP control, can be found in Chart 7.1 .

In a recent analysis on the future of HT, Dzau & Balatbat ^388^ state that, to this day, care delivery for hypertensive patients is fragmented, service providers are not aligned, and the information is siloed, and that better health care coordination and integration across different care settings and providers is needed. They also claim that, in the future, controlling or preventing HT will depend on the successful convergence of advances in digital, biotechnological, and biomedical sciences, with a special role for multidisciplinary work. ^388^

**Table:** 

Key Takeaways
In primary care for hypertensive patients, physicians are responsible for diagnosis, risk stratification, and pharmacological and nonpharmacological therapeutic management, at least twice a year.
At the population level, the most important guidelines are to keep body weight within the reference range, increase intake of fruits and vegetables, and lower sodium intake.
In multidisciplinary teams, physical education professionals should recommend decreased sedentary behavior and encourage individuals to meet minimum physical activity requirements in order to acquire healthy habits and improve quality of life for the community.
Nursing care should be person-centered, making basic information more accessible and understandable and aiding individual and collective self-care decision-making through nursing visits, home visits, and educational group activities.
Strategies for patient-centered multidisciplinary teams, with evidence of better BP control, should be deployed by multidisciplinary teams themselves.

## 8. Nonpharmacological Treatment

## 8.1. Introduction

High blood pressure (BP), smoking, obesity, unhealthy diets, and insufficient physical activity are established cardiovascular risk factors (CVRFs) and the target of interventions for hypertension (HT) control. In recent years, unconventional therapies have been investigated, involving the adoption of slow breathing, music therapy, and spirituality. In this chapter on nonpharmacological treatment (NPT), we discuss the evidence behind recommendations for smoking, eating habits, sodium, potassium, dairy, chocolate and cocoa products, vitamin D, supplements and substitutes, weight loss, alcohol consumption, physical activity and exercise, slow breathing, stress control, and spirituality and religiosity.

## 8.2. Smoking

Smoking remains one of the most important CVRFs, and in addition to cigarettes, the use of cigars, cigarillos, pipes, hookahs and vapes remains particularly high in certain countries and is associated with increased CV risk ^400^ (LR: I, LE: A). In Brazil, smoking has trended downward in the last 15 years, but the decrease has not been uniformly distributed ^401^ (LR: IIa, LE: B). Smoking has considerable potential to cause damage, such as accelerating atherothrombotic processes and temporarily increasing BP. On average, tobacco use increases BP by 5 to 10 mm Hg, ^402^ but there are no studies showing the beneficial effects of smoking cessation on HT control. Regardless, cessation should be emphasized due to the risk of CV disease and neoplasia. ^403^ Medications for smoking cessation (such as sustained-release bupropion, varenicline, nicotine gum, drops, nasal spray, and patches) are effective in helping smokers quit ^404^ (LR: IIa, LE: B).

## 8.3. Dietary Patterns

Healthy eating patterns are associated with lower BP. The DASH (Dietary Approaches to Stop Hypertension) diet is capable of lowering BP, with the effect attributed to the increase in fruit, vegetable, low-fat dairy and whole-grain intake, as well moderate consumption of nuts and lower fat, candy, sugary beverages, and red meat. The hypotensive effect is due more to the dietary pattern ( Chart 8.1 ) than to its individual components—high sodium, calcium, magnesium and fiber content, with lower levels of cholesterol and total and saturated fat ^405^ ^,^ ^406^ (LR: I, LE: A). The association between the DASH diet with sodium restriction ^406^ has resulted in a decrease in systolic BP (SBP) of 11.5 mm Hg for hypertensive individuals and 7.1 mm Hg for normotensives compared to a high-sodium diet. Meta-analyses of randomized controlled trials confirm the BP reduction effect ^406^ ^,^ ^407^ (LR: I, LE: A). Some studies suggest that adherence to the DASH diet is associated with lower risk of stroke ^408^ ^,^ ^409^ (LR: IIa, LE: B), cardiovascular mortality ^410^ (LR: I, LE: A) and kidney disease ^411^ (LR: I, LE: A).

**Chart 8.1:** – Example of food portions and quantities recommended in DASH diet for daily or weekly intake for individuals consuming 2000 kcal/day

Food group	Daily portions	Portion size/unit
Fruit	4-5	1 medium fruit 1/4 cup of dried fruit 1/2 cup fresh, frozen, or canned fruit 177 mL fruit juice
Vegetables	4-5	1 cup raw leafy green vegetable 1/2 cup of cooked vegetables 177 mL vegetable juice
Diet dairy products	2-3	237 mL milk 1 cup of yogurt 42 g cheese
Grains and derivatives**	7-8	1 slice of bread 1 cup of ready-to-eat cereal* 1/2 cup cooked cereal, rice, or pasta
Lean meat, poultry, and fish	≤ 2	85 g cooked lean meat, skinless poultry, or fish
Nuts, seeds and legumes***	4-5 times a week	1/3 cup or 42 g nuts 1 tablespoon or 14 g seeds 1/2 cup of cooked dried beans

* Portion sizes range from 1/2 cup to 1 1/4 cup. ** Corn, oat, granola, whole rice. *** Cashew nut, Brazil nut, almonds, peanuts, beans, lentils. Adapted from Fuchs, 2001.^*422*^

Like DASH, the Mediterranean diet is rich in fruits, vegetables, and whole grains and low in red meat. It has a high fat content due to the large amounts of olive oil (source of monounsaturated fatty acids) and includes the consumption of fish and nuts, as well moderate intake of red wine ^412^ (LR: IIa, LE: B). The diet decreases the risk of cardiovascular issues ^413^ ^,^ ^414^ (LR: IIa, LE: A), but its effects on blood pressure are modest ^414^ ^-^ ^417^ (LR: IIa, LE: B).

## 8.4. Sodium Intake

Worldwide habitual sodium intake is estimated at 4 g/day ^418^ (LR: IIa, LE: B), while the recommended intake for hypertensive individuals and for the general population is 2 g/day ^419^ (LR: I, LE: A). Not to depend on individual adherence to sodium restriction, which decreases in the long run, governments are now working with the food industry to lower sodium content in their products. Epidemiological data show that sodium intake is directly associated with high BP, and randomized controlled trials have shown the hypotensive effect of sodium restriction. The proof of concept is based on the dose-response curve, showing that even a small decrease in sodium intake can have an effect, stronger in hypertensive individuals, blacks and older adults ^420^ (LR: I, LE: A). Restricting sodium intake to about 1800 mg/day is associated with a 5.4 mm Hg decrease in SBP for hypertensive individuals ^421^ (LR: I, LE: A). Examples of sodium-rich foods include processed meats (ham, bologna, sausages, salami), bacon, dried meat, chicken nuggets; canned food (tomato extract, corn, peas), cheese (yellow cheese: Parmesan, provolone, prato), ready-made seasonings (Arisco^®^, Sazon^®^, soy sauce [shoyu], Worcestershire sauce, ketchup, mustard, mayo, concentrated extracts, meat tenderizers, and instant soup) and industrialized snacks (potato chips, French fries, and other snacks). ^422^ One part of salt restriction diets is to read the nutrition facts labels on all foods and choose those lower on salt (sodium chloride) and other sources of sodium, giving preference to fresh, frozen or “no salt added” canned vegetables, and to use herbs, spices, and saltless mixes to cook and season food. One should cook rice, pasta, and cereals without salt and choose items with low sodium content, deprecating frozen foods, pizza, ready-made mixes, canned soups and creams, and salad dressings. Whenever possible, wash off canned foods, such as tuna, to lower sodium intake. Some forms of salt (pink Himalayan salt and sea salt, among others) have the same sodium chloride content as table salt and rock salt.

## 8.5. Potassium

High-sodium diets are usually low on potassium, associated with higher incidence of HT. Several randomized controlled trials in population clusters have tested replacing sodium chloride table salt with low-sodium, high-potassium salt products, and led to decreases in BP ^423^ ^-^ ^428^ (LR: I, LE: A). The magnitude of the effect on blood pressure varies with dietary sodium intake and the extent of its replacement by alternative sources of food in the population. A prior meta-analysis ^428^ (LR: I, LE: A) confirmed the effect of sodium replacement for younger and older adults in the short and long term, though the hypotensive effect seems to be more pronounced for hypertensive individuals, with a mean difference of -8.87 mm Hg (95% CI: -11.19 to -6.55) in SBP and -4.04 mm Hg (95% CI: -5.70 to -2.39) in diastolic BP (DBP) over the control group ^42^ (LR: I, LE: A). A meta-analysis of sodium restriction interventions found that, in six high-quality studies, ranging from two months to three years, salt substitutes (potassium chloride replacing sodium chloride from 25 to 50%) significant lowered SBP (-5.7 mm Hg; 95% CI -8.5 to -2.8) and DBP (-2.0 mm Hg; 95% CI -3.5 to -0.4) in China ^429^ (LR: I, LE: A). Potassium-rich foods include apricot, avocado, melon, skim milk, leafy greens, fish (flounder and tuna), beans, orange, peas, prune, spinach, tomato, and raisins.

## 8.6. Dairy Products

Dairy consists of a heterogeneous food group, and its impact on health should be assessed in terms of all of its components. Though rich in saturated fatty acids (in whole milk), they may contain potentially beneficial elements, such as whey protein, phospholips from the fat globule membrane, calcium, magnesium, potassium, probiotics, and vitamins K_1_and K_2_ ^430^ ^,^ ^431^ (LR: IIa, LE: B). Cohort studies suggest dairy consumption is inversely associated with CV risk disease ^432^ ^,^ ^433^ (LR: IIa, LE: B). Some randomized controlled trials suggest a modest hypotensive effect, especially for nonfat dairy product ^434^ ^,^ ^435^ (LR: IIa, LE: A) and milk proteins ^436^ (LR: IIa, LE: B). Keep in mind that dietary guidelines recommend the consumption of low-fat dairy products ^437^ ^,^ ^438^ (LR: IIa, LE: B).

## 8.7. Chocolate and Cocoa Products

A meta-analysis of ten randomized controlled trials (n = 297) found a 4.5 mm Hg decrease (95% CI: 3.3 to 5.9) and a 2.5 mm Hg decrease (95% CI: 1.2 to 3.9) in systolic and diastolic blood pressure, respectively, from increase consumption of cocoa products. The studies were very heterogeneous and the interventions, diverse ^439^ (LR: IIa, LE: A). A recent meta-analysis found similar but weaker results ^440^ (LR: IIa, LE: A). Two aspects deserve attention, though the more recent meta-analysis had more studies. One was that heterogeneity persisted across trials with variable amounts of flavonoids. The second is that increased intake of chocolate or cocoa products adds calories to the diet, which must then be set off by some degree of dietary restriction.

## 8.8. Coffee and Caffeinated Products

In addition to caffeine ( Chart 8.2 ), coffee is also rich in bioactive compounds, such as polyphenols, especially chlorogenic acids, magnesium, and potassium, which may favor lower BP. ^441^ Caffeine can cause sharp increases in BP for over three hours, but regular consumption leads to tolerance. ^442^ Long-term coffee intake has not been associated with higher incidence rates of HT. ^443^ On the contrary, meta-analyses of cohort studies show that coffee intake is associated with a mild decrease in hypertension risk ^443^ ^,^ ^444^ (LR: IIb, LE: B). In the absence of robust experimental evidence, it is recommended that coffee intake should not exceed low to moderate amounts (≤ 200 mg of caffeine) (LR: IIa, LE: B).

**Chart 8.2:** – Caffeine content of caffeinated beverages

	Volume (mL)	Caffeine (mg)
Drip coffee	355	235
Instant coffee	237	63
Espresso	30	63
Decaf	237	2
Black tea	237	47
Green tea	237	28
Cammomile tea	237	0

*Adapted from van Dam RM, Hu FB, Willett WC, 2020.* ^*442*^

## 8.9. Vitamin D

Despite some observational studies suggesting vitamin D deficiency being associated with higher blood pressure or higher incidence rates of hypertension ^445^ ^,^ ^446^ (LR: IIb, LE: A), studies on vitamin D supplementation have found inconsistent results ^447^ ^-^ ^449^ (LR: IIb, LE: A). Therefore, the role of vitamin D in blood pressure control is still unclear.

## 8.10. Supplements and Substitutes

In addition to lowering sodium intake from processed foods, other alternatives enable us to minimize the harmful effects from sodium consumption and, at the same time, enjoy the beneficial effects of potassium. In a randomized controlled trial, Chinese participants with prior cardiovascular disease or SBP above 160 mm Hg were selected at random to receive a combination of 65% sodium chloride, 25% potassium chloride, and 10% magnesium sulfate or 100%-sodium chloride table salt. The intervention resulted in a mean decrease of 3.7 mm Hg (1.6 to 5.9) in systolic pressure, with maximum effect in 12 months, a 5.4 mm Hg (2.3 to 8.5) decrease ^426^ (LR: IIa, LE: B). A randomized controlled trial with hypertensive individuals and their families found similar but weaker results after 36 months ^28^ (LR: I, LE: A).

Though calcium supplementation may have a mild effect on preventing hypertension ^450^ (LR: IIa, LE: B), its role in treatment has not yet been established. A meta-analysis of 12 randomized controlled trials found the use of multivitamin and multimineral supplements lowered BP in individuals suffering from chronic diseases. In a subgroup consisting of 58 hypertensive individuals, the analysis found a 7.98 mm Hg (14.95 to 1.02) decrease in SBP, but negligible significance for its impact on DBP ^451^ (LR: IIa, LE: B).

## 8.11. Weight Loss

The hypertensive effect of weight gain is well known. The relationship between BP and obesity rates is practically linear. Excess body fat, especially visceral fat, is a major risk factor for increased BP, which may be responsible for 65 to 75% of cases of HT. ^452^ Weight loss lowers BP even without reaching the desired weight. In a meta-analysis of 25 studies, losing 5.1 kg in weight led to a mean decrease of 4.4 mm Hg in SBP and 3.6 mm Hg in DBP ^453^ (LR: I, LE: A). For overweight and obese individuals, weight loss is always an essential recommendation in HT treatment. Body fat assessments should not limit themselves to body mass index (BMI), but rather include central adiposity parameters, such as waist circumference (WC). Ideally, individuals should attain and maintain a healthy body weight, defined as BMI (kg/m ^2^ ) < 25 in adults (LR: I, LE: A) and, according to the Brazilian Ministry of Health, from 22 to < 27 in older adults, as well as WC (cm) < 90 for men and < 80 for women. Evidence from a meta-analysis including participants from four continents shows that, for every 5-unit increases in BMI above > 25, the risk of early death increases approximately 31%, as does the 49% risk of cardiovascular mortality ^454^ (LR: IIa, LE: B).

## 8.12. Alcohol Consumption

There is a linear relationship between alcohol consumption and BP, and alcohol abuse is linked to higher rates of HT. A recent meta-analysis, including 36 randomized controlled trials and 2865 participants, found that, for up to two drinks a day, lowering alcohol intake was not associated with significant decreases in BP. However, for individuals who took more than two drinks a day, lowering alcohol intake was associated with a greater decrease in BP, approximately 5.5 mm Hg (6.70 to 4.30) in SBP and 3.97 (4.70 to 3.25) in DBP. The decrease was more pronounced for those who drank six or more drinks a day and lowered their intake by approximately 50% ^15^ (LR: IIa, LE: B). Among drinkers, intake should not exceed 30 g of alcohol/day, ie, 1 bottle of beer (5% alcohol, 600 mL), two glasses of wine (12% alcohol, 250 mL), or one 1 dose (42% alcohol, 60 mL) of distilled beverages (whiskey, vodka, spirits). That threshold should be cut in half for low-weight men, women, the overweight, and/or those with high triglycerides. Teetotalers should not be encouraged to drink alcohol. ^15^

## 8.13. Physical Activity and Physical Exercise

Physical activity (PA) refers to any body motion that increases energy expenditure above consumption at rest, such as walking, working, housework, and leisure activities. Physical exercise (PE), in turn, refers to structured, organized, and purposeful PA, with goals like improving health and/or fitness. ^455^ Sedentary behavior is spent in low energy expenditure activities (≤ 1.5 MET), such as those performed sitting, reclining, or lying down (watching TV, sitting at the computer, playing video games, or working). ^456^ Decreasing sedentary time, even for small periods, lowers the mortality risk ^457^ (LR: IIb, LE: B).

Regular physical exercise lowers the incidence of HT. ^458^ In addition, hypertensives who follow PA health recommendations show a 27 to 50% decrease in mortality risk, but lower levels of PA also produce benefits ^53^ (LR: I, LE: A). In HT treatment, additional benefits may be obtained from structure PE, with aerobic training supplemented by resistance training. Aerobic training has a proven effect in lowering office and ambulatory BP, while dynamic resistance training and isometric handgrip resistance training lower office BP, but there is no evidence that it lowers ambulatory BP. ^459^ Chart 8.3 shows the magnitude of the effect of that training (LR: I, LE: A). ^459^ ^-^ ^461^

**Chart 8.3:** – Magnitude of blood pressure reduction in hypertensive individuals with physical training

Training	Systolic/diastolic blood pressure
Aerobic ^459^	-12.3/-6.1 mm Hg*
Aerobic ^459^	-8.8/-4.9 mm Hg**
Dynamic resistance training ^460^	-5.7/-5.2 mm Hg
Isometric handgrip resistance training ^461^	-6.5/-5.5 mm Hg

* Office blood pressure. ** Ambulatory blood pressure monitoring.

Other forms of training, such as aquatic exercise, ^462^ yoga, ^463^ tai chi, ^464^ and high-intensity interval training , ^465^ among others, also seem to lower office BP for hypertensive patients. However, there is no documented evidence on their effects on ambulatory blood pressure nor on their potential risks, so they are still not recommended. Chart 8.4 lists physical activity and physical exercise recommendations.

**Chart 8.4:** – Physical activity and physical exercise recommendations.

Recommendations
*Decrease sedentary behavior – LR: IIb, LE: B*
**Stand for 5 minutes for every 30 minutes sitting down.**
Recommended physical activity at population level – LR: I, LE: A
**Perform at least 150 minutes of moderate physical activity per week**
Physical training – aerobics supplemented with resistance training – LR: I, LE: A
*Prescription of aerobic training – mandatory*
**Various modalities: walking, running, dancing and swimming, among others.**
**Frequency: 3 to 5 times per week (more often is better)**
**Duration: 30 to 60 minutes per session (longer is better)**
**Moderate intensity defined by:**
**1) Highest intensity still able to have a conversation (without panting)**
**2) Feeling “slightly tired” to “tired” (11 to 13 in the Borg scale)**
**3) Keep heart rate (HR) during training in the following range: HRtraining = (HRmax – HRrest) x % + HRrest Where:** **HRmax:** **obtained either on a maximum exercise test, using the regular medications, or by calculating maximum HR estimated for age (220 - age). The formula may not be used with hypertensive patients suffering from heart disease, taking beta-blockers or taking dihydropyridine calcium channel blockers.** **HRrest:** **measured after 5-minute rest lying down.** **%** **: use 40% as lower threshold and 60% as upper threshold.**
*Prescription of endurance training – complementary*
**2 to 3 times a week**
**8 to 10 exercises for the major muscle groups, prioritizing unilateral execution, when possible**
**1 to 3 sets**
**10 to 15 repetitions up to moderate fatigue (repetition with decreased motion speed) – approximately 60% of 1RM**
**Long passive pauses – 90 to 120 s**

Light to moderate PA and PE may be prescribed to for individuals without heart, cerebrovascular, or renal disease without prior medical assessment. If symptoms appear during PA or PE, it should be interrupted and the individual should seek medical help. Hypertensive individuals with comorbidities, who are symptomatic or who intend to participate in high-intensity or competitive activities should undergo a prior medical evaluation. ^466^ The exercise stress test is recommended to evaluate physical fitness and to prescribe physical exercise, ^467^ enabling an assessment of BP response to physical effort and check for coronary disease in symptomatic individuals or those with multiple risk factors. Training sessions should be canceled if BP is above 160/105 mm Hg; measuring BP during aerobic training is recommended for hypertensive individuals who exhibit hyper-reactivity, and the intensity of the physical activity should be lowered if BP is above 180/105 mm Hg (LR: IIa, LE: C).

## 8.14. Slow Breathing

Slow or guided breathing requires respiratory rate reduction to 6-10 breaths/minute for 15-20 minutes/day to promote casual BP reduction. Randomized controlled trials on device guided breathing (Resperate device^®^), analyzed in a previous meta-analysis, found no significant decrease in BP after excluding five studies involving industry participation. ^468^ A recent meta-analysis, combining six voluntary slow breathing exercise trials compared to natural breathing, found a 6.36 mm Hg decrease in SBP (95% CI: 10.32 to 2.39) and a 6.39 mm Hg decrease in DBP (95% CI: 7.30 to 5.49) in DBP compared to control group participants in randomized controlled trials lasting up to six months. ^469^ Existing evidence shows that, in the short run, voluntary slow breathing exercises can lower SBP and DBP in HT patients with CV disease (LR: IIa, LE: A). In a clinical trial with a small number of participants, slow breathing was shown to lower blood pressure at rest for individuals with isolated HT, in addition to responses to static and dynamic exercises ^470^ (LR: IIb, LE: B).

The association between listening to music and deep breathing, in comparison with listening to music only, did not result in statistically significant BP reductions. Participants from both treatment groups achieved clinically significant BP reductions ^471^ (LR: IIb, LE: B).

## 8.15. Stress Control

Overall, no robust evidence on the efficacy of techniques used in stress management has been found, including behavioral therapies, transcendental meditation (LR: IIb, LE: B), other meditation techniques (LR: III, LE: C), yoga (LR: III, LE: C), relaxation therapies (LR: III, LE: C), and biofeedback approaches (LR: IIb, LE: B). There is more evidence for guided slow breathing than there is available for acupuncture (LR: III, LE B). Clinical indications show only a trend towards lowering BP, whether used separately or in combination ^472^ (LR: IIa, LE: B). In two meta-analyses, music therapy was associated with a significant reduction in SBP, ^473^ ^,^ ^474^ while in a third only its tendency to lower BP was observed ^475^ (LR: IIb; LE: A).

Meditation can be seen as the experience of emptying one's mind and making it devoid of thought; the practice of focusing one's concentration on a single object until becoming aware of that object; contemplating a single aspect of reality; or developing a given mental or even behavioral quality. ^476^ ^,^ ^477^ A systematic review showed that transcendental meditation led to a 4 mm Hg decrease in SBP and a 2 mm Hg decrease in DBP ^478^ (LR: IIb, LE: B). However, the mechanisms through which meditation lowers BP are not fully understood, and others have criticized these studies for methodological limitations. ^479^

## 8.16. Religiosity and Spirituality

Spirituality is associated with physical, psychological and social aspects, enabling a more holistic view of human beings and placing them at the center of attention and of treatment ^480^ (LR: IIb, LE: C). It may be considered a set of moral, mental and emotional values that guide thoughts, behaviors, and attitudes ^3^ ^,^ ^481^ (LR: I, LE: B). Religion, in turn, is understood as an organized system of beliefs, practices, and symbols with the purpose of bringing its adherents closer to the transcendental or the divine ^3^ ^,^ ^481^ (LR: I, LE: B).

Studies suggest there is an association between religiosity and spirituality (R/S) and all-cause mortality, cancer, and CV mortality, as well as quality of life ^482^ ^-^ ^485^ (LR: I, LE: B). The relevant mechanisms involve favorable changes in lifestyle and CVRFs, such as lower levels of serum glucose, cholesterol, fibrinogen, cortisol, and inflammatory cytokines ^481^ ^,^ ^486^ (LR: I, LE: B).

Given the multidimensional aspects of R/S and the characteristics of study populations, observational studies that assess the association between BP and/or HT risk have found heterogeneous results, but most suggest beneficial effects. ^77^ ^,^ ^481^ ^,^ ^487^ ^,^ ^488^ In the SWAN (Study of Women’s Health Across the Nation) study, with over 1600 middle-aged women as participants, daily spiritual practices were not found to be protective for SBP or HT. ^489^ In the Chicago Community Adult Health Study, frequency of religious attendance was not associated with HT, while the habit of prayer had a positive association. Spirituality was connected to diastolic HT, while the meaning of forgiveness was associated with lower DBP and lower probability of HT. ^490^ In a different study, more frequent religious attendance was associated with lower DBP, but not lower SBP ^491^ (LR: IIa, LE: B).

A recent review found that elements of R/S may interfere positively in adherence to pharmacological treatment, but other studies have found opposite or mixed effects, especially for severe and chronic diseases ^492^ (LR: IIa, LE: C). Health care professionals should learn to identify patient demands and expectations, provide adequate support, and overcome conflicts. Open-ended questions or semistructured surveys can be useful to that end ^3^ ^,^ ^493^ (LR: I, LE: B). Despite the fact that evidence from observational studies correlate R/S and HT, few clinical trials have assessed the effects of interventions in this area, especially for severe CV diseases, chronic diseases or palliative care ^481^ ^,^ ^494^ (LR: IIb, LE: B).

**Table:** 

Key Takeaways
Hypertensive individuals should be assessed in terms of smoking habits, and smoking cessation should be pursued, with the help of medications if needed, since it increases CV risk.
Diets like DASH, which increase fruit, vegetable, low-fat dairy, and whole-grain intake as well fostering moderate consumption of nuts and lower fat, candy, sugary beverages, and red meat consumption, should be prescribed
Daily sodium intake should be restricted to 2 g/day, with sodium chloride replaced by potassium chloride where there are no restrictions.
Body weight should be controlled to maintain BMI < 25 kg/m ^2^ .
Individuals should perform at least 150 minutes of moderate physical activity per week Decreasing sedentary behavior should be encouraged, with individuals standing for 5 minutes for every 30 minutes spent sitting down.

## 9. Pharmacological Treatment

## 9.1. Treatment Objectives

Cardiovascular (CV) protection is the primary objective of antihypertensive treatment. Lower blood pressure (BP) is the first goal, while the greater objective is to reduce the CV outcomes and mortality linked to hypertension (HT). ^5^ ^,^ ^37^ ^,^ ^164^ ^,^ ^495^ Meta-analyses of randomized clinical trials studying hypertensive patients show that decreasing systolic BP by 10 mm Hg and diastolic BP by 5 mm Hg with medications is accompanied by a significant decrease in relative risk for major outcomes: 37% for risk of stroke, 22% for coronary artery disease (CAD), 46% for heart failure (HF), 20% for CV mortality, and 12% for all-cause mortality. ^83^ ^,^ ^85^ ^,^ ^307^ ^,^ ^308^ ^,^ ^496^ ^,^ ^497^ The higher the CV risk, the greater the benefits, but there are benefits even for patients with small BP elevations and low to moderate CV risk. ^307^ ^,^ ^308^ ^,^ ^496^

It should be stressed that these findings come mostly from clinical trials involving high CV risk hypertensives age 50 and older, and follow-up is rarely longer than five years. Therefore, the befits for young individuals, for low- to moderate-risk individuals, and from longer treatment periods are extrapolated from the scientific evidence available. ^498^ In particular, for this patient group, we infer that assessing the impact of antihypertensive medications in protecting end-organs may be a useful indirect indicator for treatment effectiveness, especially reduction of left ventricular mass ^499^ ^,^ ^500^ and albuminuria. ^501^ Thus, adequate treatment for individuals below the age of 50 is strongly recommended.

## 9.2. General Principles of Pharmacological Treatment

Most hypertensive patients require medications in addition to lifestyle changes in order to achieve their blood-pressure targets. ^5^ ^,^ ^37^ ^,^ ^83^ ^,^ ^164^ ^,^ ^307^ ^,^ ^307^ ^,^ ^308^ ^,^ ^495^ ^,^ ^497^ ^,^ ^502^ Chart 9.1 shows the recommended treatment onset for lifestyle interventions and pharmacological treatment according to blood pressure, age, and cardiovascular risk.

**Chart 9.1:** – Onset of treatment with lifestyle interventions and pharmacological treatment according to blood pressure, age, and cardiovascular risk

Status	Scope	Recommendation	Class	Level of evidence
Onset of lifestyle interventions	All stages of hypertension and blood pressure 130-139/85-89mm Hg	To diagnosis	I	A
Onset of pharmacological treatment	Stage 2 and 3 hypertensive patients	To diagnosis	I	A
	Stage 1 hypertensives at moderate to high cardiovascular risk	To diagnosis	I	B
	Stage 1 hypertensives and low cardiovascular risk Individuals with BP 130-139/85-89 mm Hg and preexisting CVD or at high cardiovascular risk	Wait 3 months for effects of lifestyle interventions	IIa	B
	Frail older adults and/or oldest old hypertensives	SBP ≥ 160 mm Hg	I	B
	Healthy older hypertensive patients	SBP ≥ 140mm Hg	I	A
	Individuals with BP 130-139/85-89 mm Hg without preexisting CVD and at low to moderate cardiovascular risk	Not recommended	III	

The five main classes of antihypertensive medication—diuretics (DIUs), calcium channel blockers (CCBs), angiotensin-converting enzyme inhibitors (ACEIs), angiotensin II receptor blockers (ARBs), and beta-blockers (BBs)—have shown to significantly lower BP compared to placebos, as well as produce significant decreases in fatal and nonfatal CV outcomes, and this benefit is fundamentally linked to the BP decrease. ^5^ ^,^ ^37^ ^,^ ^83^ ^,^ ^164^ ^,^ ^307^ ^,^ ^308^ ^,^ ^495^ ^,^ ^497^ BBs are useful in specific clinical conditions: post-acute myocardial infarction (AMI), chest angina, HF with reduced ejection fraction (HFrEF), heart rate (HR) control, and women of childbearing age. ^5^ ^,^ ^37^ ^,^ ^164^ ^,^ ^495^ Other classes, such as alpha-blockers, centrally acting sympatholytics, aldosterone antagonists and direct vasodilators, have not been as widely studied in clinical trials, are associated with higher rates of adverse events, and should only be used when BP control has not been achieved with combinations based on the primary medication classes mentioned above. ^37^ ^,^ ^164^ ^,^ ^495^ ^,^ ^503^ ^,^ ^504^

The desirable features of antihypertensive medications are that they:

• Have shown the ability to reduce CV morbidity and mortality;
• Be effective orally;
• Be well tolerated;
• Preferably be administered in a single daily dose;
• Able of being used in association;
• Have quality control in its production.
Additional recommendations are:
• Use for at least four weeks, before any change, except for special situations;
• Do not use compound medications, which are not subject to pharmacokinetic control and pharmacovigilance;
• Patients are instructed about the importance of continuous use of antihypertensive medication, the occasional need for dose adjustments and switching or combination of drugs, and the occasional onset of adverse effects;
• There is no sufficient evidence for recommending routine nocturnal administration of antihypertensive medications, except under special conditions.

## 9.3. Therapy Regimens

Pharmacological treatment may start as monotherapy or as drug combinations. It should be emphasized that the use of drug combinations is the preferred strategy for most hypertensive patients ( Figure 9.1 ).

**Figure 9.1:** – Flow chart for pharmacological treatment

### 9.3.1. Monotherapy

Monotherapy can be the initial antihypertensive strategy for stage 1 HT patients at low CV risk ^37^ ^,^ ^164^ ^,^ ^495^ , with BP 130-139/85-89 mm Hg at high CV risk ^307^ or older adults and/or frail individuals ^4^ ( Figure 9.1 ). For these patient profiles, the desired BP decrease is small and should be achieved gradually in order to prevent adverse events. ^37^ ^,^ ^164^ ^,^ ^495^ ^,^ ^502^

Treatment should be individualized, and the initial choice of medication should be based on the general desirable characteristics of the antihypertensive medications described previously, on individuals particularities, on the presence of associated diseases and end-organ damage (EOD), and on socioeconomic conditions. ^5^ ^,^ ^37^ ^,^ ^164^ ^,^ ^495^

The preferred classes of antihypertensive medication ^5^ ^,^ ^37^ ^,^ ^164^ ^,^ ^495^ for BP control in the initial monotherapy are as follows:

• Thiazide and thiazide-like diuretics; ^83^ ^,^ ^307^ ^,^ ^497^
• CCBs; ^83^ ^,^ ^307^ ^,^ ^497^
• ACEIs; ^83^ ^,^ ^307^ ^,^ ^497^
• ARBs. ^83^ ^,^ ^307^ ^,^ ^497^

BBs may be considered as the initial drug in certain situations, ^5^ ^,^ ^37^ ^,^ ^83^ ^,^ ^164^ ^,^ ^307^ ^,^ ^495^ ^,^ ^497^ as described above, and are more frequently used in combination with other medications. Dosages may be adjusted in order to achieve recommended blood-pressure targets.

### 9.3.2. Drug Combinations

Drug combination are the preferred therapeutic strategy for most hypertensive patients, regardless of HT staging and associated CV risk ^5^ ^,^ ^37^ ^,^ ^164^ ^,^ ^495^ ^,^ ^502^ ^-^ ^507^ ( Figure 9.1 ). Treatment should begin with a combination of two medications with different mechanisms of actions, except for the association of thiazide DIUs and potassium-sparing DIUs. If the blood-pressure target is not reached, adjusting doses and/or adding a third drug are indicated. Next, more medications are added until the BP control is achieved. ^502^ ^-^ ^504^

The rationale for drug combinations is based on the incremental antihypertensive effect when working on different physiopathological mechanisms by synergistic actions and by inhibiting the activation of counter-regulatory mechanisms. ^502^ ^,^ ^503^ In addition, drug combinations have the potential of decreasing the rate of side effects due to the lower dose of each combined medication or the ability of one drug to antagonize the adverse effects of another. ^502^ ^,^ ^503^ Higher adherence to treatment and decreased therapeutic inertia are important benefits. Fixed-dose and single-pill combinations are preferable, as they are associated with higher adherence to treatment and, consequently, to better clinical results. ^502^ ^,^ ^503^

The onset of fixed-dose drug combination treatment is associate with decreased risk of CV outcomes compared to the traditional onset of treatment with monotherapy, faster achievement of blood-pressure targets, protection of end-organs, and long-term CV outcomes. ^502^ ^-^ ^507^

## 9.4. General Characteristics of Different Classes of Antihypertensive Medications

Chart 9.2 lists the antihypertensive medications available in Brazil, divided by therapeutic class.

**Chart 9.2:** – List of antihypertensive medications available in Brazil

Class	Class and Medication	Usual daily dose (mg)	Freq.*	Comments and recommendations
**Thiazide and thiazide-like diuretics**	Hydrochlorothiazide	25-50	1	Higher doses of thiazides and thiazide-like medications increase the diuretic effect without increasing antihypertensive action.
	Chlorthalidone	12.5-25	1	
	Indapamide	1.5	1	
**Loop diuretics**	Furosemide	20-240	1-3	Used in chronic renal failure (CRF), congestive heart failure (CHF), and fluid retention conditions (edema).
	Bumetanide	1-4	1-3	
**Potassium-sparing diuretics**	Spironolactone	25-100	1-2	May cause hyperpotassemia, particularly in CRF and when associated with ARBs or ACE inhibitors.
	Amiloride	2.5-5	1	Available only in combination with hydrochlorothiazide or chlorthalidone
**Dihydropyridine calcium channel blockers (CCBs)**	Amlodipine	2.5-10	1	Avoid use in patients with heart failure and reduced ejection fraction. May cause lower limb edema depending on dose.
	Felodipine	2.5-10	1	
	Nifedipine	10-60	1-3	
	Nitrendipine	10-30	1	
	Manidipine	10-30	1	
	Lacidipine	2-6	1	
	Lercanidipine	10-20	1	
	Levamlodipine	2.5-5	1	
**Nondihydropyridine calcium channel blockers (CCBs)**	Verapamil	120-360	1-2	Avoid use in patients with heart failure and reduced ejection fraction. Avoid association with beta-blockers and in patients with bradycardia.
	Diltiazem	80-240	1-2	
**Angiotensin-converting enzyme inhibitors (ACEIs)**	Captopril	25-150	2-3	Avoid use in women of childbearing age due to the high risk of fetal malformations and other gestational complications. Contraindicated in combination with other renin-angiotensin-aldosterone system inhibitors, except spironolactone for CHF. Risk of hyperpotassemia for patients suffering from renal failure or receiving potassium supplementation.
	Enalapril	5-40	1-2	
	Benazepril	10-40	1-2	
	Lisinopril	10-40	1	
	Fosinopril	10-40	1	
	Ramipril	2.5-20	1-2	
	Perindopril	2,5-10	1	
**Angiotensin II AT**_**1**_ **receptor blockers (ARBs)**	Losartan	50-100	1-2	Same recommendations as ACEIs.
	Valsartan	80-320	1	
	Irbesartan	150-300	1	
	Candesartan	8-32	1	
	Olmesartan	20-40	1	
	Telmisartan	20-80	1	
**Noncardioselective beta-blockers (BBs)**	Propranolol	80-320	2-3	Avoid sudden withdraw of BBs, as it may cause reflex tachycardia and discomfort.
	Nadolol	40-160	1	
	Pindolol	10-60	1	Has intrinsic sympathomimetic activity, leading to less bradycardia.
**Cardioselective beta-blockers**	Atenolol	50-100	1-2	
	Metoprolol	50-200	1	
	Bisoprolol	5-20	1	
	Nebivolol	2.5-10	1	Vasodilatory action via nitric oxide.
	Carvedilol	12.5-50	1-2	Alpha-blocker effect produces less bradycardia.
**Centrally acting sympatholytics**	Methyldopa	500-2.000	2	
	Clonidine	0.2-0.9	2	Abrupt clonidine withdraw may cause rebound hypertension (hypertensive crisis) via catecholamine release at synaptic endings.
	Rilmenidine	1-2	1-2	
**Alpha-blockers**	Prazosine	1-20	2-3	Initiate with low dose before lying down since it may trigger orthostatic hypotension. Progressively increase every 2 days. Other alpha-blockers are exclusively available for benign prostate hyperplasia (tamsulosin, alfuzosin, silodosin).
	Doxazosin	1-16	1	
**Direct-acting vasodilators**	Hydralazine	50-200	2-3	May cause sodium and fluid retention, hypervolemia, and reflex tachycardia. Must be used in combination with loop diuretic. Lupus-like syndrome at high dose.
**Direct renin inhibitors**	Aliskiren	150-300	1	Same recommendations as ACEIs and ARBs.

### 9.4.1. Diuretics (DIUs)

The mechanisms of antihypertensive action of DIUs are initially related to their natriuretic effects, with a decrease in the circulating volume and extracellular volume. After 4-6 weeks, circulating volume normalizes and a reduction in peripheral vascular resistance (PVR) occurs. Diuretics lower BP and CV morbidity and mortality. ^508^ ^-^ ^510^ Their antihypertensive effect is not directly related to their doses, but side effects are associated with dose and potency of diuretic action. Thiazide (hydrochlorothiazide) or thiazide-like (chlorthalidone and indapamide) DIUs at low doses should be preferred, because they are milder and longer-acting. Loop DIUs (furosemide and bumetanide) should be reserved for clinical conditions featuring sodium and fluid retention, such as renal failure (creatinine > 2.0 mg/dL or estimated glomerular filtration rate ≤ 30 mL/min/1.73m ^2^ ) and edema (HF or nephritic syndrome). Potassium-sparing DIUs (spironolactone and amiloride) are usually associated with a thiazide or loop DIU. Spironolactone is habitually used as the fourth medication in drug combinations for patients with resistant and refractory HT. This aspect is discussed in further detail in the chapter 16 to those more severe forms of HT.

Chlorthalidone has higher diuretic potency than hydrochlorothiazide, compared at proper doses, and its longer half-life made it the preferential DIU for resistant or refractory HT, since sodium and fluid retention is an important mechanism in resistance to treatment. ^504^ The indication of chlorthalidone as the preferred DIU because it promotes a higher decrease in CV events is controversial, as a meta-analysis and observational studies with large numbers of participants were not in agreement. ^495^ ^,^ ^511^ ^,^ ^512^ On the other hand, as expected from the more intense diuretic effect, these studies have found greater rates of adverse effects for chlorthalidone, particularly hydroelectrolytic and metabolic disorders. Indapamide, a thiazide-like, which has been growing in use in recent years, like chlorthalidone, has greater potency and longer-acting diuretic effect; like previous medications, it has proven antihypertensive effect, decreases CV events and has a positive metabolic profile. ^513^ Thus, there is no definitive data behind the preference for chlorthalidone in antihypertensive treatment for individuals with normal renal function, but it may be used when a larger diuretic effect is desirable, especially in resistant HT, as it is more potent than hydrochlorothiazide.

#### 9.4.1.1. Adverse Effects of Diuretics

Major adverse effects of diuretics are weakness, cramps, hypovolemia, and erectile dysfunction. Hypopotassemia is the most common metabolic effect, often accompanied by hypomagnesemia, which can induce ventricular arrhythmias, mainly extrasystole. Hypopotassemia also leads to insulin secretion, increasing glucose intolerance, and the risk of developing type 2 diabetes mellitus. Uric acid increase is an almost universal effect of DIUs, and may trigger gout crises in predisposed individuals.

The use of low doses of DIUs decreases the risk of adverse effects without hindering their antihypertensive efficacy, especially when associated with other drug classes. Spironolactone may cause gynecomastia and hyperpotassemia, and the latter is the most frequent electrolyte disorder in patients with impaired renal function. There are reports that indapamide may have a better metabolic profile than hydrochlorothiazide. ^513^

## 9.4.2. Calcium Channel Blockers (CCBs)

This medication class blocks the calcium channels in smooth muscle cell membranes in the arterioles, lowers calcium availability inside cells to impair muscle contraction, and consequently decreases peripheral vascular resistance by vasodilation. ^514^ ^,^ ^515^

CCBs can be divided into two basic forms: dihydropyridines and nondihydropyridines. Dihydropyridines (amlodipine, nifedipine, felodipine, manidipine, levamlodipine, lercanidipine, lacidipine) are predominant vasodilators, with minimum interference in HR and systolic function, and therefore are more often used as antihypertensive agents.

Nondihydropyridines, such as diphenylalkylamines (verapamil) and benzothiazepines (diltiazem), have a lower vasodilating effect, and act on the heart musculature and the cardiac conduction system. Thus, they lower HR, have an antiarrhythmic effect and can depress systolic function, primarily in patients already suffering from myocardial dysfunction, and should be avoided in individuals with that condition.

Long-acting CCBs should be preferred to prevent unwanted oscillations in HR and BP. They are effective antihypertensive medications that reduce CV morbidity and mortality. ^307^ ^,^ ^515^ ^-^ ^517^ An outcome study has reaffirmed the efficacy, tolerability and safety of this drug class HT treatment in CAD patients, ^518^ making it an alternative to BBs when the latter cannot be used, or in combination for cases of refractory angina.

### 9.4.2.1. Adverse Effects of Calcium Channel Blockers

Ankle swelling is usually the most common side effect, resulting from the vasodilating action (more arterial than venous), which causes capillary transudation. Throbbing headaches and dizziness are common. Facial blushing is more common with fast-acting dihydropyridine CCBs. Hyperchromia of the distal third of the legs (ochre dermatitis) and gingival hypertrophy are two occasional adverse effects.

Adverse effects are usually dose-dependent, may cause intolerance to dihydropyridine CCBs and may result in resistance to treatment. In these cases, lipophilic CCBs (manidipine, lercanidipine, lacidipine) may be tested, or levamlodipine at low doses. Verapamil and diltiazem can worsen HF, as well as bradycardia and atrioventricular block. Verapamil has been found to cause constipation. ^516^

## 9.4.3. Angiotensin-Converting Enzyme Inhibitors (ACEIs)

Angiotensin-converting enzyme inhibitors are effective antihypertensive drugs whose primary action is inhibiting angiotensin I converting enzyme, responsible for both transforming angiotensin I into angiotensin II (vasoconstrictor) and lowering bradykinin degradation (vasodilator). They are effective for HT treatment, lowering CV morbidity and mortality. ^307^ They have been shown to be useful in many other CV conditions, such as HFrEF and post-AMI anti-remodeling, and may have antiatherosclerotic properties. They also delay renal function decline in patients with diabetic nephropathy or kidney diseases of other etiologies, especially in case of albuminuria. ^519^

### 9.4.3.1. Adverse Effects of Angiotensin-Converting Enzyme Inhibitors

Usually well-tolerated by most hypertensive patients, its major side effect is dry coughs, affecting 5 to 20% of patients. Angioneurotic edema and skin rashes are rare. ^520^ When administered to renal failure patients, it can initially worsen renal function, usually discretely, due to adjustments in intraglomerular hemodynamics (vasodilation of efferent arterioles and lower glomerular filtration pressure) resulting in higher plasma creatinine and urea rates. ^521^ However, the initial loss of renal function is a protective mechanism, since it prevents glomerular hyperfiltration and slows down the progression of chronic kidney disease. ^522^ In case of major loss of renal function (> 30%), the medication should be withdrawn and the possibility of bilateral renal artery stenosis or renal artery stenosis in solitary functioning kidney.

ACEIs and other renin-angiotensin-aldosterone system (RAAS) blockers may cause hyperpotassemia in patients with renal failure, especially diabetic patients, and are contraindicated during pregnancy due to the risk of fetal complicactions. ^523^ ^,^ ^524^ Therefore, they should be carefully monitored when administered to adolescents and women of childbearing age.

## 9.4.4. Angiotensin II AT1 Receptor Blockers (ARBs)

ARBs antagonize angiotensin II action via the specific blockade of AT1 receptors, responsible for the primary effects of angiotensin II (vasoconstriction, cell proliferation and aldosterone release). In HT treatment, especially in populations at high CV risk or with comorbidities, they decrease CV and renal (diabetic nephropathy) morbidity and mortality ^525^ ^-^ ^531^

### 9.4.4.1. Adverse Effects of Angiotensin II AT1 Receptor Blockers

Adverse effects related to ARBs are not common, with exanthema observed in rare occasions. Like ACEIs, ARBs may promote an initial decrease in glomerular filtration via vasodilation of efferent arterioles, lowering glomerular filtration pressure, but the effect is nephroprotective in the long run. ^529^ ^-^ ^531^ Similarly to ACEIs, ARBs may cause hypercalcemia, especially in the presence of renal failure, and are contraindicated during pregnancy, and the same care should be taken for women of childbearing age.

## 9.4.5. Beta-Blockers (BBs)

Beta-blockers have complex pharmacological actions. They promote an initial decrease in cardiac output and renin secretion, with readaptation of baroreceptors and decrease in catecholamines in nervous synapses. ^532^ ^,^ ^533^

They can be divided into three categories, according to selectivity in adrenergic receptor binding: 1) nonselective: block both beta-1 adrenergic receptors, found mainly in the myocardium, and beta-2 receptors, found in smooth muscle, the lungs, blood vessels and other organs (propranolol, nadolol and pindolol, the latter displaying intrinsic sympathomimetic activity, acting as a partial adrenergic agonist and producing less bradycardia); 2) cardioselective: preferentially block beta-1 adrenergic receptors (atenolol, metoprolol, bisoprolol and nebivolol, which is the most cardioselective); and 3) vasodilator: manifests as peripheral alpha-1 adrenergic receptor antagonism (carvedilol) and nitric oxide production (nebivolol). ^532^ ^-^ ^535^ Propranolol is useful to patients with essential tremor, mitral valve prolapse, hyperkinetic syndromes (hyperthyroidism and panic disorder), vascular headache, and portal hypertension. ^532^ ^,^ ^533^

A meta-analysis ^536^ including over 130 thousand primary hypertension patients compared BBs to other classes of antihypertensive medications, placebos, and no treatment. It found that compared to other antihypertensive medications (DIUs, CCBs, ACEIs, ARBs), beta-blockers increase the risk of stroke by 16%. Compared to placebo or untreated patients, beta-blockers the lower risk of stroke, but only half as much as would be expected from observed blood pressure decreases. The meta-analysis ^534^ also found that compared to other antihypertensive medications, atenolol increases the risk of stroke by 26% and overall mortality by 8%, both significant results. This is the main reason this guideline recommends BBs as an initial antihypertensive medication only in cases for which there is a specific indication.

### 9.4.5.1. Adverse Effects of Beta-Blockers

The adverse effects are bronchospasm, bradycardia, atrioventricular conduction disorders, peripheral vasoconstriction, insomnia, nightmares, depression, asthenia, and sexual dysfunction. BBs are contraindicated for patients with asthma, chronic obstructive pulmonary disease (COPD), and second- and third-degree atrioventricular block. They may lead to glucose intolerance, induce onset of diabetes mellitus, hypertriglyceridemia, high LDL-cholesterol, and low HDL-cholesterol. The impact on glucose metabolism is potentiated when combined with DIUs. Third-generation BBs (carvedilol and nebivolol) have neutral impact or may even improve the glucose and lipid metabolism, possibly because of their vasodilatory effect, with decrease in insulin resistance and improvement of glucose uptake by peripheral tissues. ^532^ ^,^ ^535^ Studies on nebivolol have also found less sexual dysfunction, possibly due to its effect on endothelial nitric oxide synthesis. ^532^ ^,^ ^535^

## 9.4.6. Centrally Acting Sympatholytics

Centrally acting alpha-agonists stimulate the alpha-2 receptors involved in sympatho-inhibitory mechanisms. ^537^ Their well-defined effects are as follows: a decrease in sympathetic activity and baroreceptor reflex, contributing to relative bradycardia and orthostatic hypotension; mild decrease in PVR and cardiac output; lower plasma renin levels; and fluid retention. Representatives of that group include methyldopa, clonidine, and the imidazoline receptor inhibitor rilmenidine. ^538^ ^,^ ^539^ Clonidine also acts on presynaptic alpha-2 receptors, which prevent norepinephrine release. It accumulates in nerve endings and, when withdrawn suddenly, the uncontrolled release may cause an adrenergic crisis. ^537^ Despite some central alpha-2 agonism, rilmenidine has greater affinity for subtype I imidazoline receptor binding sites, causing fewer undesirable effects than clonidine. ^538^

This class of medication has no unwanted metabolic effects and do not interfere with peripheral resistance to insulin or to the lipid profile. Methyldopa is primarily indicated for HT during pregnancy, as it is used for short periods, there is a large body of experience with its use during this period, and it has a better safety profile for pregnant women and for fetuses. ^537^ ^,^ ^539^ Clonidine can be useful in HT associated with restless legs syndrome, ^540^ opioid withdrawal, ^541^ menopausal hot flashes, ^542^ diarrhea associated with diabetic neuropathy, ^543^ and sympathetic hyperactivity in patients with alcoholic cirrhosis. ^544^

### 9.4.6.1. Adverse Effects of Centrally Acting Sympatholytics

Methyldopa can cause autoimmune reactions, such as fever, hemolytic anemia, galactorrhea, and liver dysfunction, which, in most cases, disappear with cessation of use. If an adverse reaction occurs, methyldopa can be replaced by another central alpha-agonist. ^539^

There is a risk of rebound effect from the discontinuation of clonidine, especially when combined with beta-blockers, and can be dangerous in the preoperative period. ^537^ Gradual withdrawal over two to four weeks prevents the rebound effect. Medications in this class have adverse reactions due to their central action, such as drowsiness, sedation, dry mouth, fatigue, postural hypotension, and erectile dysfunction. ^537^ ^,^ ^539^

## 9.4.7. Alpha-blockers

Alpha-blockers act as competitive antagonists of postsynaptic alpha-1 receptors, decreasing PVR without changes in cardiac output. ^539^ They promote greater blood pressure decreases in standing position and in reflex tachycardia. Therefore, postural hypotension is common, often found after the first dose. The hypotensive effect is mild in monotherapy, the combined use being preferred. They have favorable and discrete action on the lipid and glucose metabolisms. ^539^ Medications in this class used as antihypertensives include doxazosin, prazosin, and terazosin.

A beneficial adjunct action of alpha-1 blockers is the relaxation of the pelvic floor musculature, which helps patients with benign prostatic hyperplasia (BPH) empty their bladders. Therefore, alpha-blockers are also used in men with BPH, particularly doxazosin, tamsulosin, alfuzosin, and silodosin.

### 9.4.7.1. Adverse Effects of Alpha-Blockers

Alpha-blockers may cause symptomatic hypotension on the first dose. Tolerance is a frequent phenomenon, requiring increasing doses to maintain the antihypertensive effect (tachyphylaxis). Alpha-blockers may cause urinary incontinence in women. There is evidence that patients treated with doxazosin are at higher risk for HF. ^539^

## 9.4.8. Direct-Acting Vasodilators

The oral medications in this class are hydralazine and minoxidil. They act directly by relaxing arterial smooth muscles, leading to a PVR decrease. ^539^

### 9.4.8.1. Adverse Effects of Direct-Acting Vasodilators

The side effects of hydralazine are headache, flushing, reflex tachycardia, and lupus-like reaction (dose-dependent). ^539^ In addition, it can cause anorexia, nausea, vomiting, and diarrhea. Vasodilators may cause sodium and fluid retention, with increased circulating volume and reflex tachycardia. A side effect of minoxidil is hirsutism, found in approximately 80% of the patients.

## 9.4.9. Direct Renin Inhibitors

Aliskiren, the only representative of this drug class commercially available, causes direct renin inhibition with consequent decrease in angiotensin II production. ^545^ Other actions may contribute to BP lowering and tissue protection, such as the reduction in plasma renin activity, ^545^ renin/prorenin receptor blockade, ^546^ and decrease in intracellular angiotensin II production. ^547^

Antihypertensive efficacy studies have confirmed its antihypertensive activity in monotherapy and in combination, at a similar level as other RAAS blockers and with the apparent additional benefit of lowering proteinuria in kidney disease patients. ^548^ ^,^ ^549^ However, there is no evidence of its benefits on CV morbidity and mortality for hypertensive and prehypertensive patients. ^550^ ^,^ ^551^

### 9.4.9.1. Adverse Effects of Direct Renin Inhibitors

They are well tolerated. Skin rashes, diarrhea (especially at high doses above 300 mg/day), creatine phosphokinase increases, and coughing may occur in less than 1% of patients. They are contraindicated during pregnancy for the same reasons as ACEIs and ARBs.

## 9.5. Antihypertensive drug combinations

Initial antihypertensive drug combination therapy seems to be associated with decreased risk of CV outcomes compared to the traditional onset of treatment with monotherapy. ^552^ Initial two-drug combinations, compared to sequential association promotes quicker control and may lower BP up to five times more, ^506^ with clear impact on EOD and long-term CV outcomes. A meta-analysis found that fixed-dose two-drug combinations improve adherence by 24% compared with a free-drug component regimen. ^553^ However, few studies focus specifically on assessing drug combinations on CV outcomes.

The ACCOMPLISH study ^554^ compared benazepril combined with hydrochlorothiazide and with amlodipine. The difference in systolic and diastolic BP between the two groups, though significant, was only 0.9/1.1 mm Hg lower in the amlodipine arm. There was a decrease in risk of primary outcomes, consisting of nonfatal AMI, stroke, hospitalization for unstable angina, myocardial revascularization surgery, and cardiopulmonary resuscitation, in favor of the benazepril-amlodipine group. The choice of hydrochlorothiazide in that study was criticized because its effect lasts less than 24 hours, unlike the longer-acting amlodipine. However, a different report found no significant differences in 24-hour BP across groups. ^555^ In patients with body mass index (BMI) > 30 kg/m ^2^ , there were no differences in primary outcomes between the two groups. ^556^ Another prespecified analysis found an addition decrease in kidney disease progressions from a benazepril-amlodipine combination. ^557^

The ASCOT-BPLA study ^558^ compared a strategy based on amlodipine adding perindopril as required versus atenolol adding bendroflumethiazide. Approximately 78% of patients in each group used combination therapy for hypertension control. There were no differences in primary outcomes, consisting of nonfatal AMI and fatal CAD, but secondary outcomes, such as stroke, fatal coronary events, CV mortality, and all-cause mortality, were all significantly lower for the amlodipine group. De novo diabetes development rates were 30% higher for the group treated with BBs and thiazide. The CAFE sub-study ^234^ found a more significant decrease in central aortic pressure from an amlodipine-perindopril combination and attributed it, at least in part, to the greater decrease in secondary outcomes for the group.

In the multicenter VALUE trial, ^527^ high CV risk patients received antihypertensive treatment based on valsartane or amlodipine. Approximately 25% of patients in both groups required the addition of 12 to 25 mg of hydrochlorothiazide for BP control. Despite the higher and earlier blood pressure decrease in the amlodipine group, the combined primary cardiac outcome at the end of four years was similar for both groups, as were the fatal AMI and all-cause mortality rates. There were fewer HF cases with valsartane and fewer nonfatal AMI and stroke cases with amlodipine.

In HOPE-3, ^309^ directed primarily to studying the effects of medications in prehypertensive patients at intermediate CV risk, an initial strategy based on a fixed-dose combination of candesartan and hydrochlorothiazide led to a 27% decrease in the composite outcome risk of CV death, nonfatal AMI, and stroke in stage 1 hypertension individuals. However, no benefits have been found for prehypertensives.

The PROGRESS trial, ^291^ which assessed patients with prior cerebrovascular disease; the ADVANCE trial, ^559^ which studied individuals with type 2 diabetes; and HYVET, ^560^ which studied patients 80 years of age or older, used an intervention based on perindopril and indapamide and showed the benefits of combining DIUs and ACEIs to lower issues such as stroke and vascular dysfunction; macro- and microvascular outcomes; and death, stroke, and HF, respectively.

Combining BBs and thiazides lowered CV outcomes when compared to a placebo in older trials, especially those involving older patients, ^509^ ^,^ ^561^ ^,^ ^562^ but underperformed the combination of thiazides and losartan in the LIFE trial, ^526^ where it provided less protection against stroke and favored glucose metabolic disorders. The use of fixed-dose combinations of thiazides with atenolol and other BBs should be restricted to specific indications for this class ^5^ ^,^ ^164^ ^,^ ^495^ given the induction of potential metabolic disorders from DIUs, such as insulin resistance, hyperglycemia, hyperuricemia, and hypopotassemia.

The combination of dihydropyridine calcium channel blockers and thiazide diuretics may be especially useful for older adults with isolated systolic hypertension or in cases where the use of RAAS blockers are contraindicated or restricted due to their potential risks, such as in women of childbearing age.

The polygon in Figure 9.2 shows the preferred (connected by a green line), contraindicated (red line) and possible but less often studied (dotted line) combinations. ^1^ In stage 3 hypertension and resistant hypertension patients, the goal is to optimize the triple treatment with preferred medications—ACEIs or ARBs, dihydropyridine CCBs, and thiazide or thiazide-like DIUs. ^37^ ^,^ ^503^ ^,^ ^504^ A clinical trial assessed the fixed triple combination of amlodipine, valsartan, and hydrochlorothiazide, all available in Brazil, for stages 2 and 3 HT patients, and found mean decreases of 39.7 mm Hg in systolic BP and 24.7 mm Hg in diastolic BP, significantly higher than two-drug combinations involving the same medications. ^563^

**c1232:** – Preferential associations of drugs according to mechanisms of action and synergy.

Source: Malachias et al., 2016.164

Failing to reach the blood-pressure target with triple therapy requires the use of a fourth drug, and the current preferred medication is spironolactone. ^37^ ^,^ ^564^ ^-^ ^567^ BBs, clonidine, ^564^ and doxazosin ^567^ are options for 4^th^or 5^th^drugs, and hydralazine ^164^ may also be added in cases of intolerance to any of the previous antihypertensive options and in resistant HT. ^503^ ^,^ ^504^ In the PATHWAY 2 study, ^567^ amiloride use was shown to be as effective as spironolactone, providing an alternate treatment for resistant HT. However, the medication is not available from manufacturers in Brazil. The ReHOT study ^564^ showed that the efficacy of clonidine is similar to that of spironolactone as the 4^th^medication for resistant HT patients. However, in 24-hour ambulatory BP analysis, spironolactone outperformed clonidine.

Treatment combining two renin-angiotensin system blockers, such as an ACEI with an ARB or any of the two with renin inhibitors, is contraindicated, since they lead to an increase in adverse effects without decreasing CV outcomes. ^568^ ^-^ ^569^

Chart 9.3 lists the major clinical trials that used combinations of antihypertensive medications, while Chart 9.4 shows the primary levels of evidence and level of recommendation of pharmacological treatments. Figure 9.1 shows the usual steps of combining medications for HT control.

**Table:** 

Key Takeaways
The primary objectives of antihypertensive treatment are lowering blood pressure and the risk of CV outcomes and mortality associated with hypertension.
Pharmacological treatment should be combined with nonpharmacological measures, and the preferred antihypertensive classes for use in monotherapy or combination therapies are: thiazide or thiazide-like diuretics, CCBs, angiotensin-converting enzyme inhibitors, angiotensin receptor blockers, and beta-blockers (with specific indications).
Combining medications is the first recommended strategy for moderate- to high-risk stage 1 hypertensive patients and stages 2 and 3 patients, preferably in a single pill. Monotherapy should be considered for low-risk stage 1 hypertensive patients and for oldest old and/or frail individuals.
Two-drug treatments should begin with an ACEI or ARB combined with a thiazide or thiazide-like DIU or a CCB. In nonobese high-risk patients, CCB combinations are preferred.
When two medications combined are unable to control BP, patients should be prescribed three drugs, usually an ACEI or ARB combined with a thiazide or thiazide-like DIU and a CCB; if needed, add spironolactone next.

**Chart 9.3:** – Drug combination studies for hypertension treatment

Study	Comparator regimen	Patient profile	Difference in SBP (mm Hg)	Primary outcome (% relative risk reduction)	p
**Combinations of diuretics**					
PREVER ^179^ (amiloride + chlorthalidone)	Losartan	Stage 1 hypertensive patients	-2.2	Not assessed. Greater SBP decrease with diuretics without glucose increase	–
**Association of ACE inhibitors and diuretics**					
PROGRESS ^291^ (perindopril + indapamide)	Placebo	Prior stroke or TIA	-9	-28% stroke	< 0.001
ADVANCE ^559^ (perindopril + indapamide)	Placebo	Diabetes	-5.6	-9% macrovascular and microvascular events	0.04
HYVET ^560^ (indapamide + perindopril)	Placebo	Hypertensive patients ≥ 80 years	-15	-34% CV events	< 0.001
**Combination of ACE inhibitors and calcium channel blockers (amlodipine)**					
ACCOMPLISH ^554^ (benazepril + amlodipine)	Benazepril + diuretic	High-risk hypertensive patients	-0.9	-19.6% compound CV events	< 0.001
ASCOT BPLA ^558^ (amlodipine + perindopril)	Beta-blocker + diuretic	Hypertensive patients with 3 or more risk factors	-2.7	Difference not significant *	NS
**Combination of angiotensin receptor blockers (olmesartan) and calcium channel blockers**					
COLM ^570^ (olmesartan + CCB)	Olmesartan + diuretic	Older hypertensive Japanese patients with CV disease or risk factors	0	Difference not significant	NS
**Combination of angiotensin receptor blockers and diuretics**					
LIFE ^526^ (losartan + diuretic)	Beta-blocker + diuretic	Hypertensive patients with LVH	-1.1	-13% CV events	0.02
Combination of calcium channel blockers and diuretics					
FEVER ^571^ (felodipine + diuretic)	Diuretic + placebo	Hypertensive patients	-4	-34% CV events	< 0.001
**Combination of calcium channel blockers and ACE inhibitors**					
SYST-EUR ^572^ (ACEI + ARB + diuretic)	Placebo	Older adults with ISH	-10	-31% CV events	< 0.001
SYST-CHINA ^573^ (ACEI + ARB + diuretic)	Placebo	Older adults with ISH	-9	-37% CV events	< 0.004
**Combinations of beta-blockers and diuretics**					
Coope and Warrender ^574^ (atenolol and diuretic)	Placebo	Older hypertensive patients	-18	-42% stroke	< 0.003
SHEP ^509^ (chlorthalidone and atenolol)	Placebo	Older hypertensive patients	-13	-36% stroke	< 0.001
STOP-H ^561^ (beta-blocker and diuretic)	Placebo	Older adults with ISH	-23	-40% CV events	< 0.004
STOP-H2 ^562^ (ACEI and CCB)	Standard treatment (BB and diuretic)	Older hypertensive patients	0	No difference in CV events	–
**Combination of two renin-angiotensin system antagonists**					
ONTARGET ^568^ (telmisartan + ramipril)	ACEI or ARB	High-risk patients	–	Worse renal outcomes	–
ALTITUDE ^569^ (aliskiren + ARB)	ACEI or ARB	High-risk diabetic patients	–	Worse renal outcomes	–
**Combination of fixed-dose calcium channel blocker, angiotensin receptor blocker, and diuretic**					
Calhoun et al. ^563^ (ARB + diuretic + CCB)	ARB + diuretic^a^or CCB^b^+ diuretic or ARB + C^C^Bc	Stage 2 and 3 hypertensive patients	a: -7.6 b: -8.2 c: -6.2	Not assessed	–
TRIUMPH ^575^ (telmisartan + amlodipine + chlorthalidone)	Usual treatment at the end of 6 months: Monotherapy in 65% and two-drug combination in 29%	Hypertensive patients	-8.8	Not assessed	–

Adapted from ESC, 2018.^*37*^ISH: isolated systolic hypertension; LVH: left ventricular hypertrophy; NS: not significant; TIA: transient ischemic attack. * significant differences in various secondary outcomes favoring ACEI + amlodipine; (a) ARB + diuretic (b) ARB + diuretic or CCB (c) diuretic or ARB + CCB.

**Chart 9.4:** – Pharmacological treatment: levels of evidence and level of recommendation

Drug combinations	LE	LR
The preferential medication classes for antihypertensive treatment are thiazide or thiazide-like DIUs, CCBs, ACEIs, and ARBs, as they have been shown to effectively lower BP and the risk of CV outcomes. BBs should be considered for specific clinical scenarios (CAD, HF and HR control)	A	I
HT treatment may be initiated with two-drug class combinations starting in stage 1 HT	B	I
Two-drug treatments should begin with an ACEI or ARB combined with a thiazide or thiazide-like DIU or a CCB	A	I
HT treatment for high CV risk patients combining an ACEI and a dihydropyridine CCB is preferred over combining an ACEI and a thiazide DIU for nonobese patients	B	I
When two medications combined are unable to control BP, patients should be prescribed three drugs, usually an ACEI or ARB combined with a thiazide or thiazide-like DIU and a CCB	A	I
When three medications combined are unable to control BP, preference should be given to adding spironolactone to the therapy regimen	B	I
HT treatment with fixed combinations enables higher adherence rates	B	IIa
HT treatment combining two renin-angiotensin system antagonists is contraindicated	A	III

## 10. Hypertension And Associated Clinical Conditions

## 10.1. Diabetes Mellitus (DM)

Hypertension (HT) is a frequent finding in DM patients, especially type 2 diabetes. Evidence shows the benefits of lowering BP for this population, with subsequent lower rates of macro and microvascular events and lower mortality. These include lower rates of chronic kidney disease (CKD), ^307^ ^,^ ^329^ diabetic retinopathy, and albuminuria. ^576^ Current data show a major reduction in cardiovascular (CV) risk for DM patients, though it remains a high-prevalence illness and an important risk factor (RF) for cardiovascular disease (CVD). ^577^ ^,^ ^578^ The relationship between DM and HT provides relevant data, such as the presence of HT in 40% of recently-diagnosed patients with type 2 DM ^579^ and that 50% of type DM patients become hypertensive before the onset of albuminuria. This population is at high CV risk, so assessing urinary albumin and creatinine excretion, fundus health, and dysautonomia should be part of the investigation. ^580^

### 10.1.1. Treatment Objectives

Randomized clinical trials show the benefits of antihypertensive treatment for this population, such as the lower incidence of stroke, coronary syndromes, and CKD when BP levels below 140/90 mm Hg are achieved. In a meta-analysis of 13 clinical trials involving DM patients, systolic blood pressure (PAS) between 131 and 135 mm Hg decreased all-cause mortality risk by 13%, while more intensive SBP control to ≤ 130 mm Hg was associated with a greater decrease in strokes. ^581^ A second meta-analysis found significant decreases in mortality from achieving a mean SBP of 138 mm Hg and a significant decrease in strokes with a mean of 122 mm Hg. ^576^ Therefore, BP control is important to lower the risk of micro and macrovascular complications and should be maintained if these benefits are to be sustained (LR: I, LE: A).

Rigorous nonpharmacological treatment is required for all diabetic hypertensive patients. Office BP ≥ 140/90 mm Hg indicates the need for pharmacological treatment. All medications used in lowering BP may be administered to diabetic patients. The evidence supports the preferential use of RAAS blockers, particularly for patients with end-organ damage (EOD). ^526^ ^,^ ^582^ ^-^ ^584^ BP often requires combination therapy, and calcium channel blockers (CCBs) and/or diuretics (DIUs) are the recommended drug classes for combinations with RAAS inhibitors. ^506^ ^,^ ^585^ Combining two or more classes in a single galenic formulation should be considered, taking into account that adherence to treatment is paramount in this high-risk population.

## 10.2. Metabolic Syndrome (MS)

MS is characterized by a set of CV risk factors, including central obesity, high glycemia, and typical dyslipidemia (high triglycerides and low HDL-cholesterol levels) associated with increased BP. ^586^ ^-^ ^588^ These metabolic changes are found in 30 to 40% of HT patients, ^589^ and the presence of high blood pressure (BP) in MS increases global CV risk by triggering mechanisms associated with prothrombotic and pro-inflammatory states. ^590^ Therefore, investigating metabolic alterations from MS and central obesity is indispensable for HT patients. Lifestyle changes (LSCs) for weight loss, lower sodium intake, and controlling dysglycemia and dyslipidemia are recommended for all patients in these conditions. ^591^ Pharmacological treatment should be initiated whenever BP ≥ 140/90 mm Hg, since there is no evidence of benefit in the use of antihypertensive agents for MS with normal BP levels. ^592^ The choice of antihypertensive medications should prioritize therapeutic classes capable of improving insulin resistance, or at least not make it worse, such as angiotensin-converting enzyme inhibitors (ACEIs), angiotensin II AT_1_receptor blockers (ARBs), and CCBs. DIUs and beta-blockers (BBs), except for direct-acting vasodilators, may be indicated as additional medications. ^593^

## 10.3. Coronary Artery Disease (CAD)

Robust epidemiological evidence connects HT to CAD. Data from the INTERHEART study show that 25% of infarctions (AMI) may be attributable to HT. ^286^ A meta-analysis assessing the impact of BP found a mean decrease of 17% in CAD for every 10 mm Hg decrease in SBP. ^85^

Treatment for HT associated with CAD, which includes post-AMI patients with chest angina and myocardial revascularization (MRV), should preferably comprise BBs, ACEIs, or ARBs, in addition to statins and acetylsalicylic acid. Beta-blockers are beneficial after AMI, especially within 2 years from the acute event. ^83^ Similarly, ACEIs and ARBs tested on that condition have also proven beneficial. ^83^ ^,^ ^594^ ^,^ ^595^ In patients with chronic CAD and multiple risk factors, such as HT, ACEIs have been found to lower relevant clinical outcomes ^596^ (LE: I; LR: A).

Regarding BP target, it is worth considering the likelihood of the J-curve effect, found in different studies, ^597^ ^-^ ^600^ in which the excessive BP decreases, mainly in diastolic blood pressure (DBP), can trigger CV events in patients with obstructive CAD. Thus, the goal is to achieve SBP < 130 mm Hg and DBP < 80 mm Hg (LR: IIa; LE: B), while levels below 120/70 mm Hg should be avoided. ^601^ Additional medications, such as CCBs and thiazide diuretics, ^181^ may be administered to achieve those BP targets.

## 10.4. Hypertension in Chronic Kidney Disease (CKD)

### 10.4.1. Patient in Conservative Treatment: Goals and Treatment

In CKD, BP levels to be achieved remain undetermined, and the evidence depends on associated morbidities. ^602^ Nondiabetic patients treated with strict targets (< 130/80 mm Hg) showed slower disease progression only in subgroups with proteinuria, and CV events could not be assessed ^603^ ^,^ ^604^ (LR: IIa; LE: A). On the other hand, a meta-analysis has found lower mortality with intensive treatment for HT. ^605^ In diabetic patients, strict targets led to decreased albuminuria, improved retinopathy and fewer strokes, but no impact on other CV outcomes ^581^ ^,^ ^606^ ^,^ ^607^ (LR: IIa; LE: A).

A study with 9361 nondiabetic patients, out of which 2646 had CKD, found a decrease in CV events in the order of 25% for the treatment group that sought SBP below 120 mm Hg. This suggests the probable benefits provided by this strategy in CV protection for CKD patients ^86^ (LR: I; LE: A).

In terms of pharmacological treatment, ACEIs or ARBs are indicated for hypertensive patients with or without albuminuria, and its use in combination therapy is proscribed ^608^ (LR: I; LE: A). Thiazide ^83^ or loop diuretics, the latter for G4 and G5 CKD, and CCBs are effective, especially in combination with ACEIs or ARBs ^609^ (LR: I; LE: A). BBs are indicated for CAD and associated heart failure (HF). ^610^ Mineralocorticoid receptor antagonists lower proteinuria, but may cause hyperpotassemia. Clinical trials of new antagonists in this class are in progress. ^611^

**Table:** 

This guideline recommends a BP target < 130/80 mm Hg for adults with HT and CKD, whether diabetic or not. Stricter targets may be sought in select cases, under strict vigilance and after patients have been informed of the risks.

#### 10.4.2. Patients in Renal Replacement Therapy (RRT): Goals and Treatment

Managing HT in patients undergoing dialysis treatment can be a challenge, especially due to the volume overload that increases BP variability, overestimating it pre-dialysis and overestimating it afterward. ^612^ There is no evidence regarding optimum BP levels for dialysis patients, but the most often accepted values immediately before and after hemodialysis (HD) are ≤ 140/90 mm Hg and ≤130/80 mm Hg, respectively ^613^ ^,^ ^614^ (LR: IIa; LE: C). For these patients, there is a (paradoxical) U-shaped association between SBP measured at the dialysis unit and CVD risk, with values above 160 mm Hg or < 110 mm Hg implicated in increased mortality ^614^ ^,^ ^615^ (LR: IIa; LE: B).

In this population, home BP readings are more reproducible, provide relevant information for therapeutic decisions, and are better associated with BP control ^613^ (LR: IIa; LE: B). Systolic means from home measurements are linearly associated with increased CV risk. ^615^ ^,^ ^616^ In addition to hypervolemia, arterial stiffness is an important cause of systolic hypertension in stage 5D CKD patients. This specific phenotype reflects the acceleration of the atherosclerosis process and premature vascular aging in this population. ^616^ Other mechanisms, such as sleep apnea, ^617^ sympathetic hyperactivity, ^618^ and erythropoietin use, should also be considered. ^616^

HT treatment for patients undergoing dialysis treatment is only effective for 1/3 of individuals, and is even harder to achieve due to hemodynamic instability during sessions, which may cause intradialytic hypotension or hypertension, leading to poorer CV prognoses ^619^ (LR: IIa; LE: B).

Treatment should start with measures focusing on achieving “dry weight,” such as salt and water restriction and ultrafiltration in dialysis ^620^ (LR: IIa; LE: A). Regardless, approximately 60% of dialysis patients require three or more antihypertensives, in several combinations, to control HT ^620^ (LR: IIa; LE: A). In this population, sympathetic nervous system (SNS) hyperactivity has a major role in the origins of HT and in CVD. Accordingly, beta blockade was superior to ACE inhibition in preventing CV morbidity and in BP control for patients with left ventricular hypertrophy (LVH) undergoing hemodialysis treatment. ^618^ In kidney transplant patients, CCBs and ARBs are the first option, since there is evidence that they prevent graft loss ^621^ ^,^ ^622^ (LR: I; LE: A).

**Table:** 

This guideline suggests the individualization of treatment in RRT, considering comorbidities, pharmacokinetics, and the cardioprotective effect of medications.

## 10.5. Heart Failure (HF)

HT has a key role in the pathophysiology of HF, leading to the onset of LVH and left ventricular diastolic and systolic dysfunction. ^623^ ^-^ ^625^ It is the greatest RF for the disease, and usually precedes the clinical syndrome by several years. In HF with preserved ejection fraction (HFpEF), HT is even more frequent, and it is the most common RF, with prevalence rates of up to 90%. ^626^

Early diagnosis of HT and adequate treatment can significantly lower the risk of developing HF, especially for older adults. Pharmacological strategies for BP control promote decreases of approximately 50% in HF incidence in adults, and a 64% decrease for those age 80 and older. ^560^ ^,^ ^627^ ^-^ ^628^ The SPRINT trial, with a stronger SBP decrease target (< 120 mm Hg) for a high CV risk population, found a decrease of 27% in total mortality and 38% in progression to HF. ^86^ ^,^ ^629^ The impact of antihypertensive treatment on preventing HF has been found for various classes of antihypertensive medication, such as BBs, DIUs, CCBs, and ACEIs. ^630^

BP targets in HF settings should be similar to those recommended for high CV risk individuals, ie, < 130/80 mm Hg. ^631^ HT treatment in HF should consider its presentation, ie, whether with preserved ejection fraction (HFpEF) or reduced ejection fraction (HFrEF) ( Chart 10.1 ). The first therapeutic antihypertensive option for HFrEF should supplement medications promoting neurohormonal blockade, be dose-optimized, and have scientific evidence proving it reduces mortality. ^632^ These medications are RAAS blockers, BBs, and aldosterone antagonists. The sacubitril/valsartane combination is a new treatment option impacting mortality reduction in HFrEF, but still with no evidence of benefits for HT. ^633^

**Chart 10.1:** – Antihypertensive treatment for heart failure patients

Antihypertensive treatment for HF patients		
Recommendations	RC	LE
**The blood-pressure target for HT and HF patients (REF and PEF) should be < 130/80 mm Hg**	I	C
**In HFrEF, antihypertensive medications with proven effect on mortality rates (BBs/ACEIs/ARBs/spironolactone) should be used**	I	A
**In HFpEF, all antihypertensive drugs may be used.**	I	C
**Nondihydropyridine calcium channel blockers and alpha-blockers are contraindicated for HFrEF.**	III	C

*HFpEF: heart failure with preserved ejection fraction; HFrEF: heart failure with reduced ejection fraction; LE: level of evidence; RC: recommendation class.*

If blood pressure levels remain high despite the neurohormonal blockade, they can also be combined with DIUs; vasodilator hydralazine-nitrate combination; or dihydropyridine CCBs. Nondihydropyridine CCBs, such as diltiazem and verapamil, and BBs are contraindicated. ^631^

Due to the strong association between HFpEF and HT, antihypertensive treatment is indicated for most patients. Diuretics should be used for BP control and for symptoms connected to hypervolemia, but randomized clinical trials have not found lower mortality rates for HFpEF patients. Despite the lack of evidence about the benefits of SNS and RAAS blockers to lower HFpEF mortality, these substances should still be used for blood pressure control. ^634^ ^-^ ^639^ Other classes of antihypertensives may also be used.

The relationship between SBP levels and CV mortality follows a J-shaped curve, especially for HFrEF. ^640^ ^,^ ^641^ Data from clinical trials such as Copernicus, DigTrial, Val-HeFT and PARADIGM-HF ^318^ ^,^ ^642^ ^-^ ^644^ have verified the relationship between lower BP values and higher mortality rates.

In HFpEF, the association between BP levels and clinical outcomes remains controversial. ^317^ ^,^ ^318^ In that patient group, BP levels should be kept within the 120-129/70-79 mm Hg target range.

10.6. Hemorrhagic Stroke and Ischemic Stroke

Ischemic and hemorrhagic stroke are the most frequent manifestations of the vascular damage caused by HT and are the leading cause of death and impairment for those patients. ^292^ Preventing all forms of stroke is possible by achieving BP target levels with adequate treatment (see Chapter 6) ^291^ ^,^ ^645^ ^-^ ^652^ (LR: IIa; LE: B).

### 10.6.1. Hemorrhagic Stroke

High BP increases the likelihood of hematoma expansion, leading to worse prognosis and increased risk of death. ^653^ Robust studies suggest that lowering BP (within 6 h) to values <140/90 mm Hg does not lower the rates of primary events, including mortality. ^654^ Therefore, immediately lowering BP for hemorrhagic stroke cases is not recommended unless SBP levels are > 220 mm Hg.

### 10.6.2. Ischemic Stroke

The benefits of lowering BP for strokes are less clear, but should be considered for thrombolysis candidates, since, in their case, if BP > 180/105 mm Hg, there may be a greater risk of hemorrhaging. ^655^ ^,^ ^656^ A meta-analysis suggests that lowering blood pressure in ischemic strokes may have a neutral effect on mortality. ^657^ ^,^ ^658^

Chart 10.2 , adapted from the ESC and ESH guidelines, ^37^ lists therapeutic targets and recommendations for acute stroke and cerebrovascular disease patients.

**Table:** 

Key Takeaways
BP control is important to lower the risk of micro and macrovascular complications and should be maintained if these benefits are to be sustained (LR: I, LE: A). Office BP ≥ 140/90 mm Hg indicates the need for pharmacological treatment preferably accompanied by LSCs and the use of RAAS blockers, while DIUs and CCBs may be added to achieve a blood-pressure target of <140/90 mm Hg.
Pharmacological treatment should be initiated for MS whenever BP ≥ 140/90 mm Hg, prioritizing the use of metabolically neutral antihypertensives or those that improve insulin sensitivity, such as ACEIs, ARBs, and CCBs.
In CKD patients, the BP target is <130/80 mm Hg, and may be stricter in select cases. In dialysis patients, achieving “dry weight” is key. Approximately 60% of dialysis patients require 3 or more antihypertensives, in several combinations, to control HT. For kidney transplant patients, CCBs and ARBs represent their first therapeutic option.
Immediately lowering BP for hemorrhagic stroke cases is not recommended unless SBP levels are ≥ 220 mm Hg; if so, use IV medications, with target SBP 180 mm Hg.
For HT and HF (PEF and REF) patients, the blood-pressure target should be <130/80 mm Hg. In HFrEF, blood pressure levels should be controlled with BBs, ARBs, and spironolactone, while all antihypertensives may be used for HFpEF.
Treatment for HT associated with CAD, which includes post-AMI patients with chest angina and myocardial revascularization (MRV), should preferably comprise beta-blockers, ACEIs, or ARBs, in addition to statins and acetylsalicylic acid, with a blood-pressure target of < 130/80 mm Hg.
J- or U-shaped curves are often seen in CAD patients, and levels below 120/70 mm Hg are to be avoided. In CKD, especially in dialysis patients, SBP levels above 160 mm Hg or <110 mm Hg have been implicated in increased mortality.

**Chart 10.2:** – Therapeutic targets and recommendations for acute stroke and cerebrovascular disease patients

Recommendations	RC	LE
**AVEH Do not lower BP in patients with SBP < 220 mm Hg If SBP ≥ 220 mm Hg, use IV medications, with target SBP 180 mm Hg**	III IIa	A B
**AVEI Lowering BP is not recommended, except for patients eligible for thrombolysis. In that case, maintain BP < 180/105 mm Hg**	III IIa	B B
**Transient ischemic attack Immediate decrease**	I	A
**Goal: keep SBP between 120 and 130 mm Hg after event**	I	A
**Secondary prevention Use renin-angiotensin system blockers + calcium channel blockers or thiazide diuretics**	I	A

*LE: level of evidence; RC: recommendation class; SBP: systolic blood pressure.*

## 11. Hypertension in Pregnancy

## 11.1 Epidemiology

Hypertensive disorders of pregnancy is one of the leading causes of maternal and perinatal mortality throughout the world. From 0.9 to 1.5% of pregnant women have chronic hypertension, and it is estimated that pre-eclampsia (PE) complicates 2 to 8% of pregnancies globally. ^659^ ^,^ ^660^ Those syndromes are causal factors associated with perinatal and maternal death, may permanently impair maternal health and may cause major issues from preterm birth associated with early indications for intervention (elective preterm birth). ^660^ In Brazil, PE is the leading cause of provider-initiated preterm birth, ^661^ and the estimated incidence rates are 1.5% for PE and 0.6% for eclampsia. ^662^ The prevalence of eclampsia in the more developed regions of Brazil is 0.2%, with an 0.8% mortality rate, ^661^ while prevalence rates in less developed regions rise to 8.1%, with a corresponding mortality rate of 22.0%. ^663^

## 11.2. Classification of Hypertension in Pregnancy

We recommend the definitions and classification put forth by the A *merican College of Obstetricians and Gynecologists* (ACOG), ^664^ ^,^ ^665^ which can be found in Chart 11.1 (LR: IIb, LE: B).

**Chart 11.1:** – Definition and classification of hypertensive disorders in pregnancy

DEFINITIONS	
Gestational hypertension	SBP ≥140 mm Hg and/or DBP ≥ 90 mm Hg, or both, measured twice and at least two hours apart.
**Severe gestational hypertension**	SBP ≥ 160 mm Hg and/or DBP ≥110 mm Hg, or both, measured twice and at least two hours apart.
**Proteinuria**	Proteinuria > 300 mg in 24 h, urine protein/creatinine ratio of 0.3 g/g of creatinine or ++ in reagent strips (quantification is ideal).
**CLASSIFICATION**	
**Pre-eclampsia (with or without severe features)**	SBP ≥140 mm Hg or DBP ≥ 90 mm Hg, or both, in general after 20 weeks of gestation and often with proteinuria*. In the absence of proteinuria, the diagnosis can be made in the presence of severe features: thrombocytopenia (< 100 000 109/L), creatinine > 1.1 mg/dL or 2x baseline creatinine, two-fold elevation in liver transaminases, APE, abdominal pain, visual symptoms or headaches, seizures, no alternative diagnoses.
**Chronic hypertension**	HT diagnosed or present before pregnancy or before 20th gestational week; or HT first diagnosed during pregnancy but that does not normalize after childbirth.
**Chronic hypertension with overlapping pre-eclampsia**	Pre-eclampsia in women with a history of HT before pregnancy or before the 20th gestational week.
**Gestational hypertension**	SBP ≥140 mm Hg or DBP ≥90 mm Hg, or both, in women with previously normal BP, after 20 gestational weeks, measured twice and at least 4 hours apart, without proteinuria or severe features, and returning to normal after childbirth.
**OTHER DIAGNOSTIC DEFINITIONS**	
**Eclampsia**	Tonic-clonic seizures in the absence of other causal conditions.
**HELLP Syndrome**	Hemolysis, elevated liver enzymes, and thrombocytopenia.
**Posterior reversible encephalopathy syndrome (PRES) and reversible cerebral vasoconstriction syndrome**	PRES with imaging abnormalities is established by the presence of vasogenic edema and hyperintensities in the posterior aspect of the brain in MNR, as well as association with visual disturbances, seizures, headaches, and sensory alterations. Reversible cerebral vasoconstriction syndrome is characterized by the narrowing of cerebral arteries with thunderclap headache or focal neurological signs.

*APE: acute pulmonary edema; BP: blood pressure; DBP: diastolic blood pressure; HT: hypertension; PRES: posterior reversible encephalopathy syndrome; SBP: systolic blood pressure.*

## 11.3. Concept and Diagnostic Criteria

Hypertension in pregnancy is defined as the presence of systolic blood pressure (SBP) ≥ 140 mm Hg and/or diastolic blood pressure (DBP) ≥ 90 mm Hg, considering the fifth Korotkoff sound, confirmed by another measurement after a 4-hour interval. Ideally, the measurement should be taken with the patient sitting down or in left lateral decubitus position, with a properly sized cuff. The manual auscultatory method is the gold standard, since automated devices may underestimate blood pressure (BP), especially in severe pre-eclampsia. Ambulatory blood pressure monitoring (ABPM) is superior to BP measurements at the physician's office and home blood pressure monitoring (HBPM) for nonpregnant women. For pregnant women, it helps avoid unnecessary treatment for white coat hypertension (WCH) and is useful in managing high-risk gestational hypertension and in detecting masked hypertension. ^37^ The role of ABPM and HBPM is still controversial in pregnancy. The International Society for the Study of Hypertension in Pregnancy (ISSHP) recommends the use of ABPM before the 20th gestational week and HBPM for follow-up. ^666^ The cutoff point for HT is ≥ 135/85 mm Hg for daytime ABPM and ≥ 130/80 mm Hg for HBPM (Chapter 3).

The definition and classification of hypertensive disorders in pregnancy can be found in Chart 11.1 .

## 11.4. Prediction and prevention of pre-eclampsia

Calcium supplementation (> 1 g/day) is not recommended for pregnant women with normal calcium intake ^667^ (LR: III, LE: A), but is recommended for those with low calcium intake and at intermediate to high risk of pre-eclampsia ^667^ (LR: I, LE: A).

Low doses of acetylsalicylic acid (ASA) (75- 150 mg/day) at the end of the first gestational trimester can be useful for primary prevention of pre-eclampsia in pregnant women at intermediate to high risk of pre-eclampsia ^668^ ^-^ ^670^ (LR: I, LE: A). However, its use is not recommended in the absence of risk ^669^ (LR: III, LE: A).

Pre-eclampsia prediction should preferentially be performed during the 1st semester using assessment methods that take into consideration maternal clinical history (risk factors) associated with Doppler ultrasound to check for flow resistance in the uterine arteries. There are also promising laboratory tests to assess angiogenesis, such as serum soluble endoglin, PIGF (placental endotelial growth factor), sFlt-1 (soluble fms-like tyrosine kinase receptor-1), and sFlt-1/PlGF ratio, but which are still not available in clinical practice. ^666^

In patients at high risk for PE, the use of calcium for low-intake populations (< 600 mg/day), a dose of 1.0 to 2g/day effectively lowers the risk of PE. ^667^ Chart 11.2 summarizes the use of ASA at low doses (75-150 mg/day) for eclampsia prevention. It should be initiated preferably before the 16th week, with no increase in maternal or fetal complications, and is recommended by international guidelines such as NICE 2019, ^671^ the WHO's ^672^ and that of the American College of Obstetricians and Gynecologists (ACOG). ^664^ A study ^670^ involving 1776 patients, comparing acetylsalicylic acid at 150 mg and a placebo, starting at 11 to 14 weeks, found a total event rate (PE) of 1.6% for the acetylsalicylic acid group and 4.3% for the placebo group (OR: 0.38, 95% CI 0.2 to 0.74, p = 0.004). This confirms the protective effect of acetylsalicylic acid for high-risk pregnant women.

In its 2019 report on screening and prevention, ^673^ the International Federation of Gynecology and Obstetrics (FIGO) suggests the use of the Fetal Medicine Foundation risk calculator to determine when acetylsalicylic acid is indicated in pre-eclampsia prevention. This useful tool is available at https://fetalmedicine.org/research/assess/pre-eclampsia/first-trimester.

**Chart 11.2:** – Recommendations for ASA use in pre-eclampsia prevention

Risk	Risk factor	Recommendation
High	Prior PE with adverse fetal outcome Multiple gestation Chronic HT DM type 1 or 2 Kidney disease Autoimmune disorder (SLE/APS)	Low-dose acetylsalicylic acid is recommended for 1 or more of these criteria.
Moderate	Nulliparity Obesity (BMI ≥ 30 kg/m ^2^ ) Family history of PE (mother or sister) Age ≥ 35 years Poor obstetric history (SGA, preterm, low weight, more than ten years between pregnancies)	Consider using low-dose acetylsalicylic acid if patient has more than one risk factor.

*APS: antiphospholipid antibody syndrome; BMI: body mass index; DM: diabetes mellitus; HT: hypertension; PE: pre-eclampsia; SGA: small for gestational age; SLE: systemic lupus erythematosus.*

## 11.5. Nonpharmacological Treatment

For SBP persisting above 160 mm Hg for more than 15 minutes, nonpharmacological treatment alone should not be used (LR: III, LE: B). Relative rest at hospital or day hospital with monitoring is suggested for pre-eclampsia (LR: IIa, LE: B). Hospitalization is indicated for patients with severe gestational hypertension (LR: I, LE: B).

Nonpharmacological treatment alone should not be used to treat severe HT persisting for > 15 min ^674^ to prevent irreversible neurological damage, since SBP values > 155 mm Hg, especially > 160 mm Hg, are detected immediately before a stroke. ^675^ Severe diastolic hypertension (> 105 or 110 mm Hg) does not develop before most strokes in pregnant women with severe pre-eclampsia. ^676^

A systematic review found no differences in outcomes between strict rest and relative rest for pregnant women with hypertension and proteinuria. ^677^ Relative rest at hospital, as compared with routine house activity, reduces the risk of severe hypertension. Rest is not routinely indicated for gestational hypertension. ^677^ Prenatal care units and hospital admissions have similar clinical outcomes for mothers and newborns, but women may prefer treatment at day hospitals. ^678^

Although there is no indication for specific care during hospitalization, maternal and fetal monitoring is required. Blood pressure should be measured periodically, with daily weight and diuresis assessment, and patients should be instructed about warning signs. Laboratory tests (CBC with platelet count, liver enzymes, uric acid, creatinine and proteinuria) should be performed once to twice a week. Fetal follow-up comprises assessment of growth, movements, well-being and biophysical profile, as well as ultrasound.

## 11.6. Expectant Management

Expectant management is not recommended after 37 gestational weeks for women with gestational hypertension and prehypertension ^679^ (LR: III, LE: B). Expectant management is suggested between the 34th and 37th gestational weeks for stable women, without clinical worsening or severe hypertension ^680^ (LR: IIa, LE: B).

Premature delivery for patients with PE can be associated with decreased mortality. ^681^ Optimum delivery time before the 32nd to 34th weeks poses a dilemma due to the uncertainty in the balance between maternal safety (end of pregnancy) and fetal maturity (expectant). ^681^ After the 34th week, survival is high and baby and placenta delivery is effective in developed countries. ^681^ Physicians tend to delay birth until the 37th week, if it is considered safe.

The HYPITAT study compared induction of labor versus expectant monitoring for severe hypertension or PE without signs of severity (at the time, called mild PE) after the 36th week. ^679^ Women in the intervention group had a 29% lower risk of worse maternal outcomes, without affecting neonatal outcomes, suggesting that expectant treatment up to 37 weeks is not indicated. ^679^ In the HYPTAT-II study, in nonsevere HT between the 34th and 37th gestational weeks, expectant management increased maternal risk as compared to immediate delivery, but decreased the occurrence of neonatal respiratory distress syndrome. ^680^ Therefore, immediate delivery is not justified, and expectant monitoring should be considered until the clinical situation worsens. If labor inducing labor before the 34th week is indicated and both maternal and fetal clinical statuses allow a 48-hour wait to see if the situation resolves, corticosteroid use for fetal pulmonary maturation may be considered. ^682^

## 11.7. Pharmacological Treatment

Urgent pharmacological treatment is indicated for severe hypertension ^674^ ^,^ ^675^ and in the presence of warning signs (LR: I, LE: B). There is no consensus BP value to indicate when pharmacological treatment should be initiated. Pharmacological treatment should be initiated when BP is above 150-160/100-110 mm Hg ^665^ ^,^ ^674^ ^,^ ^676^ with the goal of keeping it in the 120-160/80-100 mm Hg range (LR: IIb, LE: B).

The choice of the antihypertensive medication depends on the attending physician’s experience and familiarity with the drug chosen and its side effects ^683^ (LR: IIb, LE: B). The use of ACEIs, ARBs, and direct renin inhibitors is contraindicated in pregnancy (LR: I; LE: B), and atenolol and prazosin should be avoided if possible ^683^ ^,^ ^684^ (LR: IIa, LE: B).

Magnesium sulfate is recommended for eclampsia prevention and treatment (LR: I, LE: B). To avoid maternal deaths, SBP > 150-160 mm Hg should indicate urgent treatment, ^676^ in line with other Brazilian and international guidelines, which set the cutoff point at 160 mm Hg. ^164^

When to initiate pharmacological treatment for pregnant hypertensives with BP below 160/110 mm Hg is still controversial, except for pregnant women with end-organ damage (EOD). Cochrane's systematic review ^685^ showed that treating mild to moderate HT does not significantly lower maternal, fetal, and newborn morbidity.

However, the CHIPS trial, ^686^ ^,^ ^687^ which assessed aggressive treatment (DBP up to 85 mm Hg) versusnonaggressive treatment (DBP up to 100 mm Hg) in a post-hoc analysis, found a major increase in severe hypertension and unfavorable fetal outcomes, such as miscarriages, ICU stays longer than 48 hours, preterm birth, and low weight. Thus, new studies are assessing whether to administer medication starting at 140/90 mm Hg. ^665^

According to ACOG, the HT control target should be SBP > 120 and < 160 mm Hg, and DBP > 80 and < 110 mm Hg, since both hypertension and induced hypotension may harm placental perfusion and, consequently, fetal growth. The goal is to prevent the progression of EOD and cardiac and cerebrovascular complications, as well as obstetric and fetal complications. ^665^

Pharmacological therapy should begin as monotherapy using first-line medications (methyldopa, long-acting nifedipine, or beta-blockers, except atenolol). If proper control is not achieved, combine it with another first-line medication or a second-line one (thiazide diuretic, clonidine, and hydralazine); avoid combining medications from the same pharmacological class. Angiotensin-converting enzyme (ACE) inhibitors and angiotensin receptor blockers (ARBs) are formally contraindicated in case of pregnancy due to the risk of fetal malformation, which may lead to intrauterine renal failure, as are mineralocorticoid receptor antagonists, due to hormonal blockade, and atenolol, due to the high risk of fetal growth restriction associated with its use. Diuretics should also be avoided for PE patients due to the possibility of increasing intravascular volume depletion. ^665^ ^,^ ^688^ A study comparing the efficacy of labetalol, long-acting nifedipine and methyldopa for managing severe gestational hypertension suggests that all medication classes were viable options, but long-acting nifedipine was more effective than labetalol and methyldopa. ^689^

Hypertensive emergencies in pregnant women may be treated with oral nifedipine (10 mg) or with IV hydralazine. Currently, the trend is to prefer nifedipine 10 mg, which may be repeated in 10 to 20 mg orally every 20 to 30 minutes, and if patients are unresponsive after the third dose, 5 mg of IV hydralazine every 20 to 30 minutes up to a dose of 15 mg. ^674^

In exceptional situations, such as acute pulmonary edema and refractory severe hypertension, the use of sodium nitroprusside may be considered the preferential option for urgent BP control ^690^ for a maximum of 4 hours due to the risk of fetal cyanide poisoning.

In postpartum hypertension for nonchronic hypertensive patients, HT usually resolves within the 1^st^week (5 to 6 days), but the risk of complications such as stroke, acute pulmonary edema (APE), and renal failure remains during this period. There is also risk of eclampsia during this period, and 32 to 44% of women may have postpartum seizures. ^691^

Postpartum women may take all antihypertensive medications, and breastfeeding is the only limiting factor. Therefore, physicians should prioritize antihypertensives excreted in breast milk to a lesser extent.

Chart 11.3 lists the main antihypertensive medications available in Brazil in terms of breastfeeding. ^692^ ^-^ ^694^ Hypertensive crises in postpartum women may be treated in the conventional manner. A study comparing captopril and clonidine for HT control (SBP ≥ 180 mm Hg ad DBP ≥ 110 mm Hg) found no significant difference between the two, only a tendency for clonidine to perform better on the 3^rd^day postpartum. ^695^ Both were considered safe and effective for treating hypertensive emergencies in postpartum women. ^696^

**Chart 11.3:** – Actions of medications on breastfeeding

Drugs	Excretion in breast milk	Breastfeeding
**Nifedipine**	Little excretion	Allowed
**Amlodipine**	Insufficient studies	Unclear (apparently safe)
**Diltiazem, verapamil**	Insufficient studies	Unclear (use other medication)
**Clonidine**	Increased excretion	Avoid
**ACEI: enalapril, captopril**	Little excretion	Allowed without restrictions Allowed Allowed
**Lisinopril, ramipril**	Insufficient studies	Unclear (apparently safe)
**ARB: losartan, valsartan, candesartan, olmesartan, telmisartan**	Insufficient studies	Unclear (use other medication)
**Hydrochlorothiazide**	Little excretion	Use low dosage (< 50 mg)
**Chlorthalidone**	Little excretion	Slow elimination in newborns – avoid. Use low dosage
**Furosemide**	Insufficient studies	May decrease breast milk supply. Use only in case of clinical need
**Spironolactone**	Little excretion	Allowed
**Atenolol**	Increased excretion	Avoid
**Metoprolol**	Little excretion	Allowed
**Carvedilol**	Insufficient studies	Unclear
**Propranolol**	Little excretion	Allowed
**Bisoprolol**	Insufficient studies	Unclear (apparently safe)
**Hydralazine**	Little excretion	Allowed
**Methyldopa**	Little excretion	Allowed

## 11.8. Future Cardiovascular Risk

Hypertensive disorders of pregnancy are a marker of future risk (I: A), and a more careful and integrated approach should be adopted for these women in order to effectively prevent cardiovascular and kidney disease (LR: I, LE: C). Patients who develop any form of HT during pregnancy, especially with negative outcomes, such as preterm birth and early PE, experience a consistent increase in risk of future CVD and kidney disease. ^696^ ^-^ ^699^ The risk of chronic hypertension is 3 to 4 times higher, while risk of stroke is 1.8 times higher. Likewise, the risk of coronary artery disease (CAD) doubles with age. ^696^ ^,^ ^697^

In a prospective study, ^698^ gestational HT was associated with greater incidence of CAD (HR: 1.8; 95% CI: 1.3 to 2.6; p < 0.001), HF (HR: 1.7; 95% CI: 1.04 to 2.60; p = 0.03), aortic stenosis (HR: 2.9; 95% CI: 1.5 to 5.4; p < 0.001), and mitral insufficiency (HR: 5.0; 95% CI: 1.5 to 17.1; p = 0.01), showing a 30% global CV risk increase. Norwegian data show that PE is associated with a 3- to 15-fold increase in risk of stage 5 CKD. ^699^ Hypertensive disorders of pregnancy are a marker of future risk, and a more careful and integrated approach should be adopted for these women in order to effectively prevent CVD and kidney disease.

**Table:** 

Key Takeaways	
Classification	Pre-eclampsia, chronic hypertension, overlapping pre-eclampsia, and gestational hypertension.
**Prevention**	Calcium and acetylsalicylic acid for high-risk patients.
**Nonpharmacological treatment**	Should not be used for persistent SBP above 160 mm Hg for more than 15 minutes. Relative rest at hospital with monitoring for pre-eclampsia. Hospitalization of pregnant patients with severe HT.
**Expectant management**	Expectant management is suggested between the 34th and 37th gestational weeks for stable women, without clinical worsening or severe hypertension.
**Pharmacological treatment**	Urgent pharmacological treatment is indicated for severe hypertension and in the presence of warning signs. Pharmacological treatment should be initiated when BP is above 150-160/100-110 mm Hg, with the goal of keeping it in the 120-160/80-100 mm Hg range. The choice of the antihypertensive medication depends on the attending physician’s experience and familiarity with the drug chosen and its side effects. Magnesium sulfate is recommended for eclampsia prevention and treatment.

## 12. Hypertension in Children and Adolescents

## 12.1. Epidemiological Context and Importance of Hypertension in Pediatrics

The prevalence of high blood pressure (HBP) and hypertension (HT) in children and adolescents has increased in recent years. The current prevalence of HT in the pediatric age group ranges from 3% to 5%, while HBP is estimated at 10-15%. ^700^ ^,^ ^701^ In children ages 7 to 12, HBP and HT prevalence rates are 4.7% and 1.9% respectively, and both are more prevalent among the obese. ^702^

The Brazilian Study of Cardiovascular Risks in Adolescents (ERICA) evaluated 73399 Brazilian students ages 12 to 17. Total HBP prevalence in Brazil was 14.5%, and the highest rate was 29.3% for boys ages 15 to 17. The overall prevalence of HT was 9.6%, likewise highest among older children. The study found that 17.8% of prevalence rates for HT among adolescents is attributable to obesity. ^703^

Pediatric HT is usually asymptomatic, but as many as 40% of hypertensive children have left ventricular hypertrophy (LVH) at their initial diagnosis. Though oligosymptomatic in childhood, LVH is a precursor to arrhythmias and HF in adults. ^704^ Pediatric HT is also associated with the development of other changes to end-organs, such as increased carotid intima-media thickness, lower arterial distensibility, and retinal arteriolar narrowing. Blood pressure measurements from the age of 3 onwards are recommended, at least annually. ^705^

## 12.2. Definition and Etiology

In children and adolescents, the definitions of HBP and HT are related to the normal distribution curve for blood pressure (BP) and their percentile distributions. The measurement uses auscultation, taking into consideration sex, age, and height percentile of the child. ^164^ ^,^ ^706^ ^,^ ^707^

In 2017, normative BP scores and HT diagnosis and management recommendations for pediatric HT, excluding overweight and obese children and adolescents, were changed. ^5^ ^,^ ^705^ The term prehypertension has been replaced by HBP. The new recommendations, below, redefine HT staging for children and adolescents, simplify recommendations for preventive assessment in routine pediatric visits, structure the initial management of patients diagnosed with HBP or HT, and increase the importance of ABPM readings in diagnosis and management for pediatric HT.

Chart 12.1 presents up-to-date definitions of normal BP, high BP and stages 1 and 2 HT in children and adolescents by age, sex, and height percentile. ^705^ The younger the child and the higher the BP, the greater the chance of secondary HT. Parenchymal and obstructive nephropathies and renal artery stenosis are responsible for approximately 60 to 90% of cases and can affect all age ranges. Endocrine disorders, such as excessive mineralocorticoid, corticoid or catecholamine secretion, thyroid diseases, and hypercalcemia associated with hyperparathyroidism, account for approximately 5% of cases. Coarctation of the aorta is diagnosed in 2% of cases, while 5% of cases are attributed to other etiologies, such as adverse effects of vasoactive and immunosuppressant drugs, steroid abuse, central nervous system changes, and increased intracranial pressure. ^164^ ^,^ ^705^ ^-^ ^707^

**Chart 12.1:** – Updated definition of blood pressure according to age group.

Children 1 to 13 years old	Children ages ≥13 years old
Normal BP: < P90 for age, sex, and height	Normal BP: < 120 / < 80 mm Hg
High blood pressure: BP ≥ P90 and < 95th percentile for age, sex, and height or BP 120/80 mm Hg but < P95 (whichever is lowest)	High blood pressure: BP 120 / <80 mm Hg to PA129 / <80 mm Hg
Stage 1 hypertension: BP ≥ P95 for age, sex, and height up to < P95 + 12 mm Hg or BP between 130/80 and té 139/89mm Hg (whichever is lowest)	Stage 1 hypertension: BP 130/80 or up to 139/89 mm Hg
Stage 2 hypertension: BP ≥ P95 + 12 mm Hg or age, sex and height or BP ≥ 140/90 mm Hg (whichever is lowest)	Stage 2 hypertension: BP ≥ 140/90 mm Hg

*BP: blood pressure; P: percentile. Adapted from Flynn et al., 2017.^*705*^*

Primary HT seems to be the most common form of HT in adolescents It is most often associated with overweight, obesity, and family history of HT.

## 12.3. Diagnostic

### 12.3.1. BP Measurement Methods

Measuring BP in children is recommended at every clinical assessment. It should be measured annually in children and adolescents ≥ 3 years old, taking into consideration established measurement standards. For children under the age of 3, blood pressure measurements should be performed in certain situations. BP measurements should be repeated at every appointment under conditions, such as obesity, kidney disease, coarctation of the aorta, DM or chronic use of medications known to be associated with increased BP. Correctly measuring BP, following the standards established above, is a precondition for obtaining reliable readings and properly categorizing pediatric BP. ^176^ ^,^ ^705^ Preferably, it should be measured in the right arm, with the patient lying down until the age of three, and, in older children, with the patient sitting down, their arms lying at heart level, using an adequately sized cuff. The air bag should be 80 to 100% as long as the arm circumference (AC) and at least 40% as wide as the AC. Blood pressure assessments should follow the procedures described in Chapter 3. Use the auscultatory method to check for audible Korotkoff sounds to 0 mm Hg. The point where sounds become muffled is considered for DBP (Korotkoff phase IV). During the first appointment, BP should be measured on the four limbs, and when measured on lower limbs (LLs), the patient should be placed in ventral decubitus position, using an appropriately-sized cuff on their thigh and placing the stethoscope on their popliteal artery. SBP at the LLs is usually 10 to 20% higher than BP measured at the brachial artery. ^164^ Charts 12.2 and 12.3 list normal BP, high BP and stages 1 and 2 HT by sex, age, and height percentile, adapted from Flynn et al., 2017. ^705^ Some authors consider the oscillometric method adequate for initial screening in children and adolescents, which would justifying developing tables using validated devices. ^708^ ^,^ ^709^ In Brazil, Jardim et al. have developed a blood pressure reference curve for nonoverweight adolescents ages 12 to 17 using the oscillometric method. ^710^

**Chart 12.2:** – Blood pressure levels for boys by age and height percentile

**Chart 12.3:** – Blood pressure levels for girls by age and height percentile

Adapted from Flynn et al, 2017. ^705^ ^705^

The following clinical risk conditions determine the need for routine BP measurement for children 3 < years old: preterm birth, very low birth weight, intrauterine growth restriction, history of stay at neonatal intensive care unit (ICU) or umbilical catheterization after birth, congenital heart disease with or without surgical repair, recurring urinary tract infections, hematuria or proteinuria, nephropathy, solid organ transplantation, oncological disorders or bone marrow transplantation, chronic use of medications known to increase BP, system disorders associated with HT (neurofibromatosis, tuberous sclerosis, sickle cell anemia, among others), and evidence of intracranial hypertension. ^705^

The oscillometric method is recommended for measuring BP in newborns (NB). Normative BP values for the neonates 15 days old and older and gestational age after birth of 26 to 44 weeks can be found in Chart 12.4 . ^711^ Oscillometric devices, duly validated for pediatric age groups, may be used for initial BP assessments. If elevated BP is suspected from oscillometric readings, auscultation should be used to verify the finding. Pediatric HT diagnoses are based on confirming BP values ≥ 95th percentile in three different visits using auscultation. ^705^ Chart 12.5 provides a simplified list of BP values suggesting the need for additional clinical assessments. ^705^ ^,^ ^711^

**Chart 12.4:** – Estimated blood pressure levels at two weeks for neonates 26 to 44 weeks since conception.

Age since conception	50th Percentile	95th Percentile	99th Percentile
**44 weeks**			
**SBP**	88	105	110
**DBP**	50	68	73
**MAP**	63	80	85
**42 weeks**			
**SBP**	85	98	102
**DBP**	50	65	70
**MAP**	62	76	81
**40 weeks**			
**SBP**	80	95	100
**DBP**	50	65	70
**MAP**	60	75	80
**38 weeks**			
**SBP**	77	92	97
**DBP**	50	65	70
**MAP**	59	74	79
**36 weeks**			
**SBP**	72	87	92
**DBP**	50	65	70
**MAP**	57	72	71
**34 weeks**			
**SBP**	70	85	90
**DBP**	40	55	60
**MAP**	50	65	70
**32 weeks**			
**SBP**	68	83	88
**DBP**	40	55	60
**MAP**	50	65	70
**30 weeks**			
**SBP**	65	80	85
**DBP**	40	55	60
**MAP**	48	65	68
**28 weeks**			
**SBP**	60	75	80
**DBP**	38	50	54
**MAP**	45	58	63
**26 weeks**			
**SBP**	55	72	77
**DBP**	30	50	56
**MAP**	38	57	63

*DBP: diastolic blood pressure; MAP: mean arterial pressure; SBP: systolic blood pressure. Adapted from Dionne et al., 2012.^*711*^*

**Chart 12.5:** – BP values considered warning signs for additional clinical assessment by chronological age

Age	Male			Female	
	SBP	DBP	SBP		DBP
1	98	52	98		54
2	100	55	101		58
3	101	58	102		60
4	102	60	103		62
5	103	63	104		64
6	105	66	105		67
7	106	68	106		68
8	107	69	107		69
9	107	70	108		71
10	108	72	109		72
11	110	74	111		74
12	113	75	114		75
≥13	120	80	120		80

*DBP: diastolic blood pressure; SBP: systolic blood pressure. Adapted from Flynn et al., 2017.^*705*^*

## 12.4. History-Taking

Detailed data on birth, growth and development, personal antecedents of kidney, urological, endocrine, heart and neurological diseases, and lifestyle should be collected, in addition to data on the use of medications and other substances that may cause BP alterations. In addition, family antecedents for HT, kidney disease, and other CVRFs should be carefully assessed. Children ≥ 6 years old do not require extensive screening for secondary causes of HT in the presence of positive family history of HT, overweight, or obesity, and/or their history or physical examination do not suggest secondary causes. ^705^ ^,^ ^712^

## 12.5. Physical Examination

On physical examination, the patient's body mass index (BMI) should be calculated ^713^ and the physician should investigate signs of secondary HT (see Chapter 15). ^714^

## 12.6. Additional tests

Laboratory and imaging tests are aimed at defining the etiology of HT (primary or secondary) and detecting end-organ damage (EOD) and cardiovascular risk factors (CVRFs) associated with HT (Charts 12.6 and 12.7 ). ^705^ ^,^ ^715^ End-organ assessments should be performed in all children and adolescents with stages 1 and 2 HT. Sleep study by use of polysomnography is indicated for children and adolescents with sleep disorders detected while taking their histories.705

**Chart 12.6:** – Initial investigation in children and adolescents with HT.

Complete blood count
Kidney function and electrolytes (including calcium, phosphorus and magnesium)
Lipid profile
Serum uric acid
Fasting glycemia
Urinalysis and urine culture
Fundoscopy
Chest X-ray
Doppler echocardiography
Kidney and urinary tract ultrasound and Doppler ultrasound of renal arteries

*US: ultrasound.*

## 12.7. Ambulatory Blood Pressure Monitoring (ABPM)

ABPM should be used to confirm HT in children and adolescents with office BP readings compatible with high BP for at least a year or with BP readings corresponding to stage 1 HT in three outpatient visits. ^705^ It should also be considered in routine examinations for secondary HT, CKD, diabetes mellitus (DM), obstructive sleep apnea (OSA), obesity, post-operative coarctation of the aorta, preterm birth, solid organ transplantation, and RfHT. The procedure should follow standard techniques and use monitors validated for pediatric use as well as pediatric reference data. ^716^

The 6^th^ABPM guidelines and 4^th^home blood pressure monitoring (HBPM) guidelines provide the information required to analyze ABPM data from children and adolescents. ^186^ BP categorization using ABPM data takes into account, in addition to BP readings, blood pressure load parameters, and BP dipping during nighttime sleep, as shown in Chart 12.7 . ^713^

**Chart 12.7:** – Suggested ambulatory BP staging in children and adolescents

Classification	Office BP	Ambulatory SBP/DBP	Systolic/diastolic load
Normal BP	< P90	< P95	<25%
White-coat hypertension	≥ P95	< P95	<25%
High BP	≥ P90 or > 120/80 mm Hg	< P 95	≥ 25%
Masked hypertension	< P95	< P95	≥ 25%
Ambulatory hypertension	> P95	> P95 (> P90 secondary HT)	25 to 50%
Severe ambulatory hypertension	>P95	> P95	> 50%

*BP: blood pressure; P: percentile. Adapted from Flynn et al., 2017.^*705*^*

## 12.8. Therapeutic Aspects

The primary objectives of treating HT during childhood and adolescence is preventing EOD and continued HT in adulthood. The plan depends on the etiology of HT, cardiovascular (CV) risk associated with other underlying diseases, and the presence of EOD (LE: C). ^705^

## 12.9. Nonpharmacological Therapy

Nonpharmacological therapy should be introduced to all pediatric patients with BP levels above the 90th percentile or BP < 130/80 (≥ 13 years old) (LE: C). ^705^ This includes weight loss, physical exercise, dietary intervention, and stress control. The combination of these four measures leverages their impact compared to the individual effect of each intervention. ^705^

Weight loss provides good results, and the use of a motivational approach seems to be the most effective method for controlling the association between obesity and HT in childhood (LE: C). ^717^ All children and adolescents should perform at least 300 minutes of moderate to vigorous physical activity per week for their health. In addition, sedentary behavior (time spent sitting or lying down) should be restricted for this age group. Structured physical exercise has greater impact on SBP values. ^717^ It is recommended that they perform moderate-intensity aerobic exercises (30-60 minutes) at least three times a week, and daily if possible. ^705^ Resistance training can be added to this regimen. Competitive sports are not recommended for patients with uncontrolled stage 2 HT (LE:C). ^718^

Dietary interventions should include restricted sodium intake and may include potassium and calcium supplementation. Observational studies have shown the positive effects of the polyphenols found in olive oil. ^705^ ^,^ ^719^ This guideline recommends the DASH diet, which emphasizes plant-based foods and decreased intake of sugar and sweets. This measure is especially effective for HT associated with obesity (LE: B). ^719^ ^-^ ^721^ Stress control is also recommended for this age group, and may be achieved with various forms of meditation, mindfulness, and yoga (LE: C). ^705^

## 12.10. Pharmacological Therapy

Pharmacological therapy should be initiated for children with symptomatic HT, secondary to CKD or DM, presence of EOD, stage 2 HT with no apparent modifiable cause, and persistent HT nonresponsive to lifestyle changes (LSCs) (LE: B). ^705^ The treatment target is to lower BP below the 90th percentile (LE: C). Treatment should begin with an antihypertensive agent at its lowest dose, increased every two to four weeks until the target level is achieved. If this regimen is not sufficiently effective, other medication classes are added in sequence. Since many medication classes increase sodium and fluid retention, considering thiazide diuretics is recommended as the second medication for combination therapies. Overall, adverse events associated with the use of antihypertensive agents in children and adolescents have been mild (LE: B). ^705^ ^,^ ^722^

All classes of antihypertensive drugs seem safe, at least in the short run. ^722^ However, recent international guidelines recommend preferential use of angiotensin-converting enzyme inhibitors (ACEIs), ARBs, long-acting CCBs, or thiazide DIUs as first-line medications. If a third antihypertensive is needed, the recommended medications are alpha-blockers, BBs, centrally acting sympatholytics, or potassium-sparing diuretics (LE: C). ^705^ ^,^ ^722^

In secondary HT, the choice of antihypertensive should follow the physiopathological principle involved, taking into account the comorbidities present in each case. ^719^ ^-^ ^726^ Patients with resistant HT require stronger decreases in sodium intake, as well as detailed investigation of their consumption of substances or foods causing HT, adherence to therapy regimen, and maximum optimization of said regimen (LE: C). ^705^

If the patient does not respond to monotherapy for longer than six months, their referral to a specialist in HT in children and adolescents should be considered (LE: C). ^727^ Chart 12.8 provides a list of medications used in pediatric settings and their dosage. ^705^ ^,^ ^725^ ^,^ ^726^

**Chart 12.8:** – Antihypertensive medications prescribed to children and adolescents in Brazil

Medication	Age	Initial dose	Maximum dose	Interval
Clonidine	> 12 to	0.2 mg/day	2.4 mg/day	12 h
Atenolol		0.5-1 mg/kg/dose	2 mg/kg/day (max. 100 mg/day)	12-24 h
Propranolol		1-2 mg/kg/dose	4 mg/kg/day (max. 640 mg/day)	8-12 h
Amlodipine	1-5 years	0.1 mg/kg/dose	0.6 mg/kg/day (max. 5 mg/day)	24 h
	> 6 years	2.5 mg/day	10 mg/day	24 h
Isradipine	Toddler	0.05-0.1 mg/kg/dose	0.6 mg/kg/day (max. 10 mg/day)	8-12 h
Felodipine	> 6 years	2.5 mg/day	10 mg/day	24 h
Nifedipine XL		0.25-0.5 mg/kg/dose	3 mg/kg/day (max. 120 mg/day)	12-24 h
Candesartan	1-5 years	0.02 mg/kg/dose (max. 4 mg/day)	0.4 mg/kg/day (max. 16 mg/day)	12-24 h
				
Olmesartan	> 6 years	< 35 kg: 10 mg/day	< 35 kg: 20 mg/day	24 h
		> 35 kg: 20 mg/day	> 35 kg: 40 mg/day	24 h
Losartan	> 6 years	0.7 mg/kg/day (max. 50 mg/day)	1.4 mg/kg/day (max. 100 mg/day)	24 h
Valsartan	> 6 years	1.3 mg/kg/day	2.7 mg/kg/day (max. 160 mg/day)	24 h
Prazosine	> 12 years	0.05-0.1 mg/kg/dose	0.5 mg/kg/day	8 h
Furosemide		0.5-2 mg/kg/dose	6 mg/kg/day	4-12 h
Spironolactone		1 mg/kg/dose	3.3 mg/kg/day (100 mg/day)	6-12 h
Chlorthalidone	> 40 kg	0.3 (max. 50 mg/day)	2 mg/kg/day	24 h
Hydrochlorothiazide		1 mg/kg/dose	2 mg/kg/day (max. 37.5 mg/day)	
Benazepril	> 6 years	0.2 (max. 10 mg/day)	0.6 mg/kg/day (max. 40 mg/day)	24 h
Captopril	Infant	0.05 mg/kg/dose	6 mg/kg/day	6-24 h
	Toddler	0.5 mg/kg/dose	6 mg/kg/day	8 h
Enalapril	> 1 month	0.08 mg/kg/dose	0.6 mg/kg/day (max. 40 mg/day)	12-24 h
Fosinopril	> 6 years	0.2 mg/kg/dose (max. 10 mg/day)	0.6 mg/kg/day (max. 40 mg/day)	24 h
Lisinopril	> 6 years	0.07 mg/kg/dose (max. 5 mg/day)	0.6 mg/kg/day (max. 40 mg/day)	24 h
Ramipril		1.6 mg/m2/day	6 mg/m2/day	24 h
Hydralazine		0.75 mg/kg/dose	7.5 mg/kg/day (max. 200 mg/day)	6 h
Minoxidil	< 12 years	0.2 mg/kg/dose	50 mg/day	6-8 h
	> 12 years	5 mg/day	100 mg/day	

*h: hours; Max.: maximum.*

## 12.11. Follow-up of Children and Adolescents with HT

Frequency of follow-up in children and adolescents with HT depends on severity and need for treatment. Patients undergoing nonpharmacalogical therapy only should have clinical follow-up visits every 3 to 6 months, with HBPM as an adjuvant for blood pressure control.

For patients requiring medications, soon after treatment onset, follow-up visits should be scheduled every 15 to 30 days after establishing the optimum dose or need for combination. In an intermediate stage, visits should be scheduled every 4 to 6 weeks, and quarterly once HT is controlled.

Follow-up appointments should included a detailed analysis of adherence and side effects. Requesting laboratory tests depends on the medications used and severity of HT and underlying diseases; likewise, how frequently the patient is tested for EOD depends on the underlying disease and on the severity of HT. Requesting ABPM is indicated when there is no HT control or when there is risk of masked hypertension (MH), such as the late post-operative period after surgical correction for coarctation of the aorta (LE: C). ^705^

## 12.12. Hypertensive Crisis

Hypertensive emergencies (HEs) and hypertensive urgencies (HUs) are defined in Chapter 13. ^727^ There is no consensus BP level that defines HE, ^728^ though some authors suggest a cutoff point 20% above stage 2 HT (> 99th percentile). ^729^ The American Academy of Pediatrics (AAP), in turn, defines HE as any condition in which a child has BP above stage 2 HT. However, the AAP warns that children with BP > 95th percentile + 30 mm Hg run a higher risk of complications. In general, HEs are secondary to underlying diseases still requiring investigation, ^705^ and most often should be treated with intravenous (IV) medication administered to hospitalized patients, often in the ICU. Patients with HUs and no signs of end-organ impairment may initially receive central alpha-agonists, vasodilators or CCBs. ^705^ The objective of treatment is to lower BP by 25% during the first 8 hours, followed by a slow decrease over 24 to 48 hours, until reaching the 95th percentile, since accelerated decreases may cause damage, especially to the brain. ^730^ ^,^ ^731^ Chart 12.9 shows the most frequently used medications in pediatric HEs (LE: C).13. Crise Hipertensiva

**Table:** 

Key Takeaways
All children and adolescents ≥ 3 years old should have their BP measured annually.
Children 3 < years old should undergo BP measurements in case of preterm birth, very low birth weight, intrauterine growth restriction, history of stay at neonatal ICU, congenital heart disease, nephropathy, solid organ transplantation, oncological disorders, chronic use of medications known to increase BP, system disorders associated with HT, and evidence of intracranial hypertension.
All children and adolescents ≥ 3 years old should have their BP measured at every medical visit in case of overweight, chronic use of medications known to increase BP, kidney disease, coarctation of the aorta, and diabetes.
Children and adolescents should be diagnosed with HT when BP measured by auscultation in three separate visits is above the 95th percentile for their age, sex, and height percentile.
In children and adolescents diagnosed with HT, the pharmacological and nonpharmacological treatment goals should be to lower BP to below the 90th percentile for age, sex, and height percentile and to < 130/80 mm Hg in adolescents ≥ 13 years old.

**Chart 12.9:** – Major pediatric medications and doses used to control hypertensive emergencies

Medication	Route	Dose	Beginning of action	Duration
Sodium nitroprusside	IV	0.5-10 µg/kg/min	Seconds	Only during infusion
Labetalol	IV	0.25-3 mg/kg/h or bolus 0.2-1 mg/kg followed by infusion of 0.25-3 mg/kg/h	2-5 min	2-4 h
Nicardipine	IV	1-3 µg/kg/min	2-5 min	30 min-4 h, the greater, the longer the use
Hydralazine	IV IM	Bolus 0.2-0.6 mg/kg IV or IM, max. = 20mg	10-30 min	4-12 h
Esmolol	IV	Attack 100-500 µg/kg followed by infusion 50-300 µg/kg/min	Seconds	10-30 min
Phentolamine	IV	Bolus 0.05-0.1 mg/kg max. = 5 mg/dose	Seconds	15-20 min

*h: hour; IM: intramuscular; IV: intravenous; min: minute. Adapted from Flynn et al., 2017.^*705*^*

## 13. Hypertensive Crisis

## 13.1. Definition

The terms hypertensive urgency and hypertensive emergency were proposed as an operational classification of hypertensive crises (HCs) in 1993 by the V Joint National Committee on Detection Evaluation and Treatment of High Blood Pressure. ^732^

Hypertensive urgencies (HUs) are symptomatic clinical situations in which there is significant blood pressure (BP) elevation (arbitrarily defined as systolic BP (SBP) DBP ≥ 180 mm Hg and/or diastolic BP (DBP) ≥ 120 mm Hg) without acute and progressive end-organ damage (EOD) and no imminent risk of death. ^5^ ^,^ ^164^ ^,^ ^733^ ^,^ ^734^

Hypertensive emergencies (HEs), in turn, are symptomatic clinical situations in which there is significant BP elevation (arbitrarily defined as SBP ≥ 180 mm Hg and/or DBP ≥ 120 mm Hg) with acute and progressive EOD and imminent risk of death. ^5^ ^,^ ^164^ ^,^ ^733^ ^,^ ^734^

A common condition at emergency rooms is the hypertensive pseudocrisis (HTPC). In HTPCs, there is no acute EOD or immediate risk of death. In general, it is seen in uncontrolled hypertensive patients undergoing treatment, or in untreated hypertensive patients, with very high BP measurements, but who are either olygosymptomatic or asymptomatic. High BP after an emotional, painful or uncomfortable event, such as migraines, dizziness, vascular and musculoskeletal headaches and panic attacks also characterize HTPC. ^733^ ^,^ ^734^

## 13.2. Classification

HE is not defined by BP level, though it is often very high, but predominantly by the patient's clinical status. It can manifest as a cardiovascular, cerebrovascular, renal, or multi-organ event, or even as pre-eclampsia with severe features or eclampsia. Chart 13.1 shows the classification of HEs. Chart 13.2 differentiates HUs from HEs in terms of diagnosis, prognosis, and management.

**Chart 13.1:** – Classification of hypertensive emergencies

HYPERTENSIVE EMERGENCIES
**Cerebrovascular** • Hypertensive encephalopathy • Ischemic stroke • Hemorrhagic stroke • Subarachnoid hemorrhage **Cardiocirculatory** • Acute aortic dissection • Acute pulmonary edema with left ventricular failure • Acute coronary syndromes **Kidney/multiple organ failure** • Accelerated/malignant hypertension • MOD hypertension
• Severe adrenergic crises • Pheochromocytoma crisis • Drug overdose (cocaine, crack cocaine, LSD)
• Gestational hypertension • Eclampsia • Pre-eclampsia with severe features • HELLP syndrome • Severe hypertension at the end of pregnancy

*MOD: multi-organ damage. HELPP: hemolysis, elevated liver enzymes, low platelets. Adapted from Malachias et al., 2016;^*164*^Bortolotto et al., 2018;^*733*^Martion & Ribeiro, 2015;^*734*^Whelton et al., 2018^*5*^; Cremesp, 2004;^*746*^Williams et al., 2018;^*37*^Ma et al., 2020.^*778*^*

**Chart 13.2:** – Diagnosis, prognosis, and management of hypertensive urgencies and emergencies

Urgency	Emergency
Markedly high BP level	Markedly high BP level
Without acute and progressive EOD	With acute and progressive EOD
Oral drug combination	Parenteral medication
No imminent risk of death	Imminent risk of death
Early outpatient follow-up care (7 days)	Preferential ICU admission

*EOD: end-organ damage; ICU: intensive care unit.*

## 13.3. Major Epidemiological, Pathophysiological, and Prognostic Aspects

### 13.3.1. Epidemiology

Hypertensive crises account for 0.45 to 0.59% of all hospital emergency treatments, while HEs account for 25% of all cases of HC. Ischemic stroke and acute pulmonary edema (APE) are the most common conditions found in HEs ^735^ ^-^ ^737^ , with decreasing incidence in recent decades. ^738^ ^-^ ^740^

### 13.3.2. Pathophysiology

Since systemic BP is the product of cardiac output (CO) by peripheral vascular resistance (PVR), acute increases in BP may be the result of changes to those variables. Therefore, increased intravascular volume and PVR, reduced production of endogenous vasodilators, and/or activation of vasoconstrictor systems may precipitate greater vascular reactivity, resulting in HC. ^741^ ^,^ ^742^ Tissue autoregulation is compromised, particularly in the cerebral and renal vascular beds, resulting in local ischemia, which triggers a vicious circle of vasoconstriction, endothelial damage and activation of the platelet, coagulation and immune system, with myointimal proliferation, arteriole fibrinoid necrosis, and end-organ ischemia. ^741^ ^-^ ^743^ The autoregulation curve shifts to the right in chronic hypertensive patients, making both actual BP level and rate of increase important to the genesis of HE. On the other hand, that shift in the autoregulation curve predisposes patients to tissue ischemia in aggressive BP reductions in HE treatment. ^742^ ^,^ ^743^

### 13.3.3. Prognosis

The one-year mortality rate for untreated HE is approximately 80%, ^739^ and effective antihypertensive treatment is associated with significant improvements in prognosis. ^740^ Five-year survival rates are higher for individuals with HU than those with HE. ^735^ ^,^ ^744^

## 13.4. Complementary Clinical and Laboratory Investigation 164,733,734

Taking a clinical history directed to the potential cause of the disease is critical. Clinical and laboratory investigation and requesting tests should provide for the proper assessment of BP and the presence of acute EOD. Initially, BP should be measured in both arms, preferably in a calm environment, and repeatedly until stabilization (minimum of three measurements). Data on the patient’s typical BP should be rapidly collected, as well as information on situations that can trigger BP increases and comorbidities; the use or discontinuation of antihypertensive medications (particularly adrenergic antagonists); or the use of substances that can increase BP (see Chapter 15). A systematic approach, including an assessment of signs and symptoms, physical examination and complementary investigation, helps determine the presence of acute and progressive EOD, as shown in Chart 13.3 :

**Chart 13.3:** – Clinical and complementary investigation by end-organ damage of hypertensive emergencies

Primary damage in HE	Symptoms	Physical examination	Complementary investigation at physician's discretion
**Cardiovascular**	- Chest, abdominal, or back pain or discomfort; - Dyspnea; fatigue; coughing.	- HR, heart rhythm, pulse changes, gallop rhythm, jugular venous distension, and pulmonary, abdominal, and peripheral congestion; - Heart and vascular murmurs; - Four-limb blood pressure palpation.	- ECG, O_2_saturation, chest X-ray, myocardial necrosis markers, BNP, lactate dehydrogenase; - Echocardiogram; - Angiotomography, chest CT, and chest MNR.
**Neurological**	- Dizziness; headaches; - Impaired sight, hearing or speech;	- Consciousness or coma level; agitation, delirium or confusion; seizures; focal deficits; neck stiffness.	- Head CT; head MNR
**Renal**	- Change in urination frequency and volume;	- Edema or dehydration; - Change in urine aspect (hematuria); - Abdominal masses and murmurs.	- Urine I; creatinine; urea; Na^+^; K^+^; chlorine; blood gas analysis.
**Fundus**		- Papilledema; hemorrhages; exudates; - Vascular changes, such as spasms, pathological arteriovenous crossings, arterial wall thickening, and silver - or copper-wire aspect.	
**Minimum additional tests**	- ECG, chest X-ray, myocardial necrosis markers, CBC with platelet count, creatinine, urine I, and potassium.		

*BNP: atrial natriuretic peptide; CT: computed tomography; ECG: electrocardiogram; HE: hypertensive emergency; HR: heart rate; MNR: magnetic nuclear resonance. Adapted from Malachias et al., 2016;^*164*^Bortolotto et al., 2018;^*733*^Martion & Ribeiro, 2015;^*734*^Whelton et al., 2018;^*5*^Vilela-Martin et al., 2020.^*747*^*

## 13.5. General Treatment of Hypertensive Crisis

Treatment of HU ( Figure 13.1 ) should begin after a period of clinical observation in a calm environment, which helps rule out cases of pseudocrisis (treated only with rest or the use of painkillers or tranquilizers). Captopril and clonidine are indicated for acute treatment. Captopril, at a dose of 25-50mg, has peak action within 60 to 90 minutes, while clonidine is fast-acting, working in approximately 30 to 60 minutes, at a dose of 0.100 to 0.200mg. The use of immediate-release nifedipine capsules to treat HU should be banned, because it is neither safe nor effective, and causes rapid and marked BP reductions, which can result in tissue ischemia. ^745^ ^,^ ^746^

**Figure 13.1:** – Patient care flow chart for hypertensive crisis.

Adapted from Whelton et al., 2018. ^5^

There is no evidence from randomized controlled trials showing antihypertensives reduce morbidity and mortality for individuals with HE. However, based on clinical experience and the progress of patients under treatment, antihypertensive treatment is beneficial and cuts mortality. The treatment of patients with HE is aimed at rapid BP reduction to prevent the progression of EOD. Individuals should be preferentially admitted to the ICU, treated with intravenous (IV) antihypertensives, and carefully monitored during treatment to prevent hypotension. The general recommendations for BP reduction for HE are (LR: I; LE: C): ^5^ ^,^ ^164^

• Mean BP ≤ 25% in the 1st hour;
• BP 160/100-110 mm Hg in 2 to 6 h;
• BP 135/85 mm Hg in 24-48 hours.

However, HEs should be approached considering the impaired system or end-organ.

13.6. Hypertensive Emergencies in Special Situations

Chart 13.4 shows the medications indicated for the main forms of HE.

**Chart 13.4:** – Parenteral medications used to treat hypertensive emergencies

Drugs	Route of administration and dosage	Beginning	Duration	Indications	Adverse events and precautions
Sodium nitroprusside (arterial and venous vasodilator, stimulates cGMP formation)	Continuous infusion 0.25-10 mg/kg/min IV	Immediate	1-2 min	Most hypertensive emergencies	Cyanide poisoning, severe hypotension, nausea, vomiting. Attention in kidney and liver failure and high intracranial pressure. Protect from light.
Nitroglycerin (arterial and venous vasodilator)	Continuous IV infusion 5-15mg/h	2-5 min	3-5 min	Coronary failure, left ventricular failure with APE	Headache, reflex tachycardia, tachyphylaxis, flushing, metahemoglobinemia
Metoprolol (selective beta-adrenergic blocker)	5 mg IV (repeat every 10 min, if necessary up to 20 mg)	5-10 min	3-4 h	Coronary failure Acute aortic dissection (in combination with SNP)	Bradycardia, advanced AVB, heart failure, bronchospasm
Esmolol (ultra-rapid selective beta-adrenergic blocker)	Attack: 500μg/kg Intermittent IV infusion 25-50 μg/kg/min ↑ 25 μg/kg/min every 10-20 min. Maximum 300 μg/kg/min	1-2 min	1-20 min	Acute aortic dissection (in combination with SNP) Severe postoperative hypertension	Nausea, vomiting, 1st-degree atrioventricular block, bronchospasm, hypotension
* Phentolamine (alpha-adrenergic blocker)	Continuous IV infusion: 1-5mg. Maximum 15mg	1-2 min	3-5 min	Excess of catecholamines	Reflex tachycardia, flushing, dizziness, nausea, vomiting
* Trimethaphan (SNS and PSNS ganglionic blocker)	Continuous IV infusion: 0.5-1.0 mg/min. ↑ 0.5mg/min up to maximum of 15mg/min	1-5 min	10 min	Excess of catecholamines Acute aortic dissection	Tachyphylaxis
Hydralazine (direct-acting vasodilator)	10-20 mg IV or 10-40 mg IM every 6 h	10-30 min	3-12 h	Eclampsia	Tachycardia, headache, vomiting. Worsening of angina and infarction. Attention to high intracranial pressure
Diazoxide (vasodilator of arteriolar smooth muscle)	IV infusion 10-15 min 1-3 mg/kg Maximum 150 mg	1-10 min	3-18 h	Hypertensive encephalopathy	Retention of sodium, water, hyperglycemia, and hyperuricemia
* Fenoldopam (selective dopaminergic agonist)	Continuous IV infusion 0.1-1.6 μg/kg/min	5-10 min	10-15 min	Acute renal failure	Headache, nausea, flushing
* Nicardipine (calcium channel blocker)	Continuous IV infusion 5-15mg/h	5-10 min	1-4 h	Stroke Hypertensive encephalopathy Left ventricular failure with APE	Reflex tachycardia, phlebitis, avoid in patients with heart failure or myocardial ischemia
* Labetalol (alpha- and beta-adrenergic blocker)	Attack: 20-80 mg IV every 10 min Continuous IV infusion 2mg/min (maximum 300 mg/24 h)	5-10 min.	2-6 h	Stroke Acute aortic dissection (in combination with SNP)	Nausea, vomiting, atrioventricular block, bronchospasm, orthostatic hypotension
* Enalapril (ACE inhibitor)	Intermittent IV infusion 5.0 mg every 6 h up to 20 mg	15 min.	4-6 h	Left ventricular failure with APE	Hypotension, kidney failure, gestation
Furosemide (loop diuretic)	20-60 mg IV (repeat after 30 min)	2-5 min	30-90 min.	Left ventricular failure with APE Hypervolemic conditions, such as CKD, ADGN	Hypopotassemia

* Not available in Brazil. ACEI = angiotensin-converting enzyme inhibitors; ADGN = acute diffuse glomerulonephritis; APE = acute pulmonary edema; AVB = atrioventricular block; CKD = chronic kidney disease; IV = intravenous; PNS = parasympathetic nervous system; SNP = sodium nitroprusside; SNS = sympathetic nervous system. Adapted from Malachias et al., 2016;^*164*^Bortolotto et al., 2018;^*733*^Martion & Ribeiro, 2015;^*734*^Whelton et al., 2018;^*5*^Vilela-Martin et al., 2020.^*747*^

### 13.6.1. Hypertensive Encephalopathy 747,748

Hypertensive encephalopathy is a neurological HE characterized by signs and/or symptoms of cerebral edema secondary to sudden and/or sustained BP elevation. In general, it is found in chronic hypertensive patients who develop accelerated/malignant hypertension or in previously normotensive individuals with sudden increases in BP progressing to the failure of cerebral perfusion autoregulation mechanisms. It is also characterized by insidious onset and progresses with headaches, nausea, or vomiting. There may be changes to the visual field, photopsia, blurred vision, visual hallucinations, confusion, coma, generalized convulsive crises, and hyperreflexia. Treatment consists of slowly lowering BP, since rapid and intense decreases may cause cerebral hypoperfusion and loss of cerebral autoregulation mechanism. Sodium nitroprusside (SNP) is recommended in Brazil. In other countries, the following medications are available: nicardipine, clevidipine, labetalol, and fenoldopam. In the first 24 to 48 h, oral antihypertensives should be administered to better control BP.

## 13.7. Stroke

Hypertension is the primary risk factor for strokes, especially hemorrhagic strokes. ^749^ Diagnosis is based on a full neurological examination; for severity assessment purposes, use the National Institute of Health Stroke Scale (NIHSS). Head CTs and MNRs enable physicians to define the type of stroke (ischemic stroke in 85% of cases, hemorrhagic stroke in 15% of cases) and the area involved. ^164^ ^,^ ^750^ For incipient infarctions, an MNR is more sensitive than a CT.

### 13.7.1. Ischemic Stroke

BP often decreases spontaneously within 90 to 120 minutes during the acute phase. The recommendations are as follows:

1. In case of ischemic stroke with indication for thombolysis, BP reduction < 185/110 mm Hg before fibrinolytic therapy is recommended (LR: I; LE: B). ^5^ ^,^ ^652^ If BP remains > 185/110 mm Hg, thrombolytic therapy should not be administered. That recommendation also applies to individuals who are to undergo thrombectomy. ^751^ BP should be maintained < 180/105 mm Hg in the first 24 hours after thrombolysis.
2. An initial 15% decrease in BP can be applied in cases of very high BP (≥ 220/120 mm Hg) and other associated HEs (aortic dissection, acute coronary events, eclampsia, post-thrombolysis, and/or APE) (LR: I; LE: C). ^5^ ^,^ ^652^
3. In patients with BP ≥ 220/120 mm Hg that have not received thrombolytics and do not present with other HE requiring antihypertensive treatment, the benefit of starting or restarting treatment for hypertension in the first 48 to 72 h is unclear. It seems prudent to reduce BP by 15% during the first 24 h after the beginning of the ischemic stroke (LR: IIb; LE: C). ^5^ ^,^ ^652^
4. Starting or restarting antihypertensive therapy during hospitalization for neurologically stable patients with BP ≥ 140/90 mm Hg is safe for improving long-term BP control (LE: B; LR: IIa). ^5^ ^,^ ^652^
5. In other cases of ischemic stroke, reducing BP within 5 to 7 days of the event has controversial neurological effects requiring treatments be tailored for individual patients (LR: I; LE: A). ^652^

### 13.7.2. Hemorrhagic Stroke

Elevated BP increases the risk of hematoma expansion and death, in addition to worsening the prognosis for neurological recovery. However, there is no conclusive evidence in favor of rapid reductions of BP. Cerebral edema occurs in 30% of cases, usually during the first 24 hours. In those cases, decompressive craniectomy should be performed and patients transferred to specialized centers (LR: I; LE: B). ^5^ ^,^ ^752^

For individuals with acute presentation (< 6 h from onset of hemorrhagic stroke):

1. SBP > 220 mm Hg – consider BP reduction with continuous IV infusion and frequent BP monitoring (LR: IIa; LE: C). ^5^ ^,^ ^752^
2. SBP from 150 to 220 mm Hg – lowering BP below 140 mm Hg provides no benefits in terms of lower mortality or severe impairment and is potentially dangerous (LR: III; LE: A). ^5^ ^,^ ^752^ Consider a target SBP < 180 mm Hg. ^37^

### 13.7.3. Acute Coronary Syndromes

Coronary syndromes can be accompanied by BP elevation due to a reflex triggered by the ischemic myocardium. Consequently, higher PVR increases myocardial oxygen demand. The goal is to reduce the afterload without increasing the heart rate or reducing preload too much, since it would lead to increased myocardial oxygen consumption. The goal should be SBP < 140 mm Hg (avoid < 120 mm Hg) and DBP between 70 and 80 mm Hg using esmolol, metoprolol, or nitroglycerin (LR: I; LE: A). Intravenous nitrates reduce PVR, improve coronary perfusion, and have an important systemic vasodilator effect, reducing preload and myocardial oxygen consumption. Hydralazine, SNP, and nifedipine use is contraindicated, since it may promote flow steal. ^164^ ^,^ ^733^ The recommendations are:

- a) Intravenous nitroglycerin (NTG) is indicated in the first 48 hours for treatment of hypertension, persistent ischemia3 and HF, as long as hypotension, right ventricular infarction, or use of phosphodiesterase type 5 inhibitors have not been present in the previous 48 h (LR: I; LE: B). NTG use should not exclude other interventions that have proven to reduce mortality, such as beta-blockers (BBs) or ACEIs. ^753^ ^,^ ^754^
- b) The use of IV BBs is indicated for hypertensive individuals who do not present with: 1) signs of HF; 2) clinical evidence of low CO; 3) increased risk for cardiogenic shock; or 4) other contraindications for beta blockade (LR: IIa, LE: B). ^753^ ^,^ ^756^

### 13.7.4. Acute Pulmonary Edema (APE)

Approximately one third of the patients admitted with APE and HE have preserved left ventricular function, and myocardial ischemia may also be involved in the pathophysiology of APE associated with HE. ^755^ ^,^ ^756^ HE with APE should be controlled primarily in an ICU setting, with IV medication, monitoring, and gradual BP reduction. NTG and SNP are used to lower preload and afterload. Loop diuretic use also lower volume overload and, consequently, BP. In some cases, the use of noninvasive continuous positive airway pressure may be indicated for decreasing pulmonary edema and venous return.747,748,757

#### 13.7.4.1. Acute Aortic Dissection

In patients with precordial pain and high BP, acute aortic dissection should always be considered. The progression of dissection is related to BP level and ventricular ejection velocity. ^758^ Achieving proper pain management (IV opiates for analgesia), HR < 60 bpm, and SBP between 100 and 120mm Hg are important (LR: I; LE: B). ^5^ ^,^ ^747^ ^,^ ^758^ SBP < 120 mm Hg should be achieved in 20 minutes. The use of SNP in isolation is not ideal, since it increases HR and aortic ejection velocity, potentially worsening the dissection. ^5^ ^,^ ^747^ ^,^ ^758^ Thus, SNP should be associated with a BBs, initially IV, short, and titrable (metoprolol, labetalol, or esmolol), to decrease the heart rate. Alternately for asthma patients, nondihydropyridine calcium channel blockers (CCBs) may be used.

## 13.7.5. Pre-eclampsia/Eclampsia (see Chapter 11)

## 13.7.6. HE from Illicit Drug Use

Illicit substances that increase BP are sympathomimetics, potentiating the effect of catecholamines, including amphetamines and ecstasy, their illegal derivative, in addition to powder cocaine and smokable crack cocaine. ^5^ ^,^ ^759^ ^,^ ^760^ Amphetamine use causes a dose-dependent increase in BP, ^761^ leading to tachycardia tachycardia, palpitations, sweating, and arrhythmias, while ecstasy has other effects in addition to HR and BP increases (serotoninergic syndrome). ^762^

Intranasal cocaine use leads to a sudden and dangerous increases in BP levels within 15 minutes of use. In case of preexisting hypertension, higher BP elevations may occur. ^763^ Cocaine-induced vasoconstriction depends on the central sympathetic discharge, which s suppressed by the intact baroreceptor function. When the baroreflex tamponade is impaired, the result is adrenergic vasoconstriction and HC. ^764^

In lighter cases, benzodiazepines and sublingual NTG may be administered. In more severe cases, IV therapy will probably be required, the agents of choice are NTG, SNP, or phentolamine. ^759^ ^,^ ^760^ It is important to avoid BBs, since they may lead to alpha-adrenergic receptor stimulation in the presence of beta blockade, thus causing a coronary spasm. ^763^ An exception might be carvedilol, which is capable of mitigating HR and BP increases induced by smoking crack cocaine. ^765^ CCBs may also be used in cases of cocaine-induced AMI, where the assumed cause is coronary vasoconstriction. ^760^

A complicating factor of those intoxications, whether they are HUs or HEs, is the concomitant ingestion of high doses of caffeine (present in energy drinks), nicotine, or alcohol, which increase plasma NE levels. ^766^ In particular, alcohol and cocaine use in combination has a greater toxic effect than the use of either alone, ^767^ ^,^ ^768^ increasing the risk of sudden death 18- to 25-fold ^769^ due to increased bioavailability of cocaine. ^770^ Treatment includes the use of BBs, alpha-blockers, and CCBs, the latter administered before or after cocaine intake. ^760^ ^,^ ^771^

## 13.7.7. Accelerated/Malignant Hypertension

Malignant hypertension is characterized by the presence of severe general hypertension, retinopathy with papilledema, with or without renal and/or heart failure, fibrinoid necrosis of renal arterioles, and endarteritis obliterans, and may present with rapidly progressive and fatal clinical evolution. Elevated BP in the presence of retinal hemorrhages and exudates in the funds, but in the absence of papilledema, is known as accelerated hypertension. Currently, the terms “malignant” and “accelerated” are considered interchangeable, with “accelerated/malignant hypertension” used more often to define this form of HE, which, though less frequent, represents a devastating form of acute BP elevation. ^747^ ^,^ ^772^ ^,^ ^773^ The prognosis is almost always fatal if not properly recognized and left untreated, with two-year mortality rates of approximately 80%, primarily due to HF and CKD. ^774^ ^,^ ^775^ Effective treatment of malignant hypertension has significantly improved survival, but it is still accompanied by a high rate of complications. ^776^ The most rational way to manage is to prevent it by treating hypertension early and effectively. Individuals with severe hypertension who present with major LVH and renal failure should be treated as prior accelerated/malignant hypertension patients.

Patients should undergo intensive BP control using immediate-action vasodilator medications, such as SNP, which promotes fast BP control and makes individuals more responsive to classic antihypertensive therapeutics. ^164^ ^,^ ^732^ During acute control, oral antihypertensives should be administered, including diuretics, renin-angiotensin system blockers, BBs, direct-acting vasodilators (hydralazine), central adrenergic agonists (clonidine and methyldopa), and CCBs, when multiple medications are required. ^747^ ^,^ ^774^ BB use is indicated for cases of pulmonary congestion caused by diastolic dysfunction due to severe LVH. BP reductions should be gradual, keeping DBP levels above 100 mm Hg during the first few days of treatment. There may be an initial impairment in kidney function with high creatinine levels since the mechanism of autoregulation of renal flow is shifted to much higher levels than those found in mild hypertensive patients and normotensive individuals. Therefore, an adjustment period is required before returns to baseline levels. Sometimes, dialysis treatment may be required during the most acute stage. Antihypertensive treatment for this condition has had a significant impact on survival (LR: IIa; LE: B).

## 13.7.8. Hypertension with Multi-Organ Damage

Hypertension with multi-organ damage (MOD) is defined by the concurrent involvement of three of the four systems listed below: ^777^

• Renal (rapid decline of kidney function or proteinuria);
• Cardiac (major LVH or systolic dysfunction, or ventricular repolarization abnormalities, or increased troponin);
• Neurological (stroke or hypertensive encephalopathy);
• Hematological (microangiopathic hemolysis).

The definition of MOD hypertension (in the presence of multi-organ impairment) does not require the presence of Keith-Wagener Grade III or IV changes, which may be found at a later stage. ^778^ ^,^ ^779^ When comparing MOD hypertension to accelerated/malignant hypertension, the two are found to have analogous pathogeny, clinical significance, and prognosis, implying similar clinical management (LR: IIa; LE: B). ^777^ ^,^ ^780^

**Table:** 

Key Takeaways
Hypertensive crisis: acute elevation of systolic blood pressure (BP) ≥ 180 mm Hg and/or diastolic BP ≥120 mm Hg, which may or may not result in end-organ damage (EOD), divided into hypertensive urgencies (BP increase without EOD and no imminent risk of death, allowing for BP reduction within 24 to 48 h) and hypertensive emergencies (BP increase with acute EOD or in progress and immediate risk of death, requiring rapid and gradual BP decrease within minutes to hours using intravenous medication).
Hypertensive emergencies may manifest as a cardiovascular, cerebrovascular, renal or multi-organ event, or even as pre-eclampsia with severe features or eclampsia.
High BP without acute and progressive EOD rules out HE.
Uncontrolled hypertension from low adherence, difficult-to-control hypertensive pseudocrises, hypertensive urgencies, and hypertensive pseudocrises are common situations of high BP without acute or progressive EOD.
The severity of the clinical condition is not determined by absolute BP levels, but rather by the magnitude and timing of the increase. Numerical values act as a parameter, but should not be applied as absolute diagnostic criteria.

## 14. Hypertension in Older Adults

## 14.1. Introduction

The United Nations (UN) and the World Health Organization (WHO) consider as older adults all individuals age 60 and older. In high-income countries, where life expectancies are greater, the threshold has been raised to 65. ^781^ A special age group known as the “oldest old,” consisting of individuals ages 80 or older, represents the fastest-growing segment of the population. ^782^

The prevalence of multimorbidity increases with age, and over two thirds of the oldest old suffer from two or more chronic illnesses. ^783^ ^,^ ^784^ Based on a country-level study of the older adult population (ELSI-Brazil), over 60% of older adults suffered from multiple chronic illnesses, and hypertension (HT) was the second most prevalent, second only to chronic back pain. ^785^ These patients usually take multiple medications with hard-to-manage therapy regimens that increase their cost and the risk of drug interactions.

There is a direct and linear relationship between blood pressure (BP) and age, with the prevalence of HT going from approximately 7% in individuals ages 18 to 39 to over 60% in those 65 and older. The Framingham has shown that nearly two thirds of men and three fourths of women have HT at age 70. ^786^ ^,^ ^787^

Though epidemiological studies have suggested greater survival rates for individuals age 80 and older with high levels of BP, this may in part be caused by the fact that people with low BP have higher rates of multimorbidity and frailty, and therefore lower probability of survival. In the geriatric population, HT is the primary modifiable risk factor (RF) for cardiovascular morbidity and mortality, ^786^ even at more advanced ages. It is critical that we stress that HT is a modifiable RF for cognitive decline, dementia and disability. ^787^ ^,^ ^788^

In assessing mean survival rates for older adults, one should not use life expectancy at birth, but rather life expectancy “at life.” Therefore, life expectancy at age 80, in 2018, was 10.4 years for women and 8.6 years for men, more than enough time to enjoy the benefits of treatment for HT.

## 14.2. Physiopathological Mechanisms

Diastolic blood pressure (DBP) increases until approximately age 50, stabilizes from 50 to 60 and then decreases, while systolic blood pressure (SBP) tends to increase throughout the lifespan. Therefore, pulse pressure (PP = SBP – DBP), a useful hemodynamic index of arterial stiffness, increases with age. These changes are consistent with the idea that, for younger individuals, BP is largely determined by peripheral vascular resistance (PVR), while for older adults it is determined by central arterial stiffness. ^789^ ^-^ ^791^

The arterial wall thickening and endothelial dysfunction observed during the aging process are accompanied by increased stiffness and lower vascular compliance, attributed to a wide range of factors, such as salt sensitivity, chronic hemodynamic stress, and elastin fiber fragmentation and misalignment, with replacement by collagen fibers, facilitating the deposition of calcium ions. ^792^

Aortic stiffening caused by vascular aging accelerates pulse wave velocity (PWV) towards peripheral circulation (centrifugal) and reflected waves returning to the heart (centripetal). The overlap of those two waves during the proto-mesosystolic phase leads to the increases in SBP and wider PP seen in older adults. ^793^

Currently, carotid-femoral PWV measurement is considered the gold standard to assess central arterial stiffness. In the absence of comorbidities, older adults with velocities under 7.6 m/s are considered to have good vascular health and, in an isolated sample, represent fewer than 4% of individuals age 60 and older. ^794^ ^,^ ^795^ In a given urban region of Brazil, PWV values found in older adults, adjusted for BP, age and gender, averaged 9.1 m/s for normotensive individuals and 9.4 m/s for uncontrolled hypertensives. ^796^ On the other hand, for many older adults, PP amplification may be a better predictor of events and mortality than PWV. ^797^

## 14.3. Diagnosis and Therapeutic Decision

The presence of multiple comorbidities and polypharmacy may make investigating HT in older adults more difficult. Chapter 3 guidelines on BP measurement and physical and laboratory examinations should also be followed for this age group. However, the investigation of secondary causes of HT should proceed carefully and consider the risks and benefits for each procedure (see Chapter 15). ^798^

The clinical assessment of older patients, especially the oldest old, is different from traditional assessments. First, physicians should recognize that the appointment will require more time due to several factors, such as: complexity of multiple associated conditions, physical and cognitive slowness of patient, and presence of caretakers and family members, with whom the physician will have to discuss the issues inherent to the relevant therapeutics and clinical conditions. ^799^ Very frail older adults may require additional visits due to patient exhaustion. ^800^

BP measurements may produce inaccurate values due to greater blood pressure variability and a few idiosyncrasies. Major factors interfering with BP measurement in older adults are: 1. auscultatory gap; 2. pseudohypertension; and 3. postural and postprandial variations. ^801^ (see Chapter 3)

Out-of-office BP monitoring, either ambulatory (ABPM) or at home (HBPM), is increasingly valued and indicated as a diagnostic tool for SHT in older adults.. ^180^ ^,^ ^186^ Despite its limitations, self-measured BP should also be considered (see Chapter 3).

Proper blood pressure treatment and control for hypertensive older adults and the oldest old has unequivocal benefits, such as significant decreases in stroke, AMI, HF, and mortality, ^87^ ^,^ ^509^ ^,^ ^572^ ^,^ ^802^ ^,^ ^803^ in addition to preventing cognitive decline and possibly dementia. ^103^ ^,^ ^804^ ^-^ ^806^ On the other hand, exact BP levels for treating older patients, as well as treatment targets, have been the subject of debate, ^180^ and different guidelines provide different recommendations. ^5^ ^,^ ^37^ ^,^ ^807^ However, all guidelines, including this one, consider it key to perform individualized assessments. In addition to chronological age, we recommend weighing functional fitness, cognition, degree of frailty, patient expectations, comorbidities, end-organ damage and global CV risk, polypharmacy, and treatment tolerability. Recommended blood-pressure levels for older adults, both for initiating treatment and blood-pressure targets, can be found in Chart 14.1 .

**Chart 14.1:** – Hypertension treatment recommendations for older adults

	Office SBP		Office DBP	
Global condition^**1**^	Treatment threshold	Blood-pressure target ^4^ ^,^ ^5^	Treatment threshold	Target ^8^
**Healthy** ^**2**^	≥140 (I, A)	130-139 (I, A) ^6^	≥90	70-79
**Frail older adults** ^**3**^	≥160 (I, C)	140-149 (I, C) ^7^	≥90	70-79

*1: functional status is more important than chronological age; 2: including light frailty; 3: moderate to severe frailty; 4: including older adults with comorbidities: DM, CAD, CKD, stroke/TIA (not acute stage); 5: actively assess tolerability, including possible atypical symptoms; 6: stricter target (125-135 mm Hg) may be achieved in selected cases, especially for motivated older adults, < 80 years old, with optimum treatment tolerability; 7: higher limits in case of limited survival and absence of symptoms. BP reductions should be gradual; 8: DBP = avoid < 65-70 mm Hg in clinically manifested CAD patients. Note: out-of-office BP monitoring (ABPM/HBPM) should follow changes to the therapy regimen or be performed annually due to greater variability in BP with age, higher risk of orthostatic hypotension, and lower tolerability to inadequate treatment of white coat and masked hypertension.*

## 14.4. Treatment

There is no single therapeutic strategy for older adults, especially those over the age of 80 to 85 ( Chart 14.2 ). Therefore, other factors should be considered above age itself while planning the treatment: presence of comorbidities, autonomy, functional status, and degree of frailty (LR: I; LE: C). That stratification is a better predictor of possible complications both in the short and the long term in relation to different comorbidities. ^808^ ^,^ ^809^ No therapeutic intervention should be denied or withdrawn based on age alone (LR: I; LE: C).

**Chart 14.2:** – Challenges in treating SHT in older adults.

Most older adults are hypertensive, with high prevalence of ISH.
The challenges are not limited to age, but primarily to functional, social, nutritional, and mental status.
Survival rate is more closely tied to global functional status than to age itself.
A diagnosis of HT in older adults requires acknowledging their idiosyncrasies and the frequent use of out-of-office monitoring.
Therapeutic challenges are connected to adherence, presence or absence of polypharmacy, orthostatic hypotension, and comorbidities, such as urinary incontinence and fatigue, among others, common in older adults.
Clinical assessments should include functional tests, such as gait speed and the Clinical Frailty Scale.
Treatment prevents CV events, death, and cognitive decline, even at advanced ages.
LSCs work, but require greater care.
DIUs, CCBs, ACEIs/ARBs should be used in isolation or combined as initial therapies; BBs, when there is formal indication for their use.
Weight loss and loss of organ reserve at advanced ages may be associated with gradual decreases in BP and may imply in treatment deintensification.
In older adults receiving palliative care for advanced disease or severe frailty, the primary treatment objective is symptom control.

*ACEI: angiotensin-converting enzyme inhibitor; ARB: angiotensin II AT1 receptor blocker; BB: beta-blocker; CCB: calcium channel blocker; CV: cardiovascular; DIU: diuretic; HT: hypertension; ISH: isolated systolic hypertension; LSC: lifestyle changes.*

### 14.4.1. Nonpharmacological Treatment

All lifestyle change (LSC) measures that apply to younger individuals (see Chapter 8) are valid for older adults as well (LR: I; LE: B), but require greater care and more thoughtful consideration of their actual benefits—and potential risks. Older adults are more salt-sensitive, and dietary salt restrictions are more effective for this age group. ^420^ The TONE study showed that there was a 4.3 mm Hg decrease in SBP and a 2.0 mm Hg decrease in DBP for for every 80 mmol of sodium (= 2.0 g of salt) reduction in daily salt intake. Combined with concurrent weight loss, the BP reduction effect was potentiated. ^810^ Excess reduction in salt intake may lead to hyponatremia and loss of appetite and can cause malnutrition. Potassium-rich diets should be encouraged, ^811^ but require greater attention to the risk of hypercalcemia due to the frequent presence of chronic kidney disease (CKD) and the use of medications that lower potassium excretion.

Physical exercise and aerobic and resistance training are critical for older adults and should be recommended. ^52^ In older adults, especially the frail and sarcopenic, weight loss without physical exercise and adequate protein intake may lead to loss of muscle mass and worse functional fitness.

Smoking and alcohol abuse are still prevalent in older populations and should be discussed. Likewise, all medications in use by the patient need to be analyzed, as some may cause BP increases.

In recommending LSCs, physicians should consider the patient's degree of frailty, functional fitness, and other social and clinical aspects. Follow-up by a multidisciplinary team (see Chapter 7) and family/caretaker engagement are even more important for older patients.

### 14.4.2. Pharmacological Treatment

In choosing antihypertensive medication(s) for older adults, physicians should consider the high rates of comorbidities, specific contraindications, likely drug interactions and cost, as well as the availability of and clinical experience with the medication (LR: I; LE: C). Prudence dictates initiating monotherapy or combination therapy at low doses and, if needed, increase or gradually combine antihypertensives at intervals of at least two weeks (LR: I; LE: C).

Chapter 9 details when to give preference or to avoid specific antihypertensives and their combinations. Here, we highlight aspects peculiar to older patients.

The first antihypertensive may be a thiazide (or thiazide-like) diuretic, a calcium channel blocker (CCB), or a renin-angiotensin-aldosterone system (RAAS) blocker: an angiotensin-converting enzyme inhibitor (ACEI), or an angiotensin II AT_1_receptor blocker (ARB). A large number of clinical trials have studied these four classes, and they are widely used in guidelines for older adults. ^807^ ^-^ ^809^ In terms of monitoring, indications and care are similar to those for other adults (see Chapter 9).

Beta-blockers (BBs) should not be used as initial monotherapy for older adults, ^809^ except in the presence of certain comorbidities, which may actually make their indication mandatory, such as heart failure (HF) or acute coronary failure (LR; I; LE: A). ^812^ ^,^ ^813^ Patients suffering from bronchial asthma or chronic obstructive pulmonary disease (COPD), but with clinical indication for BBs, should be carefully treated with cardioselective BBs and after receiving respiratory compensation, and should bot be deprived of their benefits. ^814^ When used in combination with acetylcholinesterase inhibitors, frequently used for Alzheimer's disease, they may induce severe bradycardia. ^815^

Other classes of antihypertensives (centrally acting medications, aldosterone antagonists and direct vasodilators), as well as other invasive treatments of the sympathetic nervous system, should be seen as the exception and not used as a matter of course to treat older patients (LR: III; LE: C) (see Chapter 9).

The risk of falls in older adults can increase during the first weeks of treatment with DIUs, and with all other medication classes in the first day. In the long run, antihypertensives may actually have protective effects. ^816^ ^,^ ^817^

## 14.5. Special Situations

There is some disagreement between results from observational studies and those from randomized clinical trials (RCTs). They come primarily from the fact that frail, multimorbid older adults are underrepresented in RCTs and the high risk of bias in nonrandomized and observational studies, where the longer survival of patients with high BP may be explained by their greater organ reserve. ^818^ ^-^ ^822^

### 14.5.1. Functional Status and Frailty: Assessment and Implications

In older adults, and especially in the oldest old, functional status and frailty require special attention. With the use of systematic tests and scales, the comprehensive geriatric assessment (CGA) enables an accurate global assessment of older adults and the development of therapeutic strategies. ^823^ ^,^ ^824^ Though the ideal form of assessment, it may require the presence of a geriatrician or gerontologist. In day-to-day care for older hypertensive patients, clinicians should assess functional status and capacity to perform the activities of daily living. ^825^ ^,^ ^826^

Routine use of the gait speed test (GST) is recommended, as it is easily performed as part of regular visits and has been shown to be a prognostic predictor of survival. ^827^ ^,^ ^828^ Patients are considered frail or at risk of frailty when GST < 0.8 m/s (unable to walk 6 m in less than 8 seconds), requiring further investigation. ^820^ ^,^ ^829^ In addition, this guideline recommends the use of the “Escala Clínica de Fragilidade,” which has already been translated into Brazilian Portuguese and validated in Brazil, ^830^ based on the Canadian *Clinical Frailty Scale* , widely tested and deployed, as it is simple and reliable, provides a global view of patient condition, and determines the prognosis. ^827^ ^,^ ^828^ ^,^ ^831^ ^,^ ^832^

Frailty is associated with higher risk of HT, subclinical disease, CV events, and death. ^821^ ^,^ ^833^ ^-^ ^835^ Adequate HT control may influence the trajectory of frailty. On the other hand, advanced levels of frailty are associated with lower BP values, lower body mass index (BMI), less muscle mass, impaired cognition, and higher mortality. ^335^ ^,^ ^836^

Functionally active and independent patients with no severe comorbidities have enough organ reserve and mean survival to enjoy most of the benefits from antihypertensive treatment and should, if well tolerated, have the same blood-pressure targets as younger older adults (LR: I; LE: B). ^335^ ^,^ ^805^ ^,^ ^825^ ^,^ ^831^ On the other end of the scale, individuals with major functional loss, sarcopenia, frailty, or advanced dementia, or unable to perform self-care activities, should have their whole treatment regimen and blood-pressure targets reassessed. ^821^ ^,^ ^825^ ^,^ ^831^ ^,^ ^837^ The primary goal is to improve symptoms and quality of life. Frailer older adults were systematically excluded from several clinical trials, so studies focused specifically on this population are key. ^307^ ^,^ ^821^

Between the two extremes we find older adults with intermediate functional status and multiple non-CV comorbidities who may require very challenging therapeutic decisions. For them, deeper assessments may be critical to define the real risk-benefit ratio and to individualize therapeutic strategies. ^307^ ^,^ ^825^ ^,^ ^831^ ^,^ ^838^ ^,^ ^839^

### 14.5.2. Cognitive Decline and Dementia

In addition to its well-known role as the primary cause of strokes, HT has also been implicated as a pathogenic factor in cognitive impairment, both vascular and from Alzheimer's disease, the main causes of dementia in older adults, and more markedly in the long run. ^840^ ^-^ ^842^

In several epidemiological studies, use of antihypertensive medications is associated with less cognitive decline and dementia, especially in the long run. ^843^ RCTs have found decreased white matter damage and cognitive decline from treatment for HT, with intensive treatment even more efficient in that regard. ^5^ ^,^ ^37^ ^,^ ^844^ RCTs have not yet clearly proven decreased dementia. This may be because cognition was not the primary outcome in those RCTs, the lack of uniformity in the definition of dementia and in what tests were used, or the short duration of the trials. ^806^ ^,^ ^845^

### 14.5.3. Polypharmacy and Adherence

Polypharmacy, defined as the regular use of five or more medications, is increasingly frequent with age, ^846^ and is associated with higher probability of adverse events (AE), drug interactions, and worse adherence to treatment. ^845^

Improper adherence to pharmacological treatment is a frequent issue for older adults and one of the primary causes of inadequate BP control. Determinants of poor adherence to therapeutic regimens include misunderstanding the disease, polypharmacy, multiple daily doses, and side effects. ^847^ To that end, this guideline recommends, especially for older adults with polypharmacy, the periodic review of each prescribed medication, AE assessments, ^848^ and that antihypertensive treatment include as few pills and tablets per day as possible, using single-tablet fixed-dose combinations, in addition to emphasizing nonpharmacological measures (LR: I; LE: A) (see Chapter 17).

### 14.5.4. Deintensification and Deprescription

In different clinical situations, it may be necessary to gradually lower dosage or even deprescribe antihypertensive medications; these include symptomatic hypotension; adverse reactions; persistently below-target SBP detected out-of-office or at the physician's office; ^822^ ^-^ ^824^ changing blood-pressure targets to less rigid levels (keeping in mind that BP tends to decrease at very advanced ages due to progressively lower organ reserve and greater frailty); and end-of-life palliative care. ^837^

A key issue in treating HT in older adults, especially in the oldest old, is the careful monitoring of AEs and tolerability, with special attention to atypical signs and symptoms. Discontinuing antihypertensives seems to be safe in the short run, but without proven benefits for cognition or functional fitness to perform the activities of daily living (ADLs). ^838^ ^,^ ^849^ ^,^ ^850^

### 14.5.5. Orthostatic and Postprandial Hypotension

Because of arterial stiffness, variations in volume significantly interfere with HT control. Older adults have weaker baroreceptor reflex to hypotension, and so are likely to be subject to orthostatic hypotension (OH) and postprandial hypotension (PPH). A higher rate of neurodegenerative disease is also associated with this condition. ^851^ Approximately 20% of older adults have OH and around 30% of institutionalized older adults experience hypotension after meals. ^852^ ^,^ ^853^ Therefore, older adults should be carefully monitored for OH and PPH (LR: I; LE: B).

In RCT, HT control led to fewer CV events with no increased risk of OH or falls with injuries. ^854^ ^-^ ^856^ Poorly controlled HT and certain antihypertensive medications, such as alpha-blockers, can cause or worsen OH. The best option to control OH is to use nonpharmacological interventions, such as adequate hydration, adequate sodium intake, slow rise from decubitus, higher headrests, and compression socks. ^853^

In postprandial hypotension, older adults should avoid large meals and high intake of alcohol and carbohydrates. They should also avoid exercising after meals. In addition, medication prescriptions should be revised in order to lower polypharmacy as much as possible, paying special attention to drugs that may be contributing to OH or PPH, such as DIU, sympatholytics, nitrates, and tricyclic antidepressants.

**Table:** 

Key Takeaways
HT prevalence increases progressively with age, as do other RFs, leading to sharp rises in CV risk among older adults.
Proper diagnosis requires caution with the peculiarities of blood pressure measurement, and out-of-office BP reagins (SMBP, HBPM, ABPM) are key in older adults, for whom inadequate treatment poses more risk.
Functional and cognitive status should be evaluated. Therapeutic decisions and BP targets should be based on functional status and survival over chronological age.
Treatment lowers CV risk as well as the risk of cognitive decline. Comorbidities, more frequent in older adults, should guide what medications are chosen or avoided.
Special attention should be given to family support networks, polypharmacy, adherence, and higher risk of OH.

## 15. Secondary Hypertension

## 15.1. Introduction

Secondary hypertension is the form of hypertension (HT) due to an identifiable cause and treatable by a specific intervention which can improve or resolve blood pressure control. The actual prevalence of secondary HT is unknown, but estimated at 10 to 20%, ^857^ and may be higher or lower, depending on the population cohort (especially in terms of age), diagnostic resources available and physician expertise. It should be investigated when signs (clinical history, physical examination, or routine tests) lead to clinical suspicion ^258^ ^-^ ^860^ ( Chart 15.1 ).

**Chart 15.1:** – Signs of secondary hypertension

1	Stage 3 hypertension before age 30 or after 55
**2**	Resistant or refractory hypertension
**3**	Use of exogenous hormones, medications or other substances that may increase BP (see Chart 15.7 )
**4**	Pheochromocytoma triad: palpitations, sweating, and headaches
**5**	Signs of obstructive sleep apnea
**6**	Typical facies or phenotype for diseases that progress to hypertension
**7**	Presence of bruits in arterial areas or abdominal masses
**8**	Asymmetry or absence of LL pulse
**9**	Spontaneous or diuretic-induced severe hypopotassemia (< 3.0 mEq/L)
**10**	Abnormal urine test (glomerular hematuria (dysmorphic) or presence of albuminuria/proteinuria), lower estimated GFR, increased serum creatinine, or alterations in renal imaging

The main causes of secondary hypertension, discussed in this chapter, are shown in Figure 151 . Diagnostic investigation can focus on the age of the patient and the type of sign, as seen in Chart 15.2 . Patients with secondary HT are under higher CV and renal risk and have more end-organ damage due to higher and more sustained BP levels, as well as the activation of hormonal and molecular mechanisms. ^859^ ^,^ ^861^

**Chart 15.2:** – Major endocrine and nonendocrine causes of secondary HT, signs, and diagnostic screening

Clinical findings	Diagnostic suspicion	Additional findings
**Nonendocrine causes**		
Edema, anemia, anorexia, fatigue, high creatinine and urea, and changes in urinalysis or imaging examinations	Renal parenchymal disease	Creatinine and eGFR calculation (I: B), renal US, urinalysis (I: C) for dysmorphic proteinuria/hematuria. Albuminuria or proteinuria/urine creatinine ratio where indicated (LR: I; LE: B)
Sudden-onset HT or apparently unexplained worsening before age 30 or after 55, resistant or refractory HT or MHT, abdominal murmur, sudden APE, unexplained alteration in renal function or caused by RAAS blockers, kidney asymmetry > 1.5 cm	Renal artery stenosis	Renal Doppler US with flow velocity measurement and resistivity index (screening, but observer-dependent) (LR: I; LE: B) and/or captopril radioisotope renography (LR: III; LE: C), MNR angiography (eGFR > 30 mL/min, BOLD or digital subtraction) (LR; I; LE: B) or spiral CT (eGFR > 60 mL/min (LR: I; LE: B) Gold standard: conventional renal arteriography (LR: I, LE: A)
Higher frequency in men or postmenopausal women, snoring on most nights, sleep fragmentation with respiratory pauses or choking, excessive daytime sleepiness, nonrestorative sleep, fatigue, nocturia, morning headaches, MS	Obstructive sleep apnea (OSA)	Surveys have low accuracy for screening purposes Gold standard: polysomnography or home respiratory polygraphy. AHI < 5 events/h: no OSA; AHI 5-14.9 events/h: mild OSA; AHI 15-29.9 events/h: moderate OSA; AHI ≥ 30 events/h: severe OSA
Weakness in LLs, absent pulse or diminished amplitude, HT with SBP 10mm Hg > in ULs over ULs, interscapular and thoracic systolic murmur	Coarctation of the aorta	Chest X-ray, screening echocardiogram CT angiography of the chest or, preferably, aortic MNR (gold standard) Invasive angiography, only when additional data are required
Endocrine causes		
RHT or RfHT and/or with hypopotassemia (non obligatory) and/or with adrenal nodule	Primary hyperaldosteronism (hyperplasia or adenoma)	Aldosterone measurements (>15 ng/dL) and plasma renin activity/concentration; aldosterone/renin ratio > 30 Confirmatory testing (see Chart 15.7 ) Imaging examinations: thin-slice CT or MNR. Selective aldosterone and cortisol adrenal sampling to identify subtype, when indicated (LR: I; LE: B)
Paroxysmal HT with triad consisting of headaches, sweating, and palpitations	Pheochromocytoma and paragangliomas	Free plasma metanephrines and/or urinary fractionated metanephrines (LR: I, LE: A). CT (LR: IIa, LE: B) (screening), MNR (LR: I; LE: B) and scintigraphy (LR: IIa, LE: C) where indicated
Fatigue, weight gain, hair loss, diastolic HT, muscle weakness, sleepiness	Hypothyroidism	Screening: TSH and free T4 (LR: I, LE: B)
Increased sensitivity to heat, weight loss, tachycardia/palpitations, exophthalmos, hyperthermia, hyperreflexia, tremors, goiter	Hyperthyroidism	Screening: TSH and free T4 (LR: I; LE: B)
Renal lithiasis, osteoporosis, depression, lethargy, muscle weakness or spasms, thirst, polyuria, polydipsia, constipation	Hyperparathyroidism (hyperplasia or adenoma)	Total and/or ionized calcium, phosphorus, PTH, 24-hour urine calcium and vitamin D level (LR: I; LE: B)
Weight gain, decreased libido, fatigue, hirsutism, amenorrhea, moon face, “buffalo hump”, purple striae, central obesity, hypopotassemia	Cushing’s syndrome (hyperplasia, adenoma and excess secretion of ACTH)	Baseline cortisol, midnight salivary cortisol, 24-h urinary free cortisol, and betamethasone or dexamethasone suppression test (take 1 mg dexamethasone between 11 PM and midnight and measure serum cortisol level at 7-8 AM of the following morning). CT, MNR (LR: I; LE: B)
Increased visceral or central fat	Obesity Class 1: BMI 30 to < 35 kg/m ^2^ Class 2: BMI 35 to < 40 kg/m ^2^ Class 3: BMI ≥ 40 kg/m ^2^	BMI (weight in kg/height in m ^2^ ) and abdominal circumference (> 102 cm in men and 88 cm in women) Imaging examinations: DEXA (gold standard), CT, MNR (clinical trials) (LR; I; LE: B)
HT in up to 30% of cases, in addition to diabetes, LVH, and OSA. Other symptoms: visual defects, cranial nerve palsy, headaches, macrognathism, growth of feet and hands, soft tissue hypertrophy, macroglossia, musculoskeletal complications	Acromegaly	IGF-1 (I, B) measurement, GH serum level and GH after oral glucose overload (I, B) Location: sella turcica MNR (preferred) or CT scan

*ACTH: adrenocorticotropic hormone; AHI: apnea–hypopnea index; APE: acute pulmonary edema; BMI: body mass index; BOLD: blood oxygen level-dependent; CT: computed tomography; DEXA: dual-energy x-ray absorptiometry scanning; eGFR: estimated glomerular filtration rate; GH: growth hormone; IGF-1: insulin-like growth factor 1; LVH: left ventricular hypertrophy; MHT: malignant hypertension; MNR: magnetic nuclear resonance; OSA: obstructive sleep apnea; PTH: parathormone; RAAS: renin-angiotensin-aldosterone system; RHT: resistant hypertension; TSH: thyroid-stimulating hormone. LLs: lower limbs; ULs: upper limbs.*

## 15.2. Nonendocrine Causes

### 15.2.1. Chronic Kidney Disease (CKD)

CKD is defined by its cause and by functional or morphological abnormalities persisting for over three months with consequences for the patient's health. It is characterized by an estimated glomerular filtration rate (eGFR) < 60 mL/min or alterations in urine tests, especially albuminuria (30 mg/24 h or 30 mg/g albumin/creatinine ratio) and/or in renal morphology (LR: I; LE: C). ^862^ CKD classification and prognosis are based on eGFR and albuminuria levels (Chapter 4). HT is both cause and consequence of CKD and becomes progressively worse as kidney function declines, affecting 90% of stage 5 patients (LR: I; LE: A). ^863^ ^,^ ^864^

Tests for hypertensive patients should include serum creatinine and eGFR calculation (LR: I; LE: B) as well as urine test (LR: I; LE: C) for CKD screening. ^859^ Renal ultrasound, computed tomography (CT), or magnetic nuclear resonance (MNR) may also be necessary. Kidney biopsies are only required when there are rapid declines in eGFR, glomerular hematuria, and/or proteinuria/albuminuria in addition to HT. ^865^ HT accelerates the progression of CKD, and lowering BP attenuates the course of CKD. ^863^ ^,^ ^864^ Treatment goals and therapy regimens indicated for BP control in CKD patients can be found in Chapters 6 through 9.

### 15.2.2. Renovascular Hypertension (RVH)

Renovascular hypertension (RVH) is a common and potentially reversible cause of secondary HT due to partial or total, uni- or bilateral renal artery stenosis (RAS) or of one of its branches, triggered and maintained by significant renal ischemia. It is usually found in obstructions greater than 70%. ^164^ Its prevalence and etiology vary with age and blood pressure levels. In young adults, especially women, RVH is more frequently caused by fibromuscular dysplasia (FMD). In older adults, the most common cause is atherosclerosis, usually accompanied by peripheral and/or coronary atherosclerosis. ^866^ The clinical indicators of RVH can be found in Chart 15.3 .

**Chart 15.3:** – Clinical indicators of renovascular hypertension

Onset of hypertension before age 30
Onset of severe hypertension after age 55 associated with chronic kidney disease and congestive heart failure
Hypertension and abdominal murmur
Rapidly progressive and persistent hypertension in previously well-controlled patient
Resistant or refractory hypertension
Hypertensive crisis with end-organ damage (acute renal failure, congestive heart failure, hypertensive encephalopathy, grade 3 and 4 hypertensive retinopathy)
Worse renal function after treatment with renin-angiotensin system blockers
Unexplained renal atrophy, renal asymmetry, or unexplained worse renal function
Acute pulmonary edema

*Aboyans et al., 2018.^*867*^*

In patients with potential signs of RVH, the physician should consider diagnostic tests for those with fewer morbidities for whom revascularization treatment is indicated. ^867^ ^,^ ^868^ Renovascular disease has heterogenous clinical manifestations. The damage may evolve with minimal or even silent hemodynamic repercussions before progressing to critical levels associated with triggering hypertensive physiopathological mechanisms and renal ischemia. Revascularization procedures are indicated for FMD patients and patients with atherosclerotic etiology who cannot control BP or are suffering progressive loss of renal function or decompensated heart failure (acute pulmonary edema, heart failure and refractory angina). ^869^ A cost-effective investigation requires proper selection of candidates as well as anatomical and functional assessment of the stenosis. ^866^

The gold standard is still conventional renal arteriography, but it is invasive and should not be used as the first procedure (LR: I; LE: B). BOLD or digital subtraction MNR angiography (LR: II; LE: B) and spiral CT are as accurate as ultrasounds, and have higher sensitivity and specificity (LR: I; LE: B). Renal Doppler US is the recommended noninvasive method for screening purposes, with 75% sensitivity and 90% specificity. ^267^ ^,^ ^869^ ^-^ ^871^

The treatment objectives for RVH are reducing the morbidity and mortality associated with high BP and protecting renal function and circulation. Randomized clinical trials ^872^ and a meta-analysis ^873^ have shown ^874^ ^,^ ^875^ that pharmacological treatment matches revascularization, with similar rates of BP control and cardiovascular mortality.

RAAS-blocking medication is recommended to lower hyperfiltration in the contralateral kidney and proteinuria in unilateral RVH with adequate potassium and creatinine monitoring. The efficacy of pharmacological optimization is an important element for decisions about whether an invasive procedure is indicated. ^874^ Atherosclerotic RVH requires lifestyle changes, smoking cessation, glycemic control, and prescription of statins and antiplatelet drugs, unless contraindicated. ^874^ ^,^ ^876^

If the blood-pressure target cannot be reached and/or there are other associated clinical conditions, such as RHT or RfHT, progressive kidney dysfunction or APE episodes, the invasive procedure may be recommended, conditional on the patient's acceptance. The actual benefits of invasive treatment are controversial, and clinical trials are still needed to identify the specific population that would benefit from this sort of treatment. ^877^ ^,^ ^878^ Diagnostic recommendations for renovascular disease can be found in Chart 15.4 .

**Chart 15.4:** – Recommendations for diagnosis of renovascular disease

Recommendation	LR	LE
Doppler ultrasound of renal arteries (screening), spiral computed tomography, magnetic resonance angiography	I	B
BOLD or digital subtraction angiography may be indicated to confirm diagnosis of renal artery stenosis detected by other methods in patients with high probability of renovascular disease	IIB	C
Renal scintigraphy, serum renin before and after captopril and venous renin not indicated for screening renal artery stenosis	III	C

*Aboyans et al., 2018.^*867*^*

## 15.3. Fibromuscular Dysplasia

Fibromuscular dysplasia (FMD) is an idiopathic, segmental, stenotic, nonatherosclerotic, and noninflammatory disease of the small- and medium-caliber muscular arteries. The lesions may be symptomatic or clinically silent, hemodynamically significant or not. Approximately 80 to 90% of patients are females. The First International Consensus ^879^ recommends angiographic classification for focal and multifocal FMD. For screening purposes, Doppler ultrasound of renal arteries is recommended. Other imaging examinations coincide with those used for atherosclerotic RVH: spiral CT if eGFR > 60 mL/min or MNR if eGFR > 30 mL/min. ^879^ Renal artery angiography is the gold standard to identify damage to the renal artery. Measuring the translesional gradient to determine the hemodynamic significance of the stenosis is recommended, especially in multifocal lesions. Identification of other vascular segments affected by the disease and investigation of aneurysms and dissections are recommended ^880^ ( Figure 152 ).

Isolated angioplasty is the recommended procedure, with stenting in case of complications (arterial rupture or dissection). In the absence of contraindications, continuous antiplatelet therapy with acetylsalicylic acid at 75 to 100 mg/day is indicated to prevent thrombotic complications, and dual antiplatelet therapy may be used for a short period of four to six weeks. ^881^ Doppler ultrasound of renal arteries 30 days after angioplasty is recommended, repeated every six months for the first two years and annually afterward, to detect restenosis. ^879^ As a matter of course, all patients should be included in follow-up, and optimally undergo yearly clinical and imaging assessments.

### 15.3.1. Coarctation of the Aorta

Coarctation of the aorta is a congenital anomaly leading to narrowing of the aorta, usually juxtaductal, proximal to the ductus arteriosus or ligament. It is usually underdiagnosed, with diverse clinical presentation, from early symptoms at birth (severe) to asymptomatic into adulthood, ^882^ depending on the site and severity of the coarctation as well as the frequent presence of other congenital heart diseases impacting prognosis. ^883^ The definition of significant coarctation requires pre- and post-coarctation pressure gradient > 20 mm Hg. Clinical suspicion is based on symptoms (resistant or refractory HT, epistaxis, headache and weakness of the legs on exertion, manifestations of HF, angina, aortic dissection, or intracerebral hemorrhage) and physical examination (hypertension in the upper limbs [ULs] with SBP at least 10 mm Hg higher in the brachial artery than in the popliteal artery; absent or diminished pulse in lower limbs [LLs]; interscapular and thoracic systolic murmur). ^164^ Diagnosis is based on imaging examinations: chest X-ray (thoracic aorta with pre- and post-stenosis dilations, costal corrosion); echocardiogram, the primary screening examination (posterior protrusion, expanded isthmus, transverse aortic arch, and high-velocity continuous jet through the coarctation site); CT or MNR angiography ^884^ in case of poor acoustic window. The MNR is considered the gold standard for assessment and post-intervention follow-up and, in young individuals, does not require invasive preoperative angiography, indicated when other imaging methods cannot provide visualization of the coarctation, and in older individuals who may have CAD. Intervention treatment includes angioplasty, implantation of vascular endoprosthesis, or open surgery (hypoplasia of the aortic arch and/or need for coarctation resection). The perioperative mortality rate is very low and prognosis is relatively good, though patients with coarctation of the aorta have higher and earlier incidence of CV disease than the general population and require continuous monitoring. ^885^ The BP response to intervention treatment depends on the duration of AH prior to surgery and the patient’s age.. ^886^ Though many lower BP after an invasive procedure, most develop exercise-induced HT. The drugs of choice for both the preoperative period and residual BP after surgery are beta-blockers (BBs) and angiotensin-converting enzyme inhibitors (ACEIs) or angiotensin II AT1 receptor blockers (ARBs). ^885^ ^,^ ^887^ Renin-angiotensin-aldosterone system blockers (SRAA) should be used with caution in the preoperative period in order to avoid major decreases in blood flow distally from the coarctation, which would trigger acute kidney damage. ^885^ ^,^ ^887^

### 15.3.2. Obstructive Sleep Apnea (OSA)

#### 15.3.2.1. Concept and Epidemiology

OSA is a clinical condition characterized by the intermittent collapse of the upper airways during sleep, causing total (apnea) and partial (hypopnea) obstructions. ^888^ Respiratory pauses lead to greater respiratory efforts and lower intrathoracic pressure, which cause increased left ventricle transmural pressure, cyclical dips in oxygen saturation (intermittent hypoxia), hypercapnia (usually mild), and sleep fragmentation. ^889^ The mechanisms involved with HT include activation of the sympathetic nervous system, systemic inflammation, increased production of reactive oxygen species, and endothelial dysfunction, among others. ^889^

Traditionally, OSA severity is determined by adding up apnea and hypopnea events (known as apnea-hypopnea index [AHI]), as determined by objective sleep testing: AHI < 5 events/h: no OSA; AHI 5-14.9 events/h: mild OSA; AHI 15-29.9 events/h: moderate OSA; AHI ≥ 30 events/h: severe OSA. The prevalence of OSA in the general population is high, depending on the diagnostic criteria adopted. In adults, it affects approximately 9.6% of women and 24.8% of men. ^890^ In hypertensive patients in general, it is estimated that 56% suffer from some degree of OSA. ^891^ ^-^ ^894^ For those with resistant HT, the prevalence is estimated at > 60% and is likely the most frequent cause associated with secondary HT, ^895^ though it does not mean OSA is the only cause in most cases. Though there is evidence that normotensive OSA patients progress to higher incidence rates of HT regardless of other risk factors, ^896^ ^,^ ^897^ in clinical practice, OSA frequently arises in previously hypertensive individuals. However, this in no way minimizes the importance of OSA: there is evidence that the association between OSA and HT is linked to higher rates of end-organ damage compared to hypertensive patients without OSA. ^895^ ^,^ ^896^

#### 15.3.2.2. Clinical Presentation and Screening of OSA in Hypertension

In the general population, some predisposing factors and clinical signs and symptoms should be assessed during examination and may reinforce the clinical suspicion of OSA ^888^ ( Chart 15.5 ). OSA prevalence rates are two to three times higher in men than in women, but is also common in the latter group, especially after menopause.

**Chart 15.5:** – Frequency of primary risk factors and symptoms/clinical signs suggestive of obstructive sleep apnea (OSA)

Characteristics	Measures
Risk factors	**Odds ratio**
Overweight vs eutrophic	2.3-3.4
Obese *vs* eutrophic	4.0-10.5
Male *vs* female	1.7-3.0
Age (10-year increments)	1.4-3.2
Postmenopausal (for women)	2.8-4.3
Clinical signs and symptoms	**Prevalence (%)**
Excessive sleepiness, fatigue, or nonrestorative sleep	73-90
Reported snoring most nights	50-60
Respiratory pauses and choking observed by other person	10-15
Nocturia (2 or more times per night)	30
Nighttime gastroesophageal reflux	50-75
Morning headaches	12-18

*Adapted from Gottlieb et al., 2020.^*888*^*

However, many of these signs and symptoms may be less prominent in HT. For instance, daytime sleepiness is often absent in HT, especially in patients with resistant HT. ^899^ Screening surveys for OSA in the general population perform poorly, especially in resistant HT patients. ^900^ ^-^ ^903^ It should be mentioned that some findings in BP patterns may help screen for patients with OSA. Recent data suggest that changes in nighttime BP dipping, especially the ascending waveform (mean BP during sleep higher than when awake), increase the odds of OSA three to four times. ^904^

#### 15.3.2.3. Impact of Treatment of OSA on BP

The treatment of choice for OSA, especially in moderate to severe cases, is the use of a continuous positive airway pressure (CPAP) device. ^888^ Mandibular advancement, oropharyngeal exercises, positional therapy, and surgery can be used in less severe and select cases of OSA. ^905^

Overall, treatment of OSA has a modest impact on BP (around 2 to 3 mm Hg). ^906^ These results are partially justified by the following factors: 1) many trials and meta-analyses combine controlled and uncontrolled hypertensive and normotensive participants; ^906^ and 2) adherence to CPAP use is not always adequate. ^906^ Randomized trials have shown that the impact of OSA treatment on BP is greater for RHT patients (circa 5 mm Hg, on average), but generally do not lead to blood pressure control them. ^907^ ^-^ ^909^ A study found that the presence of altered nighttime dipping was a predictor of better response from CPAP use in lowering BP for OSA patients. ^910^ Another poorly understood finding is that individuals afflicted with excessive sleepiness have greater decreases in BP. ^911^ Chart 15.6 details a few predictors of better blood pressure response to CPAP use. ^911^

**Chart 15.6:** – Predictors of better blood pressure response to CPAP use

Clinical characteristics
Patients with better adherence to CPAP (usually > 4 hours per night)
Patients with excessive daytime sleepiness
Patients with resistant hypertension
Patients with altered nighttime BP dipping

#### 15.3.2.4. Antihypertensive Treatment in Hypertensive Patients with OSA

Thus far, there is no conclusive evidence than any particular class of antihypertensive medication is preferable for hypertensive patients suffering from OSA. ^905^ A few aspects deserve to be highlighted here:

In general, the effect of antihypertensives seems to be more effective than CPAP use on lowering BP, but combining CPAP use and an antihypertensive has additional benefits, especially for nighttime BP; ^912^

Though more effective than CPAP alone, pharmacological treatment for HT usually does not improve the severity and symptoms of OSA. Even the effect of some diuretics and salt restriction on OSA severity (based on the theory that overnight rostral fluid shift favors upper airway collapsibility) ^913^ has a very modest impact on the severity of OSA. ^914^ ^,^ ^915^

## 15.4. Endocrine Causes

### 15.4.1. Primary Hyperaldosteronism (PH)

HT accompanied by supressed plasma renin activity (PRA) and increased aldosterone excretion characterizes primary aldosteronism. ^916^ PH was considered a rare form of secondary HT (1%), but may currently be found in 22% of RHT patients. ^917^ ^,^ ^918^ Gordon et al. found the incidence of PH in the primary hypertensive population to range from 5 to 15%, and to probably be approximately 12%. ^919^ Bilateral cortical adrenal hyperplasia is the most frequent cause of PH (50-60%), while aldosterone-producing adenomas (APA) account for 40% of PH cases. ^920^ Aldosterone-producing cortical adrenal carcinomas and unilateral cortical adrenal hyperplasia are less frequent causes of PH.

The main confirmatory tests for PH are listed in Chart 15.7 , ^920^ ^-^ ^924^ while the diagnostic investigation flow chart can be found in Figure 15.3 .

**Figure 15.3.** – Flow chart of diagnostic investigation of primary hyperaldosteronism.

Adapted from Vilela & Almeida, 2016. ^924^

**Chart 15.7:** – Confirmatory testing for primary hyperaldosteronism

Test	Procedure	Dosage	Results	Disadvantages
Saline infusion test	Infusion of 2 L of 0.9% saline solution over 4 h (start from 8 to 9:30). In the sitting position, has higher sensitivity for PH diagnosis.	K^+^, aldosterone (A), renin (R) at t = 0 and after 4 h.	Sitting: A values > 6.0 to 10 ng/dL at the end of the test are positive. The cutoff value with highest sensitivity/specificity is 6.0 ng/dL. Lying: A values > 6.8 to 10 ng/dL at the end of the test are positive. The cutoff value with highest sensitivity/specificity is 6.8 ng/dL.	Side effects: hypertensive crisis, hypervolemia Contraindicated for patients with severe HT, decompensated HF, renal failure, and severe hypokalemia.
Captopril challenge test	50 mg of captopril orally with patient remaining sitting for 2 h.	A, R, K^+^and cortisol at times 0, 1 h, and 2 h.	A > 8.5-13.9 ng/dL or suppression of A > 30%. Subtract percentage cortisol decrease from percentage A decrease.	Side effects: hypotension Safe test, but with low reproducibility. Indicated for patients suffering from renal failure.
Fludrocortisone test	Fludrocortisone 0.1 mg every 6 h for 4 days.	K^+^control every 6 h. Measure A and R at 10 h of the 5^th^day.	Positive A > 6 ng/dL with suppressed R.	Side effects: Hypertensive crisis, hypervolemia, and hypokalemia. Contraindicated for patients with severe HT, decompensated HF, renal failure, and severe hypokalemia. Considered the gold standard, but must be performed under hospital admission, since it is unfeasible in clinical practice.
Intravenous furosemide test	Administer furosemide 40 mg IV and stimulate 2 hours of walking.	Measure A, R and K^+^before and after 2 hours of intermittent walking.	Positive if PRA < 2 ng/mL/h or Renin < 13 uUI/mL.	Side effects: hypokalemia and hypotension. Advantage: well tolerated and easy to perform, ideal for patients with contraindications for sodium overload

*A: aldosterone; PH: primary hyperaldosteronism; PRA: plasma renin activity; R: renin.*

The most accurate imaging examination is the thin-slice CT, and MR provides no advantages. The goal of adrenal venous catheterization with concurrent blood sampling for aldosterone and cortisol is to identify the source of aldosterone secretion, and is considered the most accurate test to differentiate PH subtypes. It is recommended for patients with normal adrenal glands or bilateral abnormalities in CT scans.

In addition, adrenal venous catheterization is indicated in patients with small adrenal nodules (< 1.5 cm) and age 40 or older at HT diagnosis, as it may be a nonfunctioning adenoma. ^920^ ^-^ ^924^ The method is invasive and depends on the radiologist's experience. The treatment of choice for APA is unilateral adrenalectomy, preferably laparoscopic, unless contraindicated.

In hyperplastic HT, treatment consists of mineralocorticoid antagonists (spironolactone 50 to 400 mg/day). ^920^ ^-^ ^924^ The primary target of pharmacological treatment should be the renin blockade (in addition to blood pressure control and correcting hypercalcemia ) in order to lower the cumulative incidence of cardiovascular events. ^925^

### 15.4.2. Pheochromocytoma

Pheochromocytomas (PHEO) are catecholamine-secreting chromaffin-cell tumors of the sympathetic adrenomedullary axis. ^926^ Ten to 15% are extra-adrenal (paragangliomas), 10% are bilateral, and 15 to 20% are malignant (from 2 to 50%, depending on genetic defect). ^927^ The incidence rate for pheochromocytoma and paraganglioma is 0.6 cases per 100 000 persons-year.

The symptoms are the classic triad: headaches, profuse sweating, and palpitations with RHT/RfHT or paroxysmal HT (50%; hypertensive crises alternating with normal BP periods). The simultaneous presence of the classic triad and a hypertensive crisis has 89% diagnostic sensitivity and 67% diagnostic specificity. ^926^

Pheochromocytoma or paraganglioma diagnosis requires confirmation of excess catecholamine secretion and documenting the anatomy of the tumor. Laboratory diagnosis is based on blood and urine catecholamine metabolite levels. Free plasma metanephrine (metanephrine and normetanephrine) has 97% sensitivity and 93% specificity, ^926^ (LR: I; LE: A), but because of its higher cost, urine metanephrine isolated or associated with urine catecholamines (epinephrine, norepinephrine, and dopamine) is indicated. Though less sensitive, high urine catecholamine values (> 2 times the upper bound) indicate high diagnostic probability. ^242^ Urine metanephrine levels are more sensitive than urine catecholamine and vanillylmandelic acid for PHEO and paraganglioma diagnoses (ungraded recommendation). ^928^

In acute stress situations (acute illness, sepsis, AMI, decompensated HF) and the use of tricyclic antidepressants, antipsychotics agents, and levodopa, among others, it may be accompanied by increased catecholamine levels (usually < 2x the upper limit of normality). The medication should be suspended two weeks before sample collection to prevent false positives. The imaging tests to locate adrenal tumors are CTs (preferably; LR: 2; LE: B) and MNRs (hypersignal at T2 for PHEO), with 89% and 98% sensitivity, respectively. ^929^ An MRI is superior in the identification of paraganglioma or lymph node metastases (LR: I; LE: B). Whole-body scintigraphy using 123I-MIBG or 68Ga DOTATE-PET-CT is very effective at locating PHEOs and paragangliomas, metastases or multiple chromaffin-cell tumors (LR: IIa; LE: C). ^930^ ^,^ ^931^

The preferential treatment is minimally invasive surgery (LR: I; LE: B), and preoperative preparation should include alpha-1-blockers (doxazosin or prazosin) and adequate hydration with increased oral sodium intake for at least 2 weeks prior to surgery. ^932^ The chronic pharmacological treatment includes alpha1-blockers, BBs (only after beginning alpha1- blockers, in the presence of symptomatic tachycardia), CCBs, ACEIs, and central action agonists. ^932^ The paroxysmal hypertensive crisis of PHEO is an emergency, and should be treated with sodium nitroprusside or injectable phentolamine and volume replacement, if necessary. ^926^

Total and early removal of the neoplasm usually determines total remission of symptoms and cure of AH, in addition to preventing metastatic disease. ^927^ ^,^ ^929^ For malignant PHEO with unresectable metastases, systemic therapy with MIBG-131 is indicated. Cytotoxic chemotherapy is indicated in case of disease progression after high cumulative dose of MIBG-131 or in case of metastasis without MIBG uptake. Zoledronic acid is indicated to fight pain and lower fracture risk in patients with bone metastases. ^927^ ^,^ ^929^ Clinical, biochemical and radiological follow-up of patients is essential to detect malignant recurrences or metastases as well as other tumors in familial syndromes. ^242^

### 15.4.3. Hypothyroidism

The clinical signs of hypothyroidism are usually nonspecific, including fatigue, sleepiness and weight gain (mild in most cases). Patients with hypothyroidism have low levels of free thyroxine (T4) and high levels of thyroid-stimulating hormone (TSH), both screening tools for the condition (LR: IIa; LE: B). ^933^ In subclinical hypothyroidism, free T4 is normal and TSH is elevated. In hypothyroidism, there is higher risk of diastolic HT. ^934^ Hypothyroidism increases vascular resistance and extracellular volume, but BP increases are usually mild (< 150/100 mm Hg).

### 15.4.4. Hyperthyroidism

Hyperthyroidism increases cardiac output due to increased peripheral oxygen consumption and increased cardiac contractility. ^935^ Systolic HT is common, but HT prevalence depends on the severity of hyperthyroidism. Atrial fibrillation occurs in 10 to 20% of hyperthyroidism patients, and is more frequent in patients age 60 and over. ^936^ The most prominent clinical conditions are Graves' disease (palpitations, weight loss, exophthalmos, goiter, tremors of the extremities, warm skin, and heat intolerance, among other symptoms) or toxic adenoma, which may be more oligosymptomatic in older individuals with toxic multinodular goiters. Diagnosis is based on free thyroxine (T4) and thyroid-stimulating hormone (TSH) levels (LR: IIa; LE: B). usually, free T4 is high and TSH is suppressed. ^937^ In subclinical hyperthyroidism, free T4 is normal and TSH is suppressed. The presence of TSH antireceptor antibodies is diagnostic for Graves' disease, but may be absent in approximately 10% of cases.

### 15.4.5. Primary Hyperparathyroidism

The frequency of HT in patients with primary hyperparathyroidism ranges from 10 to60%. ^938^ Most patients with primary hyperparathyroidism are asymptomatic, while the rest may present with polyuria, polydipsia, osteoporosis, constipation, renal calculi, and HT. The mechanisms involved in HT are undefined, and there is no direct correlation between PHT levels and calcemia with severity of HT. In primary hyperparathyroidism, HT becomes more severe with impaired renal function due to hypercalcemia. Laboratory investigation involves testing calcemia (total and/or ionized calcium), phosphorus, PTH, and 24-hour urine calcium. ^939^ Vitamin D levels (especially if < 20 ng/dL) should also be measured and supplemented to rule out secondary hyperparathyroidism and vitamin D deficiency from normocalcemic primary hyperparathyroidism.

### 15.4.6. Cushing's Syndrome

Iatrogenic Cushing's syndrome (from the use of exogenous corticoids) is relatively common, unlike endogenous Cushing's syndrome, which is rare. Among endogenous causes, Cushing's syndrome (ACTH-secreting pituitary adenoma) is responsible for 85% of cases, while 15% are caused by adrenal hyperplasia or tumors (ACTH-independent causes). HT can be found in 75-80% of Cushing's syndrome patients. The mechanisms of HT are the cortisol-enhanced vasopressor effect of catecholamines, the effect of cortisol on mineralocorticoid receptors, and RAAS activation through increased liver production of angiotensinogen. Laboratory diagnosis of hypercortisolism uses baseline cortisol (useful to rule out exogenous use of dexamethasone or betamethasone), midnight salivary cortisol, and 24-h urinary cortisol, in addition to the dexamethasone suppression test (take 1 mg dexamethasone at 11 PM and measure serum cortisol level at 7-8 AM of the following morning). Radiological investigation should be based on adrenal CT scans or pituitary MRI for ACTH-dependent hypercortisolism. Imaging examinations should only be performed after clinical and laboratory diagnosis of hypercortisolism. Treatment of endogenous Cushing's syndrome depends on the etiology of the hypercortisolism. It can be managed surgically or with medications. ^940^

### 15.4.7. Obesity

Excessive visceral fat is accompanied by major hormonal, inflammatory and endothelial abnormalities. ^941^ All these mechanisms trigger a cascade of cytokine and adipokine release, increasing insulin resistance and determining RAAS and SNS hyperactivity, causing water and sodium retention and, consequently, HT and increased CV and renal risk. Countless studies have shown the close association between increased BP and weight gain. Adopting a weight loss strategy (see Chapter 8) is a key recommendation to lower BP and decrease CV risk as well as associated diseases, such as OSA. ^942^ ^,^ ^943^

From a practical standpoint, though it has been criticized for ignoring race/ethnicity, age, sex, and other parameters, obesity is categorized according to BMI (kg/m ^2^ ) as class 1. BMI 30 to < 35; class 2, BMI 35 to < 40; and class 3, BMI ≥ 40. Measuring abdominal circumference can also help diagnose central obesity. Further studies, such as bioimpedance and more accurate and very expensive imaging examinations may be performed, especially in clinical trials, such as dual-energy x-ray absorptiometry scanning (DEXA), CT and MNR. ^944^ ^-^ ^945^

### 15.4.8. Acromegaly

In approximately 98% of cases, sporadic or familial acromegaly is caused by growth hormone-secreting pituitary adenomas. Excess growth hormone (GH) stimulates hepatic secretion of insulin-like growth factor-1 (IGF-1), the cause of most clinical manifestations.

They are more frequent between ages 30 and 50 and can be divided into microadenomas (smaller than 1 cm) and macroadenomas (1 cm or larger). The second kind accounts for over 70% of tumors causing acromegaly. HT can occur in approximately 30% of cases and is multifactorial in nature, with a hydrosaline retention component, direct antinatriuretic effect of GH, RAAS and hyperactivity, and endothelial dysfunction, in addition to dysglycemia, left ventricular hypertrophy (LVH) and OSA. Other symptoms can be found in Chart 15.2 . ^947^

Laboratory assessment begins with serum IGF-1 and GH levels (LR: I; LE: B). Very low GH levels (below 0.4 ng/mL) rule out acromegaly, especially when associated with normal IGF-1 serum levels. GH level after glucose overload (75 g) can demonstrate the nonsuppression of GH secretion (LR: I; LE: B). IGF-1 levels and GH suppression testing after glucose overload are also employed to evaluate response to treatment. Sella turcica MNR is the best imaging examination to identify the tumor and, if contraindicated, may be replaced by a sella turcica CT scan (LR: IIa; LE: B). ^946^ ^-^ ^948^ Acromegaly treatment may involve surgical procedures, radiotherapy, and pharmacological treatment with somatostatin analogs, with octreotide, lanreotide, and cabergoline available in the Brazilian Unified Health System (SUS). ^949^

## 15.5. Pharmacological Causes, Hormones, and Exogenous Substances

It is a relatively common and underestimated cause of worsening HT or even its induction, frequently circumventable or reversible. A full history of all medications, drugs and supplements in use should be taken for all hypertensive patients. ^859^

Hypertensive mechanisms vary widely and may be multifactorial, such as volume retention (glucocorticoids, ketoconazole, oral contraceptives, androgen therapy, nonsteroidal anti-inflammatory drugs [NSAID]), sympathetic hyperactivity (decongestants, amphetamines, monoamine oxidase inhibitors [MAOIs], antidepressants, other psychiatric medications, cocaine, calcineurin inhibitors), and RAAS hyperactivity (immunosuppressants). ^950^ Good clinical practice dictates that hypertensive patients should be informed when combining medications can lead to worse blood pressure control. ^951^

Angiogenesis inhibition via endothelial vascular growth factor inhibition is an antineoplastic strategy applied in various oncology settings. Blood pressure increases, even acute ones, are a common side effect. ^952^ The mechanisms involved are the activation of the endothelin system, endothelial dysfunction, and capillary rarefaction. It is recommended that blood pressure be below 140/90 mm Hg before initiating this form of treatment and that BP monitoring continue throughout the therapy ^953^ (see Chart 15.8 ).

**Table:** 

Key Takeaways
In the absence of clinical signs suggestive of secondary hypertension in adults, the indications for additional assessment are resistant hypertension and early- or late-onset hypertension and/or sudden BP increase.
Major causes of secondary hypertension, both endocrine and nonendocrine, signs, and diagnostic and screening methods can be found in Table 15.2.
The most frequent cause of secondary hypertension is primary aldosteronism (PA). The aldosterone/renin ratio is the best initial test to determine the need for additional PA assessments. Paroxysmal HT with triad consisting of headaches, sweating, and palpitations is found in pheochromocytoma.
Renal artery stenosis should be investigated when creatinine levels increase ≥ 50% after ACEI or ARB use. Recent-onset severe HT occurs in individuals > 55 years old with abdominal murmur and a > 1.5 cm size difference in the contralateral kidney. HT is severe in patients suffering from atherosclerosis or recurrent pulmonary edema. In young adults with severe HT, fibromuscular dysplasia of the renal artery should be considered.
Other causes of secondary hypertension require more specific diagnostic methods, expert knowledge, and experience in interpreting results. Treatment should be directed by specialists from referral centers.

**Chart 15.8:** – Medications, hormones, and exogenous legal and illegal substances related to the development or increased severity of HT

MEDICATIONS		MECHANISMS
Immunosuppressants (calcineurin inhibitors)	Cyclosporin and tacrolimus	Increase prostaglandin synthesis and decrease excretion of Na^+^, H_2_O, and K^+^
**Nonsteroidal anti-inflammatory drugs (NSAID) and analgesics**	Cycloxigenase-1 and -2 inhibitors Acetaminophen	Decreased prostaglandins and Na^+^and fluid retention
**Sympathomimetics**	Nasal decongestants (ephedrine, pseudoephedrine, phenylephrine)	Stimulate the central nervous system
**Anorexigenic/satietogenic medications**	Amfepramone, sibutramine	Increase norepinephrine secretion
**Antidepressants and psychiatric medications**	Tricyclics, monoamine oxidase inhibitors (MAOI), lithium, fluoxetine, selegiline, carbamazepine, clozapinem buspirone, duloxetine, venlafaxine, and desvenlafaxine	Increased norepinephrine secretion, causing sympathetic hyperactivity
**Antifungal medicines**	Ketoconazole, amphotericin B	Fluid retention
**Ergot alkaloids**	Bromocriptine	
**Combination antiretroviral therapy (CART)**		
**VEGF (vascular endothelial growth fator) antineoplastic inhibitors**	Axitinib, bevacizumab, ponatinib, pazopanibe, regorafenib, sorafenib, sunitinib	Endothelial dysfunction and lower nitric oxide
**EXOGENOUS HORMONES**		
**Glucocorticoids**		Na^+^and fluid retention
**Human recombinant erythropoietin**		Abnormal production and sensitivity to endogenous vasopressor agents, direct vasopressor action, and arterial remodeling
**Sex hormones (estrogen-replacement therapy (conjugated estrogens and estradiol; oral contraceptives))**		Stimulate angiotensinogen production
**Growth Hormone (GH)**		Multifactorial
**EXOGENOUS SUBSTANCES**		
**Alcohol**		Sympathetic hyperactivity
**Amphetamines**		Sympathetic hyperactivity
**Cocaine**		Sympathetic hyperactivity
**Plant-based supplements**		
**Liquorice**		
**Ginseng**		
***Ginkgo biloba***		

## 16. Resistant And Refractory Hypertension

## 16.1. Definition and Classification

Resistant hypertension (RHT) is defined as office BP that remains ≥ 140/90 mm Hg with the use of three or more classes of antihypertensive medications with synergistic action, in maximum tolerated or recommended doses, one of which preferably a thiazide diuretic. When patients require four or more antihypertensive medications to achieve BP control, they are considered resistant but controlled hypertensives (BP < 140/90 mm Hg) ( Figure 16.1 ). ^164^ ^,^ ^504^ ^,^ ^564^ ^,^ ^954^

**Figure 16.1:** – Classification of hypertension according to number of antihypertensive medications and blood pressure control.

Refractory hypertension (RfHT) is defined as a subgroup of patients with true RHT that maintain uncontrolled BP (BP ≥ 140/90 mm Hg), even when using four or more antihypertensives, including spironolactone and a long-acting diuretic ( Figure 16.1 ). ^955^ Pseudo-resistant hypertension is defined as the failure to control BP related to white coat hypertension, failure at BP measurement, therapeutic inertia, or lack of adherence to prescribed pharmacological and nonpharmacological treatment regimens ( Figure 16.2 ). ^164^ ^,^ ^504^ ^,^ ^564^ ^,^ ^954^ Identifying patients with true RHT, therefore, requires ruling out pseudo-resistance and associated conditions ( Figure 16.2 ), making it essential to establish specific approaches. ^164^ ^,^ ^504^ ^,^ ^564^ ^,^ ^954^

**Figure 16.2:** – Classification of resistant hypertension.

Adapted from Malachias et al., 2016; ^164^ *Carey et al., 2018;* ^954^ *Yugar-Toledo, 2020;* ^504^ *Krieger et al., 2018.* ^564^ *RHT: resistant hypertension.*

## 16.2. Epidemiology of Resistant Hypertension

In population-based studies, RHT prevalence is estimated at 12 to 15% of the hypertensive population. ^164^ ^,^ ^504^ ^,^ ^564^ ^,^ ^954^ In Brazil, the multicenter ReHOT study ^564^ found an 11.7% prevalence rate for RHT. RfHT corresponds to 3.6% of resistant hypertensives. ^164^

The main clinical conditions and characteristics associated with RHT patients can be found in Chart 16.1 . ^164^ ^,^ ^504^ ^,^ ^564^ ^,^ ^954^ ^,^ ^956^ Worse prognoses for these patients is especially associated with the following factors: prolonged exposure to high blood pressure levels, end-organ damage, excess mineralocorticoids (aldosterone), and high sodium intake. ^164^ ^,^ ^504^ ^,^ ^564^ ^,^ ^954^ ^,^ ^956^

**Chart 16.1:** – Clinical conditions and characteristics associated with RHT

Clinical characteristics	Associated conditions
• Advanced age • Afro-Brazilians • Obesity • Higher SBP • Nondipper in ABPM • Hypervolemia (even with diuretics) • Excessive salt intake • Sedentary lifestyle • WCE (30%)	• Presence of LVH • DM • Metabolic syndrome • CRF • Albuminuria

*Adapted from Malachias et al., 2016;^*164*^Carey et al., 2018;^*954*^Yugar-Toledo, 2020;^*504*^Krieger et al., 2018;^*564*^Gaddan et al., 2008;^*957*^Shimosawa, 2013.^*958*^ABPM: ambulatory blood pressure monitoring; CRF: chronic renal failure; DM: diabetes mellitus; LVH: left ventricular failure; RHT: resistant hypertension; SBP: systolic blood pressure; WCE: white-coat effect.*

## 16.3. Pathophysiology

Just as the pathophysiology of primary hypertension is multifactorial, multiple factors may also be involved in the genesis of RHT and RfHT. This determines the various degrees of refractoriness to antihypertensive medications ( Figure 16.1 ).

RHT depends more on increased blood volume than RfHT due to persistent fluid retention, increased salt sensitivity, hyperaldosteronism, and renal dysfunction. In addition, the greater expansion of chest plasma, urinary aldosterone concentration, discrete suppression of renin activity^957^and high plasma aldosterone/renin ratio, as well as high levels of atrial and brain natriuretic peptide (BNP) are found in these individuals. ^958^ ^-^ ^960^ The ratio between volume and high blood pressure is the primary physiopathological basis shown in several studies ^955^ ^,^ ^957^ ^-^ ^960^ ( Figure 16.3 ) and justifies the use of diuretic to treat RHT patients.^961^

**Figure 16.3:** – Dominant physiopathological mechanisms in resistant hypertension.

HR: Heart rate. Refractory (uncontrolled) individuals with five classes of antihypertensives are included in the refractory group (3-5%).

In contrast, RfHT patients predominantly suffer from sympathetic nervous system hyperactivity and greater vascular stiffness. ^962^ Higher pulse wave velocity values, indicating elevated vascular stiffness and higher cytokine levels, such as tumor necrosis factor-α, ^963^ may mediate vascular damage in refractory hypertensive patients. ^964^ Other factors, such as age, obesity, obstructive sleep apnea (OSA), African-American descent, altered adipokines, endothelial dysfunction, and higher metalloproteinase-2 and -9 and adhesion molecule activity, are also involved in the process. ^504^ ^,^ ^954^ ^,^ ^895^ Gene polymorphisms, especially for RAAS and the endothelial nitric oxide synthase enzyme, have also been linked to RfHT ^965^ (Figures 16.4 and 16.5).

**Figure 16.4:** – Biomolecular systems measuring imbalance between increased aldosterone synthesis, sodium retention, OSA, increased RAAS activitity (AT1 and AT2 receptors) and hypertensive cardiac disease, and increased total vascular resistance, primarily induced by expanded plasma volume (salt retention and excess aldosterone) and sympathetic hyperactivity.

JG cells: juxtaglomerular cells; LV: left ventricle; OSAS: obstructive sleep apnea syndrome; Oxidized FA: oxidized fatty acids; RAAS: renin-angiotensin-aldosterone system; SNS: sympathetic nervous system; UAW: upper airways.

## 16.4. Diagnostic Investigation

Resistant hypertension patients should be treated by specialized HT services, capable of offering a multidisciplinary approach. Diagnostic investigation stands on four pillars: ^341^ ^,^ ^504^ ^,^ ^954^ ^,^ ^966^

- a) Pseudoresistance: Ruling out improper BP measurement (especially cuff width for obese patients), therapeutic inertia, poor adherence, and use of medications that increase BP (see Chapter 15). ^504^ ^,^ ^954^ ^,^ ^967^
- b) Assessment of cardiovascular (CV) risk factor, end-organ damage (EOD), and established CV disease: Once RHT is confirmed, diagnostic investigation should begin with specific tests, following this guideline for hypertension, to assess EOD impairment and secondary hypertension. The presence of associated comorbidities should be detected in specialized tests and examinations, following clinical suspicion.
- c) Ambulatory blood pressure monitoring (ABPM) and home blood pressure monitoring (HBPM): The diagnosis of RHT is based on office BP, ^504^ ^,^ ^954^ but out-of-office assessments (ABPM or HBPM) is essential to rule out the white-coat effect and masked hypertension. ^504^ ^,^ ^954^ ^,^ ^968^ Diagnostic and therapeutic management should be based on ABPM and HBPM levels. ^504^ ^,^ ^954^ ^,^ ^968^ ^,^ ^969^ Patients with daytime and/or nighttime high blood pressure levels (true RHT or masked HT) should have their medications adjusted and repeat ABPM afterward. ^504^ ^,^ ^954^ ^,^ ^968^ ^,^ ^969^ Patients with controlled BP on ABPM should have their therapy regimen maintained, regardless of the office BP levels. For these individuals, ABPM should be repeated annually or semiannually. ^968^ ^,^ ^969^ HBPM may also be used when available. Although it does not assess the nocturnal period and overestimates blood pressure levels, HBPM achieves moderate agreement on diagnosis, with high specificity and low sensitivity. ^970^
- d) Investigation of secondary causes: Secondary causes are more frequent for resistant than for nonresistant hypertension, and the most prevalent is OSA (80%), followed by hyperaldosteronism (6-23%), renovascular disease (renal artery stenosis) (2.5-20%), and renal parenchymal disease (2-10%). ^504^ ^,^ ^954^ ^,^ ^895^ Investigating altered thyroid function (1-3%) is also warranted. ^504^ ^,^ ^954^

## 16.5. Treatment ( Chart 16.2 )

**Chart 16.2:** – Treatment of resistant hypertension

Intervention	LR	LE
Prescribe and encourage LSCs	I	B
Optimize treatment with three medications: hydrochlorothiazide, chlorthalidone or indapamid,* ACEI or ARB, and CCB†	I	B
Add spironolactone as the 4^th^medication	I	A
Add a BB and/or clonidine as the 5^th^/6^th^medication^†^	IIA	B
Add direct-acting vasodilators sequentially	IIB	C
Prescribe the nocturnal administration of one or more drugs at bedtime	IIB	B
Check and improve adherence to treatment	I	C
Do not use invasive treatment, except in research protocols	III	B
* With glomerular filtration rate ≤ 30 mL/min or CHF, use loop diuretic.		

### 16.5.1. Nonpharmacological Treatment

All RHT patients should be directed and encouraged to adopt lifestyle changes ^971^ (see Chapter 8).

### 16.5.2. Pharmacological Treatment

The basic principle of pharmacological treatment is the combination of antihypertensive medications that act on most physiopathological mechanisms of BP increases: expanded intravascular volume, sympathetic and RAAS activation and increased peripheral vascular resistance. ^504^ ^,^ ^954^ ^,^ ^972^ Three-drug treatment should include a thiazide diuretic (DIU), a RAAS blocker (ACE inhibitor or angiotensin II AT1 receptor blocker (ARB)), and long-acting dihydropyridine calcium channel blocker (CCB), at full, well-tolerated doses and adequate intervals. In case of coronary artery disease, heart failure or tachyarrhythmias, a beta-blocker (BB) should replace the CCB in the initial three-drug therapy regimen.

The correct use of DIUs is essential for treating RHT: chlorthalidone (25 mg/day) or indapamide (1.5 mg/day) are the diuretics of choice, as long as the estimated glomerular filtration rate (eGFR) is above 30 mL/min. However, at the time of this writing, only hydrochlorothiazide was available in the Brazilian public health system. In patients with stage 4 or 5 chronic kidney disease or heart failure with fluid retention, loop diuretics (furosemide) should be used instead of thiazides and administered as needed for pressure and volume control. Spironolactone (aldosterone antagonist, 25 to 50 mg/day) is the medication of choice to be added as the 4^th^drug for patients with high adherence and true RHT. ^564^ ^,^ ^567^ For spironolactone-intolerant patients, amiloride (5 to 10 mg/day) may be used instead. ^973^

For patients with uncontrolled blood pressure after adding spironolactone to the treatment regimen, BBs (especially those with vasodilatory effect) or centrally acting alpha-antagonists (clonidine) ^564^ are the 5^th^/6^th^-line medications. If blood pressure control is still out of reach, direct-acting vasodilators (hydralazine and minoxidil) may be used as the 7^th^option ^974^ ^,^ ^975^

ABPM-guided chronotherapy, with at least one antihypertensive medication administered at night (especially RAAS blockers and BBs) improves blood pressure control and reverses nondipper patterns for RHT patients in addition to lowering cardiovascular morbidity and mortality. ^976^

Adherence to treatment is key for blood pressure control. However, up to 50% of patients with RHT partially or fully do not adhere to pharmacological treatment. ^977^

### 16.5.3. New Treatments

Various invasive treatments, such as endovascular renal sympathetic denervation, carotid baroreflex activation and modulation therapy, carotid body ablation, and central iliac arteriovenous anastomosis, have not been approved and are not to be used to treat resistant hypertensive patients, except as part of research protocols. ^5^ ^,^ ^978^

**Table:** 

Key Takeaways
Resistant hypertensives are individuals adhering to treatment involving three or more classes of antihypertensive medications in optimized doses who do not progress to controlled blood pressure.
Refractory hypertensives are those adhering to treatment with five or more classes of antihypertensive medications in optimized doses who have uncontrolled blood pressure.
In Brazil, the prevalence of true resistant hypertension is 11.7% (ReHot Study).
Resistant hypertension depends more on volume, while sympathetic hyperactivity predominates in refractory hypertension.
For the three initial classes of medication for resistant hypertension, the keys are using diuretics, blocking the renin-angiotensin-aldosterone system, and using direct-acting vasodilators.

## 17. Adherence to Antihypertensive Treatment

## 17.1. Introduction

The primary goal of initiating pharmacological and nonpharmacological antihypertensive treatment is to reduce the morbidity and mortality caused by high blood pressure (BP) levels. Though treatment has proven effectiveness and efficacy, hypertension (HT) control indices are still inadequate in most countries, including Brazil. ^979^ A country-level systematic review and meta-analysis performed at the primary care level showed that HT control rates ranged from 43.7% to 67.5%. ^980^ There are many reasons for the lack of hypertension control, but one of the most important is the lack of adherence to treatment, which can have various reasons.

## 17.2. Concept and Adherence

In a 2003 report, ^981^ the World Health Organization (WHO) defined adherence as “the extent to which a person's behaviour—taking medication, following a diet, and/or executing lifestyle changes, corresponds with agreed recommendations from a health care provider.” The primary reason for inadequate HT control seems to be nonadherence with long-term treatments, both for lifestyle changes (LSCs) and for taking the medications prescribed. In 2012, in a new WHO report, the authors differentiate the processes involved, such as adherence to medication and adherence management. ^982^ According to the those guidelines, adherence to medication is made of three major components: initiation, implementation, and discontinuation. Initiation is the time from prescription to taking the first dose of the medication; implementation corresponds to the coincidence between dose taken by the patient and dose prescribed; and discontinuation indicates the break in the process, when the next dose to be taken is skipped and the treatment is later interrupted. ^982^

Though there are various synonyms for adherence, such as compliance, conformity and capacitance, the proper term for HT treatment is still “adherence.” Adherence issues are often hard to detect and even harder to quantify. To improve HT control, it is important to combine efforts in order to identify patients who are not adhering to their treatment. Dropout rates are high in the first months of treatment, and patients may also not follow their prescription when taking medications. This situation has been described in medical literature since the 1970s ^983^ and is still found in more recent reports. ^984^ ^,^ ^985^ Lack of adherence to treatment is frequently defined as hypertensive patients taking less than 80% of prescribed medications. However, the spectrum of nonadherence from zero to over a 100% for those who take more than prescribed, which is also considered nonadherence to treatment.

## 17.3. Treatment Adherence Assessment Methods

There are many ways of measuring adherence to antihypertensive pharmacological treatment in clinical practice and in research, divided into direct methods, in which there is objective proof the patient has taken the medication, and indirect methods, where various strategies estimate whether the prescribed medication has or has not been taken. Choice of methods depends on for what purpose the information obtained will be used, the resources available for assessment, acceptance, how convenient the method is for the patient, and the costs involved. ^986^ Measuring adherence is a complex task. There is no single gold standard method capable of encompassing the various facets of the process. ^344^

The WHO suggests combining an indirect method and a direct one to measure adherence ^984^ for chronic illnesses. In HT, indirect methods wind up the most widely utilized, since direct methods still lack validation, are more expensive, and are only available in research environments.

Structured self-reported scales are widely used in clinical research, such as the Morisky-Green Medication Adherence Scale. The Eight-Item Morisky Medication Adherence Scale ^987^ evolved from a previous four-item scale, ^988^ is more reliable (α= 0,83 versus α= 0,61) and has been validated for Brazilian Portuguese. ^989^ The total score is classified as follows: 8 points mean high adherence; 6-7, medium adherence; below 6, low adherence. Another instrument is the three-question Qualiaids Medication Adherence Questionnaire (QAM-Q), created by Brazilian authors. In terms of accurately detecting nonadherence, QAM-Q has 62.5% sensitivity and 85.7% specificity, 74.1% area under the ROC curve, and 90.9% positive predictive value. ^990^

A review article on the subject of adherence and HT highlights the importance of increasing the availability and accessibility of more accurate measures to assess adherence. It also stresses that this is the main reason recent guidelines have emphasized the need to discuss medication adherence as a key issue in treating HT. ^991^ Chart 17.1 clarifies the advantages and disadvantages of several pharmacological treatment adherence assessment methods. ^992^

**Chart 17.1:** – Advantages and disadvantages of several pharmacological treatment adherence assessment methods

METHODS	ADVANTAGES	DISADVANTAGES
**DIRECT METHODS**		
Blood or urine testing	Objective and allows concentration of medication to be determined.	High cost. May be affected by biological factors and by “white-coat adherence”*
Adding markers	Objective and may be use in placebos in clinical research.	Requires high-cost quantitative assays and collection of body fluid samples.
Supervised administrations	Accurate.	Patients may hide pills under their tongue and discard them afterwards. Hard to deploy in outpatient settings for hypertensive patients, may be reserved for cases of resistant and refractory hypertension.
**INDIRECT METHODS**		
Structured adherence surveys (self-reported scales)	Simple, easy, cheap, and widely used.	Subject to error as interval between visits increases. Results may be distorted by patients.
Physician's impression	Easy and cheap.	Low sensitivity.
Manual pill count	Objective, quantifiable, and easy to execute.	Requires patient collaboration in returning the medication. Data may be altered by individuals.
Refilling prescriptions	Objective and easy data collection.	Requires computer applications and centralized pharmacies and record-keeping.
Clinical response	Simple and easy to perform.	Other factors may impact clinical response in addition to adherence.
Electronic devices	Accurate and identifies standards in measurements. Results are easily quantifiable.	High-cost method, requiring repeat appointments and processing data outputs.

* “White-coat adherence”: situation where patients have higher adherence to recommended treatments before medical appointments or collection of samples for laboratory tests.

## 17.4 Factors Interfering in Adherence to Treatment

Adherence to treatment is a complex, multidimensional process, with barriers divided into five different dimensions ( Chart 17.2 ) ^985^ ^,^ ^993^ ^-^ ^996^ that provide health care professionals with a more comprehensive perspective and enable to development of more effective interventions to improve BP control. Factors such as age, income, schooling and ethnicity/race play a major role in low socioeconomic status areas. The local health system and the nature of the health care staff may also influence adherence by hypertensive patients. In terms of disease and treatment, the most relevant factors are the chronic nature of HT and the absence of symptoms, the lifelong nature of the treatment and the complex drug regimen involved in some cases, and the undesirable side effects and drug interactions. Patient-related factors include disengagement with one's health issues and forgetting to take the medication.

**Chart 17.2:** – Factors interfering with adherence to antihypertensive treatment


**SOCIODEMOGRAPHIC FACTORS** • Sex; • Age; • Low educational level; • Low income; • Racial/ethnic minorities; • Access to transportation, distance and living in rural areas; • Pandemics and disaster conditions.
**FACTORS RELATED TO PHARMACOLOGICAL TREATMENT** • Lack of medications at health care centers; • Cost of purchasing medications; • Adverse effects; • Complex dosing regimens; • Improper therapy regimen; • Continuous and prolonged treatment.
**FACTORS RELATED TO HEALTH CARE SYSTEM AND STAFF** • Inadequate doctor/patient relationship; • Absence of multidisciplinary team; • Lack of individualized treatment; • Failure to identify nonadherence; • Ineffective communication; • Overloaded health care staff; • Outdatedness.
**PATIENT-RELATED FACTORS** • Denial of diagnosis; • Poor perception of treatment benefits; • Inadequate knowledge about the disease and the treatment; • Forgetting to take the medication; • Low motivation and self-esteem; • Fear of addiction and of adverse effects of medications.
**DISEASE-RELATED FACTORS** • Absence of symptoms; • Long-term complications; • Presence of other associated comorbidities; • Alcohol and drug abuse; • Impact on quality of life.

## 17.5. Strategies to promote adherence to antihypertensive treatment

The main consequence of lack of adherence to treatment is the lack of HT control and, therefore, increased end-organ damage (EOD) and cardiovascular (CV) morbidity and mortality. These, in turn, have major economic impact, in consequence of greater health care spending and early retirement. Therefore, adopting strategies to promote better adherence to antihypertensive treatment, either in isolation or in combination, as summarized in Chart 17.3 , intends to change that scenario. ^197^ ^,^ ^997^ ^-^ ^1006^

**Chart 17.3:** – Strategies to promote adherence to antihypertensive treatment


**PATIENT INTERVENTIONS** • Motivational strategies; • Home blood pressure monitoring (measuring BP at home); • Distance telemonitoring services; • Health education to promote self-care; • Use of pill dispensers and reminders; • Encourage social and family support; • Group education sessions; • Text messaging.
**INTERVENTIONS IN PHARMACOLOGICAL TREATMENT** • Avoid high doses in monotherapy; • Choosing medications with lower adverse events profile; • More convenient dosing regimens; •Single daily dose; •Two to three antihypertensives combined in a single pill; • Easy-to-understand prescriptions (handwritten or print); • Adjusting treatment for the patient's clinical and demographic characteristics(POC, older adults, women, the obese, diabetic patients).
**HEALTH CARE SYSTEM AND STAFF INTERVENTIONS** • Bonding with patients (having a fixed health care team); • Clear communication; • Calling patients who miss their appointments; • Home visits; • Having multidisciplinary teams (physicians, nurses, pharmacists, physical educators, nutritionists, psychologists, social workers, community health workers); • Facilitate access to medications.

The strategies with the best evidence available and which could be more feasibly implemented in Brazil include:

• Self-measured BP (Level of Recommendation I/Level of Evidence B);
• More convenient dosing regimens: lower possible doses, single daily dose, combination of antihypertensives in a single pill (Level of Recommendation I/Level of Evidence A);
• Deploying multidisciplinary teams to care for hypertensive patients, including physicians, nurses, pharmacists, physical educators, physical therapists, nutritionists, psychologists, social workers, and community health workers (Level of Recommendation I/Level of Evidence B).

## 17.6. Conclusion

Optimizing antihypertensive treatment adherence indices contributes to lower morbidity, mortality, and health care costs. Current therapeutic options include both pharmacological and nonpharmacological treatment regimens with proven effectiveness. Adherence to treatment plans and consequent hypertension control is still a major challenge in health care. Thus, combining efforts to met the actual needs of hypertensives has become the primary objective in the work of changing the current hypertension scenario.18. Perspectivas

**Table:** 

Key Takeaways
HT control rates in Brazil are still unsatisfactory. There are many reasons for the lack of hypertension control, but one of the most important is the lack of adherence to treatment.
Adherence to treatment is a complex, multidimensional process, where we can identify barriers connected to sociodemographic conditions, pharmacological treatment, health care systems, patients, and the disease itself.
Adherence issues are often hard to detect and even harder to quantify.
Measuring adherence is a complex task. There is no single gold standard method capable of encompassing the various facets of the process.
The strategies with the best evidence available and which could be more feasibly implemented in Brazil are: • Self-measured blood pressure (LR: I, LE: B); • More convenient dosing regimens: lower possible doses, single daily dose, combination of antihypertensives in a single pill (LR I/LE A); • Deploying multidisciplinary teams to care for hypertensive patients, including physicians, nurses, pharmacists, physical educators, physical therapists, nutritionists, psychologists, music therapists, social workers, and community health workers (LR I/LE B).

## 18. Perspectives

## 18.1. Introduction

The goal of this chapter is to discuss, based on evolving scientific knowledge about hypertension in the last few decades and more recent evidence, the possible advances and adjustments that will impact daily clinical practices and the challenges involved in diagnosis, treatment, and follow-up for hypertensive patients. It should be stressed that, unlike the previous chapters, where recommendations were rigorously classified by level of scientific evidence and level of recommendation, this section was designed to introduce possible rational vistas, based on the knowledge we have acquired thus far for this complex, multifactorial disease, with cardiovascular (CV), cerebral, and renal consequences that heavily determine morbidity and mortality, to the point that it has become the leading cause of death throughout the world.

## 18.2. Definition, Epidemiology, and Primary Prevention

With the gradual rise in life expectancy in both developed and developing countries, prevalence rates for hypertension (HT) are likely to increase even more. On average, there was a 1.4-year gain per decade of life in developed countries and a 1.2-year gain in Latin American countries from 1980 to 2011. ^1007^ It is widely known that, as we grow older, blood pressure levels increase, and from age 60 onward, systolic blood pressure (SBP) increases while diastolic blood pressure (DBP) decreases. This leads to higher pulse pressure (PP). These are important aspects when assessing risk factors and treatment strategies ( Figure 18.1 ). ^180^ ^,^ ^1008^

**Figure 18.1:** – Systolic and diastolic blood pressure behavior throughout life and by sex.

Source: Ji H et al., 2020. ^1008^

## 18.3. Blood Pressure and Vascular Damage

The increase in CV risk starting at SBP = 115 mm Hg and DBP = 75 mm Hg is well-known, ^78^ and BP measures remain the diagnostic marker of hypertensive disease. However, science is still trying to better understand biomarkers capable of early identification of vascular damage in hypertensive disease, even before the onset of higher BP values, as well as to develop their clinical applicability. The goal is to increase the accuracy of CV risk stratification in low- to moderate-risk individuals. ^82^ ^,^ ^139^ ^,^ ^1010^

Central systolic blood pressure assessments and arterial stiffness assessed by pulse wave velocity (PWV) are based on robust evidence with the goal of early identification of vascular damage and the ability to identify and restratify individuals initially classified as having low to moderate risk but who could actually be at high risk. In addition, PWV values above 10 m/s may indicate the presence of subclinical end-organ damage, ^156^ ^,^ ^298^ ^,^ ^1011^ and increased central systolic BP is a predictor of the development of hypertension. ^1012^ Another way of assessing vascular damage is the ability to identify lost or impaired endothelial function, as well as to understand its pathophysiology, which includes genetic predisposition and chronological aging, as well as changes in inflammatory and immune activity, insulin sensitivity, and cholesterol-rich lipoproteins. ^112^ ^,^ ^114^

Currently, the method most widely used to analyze in vivo endothelial function is flow-mediated dilation (FMD), but it remains restricted to research settings. ^118^ It is possible that, with new evidence in hypertensive and cardiovascular disease, the method will become more reliable and safe for use in clinical practice, further enhancing the early identification of vascular damage. ^1013^ ^,^ ^1014^

## 18.4. Cardiac Biomarkers

Though there has been great progress in the search for markers to estimate arterial damage, we cannot underestimate older tests that identify CV risk, such as electrocardiogram (ECG), magnetic resonance or, more recently, coronary calcium score, among others, for diagnosis of left ventricular hypertrophy (LVH). ^1015^ ^,^ ^1016^ There is robust evidence in favor of using B-type natriuretic peptides and N-terminal pro-B-type natriuretic peptides (NT-proBNP) as well as high-sensitivity Troponin T (hs-TnT), in risk stratification for fatal or nonfatal CV events and all-cause mortality. B-type natriuretic peptides are secreted by myocytes as counterregulatory response to pressure or volume overload of the myocardial wall, to increased sympathetic tone and to vasoconstriction, but also integrate CV stress and hemodynamic from multiple sources. ^1017^ Cardiac troponins are structural proteins in the contractile mechanism of heart muscle cells, secreted into circulation after cell damage. ^1018^

A recent study found that simply raising NT-proBNP and/or hs-TnT in prehypertensive patients enabled the identification of approximately 1/3 of those that who would later have CV outcomes or be admitted for heart failure (HF) within 10 years, individuals who could potentially benefit from pharmacological treatment. ^1019^ It has also been shown that NT-proBNP can estimate all-cause mortality and nonfatal CV outcomes for high-risk hypertensive diabetes patients in 2.6 years with the same predictive power as the whole set of the 20 most significant clinical and laboratory variables use most frequently, such as hs-TnT, age, albumin, history of HF, heart rate, history of stroke, HbA1c, smoking, LVH in ECG, ECG Q-waves, history of atrial fibrillation, any branch block in ECG, urine albumin/creatinine ratio, SBP, sex, history of coronary artery disease, low-density lipoprotein cholesterol, estimated glomerular filtration rate, insulin use, and DBP. ^1020^

## 18.5. Diagnosis and Classification

HT diagnosis, based on in-office measurement results and using proper techniques and devices, is defined as SBP ≥ 140 mm Hg and DBP ≥ 90 mm Hg. ^164^ The latest international guidelines recommend that HT diagnosis, whenever possible, be based on in-office blood pressure measurements, preferably unattended, or else by out-of-office measurements (ABPM and HBPM). In addition, there is a debate whether reference values to establish HT should be even lower or not. ^37^ ^,^ ^164^ ^,^ ^186^ ^,^ ^1021^

It seems clear that identifying HT, whether treated or untreated, by phenotype allows for risk stratification and for the definition of more individualized treatment strategies. ^180^ ^,^ ^212^ Another interesting topic is the use of self-measured blood pressure (SMBP) as a method for increasing patient engagement with their own illness and improving adherence to treatment, in addition to providing health care professionals with more information about the patient's everyday BP levels. ^1022^

The COVID-19 pandemic means acknowledging the global development of telemedicine techniques is critical. Apparently, distance monitoring through digital platforms and mobile applications for hypertensive patients has come to stay, facilitating conversations between health care teams and patients and the consequent information exchange and beneficial adjustments to lifestyle changes and even to treatment itself, with more focus on prevention and improving disease control. However, digital technology may further expand the scope of hypertension, following the lead of diabetes mellitus, enabling the development of increasingly accurate, continuous cuff-less blood pressure monitoring devices, syncing up with the smartphones that are now within reach for most of the population. ^388^ ^,^ ^1023^ ^,^ ^1024^

Finally, it is possible that in coming years, more attention given to patients with SBP ≥ 130 mm Hg and DBP ≥ 85 mm Hg (now classified as prehypertensive in these and in other guidelines) will change our understanding of HT diagnoses. ^1025^

## 18.6. Complementary Assessment and Cardiovascular Risk Stratification

The use of biomarkers for early identification of subclinical injuries, as well as higher CV risk, even at the early stages of high BP, brings with it the expectation that, for specific indications, individuals could and should receive early care for their conditions. ^139^ There are also biomarkers for vascular damage. For some, like the ankle-brachial index (ABI), calcium score and PWV, ^298^ ^,^ ^1011^ there is evidence in favor of the previous statements, though they are not widely available in clinical practice. Meanwhile, others, such as FMD, are still restricted to research settings. ^118^ Furthermore, researchers are currently working on a set of substances associated with inflammation that may ultimately be closely connected to endothelial dysfunction and to atherosclerosis, but which still require more robust evidence before they can be used in clinical practice. ^180^ ^,^ ^271^ ^,^ ^1014^ ^,^ ^1022^ ^,^ ^1026^

In terms of CV risk stratification, the progressive integration of biomarkers will allow us to more accurately establish the true risk level for each individual, especially for intermediate-risk hypertensive patients. Approaches of this sort offer the possibility of a more personalized medicine, with greater assertiveness in decisions connected to classification and treatment. ^156^ ^,^ ^298^ ^,^ ^1011^

In addition, in the field of precision medicine, usually based on genomics and metabolomics, there are already validated clinical scores capable of identifying patients at higher risk of early hypertension, as well as reference values for some of those biomarkers for the Brazilian population, adjusted for sex and age. The possibility of less sophisticated markers certainly works towards the improvements in accuracy we want to provide indications for specific assessments in clinically preselected patients, as is already usually the case in the investigation of secondary HT in the presence of clinical signs and positive screening tests. ^158^ ^,^ ^159^

## 18.7. Goals and Treatment

Following the scientific advancements and proofs discussed in the previous sections, it is reasonable to imagine that, in particular situations, early treatment onset and the pursuit of lower BP control targets may be indicated to prevent outcomes associated with increased BP and to minimize the so-called residual risk. ^307^ ^,^ ^1027^ In addition, the pharmacological treatment strategy based on two- or even three-drug combinations (at low doses), even at the early stages of the disease, should become increasingly prominent in guideline recommendations, while monotherapy may become an important strategy for individuals now classified as high-risk prehypertensives or those with changes in biomarkers ( Figure 18.2 ). ^307^ ^,^ ^1023^ ^,^ ^1028^ ^,^ ^1029^

**Figure 18.2:** – Drug octet for hypertension treatment.

Source: Feitosa et al., 2020. ^1028^ α1B: alpha-1-adrenergic antagonist; α2A: central alpha-2 agonist; BB: beta-blocker; BB, may be indicated before specific clinical conditions; CCB: dihydropyridine calcium channel blocker; DV: direct-acting vasodilator; RAASi: renin-angiotensin-aldosterone system inhibitor; TIAZ*: long-acting thiazide or thiazide-like diuretic up to 30 mL/minute of estimated glomerular filtration rate and in the absence of hypervolemia, else switch to loop diuretic.

It is possible that in the world of hypertension, according to current studies, our goal may be to control central and peripheral BP parameters, as long as that strategy can prove its ability to maximize reductions in major CV and renal outcomes. ^1030^ ^,^ ^1031^ Finally, though the prospect seems far-off, there is a real possibility that highly specific molecular tools will be available for HT treatment, such as RNA-mediated interference, which is simply post transcription gene silencing (PTGS) of overexpression of the protein of interest. ^388^ ^,^ ^1032^ Gene therapy for HT seems promising and has led to proven results in experimental studies involving the target-gene encoding the hepatic angiotensinogen. However, there is a known path for its clinical application after it is proven to be selective, effective and, above all, safe. Though many important perspectives are on the table, apparently the greatest challenge of all, in Brazil and worldwide, is much simpler. Its goals include improving diagnosis, adequate treatment, teamwork, and better blood pressure control in order to achieve significant reductions in renal and cardiovascular morbidity and mortality.
